# 2022 ACVIM Forum Research Abstract Program

**DOI:** 10.1111/jvim.16541

**Published:** 2022-10-10

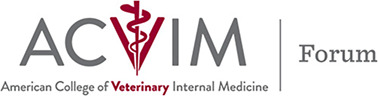

The American College of Veterinary Internal Medicine (ACVIM) Forum and the Journal of Veterinary Internal Medicine (JVIM) are not responsible for the content or dosage recommendations in the abstracts. The abstracts are not peer reviewed before publication. The opinions expressed in the abstracts are those of the author(s) and may not represent the views or position of the ACVIM. The authors are solely responsible for the content of the abstracts.

**2022 ACVIM Forum Research Abstract Program**

**June 22 – October 31, 2022**

**Research Abstract Oral Program**

**Index of Abstracts**

**Table:** 

**WEDNESDAY, JUNE 22**			
Time	#	Presenting Author	Abstract Title
**CARDIOLOGY**			
4:30 PM	C02	Andrew Chong	Retrospective Evaluation of Liver Parameters in Canine Degenerative Valvular Disease and Dilated Cardiomyopathy (ACVIM Resident Research Award Eligible & Cardiology Research Abstract Award Eligible)
4:45 PM	C03	William Clark	Assessing Myocardial Remodeling in Dogs with Myxomatous Mitral Valve Disease Using Cardiac Magnetic Resonance Imaging (ACVIM Resident Research Award Eligible & Cardiology Research Abstract Award Eligible)
5:00 PM	C04	Arianne Fabella	Comparison of Vascular Closure Techniques in Dogs Undergoing Percutaneous Transcatheter Intervention (ACVIM Resident Research Award Eligible & Cardiology Research Abstract Award Eligible)
**NEUROLOGY**			
4:30 PM	N01	Natalie Villani	Age, Breed and SOD1:c.118G Genotype Related Risk of Degenerative Myelopathy: An Update (ACVIM Resident Research Award Eligible)
4:45 PM	N02	Joseph Kowal	Electrical Impedance Myography in Healthy Dogs: Reference Values and Impact of Age (ACVIM Resident Research Award Eligible)
5:00 PM	N03	Joseph Kowal	Electrical Impedance Myography in Dogs with Degenerative Myelopathy (ACVIM Resident Research Award Eligible)
5:15 PM	N04	Patti Lawler	Comparison of Surgical Outcomes Associated with Compression Secondary to Hemorrhage and Intervertebral Disk Extrusions in Dogs (ACVIM Resident Research Award Eligible)
**ONCOLOGY**			
4:30 PM	O01	Margaret Duckett	Retrospective Evaluation of Melphalan, Vincristine, and Cytarabine Chemotherapy for the Treatment of Relapsed Canine Lymphoma (ACVIM Resident Research Award Eligible)
4:45 PM	O03	Andi Flory	Detection of Canine Cancer Prior to Clinical Signs with Blood‐Based “Liquid Biopsy”
5:00 PM	O11	Kai‐Biu Shiu	Collaborative Care Improves Client Perceptions When Managing Pets with Cancer
**SMALL ANIMAL INTERNAL MEDICINE ‐ ENDOCRINOLOGY**			
4:45 PM	EN01	Joanna Murdoch	Characterization of the Renin‐angiotensin‐aldosterone System in Telmisartan‐ or Enalapril‐treated Dogs with Proteinuric Chronic Kidney Disease (ACVIM Resident Research Award Eligible)
5:00 PM	EN02	Sarah Holm	Plasma Glucagon‐Like Peptide‐1 Concentrations in Dogs with Aminoaciduric Canine Hypoaminoacidemic Hepatopathy Syndrome: A Pilot Study (ACVIM Resident Research Award Eligible)
**SMALL ANIMAL INTERNAL MEDICINE ‐ HEPATOLOGY**			
5:15 PM	HP01	Tanner Slead	16S rRNA Amplicon Sequencing of Bile from Healthy Cats and Cats with Suspected Hepatobiliary Disease (ACVIM Resident Research Award Eligible)
**SMALL ANIMAL INTERNAL MEDICINE ‐ INFECTIOUS DISEASE**			
4:30 PM	ID01	Jennifer Chan	Incidence of Acute Kidney Injury in Dogs with Systemic Infections Treated with Amphotericin B (ACVIM Resident Research Award Eligible)
4:45 PM	ID02	Madeleine Stein	Assessment of Veterinary Technician Interest in and Barriers to Engagement with Hospital Antimicrobial Stewardship (ACVIM Resident Research Award Eligible)
**SMALL ANIMAL INTERNAL MEDICINE ‐ OTHER**			
4:30 PM	OT04	Tracy Hill	Evaluation of a Multiple‐mini Interview Format for Small Animal Internal Medicine Residency Programs
**SMALL ANIMAL INTERNAL MEDICINE ‐ RESPIRATORY**			
5:00 PM	R01	Jeremy Gallman	Infrared Thermography and 6MWT for Assessment of Thermoregulation in Dogs with Brachycephalic Obstructive Airway Syndrome (ACVIM Resident Research Award Eligible)
5:15 PM	R02	Jennifer Howard	Videofluoroscopic Swallow Study Diagnosis of Aerodigestive Disorders in Dogs (ACVIM Resident Research Award Eligible)
**EQUINE**			
4:30 PM	E02	Sarah Colmer	The Effect of Metformin on the Insulin Response to Oral Sugar in Insulin‐dysregulated Horses (ACVIM Resident Research Award Eligible)
4:45 PM	E10	Camilo Jaramillo‐Morales	Renal Dysplasia in Horses: 25 Cases (1991 ‐ 2021) (ACVIM Resident Research Award Eligible)
5:00 PM	E13	Kallie Hobbs	The Effect of Oral Trazodone on Ambulation and Recumbency in Horses (ACVIM Resident Research Award Eligible)
5:15 PM	E61	Natasha Williams	Short‐term Clinical Outcome and Racing Performance of Thoroughbreds Treated with Enrofloxacin as Juveniles: 146 Cases (ACVIM Resident Research Award Eligible)
**FOOD ANIMAL INTERNAL MEDICINE**		
4:30 PM	F01	David Martinez	Role of Nasal BRSV‐IgG1 Titers on Clinical Protection of Calves Against Experimental Challenge with BRSV (ACVIM Resident Research Award Eligible)
4:45 PM	F02	Meera Heller	Safety and Immunogenicity of Two Bovine Coronavirus Vaccines in Goats
5:00 PM	F08	William Crosby	Effect of Tulathromycin Metaphylaxis on Mannheimia Haemolytica isolation and Health Outcomes in Stocker Heifers
5:15 PM	F10	Santiago Cornejo	Bovine Myeloid Antimicrobial Peptide‐28 (BMAP‐28) mRNA Expression by Bovine Cells and Effects on Mannheimia haemolytica
**THURSDAY, JUNE 23**			
Time		Presenting Author	Abstract Title
**CARDIOLOGY**			
1:45 PM	C05	Luis Dos	Anti‐desmosomal Antibody as a Potential Biomarker of Arrhythmia Burden in Boxers with Arrhythmogenic Ventricular Cardiomyopathy (ACVIM Resident Research Award Eligible & Cardiology Research Abstract Award Eligible)
2:00 PM	C06	Luis Dos	Three‐Dimensional Echocardiographic Evaluation of Right Ventricular Function in Boxers with Arrhythmogenic Right Ventricular Cardiomyopathy (ACVIM Resident Research Award Eligible & Cardiology Research Abstract Award Eligible)
2:15 PM	C07	Leah Kruckman	Biologic Variability of Galectin‐3 and NT‐proBNP in Healthy Cats and Cats with Occult Hypertrophic Cardiomyopathy (ACVIM Resident Research Award Eligible & Cardiology Research Abstract Award Eligible)
2:30 PM	C08	Allison Masters	The Effect of Spironolactone on the Renin‐Angiotensin‐Aldosterone System in Dogs (ACVIM Resident Research Award Eligible & Cardiology Research Abstract Award Eligible)
3:00 PM	C09	Alice Morey	Evaluation of Cardiac Biomarkers and Point‐of‐care Ultrasound in Dogs with Non‐hemorrhagic Ascites (ACVIM Resident Research Award Eligible & Cardiology Research Abstract Award Eligible)
3:15 PM	C10	Sara Ostenkamp	Comparison of Single‐dose Pharmacokinetics in Dogs Receiving Vetmedin(R) and Two Formulations of Generic Pimobendan (ACVIM Resident Research Award Eligible & Cardiology Research Abstract Award Eligible)
3:30 PM	C11	Ashley Sharpe	Evaluating the Safety and Efficacy of Clevidipine in Dogs with Congestive Heart Failure (ACVIM Resident Research Award Eligible & Cardiology Research Abstract Award Eligible)
3:45 PM	C25	Sayaka Uematsu	Effect of Audible Static on Blood Pressure Measurements by Doppler Ultrasonic Sphygmomanometry in Cats (ACVIM Resident Research Award Eligible & Cardiology Research Abstract Award Eligible)
**NEUROLOGY**			
11:45 AM	N05	Arielle Ostrager	Survival in Dogs with Meningoencephalomyelitis of Unknown Etiology with and without Magnetic Resonance Imaging Lesions (ACVIM Resident Research Award Eligible)
12:00 PM	N06	Elizabeth DiPaola	Comparison of D‐dimer and Thromboelastography for Diagnosis of Cerebrovascular Accidents in Dogs (ACVIM Resident Research Award Eligible)
1:45 PM	N07	Suzanne Rosen	50‐step Walking Test’ ‐ A Pragmatic Outcome Measure for Recovery After Canine Spinal Cord Injury (ACVIM Resident Research Award Eligible)
2:00 PM	N11	Jenni Bridges	Prevalence and Clinical Features of Thoracolumbar Intervertebral Disc‐associated Epidural Hemorrhage in Dogs
2:15 PM	N12	Kiersten Forsyth	Physical Activity Is Associated with Better Cognitive Function: Results from the Dog Aging Project
2:30 PM	N13	Jacob Leslie	Frequency of MRI Abnormalities in a Population of Dogs Referred to Veterinary Neurologists
3:00 PM	N14	Alejandra Mondino	The Relationship Between Activity Patterns and Cognition, Pain, and Motivation in Senior Dogs
3:15 PM	N16	Hilary Wright	Evaluating the Benefits of Cannabidiol for Analgesia Following Surgery for Intervertebral Disc Herniation in Dogs
**NUTRITION**			
10:00 AM	NM05	Leslie Hancock	Acute Dietary Choline Chloride Toxicity in Cats: A Case Series Report
10:15 AM	NM06	Lara Sypniewski	Canine Non‐Traditional Diets Impact the Immunological Health of the Gastrointestinal Tract
10:45 AM	NM01	Katarina Yi	Effect of Fasting versus Feeding on Selected Biochemical Analytes in 100 Healthy Dogs (ACVIM Resident Research Award Eligible)
**ONCOLOGY**			
1:45 PM	O04	Shannon Kenny	Clinical Outcomes in Cats with Renal Carcinoma Undergoing Nephrectomy: A Retrospective Study (ACVIM Resident Research Award Eligible & Early Career Clinical Oncology Research Abstract Award Eligible)
2:00 PM	O06	Andi Flory	Lymphoma Detection Using Blood‐Based Liquid Biopsy
2:15 PM	O07	Jack O'Day	Alkaline Phosphatase Cytochemistry in the Diagnosis of Canine and Feline Primary Pulmonary Neoplasia (ACVIM Resident Research Award Eligible & Early Career Clinical Oncology Research Abstract Award Eligible)
2:30 PM	O08	Paolo Pazzi	Microthrombi Are Prevalent in Canine Carcinomas and Sarcomas and Positively Associated with Increased D‐Dimer Concentration
3:00 PM	O09	Aryana Razmara	Allogeneic NK Cells and Palliative Radiotherapy in the Treatment of Canine Cancer
3:15 PM	O10	Lucas Rodrigues	Clinico‐Genomic Data of Dogs with Malignant Oral Melanoma
3:30 PM	O13	Tim Williams	Investigation of Urinary Extracellular Vesicle‐Associated MicroRNAs in Dogs with Urothelial Carcinoma
3:45 PM	O12	Guannan Wang	Annotation of Canine Cancer Genomic Biomarkers Through Comparative Analysis of Human Mutations in COSMIC
**SMALL ANIMAL INTERNAL MEDICINE ‐ ENDOCRINOLOGY**			
11:30 AM	EN04	Gabriella Allegrini	Urinary Iodine Clearance Following Iodinated Contrast Administration in Normal Cats; Implications for 131I Use
11:45 AM	EN07	Petra Cerna	Validation of the Thyrotropin‐releasing Hormone Simulation Test in Healthy Cats
12:00 PM	EN08	Andrea Corsini	Total Thyroxine and Thyrotropin Concentrations During the Recovery from Non‐thyroidal Illness in Dogs: Preliminary Results
1:45 PM	EN09	Arnon Gal	Gut Microbial Whole Genome Gene‐networks and Metabolic Pathways Analysis in Diabetic Cats
2:00 PM	EN10	Arnon Gal	Subnetworks of Lipid‐derivatives and Endocrine Disrupting Compounds in Feline Hyperthyroidism
2:15 PM	EN11	Arnon Gal	Interim Analysis of a Prospective Clinical Trial of Fecal Microbial Transplantation in Diabetic Dogs
2:30 PM	EN12	Francesca Del	Hypothalamic‐pituitary‐adrenal Axis Recovery Following Intermediate‐acting Glucocorticoids Therapy in Dogs
3:00 PM	EN14	Valerie Nelson	Prevalence of Lower Urinary Tract Signs and Positive Urine Culture in Dogs with Diabetes Mellitus
3:15 PM	EN15	Mark Peterson	Relationship Between Urine Concentration and Development of Azotemia After Treatment of Hyperthyroid Cats with Radioiodine
3:30 PM	EN16	Fabio Teixeira	Investigating Hypercoagulability in Diabetic Dogs and Possible Omega‐3 Effect
3:45 PM	EN17	Heidi Ward	Efficacy and Tolerability of Generic Cinacalcet in Dogs with Primary Hyperparathyroidism
4:30 PM	EN18	Alice Watson	Immunohistochemical Markers of Aldosterone Production in Feline Primary Hyperaldosteronism
4:45 PM	EN19	Stephanie Scheemaeker	Organoids of Canine Medullary Thyroid Carcinoma and Feline Thyroid Adenomatous Hyperplasia (ESVE Award Winner)
**SMALL ANIMAL INTERNAL MEDICINE ‐ GASTROENTEROLOGY**			
10:00 AM	GI01	Elijah Ernst	Evaluation of 48h Gastric pH and Serum Gastrin Concentrations in Dogs with Chronic Kidney Disease (ACVIM Resident Research Award Eligible)
10:15 AM	GI02	Mariola Rak	Clinical and Gastrointestinal Changes in Healthy Research Dogs Administered Prednisone, Prednisone/omeprazole, or Prednisone/probiotics (ACVIM Resident Research Award Eligible)
10:30 AM	GI03	Florence Wootton	An Undernutrition Screening Score Is Associated with Treatment Response in Dogs with Inflammatory Protein‐losing Enteropathy (ACVIM Resident Research Award Eligible)
10:45 AM	GI04	Michael Hung	Serum Cobalamin and Methylmalonic Acid Concentrations in Dogs with Parvoviral Enteritis (ACVIM Resident Research Award Eligible)
11:15 AM	GI05	Alexandra Wood	The Impact of Fecal Identification Markers on the Feline Microbiome (ACVIM Resident Research Award Eligible)
11:30 AM	GI06	Leah Freilich	Effect of Nutrient Profile on Post‐Prandial GLP‐2 Plasma Concentration in Cats (ACVIM Resident Research Award Eligible)
**SMALL ANIMAL INTERNAL MEDICINE ‐ NEPHROLOGY/UROLOGY**			
2:00 PM	NU02	Sindumani Manoharan	Medical Dissolution of Presumptive Upper Tract Struvite Urolithiasis in 6 Dogs: (2012‐2018) (ACVIM Resident Research Award Eligible)
2:15 PM	NU03	Edward Vasquez	A Pilot Study Evaluating Renal Biomarker Changes Following Intravenous Iohexol Administration in a Small Population of Beagles (ACVIM Resident Research Award Eligible)
2:30 PM	NU04	Stephanie Skinner	Proteinuria at Time of Diagnosis is Associated with Shorter Survival Time in Dogs with Lymphoma (ACVIM Resident Research Award Eligible)
3:00 PM	NU07	Emily Belshin	Evaluation of the IDEXX SediVue Dx® for Identification of Canine and Feline Bacteriuria
3:15 PM	NU08	Amanda Blake	Altered Serum Amino Acid Concentrations in Dogs with Chronic Kidney Disease
3:30 PM	NU09	Nolan Chalifoux	Outcomes of Non‐Steroidal Anti‐Inflammatory Drug Toxicosis treated with Therapeutic Plasma Exchange in 62 dogs.
3:45 PM	NU10	Nolan Chalifoux	Outcomes of Fluid Therapy, Lipid Emulsion and Therapeutic Plasma Exchange for Non‐Steroidal Anti‐Inflammatory Drug Toxicosis
4:30 PM	NU11	Hilla Chen	Continuous Renal Replacement Therapy in Dogs with Acute Kidney Injury: Dose Prescription and Adequacy Assessment
4:45 PM	NU12	Ariana Cherry	Urinary miRNA 126 is Elevated in Dogs with Immune Complex‐Mediated Glomerulonephritis
5:00 PM	NU13	Candice Chu	Renal Single‐cell RNA Sequencing in Dogs With a Naturally Occurring Progressive Chronic Kidney Disease
5:15 PM	NU14	Larry Cowgill	Differentiation of Stable Kidney Function versus Progressive Dysfunction in Dogs
5:30 PM	NU15	Amanda Diaz	Evaluation of Alpha Enolase as a Marker of Renal Disease in Cats
5:45 PM	NU16	JD Foster	Population Pharmacokinetic Analysis of Enrofloxacin and Its Metabolite Ciprofloxacin in Cats with Reduced Kidney Function
1:45 PM	NU29	Jodi Westropp	Clinical Effects of Asymptomatic E. coli Administration Compared to Oral Antimicrobials for Canine Recurrent UTI
**SMALL ANIMAL INTERNAL MEDICINE ‐ OTHER**			
5:00 PM	OT02	Lisen Schortz	Quality and Timing Identified as Most Common Diagnostic‐related Incidents in Small Animal Veterinary Care
5:15 PM	OT03	Kristin Zersen	A Force‐Activated Separation Device Reduces the Rate of Intravenous Catheter Complications in Dogs
5:30 PM	OT09	Ana Clara	Efficacy and Safety of Long‐term Oral Imepitoin for Control of Canine Storm‐associated Anxiety and Fear
11:15 AM	OT10	Audrey Ruple	The PETSORT Statement: Reporting Guidelines for Randomized Controlled Trials Conducted in Dogs and Cats
**SMALL ANIMAL INTERNAL MEDICINE ‐ PHARMACOLOGY**			
11:45 AM	P01	Allison Bechtel	The Effect of Feeding on the Pharmacokinetics of Telmisartan Oral Solution in Dogs (ACVIM Resident Research Award Eligible)
12:00 PM	P02	Bonnie Purcell	Prednisolone Prescribing Practices for Dogs in Australia (ACVIM Resident Research Award Eligible)
**EQUINE**			
10:00 AM	E01	Natasha Williams	Investigating the Relationship Between Cardiac Function and Insulin Sensitivity in Horses (ACVIM Resident Research Award Eligible)
10:15 AM	E03	Brianna Clark	Evaluation of Commonly used Field‐Testing Protocols to Diagnose Insulin Dysregulation and the Association with Laminitis (ACVIM Resident Research Award Eligible)
10:30 AM	E07	Luiza Zakia	Culture‐enriched 16S RNA Sequencing Profile of Cecal Contents of Horses with and Without typhlocolitis (ACVIM Resident Research Award Eligible)
10:45 AM	E08	Sandra Diaz	Effect of Holding Time and Sampling Protocol on VCM‐Vet Parameters Using Fresh Equine Blood. (ACVIM Resident Research Award Eligible)
11:15 AM	E09	Laurence Leduc	Plasma Inflammatory Cytokine Profiles in Colitis Cases with and Without Equine Neorickettsiosis (Potomac Horse Fever) (ACVIM Resident Research Award Eligible)
11:30 AM	E11	Julianne White	Effects of Phenylbutazone, Firocoxib, and Dipyrone on Furosemide‐Induced Diuresis in Horses (ACVIM Resident Research Award Eligible)
11:45 AM	E12	Andrea Oliver	Effect of Ambulation Following 18F‐fluorodeoxyglucose Injection on Standing Positron Emission Tomography of the Equine Digit (ACVIM Resident Research Award Eligible)
12:00 PM	E14	Rachel Pfeifle	Multi‐dose Misoprostol Pharmacokinetics and Its Effect on the Fecal Microbiome in Healthy, Adult Horses (ACVIM Resident Research Award Eligible)
1:45 PM	E19	Francesca Freccero	Transthoracic Echocardiographic Parameters in Critically Ill Newborn Foals: Comparison with Healthy Foals
2:00 PM	E20	Antoine Premont	Systematic Review and Meta‐Analysis of Risk Factors for Conversion and Recurrence of Atrial Fibrillation
2:15 PM	E21	Demia de	Effect of a Single Intravenous Dose of Sirolimus on Insulin Dynamics in Healthy Adult Horses
2:30 PM	E22	Camilla Jamieson	Fluorescent Immunoassay‐Established Reference Intervals and Circannual Variation of Equine Plasma Adrenocorticotropic Hormone Concentrations in Qatar
3:00 PM	E35	Catarina Barros	Myenteric Ganglionitis Underlying Intestinal Motor Dysfunction in 3 Horses Diagnosed with Equine Herpesvirus 1 post‐mortem
3:15 PM	E36	Georgia Dollemore	Age and Breed Are Associated with the Requirement for Surgical Intervention for Left Dorsal Displacements
3:30 PM	E45	Emily Berryhill	Confirmed or Suspected Alloimmune Thrombocytopenia in Mule Foals
3:45 PM	E46	Rebecca Legere	Optimization of a mRNA Vaccine Candidate to Immunize Foals Against Rhodococcus equi
**FRIDAY, JUNE 24**			
Time		Presenting Author	Abstract Title
**CARDIOLOGY**			
1:30 PM	C13	I Ping Chan	Potential Predictors of Advanced Heart Failure Secondary to Myxomatous Mitral Valve Disease in Dogs
1:45 PM	C14	Matthew Denton	Development of a Deep Learning Model for Analysis of Narrow‐complex Heart Rhythms in Dogs
2:00 PM	C15	Laetitia Duler	Evaluation of Platelet Activation, Platelet‐leukocyte Interaction, and Hemostasis in Dogs with Pulmonary Hypertension
2:15 PM	C16	Jessica Gentile	Diagnostic Validation of a Vertebral Heart Score Machine Learning Algorithm for Canine Lateral Thoracic Radiographs
2:45 PM	C17	Arnaut Hellemans	Feasibility and Safety of High‐resolution Three‐dimensional Electroanatomical Mapping of the Complete Heart in Dogs
3:00 PM	C18	Emily Javery	Prospective Validation of a Quantitative Point‐of‐Care NT‐proBNP Assay for Screening Doberman Pinschers
3:15 PM	C19	Karin Kriström	Occurrence of Low Taurine Concentrations in a Swedish Population of Cocker Spaniels
3:30 PM	C20	Momo Kuo	An International Survey of Preferences for Echocardiographic Assessment of Left Atrial Size in Cats
4:30 PM	C21	Katherine Lopez	Evaluation of Cardiac and Endothelial Dysfunction in Canine Cancer Patients Treated with Toceranib Phosphate
4:45 PM	C22	Gitte Mampaey	Assessment of Cardiotoxicity After Combretastatin A4‐phosphate Administration in Dogs Using Two‐dimensional Speckle Tracking Echocardiography
5:00 PM	C24	Lisa Murphy	Multi‐Center Prospective Evaluation of Owner Medication Adherence for Feline Cardiovascular Disease in the Referral Setting
5:15 PM	C26	Michelle Vereb	Mitral Regurgitation Severity Index Predicts Outcome in Diverse Populations of Canine Myxomatous Mitral Valve Disease
**SMALL ANIMAL INTERNAL MEDICINE ‐ GASTROENTEROLOGY**			
9:30 AM	GI10	Amanda Blake	Altered Fecal and Serum Amino Acid Concentrations in Dogs with Chronic Enteropathy
9:45 AM	GI09	Sarah Au	Utility of Fluorescence Imitating Brightfield Imaging Microscopy for the Diagnosis of Feline Chronic Enteropathy
10:00 AM	GI08	Karin Allenspach	Fecal pancreatitis‐associated‐protein‐1 and Gastrotropin as Biomarkers for Subgroup Identification in Canine Chronic Enteropathies
1:30 PM	GI11	Johanna Holmberg	Prevalence and Clinical Features of Dogs with Chronic Enteropathy at Two Large Swedish Animal Hospitals
1:45 PM	GI12	Jamie Hui	Effects of Fecal Microbiota Transplantation on the Fecal Microbiome in Healthy Cats Administered Amoxicillin Clavulanate
2:00 PM	GI13	Albert Jergens	Efficacy of a Synbiotic‐IgY Supplement in Treatment of Canine Inflammatory Bowel Disease – Clinical Observations
2:15 PM	GI26	Chi‐Hsuan Sung	Fecal Bile Acids Profiles in Cats with Chronic Enteropathy
2:45 PM	GI15	Daniel Langlois	Evaluation of a Commercial Serologic Panel Marketed for the Diagnosis of Chronic Enteropathy in Dogs
3:00 PM	GI16	Alison Manchester	Efficacy of an Elemental Diet in Achieving Clinical Remission in Dogs with Chronic Inflammatory Enteropathy
3:15 PM	GI17	Nora Jean	To Sample or Not to Sample: Capturing Feline Fecal Microbiome Changes with High‐frequency Sample Collection
3:30 PM	GI19	Sina Marsilio	Metabolic Profiling of Serum Samples from Cats with Chronic Enteropathy
4:30 PM	GI18	Patricia Ishii	Evaluation of Intestinal Permeability in Dogs with Exocrine Pancreatic Insufficiency
4:45 PM	GI20	Matthew Salerno	Standardized Preparation of Canine Fecal Transplant Material Does Not Alter Microbial Community Structure
5:00 PM	GI21	Andrea Reisinger	Evaluation of Intestinal Barrier Dysfunction in Dogs with Acute Hemorrhagic Diarrhea Syndrome
5:15 PM	GI22	Denise Riggers	Intestinal S100/Calgranulin Expression in Cats with Chronic Enteropathies
5:30 PM	GI25	Helene Stübing	Comparison of Metronidazole versus a Synbiotic for Treating Dogs with Acute Diarrhea
5:45 PM	GI24	Jillian Smith	Feasibility Study Using Undercarboxylated Osteocalcin as a Surrogate for Serum Vitamin‐K in Chronic Enteropathy Dogs
**SMALL ANIMAL INTERNAL MEDICINE ‐ HEMATOLOGY**			
8:00 AM	HM01	Michael Barchilon	A Novel Flow Cytometric Assay to Assess Platelet Desialylation in Canine Immune Thrombocytopenia (ACVIM Resident Research Award Eligible)
8:15 AM	HM02	Larry Cowgill	Efficacy of Therapeutic Plasma Exchange (TPE) in Dogs with Immune‐mediated Hemolytic Anemia: A Case‐Controlled Study
8:30 AM	HM03	Arnaut Hellemans	Validation and Reference Interval of Activated Clotting Time in Dogs Using a Point‐of‐care Analyzer
8:45 AM	HM07	Keitaro Morishita	Evaluation of the Therapeutic Efficacy of Splenectomy in 20 Dogs with Non‐regenerative Immune‐mediated Anemia
9:15 AM	HM09	Janet Roque‐Torres	Effect of Antioxidant Supplementation on Oxidative and Storage Lesions in Canine Packed Red Blood Cells
**SMALL ANIMAL INTERNAL MEDICINE ‐ HEPATOLOGY**			
4:30 PM	HP02	Lara Baptista	Evaluation of Fasting Bile Acid Concentrations in Dogs with Sepsis
4:45 PM	HP03	Floris Dröes	Comparison of Bacteriological Culture with Molecular Methods for Identifying Bacteria in Liver Samples from Dogs
5:00 PM	HP04	Rommaneeya Leela‐arporn	Plasma Amino Acid Profiles in Dogs with Chronic Liver Disease
5:15 PM	HP05	Robert Phillips	Urine Isoprostane Concentrations in Dogs with Liver Disease
5:30 PM	HP06	Adrian Tinoco	Quantification of MicroRNAs 1275, 222, and 21 in Serum from Dogs with Chronic Hepatitis
**SMALL ANIMAL INTERNAL MEDICINE ‐ IMMUNOLOGY**			
9:30 AM	IM01	Romy Heilmann	Effect of Corticosteroid Administration on Serum C‐reactive Protein Concentrations in healthy Dogs
9:45 AM	IM02	Megan Slaughter	Evaluation for Anti‐Erythrocyte and Anti‐Platelet Antibodies in Healthy Dogs Administered Lokivetmab
10:00 AM	IM03	Anna‐Karina Weidinger	Immune Response of Cats After Simultaneous versus Separate Vaccination Against Rabies and Feline Leukemia Virus
**SMALL ANIMAL INTERNAL MEDICINE ‐ INFECTIOUS DISEASE**			
8:00 AM	ID04	Eric DiBiasio	Evaluation of MiQLab System to Detect Bacterial Pathogens and Antimicrobial Resistance Genes in Bacterial Isolates
8:15 AM	ID05	Andrew Hanzlicek	Evaluation of Anti‐Histoplasma Antibody Detection via Enzyme Immunoassay or Immunodiffusion in Cats and Dogs
8:30 AM	ID06	Christian Leutenegger	High Frequency of the Benzimidazole Resistance Genetic Marker in the Pet Dog Population in Florida
8:45 AM	ID07	Christian Leutenegger	Comparison of qPCR and Centrifugal Flotation for the Detection of Ancylostoma in experimentally Infected Beagles
9:15 AM	ID08	George Moore	Risk Factors for Acute Adverse Events in Dogs Following Vaccination
**SMALL ANIMAL INTERNAL MEDICINE ‐ OTHER**			
11:00 AM	OT05	Kiersten Forsyth	Prevalence of Owner‐reported Medical Conditions in the Most Popular Breeds in the Dog Aging Project
11:15 AM	OT06	Katharine Russell	Establishing a Frailty Phenotype for Aging Dogs
11:30 AM	OT07	Michelle Nguyen	Hepatic Dearterialization in Dogs and Cats with Massive or Diffuse Liver Tumor
11:45 AM	OT08	Cordelia Alexander‐Leeder	Medical Errors: The Experiences, Attitudes, and Perspectives of Incoming and Outgoing Fourth‐Year Veterinary Students
**SMALL ANIMAL INTERNAL MEDICINE ‐ PHARMACOLOGY**			
1:30 PM	P03	Jennifer Granick	Prevalence of Antibiotic Use for Cats and Dogs in U.S. Veterinary Teaching Hospitals, August 2020
1:45 PM	P04	Kara Maslyn	Evaluating the Effects of Telmisartan in Healthy Dogs as a Preclinical Model for Shar‐Pei Fever
2:00 PM	P05	Cindy Sotelo	Pharmacokinetics and Anti‐nausea Effects of Intravenous Ondansetron in Hospitalized Dogs Exhibiting Clinical Signs of Nausea
**SMALL ANIMAL INTERNAL MEDICINE ‐ RESPIRATORY**			
2:45 PM	R03	Julien Dandrieux	Development of a 3D‐printed Canine Airway Model as a Simulator for Canine Bronchoscopy
3:00 PM	R04	Elizabeth Rozanski	Evaluation of Sobetirome for Pulmonary Fibrosis in West Highland White Terriers
3:15 PM	R05	Aida Vientos‐Plotts	Metabolomic Profiling of Bronchoalveolar Lavage Fluid in Pet Cats with Asthma and Non‐asthmatic Respiratory Diseases
**EQUINE**			
4:30 PM	E49	Brandy Burgess	Impact of the Fecal Microbiome on Subclinical Salmonella Shedding in Horses
4:45 PM	E48	Surita du	Comparison of Microbroth Dilution and Disk Diffusion Methods for Antimicrobial Sensitivity of Equine Bacterial Isolates
5:00 PM	E53	Anna Chapman	Effect of a Supplement Containing Cannabidiol (CBD) on Sedation and Ataxia Scores and Health Parameter
5:15 PM	E54	Sonia Gonzalez‐Medina	Computed Tomographic Myelography in Horses with Cervical Vertebral Compressive Myelopathy
5:30 PM	E55	Francesca Freccero	Echocardiographic Assessment of Fluid‐responsiveness in Critically Ill Newborn Foals: A Pilot Study
5:45 PM	E63	Alessandro Migliorisi	Hyponatremia in Horses with Septic Lower Airway Disease Correlates with Severity of Inflammation

**Research Abstract ePoster Program**

**Index of Abstracts**

**Table:** 

**THURSDAY, JUNE 23**			
Time	#	Presenting Author	Abstract Title
**CARDIOLOGY**			
12:25 PM	C12	Lilian Shen	Genotyping, Echocardiographic Screening, and Whisker Papilla Fibroblast Collection in Purebred Maine Coon and Ragdoll Cats (ACVIM Resident Research Award Eligible & Cardiology Research Abstract Award Eligible)
12:25 PM	C27	Marlos Sousa	Serial Evaluation of Left Ventricular Systolic Function in Dogs Undergoing Chemotherapy with Doxorubicin
12:25 PM	C31	Annie Showers	Retrospective Evaluation of the Effect of Sotalol on Survival in Dogs with Severe Subaortic Stenosis
12:25 PM	C43	Tomoya Morita	Intrarenal Venous Flow Analysis by Ultrasound in Dogs with Heart Disease
12:45 PM	C28	Takuma Aoki	Procedural Surgical Protocol for Canine Mitral Valve Repair
12:45 PM	C32	Arane Takahashi	Effect of Autologous Blood Collection for Mitral Valve Repair on Hemodynamics and Cardiac Function
12:45 PM	C36	Logan Funk	International Evaluation of Clinical Characteristics and Outcomes in 137 Dogs with Reverse Patent Ductus Arteriosus
12:45 PM	C37	Fabio Gava	Multivariate Statistical Analysis in the Screening of Subclinical Hypertrophic Cardiomyopathy Phenotype in Domestic Cats
12:45 PM	C44	Seiya Niimi	High Prevalence and Clinical Features of Mitral Regurgitation in Young Chihuahuas Without Heart Murmurs
1:05 PM	C29	Charlotte Donnan	Retrospective Evaluation of Sacubitril/valsartan (Entresto) in 20 Dogs with Advanced Congestive Heart Failure
1:05 PM	C33	Emma Weitzhandler	Tissue Renin‐angiotensin System Enzyme Activity in Canine Post‐mortem Myocardial and Kidney Samples
1:05 PM	C38	Fabio Gava	Hypertensive Cardiomyopathy in Dogs: Echocardiography and Sex Differences
1:05 PM	C41	Emily Javery	Validation of a Point‐of‐Care Quantitative Assay for Feline NT‐proBNP
1:05 PM	C45	Dmitrii Oleynikov	Plasma Serotonin, Endothelin and VEGF‐D: A Differential Marker of Pre‐ and Postcapillary Pulmonary Hypertension
1:25 PM	C30	Laura Letwin	Case Series of Six Dogs with Primary Tricuspid and Right Ventricular Outflow Tract Neoplasms
1:25 PM	C34	Harry Cridge	Cardiovascular Abnormalities in Dogs with Acute Pancreatitis
1:25 PM	C39	Fabio Gava	Mitral Annular Plane Systolic Excursion in Dogs with Systemic Arterial Hypertension
1:25 PM	C42	Hyeon‐Jin Kim	Cartilage Intermediate Layer Protein 1 as a Novel Biomarker for Canine Myxomatous Mitral Valve Degeneration
**NEUROLOGY**			
9:25 AM	N08	Teryn Bouche	Current Practices in the Diagnosis and Management of Dogs with Degenerative Myelopathy: A Questionnaire‐Survey (ACVIM Resident Research Award Eligible)
9:25 AM	N10	Julianna Sabol	Thoracic Vertebrae: Proximity to Vital Structures and Implantation Corridor Measurements (ACVIM Resident Research Award Eligible)
9:25 AM	N18	Yeon Chae	Expression of Hyperphosphorylated Tau in Dogs with Immune‐mediated Meningoencephailitis
9:25 AM	N22	Lizabeth Lueck	Effects of Trazodone Administration on the Neurologic Examination in Healthy Dogs
9:25 AM	N24	Karen Munana	Functional Connectivity Magnetic Resonance Imaging in Dogs Anesthetized with Dexmedetomidine and Propofol
9:45 AM	N09	Lauren McAllister	A Novel Technique for Measuring Spasticity in Dogs After Acute Thoracolumbar Intervertebral Disc Extrusion (TL‐IVDE) (ACVIM Resident Research Award Eligible)
9:45 AM	N21	Dohee Lee	Neutrophil‐to‐Lymphocyte Ratio as a Diagnostic Marker in Dogs with Meningoencephalitis of Unknown Etiology
9:45 AM	N23	Christopher Mariani	Neurofilament Light Chain Concentrations in Dogs with Meningoencephalomyelitis
9:45 AM	N26	Jordan Schachar	Retrospective Review of Nerve Root Signature Associated with Cervical Intervertebral Disc Disease in Dogs
9:45 AM	N27	Amanda Valentino	Spinal Cord and Dural Sac Termination and Morphometry in Different Dog Breeds.
**NUTRITION**			
9:25 AM	NM02	Hannah Brodlie	Comparing Dried Blood Spot and Lateral Flow testing to Serum 25‐hydroxyvitamin D Concentrations in Cats (ACVIM Resident Research Award Eligible)
9:25 AM	NM08	Kathleen Gartner	Evaluating the Causes and Consequences of Canine Food Motivation Within the Dog Aging Project Pack
9:25 AM	NM10	Pooja Gupta‐Saraf	Low Carbohydrate Diet Reduces Circulating Inflammatory Gene Expression in Dogs with Chronic Gastroenteritis
9:45 AM	NM03	Kelsey Johnson	Hyperhomocysteinemia and Oxidative Stress in Greyhound Dogs (ACVIM Resident Research Award Eligible)
9:45 AM	NM09	David Griffin	Efficacy and Tolerability of a Novel Phosphate Binder Supplement in Cats
9:45 AM	NM11	Fabio Teixeira	Implications of COVID‐19 Pandemic in the Feeding Habits of Dogs Domiciled in São Paulo, Brazil
**ONCOLOGY**			
4:05 PM	O15	Hyung‐Kyu Chae	Anti‐cancer Effects of Oral Paclitaxel Against Canine Mammary Gland Cancer
4:05 PM	O16	Jeong‐Hwa Lee	Anticancer Activity of IRAK‐4 Inhibitors Against Canine Lymphoid Malignancies
4:05 PM	O17	Andi Flory	Evaluation of a Novel Blood Test for the Detection of “Difficult to Diagnose” Cancers
4:05 PM	O18	Ye‐In Oh	Identification of the Potential Candidate Genes and Signaling Pathways Involved in Lymphoma Progression in Dogs
4:05 PM	O19	Ester Yang	Pilot Study of Partial Ablation with Mechanical High‐Intensity Focused Ultrasound (Histotripsy) in Dogs with Spontaneously Occurring Soft Tissue Sarcomas (VCS Award Winner)
**SMALL ANIMAL INTERNAL MEDICINE ‐ ENDOCRINOLOGY**			
12:25 PM	EN03	Lydia Peña	Effect of Ethylenediaminetetraacetic Acid and Magnesium Chloride on Measurement of Canine Adrenocorticotropic Hormone (ACVIM Resident Research Award Eligible)
12:25 PM	EN23	Nicola Steers	Evaluation of a Flash Glucose Monitoring System in Diabetic Cats.
12:45 PM	EN20	Jeremy Evans	Variability Between Two Flash Glucose Sensor Locations in Non‐Diabetic Dogs During Rapidly Induced Hypoglycemia
1:05 PM	EN21	JoAnn Morrison	Geographic Prevalence of Naturally Occurring Feline Hyperthyroidism
1:25 PM	EN22	Andrew Narwold	Diagnosis of Canine Hyperadrenocorticism Using a Point‐of‐Care Cortisol Assay
**SMALL ANIMAL INTERNAL MEDICINE ‐ GASTROENTEROLOGY**			
6:05 PM	GI07	Charles Jones	Relationship between Magnesium, Calcium, and Parathyroid Concentrations in Dogs with Decreased 25(OH)D and Chronic Enteropathy (ACVIM Resident Research Award Eligible)
6:05 PM	GI28	Yu‐An Wu	A Randomized Non‐Controlled Open‐Label Trial in Cats Comparing Cyclosporine and Prednisolone for Treating Chronic Pancreatitis
6:05 PM	GI32	Ana Rita Pereira	Food‐responsive Enteropathy Occurrence in Dogs with Chronic Enteropathy in São Paulo‐Brazil.
6:25 PM	GI29	Yu‐An Wu	Histopathologic Examination of Serial Pancreatic Sections from Shelter Cats
6:45 PM	GI30	Jane Yu	Characterizing the Serum Proteome of Cats with Chronic Enteropathy
6:45 PM	GI31	Holly Ganz	Microbiome Responses to Fecal Microbiota Transplantation in Cats
6:45 PM	GI34	Harry Cridge	Oxidative Stress in Dogs with Acute Pancreatitis
**SMALL ANIMAL INTERNAL MEDICINE ‐ HEMATOLOGY**			
12:25 PM	HM10	Harry Cridge	Retrospective Analysis of Immunosuppressive and Anti‐thrombotic Protocols in Canine Non‐associative Immune‐mediated Hemolytic Anemia
12:45 PM	HM11	Yishan Kuo	Differences in Hematological Parameters Between Dogs with Congenital Intrahepatic and Extrahepatic Portosystemic Shunts
1:25 PM	HM13	Mei Sugawara‐Suda	Comprehensive Protein and Gene Expression Analysis of Spleen from Dogs with Non‐regenerative Immune‐mediated Anemia
**SMALL ANIMAL INTERNAL MEDICINE ‐ INFECTIOUS DISEASE**			
12:25 PM	ID03	John Shamoun	Effect of Urine Concentration on the Growth of Canine Uropathogens: An in Vitro Study (ACVIM Resident Research Award Eligible)
12:45 PM	ID09	Acácio Pacheco	Molecular Identification of Hemoparasites of Dogs in the Western Amazon
1:05 PM	ID10	Laura Rayhel	Proteinuria in Dogs with Pulmonary Coccidioidomycosis
1:25 PM	ID11	Andrea Scorza	Differentiating Giardia Duodenalis Assemblages with a Novel Beta‐giardin PCR Assay
**SMALL ANIMAL INTERNAL MEDICINE ‐ NEPHROLOGY/UROLOGY**			
6:05 PM	NU05	Sarah Lorbach	Evaluation of Health‐Related Quality of Life in Cats with Chronic Kidney Disease (ACVIM Resident Research Award Eligible)
6:05 PM	NU17	Sheng‐hui Huang	Urinary Glutathione Peroxidase 4 in Cats with Naturally Occurring Chronic Kidney Disease
6:25 PM	NU06	Olivia Murray	Safety and Efficacy of Nightly, Prophylactic Nitrofurantoin in 14 Dogs with Recurrent Urinary Tract Infections (ACVIM Resident Research Award Eligible)
6:25 PM	NU18	Sheng‐hui Huang	Non‐Transferrin‐Bound Iron in Cats with Naturally Occurring Chronic Kidney Disease
6:45 PM	NU19	Megan Kelley	Diltiazem Infusion Alterations on Glomerular Filtration Rate, Electrolyte Excretion, and Urine Output in Healthy Dogs
6:45 PM	NU20	Tzu‐Chien Kuo	Urinary Angiotensin‐Converting Enzyme 2 Concentration and Activity in Cats with Naturally Occurring Chronic Kidney Disease
**SMALL ANIMAL INTERNAL MEDICINE ‐ OTHER**			
1:05 PM	OT11	Vanessa Wilkins	End of Life Survey ‐ Free Text Analysis
**SMALL ANIMAL INTERNAL MEDICINE ‐ PHARMACOLOGY**			
12:45 PM	P08	Kristine Fraatz	Plasma Pharmacokinetics of a Hypoxia‐Inducible Factor Prolyl Hydroxylase Inhibitor (Molidustat) in Healthy Cats
1:05 PM	P09	Butch KuKanich	Fluconazole Has Variable Oral Systemic Absorption in Dogs
1:25 PM	P10	Amy Nichelason	Voluntary Acceptance of Compounding Flavors in Cats
**SMALL ANIMAL INTERNAL MEDICINE ‐ RESPIRATORY**			
1:25 PM	R06	Mark Nagel	Effectiveness of Pediatric Inhaler Chambers in Cats with Asthma
**EQUINE**			
6:05 PM	E04	Erin Elder	Assessment of the Hypothalamic‐pituitary‐adrenocortical Axis Function Utilizing a Vasopressin Stimulation Test in Healthy Foals (ACVIM Resident Research Award Eligible)
6:05 PM	E16	Amanda Craven	Clinical Findings and Outcome Predictors for Equine Multinodular Pulmonary Fibrosis: 46 Cases (2009‐2019) (ACVIM Resident Research Award Eligible)
6:05 PM	E23	Weerasekara Jayathilake	Equine Hyperinsulinemia Causes Tissue‐Specific Alterations of Cytokines and Acute Phase Proteins in a NDkB‐independent Manner
6:05 PM	E27	Toby Pinn‐Woodcock	Effects of Latitude, Age and Season on Equine Adrenocorticotropic Hormone Concentrations in the United States
6:05 PM	E29	Kathryn Timko	Effect of AMPK Agonists on Hepatic Lipid Content in Horses with Experimentally‐induced Insulin Dysregulation
6:25 PM	E06	Erin Pinnell	Attenuation of Post‐prandial Hyperglycemia by 5’‐adenosine Monophosphate‐activated Protein Kinase Agonists in Experimentally‐induced Equine Insulin Dysregulation (ACVIM Resident Research Award Eligible)
6:25 PM	E17	Kile Townsend	Developing a Tool to Detect and Track Laryngeal Dysfunction in Horses
6:25 PM	E24	Kate Kemp	Effect of Phenylbutazone Administration on Insulin and Glucose Dynamics in Horses
6:25 PM	E28	Kristen Thane	Comparison of a Newly Developed Glycemic Pellets Challenge with the Oral Sugar Test in Horses
6:25 PM	E30	Kathryn Timko	Effect of High‐carbohydrate Feeding and Corticosteroid Administration on Lipid Content of Equine Liver
6:45 PM	E18	Daniela Bedenice	Seasonal Assessment of Serum 25‐Hydroxyvitamin‐D concentrations in Healthy and Asthmatic Horses
6:45 PM	E26	Erik Peterson	Seasonal Effects of Plasma ACTH in Horses and Donkeys Residing Near the Equator
6:45 PM	E34	Hailey Maresca‐Fichter	Dynamic Insulin Responses to High Carbohydrate Diet Acclimation in Normal and Insulin Dysregulated Horses
6:45 PM	E37	Cosette Ayoub	Fecal Microbiota of Diarrheic Horses and Its Association with Laminitis
**FOOD ANIMAL INTERNAL MEDICINE**			
4:05 PM	F03	Luis Rivero	Blood Cultures and Outcomes in Sick Neonatal Beef Calves (ACVIM Resident Research Award Eligible)
4:05 PM	F04	Samantha Haw	Pathogen‐Specific Intramammary Infection Prevalence, Persistence, and Somatic Cell Count Association in Lactating Jersey Cows (ACVIM Resident Research Award Eligible)
4:05 PM	F05	Osman Safa Terzi	Left Ventricular Systolic Function in Neonatal Calves With Diarrhea
4:05 PM	F06	Daniela Bedenice	The Association Between Fecal Microbiota, Endoparasitism and Age of Adult Alpacas
**FRIDAY, JUNE 24**			
Time		Presenting Author	Abstract Title
**CARDIOLOGY**			
10:25 AM	C40	Fabio Gava	QT Interval, Electrolyte and Acid‐Base Evaluation in Dogs with Chronic Kidney Disease
10:25 AM	C51	Mutsuki Umezawa	Serum Carnitine Profile of Cats with Hypertrophic Cardiomyopathy
10:25 AM	C53	Bradley Whelchel	Peripheral Edema in Dogs: Clinical Characteristics and Etiologies
10:45 AM	C50	Selena Tavener	Up‐regulation of STATs and IRF‐1 in Canines with Heart Murmur and Associated Valvular Cardiovascular Disorder
10:45 AM	C52	Sonya Wesselowski	Taurine Concentrations in Cavalier King Charles Spaniels: Reference Intervals, Diet, and Mitral Valve Disease Effects
10:45 AM	C57	Kentaro Kurogochi	Transesophageal Echocardiography‐Related Complications During Mitral Valve Repair in Dogs
**SMALL ANIMAL INTERNAL MEDICINE ‐ HEPATOLOGY**			
12:10 PM	HP08	Emily Jachec	Clinical Use of Ursodiol in Feline Medicine
**SMALL ANIMAL INTERNAL MEDICINE ‐ IMMUNOLOGY**			
12:10 PM	IM04	Sidney Bannister	Relapse Risk Factors for Immune Mediated Hemolytic Anemia: A Retrospective Study of 223 Dogs
12:30 PM	IM05	Nida Chornarm	Use of a Flow Cytometry Assay to Detect Anti‐erythrocyte Antibodies in 116 Anemic Client‐owned Dogs
12:50 PM	IM06	Po‐Yu Liu	The Intestinal Microbiome of Dogs with Immune‐mediated Hemolytic Anemia or Immune Thrombocytopenia
1:10 PM	IM07	Ye‐In Oh	Romiplostim in Primary and Secondary Thrombocytopenia: A Retrospective Study of Dogs and Cats
1:10 PM	IM08	Jade Peralta	Anti‐oxidative and Immunomodulatory Effects of Telmisartan in Healthy Cats
**SMALL ANIMAL INTERNAL MEDICINE ‐ NEPHROLOGY/UROLOGY**			
12:10 PM	NU23	Madison McKay	Electrophoretic Urine Protein Banding Patterns as a Diagnostic Biomarker for Canine Membranoproliferative Glomerulonephritis
12:10 PM	NU32	André Vieira Le Sueur	Evaluation of Novel Renal Injury Markers in Dogs with Ehrlichiosis.
12:30 PM	NU24	Isabelle Merindol	Benign Feline Ureteral Obstruction: Outcome of Cats Undergoing Medical Management
12:30 PM	NU30	Stacie Summers	Untargeted Metabolomic Profiling of Serum from Cats with Chronic Kidney Disease
12:30 PM	NU33	André Vieira Le Sueur	C‐Reactive Protein and Canine Ehrlichiosis: A New Clinical Perspective
12:50 PM	NU21	Catherine Langston	Pilot Field Study of Hypoxia‐Inducible Factor Prolyl Hydroxylase Inhibitor in Chronic Kidney Disease‐Associated Anemic Cats
12:50 PM	NU31	Selena Tavener	Reduced Antioxidant System and Increased Inflammation in Canines with Chronic Kidney Disease
12:50 PM	NU35	Taesik Yun	Serum Concentrations of Leptin and Adiponectin in Dogs with Chronic Kidney Disease
1:10 PM	NU22	Crystal Ma	Apolipoprotein B100 Is a Potential Urine Biomarker for Membranoproliferative Glomerulonephritis in Dogs with Protein‐losing Nephropathy
1:10 PM	NU27	Gilad Segev	Urinary Cystatin B Differentiates Progressive versus Stable Stage I Chronic Kidney Disease in Dogs
1:10 PM	NU34	Zhe (Alice) Wang	Effect of Tamsulosin on Urethral Tone in Healthy Male Cats
**EQUINE**			
12:10 PM	E32	John Haffner	The Effect of Trailering on Thyrotropin Releasing Hormone Stimulation of Adrenocorticotropic Hormone Concentration in Horses
12:10 PM	E40	Natalia Watrobska	Effect of Post‐Operative Reflux on Survival of Horses with Large Colon Volvulus
12:10 PM	E44	Katarzyna Dembek	The Fecal Bacterial Microbiota in Healthy and Sick Neonatal Foals
12:10 PM	E56	Dayna Jodzio	Pain Scoring Systems for Predicting Clinical Outcomes In Hospitalized Equine Ophthalmology Patients
12:10 PM	E60	Joanne Haughan	Detection of Oxycodone Metabolites in Plasma After Oral Administration to Horses.
12:30 PM	E33	John Haffner	A Combined Procedure to Identify Pituitary Pars Intermedia Dysfunction and Insulin Dysregulation in Horses
12:30 PM	E41	Cosette Ayoub	Post‐mortem Prevalence of Gastric Ulceration in Diarrheic Horses
12:30 PM	E50	Brandy Burgess	Use of CRISPR‐SeroSeq to Detect Multiple Salmonella Serotypes in Equine Fecal Samples
12:30 PM	E57	Mariano Mora‐Pereira	Effects of Equine Platelet Lysate on ex‐vivo Vasculogenesis
12:50 PM	E38	Jordan Flood	Right Dorsal Colitis in Horses: A Retrospective Study of 35 Cases
12:50 PM	E42	Marcio Costa	Bacterial Viability in Different Preparation Protocols of Fecal Microbiota Transplantation Solution
12:50 PM	E51	Sharanne Raidal	MALDI‐TOF Mass Spectrometry for Improved Identification of Equine Bacterial Isolates
12:50 PM	E58	Bianca Amiet	Transdermal EMLA (lidocaine/prilocaine) Cream for Intravenous Catheterisation in Horses
12:50 PM	E65	Kate Hepworth‐Warren	Utility of Serum Amyloid a in Monitoring Response to Antimicrobial Therapy in Equine Pneumonia
1:10 PM	E43	Marcio Costa	Bacterial Translocation in Horses with Colic Addressed by DNA Sequencing
1:10 PM	E52	Rebecca Legere	SARS‐CoV‐2 Pseudovirus Infects Equine Bronchial Epithelial Cells In Vitro
1:10 PM	E59	Jenifer Gold	Pharmacokinetics of Single Dose Administration of 20 and 40 mg/kg of Acetaminophen in Neonatal Foals
1:10 PM	E67	Berta Mozo Vives	Comparative Efficacy and Adverse Effects of Salbutamol and N‐butylscopolammonium Bromide in Horses with Severe Asthma
**FOOD ANIMAL INTERNAL MEDICINE**			
10:25 AM	F07	Ailbhe King	Minimum Colostral Immunoglobulin G Concentration Required for Pooling to Achieve Adequate Immunity in Dairy Calves
10:45 AM	F09	Diego Gomez	Fecal Microbiota of Diarrheic Calves and Its Association with Acid‐base Disorders
10:45 AM	F13	Kylie McLaughlin	The Pharmacokinetics and Clinical Efficacy of Levamisole in Adult Alpacas after Oral Administration

**ON DEMAND**		
#	Presenting Author	Abstract Title
**CARDIOLOGY**		
C23	Matheus Mantovani	Agreement Between Doppler Ultrasound and High‐definition Oscillometric Device for Systolic Arterial Pressure in Hospitalized Dogs
C35	Hunter Enderle	Chronic Myxomatous Valve Disease Prevalence and Pathologic Features. Canine Mitral, Tricuspid, Aortic, and Pulmonic Valves
C46	Tatsuyuki Osuga	Prognostic Value of Left Atrial Stiffness Estimated Using Echocardiography in Canine Myxomatous Mitral Valve Disease
C48	Anna Reuter	Clinical Outcome of Idiopathic Juvenile Ventricular Arrhythmias in 25 Dogs
C49	Ryohei Suzuki	Echocardiographic Comparison of Right Ventricular Function Between Dogs with Pre‐ and Post‐capillary Pulmonary Hypertension
C54	Yunosuke Yuchi	Effect of Beraprost on Hemodynamics and Cardiac Function in Dogs with Pulmonary Hypertension
C56	Yunosuke Yuchi	Echocardiographic Assessment of Cardiac Function in Dogs with Pulmonary Hypertension Secondary to Respiratory Disease
**NEUROLOGY**		
N15	Cecilia Tegner	Alpha‐Chloralose Poisoning in Cats: Clinical Presentation in 24 Confirmed Cases
N17	Gibrann Castillo	Electroencephalogram and Heart Rate Variability Changes After Transcutaneous Vagus Nerve Stimulation in Healthy Dogs
N19	Lea Henze	Ondansetron in Dogs with Vestibular Nausea: A Double‐blinded, Randomized, Placebo‐controlled Crossover Study
N20	Lea Henze	Towards a Mobile App for Automated Pain Assessment in Cats using Deep Learning
**NUTRITION**		
NM04	Olivia Chiu	The Effects of Storage Temperature and RNAlater Solution on the Canine Fecal Microbiota
NM07	Alexandra Gagnon	Repertoriating Raw Food Companies in Quebec for Domestic Animals
**ONCOLOGY**		
O02	Joseph DiBenedetto	Cytomorphologic Changes in Blood Leukocytes and Erythrocytes in Dogs Receiving CHOP Treatment for Multicentric Lymphoma
O05	Chen‐Si Lin	Fibrinogen Degradation Products as a Clinical Biomarker for Cancer Diagnosis and Prognosis in Neoplastic Dogs
O14	Anna Winner	Do Dogs Get Cancer‐Associated Thrombosis? Retrospective Analysis of Characteristics of Dogs with Thrombosis: 2014‐2019
**SMALL ANIMAL INTERNAL MEDICINE ‐ ENDOCRINOLOGY**		
EN05	Alisa Berg	Assessment of the FreeStyle Libre 2 Interstitial Glucose Monitor in Hypo‐ and Euglycemia in Cats
EN06	Alisa Berg	Sterility of Refrigerated, Multidose Insulin Vials Through 6 Months of Use
EN13	Chen Gilor	A Modified Oral Glucose Tolerance Test Detects Mild Dysglycemia Caused by Capromorelin
**SMALL ANIMAL INTERNAL MEDICINE ‐ GASTROENTEROLOGY**		
GI14	Aarti Kathrani	Characterisation of the Culturable Duodenal Mycobiota of Dogs with Chronic Enteropathy
GI23	Kenneth Simpson	Placebo Controlled Trial of Hydrolyzed Fish Diets in Dogs With Chronic Enteropathy
GI27	Melanie Werner	Prevalence of Clostridioides difficile in Canine Feces and Its Association with Dysbiosis
GI33	Laura Van Vertloo	Retrospective Evaluation of the Risk of Gastrointestinal Bleeding in Dogs Receiving Ophthalmic Non‐steroidal Anti‐inflammatory Drugs
**SMALL ANIMAL INTERNAL MEDICINE ‐ HEMATOLOGY**		
HM04	Matthew Kornya	Investigation of Novel Hemostasis Parameters and Closure Curves on the Platelet Function Analyzer‐200 in Cats
HM05	Matthew Kornya	Validation of Shipping of Feline Blood Samples for Analysis on the Platelet Function Analyzer 200
HM06	Matthew Kornya	Validation of the ProCyte Dx and Visual Slide Review with the Plateletworks System in Cats
HM08	Erin Phillips	Assessing Methods to Monitor Rivaroxaban Therapy in Hypercoagulable Dogs
HM12	Kaitlyn Rank	Evaluation of Thrombin Generation as a Novel Antiplatelet Therapeutic Monitoring tool in Dogs administered Clopidogrel
**SMALL ANIMAL INTERNAL MEDICINE ‐ HEPATOLOGY**		
HP07	Tarini Ullal	Demographic and Histopathologic Associations with Elevated Hepatic Copper Concentrations in Dogs
HP09	Kayla Prentice	Retrospective Review of Clinical Presentation, Survival and Response to Therapy in Dogs with Granulomatous Hepatitis
**SMALL ANIMAL INTERNAL MEDICINE ‐ INFECTIOUS DISEASE**		
ID12	Jamie Sebastian	Evaluation of Leptospira Exposure in Feral Cat Populations in Northern California and Southern Texas
**SMALL ANIMAL INTERNAL MEDICINE ‐ NEPHROLOGY/UROLOGY**		
NU01	Aleksandra Milaszewska	Evaluation of Creatinine, Symmetric Dimethylarginine, Kidney Injury molecule‐1 and Glomerular Filtration Rate in Healthy Cats
NU26	Carolina Rivera	Urinary Clusterin and Cystatin B Concentrations in Dogs with Kidney Disease
NU28	Sarah Spencer	Mineralocorticoid Receptor Expression and Activation in Feline Chronic Kidney Disease and Associations with Disease Progression
**SMALL ANIMAL INTERNAL MEDICINE ‐ OTHER**		
OT01	Kellyn McNulty	Using End‐of‐Life Survey to Investigate the Relationship between Quality‐of‐Life, Manner and Location of Death
**SMALL ANIMAL INTERNAL MEDICINE ‐ PHARMACOLOGY**		
P06	Heta Turunen	Reversal of the Effects of a Medetomidine‐vatinoxan Combination Drug (Zenalpha) with Atipamezole
P07	Adele Williams	Clinical Audit of POM‐V/POM Prescriptions by Remote Consultation via a Veterinary Video Telemedicine Smartphone Application
**SMALL ANIMAL INTERNAL MEDICINE ‐ RESPIRATORY**		
R07	Yuta Nakazawa	Study of Nasal Microbiome in Dogs with Nasal Diseases and Healthy Dogs
**EQUINE**		
E05	Hannah Kinsella	The Enteroinsular Axis in Hospitalized Foals with Gastrointestinal Disease
E15	Muriel Sacks	How Sedation and Recumbency Influence Distribution of Ventilation Measured by Electrical Impedance Tomography in Foals
E25	Naomi Kirkwood	Prospective Assessment of Clinical Signs and ACTH Concentrations in Horses Transitioning to PPID
E31	Nicolas Galinelli	Effects of Dopamine Suppression on Postprandial Glucose and Insulin Responses to Glucose Feeding in Horses
E39	Simon Libak Haugaard	Ultrasonographic Assessment of Small Intestinal Motility Following Hyoscine Butylbromide Administration in Horses
E62	David Byrne	Electrical Impedance Tomography Can Determine Airflow in the Respiratory System of Healthy Adult Horses
E64	Kimberly Hallowell	An Updated Description of Bacterial Pneumonia in Adult Horses and Factors Associated with Non‐survival
**FOOD ANIMAL INTERNAL MEDICINE**		
F11	Mireille Meylan	Associations Between Measured Climate Parameters, Barn Characteristics, and Health Indicators in Swiss Veal Calf Herds
F12	Jennifer Davis	Detection Times of Florfenicol/florfenicol Amine in Lactating Meat and Dairy Goats, and Milk Fed Kids

## 1 CARDIOLOGY

## 2 Abstract C02

### 2.1 Retrospective Evaluation of Liver Parameters in Canine Degenerative Valvular Disease and Dilated Cardiomyopathy

#### 2.1.1 **Andrew Chong** ^1^; Meaghan Appleton^1^; Domingo Casamián‐Sorrosal^2^, DVM, DECVIM‐CA (Internal Medicine and Cardiology); Shari Raheb^1^, DVM, DVSc, DACVIM (Cardiology); Lynne O'Sullivan^3^, DVM, DVSc, DACVIM (Cardiology); Ananda Pires^1^, DVM; Sonja Fonfara^1^, Dr med vet, PhD, DECVIM (Cardiology)

##### 2.1.1.1 ^1^Ontario Veterinary College; ^2^Hospital Veterinario UCV y Departamento de Medicina, Universidad Católica de Valencia San Vicente Mártir; ^3^Atlantic Veterinary College

**Background:** There is a lack of information regarding the association between canine acquired cardiac disease and the hepatic system.

**Objective:** Evaluate the relationship between hepatic parameters, survival, and disease stages of dogs with either dilated cardiomyopathy (DCM) or degenerative valvular disease (DVD).

**Animals:** Ninety‐nine client‐owned dogs consisting of 16 healthy controls and dogs diagnosed with DVD (N=61) and DCM (N=22) in either American College of Veterinary Internal Medicine stage B or C of disease.

**Methods:** Retrospective study evaluating the association between liver parameters, type and stage of disease, and survival. Univariate and multivariate Cox proportional hazards models were used to determine parameters associated with survival.

**Results:** Differences in hepatic parameter concentrations between DCM and DVD dogs and disease stages were identified. Alanine aminotransferase (P<0.001), aspartate aminotransferase (P=0.02), and total bilirubin (P=0.005) were significant predictors of mortality in univariate analysis. In the multivariate analysis, total bilirubin was the only hepatic parameter that was an independent predictor of mortality (P=0.029).

**Conclusions:** Differences in hepatic parameters between DCM, DVD, and disease stages are likely due to disease specific hemodynamics and disease progression. These findings suggest that in dogs with DVD and DCM, the interaction between the cardiac and hepatic systems may be relevant for disease progression and outcome, as is reported for humans with cardiac disease. Further studies into the role of hepatic function in canine cardiac disease are required.

## 3 Abstract C03

### 3.1 Assessing Myocardial Remodeling in Dogs with Myxomatous Mitral Valve Disease Using Cardiac Magnetic Resonance Imaging

#### 3.1.1 **William A. Clark** ^1^; Randolph Winter^2^, DVM, PhD, DACVIM (Cardiology); Turi Aarnes^1^; Eric Green^1^; Patrick Ruz^1^; Daniel Addison^1^; Jaylyn Rhinehart^1^; Karsten Schober^1^; Harry Friel^3^

##### 3.1.1.1 ^1^The Ohio State University; ^2^Auburn University; ^3^Philips Healthcare

**Background:** Some dogs with myxomatous mitral valve disease (MMVD) develop progressive volume overload and cardiomegaly. Histopathologic studies, but not antemortem evaluations, have demonstrated myocardial fibrosis in some of these dogs. Cardiac magnetic resonance imaging (CMR) aids in antemortem diagnosis of myocardial fibrosis in humans.

**Hypothesis/Objectives:** The objectives were to evaluate dogs with MMVD and healthy dogs for myocardial ischemia and fibrosis using serum biomarkers and CMR. We hypothesized that myocardial changes would be evident on both modalities in affected dogs.

**Animals:** Six dogs with MMVD stage B2 and six healthy dogs recruited from a hospital population.

**Methods:** Prospective case‐control study. Dogs underwent echocardiography and serum cardiac biomarker measurement (cardiac troponin I (cTnI), galectin‐3 (Gal‐3)). Dogs were anesthetized for CMR, where T2 weighted images and pre‐ and post‐gadolinium contrast T1 weighted images were acquired and analyzed on dedicated software. Data were analyzed for normality and expressed as mean±SD or median(interquartile range). Student's t‐tests or Wilcoxon rank sums tests were performed.

**Results:** No significant differences were observed for pre‐contrast T1 values, post‐contrast T1 values, and T2 values (msec) (MMVD 1171.33±52.77, 728(700–764.75), and 39.2±7.6; controls 1180.83±33.89, 711(697.5–736.5), and 42.38±12.62; p=0.72, p=0.57, p=0.61). Serum cTnI (ng/mL) was greater in MMVD dogs than controls (0.067±0.019 vs. 0.033±0.021; p=0.013). Serum Gal‐3 (pg/mL) was not different between groups (MMVD 178.07±105.59, controls 305.24±123.29; p=0.08).

**Conclusions and Clinical Importance:** Myocardial fibrosis detected by CMR and Gal‐3 were not more common in MMVD dogs than controls. Dogs with MMVD stage B2 may not have clinically relevant myocardial fibrosis.

## 4 Abstract C04

### 4.1 Comparison of Vascular Closure Techniques in Dogs Undergoing Percutaneous Transcatheter Intervention

#### 4.1.1 **Arianne F. Fabella** ^1^; Lauren Markovic^2^, DVM, DACVIM (Cardiology); Amanda Coleman^3^, DVM, DACVIM (Cardiology)

##### 4.1.1.1 ^1^University of Georgia; ^2^Assistant Professor, Small Animal Medicine & Surgery, University of Georgia; ^3^Associate Professor, Small Animal Medicine & Surgery, University of Georgia

**Background:** Manual compression has been the standard‐of‐care for maintaining hemostasis after percutaneous endovascular intervention, but can be time‐consuming and associated with vascular complications. Alternative closure methods include temporary figure‐of‐eight suture (Z‐stitch) and vascular closure device (VCD) techniques.

**Hypothesis:** We hypothesized that compared to manual compression, Z‐stitch and VCD would significantly reduce time‐to‐hemostasis, and the proportion of dogs with vascular patency among treatments would not differ significantly.

**Animals:** Twenty‐one client owned dogs undergoing percutaneous transvenous interventional procedures.

**Methods:** Dogs with vessel diameter <5 mm were randomized to undergo manual compression (n=6) or Z‐stitch (n=7). Dogs with vessel diameter ≥5 mm were randomized to undergo manual compression (n=2), Z‐stitch (n=2), or VCD (n=4). Time to hemostasis, Bleeding Academic Research Consortium (BARC) score, and presence of vascular patency at 24 hours were recorded. Data are presented as median (95% CI).

**Results:** Median time to hemostasis was significantly shorter in Z‐stitch (2.9 [1.6–4.0] min) versus manual compression (10.0 [10.0–20.0] min; p<0.001) or VCD (8.5 [8.1–11.8] min); p<0.003) groups, and in VCD versus manual compression (p=0.018) groups. At 5 minutes, BARC scores were significantly higher (consistent with worse bleeding), in the manual compression, versus Z‐stitch (p=0.005), but not VCD (p=0.16) groups. There was no difference in the proportion of dogs with vascular patency at 24 hours among methods (7/8, 4/4 and 9/9 in manual compression, VCD, and Z‐stitch groups, respectively; p=0.57).

**Conclusions:** Z‐stitch and VCD are effective hemostasis methods, with Z‐stitch providing the most rapid hemostasis.

## 5 Abstract C05

### 5.1 Anti‐desmosomal Antibody as a Potential Biomarker of Arrhythmia Burden in Boxers with Arrhythmogenic Ventricular Cardiomyopathy

#### 5.1.1 **Luis Dos Santos** ^1^; Suzanne Cunningham^2^, DACVIM (Cardiology); Robert Hamilton^3^, MD; Meena Fatah^4^, BSc

##### 5.1.1.1 ^1^Purdue University; ^2^Associate Professor, Clinical Sciences, Cummings School of Veterinary Medicine, Tufts University; ^3^Professor, Hospital for Sick Children (SickKids); ^4^Department of Paediatrics (Cardiology), The Hospital for Sick Children

**Background:** Arrhythmogenic right ventricular cardiomyopathy (ARVC) is a common entity in Boxer dogs causing syncope or sudden death. As Holter and echocardiographic abnormalities frequently do not develop until after 5–6 years of age, novel biomarkers for identification of affected individuals are needed. Circulating autoantibodies to cardiac desmosomal protein desmoglein‐2 (anti‐DSG2) have been identified in the serum of people and Boxers with ARVC.

**Hypothesis/Objectives:** Anti‐DSG2 antibodies will discriminate between affected and healthy Boxer dogs and correlate with disease severity as measured by 24‐h burden of ventricular premature contractions (VPCs) in affected dogs.

**Animals:** Client‐owned Boxer dogs ≥5 years of age were recruited.

**Methods:** Prospective, cross‐sectional study. The normal group was comprised of Boxers with <100 VPCs on 24‐h Holter and normal echocardiogram. The affected group was comprised of Boxers with >300 VPCs on Holter or documented ventricular tachycardia. Cardiac biomarkers (high sensitivity troponin‐I and NT‐proBNP) were evaluated, and Western blots and direct ELISA were carried out using recombinant DSG2 protein.

**Results:** Fifty‐one dogs were enrolled (healthy = 24; affected = 27). Cardiac troponin‐I (Mann‐Whitney, p<0.001) and NT‐proBNP (Mann‐Whitney, p<0.001) were significantly higher in affected dogs, but anti‐DSG2 antibody did not differ between groups (p=0.63). A positive correlation between VPCs over 24 h and anti‐DSG2 was observed (Pearson, p=0.012, r=0.4, Figure 1).

**Figure:**
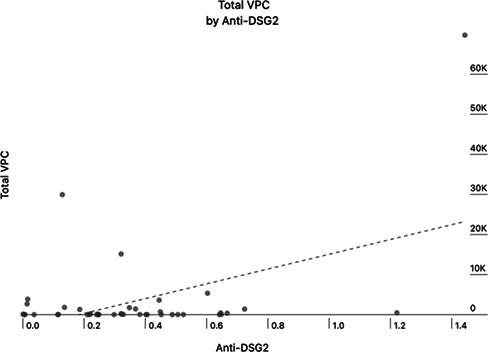
Figure 1

**Conclusion:** Anti‐DSG2 analysis did not discriminate between normal and affected dogs but may have utility for monitoring arrhythmia burden and possibly progression of ARVC.

## 6 Abstract C06

### 6.1 Three‐Dimensional Echocardiographic Evaluation of Right Ventricular Function in Boxers with Arrhythmogenic Right Ventricular Cardiomyopathy

#### 6.1.1 **Luis Dos Santos** ^1^; Suzanne Cunningham^2^, DVM, DACVIM; Elizabeth Rozanski^2^, DVM, DACVIM, DACVECC

##### 6.1.1.1 ^1^Purdue University; ^2^Associate Professor, Department of Clinical Sciences, Tufts University

**Background:** 2D‐echocardiography evaluation of the RV is limited, as a single‐plane view cannot illustrate the complex morphology of the right ventricle. Since limited criteria are available for arrhythmogenic right ventricular cardiomyopathy (ARVC) in Boxers, further diagnostic investigation is needed.

**Objectives:** To explore three‐dimensional right ventricular (3DRV) relationships between normal and ARVC Boxers.

**Animals:** Forty‐nine privately‐owned, aged‐matched (≥5yo) healthy (n=22) and ARVC afflicted Boxer dogs (n=27).

**Methods:** Prospective cross‐sectional echocardiography study. 3DRV data such as end‐diastolic (EDVRV) and end‐systolic (ESVRV) volumes, fractional area change (FAC), and ejection fraction (EF) were acquired. M‐mode tricuspid annulus plane systolic excursion (TAPSE), pulsed wave velocity of the lateral tricuspid annulus (S′), and body‐weight indices were also assessed.

**Results:** Age and body weight were similar in both groups. 3DRV analysis was consistently reproducible and data were normally distributed in the healthy group. TAPSE did not correlate to 3DRV functional parameters, such as EF (r=0.13, p=0.3) or FAC (r=0.05; p=0.68). Interestingly, S′ had positive correlation to functional parameters EF (r=0.4, p=0.005) and FAC (r=0.43, p=0.003). No correlation was found between markers of RV function, volume and body weight in this population of Boxer dogs.

**Conclusions and Clinical Importance:** 3DRV echocardiography might provide reproducible reference values for healthy Boxers and improve assessment of function and volume in afflicted ARVC Boxers.

## 7 Abstract C07

### 7.1 Biologic Variability of Galectin‐3 and NT‐proBNP in Healthy Cats and Cats with Occult Hypertrophic Cardiomyopathy

#### 7.1.1 **Leah Kruckman** ^1^; Ryan Fries^2^, DVM, DACVIM (Cardiology); Saki Kadotani^3^, DVM, DACVIM (Cardiology); Jon Stack^4^, DVM, DACVIM (Cardiology); Emily Javery^5^, DVM; Lindsey Humphries^6^, DVM; Sumana Prabhakar^6^, DVM; Gabrielle Wallace^7^, DVM, DACVIM (Cardiology)

##### 7.1.1.1 ^1^University of Illinois at Urbana‐Champaign; ^2^Assistant Professor, Veterinary Clinical Medicine, University of Illinois at Urbana‐Champaign; ^3^Clinical Assistant Professor, Veterinary Clinical Medicine, University of Illinois at Urbana‐Champaign; ^4^VCA Loomis Basin Veterinary Clinic; ^5^Cardiology Intern, Veterinary Clinical Medicine, University of Illinois at Urbana‐Champaign; ^6^Cardiology Resident, University of Illinois at Urbana‐Champaign; ^7^Pacific Northwest Pet ER and Specialty Center

**Background:** The biologic variability (BV) of a biomarker, or change in analyte concentration independent of disease progression, is required for serial monitoring. In cats, this information has not been determined for the cardiac biomarkers N‐terminal pro‐brain natriuretic peptide (NT‐proBNP) and Galectin‐3 (Gal‐3).

**Hypothesis/Objectives:** To determine the BV of Gal‐3 and NT‐proBNP in healthy and hypertrophic cardiomyopathy (HCM) cats.

**Animals:** Twelve healthy and 13 occult HCM cats.

**Methods:** NT‐proBNP and Gal‐3 were measured using a commercially available assays (IDEXX and RayBiotech). All cats had an examination, echocardiogram, blood pressure, and T4 analysis. Cats were classified as healthy or HCM based on septal or free wall thickness >6 mm and further staged as B1 (n=8) and B2 (n=5) based on left atrial diameter >16 mm. Cardiac biomarkers were evaluated within‐day, daily, and weekly over 6 weeks.

**Results:** Within‐subject (CV_I_) and between‐subject (CV_G_) coefficients of variation were calculated. For healthy cats: CV_I_ of 15.9% for Gal‐3 and 28.1% for NT‐proBNP; CV_G_ of 33.9% for Gal‐3 and 34.9% for NT‐proBNP. In healthy cats, a required % change of 47.9% for Gal‐3 and 78.9% for NT‐proBNP is required to exceed BV.

For HCM: CV_I_ of 27.9% for Gal‐3 and 29.2% for NT‐proBNP; CV_G_ of 67.1% for Gal‐3 and 79.7% for NT‐proBNP. In all HCM cats, a required % change of 83.9% for Gal‐3 and 83.5% for NT‐proBNP is required to exceed BV.

**Conclusions and Clinical Importance:** The BV of NT‐proBNP and Gal‐3 in cats should be considered when serially monitoring these cardiac biomarkers.

## 8 Abstract C08

### 8.1 The Effect of Spironolactone on the Renin‐Angiotensin‐Aldosterone System in Dogs

#### 8.1.1 **Allison Masters** ^1^; Jonathan Mochel^1^; Emilie Guillot^2^; Agnes Bourgois‐Mochel^1^; Chelsea Iennarella‐Servantz^1^; Lingnan Yuan^3^; Jessica Ward^1^

##### 8.1.1.1 ^1^College of Veterinary Medicine, Iowa State University; ^2^Ceva Sante Animale; ^3^Iowa State University

**Background:** Spironolactone has been associated with reduced risk of cardiac morbidity and mortality in humans and dogs with congestive heart failure. There is little evidence on the effect of spironolactone on biomarkers of the classical and alternative arms of the renin‐angiotensin‐aldosterone system (RAAS) in dogs.

**Hypothesis/Objectives:** To characterize the effect of spironolactone on the RAAS in healthy dogs.

**Animals:** Ten healthy purpose‐bred Beagles.

**Methods:** Study dogs were randomly allocated to two spironolactone dosing groups (2 mg/kg or 4 mg/kg PO q 24 hr for 7 days). Angiotensin peptides and aldosterone serum levels before and after treatment were quantified by LC‐MS/MS. Wilcoxon sum rank tests were performed to compare study variables between treatments and baseline. P‐values <0.1 were considered statistically significant.

**Results:** Following spironolactone treatment, dogs showed significant increases in angiotensin I (AngI median 136; IQR 77–167), AngII (median 70; IQR 39–81), and Ang(1‐5) (median 52; IQR 41–62) compared to baseline (AngI median 63; IQR 38–112; p=0.08; AngII median 32; IQR 23–52; p=0.09; Ang(1‐5) median 24; IQR 17–40; p=0.08). Changes from baseline in angiotensin peptides did not differ between study groups for the dosing range investigated.

**Conclusions and Clinical Importance:** Spironolactone treatment increases the activity of the classical and alternative RAAS pathways in healthy dogs. A greater pharmacological response was not observed for the higher dosing group, suggesting that a 2 mg/kg q 24 hr oral dose of spironolactone already reaches a plateau activity on RAAS.

## 9 Abstract C09

### 9.1 Evaluation of Cardiac Biomarkers and Point‐of‐Care Ultrasound in Dogs with Non‐hemorrhagic Ascites

#### 9.1.1 **Alice G. Morey** ^1^; James Karnia^1^; Kenneth Lamb^2^; Stacey Leach^1^, DACVIM (Cardiology); Bridget Lyons^1^, DACVECC; Laura Nafe^1^, DACVIM (SAIM); Kelly Wiggen^1^, DACVIM (Cardiology)

##### 9.1.1.1 ^1^University of Missouri; ^2^Lamb Consulting LLC

**Background:** Non‐hemorrhagic ascites (NHA) can be caused by cardiac and non‐cardiac disease. N‐terminal brain natriuretic peptide (NT‐proBNP), cardiac troponin‐I (cTnI) and point‐of‐care ultrasound (POCUS) may aid in differentiating between cardiac and non‐cardiac causes of NHA.

**Hypothesis/Objectives:** This study compared NT‐proBNP and cTnI concentrations in dogs presenting for cardiac and non‐cardiac causes of NHA. This study also compared POCUS findings, specifically hepatic venous and caudal vena cava (CaVC) distension, and gall bladder wall edema.

**Animals:** Client‐owned dogs (n=60) were enrolled based on identification of NHA with an effusion packed cell volume <10%.

**Methods:** Blood samples and POCUS were collected from dogs. Dogs were diagnosed with cardiac (n=28) or non‐cardiac (n=32) NHA based on echocardiography. The cardiac group was subdivided into pericardial disease (n=11) and non‐pericardial cardiac disease (n=17).

**Results:** A significant difference in NT‐proBNP concentration (mean±SD pmol/L) was noted between cardiac (4869±4078) and non‐cardiac (1882±2627) groups (p=0.022), with a sensitivity of 53.8% and specificity of 85.7% at a cut‐off of 4092 pmol/L. The NT‐proBNP concentrations were also significantly different between pericardial (1179±1398) and non‐pericardial cardiac (7177±3435) groups (p<0.0001). There were no significant differences in cTnI concentrations between groups. Significant differences in POCUS findings between the cardiac and non‐cardiac groups included hepatic venous distension (18/28 vs. 3/29, p<0.0001) and CaVC distension (13/27 vs. 2/29, p<0.001), but not gall bladder wall edema (4/28 vs. 3/29, p=0.7373).

**Conclusions and Clinical Importance:** NT‐proBNP and POCUS help distinguish between cardiac and non‐cardiac causes of NHA.

## 10 Abstract C10

### 10.1 Comparison of Single‐Dose Pharmacokinetics in Dogs Receiving Vetmedin® and Two Formulations of Generic Pimobendan

#### 10.1.1 **Sara M. Ostenkamp** ^1^; Andrew Woodward^2^, PhD, DACVCP; Ted Whittem^3^, BVSc, PhD, DACVCP, FANZCVS; Daniel Hogan^4^, DVM, DACVIM (Cardiology)

##### 10.1.1.1 ^1^Purdue University; ^2^University of Canberra; ^3^Professor, Veterinary Clinical Sciences, University of Melbourne; ^4^Professor, Veterinary Clinical Sciences, Purdue University

**Background:** Vetmedin® is commonly prescribed for the treatment of cardiac disease in dogs. Compounded formulations of generic pimobendan are commercially available and may offer more accurate dosing at a more affordable cost.

**Hypothesis/Objectives:** The aim of this study was to evaluate the pharmacokinetic biosimilarity of pimobendan and its active metabolite, O‐deoxymethyl pimobendan (ODMP) following a single oral dose of Vetmedin® (V), pimobendan capsule (C), and pimobendan suspension (S) in healthy dogs.

**Animals:** Six healthy, purpose‐bred research beagle dogs.

**Methods:** This was a randomized cross‐over study. Each dog received one formulation of pimobendan at a mean dose of 0.33 mg/kg (range: 0.3–0.35) and blood was collected for pharmacokinetic analysis; this was repeated for the other two formulations following a 48‐hour washout period after administration of each formulation.

**Results:** Noncompartmental modeling was used. The pimobendan geometric mean Cmax was 51.9 ng/mL (V), 51.2 ng/mL (S), and 36.1 ng/mL (C) while the ODMP Cmax was 45.3 ng/ml (V), 44.5 ng/ml (S), and 33.2 ng/ml (C). AUC were compared between formulations using a hierarchical mixed model. The ratio of AUC between V and S fell within the *a priori* acceptable range for bioequivalence (1.01; 0.88–1.16 CrI 90%), while the AUC ratio between V and C was 1.17 (0.93–1.44 CrI 90%).

**Conclusions and Clinical Importance:** Pimobendan exposure was similar between the generic suspension and Vetmedin®, but lower with the generic capsule. The results support that generic suspension and Vetmedin® are clinically exchangeable. Generic pimobendan capsule may be clinically satisfactory based on available varying dosing strategies or may require dose adjustment.

## 11 Abstract C11

### 11.1 Evaluating the Safety and Efficacy of Clevidipine in Dogs with Congestive Heart Failure

#### 11.1.1 **Ashley N. Sharpe** ^1^; Ronald Li^2^, DVM, DACVECC, PhD; Jamie Burkitt‐Creedon^3^, DVM, DACVECC; Catherine Gunther‐Harrington^4^, DVM, DACVIM (Cardiology); Joshua Stern^5^, DVM, PhD, DACVIM (Cardiology)

##### 11.1.1.1 ^1^University of California, Davis; ^2^Assistant Professor, Department of Surgical and Radiological Sciences, University of California, Davis; ^3^Department of Surgical and Radiological Sciences, University of California, Davis; ^4^Department of Medicine and Epidemiology, University of California, Davis; ^5^Chief Veterinary Medical Officer, Department of Medicine and Epidemiology, University of California, Davis

**Background:** Congestive heart failure (CHF) secondary to canine myxomatous mitral valve disease (MMVD) is common, and adjunct therapies for acute decompensation are lacking. Afterload reduction enhances cardiac output, but vasodilator options are limited. Clevidipine is a novel intravenous dihydropyridine calcium channel blocker that may aid in canine CHF management.

**Hypothesis/Objectives:** Clevidipine, compared to standard of care (SOC), will result in a 20% reduction in mean arterial pressure (MAP) in a dose‐dependent manner without adverse effects when used for afterload reduction in CHF secondary to MMVD. A secondary objective was to determine whether oscillometric blood pressure measurement (OSC) provides a reliable assessment of MAP in treated patients.

**Animals:** Ten client‐owned dogs in acute decompensated CHF secondary to MMVD.

**Methods:** Prospective, randomized, open‐label, clinical trial. Dogs enrolled received SOC alone or with clevidipine (CLEV) with continuous direct arterial (ART) and OSC monitoring throughout treatment.

**Results:** Target MAP was achieved in all CLEV patients and MAP was significantly decreased compared to SOC (p=0.0005) at doses 4.5–16.5 mcg/kg/min (5.25, IQR 4.50–9.75 mcg/kg/min). Comparison of ART and OSC methods demonstrated overestimation of MAP in both SOC and CLEV groups (bias −13.99, −15.45). There was no significant difference in BUN or creatinine following treatment in either group, and no notable hypotension was appreciated.

**Conclusions and Clinical Importance:** Clevidipine was well‐tolerated and resulted in a dose‐dependent reduction of MAP in dogs with MMVD and CHF. Direct arterial blood pressure monitoring is recommended for monitoring response to clevidipine therapy.

## 12 Abstract C12

### 12.1 Genotyping, Echocardiographic Screening, and Whisker Papilla Fibroblast Collection in Purebred Maine Coon and Ragdoll Cats

#### 12.1.1 **Lilian L. Shen** ^1^; Barbara Berrios^1^; Amara Estrada^1^; Oscar Maldonado^1^; Christina Pacak^2^

##### 12.1.1.1 ^1^University of Florida; ^2^University of Minnesota

**Background:** Hypertrophic Cardiomyopathy (HCM) is the most common inherited cardiomyopathy in cats, affecting approximately 10–15% of domestic cats. Two separate, breed‐specific genetic mutations have been linked to the development of HCM in Maine Coons and Ragdolls. Previous work has demonstrated the utility of fibroblasts as easily accessible *in vitro* models for studying inheritance, pathophysiology, and possible novel therapies for cardiomyopathies such as HCM.

**Hypothesis/Objective:** To establish a novel, non‐invasive sampling technique to create a bank of fibroblast cells for use as a future HCM drug screening platform.

**Animals:** Maine Coon and Ragdolls with or without history of cardiomyopathy were enrolled from breeding clubs and the University of Florida.

**Methods:** Cats received physical and echocardiographic examinations. Buccal mucosal swabs were collected for genotyping for the A31P and R820W mutations through North Carolina State University. Vibrissae, including follicle, were collected and fibroblasts cultured.

**Results:** 21 Maine Coons and 12 Ragdolls were enrolled. All cats were found to be wildtype for the mutations of interest. All Ragdolls were normal on echocardiogram. Two Maine Coons were diagnosed with cardiomyopathy. Following protocol optimization, whisker papilla fibroblast cultures were successfully isolated from 3/3 Maine Coons and 3/3 Ragdolls.

**Conclusions and Clinical Importance:** Vibrissae serve as a non‐invasive source of fibroblasts from cats. The identification of HCM phenotype in two cats despite wildtype genotype reinforces that development of HCM is multifactorial. Banking of feline fibroblasts has been initiated, and further expansion will facilitate *in vitro* studies of the physiology of HCM and enable testing of therapies.

## 13 Abstract C13

### 13.1 Potential Predictors of Advanced Heart Failure Secondary to Myxomatous Mitral Valve Disease in Dogs

#### 13.1.1 **I Ping Chan** ^1^; Chuan Chuan Li^2^; Chao Chin Chang^3^; Tung Hsueh^3^; Chung Chun Yang^3^; Shiun Long Lin^3^

##### 13.1.1.1 ^1^Veterinary Teaching Hospital, National Chung Hsing University; ^2^Walk Animal Hospital; ^3^National Chung Hsing University

**Background:** Predictive variables and survival time of advanced heart failure (AHF) secondary to myxomatous mitral valve disease (MMVD) in dogs remain unknown.

**Hypothesis/Objectives:** This retrospective case‐control study investigated predictive variables of AHF in dogs with MMVD.

**Animals:** The study included 38 dogs diagnosed with AHF and 38 with stable congestive heart failure (CHF) secondary to MMVD. AHF group was determined as dogs with refractory CHF treated with a total daily dosage of 8 mg/kg furosemide or the equivalent dose of torsemide. Dogs were included in the stable group, if there were no changes in the maintenance dose of furosemide (lower than 8 mg/kg/day) and other cardiac medicines above 12 weeks.

**Methods:** Logistic regression modeling was used to compare and analyze clinical, radiographic, and echocardiographic variables between groups. Survival times were analyzed using the Kaplan‐Meier method.

**Results:** Left atrium to aortic root ratio, normalized left ventricular dimension at the end‐diastole and end‐systole, isovolumic relaxation time (IVRT) and the ratio of early transmitral inflow velocity to IVRT were associated with AHF progression. The median survival times in the stable group were significantly longer than those in the AHF group. After the diagnosis of AHF, the median survival times of all‐cause mortality and cardiogenic mortality were 194 days (range 0–1,088 days) and 354 days (range 0–1,063 days), respectively.

**Conclusions and Clinical Importance:** AHF was significantly associated with enlarged left atrium, left ventricle and elevated left atrial pressure in dogs with MMVD; these dogs exhibited a survival time of approximately six months.

## 14 Abstract C14

### 14.1 Development of a Deep Learning Model for Analysis of Narrow‐Complex Heart Rhythms in Dogs

#### 14.1.1 **Matthew D. Denton** ^1^; Roger Denton^2^; Brian Scansen^3^, DVM, MS, DACVIM (Cardiology)

##### 14.1.1.1 ^1^Colorado State University; ^2^Unaffiliated; ^3^Associate Professor, Clinical Sciences, Colorado State University

**Background:** Deep learning (DL) models have been validated for human electrocardiogram (ECG) analysis, but there is minimal experience with DL models in veterinary ECG interpretation.

**Objective:** This study aimed to develop a DL model for the interpretation of narrow‐complex ECGs in dogs and to determine model accuracy against the rhythm diagnosis reported in the medical record.

**Animals:** ECG recordings from 346 dogs were selected from the ECG database of the cardiology service of the Colorado State University Veterinary Teaching Hospital, acquired between October 2020 and November 2021.

**Methods:** The DL model was programmed to classify canine ECGs into sinus rhythm, second‐degree atrioventricular block, third‐degree atrioventricular block, supraventricular ectopic complexes, and atrial fibrillation/flutter. Wide complex QRS morphologies were excluded due to similarities between ventricular origin and aberrant conduction of supraventricular rhythms. ECGs with multiple concurrent arrhythmias or excessive artifact were excluded. The DL model was trained utilizing ECG metadata, Fourier transformed data, and 6‐lead waveforms. Balanced data perturbation was employed. The model was trained using 271 recordings and tested with 75 additional recordings.

**Results:** The DL model accuracy was 93% for sinus, 80% for third‐degree atrioventricular block, 80% for supraventricular complexes, 66% for second‐degree atrioventricular block, and 25% for atrial fibrillation/flutter. Negative predictive values for these rhythms varied from 85% to 98%. Model accuracy was 81% using ECG metadata, 72% by Fourier transform, and 37% using hexaxial lead inputs.

**Conclusions and Clinical Importance:** DL models are a potential future diagnostic aid in canine ECG interpretation with further model refinement ongoing.

## 15 Abstract C15

### 15.1 Evaluation of Platelet Activation, Platelet‐Leukocyte Interaction, and Hemostasis in Dogs with Pulmonary Hypertension

#### 15.1.1 **Laetitia Duler**; Lance Visser; Nghi Nguyen; Lynelle Johnson; Joshua Stern; Ronald Li

##### 15.1.1.1 Veterinary Teaching Hospital, University of California, Davis

**Background:** The role of platelets (PLT) and their immune function in pulmonary hypertension (PH) remain poorly understood. Evidence in humans suggests vascular changes are secondary to PLT activation and thrombosis.

**Hypothesis/Objectives:** To assess PLT activation, PLT‐leukocyte interactions and thromboelastography (TEG) in dogs with PH.

**Animals:** 29 client‐owned dogs. Group 1 = 5 dogs with PH secondary to myxomatous mitral valve disease (MMVD), and 10 control MMVD dogs without PH. Group 2 = 5 dogs with PH due to respiratory diseases and 4 dogs with PH due to other causes, and 5 control dogs with respiratory diseases only.

**Methods:** Prospective observational case‐control study. PH diagnosis was based on ACVIM guidelines. Dogs underwent echocardiogram, thoracic radiographs, heartworm antigen test, TEG, and flow cytometry for PLT P‐selectin expression and PLT‐leukocyte interaction. Platelet activation and PLT‐leukocyte aggregates were measured as P‐selectin median fluorescence intensity (MFI) and percent‐positive events.

**Results:** Dogs with MMVD and PH had augmented PLT response to ADP (7153±4673 vs. 3110±1328, p=0.021) and thrombin (14,126±4280 vs. 5374±2648, p=0.0003) compared to control MMVD dogs. PH dogs in Group 2 also had augmented thrombin‐mediated activation compared to controls (20588±31693 vs. 4864±1364, p=0.02). Platelet‐leukocyte interaction was higher following ADP‐induced activation in MMVD dogs with PH (25.01%±15.24 vs. 12.92%±4.18, p=0.04). No significant differences were found in any TEG parameters between dogs with and without PH.

**Conclusion and Clinical Importance:** Platelets are hyper‐reactive in dogs with PH, thereby increasing platelet‐leukocyte aggregate formation. Platelet‐leukocyte interaction likely plays a role in the pathogenesis of PH in dogs.

## 16 Abstract C16

### 16.1 Diagnostic Validation of a Vertebral Heart Score Machine Learning Algorithm for Canine Lateral Thoracic Radiographs

#### 16.1.1 **Jessica Gentile Solomon**; Scott Bender; Pavan Durgempudi; Caitlin Robar; Joe Healy; Michael Cocchiaro; Allison Spake; Sean Turner; Christopher Watson; Donald Szlosek

##### 16.1.1.1 IDEXX Laboratories, Inc.

**Background:** The vertebral heart score (VHS) is a measurement used to index the heart size to the thoracic vertebra and is a useful tool for identifying the presence of heart disease, staging heart disease, and providing prognostic information. There is increasing development of computer‐aided algorithms to perform specific tasks, which can reduce radiograph reading times and help minimize human error.

**Hypothesis/Objectives:** The purpose of this study was to validate the use of a VHS algorithm compared to manual VHS scoring by three board‐certified veterinary cardiologists.

**Animals:** A total of 1,200 canine lateral radiographs were randomly selected from a commercial telemedicine databank.

**Methods:** A convolutional neural network centered around semantic segmentation of relevant anatomical features was developed to predict heart size and vertebral bodies. These predictions were used to calculate the VHS score. Three board‐certified cardiologists manually scored 400 images each using the traditional Buchanan method. Post‐scoring, the cardiologists evaluated the algorithm for appropriate algorithm landmarks and overall image quality.

**Results:** The 95th percentile absolute error between the cardiologist VHS score and the algorithm VHS score was 1.05 vertebrae (95% CI: 0.97–1.20 vertebrae) with a mean error of −0.09 vertebrae (95% CI: −0.12–−0.05 vertebrae) and an absolute mean error of 0.3 vertebrae. The model was well‐calibrated across the predictive range.

**Conclusions and Clinical Importance:** We found the performance of the VHS algorithm comparable to that of three board‐certified cardiologists.

## 17 Abstract C17

### 17.1 Feasibility and Safety of High‐resolution Three‐Dimensional Electroanatomical Mapping of the Complete Heart in Dogs

#### 17.1.1 **Arnaut Hellemans** ^1^; Glenn Van Steenkiste^2^, DVM, PhD; Tim Boussy^3^, MD, PhD; Mattias Duytschaever^4^, MD, PhD; Gunther Van Loon^2^, DVM, DECEIM, PhD; Tim Bosmans^5^, PhD, DVM; Emmelie Stock^6^, DVM, DECVDI, PhD; Marcin Skotarek^7^, DVM; Gitte Mampaey^7^, DVM; Margot Gheeraert^7^, DVM; Pascale Smets^7^, DVM, DECVIM, PhD

##### 17.1.1.1 ^1^Ghent University; ^2^Department of Internal Medicine, Reproduction and Population Medicine, Ghent University; ^3^Department of Cardiology, AZ Groeninge, Belgium; ^4^Universitair Ziekenhuis Gent (Ghent University Hospital), Ghent University; ^5^Department of Anesthesiology, Ghent University; ^6^Department of Medical Imaging, Ghent University; ^7^Small Animal Department, Ghent University

**Background:** Three‐dimensional electroanatomical mapping (3D EAM) has expanded radiofrequency catheter ablation (RFCA) applications in humans to almost all complex arrhythmias and has drastically reduced fluoroscopy use, yet its potential in dogs is poorly investigated.

**Objectives:** To assess the feasibility and safety of 3D EAM of all four heart chambers in dogs using minimal fluoroscopy.

**Animals:** Eight Beagles (median weight 12.2 kg (11.2–15.7)).

**Methods:** Prospective experimental trial. Three‐dimensional EAM was attempted during sinus rhythm under general anesthesia using a 22‐electrode mapping catheter and CARTO 3 system. Left heart catheterization was achieved by retrograde transaortic access (n=4) or transseptal puncture (TSP) (n=4). Primary outcomes included feasibility, safety and fluoroscopy time.

**Results:** Successful 3D EAM of the right atrium and ventricle was achieved in all dogs. Left atrial and ventricular 3D EAM was achieved in six and seven dogs respectively. Median fluoroscopy time was 6.8 min (0.0–45.1) and almost exclusively associated with TSP. Complications requiring intervention occurred in one dog only and were a transient third‐degree atrioventricular block after interatrial septum engagement, which reversed after intracardiac pacing (10.3 min), and development of pericardial effusion following TSP treated by pericardiocentesis. Peri‐operative self‐limiting arrhythmias were frequently observed. Only two dogs received lidocaine to resolve intra‐operative ventricular tachycardia.

**Conclusions and Clinical Importance:** Three‐dimensional EAM of all cardiac chambers is feasible, requires minimal fluoroscopic assistance and is promising to guide RFCA of arrhythmias in dogs. Complications were mainly related to TSP and similar to those reported in people.

## 18 Abstract C18

### 18.1 Prospective Validation of a Quantitative Point‐of‐Care NT‐proBNP Assay for Screening Doberman Pinschers

#### 18.1.1 **Emily Javery** ^1^; Ryan Fries^2^, DVM, DACVIM (Cardiology); Lindsey Humphries^3^, DVM, MS; Saki Kadotani^4^, DVM, DACVIM (Cardiology); Sumana Prabhakar^3^, DVM; Gabrielle Wallace^5^, DVM, MS, DACVIM (Cardiology)

##### 18.1.1.1 ^1^University of Illinois at Urbana‐Champaign; ^2^Assistant Professor, Veterinary Clinical Medicine, University of Illinois at Urbana‐Champaign; ^3^Cardiology Resident, Veterinary Clinical Medicine, University of Illinois at Urbana‐Champaign; ^4^Clinical Assistant Professor, Veterinary Clinical Medicine, University of Illinois at Urbana‐Champaign; ^5^Cardiologist, Pacific Northwest Pet ER and Specialty Center

**Background:** N‐terminal pro‐brain natriuretic peptide (NT‐proBNP) is useful in identifying Doberman Pinschers with echocardiographic evidence of dilated cardiomyopathy (DCM). An independently validated, quantitative point‐of‐care (POC) NT‐proBNP assay (Vcheck, BioNote USA Inc) is now commercially available and uses fresh serum, eliminating the need for specialized sample processing and shipping.

**Hypothesis/Objectives:** The purpose of this study was to prospectively screen a cohort of adult Dobermans for DCM using the Vcheck NT‐proBNP assay, echocardiography, and 3‐minute electrocardiography.

**Animals:** One hundred and twenty‐seven adult Doberman Pinschers were screened.

**Methods:** Tests performed included physical examination, transthoracic echocardiography with simultaneous 3‐lead electrocardiography, and quantitative NT‐proBNP levels using the Vcheck assay (detection range of 500–10,000 pmol/L).

**Results:** Population median age was 5 years (2–12 years), and there were 74 females and 53 males. Diagnoses included normal (n=103), DCM (n=13), Equivocal/VPC only (n=5), and MMVD (n=6). Median (range) NT‐proBNP for normal = 499 pmol/L (499–1585) and DCM = 900 pmol/L (511–2604) P=0.001. A cutoff of >509 pmol/L = 100% sensitivity and 55% specificity, the highest Youden Index was for >615 pmol/L = 85% sensitivity and 81% specificity. To achieve a 95% specificity a cutoff of >892 pmol/L was needed; however, this yielded a sensitivity of 46%.

**Conclusions and Clinical Importance:** This study indicates that quantitative NT‐proBNP using a novel POC analyzer is useful as a screening test to detect DCM in Doberman Pinschers and compares favorably with previous studies.

## 19 Abstract C19

### 19.1 Occurrence of Low Taurine Concentrations in a Swedish Population of Cocker Spaniels

#### 19.1.1 **Karin Kriström** ^1^; Jens Häggström^2^, DVM, PhD, DECVIM; Andrea Fascetti^3^, DVM, PhD, DACVIM; Mark Dirven^4^, DVM, DECVIM; Joshua Yu^5^, PhD; Anna Tidholm^4,6^, DVM, PhD, DECVIM; Ingrid Ljungvall^6^, DVM, PhD, DECVIM

##### 19.1.1.1 ^1^Swedish University of Agricultural Sciences; ^2^Professor, Clinical Sciences, Swedish University of Agricultural Sciences; ^3^Professor, University of California, Davis; ^4^AniCura Albano Small Animal Hospital; ^5^University of California, Davis, ^6^Associate Professor, Clinical Sciences, Swedish University of Agricultural Sciences

**Background:** Taurine deficiency‐induced dilated cardiomyopathy (DCM) has been reported in Cocker Spaniels (CS).

**Objectives:** To investigate the occurrence of low blood taurine concentrations in a Swedish population of CS presenting as clinically healthy or with a DCM phenotype and congestive heart failure (CHF), and to assess associations between taurine concentrations and dog characteristics and clinical features.

**Animals:** One‐hundred seventy privately owned CS (168 English (ECS) and two American (ACS)).

**Methods:** Prospectively recruited dogs underwent physical and echocardiographic examination, and blood analyses (hematology and biochemistry). Taurine concentrations were analyzed in plasma (EDTA and heparin), and whole blood (WB). Dogs were classified as having low taurine concentrations if results were below reported reference range in either type of plasma and/or WB.

**Results:** A total of 46 dogs (27%) had low taurine concentrations. Dogs with low taurine concentrations were older compared to dogs with normal concentrations (P=0.03). Other dog characteristics and clinical features did not differ between the groups. Eight of 46 dogs (17%) with low taurine concentrations, of which seven were ECS one ACS, presented with a DCM‐phenotype and CHF. Heparin was the additive that identified the highest number of dogs with low taurine concentrations.

**Conclusions and Clinical Importance:** Low taurine concentrations were common in the population of CS investigated and were found in both clinically healthy dogs as well as in all dogs with CHF. Besides age, no difference in dog characteristics and clinical features were found between dogs with normal and low taurine concentrations.

## 20 Abstract C20

### 20.1 An International Survey of Preferences for Echocardiographic Assessment of Left Atrial Size in Cats

#### 20.1.1 **Momo Y. Kuo** ^1^; Jens Häggström^2^, DVM, PhD, DECVIM‐CA (Cardiology); Sonya Gordon^3^, DVM, DVSc, DACVIM (Cardiology); Katja Höglund^4^, DVM, PhD; Etienne Côté^5^, DVM, DACVIM (Cardiology, SAIM); Mark Dirven^6^, DVM, DECVIM‐CA (Cardiology); Mark Rishniw^7^, BVSc, MS, PhD, DACVIM (Cardiology, SAIM); Ta‐Li Lu^8^, DVM, DAiCVIM (Cardiology); Yong‐Wei Hung^9^, DVM, MS, DAiCVIM (Cardiology); Ingrid Ljungvall^10^, DVM, PhD, DECVIM‐CA (Cardiology)

##### 20.1.1.1 ^1^Swedish University of Agricultural Sciences; ^2^Professor, Clinical Sciences, Swedish University of Agricultural Sciences; Professor, ^3^Texas A&M University; ^4^Associate Professor, Anatomy, Physiology and Biochemistry, Swedish University of Agricultural Sciences; ^5^Professor, Companion Animals, University of Prince Edward Island; ^6^AniCura Albano Animal Hospital; ^7^College of Veterinary Medicine, Cornell University; ^8^Chuan Animal Hospital; ^9^Cardiospecial Veterinary Hospital; ^10^Associate Professor, Department of Clinical Sciences, Swedish University of Agricultural Sciences

**Background:** Veterinary echocardiographers’ preferences for left atrial (LA) size assessment in cats have never been systematically investigated.

**Hypothesis/Objective:** Investigate echocardiographers’ preferences concerning LA size assessment in cats including positioning of the cat, acquisition views, indexing methods and timing; and whether geographic/professional profiles could be associated with echocardiographers’ preferences for assessing LA size in cats.

**Animals:** None.

**Methods:** An online survey was distributed globally to veterinary echocardiographers.

**Results:** A total of 655 veterinary echocardiographers from 6 continents and 54 countries, working in specialty practice (56%) and in general practice (38%), completed the survey. Linear 2D‐based methods were favored by most echocardiographers (n=612). Most commonly, echocardiographers combined linear 2D‐based methods with subjective assessment (n=227), while 209 used linear 2D‐based methods alone. Most echocardiographers (n=464) using linear 2D‐based methods preferred the right parasternal short‐axis view and to index the LA to the aorta. Less than 8% (n=43) used linear 2D obtained images from a right parasternal long‐axis 4‐chamber view without indexing the LA. Approximately one third (n=210/612, 34.3%) assessed LA size using linear 2D echocardiography shared the same preferences regarding cat position, acquisition view, indexing method and time‐point identification for the measurement. The responses were comparably homogeneous across geographic location, level of training, years performing echocardiography, and type of practice.

**Conclusion and Clinical Importance:** Most veterinary echocardiographers assessed LA size in cats using linear 2D echocardiography from a right parasternal short axis view, indexing the LA to the aorta. The echocardiographers’ demographic and professional profiles had a minor influence on the responses.

## 21 Abstract C21

### 21.1 Evaluation of Cardiac and Endothelial Dysfunction in Canine Cancer Patients Treated with Toceranib Phosphate

#### 21.1.1 **Katherine E. Lopez** ^1^; Dawn Meola^2^; Lori Lyn Price^3^; Jenica Upshaw^3^, MD; Iris Jaffe^3^, MD, PhD; Cheryl London^1^, DVM, PhD, DACVIM (Oncology); Vicky Yang^1^, DVM, PhD, DACVIM (Cardiology)

##### 21.1.1.1 ^1^Cummings School of Veterinary Medicine, Tufts University; ^2^Tufts University; ^3^Tufts Medical Center

**Background:** Toceranib phosphate is a tyrosine kinase inhibitor (TKI) and a non‐selective inhibitor of Vascular Endothelial Growth Factor (VEGF). While hypertension has been documented as a side effect in canine patients receiving TKIs, to date, there have not been studies evaluating left ventricular systolic function and endothelial function in canine patients.

**Objectives:** To characterize changes in echocardiographic parameters and biomarkers of endothelial function in canine patients treated with toceranib.

**Animals:** 12 client‐owned dogs diagnosed with neoplasia (mast cell tumors N=5, anal sac adenocarcinoma N=4, thyroid carcinoma, mesothelioma, and multiple N=1) receiving single agent toceranib for cancer treatment.

**Methods:** This is a prospective study approved by the Tufts University IACUC. Patients had echocardiographic exams and blood pressure measurements. Blood and urine samples were obtained to evaluate levels of VEGF, endothelin‐1, platelet derived growth factor (PDGF), prostacyclin, nitric oxide (nitrite and nitrate) metabolites, and cGMP using commercially available ELISA kits. Examination time‐points include baseline, one‐, three‐, and five‐ months after start of treatment. Statistical analysis was performed using repeated measures mixed models.

**Results:** Statistically significant decreases in urine nitrate and plasma prostacyclin concentrations were detected over time. Although not reaching statistical significance, VEGF and systolic blood pressure increased. Four of the study dogs required treatment with hypertension medication (BP >170 mm Hg). No significant changes in ejection fraction or fractional shortening were observed.

**Conclusions:** Biomarkers of endothelial dysfunction associated with hypertension, including prostacyclin and nitrate, decreased during treatment with toceranib. Larger sample size will be required to confirm the findings of this study.

## 22 Abstract C22

### 22.1 Assessment of Cardiotoxicity After Combretastatin A4‐Phosphate Administration in Dogs Using Two‐Dimensional Speckle Tracking Echocardiography

#### 22.1.1 **Gitte Mampaey** ^1^; Hilde De Rooster^2^, DVM, DECVS, PhD; Eline Abma^3^, DVM, PhD; Arnaut Hellemans^3^, DVM; Tom Schipper^4^, DVM; Bart J.G. Broeckx^5^, DVM, PhD; Pascale Smets^2^, DVM, DECVIM, PhD

##### 22.1.1.1 ^1^Ghent University; ^2^Professor, Small Animal Department, Ghent University; ^3^Small Animal Department, Ghent University; ^4^Department of Veterinary and Biosciences, Ghent University; ^5^Professor, Department of Veterinary and Biosciences, Ghent University

**Background:** Combretastatin A4‐phosphate (CA4P) is a vascular disrupting agent recently described for treatment of solid tumors in dogs. Conventional echocardiography did not show signs of cardiotoxicity. However, the gold standard for assessing myocardial damage in humans receiving cardiotoxic chemotherapeutics is two‐dimensional speckle tracking echocardiography (2D‐STE).

**Hypothesis/Objectives:** The aim of this study was to evaluate the cardiotoxic effect of CA4P in dogs, based on 2D‐STE measurements.

**Animals:** Echocardiographic images of healthy dogs and cancer patients that received CA4P were retrospectively reviewed. Seven healthy beagles and five cancer patients were included.

**Methods:** Peak global longitudinal strain (GLS), peak global circumferential strain (GCS) and peak global radial strain (GRS) were measured before and 24 hours after administration of CA4P. Strain measurements were compared with cardiac troponin I (cTnI) measurements. On seven echocardiographic examinations, each strain parameter was measured by 3 observers on 3 consecutive days to obtain intra‐observer and inter‐observer variability.

**Results:** Median GCS and GLS values decreased by 17.6% (*P*=0.002) and 24.0% (*P*=0.0005), respectively. Median GRS values were not significantly different (*P*=0.16). The decrease in GLS was correlated with a rise in cTnI (Spearman rho = −0.63, *P*=0.03). The intra‐observer coefficient of variation was 0.04, 0.13 and 0.09 for GCS, GRS and GLS, respectively, while the corresponding inter‐observer coefficients were 0.12, 0.20 and 0.11.

**Conclusions and Clinical Importance:** Administration of CA4P has a cardiotoxic effect, which can be detected by 2D‐STE strain measurements and correlates with cTnI concentrations.

## 23 Abstract C23

### 23.1 Agreement Between Doppler Ultrasound and High‐Definition Oscillometric Device for Systolic Arterial Pressure in Hospitalized Dogs

#### 23.1.1 **Matheus Matioli Mantovani** ^1^; Juliana Aires^2^; Eutálio Pimenta^2^; Any Costa^1^; Suzana Tsuruta^1^; Jacqueline Castro^1^; Luis Dos Santos^3^

##### 23.1.1.1 ^1^Federal University of Uberlandia; ^2^Federal University of Minas Gerais; ^3^Purdue University

**Background:** The agreement between systolic arterial pressure (SAP) measured by Doppler ultrasonic (DU) and by high‐definition oscillometric device (HDO) in hypotensive, normotensive, and hypertensive dogs has not yet been evaluated.

**Objectives:** To assess limits of agreement (LoA) of SAP measured by DU and HDO normotensive (90≤SAP≤160 mm Hg), hypotensive (SAP <90 mm Hg) and hypertensive (SAP >160 mm Hg) dogs.

**Animals:** Sixty privately‐owned, non‐anesthetized dogs were studied: normotensive (n=34), hypotensive (n=10) and hypertensive (n=16).

**Methods:** Prospective clinical study. Same cuff sizes were used for both methods, and readings were not recorded simultaneously. Five consecutive SAP readings were obtained from each dog with DU and HDO. All measurements were analyzed according to the criteria outlined by the ACVIM consensus statement.

**Results:** A moderate correlation was shown in normotensive (r=0,53; p<0.001) and hypertensive (r=0.52; p<0.038) groups, while a strong correlation was documented in the hypotensive (r=0,81; p<0.004) group. The SAP bias for normotension and hypotension groups was 1.00±13.55 mm Hg (LoA: −25.62 to 27.52) and −3.50±7.61 mm Hg (LoA: −18.42 to 11.43 mm Hg), respectively. No significant differences between SAP bias by DU and HDO in normotensive (P=0.93) and hypotensive (P=0.452) were appreciated.

**Conclusion:** Good agreement between methods was shown in normotensive and hypotensive groups for SAP. Since values of MAP and DAP measured by HDO are more accurate than SAP, and DU cannot provide these values, further studies evaluating agreement with invasive pressure are needed.

## 24 Abstract C24

### 24.1 Multi‐Center Prospective Evaluation of Owner Medication Adherence for Feline Cardiovascular Disease in the Referral Setting

#### 24.1.1 **Lisa A. Murphy** ^1^; Justin Allen^2^; Emily Chapel^3^; Reid Nakamura^4^; Brian O'Malley^5^; Donald Schrope^5^; Caroline Sloan^6^; Maria Wang^2^; Sarah Zimmerman^3^

##### 24.1.1.1 ^1^Friendship Hospital for Animals; ^2^VCA West LA; ^3^ACCESS; ^4^Idexx; ^5^Oracle Animal Hospital; ^6^VCA Veterinary Specialists of the Valley

**Background:** A previous study evaluating medication adherence in dogs with cardiovascular disease reported medication adherence was high. Medication adherence for cats with cardiovascular disease remains unexamined.

**Hypothesis/Objectives:** To examine chronic medication adherence for the management of feline cardiovascular disease in the referral setting.

**Methods:** Over a three‐month period, a questionnaire was distributed to owners at each investigator's hospital which employed a board‐certified veterinary cardiologist or a cardiology resident supervised by a board‐certified veterinary cardiologist. For inclusion into the study, owners had to have been medicating their cat with at least one cardiac medication requiring daily administration for at least one month. The cat did not need to have been diagnosed with congestive heart failure to participate in the study.

**Results:** Fifty‐four questionnaires were available for review. The most common diagnosis was hypertrophic cardiomyopathy (HCM) (31/54, 57.4%). Clopidogrel was reported as the medication most difficult to consistently administer (13/54, 24.0%) although 20 owners (20/54, 37.0%) reported no difficulties chronically administering cardiac medications. Thirty‐six (66.7%) owners reported the highest frequency of medications they could administer was twice‐a‐day dosing. Medication adherence in this study population was high with 50 owners (92.6%) meeting the criteria for medication adherence.

**Conclusions and Clinical Importance:** Medication adherence was reportedly high in this study. Veterinary cardiologists should be aware of owner concerns when determining treatment protocols for management of feline cardiovascular disease.

## 25 NEUROLOGY

## 26 Abstract N01

### 26.1 Age, Breed, and SOD1:c.118G Genotype Related Risk of Degenerative Myelopathy: An Update

#### 26.1.1 **Natalie A. Villani** ^1^; Julianna Sabol^2^, VMD; Joan Coates^3^, DVM, MS, DACVIM (Neurology); Gary Johnson^4^, DVM, PhD

##### 26.1.1.1 ^1^University of Missouri; ^2^North Carolina State University; ^3^Professor and Section Head, Neurology and Neurosurgery, University of Missouri; ^4^Professor, Veterinary Pathobiology, University of Missouri

**Background:** Canine degenerative myelopathy (DM) is a debilitating condition. The DM‐associated SOD1:c.118G>A genetic variant is the most common disease‐associated variant in dogs. Risk of DM associated with this variant is a controversial topic.

**Hypothesis/Objectives:** Further characterize age‐ and breed‐related risk for development of DM associated with the SOD1:c.118G>A variant.

**Animals:** Dogs with DNA voluntarily submitted for SOD1:c.118G>A variant screening were identified for follow‐up, and 2,143 dogs were included following owner response.

**Methods:** Dogs were selected from a population screened for the SOD1:c.118G>A variant prior to age 6, who were 7 or older at follow‐up. A survey was emailed to assess myelopathic signs. Responses were reviewed independent of breed, sex, or genotype, to assign dogs as having DM compatible signs or not. Kaplan‐Meier survival analysis and Cox proportional hazards regression were evaluated.

**Results:** Cumulative event curves revealed age‐related risk of disease that varies by genotype (Figure 1) and breed. Rhodesian Ridgebacks, Chesapeake Bay Retrievers, Boxers, German Shepherd Dogs, and Pembroke Welsh Corgis had sufficient numbers for analysis, and 83 breeds were represented by at least one dog. DM hazard ratio was increased over wild‐type in homozygous variant and heterozygous dogs.

**Figure:**
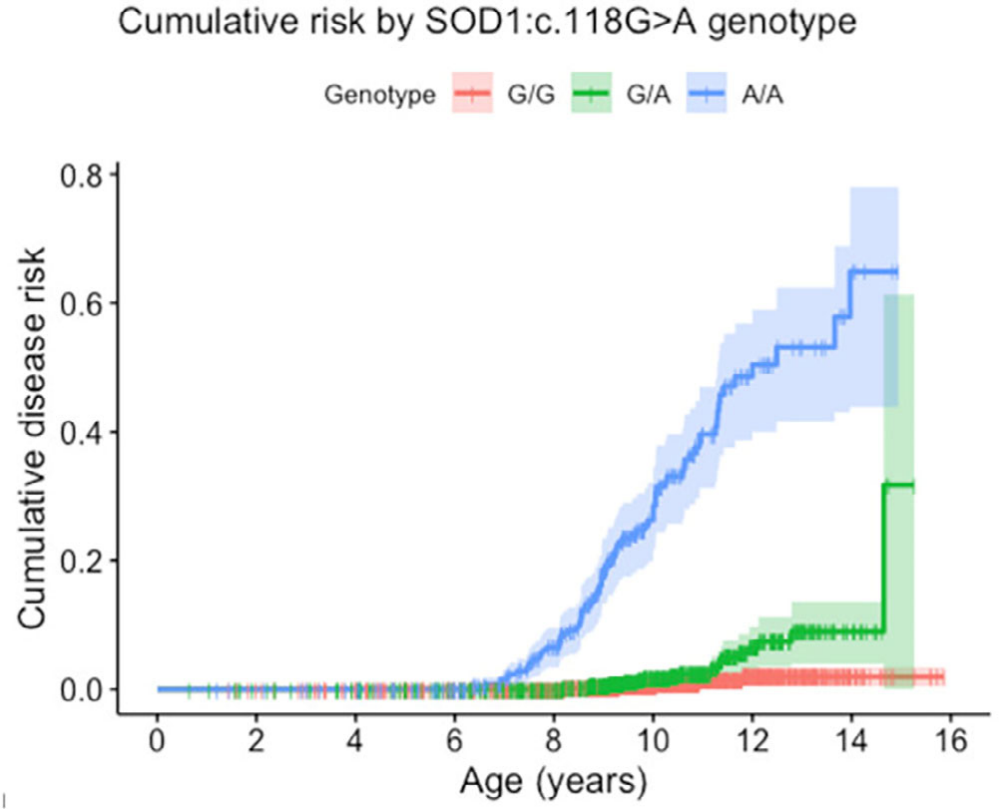
Figure 1

Cumulative event curve showing cumulative risk divided by *SOD1:c.118G>A* genotype. Animals are censored at death or ascertainment. *N: A/A = 292, G/A = 759, G/G = 1092*. Log‐rank p value = < 0.0001

**Conclusions and Clinical Importance:** This study illustrates effect of SOD1:c.118G>A genotype on development of DM‐compatible signs in several breeds, including increased risk in heterozygous dogs over wild‐type. Agreement between clinical signs and genotype underscores use of genetic testing as an adjunctive diagnostic tool and for screening of breeding animals.

## 27 Abstract N02

### 27.1 Electrical Impedance Myography in Healthy Dogs: Reference Values and Impact of Age

#### 27.1.1 **Joseph B. Kowal** ^1^; Sarah Verga^2^; Sarbesh Pandeya^2^; Randall Cochran^3^, DVM, DACVIM (Neurology); Julianna Sabol^3^, DVM; Stefanie Lim^3^, DVM; Joan Coates^3^, DVM, DACVIM (Neurology); Seward Rutkove^2^, MD

##### 27.1.1.1 ^1^University of Missouri; ^2^Beth Israel Deaconess Medical Center, Harvard Medical School; ^3^Veterinary Health Center, University of Missouri

**Background:** Electrical impedance myography (EIM) is a tool for muscle assessment based on voltages obtained with application of high‐frequency, low‐intensity electrical current. The phase value metric is calculated based on resistance and reactance values.

**Hypothesis/Objectives:** To assess normal EIM values in healthy dogs and determine an age dependence to the metric.

**Animals:** 73 healthy dogs (62 males, 49 females) were sampled for a total of 111 muscle observations. Mean age was 5.1 years (0.63–13.48 years), and mean body condition score was 5 (4 to 6).

**Methods:** EIM measurements were performed on bilateral cranio tibialis, gastrocnemius, and sartorius muscles and assessed at 100 kHz phase. A second‐degree polynomial regression was used to fit for phase with age and extract the lower limit of prediction for sex.

**Results:** Lower limits of normal were established across a range of ages. Data suggested modest reduction in phase values with age, consistent with expected decline in muscle condition. The average value decreased from 16.6° in 2‐year‐old male dogs to 12.7° in 13‐year‐old male dogs (23.4% reduction) and decreased from 15.4° in 2‐year‐old female dogs to 12.1° in 13‐year‐old female dogs (21.4% reduction).

**Conclusions and Clinical Importance:** EIM is a non‐invasive, quantitative measure of muscle pathology utilized to track muscle condition in healthy dogs. There is a small but consistent reduction in EIM values with increased age. The normal EIM values established here can serve as reference for future studies in disease.

## 28 Abstract N03

### 28.1 Electrical Impedance Myography in Dogs with Degenerative Myelopathy

#### 28.1.1 **Joseph B. Kowal** ^1^; Sarah Verga^2^; Sarbesh Pandeya^2^; Randall Cochran^3^, DVM; Julianna Sabol^3^, DVM; Seward Rutkove^2^, MD; Joan Coates^3^, DVM, DACVIM (Neurology)

##### 28.1.1.1 ^1^University of Missouri; ^2^Beth Israel Deaconess Medical Center, Harvard Medical School; ^3^College of Veterinary Medicine, University of Missouri

**Background:** Canine degenerative myelopathy (DM) causes disuse and neurogenic muscle atrophy. Electrical impedance myography (EIM) has been used to quantify muscle pathology in neuromuscular disorders.

**Hypothesis/Objectives:** To compare EIM between DM‐affected and similar aged healthy dogs, and assess EIM changes over time in DM‐affected dogs.

**Animals:** Eleven DM‐affected (histopathologically confirmed) and fifteen similar aged neurologically normal dogs.

**Methods:** A prospective observational study evaluated multifrequency EIM performed on DM‐affected dogs at baseline and during disease progression. Muscles evaluated included bilateral cranio tibialis, gastrocnemius, gracilis, sartorius, and biceps femoris. The 100 kHz phase angle (θ) was extracted from the full frequency set for analysis.

**Results:** As compared to normal dogs, DM‐affected dogs had lower phase values of the gastrocnemius on the left (θ=7.69, 13.06; p=0.002) and right (θ=6.11, 11.72; p=0.001) and lower mean phase values of combined muscles on the left (θ=9.24, 11.62; p=0.012) and right (θ=9.18, 11.72; p=0.021). Other individual muscles measured did not show significant differences, although values were consistently lower in DM‐affected dogs. A significant (p<0.05) decline in phase of the gastrocnemius was seen with disease progression over time in serially monitored DM‐affected dogs.

**Conclusions and Clinical Importance:** EIM can detect pathologic changes in muscles of DM‐affected dogs and phase progressively declines with disease progression. As a non‐invasive and quantitative measure of muscle pathology, EIM may be useful in future studies for tracking disease progression and response to therapy.

## 29 Abstract N04

### 29.1 Comparison of Surgical Outcomes Associated with Hemorrhagic Extradural Longitudinal Parenchymal Compression in the Canine Patient

#### 29.1.1 **Patti E. Lawler**; Jonathan Wood; Nicole Alleva; Mark Rishniw; Ian Porter; Philippa Johnson

##### 29.1.1.1 Cornell University

**Background:** A subset of intervertebral disk extrusion (IVDE) cases has compressive, hemorrhagic inflammation within the epidural space in addition to compression from the herniated disk material. We term this hemorrhagic extradural longitudinal parenchymal (HELP) compression. Previous reports have been conflicting in the outcomes of these cases.

**Hypothesis/Objectives:** The goals are to compare success of surgical decompression in dogs with HELP compression compared to Modified Frankel Score (MFS) matched control dogs with non‐hemorrhagic disk extrusions, evaluate the extent of spinal cord compression on MRI compared to final patient outcomes in HELP compression, and determine the compression to decompression ratio and its relation to outcomes in HELP cases.

**Animals:** 143 dogs from the neurology service were included: HELP compression dogs (n=65) and IVDE dogs (n=78).

**Methods:** This is a retrospective case‐control cross‐sectional study. Each case was assigned an MFS on admission and follow‐up. MRIs were reviewed and compression to decompression ratios were calculated based on T2 sagittal MRI images. Outcomes were assigned for each patient based on MFS at follow‐up.

**Results:** Outcomes of HELP and IVDE did not differ when taken to surgery when stratified by MFS. Dogs with HELP compression had more compressed sites than IVDE (P=0.001) and had more sites decompressed (P<0.001). Compression to decompression ratio did not differ between groups (P=0.52) and their outcomes were not significantly different.

**Conclusions and clinical importance:** When addressed surgically, dogs with HELP compression have similar outcomes to dogs with non‐hemorrhagic IVDE.

## 30 Abstract N05

### 30.1 Survival in Dogs with Meningoencephalomyelitis of Unknown Etiology with and without Magnetic Resonance Imaging Lesions

#### 30.1.1 **Arielle Ostrager** ^1^; R. Timothy Bentley^2^, BVSc (Dist), MRCVS, DACVIM‐Neurology; Melissa Lewis^3^, VMD, PhD, DACVIM‐Neurology; George Moore^4^, DVM, PhD

##### 30.1.1.1 ^1^Purdue University; ^2^Associate Professor, Neurology and Neurosurgery, VCS Courtesy Associate Professor, BMS, Veterinary Clinical Sciences, Purdue University; ^3^Assistant Professor, Neurology, Veterinary Clinical Sciences, Purdue University; ^4^Director, Clinical Trials and Professor of Epidemiology, Department of Veterinary Administration, Purdue University

**Background:** The prognosis of dogs with meningoencephalomyelitis of unknown etiology (MUE) is difficult to predict. MUE cases with normal magnetic resonance imaging (MRI) occur, but it is unknown whether this finding alters prognosis.

**Hypothesis:** Normal‐MRI MUE dogs survive longer than MUE dogs with MRI lesions.

**Animals:** 82 client‐owned dogs with MUE presenting to a university hospital from 2010–2020.

**Methods:** Retrospective study. Dogs with a clinical diagnosis of MUE were included if they were between 6 months to 10 years at diagnosis, had brain or spine MRI performed, had a CSF white blood cell count >5 cells/μL, were tested for infectious diseases, and had follow‐up for ≥3 months or until death. MRIs were reviewed for the presence or absence of MUE lesions. Deaths due to MUE were compared between dogs with and without MRI lesions using a Kaplan‐Meier curve. A log‐rank test was performed to evaluate statistical significance (p<0.05).

**Results:** Sixty dogs (73%) had MRI lesions with a median survival of >70.7 months. Twenty‐two dogs (27%) did not have MRI lesions with a median survival of >98.0 months. The death rate was 23% for dogs with MRI lesions and 9% for dogs without (p=0.152).

**Conclusions:** The majority of MUE dogs had prolonged survival. No statistically significant difference in death rate of dogs with and without MRI lesions was found. Additional outcome measures (e.g., remission rates) with extended follow‐up should be studied to determine whether the presence of MRI lesions influences prognosis in MUE.

## 31 Abstract N06

### 31.1 Comparison of D‐Dimer and Thromboelastography for Diagnosis of Cerebrovascular Accidents in Dogs

#### 31.1.1 **Elizabeth DiPaola** ^1^; Starr Cameron^2^, MS, BVetMed, DACVIM (Neurology); Scott Hetzel^3^, MS

##### 31.1.1.1 ^1^University of Wisconsin‐Madison; ^2^Clinical Assistant Professor in Small Animal Neurology, Department of Medical Sciences, University of Wisconsin‐Madison; ^3^Department of Biostatistics and Medical Informatics, University of Wisconsin‐Madison

**Background:** Cerebrovascular accidents (CVAs) in dogs are documented with magnetic resonance imaging (MRI). This imaging modality is not always available. Additionally, some CVAs may be too small to be detected or appear similar to other pathology on MRI. Thromboelastography (TEG) and D‐Dimer are utilized in human medicine for detection of CVAs but their use in veterinary patients has not been assessed.

**Hypothesis/Objectives:** To assess utility of TEG and D‐Dimer testing in diagnosing CVAs in dogs.

**Animals:** 76 client‐owned dogs with acute onset neurologic signs having a brain MRI and TEG or D‐Dimer testing performed.

**Methods:** Multicenter, retrospective study. The incidence of abnormal TEG or D‐Dimer results was compared between patients with clinical or MRI evidence of CVA and a control population (neurologic diagnosis other than CVA). Analysis methods included Fisher's or Chi‐square test for association.

**Results:** In our population, neither TEG or D‐Dimer was significantly associated with a CVA (*p*=0.374 and 0.425, respectively). The sensitivity of TEG for CVA diagnosis was significantly higher than D‐Dimer in all cases (60.6% and 25.0%, respectively; *p=*0.042) and for cases with MRI evidence alone (TEG 66.7%, D‐Dimer 23.1%, *p=*0.025). The specificity of abnormal TEG or D‐Dimer for CVA diagnosis was not significantly different, even in cases with MRI evidence alone (*p*=0.606, *p*=0.440). When comparing hemorrhagic and ischemic CVAs, an abnormal TEG or D‐Dimer were not significantly associated with diagnosis (*p*=0.168 and 0.832, respectively).

**Conclusions and Clinical Importance:** Abnormal TEG or D‐Dimer alone is not a reliable indicator of CVA in dogs.

## 32 Abstract N07

### 32.1 ‘50‐Step Walking Test’—A Pragmatic Outcome Measure for Recovery After Canine Spinal Cord Injury

#### 32.1.1 **Suzanne Rosen** ^1^; Nicholas Jeffery^2^, BVSc, FRCVS, DECVN, PhD, DSAS DECVS; Jessica Grzegorzewski^1^, AAS, BS, LVT

##### 32.1.1.1 ^1^Medical Teaching Hospital, Texas A&M Veterinary; ^2^Professor, Department of Small Animal Clinical Sciences, Veterinary Medical Teaching Hospital, Texas A&M University

**Background:** Surprisingly, there is currently no agreed definition of ‘ambulation’ following recovery after spinal cord injury (SCI) in dogs.

**Hypothesis/Objectives:** To create and validate a new definition of ambulation in dogs recovering from SCI.

**Animals:** 206 client‐owned dogs weighing <20 kg: 110 ambulatory dogs were used to determine relationship between limb (ulna) length and distance covered by 50 step cycles; 96 non‐ambulatory dogs that underwent surgical decompression for acute thoracolumbar disc herniation.

**Methods:** Prospective descriptive study. Ambulatory dogs were leash‐walked and the distance covered by 50 step cycles was measured. Ulna length was measured in each dog and a regression equation relating ulna length to 50‐step cycle distance was generated. The non‐ambulatory dogs underwent decompressive surgery and were followed until they recovered to walk the distance covered by 50 step cycles. Recovery of ability to walk and the time taken to achieve this distance were compared amongst dogs that were paraparetic, paraplegic and paraplegic with absent pain sensation.

**Results:** 11/25 dogs without pain sensation at presentation recovered the ability to walk the 50‐step distance at 4 months, compared with 69/71 dogs with intact pain sensation. Median time to recovery was 87 days in dogs presenting without pain sensation and 14 days in both paretic and plegic dogs.

**Conclusions and Clinical Importance:** The 50‐step walking test provides a standard outcome that is easily applied by owners, can reliably detect differences in outcome following different severities of thoracolumbar SCI and could be used in future clinical trials of new therapies.

## 33 Abstract N08

### 33.1 Current Practices in the Diagnosis and Management of Dogs with Degenerative Myelopathy: A Questionnaire‐Survey

#### 33.1.1 **Teryn Bouche** ^1^; Joan Coates^2^, DVM, MS, DACVIM; Sarah Moore^3^, DVM, DACVIM; Dominik Faissler^4^, DVM, DECVN; Mark Rishniw^5^, BVSc, MS, PhD, DACVIM; Natasha Olby^1^, MB, PhD, MRCVS, DACVIM

##### 33.1.1.1 ^1^Veterinary Hospital, North Carolina State University; ^2^University of Missouri; ^3^The Ohio State University; ^4^Cummings School of Veterinary Medicine at Tufts University; ^5^VIN, Cornell University

**Background:** Ante‐mortem diagnosis of degenerative myelopathy (DM) is presumptive and there are no accepted guidelines regarding management of this neurodegenerative condition.

**Hypothesis/Objectives:** To describe current practices of neurologists and rehabilitation specialists in diagnosis and management of DM.

**Animals:** None.

**Methods:** Online questionnaires regarding diagnosis and management of DM in dogs were constructed and emailed to neurology and rehabilitation listservs.

**Results:** 190 neurologists and 79 rehabilitation specialists from 20 countries completed the questionnaires. Most (142/190) neurologists and rehabilitation specialists (23/39) require SOD‐1 genetic testing for presumptive diagnosis and 82/190 neurologists also require spinal magnetic resonance imaging (MRI). Most neurologists recommend regular exercise (187/190) and physical therapy (184/190). Over 50% (102/190) of neurologists perform rechecks on dogs they diagnose with DM, while the remainder do not. Rehabilitation specialists report preservation or improvement of strength (78/79) and coordination (77/79) as therapeutic goals. They recommend at‐home exercises (75/79) as well as underwater treadmill (64/79), gait training (55/79) and strength building exercises (65/79) and report that treatment helps maintain strength (58/79), coordination (56/79), and muscle mass (56/79) with improvement in overall wellbeing (54/79). Neurologists reported that owners elect euthanasia when dogs become non‐ambulatory paraparetic while rehabilitation specialists report euthanasia when paraplegia and incontinence develop.

**Conclusion and Clinical Importance:** Most clinicians use SOD‐1 genetic testing to presumptively diagnose DM. While many neurologists reevaluate dogs diagnosed with DM, continued care is often referred to rehabilitation specialists or primary veterinarians. Treatment recommendations include exercise and rehabilitation. Many rehabilitation specialists report clinical stabilization and improved wellbeing with treatment.

## 34 Abstract N09

### 34.1 A Novel Technique for Measuring Spasticity in Dogs After Acute Thoracolumbar Intervertebral Disc Extrusion (TL‐IVDE)

#### 34.1.1 **Lauren M. McAllister**; Lane Bookenberger, DVM; Ashley Hechler, DVM, MS, DACVIM (Neurology); Ronaldo Da Costa, DMV, MSc, PhD, DACVIM (Neurology); Sarah Moore, DVM, DACVIM (Neurology)

##### 34.1.1.1 College of Veterinary Medicine, The Ohio State University

**Background:** Spasticity following spinal cord injury (SCI) occurs commonly, but ways to measure this in dogs are underexplored. Dynamometry is commonly used to quantify spasticity in people.

**Objectives:** Evaluate a handheld dynamometer for quantifying spasticity in healthy dogs and SCI‐affected dogs.

**Animals:** Prospective cohort study in 11 healthy dogs and 10 with acute TL‐IVDE (SCI‐affected).

**Methods:** A handheld dynamometer (The Commander™ console and muscle tester, JTECH Medical) was used to perform measurements at multiple thoracic and pelvic limb joints. Healthy dogs were evaluated at two sessions in a 24‐hour period. SCI‐affected dogs were evaluated 0, 14, 30, and 60 days after surgical decompression. Measurements were compared between sessions in healthy dogs and between healthy and SCI‐affected dogs using a Wilcoxon matched‐pairs signed rank test and a Mann Whitney test, respectively. P<0.05 was considered significant.

**Results:** Apart from the carpi, measurements were consistent across sessions in healthy dogs. Measurements were higher in SCI‐affected dogs for the carpi at day 0 (P<0.0003); for the carpi and stifles at 14 days (P<0.015); and for the carpi, stifles, and tarsi at days 30 and 60 (P<0.03).

**Conclusions:** Dynamometry measurements for most joints are consistent across sessions in healthy dogs, but differ between healthy and SCI‐affected dogs for at least 60 days after TL‐IVDE. The inconsistency in carpal measurements require further investigation. Dynamometry might represent a helpful quantitative measure of spasticity of pelvic limbs after SCI in dogs and could be used in future clinical studies.

## 35 Abstract N10

### 35.1 Thoracic Vertebrae: Proximity to Vital Structures and Implantation Corridor Measurements

#### 35.1.1 **Julianna Sabol**; Christopher Mariani, DVM, PhD, DACVIM (Neurology)

##### 35.1.1.1 Veterinary Hospital, North Carolina State University

**Background:** Thoracic vertebral stabilization is challenging due to unique vertebral anatomy and proximity to critical anatomical structures.

**Hypothesis/Objectives:** To quantify implantation corridor parameters of canine thoracic vertebrae and distances to adjacent critical structures.

**Animals:** 30 client‐owned dogs with a thoracic computed tomography (CT) study, grouped by weight (n=6/group; <5 kg, 5–10 kg, 10–20 kg, 20–40 kg, >40 kg).

**Methods:** Retrospective morphometric study. Implantation corridor parameters of thoracic vertebrae (T1‐T13) were measured (corridor length, width, angle from midline, angle of deviation) based on ideal trajectories, and distances to important adjacent structures were quantified.

**Results:** Implantation corridor (390 vertebrae) widths varied between 1.8 mm (T4, <5 kg) and 12.9 mm (T1, >40 kg), lengths between 8 mm (T13, <5 kg) and 33.8 mm (T5, >40 kg), angle from midline between 3° (T11, 20–40 kg) and 56° (T1, 5–10 kg), and angle of deviation between 3° (T7, <5 kg) and 45° (T13, 20–40 kg). Distances to vital structures were: subclavian artery (n=49; 5.8–40 mm), aorta (n=310; 0.8–43.4 mm), trachea (n=173; 1.3–43.5 mm), lungs (n=362; 0.4–28.3 mm), azygous vein (n=223; 0.3–21.7 mm).

**Conclusion and Clinical Importance:** Thoracic vertebrae are in close proximity to vital anatomical structures and have narrow implantation corridors with steep angles of ideal trajectory, which vary considerably by vertebra and dog size. Accurate implantation is necessary to avoid serious complications from invading adjacent anatomical structures, spinal canal breech, or ineffective implant placement.

## 36 Abstract N11

### 36.1 Prevalence and Clinical Features of Thoracolumbar Intervertebral Disc‐Associated Epidural Hemorrhage in Dogs

#### 36.1.1 **Jenni Bridges** ^1^; Rebecca Windsor^1^, DVM, DACVIM; Samuel Stewart^2^, DVM, DACVECC; Lori Fuerher‐Senecal^1^, CVT, VTS (AVTAA, AIMVT Neurology); Chand Khanna^2^, DVM, PHD, DACVIM (ONC), DACVP (HON)

##### 36.1.1.1 ^1^Wheat Ridge Animal Hospital; ^2^Ethos Veterinary Health

**Background:** Intervertebral disc‐associated epidural hemorrhage (EH) in dogs is a poorly understood neurological condition.

**Objective:** To compare the signalment, clinical presentation, MRI changes, and clinical outcome of dogs with acute thoracolumbar intervertebral disc herniation (TL‐IVDH) with and without EH.

**Animals:** 160 client‐owned dogs that underwent MRI and hemilaminectomy for acute TL‐IVDH at a private practice in Colorado, including 63 dogs with EH and 97 dogs without EH.

**Methods:** Retrospective review of medical record data from 160 dogs presenting sequentially to a single practice with acute TL‐IVDH that underwent MRI and hemilaminectomy surgery.

**Results:** 63 of 160 (39%) dogs had confirmed EH. French Bulldogs were significantly over‐represented (23/63; OR 4.1; 95% CI: 1.8–9.0; p<0.001) of the EH cases. Dogs with EH were more likely to present with clinical signs under 48 hours than dogs without EH (24–48 hours vs 48–72 hours; OR 2.4; 95% CI: 1.2–4.6; p=0.023) and were more likely to be non‐ambulatory on presentation (OR 2.1; 95% CI: 1.0–4.1; p=0.044). MRI showed that dogs with EH were more likely to have <50% cross‐sectional spinal cord compression than dogs without EH (OR 2.3 vs. 0.4; 95% CI 1.2–4.4 and 0.2–0.9, respectively), longer longitudinal spinal cord compression (3 spaces vs 1 space, p<0.001), and greater intrinsic spinal cord change (grade 3/severe vs grade 1/mild; p<0.001). The location of the intervertebral disc herniation in French Bulldogs with EH was more likely to be thoracolumbar (OR 10.8; 95% CI: 2.1–55.7; p=0.033).

**Conclusions and Clinical Importance:** French Bulldogs have a high prevalence of intervertebral disc‐associated EH. Dogs with EH have a shorter clinical course, are more likely to present non‐ambulatory, and have milder compression with more severe intrinsic spinal cord changes on MRI.

## 37 Abstract N12

### 37.1 Physical Activity is Associated with Better Cognitive Function: Results from the Dog Aging Project

#### 37.1.1 **Kiersten K. Forsyth** ^1^; Emily Bray^2^, PhD; David Raichlen^3^, PhD, MA; Gene Alexander^4^, PhD; Daniel Promislow^5^, PhD; Evan MacLean^6^, PhD

##### 37.1.1.1 ^1^College of Veterinary Medicine and Biomedical Sciences, Texas A&M University; ^2^Postdoctoral Research Associate, Arizona Canine Cognition Center, School of Anthropology, University of Arizona; ^3^Professor of Biological Sciences and Anthropology, Human and Evolutionary Biology Section, Department of Biological Sciences, University of Southern California; ^4^Professor, Department of Psychology, University of Arizona; ^5^Professor, Co‐director and PI of the Dog Aging Project, Department of Laboratory Medicine and Pathology, School of Medicine, University of Washington; ^6^Director, Arizona Canine Cognition Center, School of Anthropology, University of Arizona

**Background:** Canine Cognitive Dysfunction (CCD) is a form of dementia that shares many similarities with Alzheimer's Disease. In humans, physical activity is believed to reduce risk of Alzheimer's Disease. We explored the association between physical activity and cognitive health in a population of companion dogs, aged 6–18 years.

**Hypothesis/Objectives:** We hypothesized that physical activity would be associated with improved cognitive function and decreased risk of dementia, and that this association would remain robust when controlling for age, comorbidities, and potential confounders.

**Animals:** Our sample included 11,574 companion dogs enrolled through the Dog Aging Project. Of these, 287 had scores over the clinical threshold for CCD.

**Methods:** In this observational, cross‐sectional study, we used owner‐reported questionnaire data to quantify dog cognitive health (via the Canine Cognitive Dysfunction Rating scale), physical activity levels, health conditions, training history, and dietary supplements. We fit linear regression models with measures of cognitive health as the outcome, and physical activity—with several important covariates—as predictors.

**Results:** We found a significant negative relationship between physical activity and current severity of cognitive symptoms (estimate =‐0.12, p<0.001), magnitude of worsening over a 6‐month interval (estimate =‐0.09, p<0.001), and whether a dog reached a clinical level of CCD (odds ratio =‐0.67, p<0.001).

**Conclusions and Clinical Importance:** Physical activity was robustly associated with higher cognitive functioning in dogs; to help determine causality, future studies can follow the same dogs longitudinally or administer physical‐activity based interventions.

## 38 Abstract N13

### 38.1 Frequency of MRI Abnormalities in a Population of Dogs Referred to Veterinary Neurologists

#### 38.1.1 **Jacob S. Leslie** ^1^; Amanda Taylor^2^, DVM, CVA, DACVIM (Neurology); Laura Selmic^3^, BVetMed (Hons), MPH, DACVS‐SA, DECVS; Simon Platt^4^, BVMS, MRCVS, DACVIM (Neurology), DECVN

##### 38.1.1.1 ^1^MedVet Pittsburgh; ^2^BluePearl Specialty and Emergency Hospitals; ^3^Department of Veterinary Clinical Sciences, College of Veterinary Medicine, The Ohio State University; ^4^Small Animal Medicine and Surgery, College of Veterinary Medicine, University of Georgia

**Background:** Frequency of findings in canine brain magnetic resonance imaging (MRI) after referral to neurologists has not been reported. Multiple studies have focused on radiologist interpretations without association to confirmed neurological dysfunction.

**Hypothesis/Objectives:** Study objectives were to determine frequency of brain MRI findings in dogs with signs of intracranial disease from two referral institutions and agreement between neurologists reviewing MRIs. We hypothesized there would be no difference in frequency of MRI findings between institutions and neoplastic lesions would be the most frequent MRI diagnosis.

**Animals:** A cohort of 300 randomly selected dogs undergoing brain MRI to determine underlying cause of primary neurological presentations.

**Methods:** Medical records of dogs from two tertiary‐care institutions were collected retrospectively from 2014–2019. Brain MRIs were reviewed blindly/independently by two ACVIM board‐certified neurologists. Lesion characteristics and suspected MRI diagnosis based on published criteria were described.

**Results:** An intracranial MRI abnormality was determined in 64–71% of cases. Neoplastic (38–42%) and inflammatory (26–33%) lesions were the most common differentials. Seizures were noted in 52% of cases; 31% of dogs with seizures had normal MRIs. Interobserver agreement on abnormal scans was considered very good (Κ=0.764)

**Conclusions and Clinical Importance:** At least 29% of dogs with intracranial disease have normal MRIs. The ability of neurologists to differentiate and agree on normal versus abnormal MRIs is very good. Neoplastic and inflammatory were the most common MRI diagnoses. This data may help guide discussion with clients on expectations before imaging, and aid in empiric therapy decisions.

## 39 Abstract N14

### 39.1 The Relationship Between Activity Patterns and Cognition, Pain, and Motivation in Senior Dogs

#### 39.1.1 **Alejandra Mondino Vero** ^1^; Andrea Thomson^2^; Sara Giovagnoli^3^; Katharine Russell^4^, DVM; Margaret Gruen^5^, DVM, MVPH, PhD, DACVB; B. Duncan Lascelles^6^, BSc, BVSc, PhD, FRCVS, CertVA, DSAS (ST), DECVS, DACVS; Natasha Olby^7^, VetMB, PhD, MRCVS, DACVIM (Neurology)

##### 39.1.1.1 ^1^Department of Clinical Sciences, College of Veterinary Medicine, North Carolina State University; ^2^Vet Tech, Department of Clinical Sciences, North Carolina State University; ^3^Assistant Professor, Department of Psychology, Universita Di Bologna; ^4^NCSU; ^5^Assistant Professor, Behavioral Medicine, NCSU; ^6^Professor, Clinical Sciences, NCSU; ^7^Professor, Clinical Sciences, North Carolina State University

**Background:** Senior dogs undergo cognitive decline and suffer increasing pain, potentially altering activity. Additionally, this decline in quality of life could alter motivation to exercise. Changes in activity and sleep‐wake cycle have health consequences and influence the human‐animal bond but data on this topic are scarce.

**Objective:** Determine the relationship between activity patterns and cognition, pain, and motivation in senior dogs.

**Animals:** 32 senior dogs (12.48±1.50 years old) participating in the Longitudinal Study of Neuro‐Aging in Dogs were included in this study.

**Methods:** Weekday activity was evaluated over a 2‐week period using accelerometers. Cognitive status was assessed with the CAnine DEmentia Scale (CADES). Total pain (joint + spinal pain) was scored by physical examination. A motivation score was generated using a ratio of on‐leash/off‐leash gait speed moving towards a treat. The interaction between these parameters and activity was evaluated using functional linear modeling.

**Results:** Median (range) CADES and pain scores were 10 (0–70) and 8 (1–19) respectively; mean ± SD motivation score was 1.72±0.64. Dogs with lower CADES scores were more active between 5:30 p.m. and 8:00 p.m. Greater pain was associated with higher activity during sleep time (1:30–3:00 a.m.), and with lower activity in the early‐morning (4:00–5:30 a.m.). Finally, motivated dogs were more active between 10:00 a.m. to 2:00 p.m. and 5:00 p.m. to 8:00 p.m. (Figure 1).

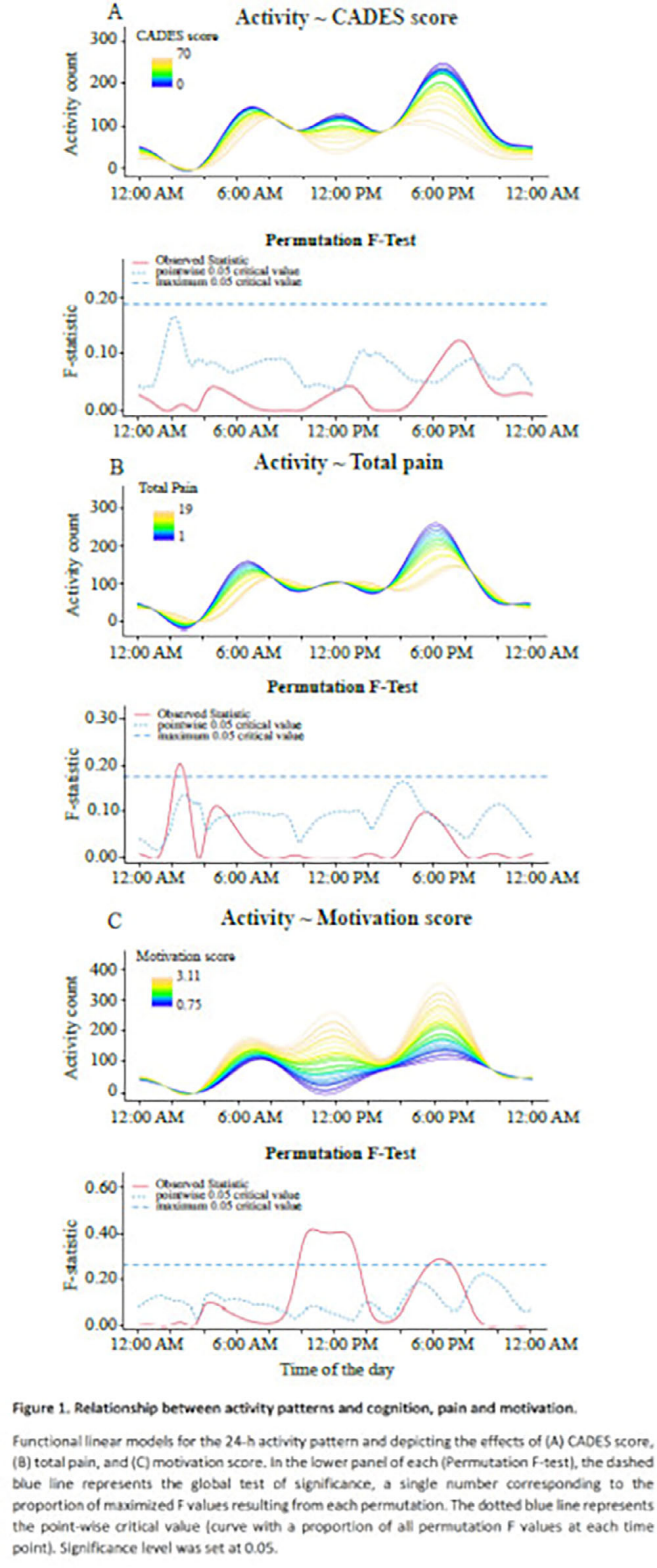

**Conclusion:** Strategies to reduce cognitive dysfunction and pain and increase motivation could improve activity patterns including quieter rest at night and greater diurnal activity.

## 40 Abstract N15

### 40.1 Alpha‐Chloralose Poisoning in Cats: Clinical Presentation in 24 Confirmed Cases

#### 40.1.1 **Cecilia Tegner** ^1^; Sandra Lundgren^1^, DVM; Kristoffer Dreimanis^1^, DVM; Annica Tevell Åberg^2^, PhD; Ulrika Windahl^3^, DVM, PhD

##### 40.1.1.1 ^1^University Animal Hospital, Swedish University of Agricultural Sciences; ^2^Department of Medicinal Chemistry, Analytical Pharmaceutical Chemistry; ^3^Swedish National Veterinary Institute

**Background:** Alpha‐chloralose (AC) is an anesthetic compound approved as a neurotoxic rodenticide in the USA since 2019. Reports on AC poisoning in companion animals are scarce. Alpha‐chloralose was previously approved in Sweden but following a surge of suspected cases of accidental AC poisoning of cats in 2019 this study was initiated, and layman use of AC was subsequently banned.

**Objectives:** To describe the clinical presentation of AC poisoning in cats.

**Animals:** Sixty‐four client‐owned, outdoor cats with suspected AC poisoning presenting to a teaching hospital in October 2019–February 2020.

**Methods:** Of 64 cats presenting with a strong clinical suspicion of AC poisoning, 24 cats were included in the case series, based on availability of surplus serum. A novel, quantitative UHPLC‐MS/MS analysis was developed and used to detect AC. Clinical signs described in the literature were tallied for each cat and disease severity was scored using set criteria.

**Results:** Alpha‐chloralose poisoning was confirmed in all 24 cases. The most common signs of intoxication were ataxia (100%), tremor (100%), cranial nerve deficits (96%), hyperesthesia (88%), bradycardia and somnolence (79% respectively). Hypothermia, previously reported in >90% of AC poisoned cats, was seen in only 38%. Disease severity differed from previous studies, with our population presenting with less severe disease, and no mortalities.

**Conclusions and Clinical Importance:** The present study describes the clinical presentation of AC poisoning in cats. Recognizing AC poisoning as a differential diagnosis in acute onset neurological signs may influence clinical decisions, and ultimately the outcome for the patient.

## 41 Abstract N16

### 41.1 Evaluating the Benefits of Cannabidiol for Analgesia Following Surgery for Intervertebral Disc Herniation in Dogs

#### 41.1.1 **Hilary L. Wright** ^1^; Annie Chen^1^, DVM, MS, DACVIM (Neurology); Clifford Petit^1^, DVM; Morgan Brace^1^; Joseph Wakshlag^2^, DVM, DACVIM (Nutrition), DACVSMR

##### 41.1.1.1 ^1^Department of Veterinary Clinical Sciences, College of Veterinary Medicine, Washington State University; ^2^Department of Clinical Sciences, College of Veterinary Medicine, Cornell University

**Background:** The use of cannabidiol (CBD) has shown promise in the treatment of pain in dogs.

**Hypothesis/Objectives:** CBD‐rich hemp extract provided with gabapentin following surgery will result in improved analgesia and decreased opioid requirements compared to patients receiving gabapentin alone.

1. No major adverse effects will be observed with CBD rich hemp and gabapentin.

**Animals:** Dogs, with intact deep pain, presenting for acute intervertebral disc herniation.

**Methods:** Double‐blinded, randomized, placebo‐controlled study.

5 mg/kg CBD‐rich hemp provided as an equal mix of CBD and CBDA in sesame oil or placebo sesame oil were provided every 8 hrs in conjunction with oral gabapentin.

After surgery, all patients were given injectable methadone and started on the oral treatments. Patients were pain scored with a validated system every 2 hours, 24 hours, and every 4 hours from 24–48 hours. Rescue analgesia (methadone) was given if pain score was >5/20 and recorded each time.

**Results:** Twenty dogs completed the study without adverse events.

Average pain scores over 48 hours for the CBD group were2.9±1.1(median 2.7). Placebo average pain scores were 3.7± 4.3, median 4.3). Mann Whitney U analysis found no significant difference (p=0.054); yet a trend towards significance with CBD rich hemp was observed.

Average number of methadone rescues over 48‐hours in the CBD group was 2.8±2.5 (median 2); placebo group mean was3.66±2.45 (median 5). Mann Whitney U Test found no significant difference (p=0.535).

**Conclusion and Clinical Importance:** Further investigation of CBD as an adjunctive analgesic following spinal surgery in dogs is warranted.

## 42 Abstract N17

### 42.1 Electroencephalogram and Heart Rate Variability Changes After Transcutaneous Vagus Nerve Stimulation in Healthy Dogs

#### 42.1.1 **Gibrann Castillo** ^1^; Luis Gaitero^1^; Sonja Fonfara^1^; Gabrielle Monteith^1^; Christopher Czura^2^; Fiona James^1^

##### 42.1.1.1 ^1^Ontario Veterinary College; ^2^Convergent Medical Technologies, Inc.

**Background:** Transcutaneous vagus nerve stimulation (tcVNS) has been used to treat epilepsy in people and dogs. Measurable changes in the electroencephalogram and heart rate variability (HRV) are reported in people using tcVNS.

**Hypothesis/Objectives:** Objective changes in brain activity and HRV will be detected by electroencephalography (EEG) and electrocardiography (ECG), respectively, after cervical tcVNS in dogs.

**Animals:** 6 client‐owned healthy dogs.

**Methods:** Prospective cohort feasibility study comparing the differences pre‐ and post‐tcVNS in frequency band power analysis (EEG) and HRV using Holter monitoring. tcVNS was applied at the maximal tolerable intensity; the animals’ reactions were noted.

**Results:** In the post‐stimulation EEG, the average power per channel was found to be significantly decreased in the theta (p=0.02) and alpha bands (p=0.04). Alpha (p<0.01), theta (p=0.01) and beta (p=0.035) frequencies post‐stimulation decreased significantly in the pooled power spectral analysis. The multivariate model did not detect any significant interaction between dog, attitude, and stimulation, neither within the same dog nor between individuals.

Holter monitoring revealed a significant increase in the HRV measured by the deviation of the inter‐beat (SDNN) index (p<0.01) and a decrease in mean heart rate (p<0.01) after tcVNS.

tcVNS was well tolerated by the animals without significant adverse effects reported.

**Conclusions and Clinical Importance:** This study suggests that tcVNS results in measurable changes in brain activity and HRV as detected by EEG and Holter monitoring, respectively, in dogs. Future investigations could confirm these findings in a larger cohort and objectively assess the potential clinical relevance of tcVNS.

## 43 Abstract N18

### 43.1 Expression of Hyperphosphorylated Tau in Dogs with Immune‐Mediated Meningoencephailitis

#### 43.1.1 **Yeon Chae** ^1^; Mingyun Son^2^; Taesik Yun^1^; Yoonhoi Koo^1^; Dohee Lee^1^; Sanggu Kim^1^; Soochong Kim^1^; Mhan‐Pyo Yang^1^; Hakhyun Kim^1^; Byeong‐Teck Kang^1^

##### 43.1.1.1 ^1^Chungbuk National University; ^2^CBNU

**Background:** Tau protein is a microtubule‐associated protein involved in the assembly and stabilization of the microtubules. The hyperphosphorylation of tau implicates microtubule stability, is demonstrated in the progression of multiple sclerosis (MS) in human medicine. MS is an autoimmune neurological disease and has many similarities with the meningoencephalitis of unknown etiology (MUE) in dogs, including pathological mechanisms.

**Objectives:** This study investigated the presence of hyperphosphorylated tau in dogs with MUE, and experimental autoimmune encephalomyelitis (EAE).

**Animals:** Total eight brain samples of the dogs were examined, including two neurological normal dogs, three dogs with MUE, and three canine EAE models.

**Methods:** Anti‐tau (phospho‐S396) antibody was used for immunohistochemistry (IHC), which stains hyperphosphorylated tau.

**Results:** In normal brain tissues, the expression of hyperphosphorylated tau was not identified. In all EAE dogs and one MUE dog, immunoreactivity for S396+ p‐tau was observed at glial cell cytoplasm and background in the periphery of the inflammatory lesion.

**Conclusions and Clinical Importance:** These result suggests that the first evidence that tau pathology may be involving neuroinflammation in the dogs, as in the human MS.

## 44 Abstract N19

### 44.1 Ondansetron in Dogs with Vestibular Nausea: A Double‐Blinded, Randomized, Placebo‐Controlled, Crossover Study

#### 44.1.1 **Lea Henze** ^1^; Sarah Foth^1^, Dr; Sebastian Meller^1^, Dr; Friederike Twele^1^, PhD; Marios Charalambous^1^, PhD; Hannah Kenward^2^, PhD; Jonathan Elliott^2^, PhD; Ludovic Pelligand^2^; Holger Volk^1^, PhD

##### 44.1.1.1 ^1^Tierärztliche Hochschule Hannover; ^2^Royal Veterinary College

**Background:** Nausea and emesis can be associated with vestibular system dysfunctions in dogs. Up to date, antiemetic drugs, such as maropitant and metoclopramide, are commonly used as therapeutic options but fail to address nausea.

**Hypothesis/Objectives:** The objective of the study was to show the efficacy of ondansetron, a 5‐HT receptor antagonist, against nausea in dogs with an acute vestibular syndrome.

**Animals:** Fourteen client‐owned dogs presented at the neurology service with clinical signs of a vestibular syndrome and nausea were included in the study.

**Methods:** A placebo‐controlled, double‐blinded, randomized, crossover‐study was designed to observe nausea before and after the administration of ondansetron. Dogs either received ondansetron or placebo (group 1). After the last behavioural assessment (2 hours later) dogs received then placebo or ondansetron respectively (group 2). The degree of nausea was scored with a former validated nausea score. In addition, arginine‐vasopressin‐serum levels, a biomarker of nausea, were determined.

**Results:** A reduction of the nausea score in the behavioral assessment was observed in all dogs. In group 1, the average score decreased significantly from 13.625 to 1.68 two hours after administration of ondansetron (p<0,0001) and in group 2 from 13.17 to 2.08 (p<0,001). The arginine‐vasopressin‐level decreased significantly four hours after administration (p<0,05).

**Conclusion and Clinical Importance:** Ondansetron showed a clinical resolution of nausea one hour after administration, while the arginine‐vasopressin level decreased four hours after administration. Ondansetron is likely beneficial for improving vestibular nausea in dogs.

## 45 Abstract N20

### 45.1 Towards a Mobile App for Automated Pain Assessment in Cats Using Deep Learning

#### 45.1.1 **Lea Henze** ^1^; Sebastian Meller^1^, Dr; Alexandra Schütter^1^, Dr; Anna Zamansky^2^, Dr; Marcelo Feighelstein^2^; Nora Dorn^1^; Holger Volk^1^, PhD

##### 45.1.1.1 ^1^Tierärztliche Hochschule Hannover; ^2^University of Haifa

**Background:** Objective pain assessment in cats is not routinely performed in veterinary practice. Many veterinarians lack the skill to objectively assess and, therefore, do not provide an adequate level of analgesia to painful cats. Automated tools based on Artificial Intelligence (AI) could provide a promising avenue to overcome these issues.

**Objective:** Our goal is to investigate, evaluate, and develop methods for automated facial analysis for pain assessment in cats that can be integrated in clinician's workflow.

**Methods:** An AI model based on Dino‐Resnet50 backbone transfer learning was trained on image dataset with 68 cats with pain and 68 cats without pain. All cats were scored using a validated pain score for cats.

We evaluated the model using a 10‐fold cross validation with 20% of train set for validation. Model was trained during 100 EPOCs with Adam optimizer and learning rate 0.0005.

**Results:** Model prediction reached accuracy of 0.64, precision of 0.64, and recall of 0.70.

**Conclusion and Clinical Importance:** The deep learning model shows promising preliminary results for binary classification of pain/no pain in cats based on images. To increase accuracy, more quality data is planned to be collected. More sophisticated pre‐processing methods, such as centering the cat's face, will be developed.

Assessing pain via an AI could clinically be beneficial as it guarantees objective pain scored independently of the examiner. This could lead to easier adjustments of the analgesic therapy to the patient's needs.

## 46 Abstract N21

### 46.1 Neutrophil‐to‐Lymphocyte Ratio as a Diagnostic Marker in Dogs with Meningoencephalitis of Unknown Etiology

#### 46.1.1 **Dohee Lee**; Jooyoung Park; Taesik Yun; Yoonhoi Koo; Yeon Chae; Hakhyun Kim; Mhan‐Pyo Yang; Byeong‐Teck Kang

##### 46.1.1.1 Laboratory of Veterinary Internal Medicine, College of Veterinary Medicine, Chungbuk National University

**Background:** The neutrophil‐to‐lymphocyte ratio (NLR) has been identified as a biomarker in several inflammatory and autoimmune diseases. It has been investigated that multiple sclerosis is associated with NLR in human patients.

**Hypothesis/Objectives:** To examine the diagnostic and prognostic value of the NLR in meningoencephalitis of unknown etiology (MUE) dogs.

**Animals:** Thirty‐eight MUE dogs, 20 hydrocephalus dogs, 10 brain tumor (BT) dogs, 32 idiopathic epilepsy (IE) dogs, and 41 healthy dogs.

**Methods:** This study was performed as case control study. Medical records were reviewed to identify dogs diagnosed with neurologic disease.

**Results:** The NLR was significantly higher in MUE dogs (median [interquartile range] = 6.08 [3.48–8.84]) than in healthy dogs (1.78 [1.36–2.20]; P<0.001). The NLR of MUE dogs was higher than that of IE (2.50 [1.58–3.43]; P<0.001) and hydrocephalus dogs (1.79 [1.32–2.83]; P<0.001), but not differed with that of BT dogs (4.27 [2.85–4.86]; P>0.99). The NLR of MUE dogs was significantly associated with brain lesion volume (P=0.02, r=0.37), but not with the survival time (P=0.31, r=0.17).

**Conclusions and Clinical Importance:** The NLR could be a biomarker for diagnosing MUE and distinguishing it from other intracranial diseases such as IE and hydrocephalus.

## 47 Abstract N22

### 47.1 Effects of Trazodone Administration on the Neurologic Examination in Healthy Dogs

#### 47.1.1 **Lizabeth C. Lueck** ^1^; Starr Cameron^2^, MS, BVETMED, DACVIM (Neurology); Natalia Zidan^2^, DVM

##### 47.1.1.1 ^1^Department of Medical Sciences, School of Veterinary Medicine, University of Wisconsin‐Madison; ^2^Clinical Assistant Professor, Department of Medical Sciences, Madison School of Veterinary Medicine, University of Wisconsin

**Background:** Trazodone is an oral anxiolytic used to reduce anxiety in dogs. Whether trazodone affects the neurologic examination in dogs has not been previously reported.

**Objectives:** To investigate whether trazodone administration is associated with changes in the neurologic examination in healthy dogs.

**Animals:** 32 healthy dogs between 1 and 6 years old, perceived by their owners as neurologically normal, with no previously diagnosed medical conditions.

**Methods:** Baseline sedation and anxiety assessments and neurologic examination were performed for each dog, followed by trazodone administration (6.25–8.60 mg/kg PO). The sedation and anxiety assessments and neurologic examination were repeated 1–2 hours after trazodone administration. The examinations were performed by a single board‐certified veterinary neurologist and were video‐recorded. The videos were randomized and reviewed by a different, masked neurologist who scored the exams.

**Results:** Seven of 32 (22%) dogs experienced worsening scores on neurologic exam after receiving trazodone, manifesting as new or progressive postural reaction deficits. Although not clinically significant, 18.75% of dogs had consciousness levels changed from bright, alert, responsive to quiet, alert, responsive after trazodone administration. There were no other changes present on neurologic exam. Sedation and anxiety scores were significantly changed post‐trazodone compared to pre‐trazodone (p=0.0001 and p=0.0001, respectively).

**Conclusions:** Most dogs did not have changes on neurologic examination after trazodone administration. However, approximately 1 out of 5 dogs have new or worsening postural reaction deficits after receiving trazodone. Ideally, trazodone should not be given prior to neurologic examination in dogs.

## 48 Abstract N23

### 48.1 Neurofilament Light Chain Concentrations in Dogs with Meningoencephalomyelitis

#### 48.1.1 **Christopher L. Mariani** ^1^; Laura Ruterbories^1^; Wojciech Panek^1^; Natasha Olby^1^; David Murdoch^2^

##### 48.1.1.1 ^1^North Carolina State University; ^2^Duke University

**Background:** Neurofilament light chain (NF‐L) is a neuronal structural protein that is released into the cerebrospinal fluid (CSF) and blood with neuronal injury and might be a useful biomarker of CNS disease in dogs.

**Hypotheses:** NF‐L concentrations are higher in dogs with meningoencephalomyelitis than in healthy dogs and are associated with clinical outcome.

**Animals:** 36 dogs with meningoencephalomyelitis from a hospital population and 24 healthy control dogs.

**Methods:** Retrospective case‐control study. Serum and CSF samples were obtained from a biobank. An ultra‐sensitive immunoassay was used to determine NF‐L concentrations. Survival times and information regarding neurological outcomes were obtained from the medical records.

**Results:** NF‐L concentrations were significantly higher in meningoencephalomyelitis dogs than in healthy controls for both serum (median 94.7 vs. 10.6 pg/ml; P<0.0001) and CSF (median 7,178.0 vs. 268.1 pg/ml; P<0.0001). However, matched serum and CSF concentrations in meningoencephalomyelitis dogs were not well correlated (Spearman's r=0.49, p=0.0589). We found no differences in CSF or serum concentrations between dogs that did or did not survive to hospital discharge (P=0.12 and 0.44 respectively) or between those surviving greater than or less than 3 months (P=0.31 and 0.66 respectively). Finally, we found no differences in CSF or serum concentrations between dogs that did or did not have persistent neurological deficits after treatment (P=0.31 and 0.99 respectively).

**Conclusion:** Dogs with meningoencephalomyelitis have markedly elevated CSF and blood concentrations of NF‐L compared to healthy controls. NF‐L was not associated with outcomes, although further study is required to adequately answer these questions.

## 49 NUTRITION

## 50 Abstract NM01

### 50.1 Effect of Fasting versus Feeding on Selected Biochemical Analytes in 100 Healthy Dogs

#### 50.1.1 **Katarina C. Yi** ^1^; Johanna Heseltine^2^, DVM, MS, DACVIM (SAIM); Nicholas Jeffrey^3^, BVSc, PhD, MSc, DECVN, DECVS, FRCVS; Audrey Cook^4^, BVMS, MSc, VetEd, MRCVS, DACVIM, DECVIM, DABVP (Feline Practice); Mary Nabity^5^, DVM, PhD, DACVP

##### 50.1.1.1 ^1^Texas A&M University; ^2^Clinical Associate Professor, Small Animal Clinical Sciences, Texas A&M University; ^3^Professor, Texas A&M University; ^4^Professor, Small Animal Clinical Sciences, Texas A&M University; ^5^Associate Professor, Department of Veterinary Pathobiology, Texas A&M University

**Background:** Fasting is often recommended prior to the collection of blood for routine biochemical analysis despite a paucity of evidence to support this requirement.

**Objectives:** To compare measurements of selected biochemical analytes collected before and after feeding in clinically healthy dogs.

**Animals:** 100 clinically healthy dogs weighing >15 kg.

**Methods:** Prospective observational study. Food was withheld for at least 10 hours. Preprandial serum was collected, and then dogs were fed their usual food at an amount equivalent to at least 2/3 resting energy requirement. Serum was collected at 2‐, 4‐, 6‐, and 8‐hours postprandially. The proportion of postprandial values that exceeded either the reported total allowable error (TEa), or for SDMA, the reference change value (RCV), was determined.

**Results:** The proportion of dogs with postprandial measurements that exceeded the TEa or RCV was 92/100 for triglycerides, 66/100 for BUN, 17/100 for glucose, 46/100 for phosphorus, 9/100 for bilirubin, 5/100 for SDMA, 2/100 for creatinine, and 0/100 for cholesterol and albumin. Of dogs with a postprandial result above the upper end of the reference interval, the proportion that had a fasted measurement above the reference interval was 3/36 for triglycerides, 7/17 for SDMA, 2/3 for creatinine, 8/10 for cholesterol, and 0/6 for glucose. Albumin and BUN never exceeded the reference interval, nor did postprandial lipase in dogs with normal fasted lipase.

**Conclusions and Clinical Importance:** Certain biochemical analytes may be impacted by feeding in healthy dogs. The impact in patients with illness requires investigation.

## 51 Abstract NM02

### 51.1 Comparing Dried Blood Spot and Lateral Flow Testing to Serum 25‐Hydroxyvitamin D Concentrations in Cats

#### 51.1.1 **Hannah Brodlie** ^1^; Jessica Quimby^1^; Rene Paschall^1^; Katelyn Brusach^1^; Hannah Klein^1^; Adam Rudinsky^1^; Huw Evans^1^; Phillip Lerche^1^; Daniel Gordon^2^; Gabriella Kratzer^1^; Jenessa Winston^1^; Valerie Parker^1^

##### 51.1.1.1 ^1^The Ohio State University; ^2^Colorado Animal Specialty & Emergency

**Background:** Several alternative tests to measure 25‐hydroxyvitamin D (25D) concentrations are commercially available, but they have not been assessed for agreement to standard methodology in cats.

**Hypothesis/Objective:** To compare three tests (two dried blood spot [DBS] tests and one lateral flow assay) to the gold‐standard measurement of serum 25D via liquid chromatography/tandem mass spectrometry: LC/MS/MS. We hypothesized that these tests would show good agreement with LC/MS/MS.

**Animals:** Six healthy purpose‐bred 2‐year‐old research cats (3 male neutered, 3 spayed female).

**Methods:** This was a prospective observational pilot study. Blood was collected at 6 timepoints over 6 weeks. Using two DBS tests and a lateral flow assay, whole blood 25D was measured and compared to serum 25D concentrations via LC/MS/MS. Agreement between methods was evaluated via Bland Altman analysis. The predetermined criterion for clinically significant variance in results was set at a difference of +/‐ 10 ng/mL or greater between the test being compared to the reference method.

**Results:** Both DBS tests and the lateral flow assay demonstrated a mean difference greater than the pre‐determined criterion for clinical significance.

Bland‐Altman analyses: Difference in 25D concentrations between methods.

**Table:** Method Comparison

	Bias	SD of bias	95% Limits of agreement
LC/MS/MS – DBS method 1	**14.67**	**7.19**	**0.5776‐ 28.76**
LC/MS/MS – DBS method 2	**‐13.16**	**3.917**	**‐21.23‐ ‐5.989**
LC/MS/MS – Lateral flow assay	‐20.09	5.067	**‐30.03‐ ‐10.16**

**Conclusions and Clinical Importance:** All three tests demonstrated poor agreement with serum LC/MS/MS concentrations. These novel methods cannot be recommended as an alternative to LC/MS/MS.

## 52 Abstract NM03

### 52.1 Hyperhomocysteinemia and Oxidative Stress in Greyhound Dogs

#### 52.1.1 **Kelsey L. Johnson** ^1^; Torrey Tiedeman^1^; Hannah Peterson^1^; Joerg Steiner^2^, med.vet., Dr.med.vet., PhD, DACVIM, DECVIM‐CA, AGAF^2^; Lauren Trepanier^3^, DVM, PhD, DACVIM, DACVCP

##### 52.1.1.1 ^1^University of Wisconsin‐Madison Veterinary Care; ^2^University Distinguished Professor (with Tenure), Small Animal Internal Medicine Dr. Mark Morris Chair in Small Animal Gastroenterology & Nutrition Director, Gastrointestinal Laboratory (GI Lab), Department of Small Animal Clinical Sciences, Texas A&M University; ^3^Melita Grunow Family Professor in Companion Animal Health Professor and Assistant Dean for Clinical and Translational Research, Department of Medical Sciences, University of Wisconsin‐Madison Veterinary Care

**Background:** Greyhounds have reported hyperhomocysteinemia (HHC) relative to other dog breeds. However, the underlying mechanisms for HHC in greyhounds, and its clinical implications, are not understood.

**Hypothesis/Objectives**

2 To determine whether HHC in clinically healthy greyhounds is associated with increased oxidative stress, as measured by plasma isoprostanes, compared to healthy non‐sighthound dogs.
3 To assess whether plasma homocysteine in healthy greyhounds correlates with low serum folate or cobalamin, which are possible mechanistic drivers of HHC.
4 To evaluate whether healthy greyhounds with HHC have decreased plasma concentrations of methionine, cysteine, or glutathione, which would support specific pathway defects.

**Animals:** 31 healthy, client‐owned greyhound dogs and 15 healthy, client‐owned non‐sighthound control dogs.

**Methods:** Prospective case‐control observational study. All dogs were screened with a physical exam, CBC, and chemistry profile. Homocysteine, folate, cobalamin, methionine, cysteine, reduced glutathione (GSH), and 8‐isoprostane‐F2 concentrations were measured in all dogs.

**Results:** Cysteine concentrations between greyhound (median 6.7 uM, range 1.9–14.2) and non‐sighthound dogs (7.9 uM, range 3.5–9.3) were not significantly different (P=0.36). Plasma GSH concentrations between greyhound (3.0 uM, range 0.6–7.1) and non‐sighthound dogs (2.6 uM, range 0.4–3.8) were also not significantly different (P=0.12). Data on folate, cobalamin, homocysteine, methionine, and 8‐isoprostane‐F2 concentrations are underway.

**Conclusions and Clinical Importance:** Greyhounds do not have significant differences in plasma cysteine and GSH concentrations compared to non‐sighthound dogs, suggesting that greyhounds do not have a defect in conversion of homocysteine to cysteine or GSH. Pending data will help elucidate other pathways of HHC and a possible association with oxidative stress.

## 53 Abstract NM04

### 53.1 The Effects of Storage Temperature and RNA*later* Solution on the Canine Fecal Microbiota

#### 53.1.1 Olivia W. Chiu^1^; Diego Gomez^2^, DVM, MSc, MVSc, PhD, DACVIM; Jennifer MacNicol^3^, BscH, MSc; Adronie Verbrugghe^4^, DVM, PhD, DECVCN

##### 53.1.1.1 ^1^University of Guelph; ^2^Assistant Professor, Clinical Studies, Ontario Veterinary College; ^3^PhD Candidate, Clinical Studies, University of Guelph; ^4^Associate Professor, Clinical Studies, Ontario Veterinary College

**Background:** Canine fecal microbiota profiling provides insight into host health and disease. Standardization of methods for handling, collection, storage, and analysis of fecal samples for microbiomics is currently inconclusive, however.

**Hypothesis/Objectives:** To investigate the effects of storage temperature and addition of RNA*later* on the microbiota profile of canine fecal samples. We hypothesized that storage temperature and the addition of RNA*later* would alter the alpha diversity and relative abundances of the microbial population.

**Animals:** Four intact female and two intact male healthy colony beagles, 1 year of age.

**Methods:** Fecal samples were homogenized and aliquoted within 15 minutes of voiding. Aliquots were subjected to four treatments: fridge (4°C), fridge + RNA*later*, freezer (‐20°C), and freezer + RNA*later*. DNA extraction, PCR amplification, then sequencing (Illumina MiSeq) were completed on all samples. Inverse Simpon, Shannon evenness, and Chao1 indexes calculated alpha diversity. Wilcoxon rank sum tests compared these indexes and relative abundances between groups.

**Results:** Samples with RNA*later* added had higher relative abundance of 5 genera (*Prevotella*, *Fusobacterium*, *Phocaeicola*, Bacteroidales, *Anaerobiospirillum*) and lower relative abundance in 4 genera (*Peptacetobacter*, *Steptococcus*, *Romboutsia*, *Faecalibacterium*) (P<0.05). No differences in relative abundance were detected between fridge and freezer samples, and alpha diversity did not differ between any treatments.

**Conclusions:** The addition of RNA*later* to fresh samples affect the relative abundance of some taxa present in the canine fecal microbiota.

## 54 Abstract NM05

### 54.1 Acute Dietary Choline Chloride Toxicity in Cats: A Case Series Report

#### 54.1.1 **Leslie B. Hancock** ^1^; Laura Gaylord^2^, DVM, DACVN

##### 54.1.1.1 ^1^The J.M. Smucker Co.; ^2^Consultant, Whole Pet Provisions

**Background:** Choline, an essential nutrient for cats, is a precursor of phosphatidylcholine, acetylcholine, and betaine. The Association of American Feed Control Officials (AAFCO) and the National Research Council (NRC) dietary recommendations for adult cats’ are 2,400 mg/kg and 2,550 mg/kg choline dry matter (DM) respectively without suggested maximums. The European Food Safety Authority (ESFA) previously deemed it virtually non‐toxic.

**Objective:** This case series describes the signalment, presentations, laboratory changes and outcomes of cats with choline chloride toxicity.

**Animals:** Medical records from 19 adult pet cats with confirmed exposure to recalled canned cat food.

**Methods:** Record reviews from January 1, 2020–January 15, 2021, report signalment, examinations, treatment, and outcomes. Data analysis was performed with JMP®, Version 16. SAS Institute Inc., Cary, NC, 1989–2021.

**Results:** Signalment: 9 neutered males, 7 ovariohysterectomized females, 3 unreported; weight (mean; SD): 5.0; ±1.5 kg; ages 1–12 years old.Observations: tachycardia (100%), vomiting (95%), ataxia (58%), fishy odor (47%), tachypnea (42%), ptyalism (42%), inappetence (26%), lethargy (21%), diarrhea (21%), abdominal pain (21%), tremors (10%) and mydriasis (5%). Onset occurred 2–96 hours post‐exposure; duration 12 hours to 5 days (excluding death/euthanized). Biochemical abnormalities: table 1. Radiographs: (n=4) gas‐distended intestines. Food analysis: 66,157–164,010 mg/kg choline DM.

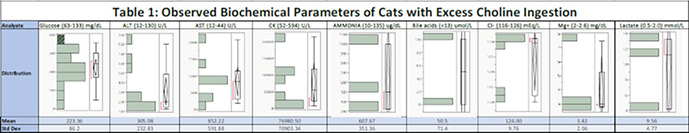

Treatments: supportive IV fluids, heat, oxygen, antiemetics, hepato‐protectants, antibiotics, neurotransmitter inhibitors, muscle relaxants and enemas.

Outcomes: 15/19 recovered within 5 days. 4 died/euthanized within 24 hrs.

**Conclusions and Clinical Importance:** Recognizing choline and probable acetylcholine toxicity may improve treatment and outcomes. Choline's safe limit <66,000 ppm DM.

## 55 Abstract NM06

### 55.1 Canine Non‐Traditional Diets Impact the Immunological Health of the Gastrointestinal Tract

#### 55.1.1 **Lara A. Sypniewski** ^1^; Kris Hiney^2^; Pratyay Rudra^3^; Dianne McFarlane^4^

##### 55.1.1.1 ^1^College of Veterinary Medicine, Oklahoma State University; ^2^Associate Professor, Department of Animal and Food Sciences, Oklahoma State University; ^3^Assistant Professor, Department of Statistics, Oklahoma State University; ^4^Department Chair and Professor, Large Animal Clinical Sciences, College of Veterinary Medicine, University of Florida

**Background:** Non‐traditional dog foods (raw meat‐based diets (RMBD), dehydrated, freeze‐dried) are becoming popular feeding choices. Limited data exists characterizing the impacts on gastrointestinal health and systemic inflammation in dogs fed a non‐traditional diet versus a traditional kibble diet (KD).

**Hypothesis/Objectives:** Serum and inflammatory markers were evaluated in dogs fed either KD or RMBD for >1 year. We hypothesized that RMBD fed dogs would have an improved intestinal and systemic inflammatory profile.

**Animals:** 55 healthy, adult client‐owned dogs fed either KD (n=27) or RMBD (n=28) for >1 year.

**Methods:** Dogs were grouped according to current feeding method (raw or kibble). Each group received a standardized diet (single brand of kibble or RMBD, with single ingredient snacks) for an additional 30 days following inclusion. Serum and feces were collected on day 30. Serum IgG, CRP, galactin‐3, soluble receptor for advanced end glycation end products (sRAGE), and fecal intestinal alkaline phosphatase (IAP), IgA and IgG were evaluated. Linear regression and correlation analysis were used to statistically analyze the data and log‐transformation was used when necessary.

**Results:** Fecal IAP, IgA and IgG levels were significantly higher in the RMBD group compared to the KF group (p<0.0004, p<0.0006, p<0.0002, respectively). No difference was observed in serum galactin‐3, IgG, CRP or sRAGE between groups.

**Conclusions and Clinical Importance:** Dogs fed RMBD for 1 year have increased fecal IAP, IgA and IgG which may reflect improved gastrointestinal health and local immune function.

## 56 Abstract NM07

### 56.1 Repertoriating Raw Food Companies in Quebec for Domestic Animals

#### 56.1.1 Alexandra Gagnon; Younes Chorfi

##### 56.1.1.1 University of Montreal

**Background:** Feeding raw food diets is popular among dog and cat owners. In Quebec, Canada, small animal raw food industry has not been explored.

**Hypothesis/Objectives:** This study was performed to draw a portrait of the small animal raw food industry in Quebec, Canada.

**Animals:** No animals were used in this study.

**Methods:** All the companies producing raw food products for dogs and cats registered in Quebec were contacted by phone or email to answer the following questions: which species (dog or cat) and which life stage the raw food diets are for? Is the product formulated to meet the nutritional levels established by the AAFCO (dog/cat)? Who is responsible for the diet formulation? Has the product undergone a feeding trial? Are bacteriological tests carried out on a regular basis? How many employees are working in the company? Does the company export its products abroad?

**Results:** 23 companies producing raw food diets for small animals are registered in Quebec. Among them 18 answered the questions. Most companies produce raw food for dogs and cats (14/18), most companies follow AAFCO guidelines (14/18), “All life stages” was the most represented (12/18), agronomists were often responsible for product formulation (11/18), the number of employees varies from 2 to 70, a few companies export outside of Quebec (6/14), feeding trials are rarely done (2/16), and bacteriological controls are often performed (10/13).

**Conclusions and Clinical Importance:** These results contribute to a better understanding of the small animal raw food industry in Quebec, Canada.

## 57 Abstract NM08

### 57.1 Evaluating the Causes and Consequences of Canine Food Motivation Within the Dog Aging Project Pack

#### 57.1.1 **Kathleen Gartner** ^1^; Kellyn McNulty^2^; Annette Fitzpatrick^3^; Audrey Ruple^4^; Zihan Zheng^5^; Kate Creevy^2^

##### 57.1.1.1 ^1^Texas A&M University; ^2^Small Animal Clinical Sciences, Texas A&M University; ^3^Epidemiology, Family Medicine, University of Washington; ^4^Population Health Sciences, Virginia Tech; ^5^Biostatistics, University of Washington

**Background:** Canine obesity is a complex multifactorial disease. While recent data suggest that eating behavior in dogs influences the development of obesity, little is known about the particular factors that may affect a dog's eating behavior.

**Objectives:** To evaluate the impact of dog food motivation and owner food management, as scored by the Dog Obesity Risk Assessment (DORA) questionnaire, on canine obesity, and to investigate the impact of demographic and environmental factors on dog food motivation.

**Animals:** USA dogs (n=410) whose owners elected to participate in the Dog Aging Project.

**Methods:** Owners (n=410) completed the DORA questionnaire and assigned their dog a body status (overweight or not overweight).

**Results:** Owner‐assigned overweight body status was positively associated with owner management score (OMS) (p<0.001) and food motivation score (FMS) (p=0.016) with an odds ratio of 1.05 (95%CI: 1.027–1.074) and 1.02 (95%CI: 1.003–1.03), respectively. Dog FMS varied among breed groups (p<0.001) with the highest average FMS in the Sporting group. None of the other evaluated demographic or environmental factors had a significant association with FMS.

**Conclusions and Clinical Importance:** Owners of overweight dogs have heightened feeding management practices, which contradicts the supposition that owner laxity in feeding practices contributes to canine obesity. Certain breed groups have an innately higher food motivation. Since food motivation is associated with overweight body status, one factor contributing to obesity development in these dogs may be their heightened drive to eat.

## 58 Abstract NM09

### 58.1 Efficacy and Tolerability of a Novel Phosphate‐Binder Supplement in Cats

#### 58.1.1 **David Griffin** ^1^; Robert Gillette^2^, DVM, MSE, DACVSMR; Rebekah Strunk^3^, MS; Carolyn Warner^4^, RVT, LVT; Denise Passmore^5^, PhD

##### 58.1.1.1 ^1^Nutramax Laboratories; ^2^Clinical Research Director, Nutramax Laboratories; ^3^Business and Scientific Assessment Manager, Nutramax Laboratories; ^4^Clinical Research Manager, Nutramax Laboratories; ^5^Scientific Technical Writer, Nutramax Laboratories

**Background:** Phosphate is an essential mineral for cats. However, excess can distress renal function in cats with CKD. Phosphate binders have shown reductions in renal phosphate load.

**Hypothesis/Objectives:** This study was a proof‐of‐concept assessing the ability of a novel mineral‐based (iron and calcium) phosphate binder to bind phosphate through gastrointestinal absorption in healthy cats.

**Animals:** This study was comprised of a laboratory population of 21 healthy, neutered male cats.

**Methods:** Cats were randomly distributed into three groups and fed standard diet twice daily for 29‐days.Two groups received 1 (Group 1) or 2 (Group 3) phosphate binder capsules with each meal, and an additional group (Group 2) served as a control. Blood was drawn on days ‐5, 6, 13, 20, and 27. Feces and urine were pooled for analysis every 72 hours beginning on day 6. Two‐way repeated‐measures ANOVA was performed. All relevant blood, urine, and fecal parameters were grouped by cat, dosing group, and time factors.

**Results:** Urinalysis showed significant reductions of phosphate in groups 1 and 3 (p=0.020; Figure 1) compared to pre‐study levels. There were no significant changes in CBC and chemistry measures. Food amounts and body weight were measured throughout. There were no significant differences in the cats’ body‐weight or food consumed, indicating acceptance of material with food.

**Figure:**
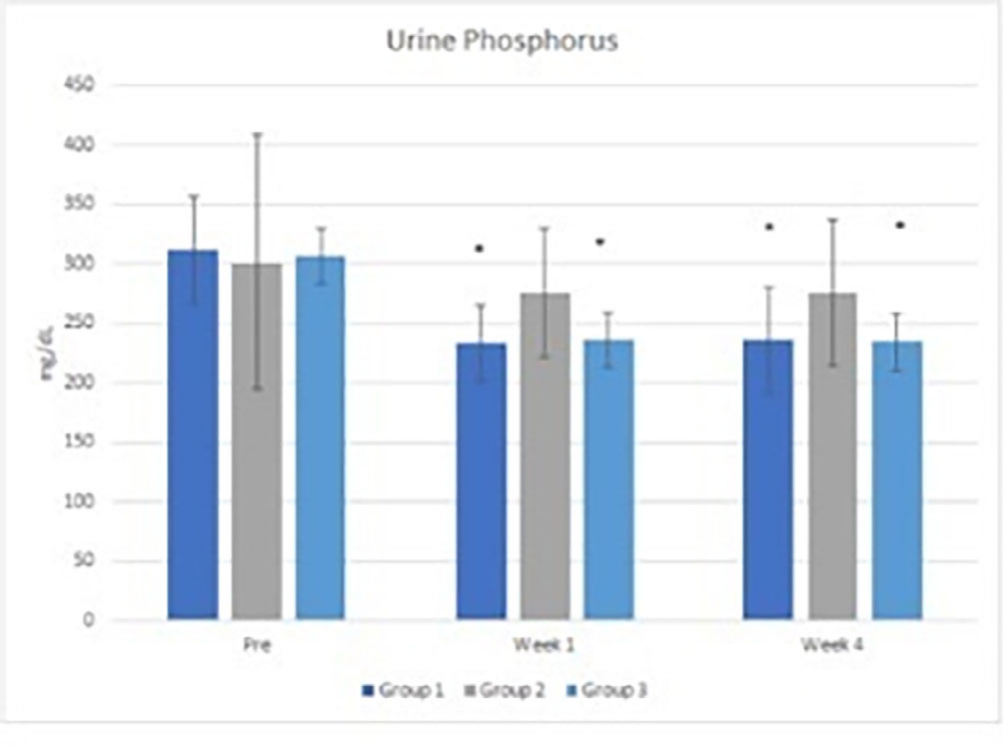
Figure 1

Pooled urine showed a significant decrease (p=0.020) in phosphorus for Groups 1 and 3 in weeks 1 and 4 when compared to the pre‐study

**Conclusions and Clinical Importance:** The study demonstrated the safety of the phosphate binder even at larger amounts. Nevertheless, the study supports, one capsule with a meal is a recommended starting point for administering to cats with CKD.

## 59 Abstract NM10

### 59.1 Low Carbohydrate Diet Reduces Circulating Inflammatory Gene Expression in Dogs with Chronic Gastroenteritis

#### 59.1.1 **Pooja Gupta‐Saraf** ^1^; Brooke Wichterman^2^; Matthew Jackson^3^; Kiran Panickar^4^, PhD

##### 59.1.1.1 ^1^Hill's Pet Nutrition; ^2^Associate Scientist, Hill's Pet Nutrition; ^3^Principal Scientist, Hill's Pet Nutrition; ^4^Manager, Life Science Laboratory, Hill's Pet Nutrition

**Background:** Companion dogs are susceptible to chronic gastroenteritis (CGE) and inflammation drives its pathogenesis. Restriction of dietary digestible carbohydrate increases ketone production which has a direct anti‐inflammatory effect.

**Hypothesis:** A diet low in digestible carbohydrate (LoCHO) will reduce inflammatory markers in dogs with CGE.

**Animals:** Canines with CGE (n=18; diagnosed by histopathological assessment) and healthy controls matched for age, gender, and weight (n=19) were housed in Hills’ animal colony.

**Methods:** The study was approved by the institutional animal care and use committee (protocol CP864.0). Healthy and CGE dogs participated in a crossover study, being fed high carbohydrate (HiCHO) then LoCHO foods for 5 weeks each in random order. Macronutrients (P/F/C; % energy): HiCHO (26/21/43); LoCHO (26/62/4). Blood was collected at the end of each feeding phase. Gene expression was assessed by NanoString nCounter and data analyzed using JMP Pro and Ingenuity Pathway Analysis.

**Results:** Feeding LoCHO food decreased inflammatory gene expression compared to HiCHO, including pathways responsible for phagocytosis, recruitment, and cellular infiltration (p=5.7E 03). LoCHO also inhibited immune cell trafficking. Food and health status interacted to influence the expression of 95 genes. Thus, JAM3, a tight junction protein, increased in CGE dogs when fed a LoCHO diet but did not in healthy dogs.

**Conclusions and Clinical Importance:** A reduction in inflammatory gene expression, including that for immune cell trafficking, in CGE dogs fed LoCHO is indicative that reduction in dietary carbohydrate may provide clinical benefit to CGE and could improve quality of life.

## 60 Abstract NM11

### 60.1 Implications of COVID‐19 Pandemic in the Feeding Habits of Dogs Domiciled in São Paulo, Brazil

#### 60.1.1 **Fabio A. Teixeira** ^1^; Tereza Trofimoff^2^, DVM; Vivian Pedrinelli^3^, DVM, MS; Mariana Porsani^4^

##### 60.1.1.1 ^1^School of Veterinary Medicine and Animal Science of University of Sao Paulo; ^2^Anclivepa's Veterinary College; ^3^School of Veterinary Medicine and Animal Science, University of Sao Paulo/Nutricare Vet, São Paulo, Brazil; ^4^Nutricare Vet

**Background:** The outbreak of COVID‐19 has impacted the whole world.

**Objectives:** To evaluate the implications of pandemic on feeding habits of dogs.

**Methods:** An online questionnaire was completed by dog owners of São Paulo, Brazil. Data were analyzed by descriptive statistics and McNemar test was used to compare profiles before and during the pandemic. Associations between variables were verified using chi‐square test.

**Participants:** 1188 dog owners.

**Results:** Weight gain occurred in 19.4% and weight loss in 5.5% of dogs. Physical activity decreased for 34.8% and increased for 14.3% of dogs (p<0.001). Daily food intake increased in 13.4% of dogs and decreased in 4.8%. 8% of owners changed their dog's diet type to reduce costs and 13.1% to make dogs lose weight. The number of animals that were fed homemade food defined by owner increased by 65.6% (p<0.001) and prescribed by a professional reduced by 43.5% (p<0.001). The number of owners who provided treats several times a day increased by 34.6% (p=0.004). Dogs ate more foods intended for human consumption, such as cookies (20% increase) and cake or bread (18% increase). There was an association between the health score attributed by owners to their own routine and to that of dogs before and during the pandemic (p<0.001).

**Conclusion:** There were changes in the feeding management of dogs during the pandemic, probably reflecting changes in the owners’ dietary profiles, which may have caused damage to pet's health. Dog's lifestyle is related to the environment and it is influenced by the historical context.

## 61 ONCOLOGY

## 62 Abstract O01

### 62.1 Retrospective Evaluation of Melphalan, Vincristine, and Cytarabine Chemotherapy for the Treatment of Relapsed Canine Lymphoma

#### 62.1.1 **Margaret E. Duckett** ^1^; Kaitlin Curran^1^; Shay Bracha^2^; Haley Leeper^1^

##### 62.1.1.1 ^1^Carlson College of Veterinary Medicine, Oregon State University; ^2^College of Veterinary Medicine, Texas A&M University

**Background:** The standard of care for treatment‐naive canine lymphoma is a multi‐agent protocol consisting of prednisone, vincristine, cyclophosphamide and doxorubicin. However, most patients relapse and combination chemotherapy can be an effective option for treating resistant lymphoma.

**Hypothesis/Objectives:** We hypothesized the combination of melphalan, vincristine and cytarabine (MOC) would be well tolerated and an effective treatment for dogs with relapsed or refractory lymphoma.

**Animals:** Twenty‐three dogs met the inclusion criteria for response and toxicity assessment. Two dogs were excluded from progression‐free survival (PFS) analysis. Both dogs had experienced clinical benefit after one cycle and were switched to a different protocol due gastrointestinal adverse events and owner preference.

**Methods:** Data was collected retrospectively. Dogs were treated with cytarabine and vincristine on day one. On day seven, dogs were treated with melphalan. This two‐week protocol was repeated for at least three cycles or until treatment failure.

**Results:** The overall response rate was 42%. The median PFS was 33 days. The overall clinical benefit was 74% for a median of 36 days. The majority of adverse events were mild and were hematologic or gastrointestinal in nature.

**Conclusions and Clinical Importance:** The combination of melphalan, vincristine and cytarabine is a safe treatment option for relapsed or refractory lymphoma in dogs. Additional studies are warranted to validate the potential benefit of the MOC protocol in a larger cohort and explore optimal dosing.

## 63 Abstract O02

### 63.1 Cytomorphologic Changes in Blood Leukocytes and Erythrocytes in Dogs Receiving CHOP Treatment for Multicentric Lymphoma

#### 63.1.1 **Joseph J. DiBenedetto** ^1^; Laura Van Vertloo^2^, DVM, DACVIM (SAIM); Margaret Musser^3^, DACVIM (Oncology); Chad Johannes^4^, DVM, DACVIM (SAIM, Oncology); Ariel Nenninger^1^, DVM; Jeremy Servantes^5^, DVM; Shannon Hostetter^6^, DVM; Austin Viall^7^, DVM, DACVP

##### 63.1.1.1 ^1^College of Veterinary Medicine, Iowa State University; ^2^Associate Professor, Department of Veterinary Clinical Sciences, Iowa State University; ^3^Assistant Professor, Department of Veterinary Clinical Sciences, College of Veterinary Medicine, Iowa State University; ^4^Associate Professor, Hospital Director, Department of Clinical Sciences, College of Veterinary Medicine, Colorado State University; ^5^Associate Veterinarian, Abraham's Equine Clinic; ^6^Associate Veterinarian, Department of Veterinary Clinical Sciences, College of Veterinary Medicine, University of Georgia; ^7^Associate Professor, Department of Veterinary Pathology, College of Veterinary Medicine, Iowa State University

**Background:** Multicentric canine lymphoma is frequently treated with the modified Madison‐Wisconsin CHOP protocol. Serial CBCs are performed to monitor patients for lymphoma‐associated disease and adverse chemotherapy‐associated hematologic effects. While certain chemotherapeutics are known to induce morphologic alterations in leukocytes and erythrocytes, chemotherapy‐induced abnormalities that may occur in dogs receiving CHOP have not been described. Identifying the leukocyte and erythrocyte cytomorphologic changes by lymphoma dogs throughout CHOP may help differentiate expected changes from adverse chemotherapy side‐effects or disease progression.

**Objectives:** The objectives of our study were to 1) assess differences in leukocyte and erythrocyte morphology between healthy matched‐control dogs and lymphoma dogs at diagnosis and 2) evaluate changes in cellular cytomorphology as lymphoma dogs progress throughout CHOP.

**Animals:** 21 dogs with lymphoma and 21 healthy, age‐breed matched control dogs from a university hospital population.

**Methods:** Retrospective descriptive study. Quantitative and qualitative leukocyte and erythrocyte data was collected from serial CBCs from dogs with lymphoma that completed 26‐week CHOP and matched controls. Differences in leukocyte and erythrocyte metrics at diagnosis, along with differences during CHOP therapy, were compared.

**Results:** No differences in leukocyte or erythrocyte cytomorphologic abnormalities were found between controls and lymphoma dogs at diagnosis. Changes in RBC count (p<0.0001), hemoglobin (p=0.00031), mean cell volume (p=0.0005), red blood cell distribution width (p<0.0001), and magnitude of polychromasia (p=0.0229) were found throughout CHOP therapy.

**Conclusion:** Our data suggests that RBC metrics and cytomorphologic changes consistent with regenerative erythropoiesis develop in lymphoma dogs being treated with CHOP.

## 64 Abstract O03

### 64.1 Detection of Canine Cancer Prior to Clinical Signs with Blood‐Based “Liquid Biopsy”

#### 64.1.1 **Andi Flory** ^1^; Lisa McLennan^1^; Betsy Peet^1^, RVT; Marissa Kroll^2^; Deirdre Stuart^3^; Devon Harris‐Taylor^4^; Stephanie Hossain^4^; Amber Wolf‐Ringwall^5^, DVM, PhD; Brenda Phillips^6^, DVM, DACVIM (Oncology); Mairin Miller^7^, DVM; Brenda Coomber^3^, PhD; Chelsea Tripp^4^, DVM, DACVIM (Oncology); Daniel Grosu^1^, MD, MBA; Dana W.Y. Tsui^1^, PhD; Ilya Chorny^1^, PhD; Susan Cho Hicks^1^, MAS; Jason Chibuk^1^, MS, CGC; Angela McCleary‐Wheeler^1^, DVM, PhD, DACVIM (Oncology); Gilberto Hernandez^1^, MPH, MBA; Kristina Kruglyak^1^, PhD; Jill Rafalko^1^, MS, CGC

##### 64.1.1.1 ^1^PetDx; ^2^Veterinary Specialty Hospital of San Diego; ^3^Ontario Veterinary College, University of Guelph; ^4^Bridge Animal Referral Center; ^5^College of Veterinary Medicine, University of Minnesota; ^6^Veterinary Specialty Hospital of San Diego; ^7^Veterinary Specialty Hospital of North County

**Background:** Guidelines‐driven screening protocols for early cancer detection in dogs are lacking, and cancer is often detected at advanced stages.

**Hypothesis/Objectives:** To examine how cancer is typically detected in dogs, and whether liquid biopsy could identify genomic abnormalities in patients with incidentally detected cancers.

**Animals:** Client‐owned dogs with definitive cancer diagnoses, enrolled in a clinical validation study for a novel blood‐based liquid biopsy test.

**Methods:** Medical records were reviewed for history and presenting complaint that led to definitive cancer diagnosis. Blood samples from these dogs were subjected to DNA extraction, proprietary library preparation, and next‐generation sequencing. Sequencing data were analyzed using an internally developed bioinformatics pipeline to detect genomic alterations associated with the presence of cancer.

**Results:** In a cohort of 358 cancer‐diagnosed subjects, 3% were detected during a wellness exam, 8% during other routine care (including pre‐dental evaluations and grooming), and 89% of cases were diagnosed after the owner reported clinical signs suggestive of cancer. Liquid biopsy results were available for at least 35 of the 41 subjects in which cancer was detected incidentally during a wellness exam or other routine care. The test returned a **Cancer Signal Detected** (positive) result in approximately 55% of subjects.

**Conclusions and Clinical Importance:** The vast majority of canine cancer is diagnosed when the patient is presented for evaluation following the onset of clinical signs. In the majority of patients with incidentally detected cancers, liquid biopsy delivered positive results, demonstrating its potential to serve as a novel cancer screening tool for dogs.

## 65 Abstract O04

### 65.1 Clinical Outcomes in Cats with Renal Carcinoma Undergoing Nephrectomy: A Retrospective Study

#### 65.1.1 **Shannon A. Kenny** ^1^; Matthew Cook^1^; Jennifer Lenz^3^; Karl Maritato^2^; Katherine Skorupski^4^; Stan Veytsman^5^; Brandan Janssens^6^; Mackenzie Pellen^8^; Catrina Silveira^7^; Laura Selmic^1^; Brian Husbands^1^

##### 65.1.1.1 ^1^College of Veterinary Medicine, The Ohio State University; ^2^MedVet Cincinnati; ^3^School of Veterinary Medicine, University of Pennsylvania; ^4^School of Veterinary Medicine, University of California; ^5^College of Veterinary Medicine, University of Minnesota; ^6^College of Veterinary Medicine and Biomedical Sciences, Colorado State University; ^7^College of Veterinary Medicine, Texas A&M University; ^8^School of Veterinary Medicine, University of Wisconsin‐Madison

**Background:** Feline renal carcinomas (RC) are uncommonly encountered. Limited information regarding clinical presentation, post‐surgical outcomes, and survival times are available.

**Hypothesis/Objectives:** The purpose of this multi‐institutional, retrospective study was to describe the presenting features and clinical outcomes of cats with RC treated with nephrectomy.

**Animals:** Thirty‐six client‐owned cats were included.

**Methods:** Medical records from participating institutions were searched to identify cats that underwent nephrectomy and had a histopathologic diagnosis of RC.

**Results:** The most common presenting complaints were weight loss (n=13) and hyporexia (n=11). Twenty‐eight cats survived to discharge (77.8%). Median progression free interval (PFI) could not be determined, as only 6 cats had suspected recurrence (16.7%) and 7 cats had suspected metastasis (19.4%). The median survival time (MST) for all cats was 203 days (95% CI: 84, 1379 days). When cases that died prior to discharge were excluded, MST increased to 1217 days (95% CI: 127, 1641 days). Preoperative erythrocytosis had a protective effect, as these patients had significantly longer MST (p=0.006). Pre‐surgical azotemia, renal tumor size, mitotic index, and tumor histologic subtype were not statistically prognostic.

**Conclusion and Clinical Importance:** For cats surviving to discharge, prolonged survival times were possible. Due to varied post‐operative treatment approaches, conclusions regarding the impact of chemotherapy could not be made. Further studies are necessary to elucidate other potential prognostic factors, the utility of adjuvant treatment, and to identify patients at‐risk in the perioperative period.

## 66 Abstract O05

### 66.1 Fibrinogen Degradation Products as a Clinical Biomarker for Cancer Diagnosis and Prognosis in Neoplastic Dogs

#### 66.1.1 **Chen‐Si Lin** ^1^; Chiao‐Hsu Ke^1^; Ka‐Mei Sio^2^; Yu‐Shan Wang^3^, PhD

##### 66.1.1.1 ^1^National Taiwan University; ^2^Department of Veterinary Medicine, National Taiwan University; ^3^Uni Pharma Co., Ltd

**Background:** Fibrinogen degradation product (FDP, DR‐70) has been used as a biomarker for cancer progression in humans for decades; however, few studies describing the clinical values of DR‐70 are available in veterinary medicine.

**Animals:** The purpose of this study is to investigate the correlation between DR‐70 values in the plasma and the development of various types of canine neoplasms. The plasma samples were collected from both normal (n=53) and tumor dogs (n=262) in National Taiwan University Veterinary Hospital.

**Methods:** The plasma DR‐70 concentrations were determined through a commercial DR‐70 enzyme‐linked immunosorbent assay (ELISA) assay.

**Results:** Expression levels of DR‐70 were significantly higher in cancer dogs (n=262, 2.140 μg/ml ± 0.6388 μg/ml) than those in tumor‐free individuals (n=53, 1.233 μg/ml ± 0.5426 μg/ml, *p*<0.0001). The area under curve of DR‐70 is 0.9089 under a 95% confidence interval from 0.8730 to 0.9466 with significant difference (*p*<0.0001). Furthermore, DR‐70 levels were positively correlated to the tumor progression whereas the values decreased with the responses to the treatment in numerous cases. These findings suggested that the canine plasma DR‐70 levels could be used as a biomarker for cancer patients. In conclusion, this study presents evidence that plasma DR‐70 levels are associated with cancer formation, therefore the increased levels of DR‐70 could be an effective cancer biomarker or canine tumor detection. This is the first study to show the clinical significance of DR70 in veterinary cancer medicine.

## 67 Abstract O06

### 67.1 Lymphoma Detection Using Blood‐Based Liquid Biopsy

#### 67.1.1 Christina McCleary‐Wheeler, DVM, PhD, DACVIM (Oncology); **Andi Flory**, DVM, DACVIM (Oncology); Kristina Kruglyak, PhD; John Tynan, PhD; Lisa McLennan; Jill Rafalko, MS, CGC; Katherine Lytle, DVM, MPH, MS; Daniel Grosu, MD, MBA; Lauren Holtvoigt, DVM, MBA; Jason Chibuk, MS, CGC; Susan Cho Hicks, MAS; Ilya Chorny, PhD; Dana W.Y. Tsui, PhD

##### 67.1.1.1 PetDx

**Background:** Lymphoma is a common and aggressive cancer in dogs.

**Hypothesis/Objectives:** To assess the performance of next‐generation sequencing‐based liquid biopsy testing in a cohort of lymphoma‐diagnosed dogs, with sub‐analyses by phenotype, stage, and substage.

**Animals:** Client‐owned dogs enrolled in a clinical validation study for a novel blood‐based liquid biopsy test.

**Methods:** Blood samples from 99 dogs with confirmed diagnoses of lymphoma were subjected to DNA extraction, proprietary library preparation, and next‐generation sequencing. Sequencing data were analyzed using an internally developed bioinformatics pipeline to detect genomic alterations associated with the presence of cancer.

**Results:** Liquid biopsy returned a positive result in 90 of 99 lymphoma‐diagnosed dogs, for an overall sensitivity of 90.9%. The test showed a 96.2% detection rate in patients with B‐cell (50/52), 88.9% in T‐cell (16/18), and 60% in indolent lymphoma (3/5); 24 patients had an “undefined” or “other” phenotype, and the detection rate was 87.5% (21/24) in these patients. There was no statistically significant difference in detection rate by cancer stage or substage across phenotypes. In 35 of the 99 lymphoma‐diagnosed dogs, a “Cancer Signal Origin” prediction of “lymphoma” was correctly provided.

**Conclusions and Clinical Importance:** Liquid biopsy was successful at detecting genomic alterations associated with cancer in over 90% of dogs with a diagnosis of lymphoma, and was further able to predict the presence of lymphoma (vs any other cancer type) in over one‐third of these patients. This test offers veterinarians a novel, noninvasive tool for the detection and characterization of lymphoma in dogs.

## 68 Abstract O07

### 68.1 Alkaline Phosphatase Cytochemistry in the Diagnosis of Canine and Feline Primary Pulmonary Neoplasia

#### 68.1.1 **Jack O'Day** ^1^; Davis Seelig^2^, DVM, PhD, DACVP; Mauren Emanuelli^3^, DVM, PhD; Daniel Heinrich^2^, DVM, DACVP

##### 68.1.1.1 ^1^University of Minnesota; ^2^Associate Professor, Veterinary Clinical Sciences, University of Minnesota; ^3^Veterinarian, Small Animal Medicine, Veterinary Teaching Hospital, Federal University of Santa Maria

**Background:** Distinguishing primary and secondary pulmonary neoplasia can be challenging via cytology. No rapid, inexpensive diagnostic tool to aid in this distinction is currently available. Alkaline phosphatase cytochemistry (ALP‐CC) has been used in people to identify primary pulmonary carcinomas. We hypothesized this approach could be applied to canine and feline lung aspirates.

**Objectives:** The primary objectives of this study were to evaluate the utility of ALP‐CC in aiding in the diagnosis of primary pulmonary epithelial neoplasia in dogs. Secondary objectives including reporting ALP staining characteristics of canine mesothelium and feline pulmonary lesions.

**Animals:** Hospital population and archived samples.

**Methods:** A retrospective case search was conducted to identify canine and feline cases with contemporaneous cytology and histopathology reports. Neoplastic and non‐neoplastic etiologies were included. Slides were prospectively stained for ALP activity. Sensitivity and specificity were calculated using the histopathologic diagnosis as the gold standard. Mesothelial cells from cavitary lavage were previously collected as part of a different study and ALP activity was evaluated.

**Results:** Fifty canine cases and six feline cases met inclusion criteria. Ninety percent of canine primary pulmonary epithelial neoplasms were ALP‐positive. The sensitivity and specificity for assignment of pulmonary epithelial origin among all samples and among all neoplastic lung lesions were 90% and 80%, and 90% and 78% respectively. Canine mesothelial cells did not display ALP positivity. Feline pulmonary carcinomas displayed ALP positivity.

**Conclusions:** ALP‐CC has good sensitivity and specificity for aiding in the diagnosis of canine primary pulmonary epithelial neoplasia, and mesothelial cells do not display ALP‐positivity.

## 69 Abstract O08

### 69.1 Microthrombi Are Prevalent in Canine Carcinomas and Sarcomas and Positively Associated with Increased D‐Dimer Concentration

#### 69.1.1 **Paolo Pazzi** ^1^; Geoffrey Fosgate^2^, BS, DVM, PhD, DACVPM; Anouska Rixon^3^, BVSc; Josef Hanekom^3^, BVSc, MSc; Annemarie Kristensen^4^, DVM, PhD, DACVIM‐SA, ECVIM‐CA and Oncology; Amelia Goddard^5^, BVSc, BVSc (Hons), MMedVet (CLD), PhD

##### 69.1.1.1 ^1^Faculty of Veterinary Science, University of Pretoria; ^2^Professor, Department of Production Animal Studies, Faculty of Veterinary Science, University of Pretoria; ^3^Dr, Department Companion Animal Clinical Studies, Faculty of Veterinary Science, University of Pretoria; ^4^Professor, Department of Veterinary Clinical Sciences, Faculty of Health and Medical Sciences, University of Copenhagen, Copenhagen, Denmark; ^5^Professor, Department Companion Animal Clinical Studies, Faculty of Veterinary Science, University of Pretoria

**Background:** Prevalence of thrombotic disease in canine carcinoma and sarcoma and its association with clinicopathological parameters has not been investigated.

**Objective:** Estimate the prevalence of thrombotic disease in dogs with carcinomas and sarcomas and estimate the association with clinicopathological parameters.

**Animals:** 30 dogs with carcinoma, 32 with sarcoma (including 11 hemangiosarcomas) and 20 healthy dogs were prospectively enrolled.

**Methods:** All dogs had complete blood count, biochemistry, and hemostasis parameters (thromboelastography, fibrinogen, D‐Dimer concentration, Factor X, VII and antithrombin activity) performed. Dogs with cancer underwent complete post‐mortem and histopathological evaluation for intra‐ and extra‐tumoral micro‐ and macro‐thrombotic disease. Measured parameters were compared between healthy dogs, dogs with cancer and dogs with and without microthrombi.

**Results:** Microthrombi were identified in 31/62 (50%) dogs with cancer; 20/31 (65%) had exclusively intra‐tumoral microthrombi while 5/31 (16%) had exclusively distant microthrombi and 6/31 (19%) had both intra‐tumoral and distant microthrombi. Microthrombi were identified in 18/32 (56%) dogs with a diagnosis of sarcoma and 13/30 (43%) carcinoma. Macrothrombi were identified in three dogs, two with concurrent microthrombi. D‐Dimer concentration was also significantly higher in dogs with microthrombi (2950 ng/ml (interquartile range: 652, 5975)) compared to dogs without microthrombi (581 ng/ml (348, 1107), P<0.001). Hemangiosarcomas had significantly higher D‐Dimer concentrations (6000 ng/mL (3928, 6000)) compared to carcinomas (662 ng/mL (425, 2950)) and healthy dogs (287 ng/mL (200, 424), P<0.001), but not different to other sarcomas (714 ng/mL (473, 3930)).

**Conclusion:** Intra‐ and extra‐tumoral microthrombi are prevalent in dogs with carcinomas and sarcomas and positively associated with increased D‐Dimer concentration.

## 70 Abstract O09

### 70.1 Allogeneic NK Cells and Palliative Radiotherapy in the Treatment of Canine Cancer

#### 70.1.1 **Aryana Razmara** ^1^; Sylvia Cruz^2^; Alicia Gingrich^2^; Sean Judge^2^; Lauren Farley^2^; Robert Rebhun^3^; Michael Kent^3^; Robert Canter^2^

##### 70.1.1.1 ^1^University of California‐Davis, Davis, CA, USA; ^2^Department of Surgery, School of Medicine, University of California‐Davis, Davis, CA, USA; ^3^Department of Surgical and Radiological Sciences, School of Veterinary Medicine, University of California‐Davis, Davis, CA, USA

**Background:** Natural killer (NK) cells are a promising cellular therapy to treat cancer partly because allogeneic sources can be used for off‐the‐shelf treatment without the risk of graft‐versus‐host disease. We evaluated the combination of palliative radiotherapy (RT) and allogeneic NK cell transfer in a first‐in‐dog feasibility trial to speed translation of novel NK modalities in both dogs and people.

**Hypothesis/Objectives:** Our objective was to establish feasibility and describe preliminary data for safety and outcomes in dogs with unresectable oral melanoma treated with allogeneic NK cells following palliative RT.

**Animals:** Five dogs with naturally occurring melanoma were enrolled in this IACUC‐approved pilot trial. Allogeneic NK cells were expanded from blood obtained from five healthy beagles.

**Methods:** Patients underwent weekly RT×4 followed by infusion of intravenous allogeneic NK cells (7.5×10^6 cells/kg). Peripheral blood was obtained for biochemical and immune monitoring at baseline and day one, seven, and fourteen days post‐treatment.

**Results:** Adverse events related to NK infusion were classified as grade 1 or 2 and included emesis, fever, lymphopenia, metabolic acidosis, and hypoglycemia. Lymphocyte counts decreased one day post NK infusion but increased and peaked on day seven post‐infusion. Median survival time was 145 days with maximum and minimum survival times of 445 days and 48 days, respectively.

**Conclusions and Clinical Importance:** Allogeneic NK cell infusions appear well tolerated when administered to dogs with melanoma. This proof‐of‐concept trial provides preliminary data validating the canine model for investigating allogeneic adoptive NK cell transfer alone or in combination with other immunotherapies.

## 71 Abstract O10

### 71.1 Clinico‐Genomic Data of Dogs with Malignant Oral Melanoma

#### 71.1.1 **Lucas Rodrigues**; Dorothy Girimonte, DVM, DACVIM; Garrett Harvey, VMD; Lindsay Lambert; Aubrey Miller; Abigail Hull; Christina Lopes; Gerry Post, DVM, MEM, DACVIM

##### 71.1.1.1 FidoCure/One Health Company

Spontaneous tumors in dogs are highly similar to human cancers histologically, genetically, molecularly and clinically, but the similarities across species correlating biomarkers and outcomes are under investigation. In this study, we identified interesting findings validating concordance across the cancer journeys. This is important to deepen the field of comparative oncology and affirm spontaneous cancer in dogs as an ideal ‘model’ for precision oncology, building upon recent work in tumor biology concordance. Using the Next‐Generation Sequencing (NGS) panel from FidoCure® Precision Medicine Platform associated with real‐world clinical information and treatment outcomes from dogs, we systematically evaluated the prognostic effect of genomic alterations of 1108 dogs from 2702 dogs enrolled in the platform. Our analysis identified mutations in TP53 and PIK3CA as prognostic markers for poor survival in dogs with an overall survival hazard ratio (OS HR) of 1.56 and 1.34, respectively (P<0.01, P=0.03). On the other hand, KIT mutations were associated with good prognosis (OS HR 0.44, P=0.02). These results are aligned with human findings where TP53 is associated with poor prognosis across several types of cancer. Among tumor types, hemangiosarcoma, histiocytic sarcoma and lymphoma showed the worst survival outcomes, OS HR=2.06 (P<0.01), HR=1.54 (P=0.01) and HR=1.47 (P=0.01), respectively. Interestingly, we also identified that tumors carrying somatic BRAF mutant tumors had a better prognosis when treated with lapatinib (OS HR 0.10, P=0.02), ATM with vorinostat (OS HR 0.01 P=0.03), and FLT3 with trametinib (OS HR 0.03, P=0.02). To the best of our knowledge, this is the largest canine tumor clinical genomic dataset and analysis. Applying real‐world evidence and data tools in the FidoCure® dataset, we successfully identified associations between gene mutations and survival and response to target therapy treatment. This study highlights how one health approach can improve not just R&D by using real‐time data from pet dogs being treated for cancer but also improving canine oncology outcomes through precision medicine and target therapy.

## 72 Abstract O11

### 72.1 Collaborative Care Improves Client Perceptions When Managing Pets with Cancer

#### 72.1.1 **Kai‐Biu Shiu** ^1^; Elizabeth Maxwell^2^, DVM, MS, DACVS‐SA, CVPP, ACVS Fellow of Surgical Oncology; Kimberly Ness^3^; Thomas Minsel^4^, PhD; Samantha Morello^5^, DVM, DACVS; Bob Murtaugh^6^, DVM, MS, DACVIM, DACVECC, FCCM

##### 72.1.1.1 ^1^VCA Pet CancerCare Alliance; ^2^Treasurer, Collaborative Care Coalition; ^3^Trone Research + Consulting; ^4^Head of Research and Data Science, Trone Research + Consulting; ^5^Center for Veterinary Business and Entrepreneurship, Cornell University; ^6^Director, Collaborative Care Coalition

**Background:** Collaboration between primary‐care veterinarians (pcVets) and veterinary oncologists is common for pets diagnosed with cancer, but no data exists that explores client perceptions of collaborative care.

**Hypothesis/Objectives:** The objectives of the study were to 1) describe client perceptions of the value of collaborative veterinary care when treating a pet for cancer, and 2) identify predictors of client satisfaction related to collaborative care between the pcVet and a veterinary oncologist.

**Sample:** The sample consisted of 890 U.S. dog owners who had pets diagnosed with cancer in the past three years.

**Methods:** Research was conducted using an online contextual survey. Scores related to client advocacy were derived from the net promoter scale, and were analyzed using predictive modeling.

**Results:** Seventy percent of clients across all income brackets rated seeing a specialist as a high value, based on money spent and patient outcome. Client perceptions of pcVets were six times more likely to improve with a referral than worsen. Delayed referral resulted in lower client satisfaction scores for pcVets. Top predictors of client satisfaction with pcVets were: being responsive to questions, staying involved with their dog's care, and willing to work with other veterinarians and specialists. For specialists, top predictors were: providing accurate cost estimates, cancer knowledge, and effectiveness in treatment and care. All were significant predictors of client advocacy (p<0.0001).

**Conclusions and Clinical Importance:** Clients view early referral and collaboration between pcVets and specialists positively, fostering client satisfaction and a positive value‐based care model for dogs diagnosed with cancer.

## 73 Abstract O12

### 73.1 Annotation of Canine Cancer Genomic Biomarkers Through Comparative Analysis of Human Mutations in COSMIC

#### 73.1.1 **Guannan Wang**; Sharda Sakthikumar; Salvatore Facista; Manisha Warrier; Derick Whitley; Zhan Yang Zhu; Will Hendricks

##### 73.1.1.1 Vidium Animal Health

Genomic diagnostics are routinely used in human cancer medicine to guide diagnosis, treatment and prognostication. Growing evidence supports that human and canine cancers share genomic and clinical similarities. However, mutation‐based biomarkers from canine data are less well‐established than those utilized in human precision medicine.

Our objective was to bridge the canine precision medicine knowledge gap via “caninization” of the largest database of human cancer mutations, the Catalogue of Somatic Mutations in Cancer (COSMIC), and consume these data into a canine cancer knowledgebase, Vidium Insight.

To identify canine equivalents of human COSMIC mutations, cross‐species conversion was performed via both genomic liftover and protein alignment, considering evolution conservation score and sequence homology. Known/predicted pathogenic mutations in COSMIC Cancer Gene Census (CGC) genes were selected for “caninization” as a proof‐of‐concept.

Of 1763970 total mutation records in 717 CGC genes in COSMIC (v95), 295439 unique, pathogenic mutations in 707 CGC genes across ~200 tumor types were initially converted. Histologically similar canine cancers exist for most primary human cancer types, thus these inferences can be informative for dogs. Of 707 human CGC genes, 95% have canine orthologs and are highly conserved among species. Canine equivalents were identified for ~80% of human mutations.

Most of the nearly 3 million documented pathogenic human cancer mutations have canine counterparts. Thus, systematic “caninization” of these human cancer mutations with established biomarker associations will not only expand genomic knowledge of canine cancers in research settings, but will also improve utilities of genomic diagnostic tools in canine cancer patient care.

## 74 Abstract O13

### 74.1 Investigation of Urinary Extracellular Vesicle‐Associated MicroRNAs in Dogs with Urothelial Carcinoma

#### 74.1.1 **Tim L. Williams**; Jenni Karttunen; Andrew Grant; Lajos Kalmar; Sarah Stewart; Fiona Karet Frankl

##### 74.1.1.1 University of Cambridge

**Background:** Urinary extracellular vesicles (UEVs) are membrane bound particles that contain microRNAs (miRNAs) from cells in the urinary tract. Evaluation of UEV‐associated miRNAs in canine urothelial carcinoma (UC) could provide novel biomarkers for UC and indicate pathways involved in tumorigenesis.

**Objectives:** Compare UEV‐associated miRNAs of dogs with UC with those of healthy dogs and dogs with urinary tract infection (UTI—primary differential diagnosis for UC).

**Animals:** Discovery cohort: 12 UC dogs, 6 healthy dogs and 5 UTI dogs. Validation cohort: 8 UC dogs, 5 healthy dogs and 8 UTI dogs.

**Methods:** UEVs were isolated from 1–5 mL of urine by ultrafiltration combined with size exclusion chromatography, prior to RNA extraction. Small RNAs were identified using next generation sequencing in the discovery cohort. Differential expression (DE) analysis of small RNA sequencing data identified candidate miRNAs, the expression of which was evaluated in both discovery and validation cohorts using digital droplet PCR (ddPCR).

**Results:** DE analysis identified 11 miRNAs that were upregulated in UEVs from UC dogs (vs. healthy), six of which (miR‐143, miR‐145, miR‐150, miR‐199, miR‐451, miR‐486) were selected for further evaluation. Increased expression of most candidate miRNAs (after normalisation to urine creatinine concentration) in UEVs of UC dogs (vs. UTI and healthy dogs) was confirmed by ddPCR in the discovery cohort, but these candidate miRNAs were not significantly over‐expressed in the validation cohort.

**Conclusions and Clinical Importance:** Differentially expressed miRNAs associated with UEVs may provide insights into the pathogenesis of canine UC, however their utility as biomarkers requires further investigation.

## 75 Abstract O14

### 75.1 Do Dogs Get Cancer‐Associated Thrombosis? Retrospective Analysis of Characteristics of Dogs with Thrombosis: 2014–2019

#### 75.1.1 **Anna Winner** ^1^; Sarah Shropshire^2^, DVM, DACVIM, PhD; Susan Lana^3^, DVM, DACVIM (Oncology); Tracy Webb^4^, DVM, DACVECC, PhD

##### 75.1.1.1 ^1^College of Veterinary Medicine and Biomedical Sciences, Colorado State University; ^2^Small Animal Internal Medicine, Colorado State University; ^3^Oncology, Colorado State University; ^4^Urgent Care Faculty, Emergency and Critical Care, Colorado State University

**Background:** The association between thromboembolic events and cancer in humans has been described over the course of almost two centuries, with the first observation taking place in the mid 1800’s. Little research exists in veterinary medicine on cancer‐associated thrombosis.

**Objective:** Describe the prevalence of neoplasia in dogs suffering from thromboembolic events and determine associated factors.

**Animals:** 154 client‐owned dogs where a thrombus or thromboembolic event was documented in the medical record.

**Methods:** Medical records were reviewed from 2014–2019. Cases were excluded if thrombi were not definitively identified or if the medical record was incomplete such that critical data was not available. Cases were examined for the presence of neoplasia, presence of metastasis, concurrent morbidities, exogenous steroid administration, recent surgery, and clinicopathological parameters including hematocrit, platelet count, nucleated cell count, and albumin concentration.

**Results:** Fifty‐five breeds were represented with Labrador Retrievers making up the majority (23/154, 14.9%). Of the 154 cases, 78 had confirmed neoplasia (50.6%) and 76 did not have evidence of neoplasia (49.4%). Of the 78 cases with neoplasia, 38 had metastatic disease (48.7%) and 40 did not have definitive evidence of metastasis (51.3%). The majority of cases were not receiving steroids (84.6%) and did not have recent surgery (88.4%).

**Conclusions and Clinical Importance:** Preliminary data from this retrospective study reveal that half of dogs with thrombosis had concurrent neoplasia and support the need for further investigation into predictive factors and preventative therapies in this patient population.

## 76 Abstract O15

### 76.1 Anti‐Cancer Effects of Oral Paclitaxel Against Canine Mammary Gland Cancer

#### 76.1.1 **Hyung‐Kyu Chae** ^1^; Hwa‐Young Youn^2^, DVM, PhD; Ga‐Hyun Lim^3^

##### 76.1.1.1 ^1^College of Veterinary Medicine, Seoul National University; ^2^Professor, Laboratory of Veterinary Internal Medicine, Seoul National University; ^3^Administrative Assistant, College of Veterinary Medicine, Seoul National University

**Background:** Canine mammary gland cancer (CMGC) is one of the most common neoplasms in intact female dogs. However, adjuvant chemotherapy has not been demonstrated to have a benefit in dogs with CMGC.

**Objectives:** The objective of this study was to investigate the anti‐cancer effects of recently developed oral paclitaxel against canine mammary gland cancer *in vitro* and *in vivo*.

**Animals:** Twenty‐four female athymic nude mice xenografted with canine mammary gland carcinoma were used in this study.

**Methods:** This was a prospectively designed experimental study. Cell cycle arrest, apoptosis and angiogenesis of mouse xenograft tumor tissues were investigated by flow cytometry, western blot analysis, TUNEL assay and immunostaining using anti‐cluster of differentiation‐31 antibody.

**Results:** Oral paclitaxel inhibited both cell viability and induced G2/M phase cell arrest and apoptosis in a concentration‐proportional manner (p<0.01). In animal experiment, the average tumor volume was significantly decreased in proportion to the administered oral paclitaxel dose (p<0.01). Histologically, oral paclitaxel induced apoptosis and showed anti‐angiogenic effect in tumor tissues (p<0.01). It was also downregulated the expression of cyclin‐D1 in tumor tissues (p<0.01).

**Conclusions and Clinical Importance:** Our results suggest that oral paclitaxel may have anti‐cancer effects on CMGC through cell cycle arrest and induction of apoptosis. This study will provide a novel approach to the treatment of dogs with CMGC.

**Figure:**
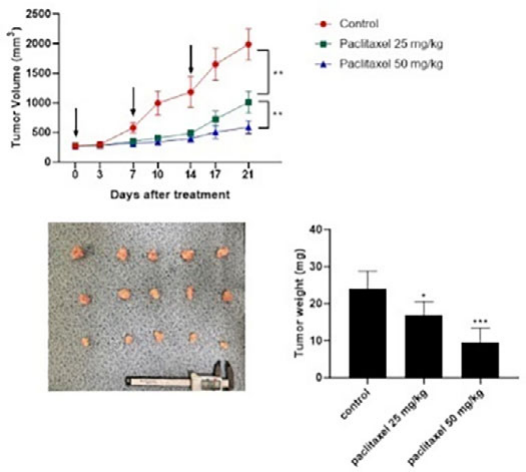
Image 1

**Figure:**
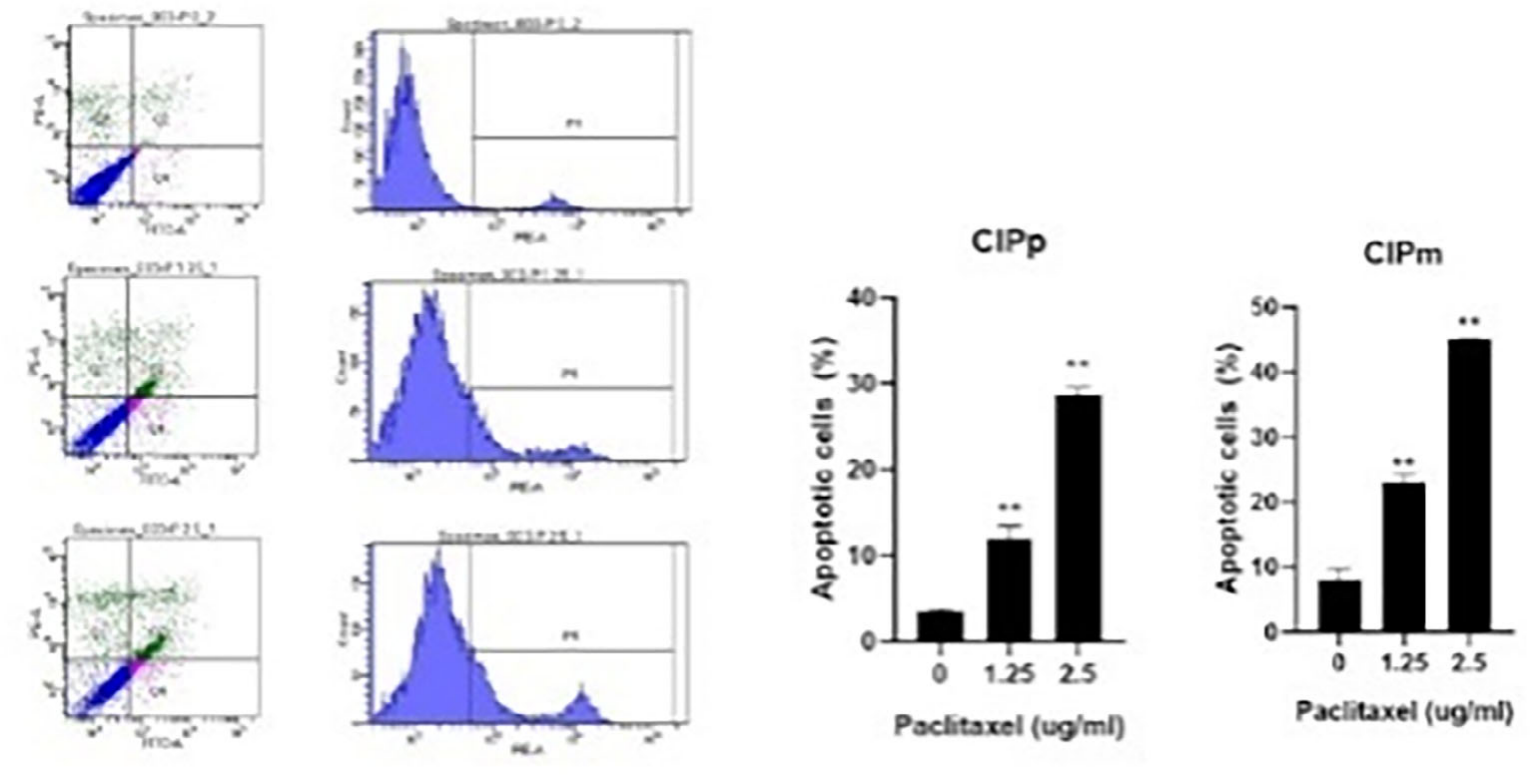
Image 2

## 77 Abstract O16

### 77.1 Anticancer Activity of IRAK‐4 Inhibitors Against Canine Lymphoid Malignancies

#### 77.1.1 **Jeong‐Hwa Lee** ^1^; Jun‐Hyeong Park^1^, DVM; Ju‐Hyun An^1^; Chong‐Woo Park^2^; Yoon‐Pyo Choi^2^; Seong‐Wook Seo^2^; Hyuk‐Woo Lee^2^; Hwa‐Young Youn^1^

##### 77.1.1.1 ^1^Seoul National University; ^2^Future Medicine Co.

**Background:** The interleukin‐1 receptor‐related kinase 4 (IRAK4), downstream of Myd88, plays an essential role in hyperactive TLR signaling seen in some B‐cell lymphomas. In particular, efficient IRAK4 inhibitors of activated B‐cell subtype of human diffuse large B‐Cell lymphoma (DLBCL) are being developed. However, the anticancer effect of IRAK‐4 inhibitors in veterinary medicine has not been elucidated. It is, therefore, explored in this study involving the GL‐1 and CL‐1 canine lymphoma cell lines *in vitro*.

**Hypothesis/Objectives:** Treatment with IRAK4 inhibitor in canine lymphoid cell lines will decrease viability and increase apoptosis according to drug concentration.

**Animals:** Healthy Beagle dogs were included as donors for canine peripheral blood mononuclear cells (cPBMCs).

**Methods:** Pre‐clinical study. MyD88 expression was analyzed using polymerase chain reaction. GL‐1 and CL‐1 cells were subjected to concentration‐ and time‐dependent treatment with an IRAK‐4 inhibitor and assessed for viability, TLR signaling association, and apoptosis using a cell counting Kit‐8 assay, Western blotting, and flow cytometry.

**Results:** The GL‐1 and CL‐1 cells exhibited enhanced MyD88 expression, however, cPBMCs did not. The IRAK‐4 inhibitor reduced cell viability in a dose‐ and time‐dependent manner, significantly reduced the phosphorylation of molecules associated with TLR signaling at IC_50_ such as IRAK1, IRAK4, NF‐κB and STAT3, and induced apoptosis in GL‐1 and CL‐1 cells.

**Conclusions and Clinical Importance:** The anticancer effect of the IRAK‐4 inhibitor on canine lymphoma cells is mediated by apoptosis via downregulation of TLR signaling. The potential of IRAK4 inhibitor as a treatment option for canine lymphoma is suggested.

**Figure:**
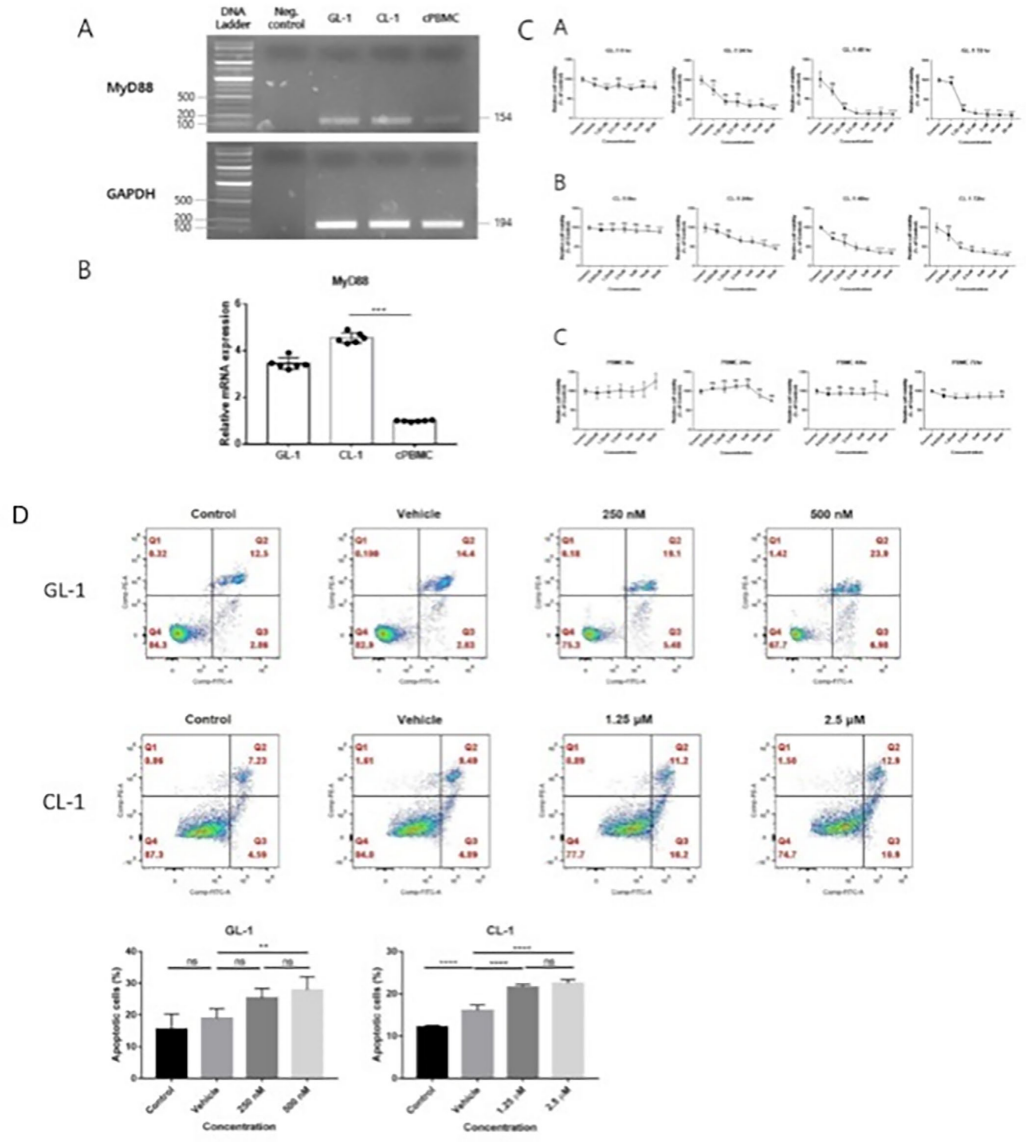
Figure 1

**Figure:**
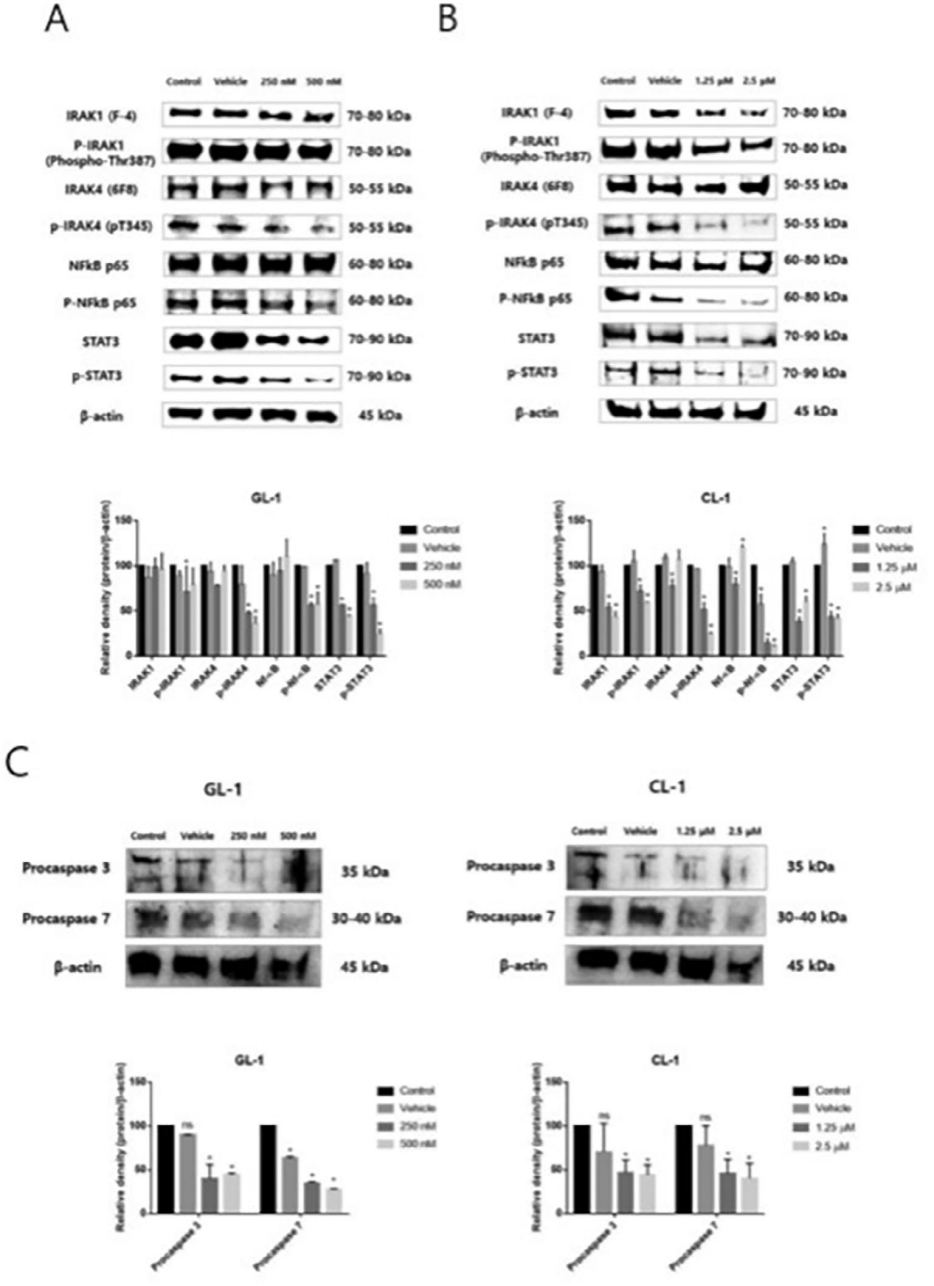
Figure 2

## 78 Abstract O17

### 78.1 Evaluation of a Novel Blood Test for the Detection of “Difficult to Diagnose” Cancers

#### 78.1.1 Katherine M. Lytle; **Andi Flory**, DVM, DACVIM (Oncology); Kristina Kruglyak, PhD; John Tynan, PhD; Lisa McLennan; Jill Rafalko, MS, CGC; Daniel Grosu, MD, MBA; Lauren Holtvoigt, DVM, MBA; Angela McCleary‐Wheeler, DVM, PhD, DACVIM (Oncology); Jason Chibuk, MS, CGC; Susan Cho Hicks, MAS; Ilya Chorny, PhD; Dana W.Y. Tsui, PhD

##### 78.1.1.1 PetDx

**Background:** Certain canine cancers occur in anatomical locations that are difficult or impossible to access by fine needle aspiration or surgical biopsy for confirmation of malignancy. Alternative means of assessing these “difficult to diagnose” (DTD) cases may offer utility to clinicians, patients, and their families.

**Hypothesis/Objectives:** To evaluate performance of a next‐generation sequencing‐based liquid biopsy test for the detection of genomic alterations associated with cancer in a population of dogs with a “difficult to diagnose” cancer.

**Animals:** Client‐owned dogs enrolled in a clinical validation study for a novel blood‐based liquid biopsy test.

**Methods:** Blood samples from an all‐comers cohort of 351 dogs with a variety of cancer diagnoses were subjected to DNA extraction, proprietary library preparation, and next‐generation sequencing. Sequencing data were analyzed using an internally developed bioinformatics pipeline to detect genomic alterations associated with the presence of cancer.

**Results:** Of 351 cancer‐diagnosed dogs, 33% (n=115) had malignant masses in locations designated as DTD, including bone and joint, central nervous system, head and neck, intra‐abdominal, and intra‐thoracic. The median age of DTD subjects was 10 years; median weight was 31.1 kg; 57% of subjects were male and 43% female; there was an equal distribution of purebred to mixed‐breed subjects. A **Cancer Signal Detection** (positive) result was issued in 54 cases, for an overall detection rate of 47% in the “DTD” cohort.

**Conclusions and Clinical Importance:** Liquid biopsy offers clinicians and families a new, noninvasive option for cancer evaluation in cases where traditional diagnostic approaches may be challenging or impossible.

## 79 Abstract O18

### 79.1 Identification of the Potential Candidate Genes and Signaling Pathways Involved in Lymphoma Progression in Dogs

#### 79.1.1 **Ye‐In Oh** ^1^; Sunirmal Sheet^2^, PhD; Bong‐Hwan Choi^3^, PhD; Kyung‐Tai Lee^3^; Kyoung‐Won Seo^3^, DVM, PhD, DAiCVIM

##### 79.1.1.1 ^1^Seoul National University; ^2^National Institute of Animal Science; ^3^Associated Professor, Internal Medicine, Seoul Nation University

**Background:** The role of several genes is not well defined in the pathway of canine high‐grade multicentric lymphoma progression but may provide important information regarding the development of canine lymphoma.

**Hypothesis/Objectives:** The goal of this study is to explore the important genes and pathways involved in the progression of canine high‐grade multicentric lymphoma and to understand the underlying molecular mechanisms by using RNA sequencing.

**Animals:** Fourteen dogs (7 dogs with high‐grade multicentric lymphoma and 7 dogs without lymphoma) were included.

**Methods:** RNAs acquired from seven pairs of lymphoma patients and non‐lymphoma blood samples from different breeds of dogs were sequenced. Sequencing reads were preprocessed, aligned with the reference genome, and assembled. The expressions were estimated through bioinformatics approaches.

**Results:** At the false discovery rate (FDR) <0.05 and fold change (FC) ≥1.5, a total of 625 differentially expressed genes (DEGs) were identified between lymphoma and non‐lymphoma samples, including 347 up‐regulated DEGs such as *SLC38A11*, *SCN3A*, *ZIC5,* etc. and 278 down‐regulated DEGs such as *LOC475937, CSMD1, KRT14,* etc. Especially, the pathway of PI3K‐Akt signaling, which was directly related to the lymphoma, was enriched in KEGG analysis. In the protein‐protein interaction network, *CDK1* was found to be the top hub gene with the highest degree of connectivity in lymphoma samples.

**Conclusions and Clinical Importance:** These findings were highlighted as a new candidate marker for the development of canine high‐grade multicentric lymphomas and facilitated the understanding of the molecular mechanisms of lymphoma progression in dogs.

## 80 Abstract O19

### 80.1 Pilot Study of Partial Ablation with Mechanical High‐Intensity Focused Ultrasound (Histotripsy) in Dogs with Spontaneously Occurring Soft Tissue Sarcomas (VCS Award Winner)

#### 80.1.1 **Ester Yang** ^1^; Lauren Arnold^2^; Sheryl Coutermarsh‐Ott^1^; Nick Dervisis^1^; Eli Vlaisavljevich^2^; Shawna Klahn^1^

##### 80.1.1.1 ^1^Virginia‐Maryland College of Veterinary Medicine; ^2^Virginia Polytechnic Institute and State University of Biomedical Engineering and Mechanics

**Introduction:** Histotripsy is a non‐thermal and non‐invasive high‐intensity focused ultrasound ablative technique that causes mechanical fragmentation of tissue resulting in liquefied cellular debris with histologically clear demarcated boundaries between treated and non‐treated tissue. Histotripsy has never been evaluated in a spontaneous cancer model. Our objective was to evaluate the safety and feasibility of histotripsy to achieve tumour ablation in dogs with soft tissue sarcoma (STS).

**Methods:** Dogs diagnosed with STS were recruited. CT of the chest, abdomen, and the tumour was performed for staging and treatment‐planning, and pre‐treatment biopsy was obtained. Safety was monitored with exams, owner reports and CBC/serum biochemistry. Partial tumour ablation was performed using a prototype Histotripsy system. Anatomical ablation characteristics were evaluated with contrast CT at 1‐ and 4‐days post‐treatment, with tumour resection 4‐days post‐treatment. Tumour ablation effectiveness was evaluated with H&E.

**Results:** Ten dogs were recruited and treated. Tumour histologies included three grade III STS, 4 grade II STS, 2 grade I STS and 1 malignant mesenchymoma. Currently, seven dogs are alive, two dogs were euthanized from recurrence or suspected metastasis and one dog was lost to follow‐up. There were no changes in bloodwork values. The mean planned ablated volume was 8.41±5.01 cm3. The mean duration of treatment was 30.43±12.67 min. Histotripsy‐related complications were generally self‐limiting and included various degrees of cutaneous injury. Post‐treatment histopathology indicated complete ablation of targeted tumour with no intact cells identified.

**Conclusion:** Histotripsy can achieve safe, rapid, and effective tumour ablation in dogs diagnosed with STS.

## 81 SMALL ANIMAL INTERNAL MEDICINE – ENDOCRINOLOGY

## 82 Abstract EN01

### 82.1 Characterization of the Renin‐Angiotensin‐Aldosterone System in Telmisartan‐ or Enalapril‐Treated Dogs with Proteinuric Chronic Kidney Disease

#### 82.1.1 **Joanna E. Murdoch** ^1^; Bianca Lourenco^2^, DVM, PhD, DACVIM (SAIM); Roy Berghaus^3^, DVM, MS, PhD, DACVPM (Epidemiology); Amanda Coleman^2^, DVM, DACVIM (Cardiology)

##### 82.1.1.1 ^1^University of Georgia; ^2^Department of Small Animal and Surgery, College of Veterinary Medicine, University of Georgia; ^3^Department of Population Health, College of Veterinary Medicine, University of Georgia

**Background:** The relative effects of angiotensin‐converting enzyme inhibitors (ACEi) and angiotensin receptor blockers (ARB) on the circulating renin‐angiotensin‐aldosterone system (RAAS) in dogs with proteinuric chronic kidney disease (CKD) are undescribed.

**Objective:** To characterize the RAAS in dogs with CKD treated with an ACEi (enalapril) or ARB (telmisartan).

**Samples:** Serum and urine from dogs with naturally occurring proteinuric CKD (n=36).

**Methods:** Serum concentrations of angiotensin (Ang) peptides (Ang I, II, III, IV, 1‐5, and 1‐7) and aldosterone, and urinary aldosterone‐to‐creatinine ratio (UACR) were determined using samples collected prior to and after 30 days of treatment with enalapril (n=17) or telmisartan (n=19). Liquid chromatography and mass spectrometry were used to determine serum concentrations of Ang peptides and aldosterone. Data were analyzed using linear mixed models controlling for concurrent treatment with amlodipine, presence of heart disease, and age.

**Results:** Relative to pre‐treatment values, serum concentrations of Ang II were significantly increased and decreased in telmisartan‐ and enalapril‐treated dogs, respectively (both P<0.001). Mean [95% CI] percentage change from pre‐treatment value in serum Ang1‐7 concentration was significantly greater in telmisartan‐ (753% [489% to 1134%]) versus enalapril‐treated (149% [69% to 268%]) dogs (P<0.001). Differences in percentage change in serum aldosterone or UACR, and significant effects of concurrent amlodipine treatment, presence of heart disease, or age, were not observed.

**Conclusions and Clinical Importance:** Compared to enalapril, treatment with telmisartan was associated with significantly greater increases in the beneficial Ang peptide Ang1‐7, representing a potential treatment advantage of the latter in dogs with CKD.

## 83 Abstract EN02

### 83.1 Plasma Glucagon‐Like Peptide‐1 Concentrations in Dogs with Aminoaciduric Canine Hypoaminoacidemic Hepatopathy Syndrome: A Pilot Study

#### 83.1.1 **Sarah M. Holm** ^1^; Seth Peng^1^; Marlena Holter^1^; Bethany Cummings^2^; John Loftus^3^, PhD, DVM, DACVIM (SAIM)

##### 83.1.1.1 ^1^Cornell University; ^2^UC Davis; ^3^Assistant Professor, Clinical Sciences, Cornell University

**Background:** Most dogs with aminoaciduric canine hypoaminoacidemic hepatopathy syndrome (ACHES) have superficial necrolytic dermatitis (SND) lesions (i.e., ACHES‐SND or hepatocutaneous syndrome). In people, SND is most commonly caused by glucagonomas that over‐produce glucagon. Dogs with ACHES are also predisposed to diabetes mellitus (DM). Glucagon‐like peptide‐1 (GLP‐1), derived from proglucagon, is important for glucose regulation, as it potentiates insulin secretion from pancreatic beta‐cells and may be dysregulated in ACHES.

**Hypothesis/Objectives:** To compare plasma concentrations of GLP‐1 between heathy and ACHES dogs, hypothesizing lower concentrations in ACHES.

**Animals:** Healthy dogs (n=5) and dogs with ACHES (n=8; ACHES‐DM n=1).

**Methods:** Preprandial and 15‐minute postprandial plasma total GLP‐1 concentrations were measured with a commercial assay (MesoScale Discovery). We compared groups by two‐way ANOVA with repeated measures or Mann‐Whitney test.

**Results:** Dogs with ACHES had significantly (P=0.02) lower plasma GLP‐1 concentrations than healthy dogs. The mean postprandial change in GLP‐1 was lower, although not significantly, in ACHES dogs (mean 1.85 pg/ml) than healthy dogs (3.5 pg/ml).

**Conclusions and Clinical Importance:** Lower plasma GLP‐1 concentrations suggest a contributing role to the propensity of DM in ACHES dogs. If lower GLP‐1 concentrations in ACHES are primarily due to reduced excretion, this may also be a surrogate marker of reduced GLP‐2 and concomitant reductions in amino acid transport function. The observed trends in GLP‐1 could reflect a unifying mechanism explaining hypoaminoacidemia, aminoaciduria, and predisposition to DM in ACHES, and an additional diagnostic or therapeutic target.

## 84 Abstract EN03

### 84.1 Effect of Ethylenediaminetetraacetic Acid and Magnesium Chloride on Measurement of Canine Adrenocorticotropic Hormone

#### 84.1.1 **Lydia Peña** ^1^; Ellen Behrend^2^; Robert Kemppainen^3^; Megan Grobman^1^; Hollie Lee^3^

##### 84.1.1.1 ^1^Veterinary Teaching Hospital, Auburn University; ^2^Veterinary Information Network; ^3^Auburn University

**Background:** Endogenous ACTH is commonly measured using a validated chemiluminescent assay (Immulite 1000). Underfilling of blood tubes can result in high EDTA concentrations, which may interfere with hormone measurement. Magnesium chloride (MgCl_2_) may reverse the effect.

**Hypothesis/Objectives:** The objectives of the study were 1) to investigate the effect of EDTA on ACTH measurement; 2) to determine if MgCl_2_ addition could overcome the effect of EDTA.

**Animals:** Purchased canine serum was used.

**Methods:** EDTA was added to samples to concentrations of 4.1 mmol/L (simulating a completely filled collection tube), 8.2 mmol/L (50% filled), and 16.4 mmol/L (25% filled). Human ACTH was added to the desired concentration creating low and high concentration pools. The effect of 5 mmol/L or 6.7 mmol/L MgCl_2_ in the presence of each EDTA concentration was evaluated. For each condition, 8 samples were created, and all samples were assayed in duplicate.

**Results:** A one‐way repeated measures ANOVA was performed to compare apparent concentrations between conditions with post hoc analysis via the Holm‐Sidak method. Significance was set at p<0.05. Addition of 8.2 mmol/L or 16.4 mmol/L EDTA significantly lowered apparent ACTH concentration compared to 4.1 mmol/L EDTA in both low and high ACTH pools. Addition of 5 mmol/L or 6.7 mmol/L MgCl_2_ overcame the effect of EDTA.

**Conclusions and Clinical Importance:** Elevated EDTA concentrations in plasma that could result from underfilling of tubes causes a significant decrease in apparent ACTH concentration. This effect can be overcome with the addition of 5 or 6.7 mmol/L MgCl_2_.

## 85 Abstract EN04

### 85.1 Urinary Iodine Clearance Following Iodinated Contrast Administration in Normal Cats; Implications for ^131^I Use

#### 85.1.1 **Gabriella M. Allegrini** ^1^; Valerie Poirier^2^, DACVIM (Oncology), DACVR‐RO; Julia Pezzali^3^; Hughes Beaufrère^4^; Anna Shoveller^3^; Matthew Kopke^5^; Janet Beeler‐Marfisi^6^, DACVP

##### 85.1.1.1 ^1^Ontario Veterinary College; ^2^Animal Cancer Center, Ontario Veterinary College, University of Guelph; ^3^Department of Animal Biosciences, Ontario Agricultural College, University of Guelph; ^4^Department of Veterinary Medicine and Epidemiology, University of California, Davis; ^5^Veterinary Nutrition Group; ^6^Department of Pathobiology, Ontario Veterinary College, University of Guelph

**Background:** Exogenous iodine sources interfere with uptake of radioactive iodine (^131^I) by the thyroid gland. This has implications for the treatment of cats with hyperthyroidism that have recently undergone computed tomography (CT) with intravenous (IV) iodinated contrast medium (ICM). In human medicine, a 4‐week delay is required between CT and ^131^I.

**Hypothesis/Objectives:** It will require 4 weeks for urinary iodine concentration (UIC) to decrease to baseline after IV ICM administration.

**Animals:** 10 healthy adult neutered male cats living in a group colony.

**Methods:** Cats were sedated and received IV ICM (600 mg iodine/kg). On samples collected prior to injection (n=3) and on days 1, 2, 3, 7, 10 and weeks 2–6 post injection, UIC and urinary creatinine concentration (UCC) were measured, and the urinary iodine creatinine ratio (ICR) was calculated. Urinary outcome variables were modelled using a linear mixed effects model with time as a fixed effect and individual cats as a random effect.

**Results:** UIC increased 37‐ to 884‐fold on day 1 post ICM injection and returned to baseline during week 2 of the study. Compared to baseline, UIC was significantly increased for days 1–7, (all p<0.001); UCC was significantly lower for days 1–10 (all p<0.029); and ICR was significantly increased from day 1–10 (all p<0.001, except day 10 p=0.049).

**Conclusions:** Urinary clearance of iodine after IV ICM administration requires between 7–14 days to return to baseline in healthy cats. This suggests that a 2‐week delay between the iodinated contrast study and ^131^I treatment is appropriate.

## 86 Abstract EN05

### 86.1 Assessment of the FreeStyle Libre 2 Interstitial Glucose Monitor in Hypo and Euglycemia in Cats

#### 86.1.1 **Alisa S. Berg** ^1^; Chiquitha Crews^2^; Adriana Alfonso‐Castro^1^; Susan Hill^1^; Chen Gilor^1^, DVM, DACVIM (SAIM), PhD

##### 86.1.1.1 ^1^University of Florida Small Animal Hospital; ^2^University of Florida

**Background:** Continuous glucose monitoring systems that measure interstitial glucose (IG) have been validated previously for use in cats. In people, the FreeStyle Libre 2 (FSL2) is considered sufficiently accurate in the hypoglycemic range to make critical treatment decisions without the need to confirm its results with a blood glucose (BG) measurement. There are currently no studies on the accuracy of FSL2 in the hypoglycemic range in cats.

**Objectives:** To determine FSL2 accuracy during hypoglycemia in cats.

**Animals:** Six healthy, purpose‐bred cats.

**Methods:** Hyperinsulinemic‐hypoglycemic clamps were performed by concurrent IV infusion of regular insulin (constant rate) and dextrose (variable rate). To account for blood‐interstitium lag time, paired BG (AlphaTrak®) and FSL2 readings were considered only when BG was stable (change over 10 min ≤0.5 mg/dL/min). Pearson's r and Bland‐Altman tests were used for correlation and bias, respectively (p<0.05 considered significant).

**Results:** Overall, BG and IG correlated strongly (r=0.85, p<0.001). IG underestimated BG by 19.3±11.4, 9.9±7.4, and 2.5±5.6 mg/dL in the 80–120 (n=47), 60–79 (n=16), and 50–59 mg/dL (n=20) ranges respectively. IG overestimated BG by 6.0±6.8 mg/dL in the 38–49 mg/dL range (n=17).

**Conclusions:** Clinicians should be cautioned that while IG underestimates BG throughout most of the eu‐hypoglycemic range, IG generally overestimates BG in severe hypoglycemia (<50 mg/dL). It is therefore recommended to measure BG in cats with low IG readings before critical treatment decisions are made.

**Figure:**
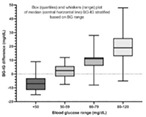
Image 1

## 87 Abstract EN06

### 87.1 Sterility of Refrigerated, Multidose Insulin Vials Through Six Months of Use

#### 87.1.1 **Alisa S. Berg** ^1^; Megan Jacob^2^, MS, PhD; Lisa Mamo^3^; Doug Pluta^4^; Katharine Lunn^5^, BVMS, MS, PhD, DACVIM

##### 87.1.1.1 ^1^Small Animal Hospital, University of Florida; ^2^Associate Professor, Director of Diagnostic Laboratories, North Carolina State University; ^3^Research Specialist, North Carolina State University; ^4^Microbiologist, North Carolina State University; ^5^Associate Professor, Small Animal Internal Medicine, North Carolina State University

**Background:** Most small animal diabetic patients are considered insulin‐dependent. Treatment can be challenging for cost‐conscious owners, particularly when insulins are discarded after 28–60 days of use, according to manufacturer recommendations. Veterinarians commonly recommend off‐label use of a single insulin vial for up to 6 months; however, limited data exist to support the safety of this practice.

**Objectives:** To evaluate sterility in refrigerated, multidose insulin vials through 6 months of routine aspiration.

**Animals:** None

**Methods:** Six vials each of Lantus (glargine, 10‐mL multidose vial, Sanofi, Bridgewater, containing metacresol preservative) and ProZinc (PZI, 10‐mL multidose vial, Boehringer Ingelheim, containing phenol preservative) insulin were refrigerated and aspirated twice daily for six months, using a new insulin syringe each time. Three of each insulin type were prepared with a single‐use alcohol swab prior to sampling. Three times weekly, aspirated samples were inoculated in Tryptic Soy Broth enrichment media (Fisher Scientific). Samples with evidence of microbial growth were cultured and speciated. Endpoints were microbial vial contamination (defined as three consecutive positive cultures of the same organism) and completion of the six‐month study period.

**Results:** Microbial contamination was not identified in any vial throughout the study period. A total of 454 aspirated samples were cultured, one of which exhibited non‐repeatable positive growth of *Staphylococcus epidermidis*. This vial was prematurely lost to breakage after 59 culture samples (29 after the positive growth).

**Conclusions:** Refrigerated phenol‐ and metacresol‐containing multidose insulin products carry minimal risk for iatrogenic infection through 6 months of use, regardless of alcohol swab preparation.

## 88 Abstract EN07

### 88.1 Validation of the Thyrotropin‐Releasing Hormone Simulation Test in Healthy Cats

#### 88.1.1 **Petra Cerna** ^1^; Mark Peterson^2^; Markos Antonakakis^1^; Jade Peralta^1^; Kristine Kofron^1^; Jennifer Hawley^1^; Michael Lappin^1^

##### 88.1.1.1 ^1^Colorado State University; ^2^Animal Endocrine Clinic

**Background:** A thyrotropin‐releasing hormone (TRH) stimulation test can be used to evaluate the integrity of the pituitary‐thyroid gland axis. Administration of exogenous TRH (0.1 mg/kg) to cats with normal thyroid function will increase thyroid stimulating hormone (TSH) and serum total thyroxine (tT_4_) concentrations. However, adverse side effects (vomiting, panting, hypersalivation) are common with that protocol.

**Hypothesis/Objectives:** To determine the lowest intravenous dose of TRH that adequately increases serum TSH and tT_4_ concentrations in normal cats. Our hypothesis was that TRH administered at doses lower than 0.1 mg/kg will adequately stimulate TSH and tT_4_ production in healthy cats and cause less side effects.

**Animals:** Six adult healthy research cats.

**Methods:** Cats were randomly administered 3 different doses of TRH (0.01, 0.05, or 0.1 mg/kg) intravenously. Serum TSH and tT_4_ concentrations were measured using Immulite® 1000 at time 0, 30, and 60 minutes after TRH administration.

**Results:** All 3 TRH doses induced a significant TSH response (0.01 mg/kg, P=0.0055; 0.05 mg/kg, P=0.0017; 0.1 mg/kg, P=0.0001). There was no statistical difference among the different TRH doses in stimulation of TSH (Figure 1). Only the lowest dose of TRH (0.01 mg/kg) induced an increase in serum T_4_ concentration (P=0.03). Administration of lower TRH doses (0.01 and 0.05 mg/kg) caused less side effects (1/6 cats) than did the highest concentration (0.1 mg/kg; 3/6 cats).

**Figure:**
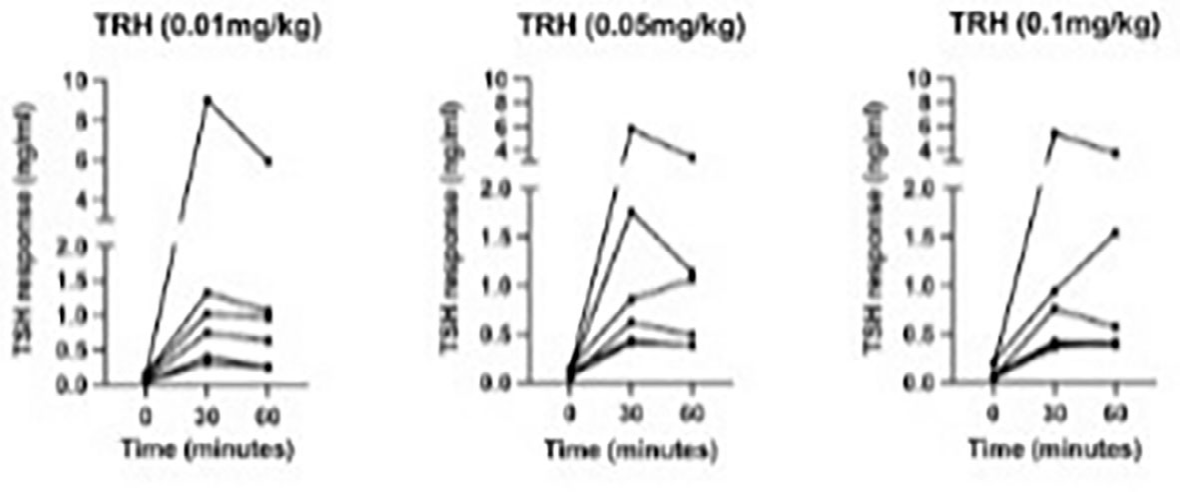
Figure 1

**Conclusions and Clinical Importance:** TRH stimulation test induced a significant TSH response at all concentrations in adult healthy cats. The results suggest using lower TRH doses to decrease the side effects.

## 89 Abstract EN08

### 89.1 Total Thyroxine and Thyrotropin Concentrations During the Recovery from Non‐Thyroidal Illness in Dogs: Preliminary Results

#### 89.1.1 **Andrea Corsini** ^1^; Francesca Del Baldo^2^; Francesco Lunetta^2^; Serena Ribichini^2^; Massimo Giunti^2^; Federico Fracassi^2^

##### 89.1.1.1 ^1^Department of Veterinary Medical Science, University of Parma; ^2^Department of Veterinary Medical Sciences, University of Bologna

**Background:** Non‐thyroidal illnesses (NTI) can affect serum total thyroxine (tT4) and thyrotropin (TSH) concentrations in dogs.

**Hypothesis and Objective:** To longitudinally evaluate tT4 and TSH in dogs in course of acute NTI and during the recovery phase.

**Animals:** Ten euthyroid client‐owned dogs from a hospital population.

**Methods:** Prospective, longitudinal, observational study. Dogs hospitalized due to acute NTI were enrolled. Serum tT4 and TSH concentrations were measured at the hospital admission (T‐1), every 72 h during the hospitalization, at the discharge (T0), at 3, 7, 14, and 21 days after discharge (T1, T2, T3, T4, respectively). Hypothyroidism was excluded based on tT4 within reference interval (WRI) and absence of clinical signs at T4.

**Results:** Mean (±SD) tT4 was 22.9 (±6.7), 28 (±9.3), 31.3 (±9.8) mmol/L, and mean (±SD) TSH was 0.22 (±0.17), 0.15 (±0.07), 0.31 (±0.19) ng/dL at T‐1, T0, T4, respectively. At T‐1, tT4 was WRI in all dogs and TSH was increased in 1/10 (10%) dogs. One out of 10 (10%) dogs showed low tT4 during the hospitalization. Both tT4 and TSH were WRI in all dogs at T0. Four out of 10 (40%) dogs showed high TSH at least once during the follow‐up period. TSH was increased in 2/10 (20%) dogs at T4. tT4 and TSH comparison between different time‐points showed no difference.

**Conclusions and Clinical Importance:** Serum TSH concentration increases above the upper reference limit in some euthyroid dogs during the recovery phase from NTI. This increase is detectable up to at least 21 days after the resolution of the acute phase of the disease.

**Figure:**
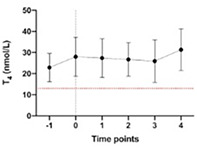
Image 1

## 90 Abstract EN09

### 90.1 Gut Microbial Whole‐Genome Gene Networks and Metabolic Pathways Analysis in Diabetic Cats

#### 90.1.1 **Arnon Gal** ^1^; Patrick Barko^2^, DVM, DACVIM (SAIM); Jenny Applebaum^3^; Paul Orr^4^; Jan Suchodolski^5^, DVM, PHD, DACVM; David Williams^6^

##### 90.1.1.1 ^1^Department of Veterinary Clinical Medicine, University of Illinois, Urbana‐Champaign; ^2^PhD Candidate, Veterinary Clinical Medicine, University of Illinois, Urbana‐Champaign; ^3^DVM Student, Veterinary Clinical Medicine, University of Illinois, Urbana‐Champaign; ^4^DVM Student, Veterinary Clinical Medicine, University of Illinois, Urbana‐Champaign; ^5^Gastrointestinal Laboratory, Texas A&M; ^6^Professor, Small Animal Internal Medicine, Veterinary Clinical Medicine, University of Illinois, Urbana‐Champaign

**Background:** Gastrointestinal microbiota participate in regulating glucose homeostasis. Enteric microbiota dysbiosis has been associated with feline diabetes mellitus (DM), and with metabolic syndrome and DM in humans and other animals, but the functional association of microbial dysbiosis with feline DM has not been investigated.

**Objectives:** Compare gut microbial gene‐networks and metabolic pathways between diabetic and nondiabetic cats.

**Animals:** Ten diabetic and 21 nondiabetic age‐matched cats.

**Methods:** Whole‐genome shotgun sequencing (Illumina NovaSeq) and HMP Unified Metabolic Analysis Network (HUMAnN v3.0) were used to generate fecal metagenomic microbiome profiles. Microbiome Multivariable Associations with Linear Models (MaAsLin v2) was used to compare the abundance of microbial genes and metabolic pathways between groups.

**Results:** The relative abundances of 849 microbial genes from 37 distinct bacterial species belonging to the phyla Firmicutes and Bacteroidetes were significantly decreased in diabetic cats (FDR<0.05). *Faecalibacterium prausnitzii* (404/849; 47.6%) was the most common species associated with genes significantly under‐represented in diabetic cats. Metabolic pathway analysis identified six distinct microbial metabolic pathways significantly under‐represented in diabetic cats; five generate pyruvate and acetyl‐CoA, and the sixth generates the urea cycle intermediates ornithine and agmatine.

**Conclusions:** In health, intestinal gluconeogenesis and ureagenesis critically downregulate hepatic gluconeogenesis and satiety that are deregulated in DM. We speculate that bacterial pyruvate and acetyl‐CoA participate in the feline host intestinal gluconeogenesis, whereas ornithine and agmatine in the host's hepatic ureagenesis. Hence, deficiency in these bacterial metabolic pathways may contribute to the development and maintenance of feline DM through increased hepatic gluconeogenesis and decreased satiety.

## 91 Abstract EN10

### 91.1 Subnetworks of Lipid‐Derivatives and Endocrine‐Disrupting Compounds in Feline Hyperthyroidism

#### 91.1.1 **Arnon Gal** ^1^; Ayelet Ziv‐Gal^2^; Patrick Barko^2^; David Williams^2^; Michael Greener^2^; Nicolas Lopez‐Villalobos^3^; Jodi Flaws^2^; Megan Mahoney^2^

##### 91.1.1.1 ^1^Department of Veterinary Clinical Medicine, University of Illinois, Urbana‐Champaign; ^2^University of Illinois, Urbana‐Champaign; ^3^School of Agriculture and Environment, Massey University

**Background:** Endocrine‐disrupting compounds (EDCs) are prevalent in the environment and have been detected in cat food and blood.

**Hypothesis/Objectives:** Determine the association between EDCs, altered metabolism, and hyperthyroidism.

**Animals:** Sixteen hyperthyroid (10 females, 6 males) and 19 euthyroid (11 females, 8 males) cats.

**Methods:** We prospectively collected urine and blood samples in which we measured creatinine‐normalized urinary EDCs (parabens and phthalates metabolites) and serum lipid metabolites by liquid chromatography‐mass spectrometry, and serum total T4 (tT4). We analyzed the data using a linear mixed effect models and used weighted correlation network analysis to identify subnetworks of serum lipids associated with thyroid status and urinary EDC concentrations.

**Results:** Hyperthyroid cats had significantly (p<0.05) increased ethyl‐paraben and decreased sum‐phthalate metabolites concentrations in their urine, compared with healthy cats. One hundred sixty‐two lipid metabolites significantly differed between groups. We identified four subnetworks of serum lipid metabolites significantly correlated with urinary EDCs and serum tT4. Two subnetworks, one containing triglycerides and another containing sphingomyelins, had positive correlations with ethyl‐, butyl‐, propyl‐ and sum‐paraben metabolites and mono‐methyl phthalate. A third phosphatidylcholines‐dominant subnetwork had positive correlations with methyl‐, propyl‐ and sum‐paraben metabolites. A fourth cardiolipins‐dominant subnetwork had negative correlations with ethyl‐, butyl‐, propyl‐ and sum‐paraben metabolites and mono‐isobutyl phthalate. Serum tT4 positively correlated with the triglycerides, sphingomyelins, and phosphatidylcholines subnetworks and negatively correlated with the cardiolipins subnetwork.

**Conclusions:** Feline hyperthyroidism is associated with differences in urine EDCs and serum lipid derivatives that warrant further investigation as agents that can affect thyroid gland transcription factors and growth.

## 92 Abstract EN11

### 92.1 Interim Analysis of a Prospective Clinical Trial of Fecal Microbial Transplantation in Diabetic Dogs

#### 92.1.1 **Arnon Gal** ^1^; Rebekah Brown^2^; Patrick Barko^2^; Alex Gochenauer^1^; Holly Ganz^3^; Jan Suchodolski^4^; Nicolas Lopez‐Villalobos^5^; Jose Ruiz^1^; David Williams^2^

##### 92.1.1.1 ^1^Department of Veterinary Clinical Medicine, University of Illinois at Urbana‐Champaign; ^2^University of Illinois at Urbana‐Champaign; ^3^AnimalBiome; ^4^Texas A&M; ^5^Massey University

**Background:** Gastrointestinal microbiota participate in regulating glucose homeostasis. Enteric microbiota dysbiosis has been associated with canine diabetes mellitus, and with metabolic syndrome and diabetes in humans and other animals. The effect of fecal microbial transplantation (FMT) on clinical glycemic control in diabetic dogs has not been evaluated.

**Hypothesis/Objectives:** FMT will improve clinical glycemic control in diabetic dogs.

**Animals:** Nine diabetic dogs (4 control; 5 FMT) with median (IQR) age 114 (26) months.

**Methods:** Prospective, double‐blind, placebo‐controlled randomized study. Dogs received daily 1 g of healthy‐donor lyophilized feces or corn‐starch in acid‐resistant capsule for 2 months and were treated with insulin Toujeo per a sliding‐scale insulin dosing protocol that was based on the 60‐minute postprandial interstitial glucose (IG) levels. 24‐hour water intake (24WI) adjusted to bodyweight, and serum fructosamine were analyzed with a linear mixed effect model for repeated measures with age, bodyweight, and baseline values of dependent variables as covariates; period, group and their interaction as fixed effects; and dog as random effect to account for repeated measures on the same dog. The area under the curve (AUC) of IG divided by AUC of daily insulin dose (AUC_IG/ins_) was analyzed by a t‐test, and the percent change from baseline of HbA1C was analyzed by the Mann‐Whitney nonparametric test. *P* value was set <0.05.

**Results:** See figure for results.

**Conclusions:** Our interim analysis cautiously indicates a trend for improved glycemic control in diabetic dogs that were treated with oral lyophilized fecal capsules.

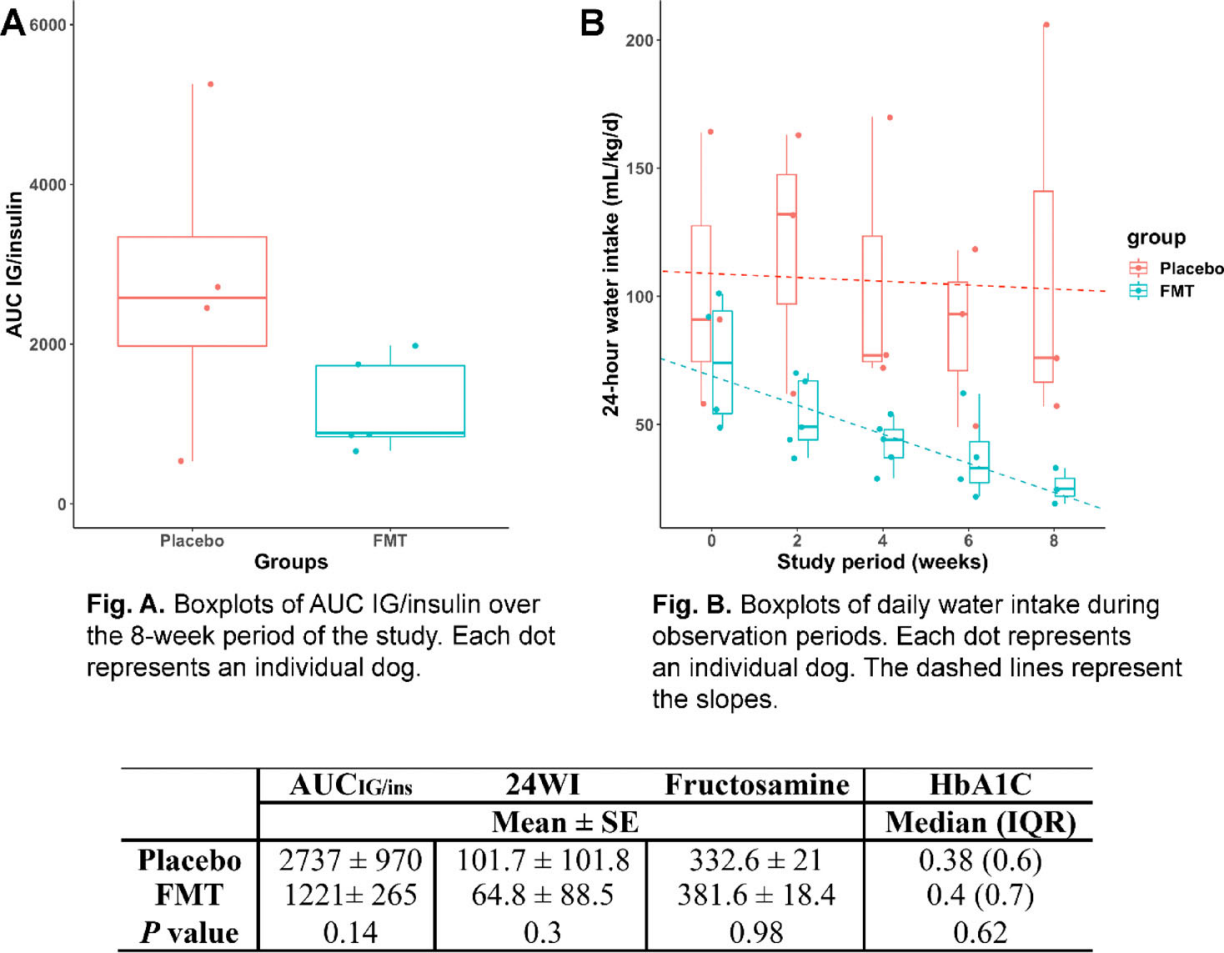

## 93 Abstract EN12

### 93.1 Hypothalamic‐Pituitary‐Adrenal Axis Recovery Following Intermediate‐Acting Glucocorticoids Therapy in Dogs

#### 93.1.1 **Francesca Del Baldo** ^1^; Andrea Corsini^2^; Ada Sapignoli^1^; Alessandro Tirolo^1^; Michele Tumbarello^1^; Katerina Vasylyeva^1^; Federico Fracassi^3^, DVM, PhD, DECVIM‐CA

##### 93.1.1.1 ^1^University of Bologna; ^2^University of Parma; ^3^Associate Professor, University of Bologna

**Background:** In dogs, duration of hypothalamic‐pituitary‐adrenal axis (HPA‐axis) suppression after intermediate‐acting systemic glucocorticoids (IAGC)therapy is reported to vary from a few days up to 7 weeks after GC discontinuation. However, this data derives mainly from experimental studies on healthy dogs and not from animals with spontaneous disease.

**Hypothesis and Objective:** To determine the timeline for recovery of the HPA axis in a group of chronically ill dogs treated with IAGC.

**Animals:** Seventeen client‐owned dogs that received IAGC for at least one week.

**Methods:** Single‐center prospective observational study. ACTH stimulation test, endogenous ACTH (eACTH), serum biochemistry and urinalysis were performed at T0 (2–6 days after IAGC discontinuation)and then every two weeks until the documentation of HPA‐axis recovery (post‐ACTH cortisol >6 μg/dl).

**Results:** Median time of the HPA axis recovery was 3 days (2–133 days). 11/17 dogs (65%) showed recovery of the HPA‐axis at T0, 3/17 (17%) after 15‐17 days and one dog (6%) at 31, 64, 133 days, respectively. Cumulative dose, maximum dose, median daily dose, duration of treatment with IACG and body weight were not correlated with the timing of HPA‐axis recovery. ALT, GGT, ALP and eACTH were significantly correlated with the post‐ACTH cortisol (rs=−0.45, P=0.0059; rs=−0.33, P=0.047; r=−0.30, P=0.045, rs=0.44, P=0.008, respectively). Timing of HPA‐axis recovery in dogs underwent alternate‐day tapering process was not different compared to dogs who did not (3.5 vs. 3 days, P=0.98).

**Conclusion and Clinical Importance:** The majority of dogs showed recovery of the HPA‐axis within 4 days after IAGC discontinuation. However, 12% of dogs required more than 8 weeks.

## 94 Abstract EN13

### 94.1 A Modified Oral Glucose Tolerance Test Detects Mild Dysglycemia Caused by Capromorelin

#### 94.1.1 **Chen Gilor**; Chiquitha Crews, BSc; Adriana Alfonso‐Castro; Susan Hill, MD

##### 94.1.1.1 University of Florida

**Background:** The oral glucose tolerance test (OGTT) is the most sensitive test for detecting prediabetes in people; however, it cannot be used in cats because of the stress associated with force feeding glucose. In non‐diabetic cats, capromorelin blunts insulin secretion and causes glucose intolerance.

**Objectives:** To develop a modified, stress‐free OGTT and assess its ability to detect mild dysglycemia caused by capromorelin in cats.

**Animals:** Ten purpose‐bred healthy cats.

**Methods:** Repeated‐measure study. Blood samples were collected via previously implanted vascular access ports. After 16 h fasting, capromorelin (3 mg/kg) or placebo were administered at ‐60 min, before offering at time zero 4 g/Kg of a highly palatable diet (Hill's a/d), mixed with 0.5 g/Kg of glucose. Blood glucose concentrations (BG) were measured at ‐70, ‐61, ‐10 and ‐1 min before, and at 15, 30, 45, 60, 120, and 180 min post‐feeding. A mixed‐model analysis was used to compare BG between time points and treatments with Bonferroni adjusted p‐values for multiple comparisons.

**Results:** All cats voluntarily consumed the glucose‐rich meal in its entirety in <2 min. Compared to placebo, BG was increased after capromorelin at ‐1, 30, 45, 60 and 180 min (adjusted p<0.05, Figure 1). Post‐feeding, BG best discriminated capromorelin and placebo at 45 min, differing by (median [range]; 95% CI) 40% (12–69%); 29.6–50.1%.

**Conclusions:** This study demonstrates a simple protocol for a stress‐free modified OGTT. Future studies should examine the utility of a one‐sample (45 min post‐feeding) ear‐prick protocol for diagnosis of prediabetes in cats.

**Figure:**
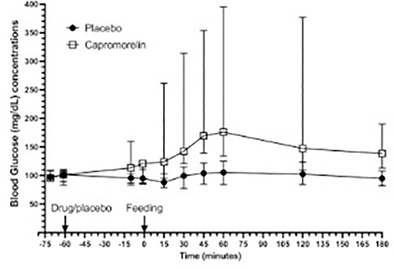
**Image 1.** Median (range) blood glucose concentrations in 10 healthy purpose‐bred cats after feeding a highly palatable diet mixed with glucose (0.5 g/Kg).

## 95 Abstract EN14

### 95.1 Prevalence of Lower Urinary Tract Signs and Positive Urine Culture in Dogs with Diabetes Mellitus

#### 95.1.1 **Valerie Nelson** ^1^; Amy Downey^2^, DVM; Sarah Shropshire^3^, DVM, PhD, DACVIM; Stacie Summers^3^, DVM, PhD, DACVIM

##### 95.1.1.1 ^1^Colorado State University; ^2^Resident, Small Animal Surgery, University of California, Davis; ^3^Assistant Professor, Small Animal Internal Medicine, Colorado State University; ^4^Assitant Professor, Small Animal Internal Medicine, Oregon State University

**Background:** Historically, routine urine cultures were commonly performed in diabetic dogs regardless of lower urinary tract signs (LUTS). However, recent guidelines do not recommend screening urine cultures in diabetic dogs that are not showing LUTS. No recent studies have evaluated the association between clinical signs and positive urine culture as defined under the current guidelines in dogs with diabetes mellitus.

**Objective:** Determine the prevalence of subclinical bacteriuria (i.e., positive urine culture without LUTS) in dogs with diabetes mellitus.

**Animals:** 107 dogs with diabetes mellitus.

**Methods:** Retrospective study evaluating diabetic dogs with a paired urinalysis and urine culture. Relationship between presence of LUTS, pyuria, and bacteriuria and urine culture results were compared using Fisher exact testing.

**Results:** Fifteen dogs (14%) had a positive urine culture, of which 8 (53%) had pyuria, 15 (100%) had bacteriuria, and 4 (27%) had LUTS. Of the 88 dogs (82%) without LUTS, 11 (13%) had a positive culture. A significant association was found between a positive urine culture and the presence of pyuria (OR infinity; 95% CI 20.34–infinity, P<0.00001) and bacteriuria (OR infinity; 95% CI 164.4–infinity, P<0.00001). No association was found between urine culture results and the presence of LUTS (OR 1.87; 95% CI 0.59–6.85, P=0.46).

**Conclusion and Clinical Importance:** Subclinical bacteriuria occurred in this cohort of dogs, and our findings reinforce the recommendation that urine cultures should not be routinely performed in diabetic dogs particularly if pyuria and bacteriuria are also absent.

## 96 Abstract EN15

### 96.1 Relationship Between Urine Concentration and Development of Azotemia After Treatment of Hyperthyroid Cats with Radioiodine

#### 96.1.1 **Mark E. Peterson** ^1^; Dennis Chew^2^, DVM, DACVIM (SAIM); Mark Rishniw^3^, DVM, DACVIM (SAIM)

##### 96.1.1.1 ^1^Animal Endocrine Clinic; ^2^Professor Emeritus, Veterinary Clinical Sciences, College of Veterinary Medicine, The Ohio State University; ^3^Department of Clinical Sciences, College of Veterinary Medicine, Cornell University

**Background:** Many clinicians believe that hyperthyroidism can cause polyuria and sub‐optimally concentrated urine in cats. Prevalence of dilute vs. concentrated urine concentration in hyperthyroid cats, and if this changes after treatment, are unknown.

**Objectives:** To determine proportion of hyperthyroid cats with serum creatinine (SCr) <2.0 mg/dL and USG <1.035 prior to treatment. To determine if urine concentration changes after euthyroidism is restored, and whether an initial USG <1.035 predicts post‐treatment azotemia (SCr >2.0 mg/dL).

**Animals:** 614 hyperthyroid cats.

**Methods:** Prospective, before‐after study. Cats had serum T_4_, T_3_ and creatinine concentrations and USG measured before and 6 months after successful treatment with radioiodine. Cats with pretreatment serum creatinine >2.0 mg/dL or treatment failure were excluded.**Results:** 296/614 (48.2%) hyperthyroid cats had USG <1.035, and 318/614 (51.8%) had USG ≥1.035 before treatment. After treating cats with initial USG <1.035, 257/296 (86.8%) remained <1.035, but 39/296 (13.2%) became concentrated (Figure). After treating cats with USG ≥1.035, most still had USG ≥1.035, but 63/318 (20.4%) became USG <1.035. Fewer cats with pre‐treatment USG ≥1.035 (19/318; 6.0%) developed post‐treatment azotemia than cats with pre‐treatment USG <1.035 (121/296; 40.9%; P<0.001, Chi‐squared). USG <1.035 had a high negative predictive value (94%) for post‐treatment azotemia, but poor positive predictive value (41%).

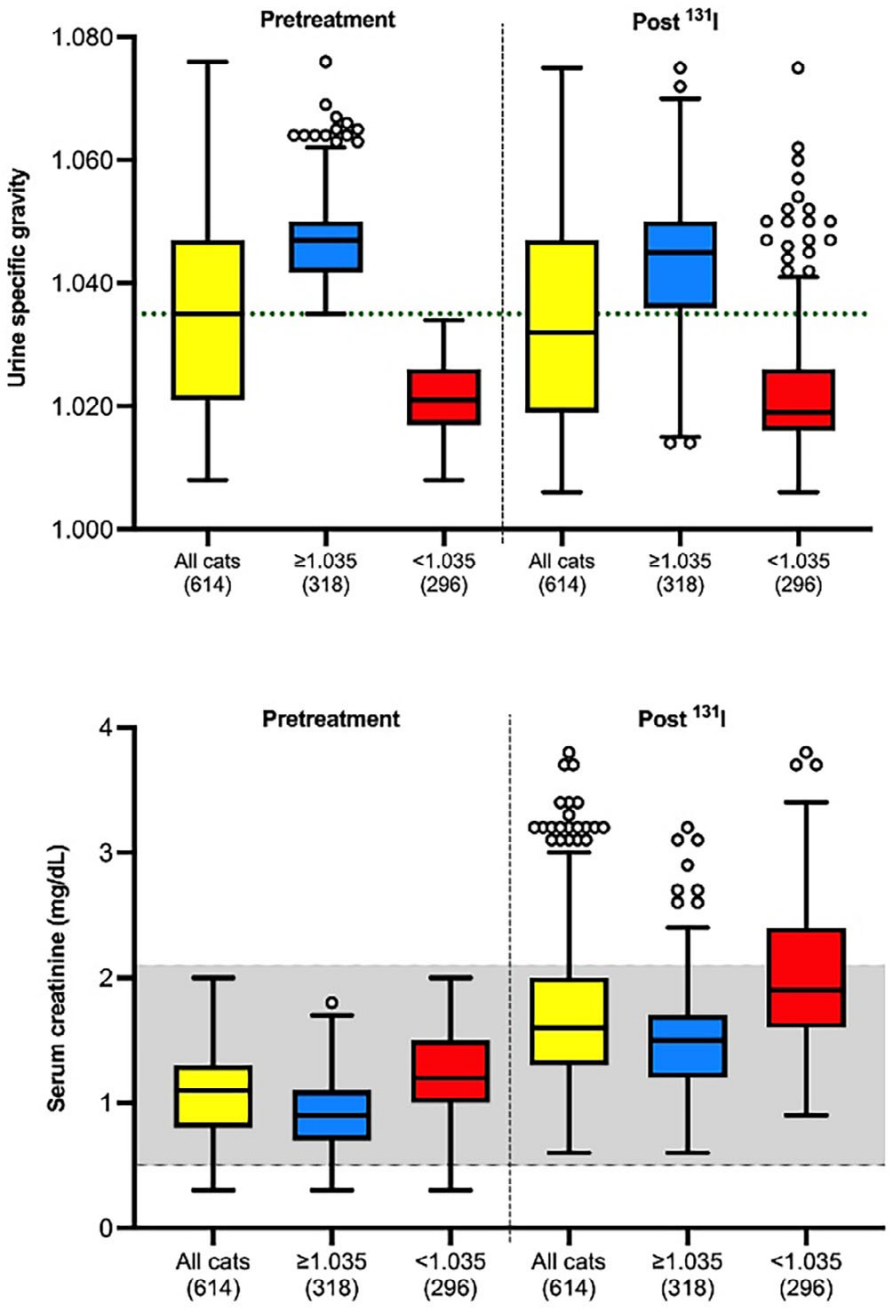

**Conclusions/Clinical Importance:** Hyperthyroid cats have equal prevalence of UGS ≥1.035 vs. <1.035, which tends not to change after radioiodine treatment. Of cats with USG <1.035, nearly half developed SCr >2.0 mg/dL after treatment, whereas cats with USG ≥1.035 had a low probability of developing SCr >2.0 mg/L.

## 97 Abstract EN16

### 97.1 Investigating Hypercoagulability in Diabetic Dogs and Possible Omega‐3 Effect

#### 97.1.1 **Fabio A. Teixeira** ^1^; Vinicius Oliveira^1^, DVM; Tatiane Pooli^1^; Gabriel Santos^1^; Fernanda Tavares^1^; Cristiana Pontieri^2^; Aline Ambrosio^1^; Denise Fantoni^1^; Marcio Brunetto^1^

##### 97.1.1.1 ^1^School of Veterinary Medicine and Animal Science of University, Sao Paulo; ^2^Grandfood Ind and Com LTDA, Premier Pet

**Background:** Hemostatic abnormalities in diabetic subjects result in hypercoagulability. Omega‐3 is an interesting supplementation to endocrinopathies, but it can impact hemostatic system.

**Objectives:** To compare the hemostatic parameters between healthy and diabetic dogs and to investigate impact of omega‐3 supplementation on those parameters.

**Animals:** Seventeen healthy dogs and sixteen diabetic dogs with good glycemic control.

**Methods:** Healthy dogs (HE group) ate for three months the same higher‐fiber extruded diet recommended to diabetic dogs. Diabetic dogs received, randomly and by a double‐blind way, that higher‐fiber diet (DM group) and the same extruded diet enriched with 5.0% inclusion fish oil (DMω3), for three months each period. After each period, a blood sample was collected and coagulation panel [prothrombin time (PT), activated partial thromboplastin time (aPTT), plasmatic fibrinogen levels (FB), and platelet count (PC)] analysis was used to assess coagulation. Data of HE vs. DM and DM vs. DMω3 were compared using T test or Wilcoxon test (p<0.05).

**Results:** There was no age difference between healthy and diabetic dogs (p=0.86). Diabetic dogs had lower PT (p<0.001) and aPTT (p<0.01), and higher FB (p<0.001) and PC (p=0.02) than healthy dogs (Table 1). Supplementation of omega‐3 was approximately 142 mg EPA+DHA/kg body weight. There were no differences (p>0.05) on coagulation panel among dogs receiving or not omega‐3 (Table 1).

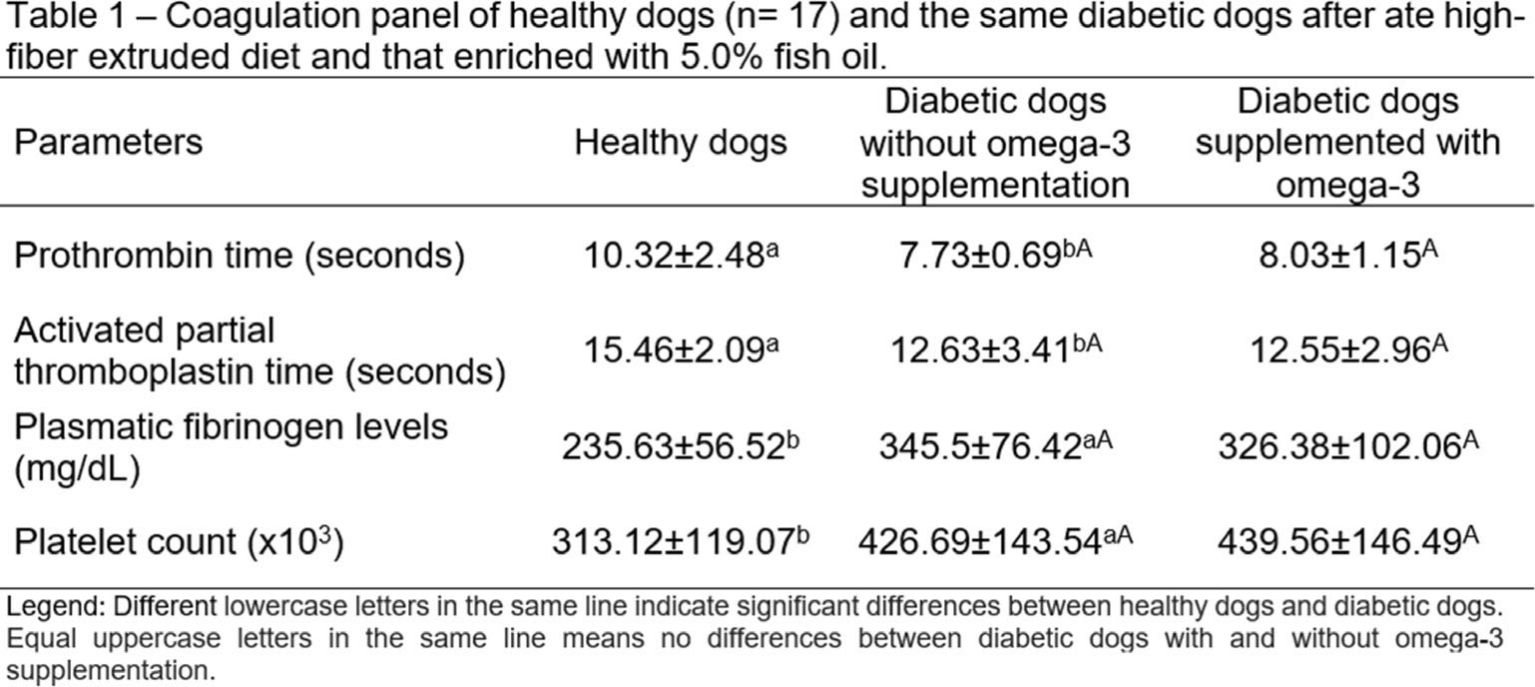

**Conclusions:** Diabetic dogs had hypercoagulopathy profile and fish oil supplementation did not impact PT, aPTT, plasmatic fibrinogen level and platelet counts.

## 98 Abstract EN17

### 98.1 Efficacy and Tolerability of Generic Cinacalcet in Dogs with Primary Hyperparathyroidism

#### 98.1.1 **Heidi Ward** ^1^; Patricia Schenck^2^, DVM, PhD; Mark Lupo^3^, MD, FACE, ECNU; Jillian Dano^4^, PharmD; Dennis Chew^5^

##### 98.1.1.1 ^1^Gulfcoast Veterinary Oncology and Internal Medicine; ^2^Private Consultant; ^3^Medical Director, Thyroid and Endocrine Center of Florida; ^4^Sarasota Apothecary; ^5^Professor Emeritus, College of Veterinary Medicine, The Ohio State University

**Background:** Cinacalcet is approved to treat severe hypercalcemia in humans with primary hyperparathyroidism (PHPT) who are unable to undergo parathyroidectomy. In 2018, generic cinacalcet became available and potentially affordable for veterinary patients.

**Hypothesis:** Cinacalcet will be well‐tolerated and effective in the management of hypercalcemia associated with PHPT in dogs.

**Animals:** Fourteen dogs diagnosed with symptomatic PHPT and ionized calcium values greater than 1.5 mmol/L as measured by the Zoetis iSTAT Alinity.

**Methods:** Cinacalcet was initially dosed at 0.5 mg/kg once daily. Dosages were increased weekly until the iCa fell below 1.45 mmol/L, side effects developed, or a dose of 10 mg/kg BID was reached. PTH was measured at baseline, and again when the target iCa was achieved. SPSS v24 was used to analyze the data with significance at p<0.05.

**Results:** Mean iCa concentration was 1.74±0.19 mmol/L at baseline and 1.39±0.28 mmol/L post‐cinacalcet (p=0.033). PTH levels were 13.68±7.35 pmol/L pretreatment and 9.96±7.31 pmol/L post‐cinacalcet (p=0.194). With doses ranging from 1 mg/kg every 4 days to 9.4 mg/kg BID, 11 (78%) dogs achieved the target iCa <1.45 mmol/L. The average number of days dogs remained within target iCa was 33.6 days (7–84). Side‐effects were observed in 64% of dogs: lethargy and anorexia (9), vomiting (2), and nonhypocalcemic associated episodes of shaking (3).

**Conclusion:** Cinacalcet was effective in significantly decreasing iCa in dogs with PHPT. However, there was considerable inter‐individual variation in dose response, side‐effects, and the duration of within‐target iCa.

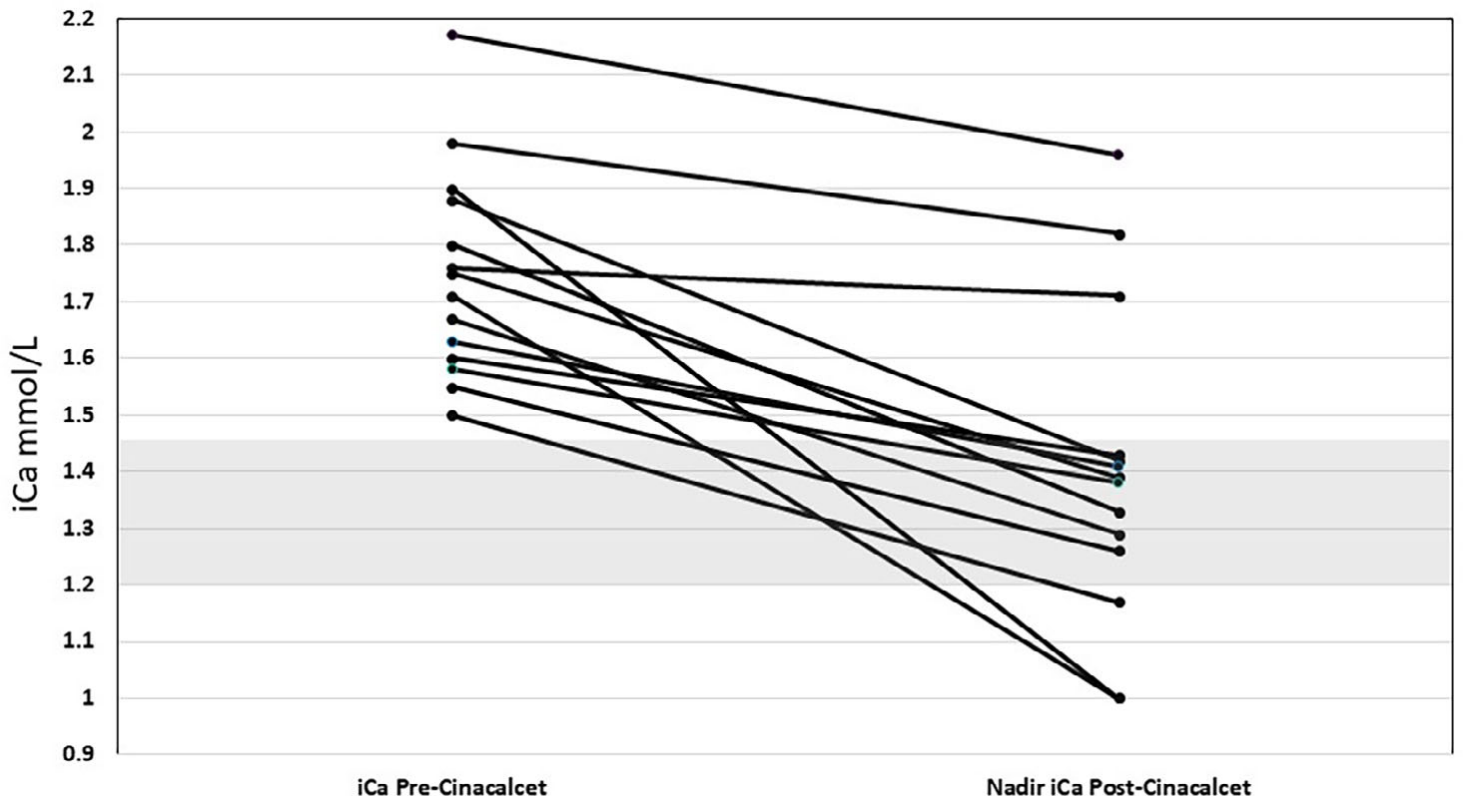

## 99 Abstract EN18

### 99.1 Immunohistochemical Markers of Aldosterone Production in Feline Primary Hyperaldosteronism

#### 99.1.1 **Alice H. Watson** ^1^; Andrew Clear^2^; Morris Brown^2^; Harriet Syme^1^

##### 99.1.1.1 ^1^Royal Veterinary College; ^2^Queen Mary, University of London

**Background:** Cats have one CYP11B enzyme for aldosterone and cortisol synthesis; no validated immunohistochemical marker of aldosterone production exists. Ki67 is a marker of proliferation used to predict malignancy in other tumours.

**Hypothesis/Objectives:** Feline aldosterone production is associated with CYP11B, KCNJ5 and VSNL1 presence, and CYP17A1 absence. Ki67 index is higher in malignant lesions.

**Animals:** Two normal cats and 18 with primary hyperaldosteronism.

**Methods:** Retrospective case series. CYP11B, KCNJ5, VSNL1, CYP17A1 and Ki67 were detected by immunohistochemistry on formalin‐fixed paraffin‐embedded adrenal tissue (normal; n=2, adenoma; n=11, adenocarcinoma; n=7). Tumour protein expression was semi‐quantified by multiplying staining intensity (0–3) by percentage area, giving an overall H‐score (0–300). Percentage of Ki67 positive nuclei was calculated using QuPath. Mann‐Whitney U and Wilcoxon signed‐rank tests were used for independent and paired variables respectively. Results are reported as median [range].

**Results:** Normal zona glomerulosa was CYP11B, KCNJ5 and VSNL1 positive, and CYP17A1 negative. CYP11B (215, [80–300]) had significantly higher expression than CYP17A1 (72.5, [6–155]) in adenomas (p=0.004) and carcinomas (p=0.016). KCNJ5 and VSNL1 expression varied and was not different between adenomas and carcinomas (KCNJ5 220, [0–268] versus 35, [0–290]; p=0.2, VSNL1 210, [0–300] versus 130, [0–295]; p=0.3). Ki67 percentage did not differ (p=0.13) between adenomas (2.09, [0.02–4.29]) and adenocarcinomas (2.6, [0.13–13.46]).

**Conclusions and Clinical Importance:** High CYP11B expression and low, but not absent, CYP17A1 expression appears to be the most reliable indicator aldosterone producing adrenal tissue. Ki67 proliferative index is not predictive of malignancy.

## 100 Abstract EN19

### 100.1 Organoids of Canine Medullary Thyroid Carcinoma and Feline Thyroid Adenomatous Hyperplasia (ESVE Award Winner)

#### 100.1.1 **Stephanie Scheemaeker** ^1^; Miguel Campos^2^; Marine Inglebert^2^; Sylvie Daminet^1^; Martina Dettwiler^3^; Anna Letko^2^; Cord Drögemüller^2^; Federico Massari^4^; Martin Kessler^5^; Sven Rottenberg^2^

##### 100.1.1.1 ^1^University of Ghent; ^2^University of Bern; ^3^Vetscope Pathologie Dettwiler; ^4^DOCVET Clinica Veterinaria Nervianese; ^5^Tierklinik Hofheim

**Background:** Aetiology and pathophysiology of canine medullary thyroid carcinoma (cMTC) and functional feline thyroid adenomatous hyperplasia (fTAH) remain poorly understood. 3D organoid culture could constitute an excellent *in vitro* model.

**Hypothesis/Objectives:** Establish and characterize patient‐derived organoid cultures of cMTC and fTAH, explore new therapeutic options for cMTC in cMTC organoids and evaluate the effect of TSH on growth and thyroid hormone (TH) production in fTAH organoids.

**Animals:** One frozen cMTC and fTAH patient‐derived tissue sample.

**Methods:** Organoids from one cMTC and fTAH were cultured. Histology and immunohistochemistry for thyroglobulin, calcitonin, vimentin and Ki‐67 were performed. While immunohistochemistry for thyroid transcription factor‐1 (TTF‐1) and synaptophysin was also performed on cMTC organoids. CellTiter‐Blue® viability assays were used to evaluate the effect of carboplatin, meloxicam and toceranib phosphate on cMTC organoid growth and to evaluate the effect of different concentrations of TSH on fTAH organoid growth. TH production by fTAH organoids was evaluated after incubation with sodium iodide and different TSH‐concentrations.

**Results:** Organoids were cultured for 99 (cMTC) and 65 (fTAH) days. Organoids resembled the primary tissue histologically. TTF‐1 and calcitonin immunolabeling in cMTC organoids confirmed thyroid and C‐cell origin. The fTAH organoid cells showed thyroglobulin immunolabeling (5–10%) and no calcitonin immunolabeling. No drug had effect on cMTC organoid growth. TSH had no effect on fTAH organoid growth and no TH production was detected.

**Conclusions and Clinical Importance:** Organoid cultures provide a promising *in vitro* model to explore pathophysiology and new treatment modalities for cMTC and fTAH.

## 101 Abstract EN20

### 101.1 Variability Between Two Flash Glucose Sensor Locations in Non‐Diabetic Dogs During Rapidly Induced Hypoglycemia

#### 101.1.1 **Jeremy B. Evans** ^1^; Jonathan Lidbury^2^, BVMS, MRCVS, PhD, DACVIM, DECVIM‐CA; Nicholas Jeffery^1^, DVM, PhD; Shannon Washburn^3^, DVM, PhD; Carly Patterson^4^, DVM, DACVIM

##### 101.1.1.1 ^1^Veterinary Teaching Hospital, Texas A&M University; ^2^Associate Professor, Veterinary Teaching Hospital, Texas A&M University; ^3^Veterinary Medicine & Biomedical Sciences, Texas A&M University; ^4^Clinical Assistant Professor, Veterinary Teaching Hospital, Texas A&M University

**Background:** Flash glucose monitoring systems (FGMS) are frequently used for convenient interstitial glucose (IG) monitoring in dogs with diabetes mellitus and are typically placed between the scapulae.

**Objective:** To evaluate the variability between glucose measurements from FGMS placed in 2 locations (between the scapulae and over the hip) in non‐diabetic dogs during rapidly induced hypoglycemia.

**Animals:** Twenty‐four apparently healthy colony dogs that were subjects in a teaching laboratory.

**Methods:** Prospective interventional study. FGMS (FreeStyle Libre, Abbott) were placed between the scapulae and over the hip of all dogs. Regular insulin was administered at 0.3 u/kg intravenously and the subsequent hypoglycemia was corrected. Prior to insulin administration and every 10 minutes over a 90‐minute period, IG was recorded from both locations with FGMS. Concurrently at each timepoint, blood glucose was obtained with a portable blood glucose meter (PBGM) from a venous blood sample. Measurement between FGMS locations were compared using Bland Altman plots (average vs. scapulae—hip) and Passing‐Bablok regression.

**Results:** A total of 209 out of a possible 240 paired FGMS measurements were available for comparison. There was a constant bias of 5.6 mg/dL with 95% limits of agreement of ‐26.3 to 37.5 mg/dL between FGMS locations. There was a correlation between FGMS locations (r=0.731; intercept=‐2.676; slope=0.971).

**Conclusions and Clinical Importance:** In this model of rapidly induced hypoglycemia, there was wide divergence between IG paired sample readings from FGMS placed between the scapulae and over the hip.

## 102 Abstract EN21

### 102.1 Geographic Prevalence of Naturally Occurring Feline Hyperthyroidism

#### 102.1.1 **JoAnn Morrison**; Nate Spofford, MPH

##### 102.1.1.1 Banfield Pet Hospital

**Background:** The etiology of feline hyperthyroidism has not been completely elucidated. As part of ongoing research, the geographic prevalence of feline hyperthyroidism was investigated.

**Hypothesis/Objectives:** A non‐uniform geographic distribution of feline hyperthyroidism could generate additional insight into pathophysiology.

**Animals:** Privately owned cats with naturally occurring hyperthyroidism presented to 1084 Banfield Pet Hospitals from July 12, 2020, to July 17, 2021. All hospitals function as primary care facilities and do not offer specialized services (e.g., radioactive iodine). There was no directed recruitment of affected cats, and this population was anticipated to approximate naturally occurring illness.

**Methods:** Electronic medical records were retrospectively reviewed to identify cases. Inclusion criteria were a clinical diagnosis of feline hyperthyroidism and/or total thyroxine concentration greater than or equal to 5.5 μg/dl or 60 nmol/L. The percentage of unique patients diagnosed with hyperthyroidism was calculated. Direct standardization was applied to calculate age‐adjusted disease prevalence by state using Banfield's overall feline population as the standard population and six age groups (delineated by years): 0–<8, 8–10, 11–13, 14–16, 17–19 and 20+.

**Results:** Overall hyperthyroid prevalence was 177 per 10,000 cats seen (95% CI, 174–180 per 10,000). The Pacific Northwest and the Eastern Seaboard had the highest prevalence of feline hyperthyroidism (≥200 cases per 10,000; Image 1).

**Figure:**
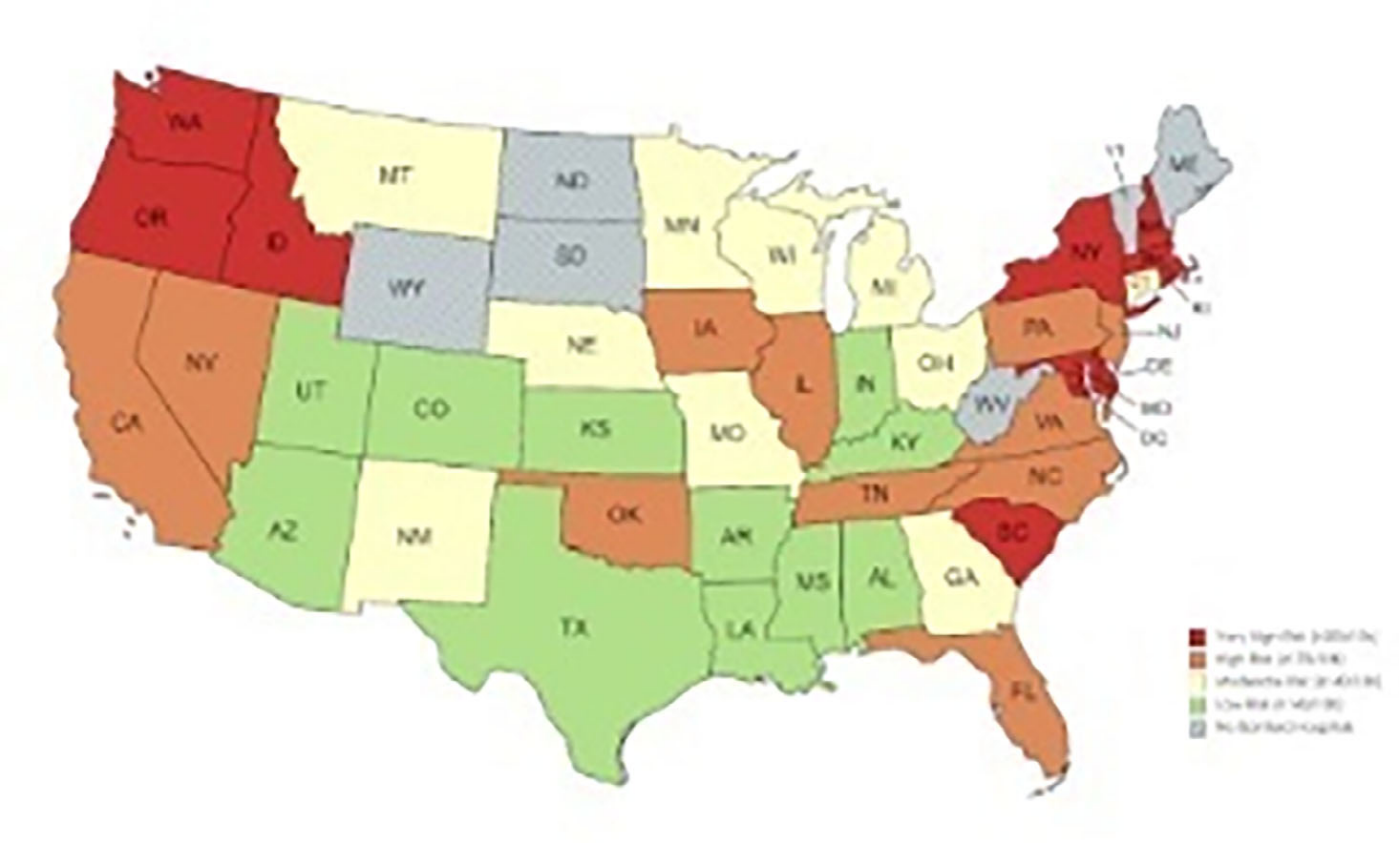
**Image 1.** Geographic prevalence of feline hyperthyroidism in the United States, July 2020–2021

**Conclusions and Clinical Importance:** This study presents new information that may be helpful in determining risk factors for the development of feline hyperthyroidism. By delineating specific areas of increased risk, further investigations may be focused on dietary, environmental, genetic or other factors.

## 103 Abstract EN22

### 103.1 Diagnosis of Canine Hyperadrenocorticism Using a Point‐of‐Care Cortisol Assay

#### 103.1.1 **Andrew J. Narwold, Jr.** ^1^; Cynthia Ward^2^, VMD, PHD, DACVIM (SAIM); Ashley Wood^1^, PhD

##### 103.1.1.1 ^1^Zomedica Inc.; ^2^CRW Consulting

**Background:** Hyperadrenocorticism, commonly referred to as Cushing's disease, is a common endocrine disorder in dogs. For suspected Cushingoid dogs, a low‐dose dexamethasone test (LDDST) is considered the diagnostic test of choice. In‐clinic testing for Cushing's disease would be more convenient for the practitioner and allow for more timely results. An appropriate testing platform with well‐defined and accurate hormone measurements is needed.

**Objective:** Assess the diagnostic performance of canine cortisol measurements for Cushing's disease at the point of care.

**Animals:** Forty‐one canines with clinical and clinicopathologic signs of Cushing's disease from three veterinary clinics and 40 healthy research colony canines.

**Methods:** All animals underwent a LDDST and serum cortisol levels were measured using the in‐clinic TRUFORMA® diagnostic platform. Animals suspected of Cushing's disease underwent a physical exam and screening workup, including CBC, chemistry, urinalysis, and LDDST performed at a reference lab. Blinded to TRUFORMA results, a board‐certified endocrinologist diagnosed the animals based on the reference lab results in addition to clinical and clinicopathologic signs.

**Results:** Healthy animals showed reduced cortisol levels 8 hours post‐dexamethasone injection compared to animals suspected, but not diagnosed with Cushing's disease (p=0.0225). Animals suspected and then diagnosed with Cushing's disease showed an increased cortisol level at 8 hours post‐dexamethasone injection (p=<0.0001). Using a diagnostic cutoff of 2.5 μg/dL for the 8‐hour TRUFORMA cortisol concentration, 40 of the 41 suspected animals (97.6%) were diagnosed in agreement with the endocrinologist.

**Conclusion:** Using an appropriate cutoff, the TRUFORMA point‐of‐care cortisol assay successfully distinguished animals with hyperadrenocorticism.

**Figure:**
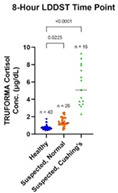
Image 1

## 104 Abstract EN23

### 104.1 Evaluation of a Flash Glucose Monitoring System in Diabetic Cats.

#### 104.1.1 **Nicola Steers**; Stefanie DeMonaco, DVM, MS, DACVIM (SAIM); Timothy Bolton, DVM, DACVIM (SAIM); Virginia Corrigan, DVM, MPH, DABVP, CCRP

##### 104.1.1.1 Virginia‐Maryland Regional College of Veterinary Medicine

**Background:** Diabetes mellitus is a common endocrine disease in cats. Good glycemic control may help achieve remission. The flash glucose monitoring system (FGMS—FreeStyle Libre system; FSL) allows for 14‐day continuous monitoring of interstitial blood glucose.

**Hypothesis/Objectives:** To evaluate the correlation of blood glucose concentration when measured by the FGMS as compared to the portable blood glucose monitor (PBGM—AlphaTRAK; AT) and the laboratory standard (hexokinase method; HM).

**Animals:** Twenty‐nine diabetic cats from hospital population. Exclusion criteria: diabetic ketoacidosis, dehydration, dermatological disease, fractious nature.

**Methods:** Prospective, observational study. Sensors placed on the lateral thorax on cats at initial visit. In‐hospital and at‐home blood glucose curves (BGC) performed using AT and FSL in each cat. Plasma glucose concentration assessed by HM once per in‐hospital curve. Correlations between FSL and AT, and FSL and HM evaluated using Lin's concordance correlation coefficient (CCC). Strength of agreement defined as excellent (>0.99), substantial (0.95–0.99), moderate (0.90–0.95), and poor (<0.90) based on Lin's CCC value. Adverse sensor wear events recorded.

**Results:** Two‐hundred fifty‐two glucose measurements compared between AT and FSL, and poor agreement was found. Thirty‐seven glucose measurements compared between FSL and HM, and substantial agreement was found. Median duration of sensor operation 9 days (range 1–13 days). Adverse events minor including skin irritation and temporary alopecia.

**Conclusions and Clinical Importance:** The FGMS is accurate compared to the HM and should be considered to facilitate at home BGC for diabetic cats. Sensors do not operate for the full period in a majority of patients.

## 105 SMALL ANIMAL INTERNAL MEDICINE ‐ GASTROENTEROLOGY

## 106 Abstract GI01

### 106.1 Evaluation of 48‐hour Gastric pH and Serum Gastrin Concentrations in Dogs with Chronic Kidney Disease

#### 106.1.1 **Elijah S. Ernst** ^1^; Patricia Secoura^2^; Josh Price^3^; Adam Birkenheuer^4^; Shelly Vaden^5^; Jonathan Lidbury^6^; Emily Gould^6^; Jörg Steiner^6^; M. Katherine Tolbert^6^

##### 106.1.1.1 ^1^North Carolina State University, Raleigh, NC, USA; ^2^Department of Clinical Sciences, College of Veterinary Medicine, North Carolina State University, Raleigh, NC, USA; ^3^Department of Small Animal Clinical Sciences, University of Tennessee, College of Veterinary Medicine, Knoxville, TN, USA; ^4^Department of Clinical Sciences, College of Veterinary Medicine, North Carolina State University, Raleigh, NC, USA; ^5^Department of Clinical Sciences, College of Veterinary Medicine, North Carolina State University, Raleigh, NC, USA; ^6^Gastrointestinal Laboratory, Department of Small Animal Clinical Sciences, College of Veterinary Medicine and Biomedical Sciences, Texas A&M University, College Station, TX, USA

**Background:** Gastric hyperacidity is reported to cause gastric ulceration in dogs with chronic kidney disease (CKD), yet there are no studies evaluating gastric pH in dogs with CKD.

**Hypothesis/Objectives:** Compare gastric pH and serum gastrin concentrations in dogs with CKD to healthy, age‐matched dogs.

**Animals:** Client‐owned dogs with CKD (International Renal Interest Society [IRIS] Stage I‐IV) and age‐matched, healthy dogs. Inclusion criteria for control dogs were absence of azotemia, USG >1.015, and normal body condition. Exclusion criteria for all dogs included historical or biochemical evidence of hepatobiliary disease, primary gastrointestinal disease, uncontrolled endocrine disease, or use of interfering drugs, including acid suppressants within 7 days of pH monitoring.

**Methods:** Prospective, case‐control study. Minimum database and serum gastrin concentration were evaluated within 24 hours of pH monitoring, which was performed by radiographic‐assisted placement of a pH capsule in the gastric fundus. Continuous pH recordings were compared over time and between groups using a repeated measures mixed‐model ANOVA.

**Results:** Thirteen dogs with CKD (mean±SD, age: 8.9±5.0 y) and 11 healthy dogs (age: 8.3±3.3 y) were enrolled. No significant differences were observed between groups for any pH parameters, including mean gastric pH (CKD: 2.37±0.87; healthy: 2.31±0.98; p=0.77). Serum gastrin concentrations for all dogs were within the reference interval.

**Conclusions and Clinical Importance:** Dogs with CKD do not have gastric hyperacidity compared to age‐matched, healthy dogs. These results do not support the routine use of acid‐suppressing drugs in dogs with CKD.

## 107 Abstract GI02

### 107.1 Clinical and Gastrointestinal Changes in Healthy Research Dogs Administered Prednisone, Prednisone/Omeprazole, or Prednisone/Probiotics

#### 107.1.1 **Mariola B. Rak** ^1^; Tamberlyn Moyers^2^, LVMT VTS (Nutrition); Joshua Price^3^, MS; Jacqueline Whittemore^4^, DVM, DACVIM (SAIM), PhD

##### 107.1.1.1 ^1^College of Veterinary Medicine, University of Tennessee; ^2^Department of Small Animal Clinical Sciences, College of Veterinary Medicine, University of Tennessee; ^3^ASA PStat Accredited Professional Statistician, The Office of Information Technology, Veterinary Medical Center, University of Tennessee; ^4^Clinical Associate Professor of Medicine, Veterinary Medical Center, Department of Small Animal Clinical Sciences, University of Tennessee

**Background:** Previously evaluated gastroprotectants did not prevent glucocorticoid‐induced gastric bleeding in dogs, but neither probiotics or twice‐daily omeprazole were evaluated.

**Hypothesis/Objectives:** Compare gastrointestinal bleeding among dogs administered prednisone, prednisone with omeprazole, or prednisone with probiotics.

**Animals:** Twenty‐four healthy research dogs.

**Methods:** Double‐blinded, placebo‐controlled randomized trial. Dogs received placebo, prednisone (2 mg/kg q 24 h), prednisone/omeprazole (1 mg/kg q 12 h), or prednisone/probiotics (11.2–22.5 billion CFU/kg q 24 h) for 28 days. Clinical signs and endoscopic gastrointestinal mucosal lesions were determined at baseline, day 14, and day 28 of treatment. Results were compared using split‐plot repeated measures mixed model ANOVAs.

**Results:** Attitude, vomiting, and food intake did not differ among groups. Fecal score differed over time depending on treatment received (F [6,40]=2.65, P<0.029). *Post hoc* tests revealed scores increased in the prednisone‐receiving groups between baseline and day 28 (P<0.001, for each). Nineteen of 33 (58%) instances of fecal score ≥5 occurred in the prednisone/omeprazole group. Gastric mucosal lesion scores differed by treatment‐by‐time (F [6,60]=2.86, P=0.016), between treatments (F [3,60]=4.9, P=0.004), and over time (F [2,60]=16.5, P<0.001). *Post hoc* tests revealed lesion scores increased between baseline and days 14 and 28 for prednisone‐receiving groups (P≤0.03, for all). Ulcers occurred in 6 dogs: prednisone alone, 2; prednisone/omeprazole, 2; and prednisone/probiotics, 3.

**Conclusions and Clinical Importance:** Consistent with previous studies, prednisone induced gastric bleeding. Bleeding was not mitigated by co‐administration of omeprazole or probiotics.

## 108 Abstract GI03

### 108.1 An Undernutrition Screening Score Is Associated with Treatment Response in Dogs with Inflammatory Protein‐Losing Enteropathy

#### 108.1.1 **Florence E. Wootton** ^1^; Christopher Hoey^2^, BSc, BVetMed, PGDVCP, MRCVS; Glynn Woods^3^; Silke Salavati Schmitz^3^, PhD, PGCert, FHEA, DECVIM‐CA, FRCVS; Jenny Reeve^2^, BVSc, DECVIM‐CA, MRCVS, FHEA; Aarti Kathrani^3^, BVetMed (Hons), PhD, DACVIM (SAIM), DACVIM (Nutrition), FHEA

##### 108.1.1.1 ^1^Royal Veterinary College; ^2^Internal Medicine, University of Bristol; ^3^Internal Medicine, The Royal (Dick) School of Veterinary Studies

**Background:** The impact of undernutrition in dogs with inflammatory protein‐losing enteropathy (iPLE) is currently unknown.

**Objectives:** To determine if an undernutrition screening score (USS) at the time of histopathologic diagnosis is associated with length of hospitalization, treatment response, clinical and biochemical remission, and death in dogs with iPLE.

**Animals:** Fifty‐seven dogs with iPLE prospectively recruited from three referral hospitals in the U.K.

**Methods:** Dogs with iPLE were recruited at the time of histopathologic diagnosis. The USS and the canine chronic enteropathy clinical activity index (CCECAI) were recorded at this time, along with historical and clinicopathologic data. The USS was scored out of 18 and consisted of parameters related to appetite, weight loss, and body, muscle and skin condition, with higher scores reflecting worse undernutrition. Follow‐up was obtained for at least 6‐months after histopathologic diagnosis.

**Results:** Dogs that were hospitalized for >5 days had higher USS (p=0.005) and CCECAI (p<0.001) at diagnosis. Dogs that failed to respond to treatment prescribed at diagnosis had higher USS (p=0.011) and CCECAI (p=0.002). Dogs that failed to achieve clinical remission within 6‐months had higher USS (p=0.037) and CCECAI (p=0.041). Dogs that died or were euthanized due to iPLE within 6‐months of diagnosis had higher CCECAI (P=0.001). The USS and CCECAI were positively correlated (p=0.001, correlation‐coefficient=0.414).

**Conclusions and Clinical Importance:** A higher USS at diagnosis is associated with negative outcomes in dogs with iPLE. Further studies are required to determine if nutritional intervention to correct undernutrition at diagnosis, helps to improve these outcome parameters.

## 109 Abstract GI04

### 109.1 Serum Cobalamin and Methylmalonic Acid Concentrations in Dogs with Parvoviral Enteritis

#### 109.1.1 **Michael G. Hung** ^1^; Justin Heinz^2^, DVM, DACVECC; Joerg Steiner^3^, MedVet, DrMedVet, PhD, DACVIM, DECVIM‐CA, AGAF; Jan Suchodolski^4^, MedVet, DrVetMed, PhD, AGAF, DACVM; Jonathan Lidbury^5^, BVMS, MRCVS, PhD, DACVIM, DECVIM‐CA

##### 109.1.1.1 ^1^Small Animal Teaching Hospital, Texas A&M University; ^2^Clinical Assistant Professor, Veterinary Medical Teaching Hospital, Small Animal Clinical Sciences, Texas A&M University; ^3^Director, Gastrointestinal Laboratory, College of Veterinary Medicine and Biomedical Sciences, Texas A&M University; ^4^Associate Director for Research, Gastrointestinal Laboratory, College of Veterinary Medicine and Biomedical Sciences, Texas A&M University; ^5^Associate Professor, Veterinary Medical Teaching Hospital, Small Animal Clinical Sciences, Texas A&M University

**Background:** Canine parvovirus causes significant intestinal damage, potentially resulting in malabsorption. Measurement of cobalamin is used as a marker of ileal malabsorption in patients with chronic enteropathy. Prevalence of cobalamin deficiency in dogs with parvoviral enteritis has not been described.

**Objective:** Determine serum cobalamin and methylmalonic acid (MMA) concentrations in dogs with parvoviral enteritis, healthy dogs, and dogs with non‐parvovirus acute diarrhea (non‐PVAD) of similar age.

**Animals:** Thirty‐one healthy control dogs, 30 dogs with parvoviral enteritis (positive fecal parvovirus antigen test or PCR), and 24 dogs with non‐PVAD (negative antigen test and PCR). Dogs were <10 months and had not received cobalamin supplementation.

**Methods:** Prospective, case‐control study. Serum cobalamin (all dogs) and MMA (63 dogs) concentrations were measured within 24 hours of presentation.

**Results:** Median (min–max) cobalamin concentrations were 784 ng/L (293–1,619) in the healthy group, 447 ng/L (<150–805) in the parvovirus group, and 527 ng/L (160–8,838) in the non‐PVAD group. Serum MMA concentrations were 815 mmol/L (588–1,466), 858 (554–2,870), and 776 (392–831). Serum cobalamin concentrations were significantly lower in the parvovirus group than in the healthy group (p=0.0002), but not the non‐PVAD group (p=0.1517). No significant differences in serum MMA were found (p=0.1623).

**Conclusions and Clinical Importance:** Dogs with parvoviral enteritis had lower serum cobalamin concentrations than healthy controls, but did not appear to have cobalamin deficiency at the cellular level.

## 110 Abstract GI05

### 110.1 The Impact of Fecal Identification Markers on the Feline Microbiome

#### 110.1.1 **Alexandra Wood** ^1^; Nora Jean Nealon^2^, DVM, PhD; Hannah Klein^3^, DVM; Adam Rudinsky^4^, DVM, MS, DACVIM (SAIM); Matt Salerno^5^; Jessica Quimby^4^, DVM, PhD, DACVIM (SAIM); Valerie Parker^6^, DVM, DACVIM (SAIM), DACVN; James Howard^7^, DVM, MS, DACVS (SA); Jenessa Winston^7^, DVM, PhD, DACVIM (SAIM)

##### 110.1.1.1 ^1^College of Veterinary Medicine, The Ohio State University; ^2^Post Doctoral Scholar, Veterinary Clinical Sciences, College of Veterinary Medicine, The Ohio State University; ^3^Internal Medicine Resident, Veterinary Clinical Sciences, College of Veterinary Medicine, The Ohio State University; ^4^Associate Professor, Veterinary Clinical Sciences, College of Veterinary Medicine, The Ohio State University; ^5^Veterinary Student, Veterinary Clinical Sciences, College of Veterinary Medicine, The Ohio State University; ^6^Professor, Clinical, Veterinary Clinical Sciences, College of Veterinary Medicine The Ohio State University; ^7^Assistant Professor, Clinical, Veterinary Clinical Sciences, College of Veterinary Medicine, The Ohio State University

**Background:** Fecal collection in cats is required for research and clinical purposes but fecal identification is limited by feline elimination behaviors in group‐housing and multi‐cat households. Crayon shavings and glitter have been used as fecal markers, but their impact on the fecal microbiome is unknown.

**Objective:** To determine the impact of fecal identification markers, specifically crayon and glitter, on the feline microbiome.

**Animals:** This clinical trial was performed in six healthy and sterilized purpose‐bred cats (3 male, 3 female) group‐housed in a dedicated research facility.

**Methods:** A randomized crossover experiment was performed. Cats received either glitter or crayon shavings mixed in a feline adult maintenance diet for 14 days. A 14‐day washout was performed between crossovers. Feces was collected daily from each cat for 16S rRNA gene sequencing (V4 region). Amplicon analysis was performed in R‐studio (dada2; phyloseq). Pairwise PERMANOVA analysis compared samples across experimental phases and marker order.

**Results:** Fecal identification markers were readily identified in the feces of all cats. Statistically significant changes to the microbiome were identified with marker order (P<0.05) and across experimental phases (P<0.05) for 5/6 cats. Microbiome changes were cat‐dependent (P<0.001), suggesting idiosyncratic microbiome responses to glitter and crayon.

**Conclusions and Clinical Importance:** Both glitter and crayon allow for identification of individual feline fecal samples; however, these markers may impact the microbiome and therefore are not recommended in studies with microbiome endpoints. The difference in response to these markers among cats highlights the individuality of the feline microbiome.

## 111 Abstract GI06

### 111.1 Effect of Nutrient Profile on Post‐Prandial GLP‐2 Plasma Concentration in Cats

#### 111.1.1 **Leah S. Freilich** ^1^; Maria Jugan^2^, DVM, MS, DACVIM (SAIM); Zackery Bieberly^3^

##### 111.1.1.1 ^1^Kansas State University; ^2^Assistant Professor, Small Animal Internal Medicine, Clinical Sciences, Kansas State University; ^3^DVM Candidate 2022, Clinical Sciences, Kansas State University

**Background:** Glucagon‐like peptide‐2 (GLP‐2) is an enteroendocrine hormone responsible for gastrointestinal (GI) health with nutrient‐dependent secretion.

**Hypothesis/Objectives:** Maximal GLP‐2 plasma concentration occurs 30 minutes after meal ingestion, and a high‐fat meal provides greater GLP‐2 secretion stimulus than high‐protein or high‐carbohydrate meals.

**Animals:** Nine healthy research cats.

**Methods:** This was a randomized, cross‐over study. Cats were fed a standardized high‐fat, high‐carbohydrate, or high‐protein meal after an overnight fast. Cats received all 3 diets in random order with a 24‐hour period between sampling days. Blood samples were collected at baseline, 30, 60, 75, 90, and 120 minutes post‐meal. Plasma GLP‐2 concentration was measured using a commercial feline ELISA. The Friedman test (non‐normal data) or one‐way repeated measures ANOVA (normal data) was used to evaluate maximal GLP‐2 secretion within each diet. GLP‐2 concentrations were compared among diets using a mixed analysis of variance accounting for repeated measures.

**Results:** Plasma GLP‐2 concentration 30 minutes post high‐fat meal (1.64±0.23 ng/mL) was higher than 90 minutes (1.39±0.31 ng/mL; P=0.03) and 120 minutes (1.44±0.27 ng/mL; P=0.03) post‐meal. There was no difference in post‐prandial GLP‐2 concentration after the high‐protein or high‐carbohydrate meal compared to baseline. There was no difference in post‐prandial GLP‐2 concentrations between the meal types.

**Conclusions and Clinical Importance:** Results suggest that ingested fat is a small but significant stimulus for GLP‐2 secretion in cats. Further understanding of GLP‐2 secretion may help diagnose and treat feline GI disorders.

## 112 Abstract GI07

### 112.1 Relationship Between Magnesium, Calcium, and Parathyroid Concentrations in Dogs with Decreased 25(OH)D and Chronic Enteropathy

#### 112.1.1 **Charles Jones** ^1^; Sara Jablonski Wennogle^2^, DVM, PhD, DACVIM (SAIM); Brian Petroff^3^, DVM, PhD; Daniel Langlois^4^, DVM, DACVIM (SAIM)

##### 112.1.1.1 ^1^Michigan State University; ^2^Assistant Professor, Small Animal Clinical Sciences, Michigan State University; ^3^Professor, Pathobiology and Diagnostic Investigation, Michigan State University; ^4^Associate Professor, Small Animal Clinical Sciences, Michigan State University

**Background:** Dogs with chronic enteropathy (CE) or protein‐losing enteropathy (PLE) and low serum 25‐hydroxyvitamin D (25[OH]D) variably develop ionized hypocalcemia and/or secondary hyperparathyroidism (SHPT). Serum magnesium concentrations influence calcium homeostasis and secretion of PTH. However, little is known about the relationship between magnesium and the development of ionized hypocalcemia and SHPT in dogs with chronic gastrointestinal (GI) disease and decreased 25[OH]D.

**Objectives:** Evaluate relationships between ionized magnesium (iMg) and PTH, ionized calcium (iCa), and 25(OH)D in dogs with CE +/‐ PLE and decreased 25(OH)D.

**Animals:** 50 dogs with CE +/‐ PLE and decreased serum 25(OH)D.

**Methods:** Retrospective search of submissions database at Michigan State University Veterinary Diagnostic Laboratory for vitamin D profiles submitted 2017–2020. Submitting practices contacted to obtain consent for use of patient data, confirmation of GI disease, and case information. Cases excluded if supplemented with Ca, Mg, or vitamin D. Spearman correlation performed to evaluate relationships between iMg and PTH, 25(OH)D, and iCa. Ionized Mg concentrations compared between dogs with SHPT and those with normal parathyroid hormone concentrations.

**Results:** Concentrations of iMg were weakly negatively correlated with PTH concentrations (rho,‐0.31; P=0.032), and weakly positively correlated with serum 25(OH)D (rho,.34, P=0.02) and iCa (rho,.42, P=0.003) concentrations. Dogs with decreased 25(OH)D and SHPT were more likely to have ionized hypomagnesemia compared to dogs with decreased 25(OH)D and normal parathyroid hormone concentrations (P=0.015).

**Conclusions and Clinical Importance:** Hypomagnesemia may contribute to alterations in iCa and parathyroid hormone in dogs with CE +/‐ PLE and decreased 25(OH)D.

## 113 Abstract GI08

### 113.1 Fecal Pancreatitis‐Associated‐Protein‐1 and Gastrotropin as Biomarkers for Subgroup Identification in Canine Chronic Enteropathies

#### 113.1.1 **Karin Allenspach** ^1^; Chris Chadwick, PhD^2^; David Eckersall^3^, DVM, PhD; Vojtech Gabriel^4^, DVM; Chelsea Iennarella‐Servantez^5^, BS, MS, DVM/PhD; Lingnan Yuan^6^, PhD; Jonathan Mochel^7^, DVM, MS, PhD, DECVPT

##### 113.1.1.1 ^1^Iowa State University; ^2^President, LifeDiagnostics, Inc.; ^3^Professor of Veterinary Biochemistry, School of Veterinary Medicine, University of Glasgow; ^4^PhD Student, Biomedical Sciences, Iowa State University; ^5^Dual Degree Student, Biomedical Sciences, Iowa State University; ^6^Post‐Doctoral Scientist, Biomedical Sciences, Iowa State University; ^7^Associate Professor of Pharmacology, Biomedical Sciences, Iowa State University

Non‐invasive biomarkers to predict treatment responses in canine chronic enteropathies (CE) are urgently needed. In a recent exploratory proteomic study, several pancreatic/intestinal enzymes, acute phase proteins and immunoglobulins (Ig) were found to be differentially present in feces from healthy dogs and dogs with CE. In this follow‐up study, we sought to evaluate the discriminatory ability of these biomarkers to predict subgroups of dogs with CE.

Fifty‐eight dogs with CE were included in the study (25 food‐responsive diarrhea (FRD), 12 antibiotic‐responsive diarrhea (ARD), 10 steroid‐responsive diarrhea (SRD), 11 chronic diarrhea of other causes). Spatial Proximity Analyte Reagent Capture Luminescence (SPARCL^TM^) assays were developed to measure the fecal concentrations of the following analytes: pancreatitis‐associated‐protein‐1 (PAP1), haptoglobin (HP), S100A8, IgG1, IgG2, IgGFc, alpha‐1‐acid glycoprotein (AGP), ceruloplasmin, C‐reactive protein (CRP), intestinal fatty acid‐binding protein (FABP), liver‐FABP, and gastrotropin. Multivariate logistic regression was used to assess the predictive ability of analytes to differentiate between CE and chronic diarrhea of other causes, and between subgroups of CE.

Fecal levels of gastrotropin were predictive of CE vs. chronic diarrhea due to other causes. In addition, dogs with lower fecal HP and CRP were more likely to be FRD. Furthermore, higher fecal concentrations of PAP‐1 were significantly associated with ARD. For SRD, higher CCECAI and weight loss, and higher fecal concentrations of IgG1, IgG2, CRP and gastrotropin were identified as markers with predictive ability.

In conclusion, this study suggests that novel fecal biomarkers PAP‐1 and gastrotropin could be predictive of ARD and SRD, respectively, in dogs with CE.

## 114 Abstract GI09

### 114.1 Utility of Fluorescence Imitating Brightfield Imaging Microscopy for the Diagnosis of Feline Chronic Enteropathy

#### 114.1.1 **Sarah G. Au Yeung** ^1^; Paula Giaretta^2^; Taryn Morningstar^3^, BS; Eduardo Masuda^4^; Maria Questa^5^, MS, PhD; Farzad Fereidouni^6^, PD; Richard Levenson^7^, MD; Sina Marsilio^8^, DrMedVet, DACVI (SAIM), DECVIM‐CA

##### 114.1.1.1 ^1^School of Veterinary Medicine, UC Davis; ^2^Gastrointestinal Laboratory, Department of Small Animal Clinical Sciences, College of Veterinary Medicine and Biomedical Sciences, Texas A&M; ^3^Pathology and Laboratory Medicine, UC Davis Health; ^4^Axys Análises; ^5^Postdoctoral Scholar, Veterinary Medicine and Epidemiology, School of Veterinary Medicine, UC Davis; ^6^Assistant Adjunct Professor, Pathology and Laboratory Medicine, UC Davis Health; ^7^Professor and Vice Chair for Strategic Technologies, Pathology and Laboratory Medicine, UC Davis Health; ^8^Assistant Professor, Veterinary Medicine and Epidemiology, School of Veterinary Medicine, UC Davis

**Background:** Fluorescence imitating brightfield imaging (FIBI) is a novel microscopy method allowing for real‐time, non‐destructive, slide‐free tissue imaging of fresh, formalin‐fixed, or even paraffin‐embedded tissue. The non‐destructive nature of technology permits tissue preservation for further downstream analysis.

**Hypothesis/Objectives:** To assess the utility of FIBI compared to conventional H&E‐stained histology slides in feline gastrointestinal histopathology.

**Animals:** Formalin‐fixed paraffin‐embedded (FFPE) full‐thickness small intestinal tissue specimens from 50 cases of feline chronic enteropathy (FCE).

**Methods:** Observational study. The ability of FIBI to evaluate predetermined morphological features (epithelium, villi, crypts, lacteals, fibrosis, submucosa, muscularis propria) and inflammatory cells was assessed on a 3‐point scale (0=FIBI cannot identify the feature; 1=FIBI can identify the feature; 2=FIBI can identify the feature with more certainty than H&E). H&E and FIBI images were also scored according to World Small Animal Veterinary Association (WSAVA) Gastrointestinal Standardization Group guidelines.

**Results:** FIBI identified morphological features with similar or in some cases higher confidence compared to H&E images (subscore 0.90). The identification of inflammatory cells was less consistent (subscore 0.48). FIBI and H&E showed an overall poor agreement with regards to the assigned WSAVA scores.

**Conclusions and Clinical Importance:** While FIBI showed an equal or better ability to identify morphological features in intestinal biopsy specimens, identification of inflammatory cells is currently inferior compared to H&E‐based imaging. Future studies on the utility of FIBI as a diagnostic tool for non‐inflammatory histopathologic lesions are warranted.

## 115 Abstract GI10

### 115.1 Altered Fecal and Serum Amino Acid Concentrations in Dogs with Chronic Enteropathy

#### 115.1.1 **Amanda B. Blake**; Patricia Ishii; Robert Phillips; Jonathan Lidbury; Joerg Steiner; Jan Suchodolski

##### 115.1.1.1 Gastrointestinal Lab, Veterinary Small Animal Clinical Sciences, Texas A&M University

**Background:** Amino acids (AA) affect many metabolic processes and serve as an important energy source for enterocytes. Alterations in plasma or serum AA concentrations have previously been reported in dogs and cats with gastrointestinal (GI) disease. However, studies suggest that measurement of fecal AA may be more useful in discriminating human inflammatory bowel disease (IBD) patients from healthy controls.

**Objectives:** Compare fecal and serum AA concentrations between dogs with chronic enteropathy (CE) and healthy control dogs (HC).

**Animals:** Feces and serum were collected from HC dogs (n=29 and 23, respectively) and dogs with CE (n=29 and 15, respectively). GI biopsies were also collected (stomach, small intestine, colon; HC=13 and CE=8).

**Methods:** Retrospective cross‐sectional study. Fecal and serum AA concentrations (measured on an Agilent HPLC and Biochrom 30+ AA analyser, respectively) were compared between groups with Mann‐Whitney tests. Correlation analysis was performed between fecal and serum AA concentrations and histopathological scores. Results were corrected for multiple comparisons using Benjamini‐Hochberg method.

**Results:** Fecal concentrations of tryptophan were significantly higher (Q=0.023) in dogs with CE than in HC dogs. Serum concentrations of valine were significantly higher (Q=0.026) in dogs with CE than in HC. After correction for multiple comparisons, all correlations with histopathological scores failed to reach statistical significance.

**Conclusions:** Fecal and serum AA profiles are altered in dogs with CE. Therefore, some AA may have the potential to be used as future therapeutic targets.

## 116 Abstract GI11

### 116.1 Prevalence and Clinical Features of Dogs with Chronic Enteropathy at Two Large Swedish Animal Hospitals

#### 116.1.1 **Johanna Holmberg** ^1^; Lena Pelander^2^, DVM, PhD, DECVIM‐CA (SAIM); Ingrid Ljungvall^3^, DVM, PhD, DECVIM‐CA (Cardiology); Caroline Harlos^4^, DVM; Thomas Spillmann^5^, DVM, PhD, DECVIM‐CA (SAIM); Jens Häggström^6^, DVM, PhD, DECVIM‐CA (Cardiology)

##### 116.1.1.1 ^1^Swedish University of Agricultural Sciences; ^2^Assistant Professor, Clinical Sciences, Swedish University of Agricultural Sciences; ^3^Associate Professor, Clinical Sciences, Swedish University of Agricultural Sciences; ^4^Anicura Albano; ^5^Professor, University of Helsinki; ^6^Professor, Clinical Sciences, Swedish University of Agricultural Sciences

**Background:** Information about prevalence of canine chronic enteropathy (CE) is limited.

**Objectives:** Investigate period prevalence, clinical features and diagnostic results of CE in dogs at two Swedish animal hospitals.

**Animals:** Eight hundred fourteen dogs with CE.

**Methods:** A retrospective medical record search was performed to identify dogs with CE including dogs with ≥3 visits because of gastrointestinal disease and/or undergone gastroduodenoscopy/colonoscopy during 2013–2018. Dog characteristics, case history, physical examination, diagnostic results, therapeutic protocol and response were recorded.

**Results:** Eight hundred fourteen dogs met inclusion criteria for CE. Period prevalence of CE was 1.1% of total number of dogs visiting the hospitals, and 4.4% of all dogs presenting with gastrointestinal signs. Breeds with highest risk were Norwegian Lundehund, West Highland White Terrier, and Miniature Poodle. Dogs presented at median age of 3.8 (IQR 1.8–6.8) years. French Bulldogs and Miniature Schnauzers presented younger (below 2.5 years) compared to other breeds (P<0.05). In a subset of dogs, hypoalbuminemia (116/662, 17.5%), hypocobalaminemia (98/647, 15.1%), and increased C‐reactive protein (145/267, 54.3%) were recorded. Treatment outcome was classified in 72.9% of dogs, and characterized as immunosuppressant‐responsive (55.2%), food‐responsive (11.4%), non‐responsive (5.2%) and antibiotic‐responsive (1.1%). Non‐responsive dogs were more likely to present with hypoprotein/albuminemia, anemia, and high CRP (P<0.05).

**Conclusions and Clinical Importance:** Prevalence of dogs with CE at Swedish hospitals was in agreement with earlier reports. Risk breeds differed slightly from previous reports and earlier age of CE onset was found in two breeds. The largest proportion of dogs was immunosuppressant‐responsive and the smallest antibiotic‐responsive.

## 117 Abstract GI12

### 117.1 Effects of Fecal Microbiota Transplantation on the Fecal Microbiome in Healthy Cats Administered Amoxicillin Clavulanate

#### 117.1.1 **Jamie Hui** ^1^; Frederic Gaschen^2^, DrMedVet, DrHabil, DACVIM, DECVIM‐CA; Chi‐Hsuan Sung^3^, DVM; Rachel Pilla^4^; Jan Suchodolski^5^, MedVet, DrVetMed, PhD, AGAF, DACVM

##### 117.1.1.1 ^1^Louisiana State University; ^2^Professor, Veterinary Clinical Sciences, Louisiana State University; ^3^Graduate Research Assistant, Gastrointestinal Laboratory, Department of Small Animal Clinical Sciences, Texas A&M University; ^4^Research Assistant Professor, Gastrointestinal Laboratory, Department of Small Animal Clinical Sciences, Texas A&M University; ^5^Professor and Associate Director for Research, Head of Microbiome Sciences, Gastrointestinal Laboratory, Department of Small Animal Clinical Sciences, Texas A&M University

**Background:** Intestinal dysbiosis occurs frequently in cats with gastrointestinal diseases or following antibiotic administration. Fecal microbiota transplantation (FMT) may be beneficial in people and dogs with gastrointestinal disease, but data in cats is limited.

**Objectives:** To investigate the effects of FMT in shortening the recovery of the fecal microbiome in healthy cats after amoxicillin and clavulanate administration.

**Animals:** Eighteen healthy research cats.

**Methods:** Prospective, randomized, controlled, IACUC‐approved clinical trial. All cats received a 10‐day course of antibiotics, then were randomized to receive FMT via enema (n=10) or no treatment (n=8). Fecal samples were collected at baseline, immediately after antibiotic administration, and 7, 14, 21 and 56 days later. Abundance of 8 bacterial taxa was determined via qPCR and a fecal feline dysbiosis index (FDI) was calculated. A 2‐way repeated measures ANOVA with *post hoc* pairwise comparisons was used.

**Results:** The FDI increased in all cats after antibiotics (baseline ‐2.4±0.1, post‐antibiotics 2.7±0.4, p<0.0005). No adverse reactions were observed during or following FMT. The FDI of control cats was still increased compared to baseline 7, 14 and 21 days after antibiotic discontinuation (0.4±1.9; 0.5±1.9; ‐0.1±2.2 and ‐1.3±2.4 respectively, all p<0.05) while it was not significantly different from baseline in FMT‐treated cats.

**Clinical Importance:** FMT was a well‐tolerated procedure that has the potential to accelerate reversal of dysbiosis in this feline model of acute antibiotic‐induced dysbiosis. The efficacy of FMT seen in this study may serve as a platform for investigation of its role in cats with various enteropathies.

## 118 Abstract GI13

### 118.1 Abstract GI13:Efficacy of a Synbiotic‐IgY Supplement in Treatment of Canine Inflammatory Bowel Disease—Clinical Observations

#### 118.1.1 **Albert E. Jergens** ^1^; Karin Allenspach^1^; Jonathan Mochel^2^, DVM, PhD; Agnes Bourgois‐Mochel^1^; Dipak Sahoo^1^; Chris Zdyrski^1^; Vojtech Gabriel^1^; Sichao Mao^1^; Chelsea IIennarella‐Servantez^1^; Yuan Lingnan^1^; Yu Ting Liu^1^; Adam Rudinsky^3^; Jenessa Winston^3^; Valerie Parker^3^

##### 118.1.1.1 ^1^Iowa State University; ^2^Associate Professor, Iowa State University; ^3^The Ohio State University

**Background:** Dysbiosis is associated with mucosal inflammation in dogs with inflammatory bowel disease (IBD). Treatments attempting to reduce mucosal inflammation by normalizing dysbiotic states are a rapidly growing research area. A dietary supplement containing a synbiotic and avian Immunoglobulin Y (IgY) was developed to correct gut dysbiosis, reduce gastrointestinal (GI) signs, and target intestinal inflammation.

**Objectives:** To investigate the efficacy of a synbiotic/IgY supplement on clinical disease activity, intestinal histopathology, gut microbiome, and serum metabolome in dogs with IBD.

**Animals:** Twenty dogs diagnosed with IBD were randomized to receive a hydrolyzed diet with (n=11) or without (n=9) synbiotic/IgY supplement for 6 weeks. Dogs failing remission after two weeks of primary treatment also received oral cyclosporine at 5 mg/kg.

**Methods:** Clinical disease activity, endoscopic lesions, histologic inflammation, and frequency of treatment escalation were assessed in both groups before and after treatment. Biologic samples for microbiologic and metabolomic analyses were archived for later analysis.

**Results:** There were no differences in baseline clinical parameters between groups (p>0.05). Final data analysis showed decreased clinical disease activity and endoscopic scores in both groups after treatment (p<0.05). Five dogs required treatment escalation with four of five dogs maintaining remission until trial completion. There was no difference in histologic inflammation pre‐ versus post‐treatment for either group (p>0.05).

**Conclusions:** While this study was likely underpowered, the administration of a synbiotic/IgY supplement with hydrolyzed diet over 6 weeks was well tolerated and associated with reduced clinical disease activity and endoscopic scores, like placebo.

## 119 Abstract GI14

### 119.1 Characterisation of the Culturable Duodenal Mycobiota of Dogs with Chronic Enteropathy

#### 119.1.1 **Aarti Kathrani** ^1^; Ross Bond^1^; Bart Theelen^2^

##### 119.1.1.1 ^1^Royal Veterinary College; ^2^Westerdijk Fungal Biodiversity Institute

**Background:** Fungi play a role in the pathogenesis of human inflammatory bowel disease.

**Objective:** To explore the presence of culturable fungi in the duodenum of dogs with chronic gastrointestinal signs undergoing routine endoscopic examination.

**Animals:** Forty‐five client‐owned dogs with chronic gastrointestinal signs from our referral hospital.

**Methods:** Quantitative microbial culture was performed on duodenal juice; samples were cultured on Sabouraud's dextrose agar (30 & 37°C) and modified Dixon's agar (32°C) for 14‐days. Isolates were identified phenotypically, genotypically (ITS‐sequencing), and by Matrix‐Assisted Laser Desorption Ionization‐time Of Flight. Yeast presence was evaluated by cytologic and histopathologic examination of smears and biopsy specimens.

**Results:** Forty‐five dogs were recruited with chronic inflammatory enteropathy (CIE; n=38), granulomatous colitis (n=2), gastric adenocarcinoma (n=2), duodenal small cell lymphoma (n=1), and idiopathic gastrointestinal hemorrhage (n=2). Fungi were cultured from 14 dogs: *M. pachydermatis* from eight (CIE [n=7] [along with *Candida albicans* n=1]; granulomatous colitis [n=1]), and *M. sympodialis* from another (gastric adenocarcinoma). Five dogs with CIE yielded other yeasts (*C. albicans*, *C. glabrata*, *Kazachstania slooffiae*, *K. telluris*, *Pichia kudriavzevii* [syn. *C. krusei*]). Dogs with yeast growth had significantly higher serum vitamin B12 (p=0.003), canine chronic enteropathy clinical activity index (p=0.034), and dermatologic signs (p=0.043) at the time of isolation versus those with no growth.

**Conclusions and Clinical Importance:** To our knowledge, this is the first report of isolation of *M. pachydermatis*, and *M. sympodialis* spp. from the canine duodenum. Further studies are needed to determine whether their presence has a pathogenic effect and whether antifungal therapy is warranted.

## 120 Abstract GI15

### 120.1 Evaluation of a Commercial Serologic Panel Marketed for the Diagnosis of Chronic Enteropathy in Dogs

#### 120.1.1 **Daniel K. Langlois** ^1^; Jessica Pritchard^2^, DVM, MS, DACVIM (SAIM); M. Katherine Tolbert^3^, DVM, PhD, DACVIM (SAIM); Gary Block^4^; Andrew Hanzlicek^5^; Joerg Steiner^3^; Jared Jaffey^6^; Sara Jablonski Wennogle^1^

##### 120.1.1.1 ^1^College of Veterinary Medicine, Michigan State University; ^2^School of Veterinary Medicine, University of Wisconsin; ^3^College of Veterinary Medicine and Biomedical Sciences, Texas A&M University; ^4^Ocean State Veterinary Specialists; ^5^MiraVista Diagnostics; ^6^College of Veterinary Medicine, Midwestern University

**Background:** It has been suggested that a panel of IgA‐based serologic assays aids in the diagnosis of chronic enteropathy (CE) in dogs, a syndrome which includes conditions such as food‐responsive enteropathy, immunosuppressant responsive enteropathy, and inflammatory bowel disease (also referred to as chronic inflammatory enteropathy). However, it is unclear whether these markers discriminate between CE and other types of primary intestinal disorders.

**Objectives:** To evaluate a commercially available diagnostic panel that measures serum levels of IgA directed against OmpC (ACA), canine calprotectin (ACNA), and gliadin‐derived peptides (AGA) in dogs with well‐characterized intestinal diseases.

**Animals:** Thirty‐two dogs with primary intestinal disease.

**Methods:** Serum ACA, ACNA, and AGA levels were measured in dogs with CE and dogs with other forms of primary intestinal disease (non‐CE population), including bacterial, fungal, parasitic, and neoplastic enteropathies. Serum IgA levels were compared between populations, and sensitivities and specificities were calculated using laboratory‐provided cut‐points.

**Results:** Twelve of 15 (80.0%) CE dogs and 14 of 17 (82.4%) non‐CE dogs had abnormally high levels of at least 2 markers; these proportions were not significantly different (P=0.99). A serum ACA level ≥15 EU/mL was 80.0% sensitive for CE, but specificity was only 29.4%.

**Conclusions and Clinical Importance:** The serologic panel was moderately sensitive, but poorly specific for a CE diagnosis. Common treatments for CE, such as dietary modifications and immunosuppressants, might delay appropriate therapy or even be detrimental for many of the patients with non‐CE diseases that commonly showed high ACA, ACNA, and AGA levels.

## 121 Abstract GI16

### 121.1 Efficacy of an Elemental Diet in Achieving Clinical Remission in Dogs with Chronic Inflammatory Enteropathy

#### 121.1.1 **Alison C. Manchester** ^1^; Steven Dow^2^, DVM, PhD, DACVIM (SAIM); Jason Gagne^3^, DVM, DACVN; Michael Lappin^2^, DVM, PhD, DACVIM (SAIM)

##### 121.1.1.1 ^1^Colorado State University; ^2^Professor, Colorado State University; ^3^Nestle Purina

**Background:** Diet may induce clinical remission in dogs with chronic inflammatory enteropathy (CIE). Elemental diets (ED) made from individual amino acids are highly digestible with minimal antigenic stimulation and can be effective for remission from Crohn's disease in humans.

**Hypothesis/Objectives:** To evaluate whether exclusive ED feeding would help CIE dogs achieve clinical remission.

**Animals:** Client‐owned dogs (n=23) not experiencing adequate relief from their CIE were enrolled in this prospective, uncontrolled study.

**Methods:** A thorough workup including upper and lower gastrointestinal endoscopy and biopsy was performed. CIBDAI, serum chemistry and cytokine concentrations (MILLIPLEX®) were evaluated before and after 2 (T2) and 8 weeks (T3) of ED feeding. Values were compared using a mixed model with P values adjusted for multiple comparisons.

**Results:** Eight dogs exited the study before T3; one would not eat the diet, 1 required prednisone for unrelated reasons, and 5 had ongoing signs of CIE. At T2, 16/22 dogs (73%) accepting the diet were experiencing adequate relief with median [range] decrease in CIBDAI from 6 [3–12] to 2 [0–9] (P<0.005). All 16 dogs remaining at T3 were experiencing adequate relief with a median CIBDAI of 2 [0–3]. Serum ALP concentrations [mean±SD] were higher at T3 [106 U/L±57]) than before feeding ([50±34]; P<0.005). Differences in serum albumin, cholesterol, and cytokine concentrations were not detected.

**Conclusions and Clinical Importance:** The ED was palatable for 95% of dogs and successfully induced clinical remission in 73%. Work is ongoing to determine the mechanisms behind the ED's positive effects.

## 122 Abstract GI17

### 122.1 To Sample or Not to Sample: Capturing Feline Fecal Microbiome Changes with High‐Frequency Sample Collection

#### 122.1.1 **Nora Jean Nealon** ^1^; Hannah Klein^2^, DVM; Matt Salerno^3^; Adam Rudinsky^4^, DVM, MS, DACVIM (SAIM); Jessica Quimby^4^, DVM, PhD, DACVIM (SAIM); Valerie Parker^5^, DVM, DACVIM (SAIM), DACVN; James Howard^6^, DVM, MS, DACVS‐SA; Jenessa Winston^6^, DVM, PhD, DACVIM (SAIM)

##### 122.1.1.1 ^1^College of Veterinary Medicine ,The Ohio State University; ^2^Internal Medicine Resident, Veterinary Clinical Sciences, College of Veterinary Medicine, The Ohio State University; ^3^Veterinary Student, Veterinary Clinical Sciences, College of Veterinary Medicine, The Ohio State University; ^4^Associate Professor, Veterinary Clinical Sciences, College of Veterinary Medicine, The Ohio State University; ^5^Professor, Clinical, Veterinary Clinical Sciences, College of Veterinary Medicine, The Ohio State University; ^6^Assistant Professor, Veterinary Clinical Sciences, College of Veterinary Medicine, The Ohio State University

**Background:** Microbiome‐based fecal evaluations are promising diagnostic tools for assessing feline health. However, given inherent daily changes in the gut microbiome, the ideal sampling frequency is unknown.

**Hypothesis:** High‐frequency fecal sampling is more effective than weekly sampling at capturing changes in the feline fecal microbiome in response to a new diet.

**Animals:** This clinical trial was performed in six healthy and sterilized purpose‐bred cats (3 male, 3 female) group‐housed in a research facility.

**Methods:** All cats were fed an adult commercial wet food diet, at maintenance needs, to which they had no previous exposure. Feces was collected daily for 36 days. The 16s rRNA microbiome analysis (V4 region) was performed using R‐Studio. Pairwise PERMANOVA evaluated changes in composition. A subset of data (once‐weekly samples) was compared to the high‐frequency (daily) dataset to evaluate daily versus weekly fecal sampling effects.

**Results:** With high‐frequency sampling, the fecal microbiome composition of each cat differed by day (P<0.01). Despite being co‐housed, daily sampling identified distinct microbiome compositions between cats at all time points (P<0.0001). With once weekly sampling, no compositional differences between cats were detected and there were decreased differences over time (sampling week) (P<0.05) for all cats.

**Conclusions and Clinical Importance:** Daily sampling or pooled samples may more accurately capture fecal microbiome dynamics compared to weekly sampling. Ongoing analysis will evaluate taxa contributing to daily versus weekly microbiome changes and compare sampling frequencies (e.g., twice weekly) to daily sampling to establish appropriate sampling frequencies for future microbiome studies.

## 123 Abstract GI18

### 123.1 Evaluation of Intestinal Permeability in Dogs with Exocrine Pancreatic Insufficiency

#### 123.1.1 **Patricia Eri Ishii** ^1^; Vinicius V. Oliveira^2^; Fabio Teixeira^3^; Barbara Codeas^3^; Rafael Oliveira^3^; Daniela Machado^4^; Cristiana Pontieri^4^; Joerg Steiner^1^; Jan Suchodolski^1^; Marcio Brunetto^3^

##### 123.1.1.1 ^1^Gastrointestinal Laboratory, Department of Small Animal Clinical Sciences, Texas A&M University; ^2^University of São Paulo; ^3^Veterinary Nutrology Service, Teaching Veterinary Hospital, School of Veterinary Medicine and Animal Science, University of São Paulo; ^4^Grandfood Industry and Commerce LTDA (Premier Pet)

**Background:** Exocrine pancreatic insufficiency (EPI) is characterized by insufficient synthesis and secretion of enzymes by the exocrine pancreas. This in turn leads to maldigestion and intestinal dysbiosis, and may or may not lead to disturbances in intestinal integrity. Disturbances of the intestinal integrity can be assessed by evaluation of intestinal permeability (IP) and have been reported in humans with EPI. Iohexol has recently been described as a marker for IP in healthy dogs and dogs with chronic enteropathy.

**Objectives:** To compare IP between dogs with EPI and healthy control dogs receiving the same diet.

**Animals:** Twenty dogs were selected for this study: 10 dogs with EPI, currently showing no clinical signs and receiving pancreatic enzyme replacement therapy (PERT) and 10 healthy control dogs. Study and control group were matched according to size and age

**Methods:** Dogs received the same extruded commercial diet. Food was withheld for 12 hours before oral administration of 2.0 mL/kg of iohexol. Blood samples were collected from the jugular vein two hours after iohexol administration, centrifuged, and frozen

**Results:** Serum iohexol concentrations were significantly higher (p=0.0147) in dogs with EPI on PERT (median: 60.6 μg/mL range: 21.0–186.3) compared to healthy control dogs (median: 29.0 μg/mL range: 20.7–56.7).

**Conclusions:** This study suggests that despite receiving PERT, showing no clinical signs, and maintaining normal body condition, dogs with EPI show an increased IP when compared to healthy control dogs. An increase in IP may contribute to the gastrointestinal dysfunctions in dogs with EPI.

## 124 Abstract GI19

### 124.1 Metabolic Profiling of Serum Samples from Cats with Chronic Enteropathy

#### 124.1.1 **Sina Marsilio** ^1^; Mara Questa^1^; Betty Chow^2^; Steve Hill^3^; Jonathan Lidbury^4^; Joerg Steiner^4^; Jan Suchodolski^4^; Sina Marsilio^1^

##### 124.1.1.1 ^1^University of California, Davis; ^2^Veterinary Specialty Hospital; ^3^Flagstaff Veterinary Internal Medicine Consulting (FLG VIM‐C); ^4^Gastrointestinal Laboratory, Texas A&M University

**Background:** Alterations of the fecal metabolome have previously been described in cats with chronic enteropathy (CE). Characterizing the serum metabolome could provide further information on alterations of metabolic pathways and may identify diagnostic or therapeutic targets.

**Hypothesis/Objectives:** To characterize the serum metabolome of cats with CE before and after treatment.

**Animals:** Serum samples from 14 healthy cats and 26 cats with CE (13 cats with inflammatory bowel disease (IBD), 13 cats with small cell intestinal lymphoma (SCL)), before and 4 weeks after treatment with corticosteroids and/or chlorambucil.

**Methods:** Untargeted metabolomic analysis of serum using high performance chromatography–tandem mass spectroscopy. Differences between healthy and CE cats were evaluated using the Wilcoxon rank sum test, adjusted using false discovery rate and expressed as q‐values. Statistical significance was set at q<0.05.

**Results:** Metabolomics analysis identified 1,082 named metabolites, of which 130 were significantly different between cats with CE and healthy controls at baseline. Random forest analysis revealed a predictive accuracy of 85% for differentiating controls from cats with CE. Metabolic pathways found to be significantly altered included polyunsaturated fatty acid, phospholipid, amino acid, and indole metabolism. Several metabolites were found to be significantly different between cats with IBD versus SCL, including 3,4‐dihydroxybenzoic acid, malic acid, arginine, and several fatty acids. Metabolic profiles of cats with CE after 4 weeks of treatment were similar to healthy control cats.

**Conclusion:** FCE cats exhibited disruptions in several metabolic pathways, some differential for IBD and SCL. Metabolic profiles partially recovered following treatment.

## 125 Abstract GI20

### 125.1 Standardized Preparation of Canine Fecal Transplant Material Does Not Alter Microbial Community Structure

#### 125.1.1 Matthew C. Salerno^1^; Nina Randolph^2^, DVM; Hannah Klein^3^; Jenessa Winston^4^, DVM, PhD, DACVIM

##### 125.1.1.1 ^1^Veterinary Student, College of Veterinary Medicine, Veterinary Clinical Sciences, The Ohio State University; ^2^Resident and PhD Student, College of Veterinary Medicine, The Ohio State University; ^3^Internal Medicine Resident, Veterinary Clinical Sciences, College of Veterinary Medicine, The Ohio State University; ^4^Assistant Professor, Veterinary Clinical Sciences, College of Veterinary Medicine, The Ohio State University

**Background:** Fecal microbiota transplantation (FMT) is the delivery of fecal material from a healthy donor into a diseased recipient to confer a health benefit. FMT is routinely delivered as a slurry via enema or nasogastric tube, but can also be encapsulated for convenient oral administration. The microbial impacts of processing feces for encapsulation are unknown.

**Hypothesis/Objectives:** We aimed to investigate if standardized fecal processing into a 10% glycerol FMT slurry alters the microbial community structure. We hypothesized that microbial community structure would not vary between processing steps.

**Animals:** Feces from four screened healthy canine fecal donors at The Ohio State University Companion Animal Fecal Bank were utilized for this study.

**Methods:** Batches of canine feces underwent a standardized FMT processing protocol. Samples from each donor, across multiple batches, were pulled from specific steps during processing and underwent DNA extraction and Illumina sequencing of the V4 region of the 16S rRNA gene. Amplicon sequence analysis was performed using MOTHUR, DADA2 and R‐Studio.

**Results:** Analysis revealed significant differences in alpha diversity (observed, Shannon index) and beta diversity (NMDS, Jaccard index) measures between donors. Within an individual donor, no significant differences were observed between processing steps (AMOVA). Sequence analysis using amplicon sequence variants (ASVs) and operational taxonomic units (OTUs) both yielded similar results.

**Conclusions and Clinical Importance:** Standardized FMT processing for encapsulation does not significantly alter the microbial composition of feces from healthy canine donors, further promoting the practicality of oral FMT administration in canine medicine. Additional studies evaluating microbial viability are required.

## 126 Abstract GI21

### 126.1 Evaluation of Intestinal Barrier Dysfunction in Dogs with Acute Hemorrhagic Diarrhea Syndrome

#### 126.1.1 **Andrea Reisinger** ^1^; Helene Stübing^1^; Patricia Ishii^2^; Jan Suchodolski^2^; Stefan Unterer^3^; Jörg Steiner^2^; Jonathan Lidbury^2^; Katrin Hartmann^1^; Kathrin Busch^1^

##### 126.1.1.1 ^1^Clinic of Small Animal Medicine, Centre for Clinical Veterinary Medicine, LMU Munich, Munich, Germany; ^2^Gastrointestinal Laboratory, Department of Small Animal Clinical Sciences, Texas A&M University, College Station, TX, USA; ^3^Clinic for Small Animal Internal Medicine, Vetsuisse Faculty, University of Zurich, Zurich, Switzerland

**Background:** Acute hemorrhagic diarrhea syndrome (AHDS) is characterized by the occurrence of acute bloody diarrhea. Histopathologic examination of intestinal biopsies from dogs with AHDS typically reveals necrotizing enteritis. Therefore, the presence of intestinal barrier dysfunction is suspected, which might be associated with clinical severity.

**Hypothesis:** Dogs with AHDS have increased intestinal permeability, which is associated with clinical severity.

**Animals:** Thirty client‐owned dogs with AHDS and 25 healthy control dogs (HC).

**Methods:** Prospective clinical trial. The AHDS‐index was evaluated in all dogs at the day of presentation. On the same day, dogs were administered 2 ml/kg of iohexol orally. After two hours, serum was obtained to measure iohexol concentration (SIC) by enzyme‐linked immunosorbent assay. The same procedure was performed in the HC group. A Mann‐Whitney test was used to compare SIC between AHDS and HC. The association between the AHDS‐index and SIC was evaluated using Spearman's rank correlation.

**Results:** SIC were significantly higher (p=0.002) in AHDS (median: 46 μg/ml; min–max: 9–246) compared to HC (30 μg/ml; 11–57). There was a significant positive correlation between AHDS‐index and SIC (r_s_=0.4; p=0.020). Furthermore, SIC was significantly higher (p=0.001) in dogs with severe (AHDS‐index ≥9) (median: 80 μg/ml; min–max: 25–246) compared to dogs with mild to moderate disease (AHDS‐index <9) (31 μg/ml; 9–54).

**Conclusions and Clinical Importance:** Dogs with AHDS have significantly increased intestinal permeability as assessed by iohexol. In addition, a significant correlation between clinical severity and permeability was detected.

## 127 Abstract GI22

### 127.1 Intestinal S100/Calgranulin Expression in Cats with Chronic Enteropathies

#### 127.1.1 **Denise S. Riggers** ^1^; Corinne Gurtner^2^, DrMedVet, DECVP, FVH Pathology; Martina Protschka^3^, Dr; Denny Böttcher^4^, DrMedVet, Fachtierarzt für Pathologie; Wolf von Bomhard^5^, DrMedVet, Fachtierarzt für Pathologie, DECVP; Gottfried Alber^6^, Professor, DrMedVet; Karsten Winter^7^, DrRerHum; Johannes Seeger^8^, Professor, DrMedVet, Fachtierarzt für Anatomie; Joerg Steiner^9^, DrMedVet, PhD, DACVIM, DECVIM‐CA, AGAF; Romy Heilmann^10^, Professor, DrMedVet, DACVIM (SAIM), DECVIM‐CA, MANZCVS, PhD

##### 127.1.1.1 ^1^Department for Small Animals, College of Veterinary Medicine, University of Leipzig, Leipzig, Germany; ^2^Clinical Instructor Pathology, Institute of Animal Pathology, Vetsuisse Faculty, University of Bern, Switzerland; ^3^Research Scientist, Institute of Immunology/Molecular Pathogenesis, Center for Biotechnology and Biomedicine, College of Veterinary Medicine, University of Leipzig, Leipzig, Germany; ^4^Clinical Scientist, Institute of Pathology, Faculty of Veterinary Medicine, University of Leipzig, Leipzig, Germany; ^5^Laboratory for Veterinary Pathology, Munich, Germany; ^6^Professor, Veterinary Immunology, Institute of Immunology/Molecular Pathogenesis, Center for Biotechnology and Biomedicine, College of Veterinary Medicine, University of Leipzig, Leipzig, Germany; ^7^Institute of Anatomy, College of Medicine, Leipzig University, Leipzig, Germany; ^8^Professor, Histology and Embryology, Institute of Veterinary Anatomy, College of Veterinary Medicine, University of Leipzig, Leipzig, Germany; ^9^Professor, Small Animal Internal Medicine; Director, Gastrointestinal Laboratory, Department of Small Animal Clinical Sciences, College of Veterinary Medicine and Biomedical Sciences, Texas A&M University; ^10^Professor, Small Animal Internal Medicine, Department for Small Animals, College of Veterinary Medicine, University of Leipzig, Leipzig, Germany

**Background:** Diagnosis of chronic inflammatory enteropathies (CIE) in cats and differentiation from intestinal lymphoma using currently available diagnostics is challenging. The S100/calgranulins appear as useful non‐invasive biomarkers for canine CIE, with increased intestinal expression, but have not been evaluated in cats.

**Hypothesis:** We hypothesized that the S100/calgranulins are differentially expressed between feline CIE and intestinal lymphoma, and correlate with histological and/or clinical disease severity.

**Animals:** Retrospective case‐control study including patient data and gastrointestinal tissue from 16 cats with CIE and 8 with intestinal lymphoma.

**Methods:** Gastrointestinal tissue biopsies were immunohistochemically stained using species‐specific polyclonal α‐S100A8/A9 and α‐S100A12 antibodies. Epithelial and lamina propria S100A8/A9^+^ and S100A12^+^ cells per 10,000 μm^2^ were compared between groups of cats and tested for a correlation with clinicopathologic abnormalities, clinical disease severity (feline chronic enteropathy activity index [FCEAI] score), and histologic lesions.

**Results:** S100A8/A9^+^ and S100A12^+^ cells were detected in all gastrointestinal segments, without significant differences between CIE and lymphoma. Segmental inflammatory lesions were moderately to strongly correlated with increased S100/calgranulin‐positive cell counts. Increased ileal S100A12^+^ cell counts correlated with hypocobalaminemia (*P*=0.0229). Hypofolatemia was linked to higher duodenal S100A8/A9^+^ cell counts. FCEAI scores were higher in cats with lymphoma than CIE (*P*=0.0101) and were linked to higher duodenal S100A12^+^ cell counts (*P*=0.0385).

**Conclusions and Clinical Importance:** These findings suggest a role of the S100/calgranulins in the pathogenesis of the spectrum of feline chronic enteropathies and emphasize the diagnostic value of B vitamin determination and clinical disease grading.

## 128 Abstract GI23

### 128.1 Placebo‐Controlled Trial of Hydrolyzed Fish Diets in Dogs with Chronic Enteropathy

#### 128.1.1 **Kenneth Simpson**; Meredith Miller; John Loftus; Mark Rishniw; Carol Frederick; Joseph Wakshlag

##### 128.1.1.1 Cornell University

**Background:** Dietary modification can induce clinical remission in dogs with chronic enteropathy. Palatability impacts dietary therapy in dogs with protein losing enteropathy (PLE).

**Objectives:** To compare the ability of isocaloric diets composed of (A) hydrolyzed fish, rice starch and fish oil (HF), (B) HF plus prebiotics, turmeric and high cobalamin (HF+), or (C) highly digestible non‐hydrolyzed mixed protein source diet (Placebo) to resolve clinical signs and maintain serum B12 in dogs with chronic enteropathy. To evaluate palatability and weight gain in dogs with PLE.

**Animals:** Thirty‐one client owned dogs with chronic enteropathy: 23 non‐PLE, 8 PLE.

**Methods:** Randomized, blinded, placebo‐controlled clinical trial. Each diet was fed for 2 wks, with responders continuing for 12 wks. Non‐responders crossed over to another diet for 12 wks. Clinical response was determined by CCEAI and stool consistency, with long term follow‐up at 26 wks. Concurrent medications were allowed in PLE.

**Results:** Nineteen of 23 dogs with non‐PLE chronic enteropathy responded solely to their initial diet, with no difference between diets (P<0.05. Table 1). Four dogs crossed over, with sustained remission in 18/18 at 6 months (Table 1). Serum B12 was maintained by diet in chronic enteropathy. Dogs with PLE fed HF or HF+ found it palatable and gained weight, with 2/8 responding to diet alone. **Table 1**

**Figure:**
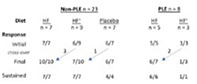
Dogs with chronic enteropathy

**Conclusions and Clinical Importance:** Changing diet, independent of antigen restriction or supplementation, was associated with long‐term clinical remission in dogs with chronic enteropathy, and a subset of PLE. Further study is required to determine the basis of this clinical response.

## 129 SMALL ANIMAL INTERNAL MEDICINE ‐ HEMATOLOGY

## 130 Abstract HM01

### 130.1 A Novel Flow Cytometric Assay to Assess Platelet Desialylation in Canine Immune Thrombocytopenia

#### 130.1.1 **Michael Barchilon** ^1^; Kyle Krellwitz^2^; Marjory Brooks^3^; Austin Viall^2^; Dana LeVine^4^

##### 130.1.1.1 ^1^Iowa State University; ^2^Veterinary Pathology, Iowa State University; ^3^Population Medicine and Diagnostic Sciences, Cornell University; ^4^Clinical Sciences, Auburn University

**Background:** Immune thrombocytopenia (ITP) is a common hemostatic disorder of dogs leading to significant morbidity and mortality, often from side effects of immunosuppression. In ITP, autoantibodies target platelets for macrophage clearance. Human studies demonstrate autoantibodies also trigger platelet desialylation. Desialylation marks platelets for clearance, thus preventing desialylation could lead to new, safer treatment strategies. Studying desialylation in canine ITP requires a platelet desialylation assay.

**Objective:** Develop a flow cytometric assay to detect exposed platelet β‐galactose residues as a marker of desialylation based on binding of FITC‐labeled *Ricinus communis* agglutinin (RCA).

**Animals:** 17 healthy, client‐owned dogs recruited from a veterinary teaching hospital.

**Methods:** Platelet‐rich plasma (PRP) was isolated from EDTA‐anticoagulated blood. *Ex vivo* desialylated platelets were generated by neuraminidase C treatment (positive control); β‐lactose incubated PRP served as negative control. Controls and native PRP were labeled with RCA‐FITC and desialylation was quantified flow cytometrically by mean fluorescence intensity/forward scatter (MFI/FSC). Following optimization, analytic precision, performance in serially diluted PRP, and sample stability were assessed.

**Results:** RCA MFI/FSC remained stable for 48 hr post‐blood collection at 25°C (p=0.06) and 4°C (p=0.24). Precision was acceptable with coefficient of variation=4.8%. RCA MFI/FSC was not influenced by platelet counts ranging from 200,000–10,000 platelets/μL (p=0.14).

**Conclusions and Clinical Importance:** This flow cytometric assay detects desialylated platelets with good analytic performance through a wide range of platelet counts. This assay will enable pathophysiologic investigations of platelet desialylation in canine ITP and set the stage for novel therapeutics.

## 131 Abstract HM02

### 131.1 Efficacy of Therapeutic Plasma Exchange (TPE) in Dogs with Immune‐mediated Hemolytic Anemia: A Case‐Controlled Study

#### 131.1.1 **Larry D. Cowgill** ^1^; Sean Naylor^2^; Carrie Palm^3^, DVM, DACVIM (SAIM), MS; John Kirby^2^

##### 131.1.1.1 ^1^School of Veterinary Medicine, University of California‐Davis, Davis, CA, USA; ^2^Technician, Veterinary Medical Teaching Hospital, School of Veterinary Medicine, University of California; ^3^Associate Professor, Medicine and Epidemiology, School of Veterinary Medicine, University of California

**Background:** TPE has emerged as adjunctive therapy for severe IMHA in dogs but has not been compared to conventional therapy. We report the efficacy of adjunctive TPE to comparator dogs managed medically.

**Hypothesis/Objectives:** Effective TPE delivery improved survival in dogs with severe IMHA.

**Animals:** 34 client‐owned dogs with primary or secondary IMHA (HCT <20%, spherocytosis, reticulocytosis, transfusion dependency, known outcome) treated with adjunctive TPE at UC Davis between 2010–2020 were compared to 72 similarly affected, randomly selected dogs managed medically over the same interval. Both cohorts were censored for euthanasia within 3 days for financial concerns yielding 32 TPE and 68 Control dogs.

**Methods:** 100 TPE treatment were delivered on a Spectra Optia. Controls were managed with conventional immunosuppression. Kaplan‐Meier (Logrank) survival analysis was compared between all TPE, dogs with ≥3TPE treatments, and Controls at 30 days.

**Results:** TPE dose was 1.4±0.3 plasma volumes (PV)/session. 4/6 non‐surviving dogs received ≤2 TPE treatments (2.0±0.6 PV); 28 dogs received ≥3TPE treatments delivering 4.2±0.6 PV/dog. 30‐day survival for all TPE and medically managed dogs was 76.5% and 57.5%, respectively (*P*=0.06). When censored for financial‐directed euthanasia, survival for TPE and Control cohorts was 81.3% vs 61.8%, respectively (*P*=0.058, Fig). Survival for dog receiving ≥3TPE (>4.0 PV delivered) was 92.9% vs Control (*P*=0.003). Hazard ratio for Control/≥3TPE was 6.4.

**Figure:**
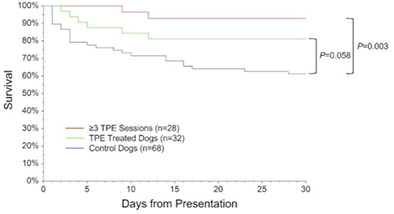
Figure 1

**Conclusions:** Delivery of TPE (>4 total PV) was associated with improved survival in dogs with IMHA compared to medical management.

## 132 Abstract HM03

### 132.1 Validation and Reference Interval of Activated Clotting Time in Dogs Using a Point‐of‐Care Analyzer

#### 132.1.1 **Arnaut Hellemans** ^1^; Nausikaa Devriendt^2^, DVM, DECVS, PhD; Filip De Somer^3^, PhD; Sofie Marynissen^2^, DVM, DECVIM; Sylvie Daminet^2^, DVM, DECVIM, PhD; Dominique Paepe^2^, DVM, DECVIM, PhD; Pascale Smets^2^, DVM, DECVIM, PhD

##### 132.1.1.1 ^1^Ghent University; ^2^Small Animal Department, Ghent University; ^3^Faculty of Medicine and Health Sciences, Heart Centre, Ghent University

**Background:** Activated clotting times (ACT) are measured to titrate anticoagulant dose during intravascular procedures. Point‐of‐care (POC) analyzers could improve test practicality and accuracy compared to manual tests, but a reference interval (RI) for dogs is lacking.

**Objectives:** To establish a RI for ACT in adult dogs using a POC analyzer, to quantify longitudinal intra‐subject variability and to investigate device reliability and inter‐device agreement.

**Animals:** Forty‐two healthy dogs, including 28 client‐owned and 14 research dogs.

**Methods:** Prospective measurements were performed on fresh venous blood samples using the i‐STAT 1 (Abbott) POC analyzer and kaolin cartridges. The RI was determined using the Robust method. Intra‐subject within‐ and between‐day variability were quantified between baseline and 2 hour (n=8) or 48 hour (n=10). Device reliability and inter‐device agreement were studied by duplicate measurements (n=12) on identical devices.

**Results:** Mean, lower and upper reference limits for ACT were 92.9±9.1 seconds, 74.4 seconds (90% confidence interval (CI): 70.1–78.3 seconds) and 111.2 seconds (90% CI: 107.5–115.2 seconds), respectively. Coefficients of variation of intra‐subject within‐day (8.1%) and between‐day (10.4%) variability were low, yet resulted in a small but significant between‐day difference (P=0.020). Device reliability assessed by intraclass correlation coefficient was excellent (0.99, 95% CI 0.997–1.000) and measurements between devices were not significantly different (P=0.593).

**Conclusions and Clinical Importance:** The POC analyzer has an excellent reliability and test simplicity, which favors its use over manual ACT assays.

## 133 Abstract HM04

### 133.1 Investigation of Novel Hemostasis Parameters and Closure Curves on the Platelet Function Analyzer‐200 in Cats

#### 133.1.1 **Matthew Kornya**; Anthony Abrams‐Ogg, DACVIM (SAIM); Shauna Blois, DACVIM (SAIM); Shari Raheb, DACVIM (Cardiology); Darren Wood, DACVP (Clinical)

##### 133.1.1.1 Ontario Veterinary College

**Background:** The previously undescribed Primary Hemostasis Components (PHC) 1 and 2 on the PFA‐200 analyzer are for additional variables that normalize platelet function to a transferable, intuitive, percentage‐based system. Closure curves may also be visually inspected and may predict closure vs non‐closure in animals with flow obstruction.

**Objectives/Hypothesis:** To investigate the PHC parameters and curve analysis for determination of platelet function in cats.

**Animals:** 20 prospective healthy cats between 2–10y with normal physical examination, CBC and biochemistry; 34 healthy retrospective blood donor cats, and 14 retrospective cats receiving therapy with clopidogrel.

**Methods:** Normal ranges were determined for CT, PHC(1), and PHC(2). ROC curves were created to determine utility of these values to assess clopidogrel effect. Visual analysis of the closure curves was performed.

**Results:** There was no difference in CT for healthy cats and blood donors. Reference interval for CT was 20–90s (COL/ADP) and 36–119s (P2Y). RI for PHC(1) was 51.1s–145.1s (COL/ADP) and 41.5s–165.6s(P2Y). RI for PHC(2) was 94.2s–105.9s (COL/ADP) and 87.2s–105.2s(P2Y).

AUCROCs for clopidogrel effect were excellent with no difference between tests. Optimal cut‐offs for clopidogrel response were >76s (COL/ADP CT), >211s (P2Y CT); <74.1s (COL/ADP PHC(1)), <72.1s (P2Y PHC(1)); and <96.6s (COL/ADP PHC(2)),<76.0s (P2Y PHC(2)).

Six classes of closure curve were identified, of which two were associated with non‐closure.

**Conclusions and Clinical Importance:** PHC(1) and PHC(2) perform comparably to CT and provide an expression of platelet function that may be compared between cartridges, and laboratories. Closure curves may be used to predict non‐closure.

## 134 Abstract HM05

### 134.1 Validation of Shipping of Feline Blood Samples for Analysis on the Platelet Function Analyzer 200

#### 134.1.1 **Matthew Kornya**; Anthony Abrams‐Ogg, DACVIM (SAIM); Shauna Blois, DACVIM (SAIM); Shari Raheb, DACVIM (Cardiology); Darren Wood, DACVP (Clinical)

##### 134.1.1.1 Ontario Veterinary College

**Background:** The Platelet Function Analyzer‐200 (PFA‐200) allows determination of clopidogrel effect in cats, however, samples must be analyzed within four hours of collection making routine monitoring infeasible. Samples in dogs and pilot data in cats have demonstrated feasible analysis after shipping and storage.

**Objectives/Hypothesis:** To validate the shipment of feline blood samples for analysis on the PFA‐200 24h after collection.

**Animals:** 20 healthy cats between 2–10y with normal physical examination, CBC and biochemistry, and four cats receiving clopidogrel therapy.

**Methods:** Blood was collected by jugular venipuncture. A CBC was analyzed at point‐of‐care to confirm adequate platelet counts. Samples were divided and analyzed in duplicate at point of‐care with the COL/ADP and P2Y cartridges; and packaged in insulated containers with ice packs, transported to a distant location and then back the following day, and analyzed 24h after initial collection. Results of point‐of‐care and shipped samples were compared.

**Results:** When used to categorize cats as clopidogrel responsive vs resistant, agreement was 0.85 (COL/ADP) and 0.95 (P2Y). Bland‐Altman analysis showed minimal outliers and no bias. Median closure time was significantly different between point‐of‐care (COL/ADP 51.5s, P2Y 50.5s) and shipped samples (COL/ADP 78.75s, P2Y 65.5s) (p<0.001). The occurrence of flow obstructions was not significantly different between groups (P=0.525).

**Conclusions and Clinical Importance:** Feline blood may be shipped and analyzed up to 24h after collection with the PFA‐200 with comparable results to point‐of‐care testing. This allows practical, remote determination of clopidogrel effect in cats.

## 135 Abstract HM06

### 135.1 Validation of the ProCyte Dx and Visual Slide Review with the Plateletworks System in Cats

#### 135.1.1 **Matthew Kornya**; Anthony Abrams‐Ogg, DACVIM (SAIM); Shauna Blois, DACVIM (SAIM); Shari Raheb, DACVIM (Cardiology); Darren Wood, DACVP (Clinical)

##### 135.1.1.1 Ontario Veterinary College

**Background:** Determining platelet function in cats may be useful for monitoring clopidogrel effect. Plateletworks (PW) exposes citrated blood to a platelet agonist, and determines “percent aggregation” based on platelet counts obtained with a hematology analyzer. PW was developed for impedance analyzers and has been validated for the Siemens ADVIA 2120 optical analyzer, but not for point‐of‐care optical analyzers.

**Objectives/Hypothesis:** To validate Plateletworks for the IDEXX ProCyte Dx analyzer and visual slide review.

**Animals:** 20 healthy cats between 2–10y and 5 cats receiving clopidogrel therapy.

**Methods:** Jugular blood samples were aliquoted into ADP agonist and EDTA tubes, divided, and analyzed simultaneously on ADVIA 2100 and ProCyte Dx analyzers at 10min, 30min, 45min, 1h. Slides were prepared and degree of clumping scored by a blinded observer. Percent aggregation was the primary outcome.

**Results:** Association of aggregation was 0.8166 between analyzers. Median values were significantly different (p=0.0004), but Bland‐Altman analysis showed minimal outliers and no bias. Maximal coefficient of variation was lower for ProCyte Dx than ADVIA 2120. When classifying as non‐, low‐ or high‐aggregators, Cohen's Kappa was 0.714. Correlation was substantial between both analyzers and slide review. There was no significant change in aggregation within the first hour, however there was a trend to increase at 30 min, followed by a slight decline. Cats on clopidogrel were categorized as responsive on both analyzers.

**Conclusions and Clinical Importance:** Plateletworks may be used in clinics with ProCyte Dx analyzers or visual slide review. The timespan for analysis is longer than previously reported.

## 136 Abstract HM07

### 136.1 Evaluation of the Therapeutic Efficacy of Splenectomy in 20 Dogs with Non‐regenerative Immune‐mediated Anemia

#### 136.1.1 **Keitaro Morishita** ^1^; Mei Sugawara^1^; Junpei Yamazaki^1^; Sangho Kim^1^; Kenji Hosoya^1^; Noboru Sasaki^1^; Kensuke Nakamura^1^; Hiroshi Ohta^2^; Mitsuyoshi Takiguchi^1^

##### 136.1.1.1 ^1^Hokkaido University; ^2^Rakuno Gakuen University

**Background:** To our knowledge, there are no reports summarizing the therapeutic effects of splenectomy in dogs with non‐regenerative immune‐mediated anemia (NRIMA).

**Hypothesis/Objectives:** Evaluation of the therapeutic efficacy of splenectomy as an alternative to immunosuppressive therapy.

**Animals:** Twenty client‐owned dogs with NRIMA who had splenectomies performed at Hokkaido University Veterinary Teaching Hospital between 2015 and 2021.

**Methods:** Retrospective case series.

**Results:** The median age of the included dogs was 11.5 years (range 3–15 years). The reasons for splenectomy were:

5 No response to immunosuppressive therapy (n=13)
6 Temporary response to immunosuppressive therapy but subsequent loss of response (n=5)
7 Suspected complications of immunosuppressive therapy (n=1)
8 First‐line treatment (n=1)

Nineteen dogs were treated with immunosuppressive therapy prior to splenectomy, and the median duration of treatment was 113 days (range 50–295 days). Two dogs died within 7 days of surgery, and the remaining 18 dogs showed an increase in reticulocyte count at a median of 16 days (range 1–49 days) after splenectomy, with a median maximum reticulocyte count of 137,900/μl (range 71,700–368,200/μl). During the study period, seven dogs had complete remission of anemia, eight dogs had persistent mild to moderate anemia but could be maintained without blood transfusion, and three dogs had worsening non‐regenerative anemia that required therapeutic intervention.

**Conclusions and Clinical Importance:** Fifteen out of 20 dogs (75%) improved after splenectomy and decreased their dependency on transfusions, suggesting that splenectomy is an effective treatment for NRIMA.

## 137 Abstract HM08

### 137.1 Assessing Methods to Monitor Rivaroxaban Therapy in Hypercoagulable Dogs

#### 137.1.1 **Erin M. Phillips** ^1^; Anthony Abrams‐Ogg^1^, DACVIM (SAIM), PhD; Shauna Blois^1^, DACVIM (SAIM), PhD; Benoit Cuq^2^, DACVIM (SAIM), MRCVS; Darren Wood^1^, PhD

##### 137.1.1.1 ^1^Ontario Veterinary College; ^2^University College Dublin

**Background:** Measurement of rivaroxaban‐specific anti‐Xa activity is recommended for monitoring rivaroxaban efficacy in veterinary medicine, but this assay is performed only at specialized reference laboratories. Accurate detection of rivaroxaban effect using other hemostatic tests would make therapeutic monitoring more timely and accessible.

**Objective:** To assess the results of rivaroxaban‐specific anti‐Xa activity with prothrombin time (PT), partial thromboplastin time (PTT), fibrinogen concentration, tissue‐factor and kaolin‐activated thromboelastography (TEG) and thrombin generation (TG) activity in hypercoagulable dogs.

**Animals:** 9 client‐owned dogs diagnosed with hypercoagulability and/or thromboembolic disease and prescribed rivaroxaban, recruited from the OVC‐HSC population from August 2020 to November 2021.

**Methods:** Prospective clinical trial. Blood samples were collected by direct jugular venipuncture prior to treatment, then 1‐week and 1 to 3‐months after initiation of therapy. PT, PTT, fibrinogen, anti‐Xa activity, TEG, and TG were performed at each visit (3 hours after rivaroxaban dosing).

**Results:** The average rivaroxaban dose was 1.4 mg/kg/day. There was a strong correlation between the anti‐Xa assay and PT (r=0.806, p=0.0002). Anti‐Xa was strongly positively correlated with TG variables ttpeak (r=0.819, P=0.0001) and lag time (r=0.746, P=0.0009), and negatively correlated with TG variables ETP (r=‐0.568, P=0.022) and peak (r=‐0.706, P=0.002). There was a poor correlation between anti‐Xa assay and PTT, fibrinogen, and all TEG variables.

**Conclusion and Clinical Importance:** Prothrombin time is strongly correlated with rivaroxaban‐specific anti‐Xa activity and may be a convenient method to monitor dogs receiving rivaroxaban therapy.

## 138 Abstract HM09

### 138.1 Effect of Antioxidant Supplementation on Oxidative and Storage Lesions in Canine Packed Red Blood Cells

#### 138.1.1 **Janet L. Roque‐Torres** ^1^; Andrew Woolcock^2^, DVM, DACVIM; Andrea Santos^3^, DVM, MSc, PhD, DACVP; George Moore^4^, DVM, PhD, DACVIM, DACVPM

##### 138.1.1.1 ^1^College of Veterinary Medicine, Purdue University; ^2^Associate Professor, Small Animal Internal Medicine, Veterinary Clinical Services, College of Veterinary Medicine, Purdue University; ^3^Assistant Professor of Veterinary Clinical Pathology, Comparative Pathobiology, College of Veterinary Medicine, Purdue University; ^4^Director, Clinical Trials and Department of Epidemiology, Veterinary Administration, College of Veterinary Medicine, Purdue University

**Background:** Storage of canine packed red blood cells (pRBC) leads to time‐dependent lesions which may increase risk of transfusion reactions. Oxidative stress is a contributor of storage lesions, but supplementation of antioxidants in canine pRBC has not been investigated.

**Objective:** Describe storage and oxidative lesions in canine pRBC during routine storage when supplemented with saline (control, Group 1), N‐acetylcysteine (NAC) and ascorbic acid (AA; Group 2), and AA and a‐tocopherol (VE; Group 3)

**Hypothesis:** Storage and oxidative lesions in canine pRBC [measured by glutathione (GSH), thiobarbituric acid reactive substances, and flow cytometry for intraerythrocytic reactive oxygen species (ROS)] will be decreased with antioxidant supplementation.

**Methods: N**ine leukoreduced units of canine pRBC were aseptically separated into three aliquots (Groups 1, 2, 3). Antioxidants were supplemented on Day 1 after baseline samples collected. Additional samples were collected on days 7, 28, and 42. Units were collected in 3 batches, with assays performed at the end of each storage period. Type 3 tests of fixed effects compared the impact of group and time on each measurement.

**Results:** All groups showed storage lesions and GSH depletion by day 42 compared to baseline, regardless of antioxidant supplementation. Intraerythrocytic ROS accumulation was lower in Group 3 (AA & VE) compared to other groups at all time points after baseline (p<0.0001).

**Conclusions and Clinical Importance:** Supplementation of canine pRBC with AA and VE reduced oxidative stress but not storage lesions. Future studies should evaluate the clinical use and incidence of transfusion reaction with AA/VE‐supplemented pRBC.

## 139 Abstract HM10

### 139.1 Retrospective Analysis of Immunosuppressive and Anti‐Thrombotic Protocols in Canine Non‐associative Immune‐Mediated Hemolytic Anemia

#### 139.1.1 **Harry Cridge** ^1^; Jennifer Weng^2^, DVM; Haley Abbott^3^; Jose Mix^3^; Nyssa Levy^4^, MS, DVM, DACVECC; Robert Wills^5^, DVM, PhD, DACVPM (Epidemiology); Andrew Mackin^5^, BVMS, DVSc, FANZCVSc, DACVIM (SAIM); John Thomason^6^, DVM, MS, DACVIM (SAIM)

##### 139.1.1.1 ^1^Michigan State University; ^2^Rotating Intern, College of Veterinary Medicine, Michigan State University; ^3^Veterinary Student, College of Veterinary Medicine, Michigan State University; ^4^Assistant Professor, College of Veterinary Medicine, Michigan State University; ^5^Professor, College of Veterinary Medicine, Mississippi State University; ^6^Associate Professor, College of Veterinary Medicine, Mississippi State University

**Background:** Evidence supporting different therapeutic protocols for non‐associative immune‐mediated hemolytic anemia (na‐IMHA) is weak.

**Hypothesis/Objectives:** Investigate the impact of drug selection on outcome in na‐IMHA.

**Animals:** 242 client‐owned dogs.

**Methods:** Multi‐institutional retrospective study (2015–2020). Immunosuppressive efficacy was determined by time to PCV stabilization, duration of hospitalization, and disease relapse and mortality rates. Anti‐thrombotic efficacy was determined by presence of clinically suspected or confirmed thrombosis. Data was analyzed using mixed model linear and logistic regression.

**Results:** Use of corticosteroids alone versus a multi‐agent protocol had no significant effect on time to PCV stabilization (p=0.55), duration of hospitalization (p=0.13), or mortality (p=0.58): mortality with 1 immunosuppressive drug was 17.6%, and with 2 drugs was 28.3%. A higher rate of relapse (p=0.04, OR: 3.97) during follow‐up (median 31 days) was detected in dogs receiving a corticosteroid alone (11.3%) compared to multiple agents (3.1%). When comparing specific drug protocols, there was no significant effect on time to PCV stabilization (p=0.31), relapse (p=0.44), or mortality (p=0.08). Duration of hospitalization was significantly longer, by 1.8 days, for the corticosteroid with mycophenolate group (p=0.02). Use of clopidogrel versus a multi‐agent protocol had no effect on development of confirmed (p=0.41) or suspected (p=0.37) thromboses.

**Conclusions and Clinical Importance:** Corticosteroids alone may be suitable for the initial management of na‐IMHA, although an increased risk of relapse may be noted. Use of multiple anti‐thrombotic agents does not reduce risk of thrombosis.

## 140 Abstract HM11

### 140.1 Differences in Hematological Parameters Between Dogs with Congenital Intrahepatic and Extrahepatic Portosystemic Shunts

#### 140.1.1 **Yishan Kuo** ^1^; Stacie Summers^2^, DVM, PhD, DACVIM

##### 140.1.1.1 ^1^Oregon State University; ^2^Assistant Professor, Carlson College of Veterinary Medicine, Oregon State University

**Background:** Dogs with congenital portosystemic shunts commonly have hematological abnormalities. Unlike dogs with extrahepatic portosystemic shunt (EHPSS), dogs with intrahepatic portosystemic shunt (IHPSS) are predisposed to gastrointestinal ulceration and bleeding.

**Objective:** To compare hematological parameters between dogs with IHPSS and EHPSS.

**Animals:** Thirty‐two dogs with EHPSS and 23 dogs with IHPSS.

**Methods:** Retrospective study of client‐owned dogs with a confirmed congenital portosystemic shunt. Erythrogram parameters and history of blood transfusion prior to shunt closure were recorded. An unpaired Student t‐test was used to compare values between dogs with IHPSS and EHPSS.

**Results:** Twelve IHPSS dogs (52%) and 3 EHPSS dogs (9%) had a hematocrit below the reference interval (<37%). Two IHPSS dogs and no EHPSS dogs had a history of a blood transfusion. Compared to EHPSS dogs, IHPSS dogs had a significantly lower hemoglobin (P=0.0004; mean difference +/‐ SEM: 2.4 +/‐ 0.6 g/dL), hematocrit (P=0.004; 5.1 +/‐ 1.7%), mean cell volume (P<0.0001; 8.3 +/‐ 2.0 fL), mean cell hemoglobin concentration (P=0.001; 1.9 +/‐ 0.5 g/dL), and plasma protein (P=0.04; 0.4 +/‐ 0.01 g/dL). Reticulocyte count was measured in 16 IHPSS dogs and 16 EHPSS dogs, and no significant difference was found between groups (P=0.7).

**Conclusions:** IHPSS dogs had lower hematological values than EHPSS dogs. In a few IHPSS dogs, a blood transfusion was required for treatment of anemia. These differences may be partly attributable to blood loss secondary to gastrointestinal bleeding in dogs with IHPSS.

## 141 Abstract HM12

### 141.1 Evaluation of Thrombin Generation as a Novel Antiplatelet Therapeutic Monitoring Tool in Dogs Administered Clopidogrel

#### 141.1.1 **Kaitlyn Rank** ^1^; Alex Lynch^2^, BVSc (Hons) DACVECC MRCVS; Laura Ruterbories^3^, BS, RVT, RLATG, VTS (LAM); Ronald H. Li^4^, DVM PhD DACVECC; Yu Ueda^5^, DVM PhD DACVECC

##### 141.1.1.1 ^1^North Carolina State University; ^2^Assistant Professor in Emergency and Critical Care, Department of Clinical Sciences, North Carolina State University; ^3^Research Specialist, Veterinary Hemostasis and Neuro‐Oncology, Department of Clinical Sciences, North Carolina State University; ^4^Assistant Professor in Emergency and Critical Care, Surgical and Radiological Sciences, North Carolina State University; ^5^Clinical Assistant Professor in Emergency and Critical Care, Department of Clinical Sciences, North Carolina State University

**Background:** The platelet inhibitory effect of clopidogrel can vary between individuals. A proprietary modified thromboelastography protocol (TEG Platelet Mapping [TEG‐PM]) can be used for therapeutic monitoring but is not widely available. Thrombin generation (TG) might offer a viable monitoring alternative.

**Hypothesis/Objectives:** Evaluate TG for antiplatelet monitoring in dogs administered clopidogrel.

**Animals:** 6 healthy mix‐breed dogs.

**Methods:** 2 mg/kg clopidogrel was administered orally once daily for 7 days. Blood was collected for TEG‐PM and TG on day 0 and day 7. Platelet inhibitory effect was assessed by percentage platelet inhibition (% inhibition) on TEG‐PM and with the TG variables lag time, peak, and endogenous thrombin potential. Comparisons between day 0 and day 7 results were made using a paired student's t‐test. Data are presented as mean +/‐ SD.

**Results:** Significant changes in % inhibition were noted on TEG‐PM [day 0: 57.7 +/‐ 27.3%, day 7: 98.6 +/‐ 4.0%, P=0.02)], but without significant changes in TG variables: lag time (day 0: 1.8 +/‐ 0.2 min, day 7: 1.8 +/‐ 0.2 minutes, P=0.42); peak (day 0: 76.4 +/‐ 6.9 nM, day 7: 72.7 +/‐ 10.4 nM, P=0.49); and endogenous thrombin potential (day 0: 399.2 +/‐ 26.9, day 7: 392.2 +/‐ 32.4, P=0.49).

**Conclusions and Clinical Importance:** No significant changes in thrombin generation variables were detected in healthy dogs administered clopidogrel, despite concomitant evidence of platelet inhibition with TEG Platelet Mapping. Thrombin generation does not appear to be a useful antiplatelet therapeutic monitoring tool in dogs administered clopidogrel.

## 142 Abstract HM13

### 142.1 Comprehensive Protein and Gene Expression Analysis of Spleen from Dogs with Non‐regenerative Immune‐Mediated Anemia

#### 142.1.1 **Mei Sugawara‐Suda** ^1^; Jumpei Yamazaki^2^, DVM, PhD; Keitaro Morishita^3^, DVM, AiCVIM, PhD; Kenyu Imai^1^; Sangho Kim^4^, DVM, PhD; Kenji Hosoya^5^, DVM, PhD, ACVR, ACVIM, JCVS, AiCVIM; Noboru Sasaki^6^, DVM, PhD; Kensuke Nakamura^7^, DVM, PhD, AiCVIM; Mitsuyoshi Takiguchi^8^, DVM, PhD, AiCVIM

##### 142.1.1.1 ^1^Hokkaido University; ^2^Specially Appointed Associate Professor, Translational Research Unit, Veterinary Teaching Hospital, Graduate School of Veterinary Medicine, Hokkaido University; ^3^Assistant Professor, Veterinary Teaching Hospital, Graduate School of Veterinary Medicine, Hokkaido University; ^4^Assistant Professor, Laboratory of Veterinary Surgery, Graduate School of Veterinary Medicine, Hokkaido University; ^5^Associate Professor, Laboratory of Advanced Veterinary Medicine, Graduate School of Veterinary Medicine, Hokkaido University; ^6^Assistant Professor, Laboratory of Veterinary Internal Medicine, Department of Clinical Sciences, Faculty of Veterinary Medicine, Hokkaido University; ^7^Associate Professor, Laboratory of Veterinary Internal Medicine, Department of Clinical Sciences, Faculty of Veterinary Medicine, Hokkaido University; ^8^Professor, Laboratory of Veterinary Internal Medicine, Department of Clinical Sciences, Faculty of Veterinary Medicine, Hokkaido University

**Background:** Some non‐regenerative immune‐mediated anemia (NRIMA) patients resist immunosuppressive therapy. Our preliminary study indicated that splenectomy improved non‐regenerative anemia for these cases.

**Hypothesis/Objectives:** The spleen releases humoral factors that suppress hematopoiesis leading to non‐regenerative anemia.

**Animals:** Fifteen dogs with NRIMA and three healthy control dogs.

**Methods:** Protein expression and gene expression were analyzed. Four serum samples pre‐ and post‐splenectomy were used in liquid chromatography with tandem mass spectrometry. Protein identification and quantification were performed by Scaffold DIA and Prosit (database UP000002254). Differentially expressed proteins were defined using paired t‐tests with p‐value <0.05 and fold change >1.5 or <0.7. RNA was extracted and subjected to RNA sequencing (Illumina NextSeq 500). Sequencing reads (1M single‐Read/sample) were aligned to the canine reference genome assembly CanFam3.1. Gene expression was measured, and differentially expressed genes (DEG) identified using DESeq2 with adjusted p‐values <0.05.

**Results:** Of 505 proteins detected, 59 (including haptoglobin and ficolin 1) were identified as differentially expressed between pre‐ and post‐splenectomy dogs. Pathway analysis for these revealed the enrichment of 16 pathways including complement activation. A total of 1389 DEGs were identified between NRIMA and healthy dogs; 681 were downregulated including EPB41, and 708 were upregulated including S100A12, S100A8, and S100A9 in NRIMA.

**Conclusions and Clinical Importance:** We obtained lists of proteins and genes that might be involved in suppressing normal hematopoiesis before splenectomy, including those that regulate complement activation. These findings could help understand the pathological mechanisms of NRIMA.

## 143 SMALL ANIMAL INTERNAL MEDICINE – HEPATOLOGY

## 144 Abstract HP01

### 144.1 16S rRNA Amplicon Sequencing of Bile from Healthy Cats and Cats with Suspected Hepatobiliary Disease

#### 144.1.1 **Tanner Slead** ^1^; Ben Callahan^2^; Megan Schreeg^1^; Gabriela Seiler^1^; Devorah Stowe^1^; Andrea Azcarte‐Peril^3^; Megan Jacob^1^; Jody Gookin^1^

##### 144.1.1.1 ^1^Veterinary Hospital, North Carolina State University; ^2^NCSU Microbiome Methods in Health and Disease Laboratory; ^3^UNC Microbiome Core Facility

**Background:** Bacterial cholangitis is a common disease of cats yet existence of a bile microbiome in cats is unknown. This knowledge gap may limit our understanding of the pathogenesis of hepatobiliary diseases in cats.

**Objectives:** To establish 1) if healthy cats harbor a core microbiota in gallbladder bile and 2) any relationship between results of conventional bile culture, cytology, and 16S rRNA gene amplicon sequencing (16S sequencing).

**Animals:** 43 cats with suspected hepatobiliary disease and 18 control cats from a local animal control facility.

**Methods:** Case‐control study. Bile was collected via percutaneous ultrasound‐guided cholecystocentesis from cats with suspected hepatobiliary disease and post‐mortem from control cats having concurrent histopathologic examination to exclude underlying disease. Bile cytology, aerobic/anaerobic culture, and 16S sequencing were performed.

**Results:** Both bile culture and 16S sequencing results were attained for 40 cats with suspected hepatobiliary disease and 18 controls. A positive culture was obtained from 10 cats with suspected hepatobiliary disease and 0 controls. 16S sequencing did not identify existence of a core bile microbiome. Cats with positive culture results for *E. coli* (n=7) had a concurrent bloom of additional pathogenic bacteria identified by 16S sequencing. Cats that were culture‐positive for non‐*E. coli* species (n=3) had neither the cultured bacteria nor other bacteria identified by sequencing.

**Conclusions and Clinical Importance:** Cat bile does not harbor a microbiome but may contain bacterial pathogens not identified by culture. These pathogens may contribute to pathogenesis of hepatobiliary disease, particularly in cats with bile *E. coli* infection.

## 145 Abstract HP02

### 145.1 Evaluation of Fasting Bile Acid Concentrations in Dogs with Sepsis

#### 145.1.1 **Lara Baptista** ^1^; Andrea Di bella^2^, DECVIM‐CA; Danica Pollard^3^, PhD

##### 145.1.1.1 ^1^Southern Counties Veterinary Specialists; ^2^RCVS and European Diplomate in Small Animal Medicine, Internal Medicine, Southern Counties Veterinary Specialists; ^3^The Rodhams

**Background:** Serum bile acid concentrations represent an early predictor of short‐term survival in critically ill people; however, their use as biomarkers in septic dogs has not been previously established.

**Objective:** Evaluate if fasting serum bile acid concentrations differ between septic and non‐septic dogs and identify any relationship with survival and hospitalization period in septic dogs.

**Animals:** Twenty‐six client‐owned dogs diagnosed with sepsis. Dogs presenting with a non‐hepatobiliary medical condition (non‐septic group; n=21) and dogs admitted for an elective orthopedic procedure, considered otherwise healthy (orthopedic group; n=29), were included as control groups.

**Methods:** Retrospective observational study of medical records of a single referral center from 2008 to 2020. Fasting serum bile acids, biochemical and hematological parameters were compared between groups, using the Kruskal‐Wallis with *post hoc* Dunn's test or one‐way ANOVA with *post hoc* Tukey's test. For septic dogs, the relationship between fasting serum bile acids and hospitalization period was assessed using Spearman rank correlation, and survival to discharge using the Mann‐Whitney U test.

**Results:** Fasting serum bile acids were significantly higher in the septic (median 17.5 μmol/L, interquartile range [IQR] 7.5–26.9 μmol/L) compared to the non‐septic group (median 6.9 μmol/L, IQR 4.7–9 μmol/L; *P*=0.013). However, no difference was identified between the septic and the orthopedic group (median 12 μmol/L, IQR 9–14 μmol/L; *P*=0.471). Fasting serum bile acid concentrations were not associated with hospitalization period (Spearman's rho =‐0.129; *P*=0.529) nor with survival (*P*=0.918).

**Conclusions and Clinical Importance:** Fasting serum bile acid concentrations were not considered prognostic indicators.

## 146 Abstract HP03

### 146.1 Comparison of Bacteriological Culture with Molecular Methods for Identifying Bacteria in Liver Samples from Dogs

#### 146.1.1 **Floris C. Dröes** ^1^; Courtney Jarvis^2^, PhD; Shannara Welch^3^; Jing Wu^4^; Jan Suchodolski^5^, MedVet, DrVetMed, PhD, AGAF, DACVM (Immunology); Sara Lawhon^6^, DVM, PhD, DACVM; Jonathan Lidbury^7^, BVMS, MRCVS, PhD, DACVIM, DECVIM‐CA; Jörg Steiner^8^, MedVet, DrMedVet, PhD, DACVIM, DECVIM‐CA, AGAF

##### 146.1.1.1 ^1^Gastrointestinal Laboratory, College of Veterinary Medicine and Biomedical Sciences, Texas A&M University; ^2^Director of Bioinformatics, MicroGen DX; ^3^Microbiology Technician I, Vet Med‐Teaching Hospital, Clinical Microbiology Laboratory, VMTH, Texas A&M University; ^4^Technical Laboratory Coordinator, Vet Med‐Teaching Hospital, Clinical Microbiology Laboratory, VMTH, Texas A&M University; ^5^Professor and Associate Director for Research/Head of Microbiome Science, Department of Small Animal Clinical Science, Gastrointestinal Laboratory, Texas A&M University; ^6^Professor in Veterinary Pathobiology/Director, Clinical Microbiology and Immunology, VTPB, Department of Veterinary Pathobiology, Texas A&M University; ^7^Associate Professor, Small Animal Internal Medicine Associate Director for Clinical Services, Department of Small Animal Clinical Science, Gastrointestinal Laboratory, Texas A&M University; ^8^University Regents Professor/University Distinguished Professor (with Tenure), Small Animal Internal Medicine/Dr. Mark Morris Chair in Small Animal Gastroenterology and Nutrition/Director, Gastrointestinal Laboratory (GI Lab), Department of Small Animal Clinical Science, Gastrointestinal Laboratory, Texas A&M University

**Background:** Culture has traditionally been used to detect and identify bacteria. Results can take several days and some species of bacteria are fastidious. Molecular methods could allow results to be obtained more quickly and might allow detection of fastidious species.

**Objective:** To compare bacteriological culture with 16S rRNA gene next‐generation sequencing (NGS) and qPCR for identifying bacteria in liver samples from dogs.

**Animals:** Liver biopsies obtained from 18 dogs presented for diagnostic workup of chronic liver disease.

**Methods:** Prospective cross‐sectional study. Liver biopsies were macerated and plated on standard media for aerobic and anaerobic bacteriological culture. The same biopsy sample was transported overnight in an anaerobic container on ice packs for NGS and qPCR. For 10 dogs, negative controls were obtained by swabbing the macerator and culture broth (together, or separately) prior to sample processing.

**Results:** Seventeen samples had a negative bacteriological culture. One culture positive sample (*Corynebacterium tuberculostearicum*) was negative by molecular methods. Four of 18 samples (22%) were positive by molecular methods only; reported bacteria varied widely. Concordant negative results between bacteriological culture and molecular methods were obtained in 13/18 (72%) samples. In 5/10 (50%) negative controls, NGS detected *E. coli* DNA (10^5^–10^7^ copies/gram).

**Conclusions and Clinical Importance:** Concordant negative results from bacteriological culture and molecular methods were obtained in a majority of liver samples from dogs. However, the clinical relevance of bacterial DNA in liver samples from dogs remains uncertain as half of the negative controls were reported to contain bacterial DNA.

## 147 Abstract HP04

### 147.1 Plasma Amino Acid Profiles in Dogs with Chronic Liver Disease

#### 147.1.1 **Rommaneeya Leela‐Arporn** ^1^; Karah Burns DeMarle^2^, DVM, DACVIM (SAIM); Calin Heinze^3^, VMD, MS, DACVN; Cynthia Leveille‐Webster^4^, DVM, DACVIM (SAIM)

##### 147.1.1.1 ^1^Cummings School of Veterinary Medicine, Tufts University; ^2^Veterinary Internist, MedVet Medical and Cancer for Pets; ^3^Associate Professor, Cummings School of Veterinary Medicine, Tufts University; ^4^Professor, Clinical Sciences, Cummings School of Veterinary Medicine, Tufts University

**Background:** Dogs with hepatocutaneous syndrome (HCS) have plasma hypoaminoacidemia. The specificity of this finding among dogs with other chronic hepatopathies (CH) is unknown.

**Hypothesis/Objectives:** To compare plasma amino acid panels (PAA) from a retrospective cohort of dogs with HCS with PAA in dogs with non‐HCS CH.

**Animals:** Forty client‐owned dogs with CH.

**Methods:** Dogs undergoing hepatic biopsy for chronic serum liver enzyme elevations were recruited. Twelve dogs each with vacuolar hepatopathy or chronic inflammatory disease and 8 dogs with congenital vascular/biliary disease were enrolled. Eight cases of HCS with PAA were retrospectively enrolled. PAA analysis was done at the UC Davis Amino Acid Laboratory. PAA concentrations in the 4 groups were compared using one‐way ANOVA and the Kruskal‐Wallis test, followed by the Tukey Kramer HSD and Steel‐Dwass test for *post hoc* analysis, respectively. To find a unique PAA pattern in dogs with HCS, Lasso regression analysis with AIC forward selection was performed.

**Results:** There was no difference in breed, age or sex among the 4 groups. Compared to non‐HCS liver diseases, dogs with HCS had significantly lower concentrations of aspartic acid, threonine, serine, asparagine, glutamine, glycine, alanine, citrulline, methionine, lysine, histidine, arginine, and proline (*P*<0.0019 after Bonferroni correction). The Lasso regression analysis revealed that low glutamine, glycine, citrulline, arginine and proline concentrations were characteristic of dogs with HCS.

**Conclusions and Clinical Importance:** Plasma hypoaminoacidemia does not occur in dogs with non‐HCS CH. Determination of PAA is warranted as a noninvasive biomarker for diagnosis of HCS in dogs.

## 148 Abstract HP05

### 148.1 Abstracts HP05: Urine Isoprostane Concentrations in Dogs with Liver Disease

#### 148.1.1 **Robert Kyle Phillips** ^1^; Jörg Steiner^2^, MedVet, DrMedVet, PhD, DACVIM, DECVIM‐CA, AGAF; Jan Suchodolski^3^, MedVet, DrVetMed, PhD, AGAF, DACVM; Jonathan Lidbury^4^, BVMS, MRCVS, PhD, DACVIM, DECVIM‐CA

##### 148.1.1.1 ^1^GI Lab, Texas A&M University; ^2^Director of Gastrointestinal Laboratory, Small Animal Internal Medicine, GI Lab, Texas A&M University; ^3^Associate Director for Research, Small Animal Internal Medicine, GI Lab, Texas A&M University; ^4^Associate Director for Clinical Services, Small Animal Internal Medicine, GI Lab, Texas A&M University

**Background:** Isoprostanes are stable end products of lipid peroxidation that have been used as markers of oxidative stress in humans and dogs. It has previously been reported that a cohort of dogs with liver disease (inflammatory, metabolic, vascular, or neoplastic) had increased urine isoprostane concentrations compared to healthy control (HC) dogs. However, urine isoprostane concentrations have not been individually reported for different types of canine liver disease.

**Hypothesis/Objectives:** To assess oxidative stress in dogs with different types of liver disease.

**Animals:** Urine was collected from 20 HC dogs and from dogs with liver disease, including 25 with chronic hepatitis (CH), 7 with steroid hepatopathy (SH), and 8 with congenital portosystemic shunt (CPSS).

**Methods:** Prospective, observational study. Urinary 15‐F_2t_‐isoprostane concentrations were measured by gas chromatography/negative ion chemical ionization mass spectrometry and normalized to urine creatinine concentrations. Concentrations were compared between groups using a Kruskal‐Wallis test, followed by Dunn's multiple comparisons tests. Significance was set at P<0.05.

**Results:** The median [range] urine 15‐F_2t_‐isoprostane to creatinine ratios (ng/mg UCr) were 3.6 [2.2–12.4], 5.7 [2.4–11.3], 4.8 [2.4–8.6], and 12.5 [2.9–22.9] for HC dogs and dogs with CH, SH, or CPSS, respectively. CPSS dogs had significantly higher urine isoprostane concentrations compared to HC dogs (P=0.004).

**Conclusions and Clinical Importance:** These findings suggest that dogs with CPSS experience increased oxidative stress compared to HC dogs. If confirmed in a larger group of dogs, this may have therapeutic implications.

## 149 Abstract HP06

### 149.1 Quantification of MicroRNAs 1275, 222, and 21 in Serum from Dogs with Chronic Hepatitis

#### 149.1.1 **Adrian Tinoco Najera**; Patricia Eri‐Ishii; Yuri Lawrence; Jan Suchodolski; Joerg Steiner; John Lidbury

##### 149.1.1.1 Veterinary Gastrointestinal Laboratory, Texas A&M University

**Background:** MicroRNAs 1275, 222, and 21 are involved in copper homeostasis and their serum expression has been reported to be altered in human patients with Wilson's disease.

**Objective:** To quantify microRNAs 1275, 222, and 21 in serum from dogs with copper‐associated chronic hepatitis (CuCH), dogs with idiopathic chronic hepatitis (iCH), and healthy control dogs.

**Animals:** Stored serum samples from 8 dogs with histologically confirmed CuCH, 8 dogs with histologically confirmed iCH, and 8 healthy control dogs.

**Methods:** Retrospective cross‐sectional study. Target MicroRNAs were quantified by real‐time, reverse transcription polymerase chain reaction (RT‐qPCR). MicroRNA 26b was used as housekeeping gene. Relative quantification was calculated using the ΔΔCt method. Kruskal‐Wallis tests were used to compare differences in copy numbers between groups. Statistical significance was set at P<0.05.

**Results:** Dogs with CuCH showed a decreased expression of MicroRNA 1275 compared to healthy controls (CuCH [median; range]: 0.15; 0.0–0.40; healthy controls: 1.7; 0.10–10.3) but this difference did not reach statistical significance (P=0.06; Figure 1). Other *post hoc* comparisons between groups were not significant (P=0.13). No significant differences between groups were observed for MicroRNA 222 (P=0.10) or 21 (P=0.15), respectively.

**Figure:**
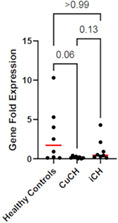
**Figure 1.** Kruskal‐Wallis test with multiple comparisons for MicroRNA 1275 CuCH = Copper associated chronic hepatitis, iCH = idiopathic chronic hepatitis.

**Conclusions and Clinical Importance:** These results, from a small number of dogs, do not support the diagnostic utility of microRNA 222 or 21 for the diagnosis of CuCH in dogs. However, larger cohort studies evaluating the utility of MicroRNA 1275 are warranted to determine the diagnostic utility of MicroRNA 1275.

## 150 Abstract HP07

### 150.1 Demographic and Histopathologic Associations with Elevated Hepatic Copper Concentrations in Dogs

#### 150.1.1 **Tarini V. Ullal** ^1^; Steven Lakin^2^, DVM, PhD; Gallagher Brooke^2^; Nicholas Sbardetelli^2^; Zaid Abdo^3^, BSc, MSc, PhD; David Twedt^4^, DVM, DACVIM (SAIM)

##### 150.1.1.1 ^1^University of California, Davis; ^2^Colorado State University; ^3^Professor, Microbiology, Immunology, Pathology, Colorado State University; ^4^Professor, Colorado State University

**Background:** Copper associated hepatopathy (CAH) has become an important and prevalent malady since the 1990s, coincidental with changes in copper content in commercial dog foods. Understanding the demographic and histopathologic features related to hepatic copper (Cu) concentrations may aid in identifying and better understanding dogs with CAH.

**Hypothesis/Objectives:** The primary aim was to describe associations between hepatic Cu concentrations and demographic and histopathologic features.

**Animals:** Dogs that underwent liver histopathology and Cu quantification at a veterinary diagnostic laboratory between July 2010 and February 2020.

**Methods:** Data was retrospectively collected from medical records. A Gaussian multiple regression model on the log scale was used to evaluate associations between hepatic Cu and demographic features and key histologic features of necroinflammation.

**Results:** Of 4,559 cases meeting criteria, 49% had hepatic Cu >400 and 19% had Cu >1000 μg/g dw (dry weight) (reference range 120–400 μg/g dw). Mean hepatic Cu was 704 μg/g dw, range 4.5–31,500. Severity of inflammation (mild, moderate, and severe) and necrosis/apoptosis were associated with increased hepatic Cu (p<0.01). Age was negatively associated (p<0.02), but specific breeds (Doberman pinscher, Labrador, and West Highland white terrier) and the diagnosis of chronic hepatitis was associated with increased hepatic Cu (p<0.001).

**Conclusion and Clinical Importance:** Elevated hepatic Cu is prevalent amongst dogs. Severity of inflammation and necrosis/apoptosis are predictive of higher hepatic Cu. Dogs with necroinflammation of the liver have significantly higher hepatic Cu compared to dogs without.

**Figure:**
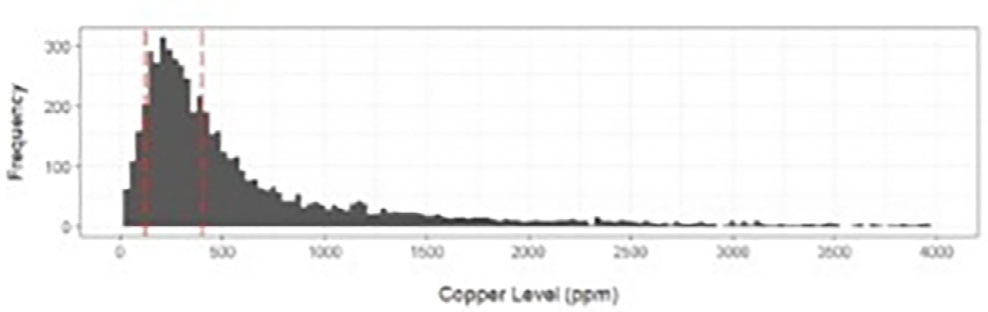
**Image 1** Truncated copper level distribution, normal range in red

**Figure:**
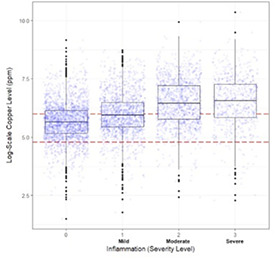
**Image 2.** Inflammation severity against log‐scale copper level

## 151 Abstract HP08

### 151.1 Clinical Use of Ursodiol in Feline Medicine

#### 151.1.1 **Emily Jachec** ^1^; Alexandra Wood^2^, DVM; Jenessa Winston^3^, DVM, PhD, DACVIM

##### 151.1.1.1 ^1^College of Veterinary Medicine, The Ohio State University;^2^Internal Medicine Resident, Veterinary Clinical Sciences, The Ohio State University; ^3^Assistant Professor, Veterinary Clinical Sciences, The Ohio State University

**Background:** Ursodiol is a naturally occurring bile acid prescribed to cats for a wide range of gallbladder and liver conditions. Ursodiol is known for its ability to expand the bile acid pool by increasing bile flow and displacing hepatotoxic bile acids. Additionally, ursodiol has anti‐inflammatory and anti‐fibrotic properties. Therefore, ursodiol is used in a variety of feline hepatic diseases, including acute/chronic hepatopathies, cholangiohepatitis, and for gallbladder abnormalities. Additionally, veterinarians prescribe a wide range of dosages and duration of ursodiol administration. Prescribing trends and clinical characteristics of feline patients receiving ursodiol is unknown.

**Objective:** This retrospective study aimed to determine the clinical use, dosage, and duration of therapy of ursodiol administration in feline patients at a teaching hospital over 13 years (2008–2021).

**Animals:** Since 2008, 88 feline patients were prescribed ursodiol at the Ohio State University Veterinary Medical Center and were included in this retrospective study.

**Methods:** Metadata from these patients, including signalment, history, diagnostics (including laboratory data and diagnostic imaging), ursodiol dosage/duration, and patient outcomes were recorded.

**Results:** Ursodiol was most prescribed as a hepatoprotectant (29/88, 33%), for gallbladder sludge (26/88; 30%), or for cholestasis (17/88; 19%) in feline patients. The most common prescribed dose was 10.89 mg/kg/day (range 5.2 to 33.9 mg/kg/day). An accurate duration of ursodiol administration was unable to be determined.

**Conclusions and Clinical Importance:** Overall, these findings document ursodiol prescribing trends and clinical characteristics of feline patients presenting to a teaching hospital. These results can help guide novel applications of ursodiol administration in feline patients.

## 152 Abstract HP09

### 152.1 Retrospective Review of Clinical Presentation, Survival and Response to Therapy in Dogs with Granulomatous Hepatitis

#### 152.1.1 **Kayla Prentice** ^1^; Julie Callahan‐Clark^2^, DVM, DACVIM (SAIM); Nicola Parry^3^, BSc, MSc, BVSc, DACVP, FRCVS; Cynthia Leveille‐Webster^4^, DVM, DACVIM (SAIM)

##### 152.1.1.1 ^1^Tufts VETS; ^2^Staff Internist, Tufts VETS; ^3^Head of Pathology, Pathology, School of Veterinary Medicine, University of Surrey; ^4^Cummings School of Veterinary Medicine at Tufts University

**Background:** Granulomatous hepatitis (GH) is a form of chronic hepatitis in dogs on which limited information has been published.

**Hypothesis/Objective:** The objective was to describe the presentation, imaging findings, survival time, and response to immunosuppressants in dogs with GH.

**Animals:** Twenty‐nine client‐owned dogs with confirmation of GH on hepatic biopsy.

**Methods:** This was a retrospective observational study. Pathology records from an academic medical center were searched. Inclusion criteria included a histopathologic diagnosis of GH based on WSAVA guidelines, absence of an identified etiology and a medical record which included clinical presentation, clinical pathologic and ultrasound findings, treatment, and adequate follow‐up to determine survival times.

**Results:** The median age was 7 yr (range, 0.66 to 12 yrs). Nineteen breeds were represented. Decreased appetite (19/29), lethargy (16/29), and fever (13/29) were seen. All dogs had increases in serum transaminases, while 21/29 and 12/24 had hyperbilirubinemia and neutrophilia, respectively. Hepatomegaly (12/26) and hypoechoic nodules (7/26) were common ultrasound findings. Histopathologic necroinflammatory scores were moderate to severe in 90% of dogs, while fibrosis scores were commonly graded as mild (75%). Overall survival was 635 days (range, 1–6442 days). Kaplan Maier analysis showed that 19 dogs treated with immunosuppressants survived longer (1074 days, range 18 to 2482) than the 8 dogs not treated with them, (480 days, range 1 to 1526). (p=0.04). Effect size was 0.80 (95% confidence interval, 0.36–1.23)

**Conclusion and Clinical Importance:** GH in dogs is associated with high histopathologic grade, fever, neutrophilia, and an apparent improved survival with immunosuppressants.

## 153 SMALL ANIMAL INTERNAL MEDICINE ‐ IMMUNOLOGY

## 154 Abstract IM01

### 154.1 Effect of Corticosteroid Administration on Serum C‐reactive Protein Concentrations in Healthy Dogs

#### 154.1.1 **Romy M. Heilmann** ^1^; Niels Grützner^2^; Peter Kook^3^; Stefan Schellenberg^4^; Jan Suchodolski^5^; Joerg Steiner^5^

##### 154.1.1.1 ^1^College of Veterinary Medicine, University of Leipzig; ^2^Veterinary Specialist in Porcine Health Management, VetaRegio; ^3^Vetsuisse Faculty, University of Zurich; ^4^Small Animal Clinic Aarau West; ^5^Gastrointestinal Laboratory, College of Veterinary Medicine and Biomedical Sciences, Texas A&M University

**Background:** Serum C‐reactive protein (CRP) is a biomarker of systemic inflammation in dogs and is used in clinical practice to aid in diagnosing and monitoring primary inflammatory conditions (e.g., immune‐mediated polyarthritis). Treatment of these diseases often includes anti‐inflammatory or immunosuppressive doses of corticosteroids. However, potential effects of corticosteroid administration on serum CRP concentrations in dogs have not been studied.

**Objective:** To evaluate serial serum CRP concentrations in healthy dogs receiving high‐dose corticosteroid treatment of >4 weeks.

**Animals:** Samples from a prospective cohort study including 12 healthy Beagle dogs were used.

**Methods:** Serum samples obtained before initiation (baseline) and on days 1, 5, and 28 of receiving either 8 mg/kg hydrocortisone PO q12h (6 dogs) or placebo (6 control dogs) were used. Serum CRP concentrations were measured in all samples (stored at ‐80°C) using the TriDelta canine CRP‐ELISA (total of 2 assay runs using the same assay reagents). A MANOVA model with Box‐Cox transformed (l=‐0.93) data followed by individual t‐tests served for statistical analysis, with *P*<0.05 indicating significance.

**Results:** Serum CRP concentrations slightly decreased over the 4 weeks in both groups of dogs, without a significant effect of hydrocortisone administration (*P*=0.7605; MANOVA). Two baseline samples and one sample obtained on day 1 of hydrocortisone treatment yielded serum CRP concentrations above the upper reference limit (>7.6 mg/L); all other measurements were within the reference interval.

**Conclusions and Clinical Importance:** These findings suggest a lack of a confounding effect of hydrocortisone administration on serum CRP concentrations in dogs.

## 155 Abstract IM02

### 155.1 Evaluation for Anti‐Erythrocyte and Anti‐Platelet Antibodies in Healthy Dogs Administered Lokivetmab

#### 155.1.1 **Megan Slaughter**; Michael Lappin, DVM, PhD, DACVIM; Nida Chornarm, DVM, PhD Candidate; Sarah Shropshire, DVM, PhD, DACVIM

##### 155.1.1.1 College of Veterinary Medicine and Biomedical Sciences, Colorado State University

**Background:** Lokivetmab (Cytopoint®) is an anti‐IL‐31 monoclonal antibody used for management of canine allergic pruritis. IL‐31 is a cytokine shown to activate the neuronal signaling pathway associated with itch in dogs. Multiple publications confer the efficacy and safety of lokivetmab for allergic disease when given at recommended dosages. However, there have been anecdotal concerns that lokivetmab could trigger an immune‐mediated response leading to immune‐mediated hemolytic anemia (IMHA) or immune thrombocytopenia (ITP). No study to date has evaluated for the development of antibodies against erythrocytes or platelets when using this drug.

**Hypothesis/Objectives:** Determine if anti‐erythrocyte antibodies (AEA) or anti‐platelet antibodies (APA) develop following a standard dose therapy of lokivetmab in healthy dogs. We hypothesized that the development of AEA or APA following a single‐dose of lokivetmab would be rare and unlikely of clinical significance.

**Animals:** 8 healthy adult purpose‐bred research beagles.

**Methods:** Prospective descriptive study. Whole blood samples were taken at baseline (day 0) prior to lokivetmab administration and on days 14 and 28 following lokivetmab administration. A CBC and AEA and APA direct flow cytometry assays were performed at all time points.

**Results:** No dogs exhibited anemia or thrombocytopenia and all were negative for AEA and APA at all measured time points.

**Conclusions and Clinical Importance:** There was no evidence of APA or AEA formation in healthy dogs administered lokivetmab at standard dosages. Future work includes a clinical trial for the evaluation of anemia, thrombocytopenia, AEA, and APA in client‐owned allergic dogs receiving lokivetmab.

## 156 Abstract IM03

### 156.1 Immune Response of Cats After Simultaneous versus Separate Vaccination Against Rabies and Feline Leukemia Virus

#### 156.1.1 **Anna‐Karina Weidinger** ^1^; Michèle Bergmann^1^; Matthias König^2^; Yury Zablotski^1^; Katrin Hartmann^1^

##### 156.1.1.1 ^1^Clinic of Small Animal Medicine, LMU Munich; ^2^Viro Vet Diagnostic, Biomedical Research Centre, Giessen

**Background:** To minimize the risk of feline injection site‐associated sarcomas, cats should be vaccinated with non‐adjuvanted feline leukemia virus (FeLV) and rabies vector vaccines, which are currently not licensed for simultaneous use and have to be administered on separate visits.

**Hypothesis:** The aim of the study was to investigate the immune response of cats after vaccination against rabies and FeLV at simultaneous **versus** separate visits using canarypox‐vectored vaccines.

**Animals:** 106 healthy, FeLV antigen‐negative, client‐owned kittens (8–12 weeks) were included.

**Methods:** In this prospective randomized study, all cats received primary vaccinations against rabies (day 0) and FeLV (day 28, 56). After one year, the study group (n=52) received booster vaccinations against rabies and FeLV simultaneously (day 364). The control group (n=54) received booster vaccinations against rabies (day 364) and FeLV (day 392) separately (4 weeks apart). Anti‐rabies antibodies were determined by fluorescent neutralization assay (days 28, 364, 392) and significant differences in anti‐rabies antibodies between both groups were determined by Mann‐Whitney U test.

**Results:** Four weeks after the one‐year booster vaccination against rabies, all cats had anti‐rabies antibodies (≥0.5 IU/ml), and there was no significant difference (p=0.141) in the median titer between the study group (14.30 IU/mL) and the control group (21.39 IU/mL).

**Conclusions and Clinical Importance:** According to the World Health Organization and the World Organization for Animal Health, anti‐rabies antibodies ≥0.5 IU/ml indicate protection against rabies. Simultaneous administration of FeLV and rabies vector vaccines does not interfere with the development of anti‐rabies antibodies.

## 157 Abstract IM04

### 157.1 Relapse Risk Factors for Immune Mediated Hemolytic Anemia: A Retrospective Study of 223 Dogs

#### 157.1.1 **Sidney Bannister** ^1^; Deborah Keys^2^, PhD; Ewan Wolff^3^, DVM, PhD, DACVIM

##### 157.1.1.1 ^1^BluePearl Maitland; ^2^Kaleidoscope Statistics, LLC; ^3^Internal Medicine, BluePearl Maitland

**Background:** IMHA (immune mediated hemolytic anemia) is defined as an immune mediated destruction of red blood cells. Relapses are known to occur but risk factors are poorly defined.

**Hypothesis:** We hypothesize that patients with more severe IMHA on presentation (lower PCV at diagnosis, more transfusions given) would have higher relapse rates.

**Animals:** IMHA was defined as a PCV less than 30% at diagnosis and if one of the following were identified: spherocytes, positive Coombs’ test, positive slide agglutination. Records between January 2005 to December 2019 from one specialty hospital found 223 dogs who met these criteria.

**Methods:** This was a retrospective case‐control study with a significance threshold of 0.05.

**Results:** There were 21 relapses. Overall relapse probabilities (95% CI) at 3 and 12 months were 5% (2–11) and 12% (7–20). Dogs who received two or more transfusions had a higher probability of relapse than dogs who received one transfusion or none (p=0.044). Dogs who had PCV <10% had a 27% (95% CI 10–63) probability of relapsing within 1 year compared to dogs who had PCV >10% who had a 9% (95% CI 5–18) probability of relapsing within 1 year (p=0.010 log‐rank).

**Conclusions:** Patients with extremely low PCVs at diagnosis or need multiple transfusions are significantly more likely to relapse. These patients would likely have a poorer prognosis.

## 158 Abstract IM05

### 158.1 Use of a Flow Cytometry Assay to Detect Anti‐erythrocyte Antibodies in 116 Anemic Client‐Owned Dogs

#### 158.1.1 **Nida Chornarm** ^1^; Melissa Brewer^2^; Sarah Shropshire^3^, DVM, PhD, DACVIM; Christine Olver^4^; Steve Dow^3^; Michael Lappin^3^

##### 158.1.1.1 ^1^Colorado State University; ^2^Center for Companion Animal Studies, Colorado State University; ^3^Department of Clinical Sciences, College of Veterinary Medicine and Biomedical Sciences, Colorado State University; ^4^Department of Microbiology, Immunology, and Pathology, College of Veterinary Medicine and Biomedical Sciences, Colorado State University

**Background:** Anti‐erythrocyte antibodies play a major role in primary and secondary immune‐mediated hemolytic anemia (IMHA). A direct flow cytometry assay to detect antibodies on the RBC is quantitative (% IgG binding) and an alternative to direct Coombs testing.

**Hypothesis/Objectives:** To report the % IgG binding among groups of anemic dogs and to compare to direct Coombs test results when available.

**Animals:** Final diagnoses for 116 client‐owned, anemic dogs were classified as idiopathic IMHA, neoplasia, blood‐borne pathogen infection, and other etiologies.

**Methods:** Whole blood in EDTA was stored at 4°C and analyzed for % IgG binding and direct Coombs test (positive >1:4) within 24 hours of collection. The % IgG binding among disease categories was not normally distributed and so medians were compared by Mann‐Whitney U test.

**Results:** % IgG binding was detected in some dogs with IMHA (30/46 [65.2%]; median 10.63%, range 0.24–99.8), neoplasia (3/34 [8.8%]; 3.16% 0.14–16.85), blood‐borne pathogens (1/1 [100%]; 5.18%), and with other etiologies (5/35 [14.3%]; 0.83%, 0.16–95.1). Results from flow and Coombs test were discordant for 13 of 42 dogs (30.9%) and glucocorticoids had been administered to 11/13 (84.6%) dogs with discordant results before testing.

**Conclusions and Clinical Importance:** Dogs with idiopathic IMHA have significantly higher % IgG binding than dogs with other causes of anemia. Explanations for the discordant results for the 2 assays are currently being explored.

## 159 Abstract IM06

### 159.1 The Intestinal Microbiome of Dogs with Immune‐Mediated Hemolytic Anemia or Immune Thrombocytopenia

#### 159.1.1 **Po‐Yu Liu** ^1^; Kathryn McGonigle^2^; Alicia Carroll^2^; June DiBona^3^; Heather Scavello^2^; Rene Martins^2^; Sanya Mehta^2^; Elise Krespan^2^; Elizabeth Lunde^4^; Dana LeVine^5^; Claire Fellman^6^; Robert Goggs^7^; Dong Xia^1^; Daniel Beiting^3^; Oliver Garden^8^

##### 159.1.1.1 ^1^Royal Veterinary College; ^2^University of Pennsylvania; ^3^School of Veterinary Medicine, University of Pennsylvania; ^4^Auburn University; ^5^Department of Clinical Sciences, Auburn University; ^6^Cummings School of Veterinary Medicine, Tufts University; ^7^Cornell University; ^8^School of Veterinary Medicine, Louisiana State University

**Background:** Little is known about the impact of the intestinal microbiome on immune‐mediated hemolytic anemia (IMHA) and immune thrombocytopenia (ITP) in dogs.

**Hypothesis/Objectives:** We hypothesize that IMHA and ITP in dogs are associated with intestinal dysbiosis. We evaluated the fecal microbiome of diseased dogs at presentation and after initiation of treatment.

**Animals:** 25 affected and 13 healthy control dogs were included in the study. Samples were collected from affected dogs at presentation, and 2 and 8 weeks after initiation of treatment.

**Methods:** 16S rRNA gene sequencing was used to profile the fecal microbiome of affected dogs and healthy controls. Microbiome diversity and composition were analyzed in the context of disease status.

**Results:** The fecal microbiota of diseased dogs were significantly more diverse than those of healthy dogs at presentation (Shannon and Simpson indices, p≤0.03). The genus *Mitsuokella* was enriched in diseased dogs (q<0.001), whereas the genus *Treponema* was enriched in healthy dogs. The genus *Treponema* was also an enterotype of all study dogs (odds ratio [OR]=0.19, 95% confidence interval [CI]=0.04–0.68). The Pirellulaceae p−1088−a5 (OR=2.59, 95% CI=1.14−7.27) and Lachnospiraceae NK4A136 (OR=3.13, 95% CI=1.02−11.77) groups were associated with disease. Beta diversity analysis demonstrated a shift in microbiome composition toward that of healthy dogs with treatment.

**Conclusions and Clinical Importance:** IMHA and ITP in dogs may be associated with intestinal dysbiosis. Specific bacterial genera may serve as disease biomarkers. Additional studies are warranted to evaluate whether microbiome composition or diversity play a causal role in disease initiation or progression.

## 160 Abstract IM07

### 160.1 Romiplostim in Primary and Secondary Thrombocytopenia: A Retrospective Study of Dogs and Cats

#### 160.1.1 **Ye‐In Oh** ^1^; Jin‐Kyoung Kim^2^, DVM; Yong‐hun Oh^3^, DVM; Ju‐Hyun Ahn^3^, DVM, PhD; Su‐Min Park^3^, DVM; Tae‐Hee Kim^3^, DVM, MS; Kyoung‐Won Seo^4^, DVM, PhD, DAiCVIM; Hwa‐Young Youn^5^, DVM, PhD

##### 160.1.1.1 ^1^Seoul National University; ^2^Director, Internal Medicine, Haemaru Referral Animal Hospital; ^3^Internal Medicine, Seoul National University; ^4^Associated Professor, Internal Medicine, Seoul National University; ^5^Professor, Internal Medicine, Seoul

**Background:** Romiplostim is a thrombopoietin receptor agonist and is used for the treatment of immune‐mediated thrombocytopenia (IMT) in humans and there is limited literature in dogs.

**Hypothesis/Objectives:** The aim of this study is to investigate the effects and adverse events of romiplostim in dogs and cats with primary thrombocytopenia or secondary thrombocytopenia.

**Animals:** Twenty‐eight dogs and 2 cats were included.

**Methods:** A retrospective study was conducted by extracting data from electrical medical records from 2020 to 2021. We evaluated the effects of treatment and adverse events of romiplostim for primary IMT and secondary thrombocytopenia caused by several underlying conditions including lymphoma, babesiosis, pancytopenia, feline leukemia virus infection, post‐vaccination, liver failure, and systemic lupus erythematous.

**Results:** The platelet count increased to the reference range in 15 dogs, and 10 of them survived. Response rate was primary IMT 58.3% (7/12), pancytopenia of unknown cause 100% (3/3), chemotherapy‐induced thrombocytopenia 66.6% (2/3), hepatic failure 100% (1/1), post‐vaccination 100% (1/1), babesiosis 100% (1/1), primary pancytopenia 0% (0/4), paraneoplastic thrombocytopenia 0% (0/3). The recovery period of platelet count after administration of romiplostim was 7 (range, 2–10) days. Romiplostim was well‐tolerated.

**Conclusions and Clinical Importance:** Romiplostim was well tolerated and effective in approximately one‐half of primary IMT and secondary thrombocytopenia in dogs.

## 161 Abstract IM08

### 161.1 Anti‐oxidative and Immunomodulatory Effects of Telmisartan in Healthy Cats

#### 161.1.1 **Jade Peralta**; Petra Cerna; Jennifer Hawley; Michael Lappin; Alice Wang

##### 161.1.1.1 College of Veterinary Medicine and Biomedical Sciences, Colorado State University;

**Background:** Telmisartan is licensed in the USA to control hypertension in cats. In other species, telmisartan also has immunomodulatory properties and is an anti‐oxidant.

**Objective/Hypothesis:** To determine whether telmisartan administered to cats has antioxidant or immune modulation effects.

**Animals:** Eight healthy, young adult, purpose‐bred, mixed sex cats.

**Methods:** Cats (n=8) were administered telmisartan at 1.5 mg/kg PO twice daily for 14 days followed by 1.5 mg/kg PO once daily for 14 days. Superoxide dismutase (SOD) and plasma glutathione peroxidase (GPx) activities were determined in plasma using commercial kits and compared between day 0 and 14. The mRNA of select cytokine genes detected in blood by RT‐PCR from the telmisartan cats on day 14 and 28 and compared to 6 control cats. Data were assessed for normalcy and then compared by Student's T test or Mann Whitney U test.

**Results:** Plasma SOD concentrations between day 0 and day 14 were similar. Plasma GPx activity was increased on day 14 (mean=27, std=21.9) compared to day 0 (mean=23.5, std=26.3) but not significant (P=0.78). While no differences in IL1‐beta, IL6, IFN‐alpha, and IFN‐beta were detected, TNF‐alpha levels were greater in the day 14 (P=0.04) and 28 (P=0.01) samples and IFN‐gamma levels were significantly greater in the day 28 samples (P=0.02).

**Conclusions and Clinical Importance:** Telmisartan has immunomodulatory effects in healthy cats, which suggests potential alternate uses of this drug.

## 162 SMALL ANIMAL INTERNAL MEDICINE ‐ INFECTIOUS DISEASE

## 163 Abstract ID01

### 163.1 Incidence of Acute Kidney Injury in Dogs with Systemic Infections Treated with Amphotericin B

#### 163.1.1 **Jennifer Chan** ^1^; Krystle Reagan^2^, DVM, PhD, DACVIM (SAIM); Jonathan Dear^2^, DVM, MAS, DACVIM (SAIM); Carrie Palm^2^, DVM, MAS, DACVIM (SAIM)

##### 163.1.1.1 ^1^University of California‐Davis, Davis, CA, USA; ^2^Assistant Professor, Medicine and Epidemiology, University of California‐Davis, Davis, CA, USA

**Background:** Amphotericin‐B (AmB) is an essential medication for the treatment of systemic mycoses. Nephrotoxicity is a known complication of AmB treatment but the incidence and risk factors for acute kidney injury (AKI) following its administration are not known in dogs.

**Objective:** Determine the incidence of and risk factors for AKI in dogs receiving AmB.

**Animals:** 51‐client owned dogs receiving AmB for the treatment of systemic infections.

**Methods:** Retrospective study. Signalment, potential risk factors (dehydration, renal disease, recent non‐steroidal anti‐inflammatory drug administration, recent anesthesia, ICU hospitalization, neurologic deficits, inpatient status), AKI development (creatinine >0.3 mg/dL from baseline), drug formulation (deoxycholate (AmB‐D) or lipid complex (ABLC)), dose, and treatment duration were recorded. Incidence of AKI was determined, and odds ratios were calculated for potential risk factors.

**Results:** Incidence of AKI was 5/12 (41%) for dogs receiving AmB‐D and 14/39 (36%) for dogs receiving ABLC. Of the 19 dogs that developed AKI, 16 (84%) continued treatment after a break in the planned dosing protocol. Median cumulative dose administered prior to first AKI was 6.9 mg/kg for AmB‐D and 22.5 mg/kg for ABLC. ICU hospitalization (odds ratio (OR) 0.21, 95% confidence interval (CI) 0.58–0.87) and inpatient status (OR 0.25, and 95% CI 0.07–0.86) were associated with a decreased odds of AKI.

**Conclusions and Clinical Importance:** Incidence of AKI with AmB is common, but does not always preclude continued treatment. The incidence of AKI is similar between AmB‐D and ABLC, but dogs receiving ABLC tolerated a higher total dose before AKI developed.

## 164 Abstract ID02

### 164.1 Assessment of Veterinary Technician Interest in and Barriers to Engagement with Hospital Antimicrobial Stewardship

#### 164.1.1 **Madeleine R. Stein** ^1^; Claire Fellman^1^, DVM, PhD, DACVIM, DACVCP; Annie Wayne^2^, DVM, MPH, DAVECC; Kirthana Beaulac^3^, PharmD; Shira Doron^4^, MD

##### 164.1.1.1 ^1^Cummings School of Veterinary Medicine; ^2^Massachusetts Veterinary Referral Hospital, Ethos Veterinary Health;^3^Emerson Hospital; ^4^School of Medicine, Tufts Medical Center, Tufts University

**Background:** Antimicrobial stewardship programs (ASPs) are increasingly prevalent in companion animal hospitals, but despite the critical role of veterinary technicians in patient care, there is often a lack of ASP focus on support staff.

**Hypothesis/Objectives:** To establish baseline attitudes and practices of veterinary technicians in a tertiary care center around antimicrobial use, prior to the institution of a hospital ASP.

**Animals:** None.

**Methods:** This was a prospective, survey‐based study. Data were collected through means of an online questionnaire, consisting of a combination of closed and open‐ended questions, sent via a targeted email list. Descriptive statistics were used for data analysis.

**Results:** A total of 39 completed responses were received. Seventy‐nine percent of participants felt that antimicrobial stewardship was important, and 54% wanted to be more involved in the ASP. Most participants were comfortable suggesting non‐antimicrobial treatment alternatives with doctors or students (92%), but fewer were comfortable discussing the need for antimicrobial time‐outs (54%). Technicians listed a lack of familiarity with appropriate treatment choices, and a perception that some doctors may not be open to input from support staff among their barriers to conversations with doctors. Technician suggestions on how to include support staff in the ASP included education on antimicrobial use, and a focus on non‐prescribing practices such as infection control.

**Conclusions and Clinical Importance:** Our research demonstrates an interest in and a desire to learn more about antimicrobial stewardship in this technician population. Targeted engagement of veterinary support staff may increase success within hospital ASPs.

## 165 Abstract ID03

### 165.1 Effect of Urine Concentration on the Growth of Canine Uropathogens: An *in Vitro* Study

#### 165.1.1 **John A. Shamoun** ^1^; Megan Jacob^2^, MS, PhD

##### 165.1.1.1 ^1^College of Veterinary Medicine, North Carolina State University; ^2^Associate Professor and Director of Diagnostic Laboratories, Population Health and Pathobiology, College of Veterinary Medicine, North Carolina State University

**Background:** Conflicting data exist regarding the effect of urine concentration on the growth of canine uropathogens in clinical or subclinical urinary tract infection (UTI). Assessing bacterial growth of uropathogens in varying urine concentrations may improve our understanding of factors influencing pathogenesis or treatment of UTI.

**Hypothesis:** Different uropathogens will exhibit unique growth patterns that are dependent on urine concentration and specific *in vitro* urine diluent.

**Animals:** Stored, frozen, culture negative canine urine was used, which was previously collected from healthy patients.

**Methods:** The urine was diluted from a starting specific gravity (USG) of 1.035 with phosphate buffered saline or sterile water to final USGs of 1.005, 1.015, or 1.025. Each was inoculated with 1:100 0.5 McFarland concentration of 1 of 4 clinical uropathogens (Escherichia coli, Enterococcus faecalis, *Staphylococcus pseudintermedius*, and *Klebsiella pneumoniae*) and incubated. The bacterial concentration (CFU/mL) was determined at hour 0 and 8. All counts were log transformed and the differences from pre‐ to post‐incubation were represented graphically for each organism.

**Results:** Some organisms exhibit stunted growth in very dilute (*E. coli*, *S. pseudintermedius*) and well concentrated urine (*E. coli*, *K. pneumoniae*), while others exhibit modest growth independent of urine concentration (*E. faecalis*) and diluent. There was not a consistent diluent effect on supporting uropathogen growth *in vitro*.

**Conclusions:** Different bacterial genera previously associated with UTI exhibit unique growth patterns that are dependent on urine concentration and diluent, which may suggest that uropathogens have individually specific environmental preferences for optimal growth.

## 166 Abstract ID04

### 166.1 Evaluation of MiQLab System to Detect Bacterial Pathogens and Antimicrobial Resistance Genes in Bacterial Isolates

#### 166.1.1 **Eric C. DiBiasio** ^1^; Manoj Nair^2^, MVSc, PhD; Zihua Wang^3^, MBA, PhD; Nathan Walsh^4^, PhD; Jack Regan^5^, PhD

##### 166.1.1.1 ^1^LexaGene; ^2^Director, Applications and Assay Development, LexaGene; ^3^Senior Staff Scientist, LexaGene; ^4^Vice President of Applications, Bioinformatics, LexaGene; ^5^CEO and Founder, LexaGene

Bacterial culture and antimicrobial susceptibility testing is considered the gold standard to confirm the presence of an infection and antimicrobial resistance, but typically requires 3–5 days to obtain results. Genotypic detection of specific pathogens and their associated antimicrobial resistance genes can be used to predict antimicrobial resistance in bacterial pathogens and can deliver accurate results in hours rather than days.

This study was carried out as a proof‐of‐principle evaluation of MiQLab System to identify bacterial pathogens and their resistance genes. The MiQLab System equipped with MiQLab Bacterial and AMR test V2 can detect 10 common bacterial pathogens infecting cats and dogs and resistance genes for common first‐line veterinary antimicrobials such as beta‐lactams, sulphonamide‐trimethoprim combinations, lincosamides and tetracyclines.

The study evaluated the ability of the MiQLab to detect bacterial pathogens and resistance genes in highly characterised bacterial isolates with available genetic information. The overall percent agreement for bacterial identification between the MiQLab and bacterial culture was 100%. Similarly, the overall percent agreements between antimicrobial resistance gene detection on the MiQLab and genetic sequence data was 96.0%.

These results indicate that MiQLab's qPCR‐based genotypic detection technology can be used to identify bacterial pathogens and their associated antimicrobial resistance genes accurately with a rapid turn‐around time.

## 167 Abstract ID05

### 167.1 Evaluation of Anti‐Histoplasma Antibody Detection via Enzyme Immunoassay or Immunodiffusion in Cats and Dogs

#### 167.1.1 **Andrew S. Hanzlicek** ^1^; Timmons Delicia^2^, RVT; Durkin Michelle^3^; Laura Nafe^4^, DVM, MS, DACVIM (SAIM); Tims Rebecca^5^, DVM; Joseph Wheat^6^, MD

##### 167.1.1.1 ^1^MiraVista Diagnostics; ^2^Oklahoma State University; ^3^Senior Scientist, MiraVista Diagnostics; ^4^Assistant Teaching Professor, Veterinary Medicine and Surgery, University of Missouri; ^5^Veterinary Specialists of North Texas; ^6^Medical Director, MiraVista Diagnostics

**Background:** Anti‐*Histoplasma* antibody detection by enzyme immunoassay (EIA) or immunodiffusion (ID) is used to support the diagnosis of histoplasmosis in cats and dogs. There is a paucity of published diagnostic performance data.

**Objectives:** The primary objective was to describe the performance of IgG EIA and ID for anti‐*Histoplasma* antibody detection in cats and dogs. A secondary aim was to describe the clinical utility of antibody detection when combined with antigen detection.

**Animals:** 51 animals with pathology proven histoplasmosis and 160 animals without histoplasmosis.

**Methods:** This is a retrospective cohort study. Results of *Histoplasma* antigen EIA at the time of diagnosis was included in extracted data. Stored serum samples were tested for anti‐*Histoplasma* antibodies by EIA and ID. Diagnostic sensitivity was calculated.

**Results:** Diagnostic sensitivity of IgG EIA was 24/30 (80.0%) in cats and 17/21 (81.0%) in dogs. Diagnostic sensitivity of ID was 0/30 (0%) in cats and 3/21 (14.3%) in dogs. Diagnostic sensitivity of *Histoplasma* antigen in urine was 28/30 (93.3%) in cats and 19/21 (90.5%) in dogs. When interpreted in parallel, combined diagnostic sensitivity of antigen EIA and IgG EIA was 51/51 (100%) in both species. In animals not diagnosed with histoplasmosis, anti‐*Histoplasma* IgG antibodies were detected in 1/21 (4.8%) of cats and 10/138 (7.2%) of dogs.

**Conclusions and Clinical Importance:** Immunodiffusion is not recommended for anti‐*Histoplasma* antibody detection in cats and dogs. Anti*‐Histoplasma* antibody detection by EIA should be considered with suspected histoplasmosis but no detectable *Histoplasma* antigen in urine.

## 168 Abstract ID06

### 168.1 High Frequency of the Benzimidazole Resistance Genetic Marker in the Pet Dog Population in Florida

#### 168.1.1 **Christian M. Leutenegger** ^1^; Cecilia Lozoya^2^; Jeffrey Tereski^3^; Jennifer Ogeer^4^, BSc, DVM, MSc, MBA, MA; Rene Lallier^1^, DVM, MBA

##### 168.1.1.1 ^1^Antech Diagnostics; ^2^Manager Molecular Diagnostics, Antech Diagnostics; ^3^Molecular Diagnostics Technician, Antech Diagnostics; ^4^VP Medical Science and Innovation, Antech Diagnostics

**Background:** Three single nucleotide polymorphisms (SNP) in the highly conserved beta‐tubulin gene confer Benzimidazole resistance in many nematode parasites of veterinary and human importance. The F167Y SNP is associated with *Ancylostoma caninum* benzimidazole resistance in Greyhounds and other dog breeds.

**Objective:** A F167Y allele‐specific real‐time PCR (qPCR) was adopted and validated to screen hookworm positive samples.

**Animals:** Ninety‐two fecal samples that tested positive for *Ancylostoma* by routine ova and parasite (O&P) and confirmed by *Ancylostoma* qPCR were included and screened for the presence of the 167Y resistance marker.

**Methods:** The *Ancylostoma* O&P positive fecal samples were verified by *Ancylostoma* specific qPCR. The 167Y resistance allele‐specific qPCR was used to determine the frequency of Benzimidazole resistance genetic marker.

**Results:** The 167Y allele resistance marker was detected in 25 of the 87 (28.7%) *Ancylostoma* O&P and qPCR positive samples. Resistant allele positive samples were confirmed by beta‐tubulin gene sequencing using flanking primers. No breed information was available from 10 samples; two samples were from a mixed breed and German Shepherd Dog, respectively. One sample was represented by each of the following breeds: Boxer, Golden doodle, Yorkshire terrier, Labrador retriever, Basenji, Siberian husky, American Staffordshire terrier, Beagle, Australia cattle dog, poodle and dachshund, respectively.

**Conclusions and Clinical Importance:** Anthelmintic resistance in hookworms is an emerging concern in veterinary medicine. Routine identification of the benzimidazole resistance genetic marker for *Ancylostoma* positive samples will aid in monitoring the prevalence of hookworm resistance and provide veterinarians with information that guides appropriate treatment decisions.

## 169 Abstract ID07

### 169.1 Comparison of qPCR and Centrifugal Flotation for the Detection of Ancylostoma in Experimentally Infected Beagles

#### 169.1.1 **Christian M. Leutenegger** ^1^; Patricia Lopes Sicupira Franco^2^, BVSc, MSc, DSc; Jennifer Hawley^3^; Cecilia Lozoya^1^; Jeffrey Tereski^4^; Lindsay Starkey^5^, DVM, PhD, ACVM‐Parasitology; Jamie Butler^6^; Jennifer Ogeer^7^, BSc, DVM, MSc, MBA, MA; Rene Lallier^1^; Byron Blagburn^8^, PhD; Michael Lappin^9^, DVM, PhD, DACVIM

##### 169.1.1.1 ^1^Antech Diagnostics; ^2^Research Associate, Translational Medicine Institute, Colorado State University; ^3^Research Coordinator, Center for Companion Animal Studies, Colorado State University; ^4^Molecular Diagnostics Technician, Innovation, Antech Diagnostics; ^5^Assistant Professor, Pathobiology, Auburn University; ^6^Pathobiology, Auburn University; ^7^VP Medical Science and Innovation, Innovation, Antech Diagnostics; ^8^University Distinguished Professor, Pathobiology, Auburn University; ^9^Kenneth W. Smith Professor in Small Animal Clinical Veterinary Medicine, Small Animal Internal Medicine, Colorado State University

**Background:** Ova and parasite (O&P) by centrifugal flotation is a common method for the detection of hookworm eggs in fecal samples. Molecular diagnostics using real‐time quantitative PCR (qPCR) is a highly sensitive method to detect nucleic acids of infectious agents, including parasites.

**Objective:** This study compared O&P with qPCR for the detection of *Ancylostoma caninum* in experimentally and *Ancylostoma* spp. in naturally infected dogs and cats.

**Animals:** Five beagles treated with an anthelmintic drug at day minus 10 were experimentally infected on day 0 with 300 *Ancylostoma caninum* infective larvae. Fecal samples were collected at regular intervals.

**Field Samples:** A total of 790 fecal samples were included in the comparison of O&P with qPCR to detect *Ancylostoma* spp.

**Methods:** Fecal samples from experimentally infected beagles were analyzed within 48 hours using O&P zinc sulfate centrifugation and flotation and qPCR for *Ancylostoma*. The O&P *Ancylostoma* positive field samples were tested for *Ancylostoma* spp. by qPCR.

**Results:** In the experimentally infected beagles, all five dogs tested qPCR positive by day 17 whereas all five dogs tested positive by O&P by day 28. In the field samples, overall agreement was 97.5% between O&P and qPCR.

**Conclusions and Clinical Importance:** The high correlation of O&P *Ancylostoma* positive samples with qPCR confirms the high sensitivity of this molecular diagnostic assay. In experimentally infected dogs, qPCR indicates earlier and more consistent detection of *Ancylostoma caninum* eggs in feces than O&P.

## 170 Abstract ID08

### 170.1 Risk Factors for Acute Adverse Events in Dogs Following Vaccination

#### 170.1.1 **George E. Moore** ^1^; Jo Ann Morrison^2^, DVM, MS, DACVIM (SAIM); Nathaniel Spofford^3^; Mike Yang^3^

##### 170.1.1.1 ^1^Purdue University; ^2^Director, Veterinary Science, Banfield Pet Hospital; ^3^Banfield Pet Hospital

**Background:** Immediate‐type hypersensitivity reactions and other acute adverse events have been documented in dogs following vaccination. Risk factors have been investigated >15 years ago, but changes in vaccines or shifting genetic profiles may have altered risk factors.

**Objectives:** Determine incidence rates and potential risk factors for adverse events (AEs) recorded within 3 days of vaccine administration in dogs.

**Animals:** 4,954,565 dogs vaccinated at 21,103,149 office visits at Banfield Pet Hospitals.

**Methods:** Electronic medical records from 2016–2020 were used in a retrospective cohort study identifying all dogs receiving vaccinations and documenting potential vaccine‐associated AEs within 3 days following vaccination. Incidence rates for AEs with 95% CIs were calculated, and associations for potential risk factors were assessed by logistic regression.

**Results:** The overall documented AE rate was 0.001845 (0.1845%, or 18.45 AE/10,000 dogs), or <1/500. The 5 breeds (n>10,000) with highest AE rates were Dachshund, Boston terrier, miniature Pinscher, French Bulldog, and Havanese (68.62, 63.28, 53.73, 49.28, and 35.82 AE per 10,000, respectively). Increasing the number of vaccines administered increased AE risk in dogs ≤20 kg (OR 1.23; 95%CI: 1.22–1.24) but not in dogs >20 kg (OR 1.01; 95% CI: 0.99–1.02). AE rates for DAPP, leptospirosis, rabies, or Borrelia vaccines administered alone were similar (19.22–21.31 AE/10,000).

**Conclusions and Clinical Importance:** Small dogs receiving multiple vaccines per office visit remain at increased risk of AEs following vaccination, but AE rate by breed suggests genetics may be the greater risk factor for an acute AE.

## 171 Abstract ID09

### 171.1 Molecular Identification of Hemoparasites of Dogs in the Western Amazon

#### 171.1.1 **Acácio D. Pacheco** ^1^; Siham Kassab^2^, DVM, MsC; Emerson Dankar^3^; Cintia Daudt^4^; João Fábio Soares^5^, DVM, PhD; Soraia Souza^6^, DVM, PhD; Flavio Silva^7^, DVM, PhD

##### 171.1.1.1 ^1^Universidade Federal do Acre; ^2^Anesthesiology Resident, Department of Veterinary Medicine, UFRGS; ^3^Veterinary; ^4^Center for Biological and Natural Sciences, UFAC/Rio Branco; ^5^Adjunct Professor, Department of Veterinary Medicine, UFRGS; ^6^Adjunct Professor, Department of Veterinary Medicine, Federal University of Paraná; ^7^Adjunct Professor, Center for Biological and Natural Sciences, UFAC/Rio Branco

**Background:** Canine hemoparasitosis is a set of diseases caused by intracellular microorganisms, such as bacteria and protozoa, transmitted mainly by ticks. Its diagnosis is usually made based on the observation of suggestive hematological changes such as anemia, thrombocytopenia, and leucopenia, however, tests with greater sensitivity are recommended, such as serology and PCR. Despite the wide distribution, there are few reports on the prevalence in the North, more specifically in the Western Amazon.

**Hypothesis/Objectives:** The present study aimed to assess the prevalence of *Ehrlichia* spp., *Babesia* spp., and *Anaplasma* spp. in thrombocytopenic dogs or with inclusions suggestive of hemoparasites in the municipality of Rio Branco, Acre.

**Methods:** Blood samples were collected from 70 dogs with suggestive inclusions of hemoparasites or thrombocytopenic conditions and submitted to hematological analysis, SNAP 4DX test, and Polymerase Chain Reaction for piroplasmas and agents of the family Anaplasmataceae.

**Results:** The serological evaluation was considered reactive in 91.43% of the dogs, while the PCR resulted in 67.14% of positivity, considering one or more agents. Infection by *Ehrlichia canis* corresponded to the largest number of cases with 61.43% of the total, followed by 14.28% for *Babesia canis vogeli* and 2.85% for *Anaplasma platys*. Eight cases of co‐infection with *E. canis* and *B. vogeli* were confirmed.

**Conclusion:** With the present study it was possible to conclude that the infection by *E. canis* was more prevalent in the evaluated population and that the association between the diagnostic methods is essential to confirm the cases.

## 172 Abstract ID10

### 172.1 Proteinuria in Dogs with Pulmonary Coccidioidomycosis

#### 172.1.1 **Laura H. Rayhel** ^1^; Mark Acierno^2^, DVM, MBA, DACVIM (SAIM); Andrew Hanzlicek^3^; Jessica Hokamp^4^, DVM, PhD, DACVP (Clinical); Jared Jaffey^5^, DVM, MS, DACVIM (SAIM)

##### 172.1.1.1 ^1^Midwestern University Companion Animal Clinic; ^2^Professor, Small Animal Internal Medicine, Midwestern University Companion Animal Clinic; ^3^Director, Veterinary Medicine and Research, Mira Vista Veterinary Diagnostics; ^4^Assistant Professor, Veterinary Biosciences, The Ohio State University;^5^Assistant Professor, Small Animal Internal Medicine, Midwestern University Companion Animal Clinic

**Background:** Immune‐complex glomerulonephritis occurs secondary to multiple infectious diseases. It is unknown whether dogs with pulmonary coccidioidomycosis develop evidence of glomerular damage.

**Hypothesis:** Dogs with pulmonary coccidioidomycosis will exhibit evidence of glomerular damage on minimum database, urine protein:creatinine ratio (UPC), and urine sodium dodecyl sulphate–polyacrylamide gel electrophoresis (uSDS‐PAGE) tests at diagnosis.

**Animals:** Client‐owned dogs with pulmonary coccidioidomycosis were included if: >1 respiratory sign, consistent thoracic radiographic or computed tomographic abnormalities, and >1 positive diagnostic test for coccidioidomycosis. Dogs with non‐coccidioidomycosis causes for proteinuria were excluded.

**Methods:** Serum biochemistry, urinalysis, UPC, and uSDS‐PAGE were performed at diagnosis, within seven days of starting commercial azole antifungal therapy.

**Results:** Thirty‐one dogs (16 female, 11 spayed; 15 male, 12 castrated; median age 4.8 years; median weight 22.9 kg) were enrolled. One was azotemic (creatinine 4.5, BUN 51), but urine specific gravity (USG) indicated pre‐renal azotemia (1.058). Median creatinine was 0.9 (range 0.3–4.5) and median BUN was 14 (6–51). Complete urinalyses were available for 30 dogs; none had active sediment. Median USG was 1.041 (1.016–1.058). UPC and uSDS‐PAGE were available for 27 dogs. Median UPC was 0.17 (0.06–1.81); 18.5% had UPC >0.5 and 11.1% >1. On uSDS‐PAGE, 14/27 (51.9%) had none‐minimal, 4/27 (14.8%) minimal‐mild, 6/27 (22.2%) mild, 2/27 (7.4%) mild‐moderate, and 1/28 (3.7%) moderate glomerular damage. 21/27 (77.8%) had none, 5/27 (18.5%) none‐minimal, and 1/27 (3.7%) minimal tubular damage.

**Conclusions:** A subset of dogs with pulmonary coccidioidomycosis have evidence of glomerular damage at diagnosis.

## 173 Abstract ID11

### 173.1 Differentiating *Giardia duodenalis* Assemblages with a Novel Beta‐Giardin PCR Assay

#### 173.1.1 **Andrea V. Scorza** ^1^; Christian Leutenegger^2^, Dr.Med.Vet., BSc, PhD, FVH; Cecilia Lozoya^3^; Jeffrey Tereski^3^; Michael Lappin^1^

##### 173.1.1.1 ^1^Colorado State University; ^2^Director Molecular Diagnostics, R&D, Antech Diagnostics; ^3^Antech Diagnostics

**Background:** *Giardia duodenalis* is a common pathogen of dogs and cats, and it comprises several genotypes (assemblages A‐G). The common assemblages affecting small animals (C, D, F) are not associated with disease in humans. However, Assemblages A and B that are uncommon in small animals can be zoonotic. Several assays can be used for detection of *G. duodenalis*, but there is a need for an accurate and faster diagnosis of *G. duodenalis* zoonotic assemblages.

**Objective:** The aim of this study was to validate a beta‐giardin gene real‐time PCR (bg‐qPCR) for detection of *G. duodenalis* zoonotic assemblages A and B in dog and cat samples.

**Animals:** 161 canine and feline fecal samples submitted to Antech Diagnostics for O&P with *Giardia* ELISA were additionally analyzed by quantitative PCR (qPCR).

**Methods:** Samples that tested positive for *G. duodenalis* by O&P, *Giardia* ELISA, and qPCR were used to validate the bg‐qPCR. *Giardia* qPCR positive samples were confirmed by gene sequencing. Bg‐qPCR results were confirmed using the flanking primers of the assay.

**Results:** Of 161 *Giardia* confirmed positive fecal samples, *G. duodenalis* genotypes A or B were identified in 6 samples (3.7%) by qPCR. The A (3 samples) and B (3 samples) assemblages were confirmed by bg‐PCR. Two of the three B assemblage samples typed as BIII by bg‐qPCR, which is a human zoonotic sub‐assemblage.

**Conclusions and Clinical Importance:** In this small sample set, Bg‐qPCR accurately identified the potentially zoonotic *Giardia duodenalis* A/B assemblages and provides a new method for determining zoonotic risk.

## 174 Abstract ID12

### 174.1 Evaluation of Leptospira Exposure in Feral Cat Populations in Northern California and Southern Texas

#### 174.1.1 **Jamie F. Sebastian** ^1^; Krystle Reagan^2^, DVM, PhD, DACVIM; Tess Peavy^3^, DVM; Italo Zecca^4^, MPH, PhD; Sarah Hamer^4^, DVM, MS, PhD; Jane Sykes^2^, BVSc (Hons), PhD, MBA, DACVIM

##### 174.1.1.1 ^1^Veterinary Medical Teaching Hospital, University of California‐Davis; ^2^Department of Medicine and Epidemiology, University of California‐Davis; ^3^Public Vet Inc.; ^4^College of Veterinary Medicine & Biomedical Sciences, Texas A&M University; ^2^Department of Medicine and Epidemiology, University of California‐Davis

**Background:** Leptospirosis is a re‐emergent zoonotic bacterial infection that causes renal and hepatic injury. Reservoir hosts maintain a carrier state in endemic regions. Feral cats in some areas have high prevalence of *Leptospira* antibodies and leptospires in renal tissue. This finding indicates feral cats may play a role in *Leptospira* epidemiology or act as sentinels for detection of *Leptospira* in the environment.

**Hypothesis/Objectives:** Feral cats in endemic regions of the United States are exposed to and potentially infected with *Leptospira*.

**Animals:** Feral cats from northern California (n=52) and southern Texas (n=75).

**Methods:** Whole blood, sera, and urine specimens were collected from cats in California, October 2020. Sera was collected from feral cats in Texas throughout 2017. Microagglutination tests (MAT) (n=127) and IDEXX SNAPLepto tests (n=106) were performed on cat sera. MAT serogroups tested included Icterohaemorrhagiae, Canicola, Hardjo, Bratislava, Grippotyphosa, and Pomona. *Leptospira* PCR was performed on whole blood and urine specimens from California cats.

**Results:** MAT was positive in 17.3% (9/52) California cats and 8.0% (6/75) Texas cats (p<0.05). The median (range) positive cat MAT titers were 1:100(1:100–1:200) in California cats and 1:200(1:100–1:800) in Texas cats. The highest serogroup MAT titers included Icterohaemorrhagiae (8/15) and Hardjo (7/15). All SNAP test and *Leptospira* PCR results were negative.

**Conclusions and Clinical Importance:** Feral cats mount antibodies to *Leptospira* and may act as sentinel hosts. No cats had evidence of active infection. Cats in these regions are exposed to *Leptospira*, but their role in maintenance or shedding of Leptospires requires further investigation.

## 175 SMALL ANIMAL INTERNAL MEDICINE ‐ NEPHROLOGY/UROLOGY

## 176 Abstract NU01

### 176.1 Evaluation of Creatinine, Symmetric Dimethylarginine, Kidney Injury Molecule‐1 and Glomerular Filtration Rate in Healthy Cats

#### 176.1.1 **Aleksandra Milaszewska**; Alice Defarges, DVM, DACVIM (SAIM); Xiu Ting Yiew, DVM, DACVECC; Michelle Oblak, DVM, DACVS; Briggitte Brisson, DVAm, DACVS; Dorothee Bienzle, DVM, PhD, DACVP

##### 176.1.1.1 University of Guelph

**Background:** Current serum and urine markers are poorly sensitive for detecting acute kidney injury (AKI) in cats.

**Hypothesis/Objectives:** To compare changes in glomerular filtration rate (GFR), kidney injury molecule‐1 (KIM‐1), serum creatinine (sCr), and serum symmetric dimethylarginine (SDMA) in healthy cats associated with general anesthesia (GA).

**Animals:** Nine purpose‐bred male cats (mean age 12 months, range 11–¬16), deemed healthy based on physical exam, blood analysis, and urinalysis.

**Methods:** Cats were placed under GA (isoflurane) twice for 180 minutes, on average eight days apart (range 4¬–13 days). Baseline GFR, KIM¬–1, sCr, SDMA were measured prior to the first GA, then 24 hours after each GA, and again four days after the last GA. The GFR was calculated from clearance of injected exogenous creatinine. Acute injury was defined as a decrease in GFR by ³25%.

**Results:** Mean GFR at baseline was 2.46 mL/kg/min; range 1.17¬–5.75. Six of nine cats experienced GFR decrease of ³25% associated with 10 of 18 GAs. KIM‐1 increased in 6 of 9 cats (mean 0.408, range 0.027–0.254), while sCr increased by ³26.4 μmol/L in one cat. SDMA concentration was increased by ³3 μg/dL in 2 of 9 cats.

**Conclusions and Clinical Importance:** A decrease in GFR and increase in urine KIM‐1 is relatively frequent in cats anesthetized for 180 minutes, while an increase in sCr and SDMA is less frequent. These findings suggest that urine KIM‐1 may be a sensitive biomarker to detect reduced GFR, and therefore AKI in clinically ill cats.

## 177 Abstract NU02

### 177.1 Medical Dissolution of Presumptive Upper Tract Struvite Urolithiasis in 6 Dogs: (2012–2018)

#### 177.1.1 **Sindumani Manoharan** ^1^; Allyson Berent^2^, DVM, DACVIM (SAIM); Chick Weisse^3^, VMD, DACVS‐SA; Kira Purdon^4^, DVM; Demetrius Bagley^5^, MD

##### 177.1.1.1 ^1^University of Pennsylvania; ^2^Specialist in Internal Medicine, Co‐Director of The Katharine and William Rayner Interventional Radiology and Endoscopy Service, The Animal Medical Center; ^3^Specialist in Surgery, Service Head of Interventional Radiology and Endoscopy, The Animal Medical Center; ^4^Veterinarian, The Animal Medical Center; ^5^Nathan Lewis Hatfield Professor of Urology, Thomas Jefferson University

**Background:** Medical management of struvite uroliths is well documented. Dissolution of struvite nephroureterolithiasis has not specifically been reported in dogs.

**Objective:** Describe the medical dissolution of upper tract uroliths and clinical outcomes in a series of dogs.

**Animals:** 6 client‐owned dogs.

**Methods:** A retrospective review of medical records of dogs that underwent medical dissolution of upper tract uroliths utilizing dissolution diet, antibiotic therapy, and double pigtail ureteral stent(s) placement, when indicated, was performed. Medical management was continued for 4 weeks beyond stone dissolution. Information on pre‐ and post‐dissolution biochemical, microbiological, imaging, and clinical outcomes were recorded.

**Results:** Six female dogs (9 renal units) with bilateral (3) or unilateral (3) nephro‐ and/or ureterolithiasis were included. The median age and weight was 5 years (range, 4–10) and 7.41 kg (range, 3.42–12.86), respectively. All had a positive urine culture of *Staphylococcus pseudintermedius* with a median urine pH of 7.25. A ureteral stent(s) was placed endoscopically in 5/6 dogs (5/9 ureters) for obstructive ureterolithiasis or a non‐obstructive massive nephrolith to prevent potential obstruction post‐dissolution. Four dogs had evidence of concurrent pyonephrosis. All dogs had initial evidence of stone dissolution at a median of 1.1 months (range, 0.42–5.9), with complete dissolution of ureteroliths, nephroliths, and cystoliths at a median of 3.9 (range, 1.5–7.6), 5.3 (range, 1.5–7.6), and 0.87 months (range, 0.42–5.9), respectively. The median follow‐up time was 519 days (range, 177–2492 days).

**Conclusion and Clinical Importance:** Medical dissolution, +/‐ decompression, should be considered as a minimally invasive treatment option for dogs with suspected struvite nephroureterolithiasis prior to considering more invasive options.

## 178 Abstract NU03

### 178.1 A Pilot Study Evaluating Renal Biomarker Changes Following Intravenous Iohexol Administration in a Small Population of Beagles

#### 178.1.1 **Edward J. Vasquez** ^1^; Laura Van Vertloo^2^, DVM, MS, DACVIM; Jean‐Sebastien Palerme^2^, DVM, MSc, DACVIM

##### 178.1.1.1 ^1^ISU; ^2^Assistant Professor of Small Animal Internal Medicine, Small Animal Internal Medicine, Iowa State University

**Background:** Despite being one of the most common causes of hospital‐acquired acute kidney injury (AKI) in people, contrast‐induced nephropathy (CIN) remains poorly studied in veterinary medicine. Novel biomarkers have been shown to be more sensitive than creatinine for the detection of CIN in people. However, no data exists documenting changes in renal biomarkers in dogs receiving intravenous contrast media.

**Hypothesis/Objectives:** To characterize changes in concentrations of serum creatinine, cystatin C, symmetric dimethylarginine (SDMA), and both serum and urinary neutrophil gelatinase‐associated lipocalin (NGAL) in a small population of healthy sedated beagles receiving intravenous contrast.

**Animals:** 6 healthy purpose‐bred beagles.

**Methods:** Randomized cross‐over study. On day 1, dogs underwent either 15‐minute sedation (0.2 mg/kg butorphanol and 5 mcg/kg dexmedetomidine) or 15‐minute sedation with intravenous iohexol administration (2.2 ml/kg), with blood and urine samples collected at baseline, 4 hours, and 72 hours post‐intervention for measurement of serum creatinine, BUN, NGAL, and cystatin C, as well as urinary NGAL. Treatment groups were reversed on Day 5. Analysis was performed using a generalized linear mixed‐effects model with significance set at p<0.05.

**Results:** Serum creatinine, SDMA, cystatin C, NGAL and urinary NGAL of dogs receiving iohexol (mean values: 0.62 mg/dl, 10.7 μg/dl, 121.97 pg/ml, 20946 pg/ml, 233.01 pg/ml, respectively) did not differ significantly from control dogs and did not increase significantly from baseline at 4 or 72 hours post‐intervention.

**Conclusions and Clinical Importance:** Administration of a standard dose of iohexol to healthy sedated dogs resulted in neither AKI nor increases in novel renal biomarkers.

## 179 Abstract NU04

### 179.1 Proteinuria at Time of Diagnosis is Associated with Shorter Survival Time in Dogs with Lymphoma

#### 179.1.1 **Stephanie Skinner** ^1^; Andrew Specht^2^, DVM, DACVIM (SAIM); Victoria Cicchirillo^3^, DVM; Stacey Fox‐Alvarez^4^, DVM, DACVIM (Medical Oncology); Autumn Harris^5^, DVM, DACVIM (SAIM)

##### 179.1.1.1 ^1^University of Florida; ^2^Clinical Professor, Small Animal Clinical Sciences, University of Florida; ^3^Resident, Oncology, Small Animal Clinical Sciences, University of Florida; ^4^Clinical Assistant Professor, Small Animal Clinical Sciences, University of Florida; ^5^Assistant Professor, Small Animal Clinical Sciences, University of Florida

**Background:** Lymphoma has been implicated as a possible cause of proteinuria in dogs. However, information about the potential significance of proteinuria in these patients is limited.

**Objectives:** The primary objective was to determine if presence of proteinuria at diagnosis was associated with survival times in dogs with lymphoma. A secondary objective was to determine if lymphoma stage (I‐V) or type (B vs T) were associated with presence of proteinuria.

**Animals:** Records of 476 dogs with a new diagnosis of lymphoma (by cytology, histopathology, or PCR for antigen receptor rearrangement) between 2008–2020 were reviewed. Inclusion required concurrent serum chemistry and urine protein assessment. Dogs were excluded for: 1) treatment within two months with, steroid, anti‐neoplastic or anti‐proteinuric therapies, 2) diagnosed hypercortisolism, 3) urine pH >8, or 4) active urine sediment. Data from 86 dogs was analyzed.

**Methods:** This was a retrospective cohort study with dogs divided into proteinuric (22/86) or non‐proteinuric (64/86) groups based on urine protein of ≥ or <30 mg/dL at time of diagnosis. Stage and type information was available for 81 and 49 dogs respectively.

**Results:** There was a significant difference in survival between proteinuric (233±39 days) and non‐proteinuric (437±60 days) groups (p<0.04; Log‐Rank). No difference in prevalence of proteinuria was identified based on stages I‐V or types B and T.

**Conclusions and Clinical Importance:** Further evaluation of the association between presence of proteinuria and shorter survival time and whether it could be ameliorated by directed treatment efforts is important.

## 180 Abstract NU05

### 180.1 Evaluation of Health‐Related Quality of Life in Cats with Chronic Kidney Disease

#### 180.1.1 **Sarah K. Lorbach** ^1^; Jessica Quimby^2^, DVM, PhD, DACVIM; Eline Nijveldt^3^, DVM; Rene Paschall^3^, DVM; Jacky Reid^4^, BVMS, PhD, DVA, DECVAA, MRCA, MRCVS

##### 180.1.1.1 ^1^The Ohio State University; ^2^Associate Professor, Veterinary Clinical Sciences, Ohio State University; ^3^Intern, Veterinary Clinical Sciences, Ohio State University; ^4^Professor, University of Glasgow

**Background:** A validated generic health‐related quality of life (HRQoL) tool exists for cats in which the healthy population average is a score of 50, and 70% of healthy cats score 44.8 or higher.

**Hypothesis/Objectives:** Cats with CKD will have decreased HRQoL scores and specific clinical factors will negatively impact their score.

**Animals:** 68 cats with CKD.

**Methods:** Owners completed a validated generic HRQoL tool (VetMetrica™) and clinical data (history, labwork, physical exam findings, etc.) were collected. Vitality, comfort, and emotional well‐being (EWB) scores were compared between variables with non‐parametric analyses as appropriate.

**Results:** Lower EWB scores were associated with several clinical factors (Table 1). In addition, cats with IRIS stage 3&4 CKD had lower vitality scores (median 38.2, range 4.3–54.0) than IRIS stage 1&2 (43.1, 12.8–64.0) (P=0.04). Cats with constipation had lower comfort scores (29.5, 21.2–59.5) than those with reportedly normal stools (37.1, 25.3–59.6) (P=0.0003). Hematocrit was positively correlated with EWB (P=0.005, r=0.33). Number of medications administered was negatively correlated with EWB (P=0.03, r=‐0.25).

**Table:** Table 1

	EWB score Median (range)	P value
**IRIS Stage**		
1&2	47.3 (11.2–58.8)	0.04
3&4	30.8 (1.6–54.4)	
**Anemia**		
Yes	25.9 (10.4–54.4)	0.005
No	46.6 (1.6–58.8)	
**Appetite**		
Abnormal	29.1 (1.6–56.7)	0.001
Normal	48.1 (11.2–58.8)	
**Constipation**		
Yes	22 (1.6–58.8)	0.0001
No	47.9 (11.2–58.8)	
**Muscle condition score**		
Normal/Mild	48 (11.2–58.8)	0.008
Moderate/Severe	35.5 (1.6–56.7)	

**Conclusions and Clinical Importance:** Several clinical factors are associated with decreased HRQoL and imply that addressing these abnormalities may improve wellbeing in CKD cats.

## 181 Abstract NU06

### 181.1 Safety and Efficacy of Nightly, Prophylactic Nitrofurantoin in 14 Dogs with Recurrent Urinary Tract Infections

#### 181.1.1 **Olivia Murray** ^1^; Eva Furrow^2^, VMD, PhD, DACVIM (SAIM); Jennifer Granick^2^, DVM, PhD, DACVIM (SAIM); Lindsay Merkel^2^, DVM, DACVIM (SAIM); Jody Lulich^2^, DVM, PhD, DACVIM (SAIM)

##### 181.1.1.1 ^1^University of Minnesota; ^2^College of Veterinary Medicine, University of Minnesota;

**Background:** Prophylactic antimicrobials for recurrent urinary tract infections (rUTIs) are an important preventative option for at‐risk humans. Data is needed to determine if prophylactic antimicrobial therapy is also safe and efficacious for rUTIs in dogs.

**Objectives:** To report efficacy and adverse events in dogs receiving single‐dose, nightly nitrofurantoin therapy as antimicrobial prophylaxis for rUTIs.

**Animals:** Fourteen client‐owned dogs.

**Methods:** Retrospective case series. Data on urinary history, diagnostic investigation, protocol, adverse events, and efficacy (through serial urine cultures) were extracted from medical records.

**Results:** Prior to therapy, dogs with rUTIs averaged 0.8 positive urine cultures per month. After standard antimicrobial therapy for a UTI, nitrofurantoin was prescribed as a single‐dose, nightly therapy (median dosage: 4.0 mg/kg PO q24 hours) for a median duration of 153 days. Nine dogs had no positive urine cultures while on therapy. Of these, 5 (3 which discontinued nitrofurantoin and 2 which remained on nitrofurantoin) had no return of clinical signs or bacteriuria at time of death or last follow‐up evaluation at a median of 238 days (range 35–1406) after institution of therapy, and 4 had suspected or confirmed bacteriuria 10–21 days post discontinuation. Five dogs developed bacteriuria while on therapy, 4 of which were nitrofurantoin‐resistant *Proteus* spp. Most adverse events were minor and non‐specific; none were definitively attributable to the drug.

**Conclusions:** Single‐dose, nightly nitrofurantoin therapy appears well tolerated and might be efficacious for rUTIs in dogs.

## 182 Abstract NU07

### 182.1 Evaluation of the IDEXX SediVue Dx® for Identification of Canine and Feline Bacteriuria

#### 182.1.1 **Emily Belshin**; Sheri Ross, DVM, PhD, DACVIM (Internal Medicine); Cedric Dufayet, DVM

##### 182.1.1.1 Department of Nephrology, Urology, and Hemodialysis, Veterinary Medical Center, University of California, San Diego, CA, USA

**Background:** Detection of bacteriuria is important when evaluating patients with lower urinary tract symptoms. There is a need for accurate and rapid testing to confirm the presence of bacteria in urine samples.

**Hypothesis/Objective:** The IDEXX SediVue Bacteria Confirmation Kit™ will increase the specificity of SediVue Dx® bacteriuria detection system.

**Animals:** 122 client‐owned dogs and cats presenting to the nephrology/urology service.

**Methods:** Patients requiring urinalysis and urine culture as part of their diagnostic evaluation were included in the study. Urine samples obtained between Nov 2020 and Nov 2021 analyzed on IDEXX SediVue Dx® and submitted for quantitative urine culture were included. Samples were recorded as positive or negative for bacteria by the SediVue. Samples with “suspect presence” of bacteria were further evaluated using the SediVue bacteria confirmation kit™ as prompted by the SediVue. All urine samples were submitted to an external lab for quantitative urine culture.

**Results:** 197 urine samples from 103 dogs and 19 cats, of all sexes and breeds were evaluated. Sensitivity of the SediVue analysis for initial detection of bacteria was 85.5%, specificity was 70.2%, positive predictive value (PPV) was 64.4%, and negative predictive value (NPV) was 88.5%. After prompted bacterial confirmation, revised sensitivity/specificity was 81.6% and 90.9% and PPV/NPV was 84.9% and 88.7%.

**Conclusions and Clinical Importance:** When compared to quantitative urine culture, the addition of the IDEXX bacteria confirmation kit™ to the SediVue Dx® increases both specificity and PPV when determining the presence of bacteria in a urine sample.

## 183 Abstract NU08

### 183.1 Altered Serum Amino Acid Concentrations in Dogs with Chronic Kidney Disease

#### 183.1.1 **Amanda B. Blake** ^1^; Elijah Ernst^2^; Jonathan Lidbury^3^; Joerg Steiner^3^; Jan Suchodolski^3^; M. Katherine Tolbert^3^

##### 183.1.1.1 ^1^Gastrointestinal Lab, Texas A&M University; ^2^Clinical Sciences, College of Veterinary Medicine, North Carolina State University; ^3^Veterinary Small Animal Clinical Sciences, Gastrointestinal Lab, Texas A&M University

**Background:** Amino acids (AA) serve as markers of nutritional status as well as indicators of kidney function (e.g., SDMA). Altered AA concentrations are observed in dogs and humans with chronic kidney disease (CKD).

**Objectives:** Compare serum AA concentrations between dogs with CKD and healthy control dogs (HCDs).

**Animals:** Serum was collected from dogs with CKD (n=9) and age and weight‐matched HCDs (n=8).

**Methods:** Prospective case‐control study. All dogs consumed a therapeutic renal diet for 3 days prior to sampling. Serum AA concentrations were measured with an AA analyser (Biochrom 30+) with post‐column ninhydrin derivatization. AA concentrations were compared between groups with Mann‐Whitney tests and corrected for multiple comparisons using Benjamini‐Hochberg method.

**Results:** Serum concentrations of tyrosine (*Q*=0.027), and tryptophan (*Q*=0.041) were significantly lower in dogs with CKD compared to HCDs. Serum concentrations of citrulline (*Q*=0.041), 1‐methylhistidine (1MH, *Q*=0.041), and carnosine (*Q*=0.014) were significantly higher in dogs with CKD compared to HCDs. The 3‐methylhistidine (3MH):1MH ratio was higher in some dogs with CKD (*Q*=0.078).

**Conclusions:** Serum concentrations of proteinogenic AA are lower in dogs with CKD. Concentrations of AA that are indicators of muscle catabolism were higher in dogs with CKD, as was 3MH:1MH ratio in some dogs. This ratio serves as an indicator of muscle protein degradation after accounting for reduced renal clearance and might be a possible predictor of muscle loss.

## 184 Abstract NU09

### 184.1 Outcomes of Non‐Steroidal Anti‐Inflammatory Drug Toxicosis treated with Therapeutic Plasma Exchange in 62 dogs.

#### 184.1.1 **Nolan Chalifoux** ^1,2^, DVM; Emmanuelle Butty^3^, MV, DACVIM (SAIM); Katie Mauro^4^, DVM, DACVECC; Rachel Moyle^5^, DVM, DACVECC; Caryn Ehrhardt^6^, DVM; James Robertson^7^; Mary Labato^8^, DVM, DACVIM (SAIM); Christine Culler^5^, DVM, MS, DACVECC; Leonel Londoño^9^, DVM, DACVECC; Alessio Vigani^10^, DVM, PhD, DACVAA, DACVECC; Yu Ueda^11^, DVM, PhD, DACVECC; Steven Suter^12^, DVM, PhD, DACVIM; Shelly Vaden^13^, DVM, PhD, DACVIM; Alex Lynch^14^, BVSc (Hons), DACVECC, MRCVS

##### 184.1.1.1 ^1^Ryan Veterinary Hospital, University of Pennsylvania; ^2^Resident, Emergency, and Critical Care, Department of Clinical Sciences and Advanced Medicine, University of Pennsylvania; ^3^Urology and Nephrology Fellow, Cummings School of Veterinary Medicine, Tufts University; ^4^Assistant Professor of Clinical Extracorporeal Therapies, Department of Clinical Sciences and Advanced Medicine, University of Pennsylvania; ^5^BluePearl Pet Hospital; ^6^Emergency and Critical Care Resident, Department of Small Animal Clinical Sciences, College of Veterinary Medicine, University of Florida; ^7^North Carolina State University; ^8^Department of Clinical Sciences, Cummings School of Veterinary Medicine, Tufts University; ^9^Capital Vet Specialists; ^10^University of Zurich; ^11^Clinical Assistant Professor in Emergency and Critical Care, Department of Clinical Sciences, North Carolina State University; ^12^Professor of Medical Oncology, Department of Clinical Sciences, North Carolina State University; ^13^Professor of Internal Medicine (Nephrology and Urology), Department of Clinical Sciences, North Carolina State University; ^14^Assistant Professor in Emergency and Critical Care, Department of Clinical Sciences, North Carolina State University

**Background:** Therapeutic plasma exchange (TPE) is gaining popularity for the management of non‐steroidal anti‐inflammatory drug (NSAID) overdose in dogs.

**Hypothesis/Objectives:** Describe a population of dogs treated with TPE for NSAID overdose.

**Animals:** Sixty‐two dogs with NSAID overdose treated with TPE.

**Methods:** Multicenter retrospective study of dogs treated with TPE for ibuprofen, carprofen, or naproxen overdose.

**Results:** The median dose of ibuprofen, carprofen or naproxen ingested was 206 mg/kg (range, 25–4857 mg/kg), 78 mg/kg (range, 11–625 mg/kg) and 81 mg/kg (range, 8–3000 mg/kg). Two (3.2%), 14 (22.6%) and 46 (74.2%) dogs ingested a gastrointestinal, renal, and neurological toxic dose, respectively. The median time elapsed between ingestion and presentation was 4 hours (range, 1–20 hours). The median total replacement volume was 1300 mls (range, 220–3208 mls) which equated to 1.6 plasma volumes (range, 0.4–2.2). The median duration of TPE sessions was 2 hours (range, 1–4.5 hours). Adverse events were reported during 29 (46.7%) TPE sessions, comprising circuit clotting (12.9%), urticaria (12.9%), hypocalcemia (9.6%) and hypotension (9.6%). The median duration of hospitalization was 2.25 days (range 1–11 days). Sixty‐one (98.4%) dogs survived to discharge and none were re‐hospitalized. Thirty‐three (97%) of the 34 dogs with at least one follow‐up visit were not azotemic at the time of recheck.

**Conclusions and Clinical Importance:** Our TPE–treated population had an excellent outcome, despite exposure to high NSAID doses. When TPE is available and the time frame is adequate, this extracorporeal modality should be recommended in cases of severe NSAID overdose.

## 185 Abstract NU10

### 185.1 Outcomes of Fluid Therapy, Lipid Emulsion, and Therapeutic Plasma Exchange for Non‐Steroidal Anti‐Inflammatory Drug Toxicosis

#### 185.1.1 **Nolan Chalifoux** ^1^; Emmanuelle Butty^2^, MedVet, DACVIM (SAIM); Katie Mauro^3^, DVM, DACVECC; Rachel Moyle^4^, DVM, DACVECC; Caryn Ehrhardt^5^, DVM; James Robertson^6^, MA; Mary Labato^7^, DVM, DACVIM (SAIM); Christine Culler^4^, DVM, MS, DACVECC; Leonel Londoño^8^, DVM, DACVECC; Alessio Vigani^9^, DVM, PhD, DACVAA, DACVECC; Yu Ueda^10^, DVM, PhD, DACVECC; Steven Suter^11^, DVM, PhD, DACVIM; Shelly Vaden^12^, DVM, PhD, DACVIM; Alex Lynch^13^, BVSc (Hons), DACVECC, MRCVS

##### 185.1.1.1 ^1^Ryan Veterinary Hospital, University of Pennsylvania; ^2^Urology/Nephrology Fellow, Foster Hospital for Small Animals, Department of Clinical Sciences, Tufts University; ^3^Assistant Professor of Clinical Extracorporeal Therapies, Ryan Veterinary Hospital, Department of Clinical Sciences and Advanced Medicine, University of Pennsylvania; ^4^BluePearl Pet Hospital; ^5^Resident, Emergency, and Critical Care, Small Animal Hospital, Department of Small Animal Clinical Sciences, University of Florida; ^6^Research Associate, Department of Clinical Sciences, North Carolina State University; ^7^Clinical Professor of Internal Medicine, Foster Hospital for Small Animals, Department of Clinical Sciences, Tufts University; ^8^Capital Vet Specialists; ^9^Medical Director Small Animal Emergency, Critical Care, and Extracorporeal Therapy, University of Zurich; ^10^Clinical Assistant Professor in Emergency and Critical Care, Department of Clinical Sciences, North Carolina State University; ^11^Professor of Medical Oncology, Department of Clinical Sciences, North Carolina State University; ^12^Professor of Internal Medicine (Nephrology and Urology), Department of Clinical Sciences, North Carolina State University; ^13^Assistant Professor in Emergency and Critical Care, Department of Clinical Sciences, North Carolina State University

**Background:** Traditional management of non‐steroidal anti‐inflammatory drug (NSAID) intoxications includes gastrointestinal decontamination, intravenous administration of fluids (IVF), and gastroprotection. Intravenous administration of lipid emulsion (ILE) and therapeutic plasma exchange (TPE) are increasingly popular novel therapeutic strategies.

**Hypothesis/Objectives:** Compare outcomes of dogs treated with IVF, ILE, and TPE for NSAID intoxications and evaluate outcome predictors for drug subgroups.

**Animals:** Four hundred and thirty‐four dogs with NSAID intoxications (2015–2020).

**Methods:** Multicenter retrospective study of ibuprofen, carprofen, and naproxen intoxication. An ordinal outcome was defined as mild gastrointestinal, moderate kidney, or severe central nervous system signs.

**Results:** TPE and ILE were associated with more severe clinical signs throughout hospitalization (P=0.033), but either ILE or TPE were more likely to be used as the exposed dose increased (P<0.001). Dogs treated with IVF had a higher maximal creatinine concentration compared to IVF + ILE (P=0.013), though values remained normal for both groups. Increased maximum time to presentation (P<0.001), and higher baseline creatinine (P<0.001), and PCV (P=0.007) were associated with greater clinical severity; while emesis induction reduced clinical signs (P<0.001). Ibuprofen toxicosis was associated with worse clinical signs compared to carprofen (P=0.028) and maximal ibuprofen exposure dose was associated with worse clinical signs (P<0.001). Total survival rate was 99%.

**Conclusions and Clinical Importance:** NSAID toxicosis generally carries an excellent prognosis in dogs. The clinical utility of ILE and TPE in addition to IVF therapy remains to be determined.

## 186 Abstract NU11

### 186.1 Continuous Renal Replacement Therapy in Dogs with Acute Kidney Injury: Dose Prescription and Adequacy Assessment

#### 186.1.1 **Hilla Chen** ^1^; Gilad Segev^2^, DECVIM

##### 186.1.1.1 ^1^Koret School of Veterinary Medicine, The Hebrew University of Jerusalem; ^2^Koret Veterinary Hospital

**Background:** Continuous renal replacement therapy (CRRT) is increasingly used for the management of dogs with acute kidney injury.

**Objectives:** To describe the management of dogs with AKI by CRRT, and to investigate the relationship between a prescribed CRRT target dose and the hourly urea reduction ratio.

**Animals:** Forty‐five client‐owned dogs diagnosed with severe AKI, receiving 48 CRRT treatments at a veterinary teaching hospital.

**Methods:** Retrospective study. Search of medical records of dogs with AKI managed by CRRT.

**Results:** Median serum urea and creatinine at CRRT initiation were 252 mg/dL (range, 64–603 mg/dL) and 9.0 mg/dL (range, 4.3–42.2 mg/dL), respectively. Median treatment duration was 21 hours (range, 3–32). Systemic heparinization and regional citrate anticoagulation were used in twenty‐four treatments each (50%). The median CRRT target dose for the entire treatment was 1 ml/kg/min (range, 0.3–2.5 ml/kg/min). Urea reduction ratio (URR) for the entire treatment was 76% (range, 11–92%) and the median hourly URR was 4%. While CRRT dose was increased gradually from 0.9 ml/kg/min to 1.4 ml/kg/min (*P*<0.001) the hourly URR decreased from 6.5% to 5.5% (*P*=0.05). The main complication was clotting of the extra‐corporeal circuit, occurring in 6/48 treatments (13%).

**Conclusions and Clinical Importance:** CRRT is a safe treatment modality for dogs with AKI, when the prescribed dose is based on the current veterinary guidelines for intermittent hemodialysis and human practice guidelines for CRRT. Treatment adequacy was maintained by gradually increasing the dose according to the actual URR.

## 187 Abstract NU12

### 187.1 Urinary miRNA 126 is Elevated in Dogs with Immune Complex‐Mediated Glomerulonephritis

#### 187.1.1 **Ariana D. Cherry** ^1^; Candice Chu^2^, PhD, DVM, DACVP; Rachel Cianciolo^3^, PhD, DVM, DACVP; Jessica Hokamp^3^, PhD, DVM, DACVP; Sarah Jacobson^4^; Mary Nabity^5^, PhD, DVM, DACVP

##### 187.1.1.1 ^1^Texas A&M University; ^2^Assistant Professor of Clinical Pathology, Veterinary Pathobiology, School of Veterinary Medicine, University of Pennsylvania; ^3^Clinical Associate Professor, The Ohio State University; ^4^Veterinary Student, College of Veterinary Medicine and Biomedical Sciences, Texas A&M University; ^5^Associate Professor, Veterinary Pathobiology, Texas A&M University

**Background:** Most proteinuric dogs with naturally occurring chronic kidney disease have one of three categories of glomerular disease, each with different treatment and prognostic considerations: immune complex‐mediated glomerulonephritis (ICGN), glomerulosclerosis (GS), or amyloidosis (AMYL). Urinary microRNAs (miRs) have the potential to be non‐invasive biomarkers of diagnostic value.

**Hypothesis/Objective:** We hypothesized that urinary miR‐126 would differentiate dogs with ICGN from GS and AMYL, and the expression of miRs‐21, 182, and 486 would differentiate azotemic from non‐azotemic dogs.

**Animals:** Archived urine from 39 dogs with azotemic and non‐azotemic glomerular disease (11 with AMYL, 11 with GS, 17 with ICGN) and 5 clinically healthy dogs were selected. ICGN dogs included 10 with membranous glomerulonephritis (MGN) and 7 with membranoproliferative glomerulonephritis (MPGN).

**Methods:** Retrospective. Urinary miRs‐126, 21, 182, and 486 were quantified using RT‐PCR with global mean normalization. One‐way ANOVA and Mann Whitney tests evaluated differences between disease groups (P<0.05).

**Results:** Regardless of azotemia status, urinary miR‐126 was significantly higher in dogs with ICGN and GS than AMYL and healthy controls (P<0.001) and in ICGN than GS (P<0.001). The expression of miR‐126 was 5.8 times higher in dogs with ICGN compared to GS, with no difference between MGN and MPGN (P=1.00). Expression of urinary miR‐21 (P=0.71), miR‐182 (P=0.79), and miR‐486 (P=0.95) were not different between azotemic and non‐azotemic dogs.

**Conclusions and Clinical Importance:** MiR‐126 could help identify dogs that could benefit from immunosuppressive therapy in the absence of a biopsy.

## 188 Abstract NU13

### 188.1 Renal Single‐Cell RNA Sequencing in Dogs with a Naturally Occurring Progressive Chronic Kidney Disease

#### 188.1.1 **Candice P. Chu** ^1^; Daniel Osorio^2^, PhD; Mary Nabity^3^, DVM, PhD, DACVP

##### 188.1.1.1 ^1^University of Pennsylvania; ^2^Postdoctoral Fellow, Livestrong Cancer Institutes; ^3^Associate Professor, Veterinary Pathobiology, Texas A&M University

**Background:** Single‐cell RNA sequencing (scRNA‐seq) is a novel technology to profile gene expression on the cellular level. To date, the application of scRNA‐seq in canine tissues is limited, including in chronic kidney disease.

**Hypothesis/Objectives:** We aimed to identify cell clusters in diseased canine kidney tissues and hypothesized that altered gene expression can be detected in advanced CKD.

**Animals:** One affected and one carrier dog from a research colony with X‐linked hereditary nephropathy, a naturally occurring animal model for canine CKD.

**Methods:** Prospective study. One gram of fresh kidney cortex was collected after euthanasia for renal failure. Single‐cell suspension was submitted for library preparation using the Chromium Single‐Cell 3’ Kit and sequenced on an Illumina NovaSeq. Data were analyzed and annotated using CellRanger, CellMarker, and PanglaoDB. Differential expression analysis was performed in Seurat R‐packages with the Wilcoxon Rank Sum test (adjusted P‐value <0.05).

**Results:** In total, we recovered 15,212 cells and identified 14 cell clusters in the kidney tissues, including major kidney cells (proximal and distal tubule cells, podocytes, mesangial cells, and collecting duct cells) and inflammatory cells. Comparing renal cells in the affected dog (rapid disease progression) to the carrier (slow disease progression), 133 differentially expressed genes were identified, such as MT2A (P<4.65E‐35) and APOC3 (P<2.74E‐68) that were involved in oxidative damage, fibrosis, and impeded vascular regeneration in human CKD.

**Conclusions and Clinical Importance:** We showed that scRNA‐seq is suitable for discovering promising therapeutic targets in canine tissues and providing new insights into CKD pathogenesis.

## 189 Abstract NU14

### 189.1 Differentiation of Stable Kidney Function versus Progressive Dysfunction in Dogs

#### 189.1.1 **Larry D. Cowgill** ^1^; Gilad Segev^2^, DVM, DECVIM‐CA; Shelly Vaden^3^, DVM, PhD, DACVIM (SAIM); Sheri Ross^4^, DVM, PhD, DACVIM (SAIM); Cedric Dufayet^4^, DVM; Leah Cohn^5^, DVM, PhD, DACVIM (SAIM); Mary Nabity^6^, DVM, PhD, DACVP; Giosi Farace^7^, PhD; Donald Szlosek^7^, MPH; Zenhwa Ouyang^7^, VMD, PhD, MS, MSE; Sarah Peterson^7^, MD, PhD; Melissa Beall^7^, DVM, PhD; Murthy Yerramilli^7^, PhD; David Polzin^8^, DVM, PhD, DACVIM (SAIM)

##### 189.1.1.1 ^1^School of Veterinary Medicine, University of California‐Davis, Davis, CA, USA; ^2^Hebrew University; ^3^North Carolina State University; ^4^Veterinary Medical Center, University of California, San Diego, CA, USA; ^5^University of Missouri; ^6^Texas A&M University; ^7^IDEXX Laboratories, Inc.; ^8^University of Minnesota

**Background:** Markers of kidney function have been used variably to define stable versus progressive chronic kidney disease (CKD), but quantitative criteria to distinguish these populations are lacking.

**Objective:** Assessment of 1/creatinine and 1/IDEXX SDMA® slope cutoffs to distinguish stable versus progressive CKD.

**Animals:** 113 clinically healthy University staff‐owned dogs and 29 male colony dogs with progressive X‐linked hereditary nephropathy (XLHN).

**Methods:** Retrospective analysis combining two prospective observational studies, one tracking kidney function markers in healthy dogs (HD) to a maximum of 3 years, and one tracking kidney function markers in male colony dogs with progressive XLHN to a maximum of 1 year. The minimum slope of 1/creatinine or 1/IDEXX SDMA® from HD was assigned as the slope cutoff for stable kidney function.

**Results:** The stable versus progressive slope cutoff was ‐0.0119 week*dL/mg for 1/creatinine (Figure 1, dashed line) and ‐0.0007 week*dL/μg for 1/IDEXX SDMA® (Figure 2, dashed line).

**Figure:**
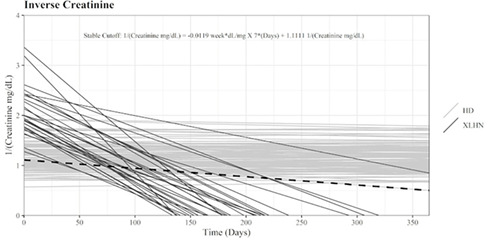
Figure 1

**Figure:**
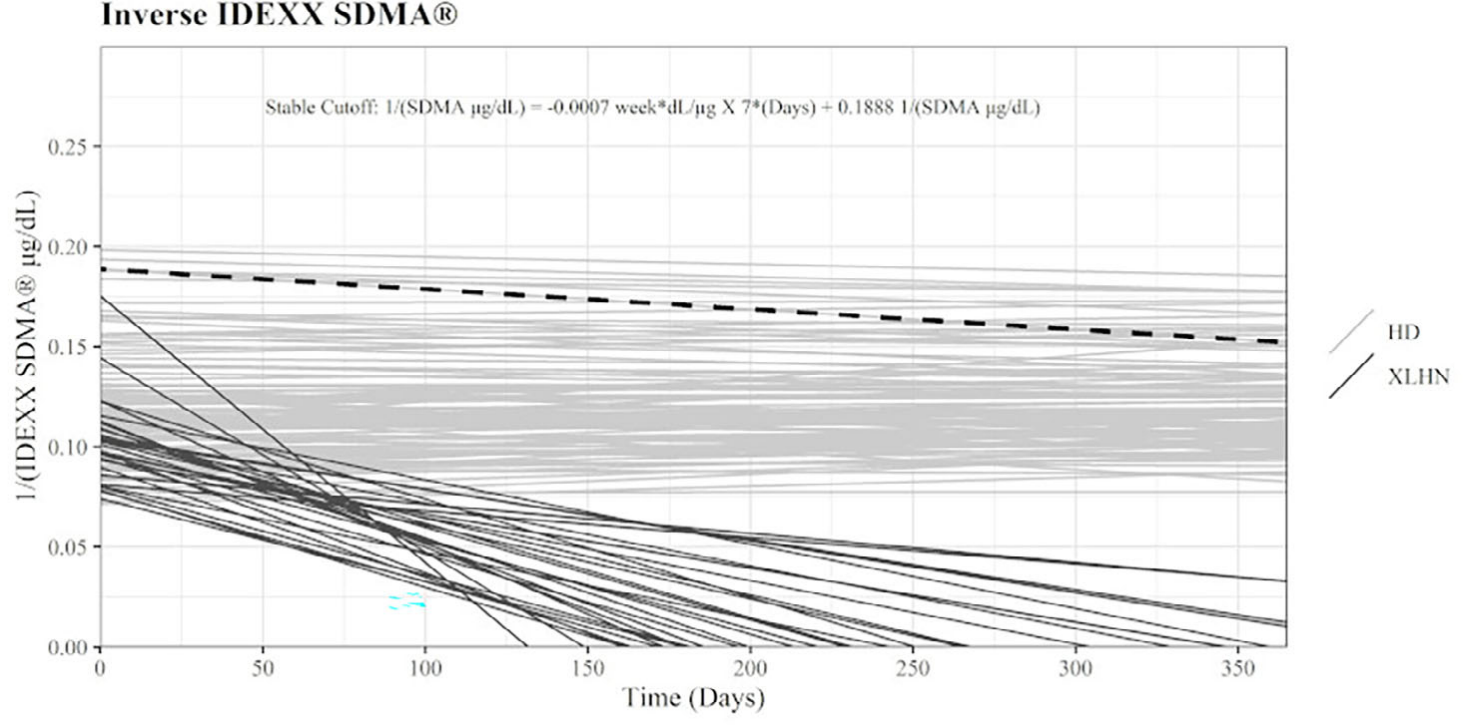
Figure 2

**Conclusions and Clinical Importance:** In the studied CKD population, progressive dysfunction can be distinguished from stable kidney function by using the slope of 1/creatinine or 1/IDEXX SDMA®. These criteria may serve to characterize CKD in other cohorts of dogs and to establish guidelines for progression rate in dogs with naturally acquired CKD.

## 190 Abstract NU15

### 190.1 Evaluation of Alpha Enolase as a Marker of Renal Disease in Cats

#### 190.1.1 **Amanda Diaz** ^1^; Jennifer Hawley^1^, BS; Stacie Summers^2^, DVM, PhD, DACVIM; Shannon McLeland^3^, DVM, PhD, DACVP; Michael Lappin^1^, DVM, PhD, DACVIM

##### 190.1.1.1 ^1^Colorado State University; ^2^Oregon State University; ^3^The Ohio State University

**Background:** Kidney disease is a common cause of mortality in cats and additional markers for early diagnosis are needed. Alpha‐enolase is a glycolytic pathway enzyme and is a potential biomarker for kidney disease as it changes distribution in the kidneys with age.

**Hypothesis/Objectives:** As kidneys are damaged, the degeneration of renal tubules will lead to increased concentrations of alpha‐enolase in the urine and serum.

**Animals:** Remnant urine and serum samples from cats in previous studies stored at ‐80°C until assayed.

**Methods:** Alpha‐enolase is highly conserved and so a human kit (Abnova Corp. Taiwan) was used. Alpha‐enolase was spiked into urine of normal cats to determine if inhibitors were present and to optimize the technique. Alpha‐enolase was measured in the serum and urine from 6 cats in a vaccine hyper‐inoculation model that had shown redistribution of α‐enolase within the kidneys. Lastly, α‐enolase was measured in serum and urine from 21 client‐owned cats with and without kidney disease.

**Results:** Detection of α‐enolase was optimal if urine was normalized to a USG of 1.010. While vaccine hyper‐inoculation changed α‐enolase distribution in kidneys, it was only detected in 2 sera and no urine samples. In client‐owned cats with and without CKD, α‐enolase was only detected in 2 sera and no urine samples.

**Conclusions and Clinical Importance:** Detection of α‐enolase was not beneficial as a marker of kidney changes in the 2 sample sets assessed to date. Assessment of samples from cats with acute kidney injury is ongoing.

## 191 Abstract NU16

### 191.1 Population Pharmacokinetic Analysis of Enrofloxacin and Its Metabolite Ciprofloxacin in Cats with Reduced Kidney Function

#### 191.1.1 **JD Foster** ^1^; Mahmoud Abouraya^2^, DVM, PhD, DACVCP; Mark Papich^3^, DVM, MS, DACVCP; Nancy Muma^4^, PhD

##### 191.1.1.1 ^1^Friendship Hospital for Animals; ^2^FDA; ^3^Professor of Clinical Pharmacology, College of Veterinary Medicine, North Carolina State University; ^4^Professor and Chair, Department of Pharmacology and Toxicology, School of Pharmacy, University of Kansas

**Background:** Enrofloxacin is commonly used for treating pyelonephritis as it achieves high tissue concentrations and is eliminated predominantly in urine. In other species, when glomerular filtration is reduced some fluoroquinolones have decreased clearance and drug accumulation within plasma. It is unknown if enrofloxacin accumulation occurs in cats with reduced kidney function.

**Hypothesis/Objectives:** To determine if enrofloxacin and its active metabolite ciprofloxacin have reduced clearance in azotemic cats.

**Animals:** Hospitalized client owned cats who were prescribed enrofloxacin were eligible for enrollment.

**Methods:** Enrofloxacin (dose 5 mg/kg) was administered to 34 cats hospitalized for clinical illness with variable degree of kidney dysfunction. In a prospective study, sparse blood sampling was used to obtain population pharmacokinetic results with nonlinear mixed‐effects modeling. Plasma enrofloxacin and ciprofloxacin concentrations were measured and summed to obtain the total fluoroquinolone concentration. A model of ciprofloxacin metabolism from enrofloxacin was created and evaluated for covariate effects on clearance, volume of distribution, and the metabolic rate of ciprofloxacin generation from enrofloxacin.

**Results:** Body weight was the only covariate found to affect total fluoroquinolone volume of distribution and clearance. Kidney function did not have a significant effect on total fluoroquinolone, enrofloxacin, or ciprofloxacin clearance. Blood urea nitrogen concentration had an effect on the metabolic generation of ciprofloxacin from enrofloxacin, but other markers of kidney function did not.

**Conclusions and Clinical Importance:** Decreased kidney function did not affect plasma total fluoroquinolone, enrofloxacin, or ciprofloxacin clearance. Adjustment of enrofloxacin dosage is not indicated for azotemic cats, however further study of multiple doses is needed.

## 192 Abstract NU17

### 192.1 Urinary Glutathione Peroxidase Four in Cats with Naturally Occurring Chronic Kidney Disease

#### 192.1.1 **Sheng‐hui Huang** ^1^; Ya‐jane Lee^2^, PhD

##### 192.1.1.1 ^1^Institute of Veterinary Clinical Science, School of Veterinary Medicine, College of Bio‐Resources and Agriculture, National Taiwan University, Taipei, Taiwan; ^2^Associate Professor, School of Veterinary Medicine, National Taiwan University, Taipei, Taiwan

**Background:** Glutathione peroxidase 4 (GPX4) is a biomarker of ferroptosis, which is characterized by iron‐catalyzed accumulation of lethal lipid reactive oxygen species (ROS), leading to glutathione inactivation and cell death. GPX4 inactivation is involved in post‐ischemic kidney injury and kidney fibrosis. However, the role of GPX4 in cats with chronic kidney disease (CKD) remain largely unknown.

**Hypothesis:** Urinary GPX4 may be associate with clinical parameters and subgroups in cats with CKD.

**Animals:** Twenty‐seven CKD and 7 healthy cats.

**Methods:** Case control study. Cats were grouped according to International Renal Interest Society staging system. Urinary GPX4 was measured by commercial ELISA kit for cats.

**Results:** The urinary GPX4 of CKD cats (median [interquartile range]: 24.6 [22.1–26.0] ng/mL) was significantly lower than those of healthy cats (26.9 [26.1–28.2] ng/mL, P=0.003). After adjusting with urine creatinine, urinary GPX4‐to‐creatinine ratio (UGCR) was significantly higher in CKD cats (0.257 [0.158–0.358]×10–4) compared to controls (0.097 [0.071–0.108]×10–4, P=0.007). UGCR was increased in cats with proteinuria (0.35 [0.27–0.55]) compared to those without (0.20 [0.11–0.33]×10–4, P=0.004). By stepwise selection of variables which were significantly correlated with UGCR, including creatinine, blood urea nitrogen, phosphate, urine specific gravity (USG), hematocrit and hemoglobin (all P<0.005) in simple linear regression, higher phosphate (P=0.002) and lower USG (P=0.027) are independent factors associated with higher UGCR.

**Conclusions and Clinical Importance:** Increased urinary GPX4‐to‐creatinine ratio was significantly associated with decline of renal function in cats.

**Table:** Table 1. The differences in various variables between the control and CKD groups

				CKD groups (N=27)		
Parameters	Control (N=7)	Early stage^a^		Late stage^b^		P value
GPX4 (ng/mL)	26.9 (26.08–28.19)	22.6 (21.25–24.98)^c^	N=14	25.6 (23.97–26.32)	N=12	0.003
UGCR (×10^‐4^)	0.097 (0.072–0.108)	0.21 (0.07–0.32)	N=14	0.26 (0.19–0.42)c	N=12	0.007
Creatinine (mg/dL)	1.6 (1.5–1.6)	2.3 (1.75–2.5)	N=14	3.8 (3.25–4.5)^c,d^	N=13	<0.001
BUN (mg/dL)	26 (21–28)	29.5 (22.5–34.5)	N=14	43.5 (38.25–63.0)^c,d^	N=13	0.001
Albumin (g/dL)	3.8 (3.6–4.2)	3.2 (3.1–3.38)c	N=8	3.4 (2.8–3.65)c	N=9	0.006
Hematocrit (%)	39.9 (33.5–48.4)	40.2 (38.23–44.23)	N=14	36.3 (30.0–42.0)	N=13	0.154
Phosphate (mg/dL)	‐	4.0 (3.6–5.0)	N=11	4.6 (4.2–7.7)	N=13	0.068
Sodium (mEq/L)	157.7 (155.7–157.9)	155.1 (153.5–156.4)	N=14	155.2 (152.8–159.3)	N=13	0.258
Potassium (mEq/L)	3.81 (3.67–3.88)	3.72 (3.46–4.05)	N=14	3.87 (3.66–4.30)	N=13	0.399
Chloride (mEq/L)	115.7 (114.6–116.5)	117.3 (115.4–118.7)	N=14	118.0 (116.0–119.8)	N=13	0.463
USG	1.046 (1.043–1.055)	1.013 (1.010–1.037)c	N=14	1.012 (1.009–1.014)c	N=13	0.001
UPC	0.03 (0.02–0.06)	0.10 (0.03–0.19)	N=11	0.63 (0.13–0.84)c	N=11	0.004

Variables were tested with Kruskal Wallis Test and represented as the median (interquartile range, IQR). The *post hoc* analysis was Dunn's test. Abbreviation: GPX4, Glutathione peroxidase 4; UGCR, urinary GPX4‐to‐creatinine ratio; BUN, Blood urea nitrogen; USG, urine specific gravity; UPC, urine protein‐to‐creatinine ratio a: IRIS stage I and stage II b: IRIS stage III and stage IV c: Achieved significance with control group. d: Achieved significance with early stage group.

**Table:** Table 2. Simple and stepwise multiple linear regression analysis for UGCR as a dependent variable

Simple regression analysis				Stepwise multiple regression analysis		
Parameters	Adjusted β	t	*P* value*	Adjusted β	t	*P* value*
Creatinine (mg/dL)	0.504	3.031	0.005			
BUN (mg/dL)	0.667	4.56	<0.001			
Hematocrit (%)	‐0.566	‐3.567	0.001			
Hemoglobin (g/dL)	‐0.566	‐3.564	0.001			
ALT(IU/L)	0.703	2.798	0.023			
AST (IU/L)	0.634	2.459	0.036			
Phosphate (mg/dL)	0.687	4.633	<0.001	0.539	3.556	0.002
WBC (/μL)	0.157	0.824	0.417			
Platelet (K/μL)	0.318	1.714	0.093			
Albumin (g/dL)	0.203	0.827	0.42			
Sodium (mEq/L)	‐0.122	‐0.637	0.53			
Potassium (mEq/L)	‐0.18	‐0.951	0.35			
Chloride (mEq/L)	‐0.24	‐1.285	0.21			
USG	‐0.582	‐3.715	0.001	‐0.36	‐2.375	0.027
UPC	0.175	0.814	0.425			

Abbreviations: UGCR, urinary GPX4‐to‐creatinine ratio; BUN, Blood urea nitrogen; ALT, Alanine Aminotransferase; AST, Aspartate transaminase; WBC, White blood cells; USG, urine specific gravity; UPC, urine protein‐to‐creatinine ratio. *P value<0.05 as significant.

## 193 Abstract NU18

### 193.1 Non‐Transferrin‐Bound Iron in Cats with Naturally Occurring Chronic Kidney Disease

#### 193.1.1 **Sheng‐hui Huang** ^1^; Ya‐Li Chang^2^, MS; Ya‐jane Lee^3^, PhD

##### 193.1.1.1 ^1^Institute of Veterinary Clinical Science, School of Veterinary Medicine, College of Bio‐Resources and Agriculture, National Taiwan University, Taipei, Taiwan; ^2^Institute of Veterinary Clinical Science, School of Veterinary Medicine, College of Bio‐Resources and Agriculture, National Taiwan University, Taipei, Taiwan; ^3^Associate Professor, School of Veterinary Medicine, National Taiwan University, Taipei, Taiwan

**Background:** Non‐transferrin‐bound iron (NTBI), generating reactive oxygen species (ROS) via Fenton reaction, induce oxidative damage in renal molecules. NTBI has been identified in end‐stage renal disease patients and animal models. However, the role of NTBI in cats with chronic kidney disease (CKD) has not been previously published.

**Hypothesis:** By accumulation of iron‐mediated ROS, NTBI may be associate with clinical parameters and subgroups in cats with CKD.

**Animals:** Fifty‐two CKD and 17 healthy cats.

**Method:** Cats with CKD were grouped by the standard of International Renal Interest Society (IRIS) staging system. Serum NTBI is measured by Inductively Coupled Plasma Mass Spectrometric.

**Results:** After adjusting with hematocrit, serum NTBI‐to‐hematocrit ratio (SNHR) were determined. SNHR was significantly elevated in late‐CKD stage group (median [interquartile range]: 0.20 [0.170–.24]×10–2 ppb) compared to controls (0.16 [0.15–0.19]×10–2 ppb, P=0.035). In CKD group, SNHR was increased in cats with proteinuria (0.20 [0.18–0.25] 10–2 ppb) compared to those without (0.17 [0.14–0.19] 10–2 ppb, P=0.007). SNHR of hypertensive cats (0.25 [0.18–0.3] 10–2 ppb) was significantly higher than those of non‐hypertensive cats (0.17 [0.15–0.19] 10–2 ppb, P=0.034). Higher SNHR was significantly correlated with higher creatinine (P=0.023), BUN (P=0.003) and UPC (P=0.04) in simple linear regression.

**Conclusions and Clinical Importance:** Elevation of serum NTBI‐to‐hematocrit ratio was significantly associated with late‐stage CKD in cats. In CKD cats with hypertensive and proteinuria, SNHR was found to be significantly higher than those without.

**Table:** Table 1. The differences in various variables between the control and CKD groups

				CKD groups (N=27)		
Parameters	Control (N=17)	Early stage^a^		Late stage^b^		*P* value
SNHR (×10^‐2^ ppb)	0.16 (0.15–0.19)	0.17 (0.14–0.18)	N=34	0.20 (0.17–0.24)^c^	N=16	0.035
Age (year)	4.94 (1–7.5)	9.24 (5.75–13)c	N=34	14 (8.75–16)^cd^	N=18	<0.001
Creatinine (mg/dL)	1.60 (1.2–1.7)	2.00 (1.9–2.32)	N=34	3.55 (3.07–4.52)cd	N=18	<0.001
BUN (mg/dL)	22 (19–25.5)	23 (21.75–31)	N=34	40.5 (33–64)^cd^	N=18	<0.001
Hematocrit (%)	42.56 (37.56–48.85)	40.10 (37.15–42.63)	N=34	33.86 (26.95–38.4)^cd^	N=17	<0.001
RBC (106/μL)	8.63 (7.53–9.65)	8.29 (7.65–9.31)	N=34	7.30 (6.13–8.05)^c^	N=17	0.034
Hemoglobin (g/L)	14.44 (13–16.12)	13.69 (12.6–14.45)	N=34	11.46 (9.5–12.75)^cd^	N=17	<0.001
Platelet (K/μL)	223 (133–280)	283 (219–380)	N=33	287 (213–423)	N=17	0.125
Albumin (g/dL)	3.60 (3.35–3.75)	3.48 (3.15–3.7)	N=25	3.27 (2.98–3.43)	N=14	0.064
Phosphate (mg/dL)	‐	4.55 (3.95–5.1)	N=24	4.90 (4.2–6)	N=18	0.121
Total calcium (mg/dL)	‐	10.6 (10.33–11.3)	N=10	11.5 (10.5–15)	N=7	0.105
USG	1.05 (1.04–1.05)	1.02 (1.01–1.03) c	N=34	1.01 (1–1.02)^c^	N=18	<0.001
Urinary pH	6.45 (5.94–6.87)	6.40 (5.93–6.65)	N=34	5.98 (5.93–6.4)	N=18	0.030
UPC	0.02 (0.02–0.02)	0.075 (0.02–0.16)^c^	N=34	0.19 (0.11–1.04)^cd^	N=18	<0.001
Sodium (mEq/L)	157. (155–158.7)	155.65 (154–157.8)	N=24	155.25 (153.6–158.9)	N=18	0.699
Potassium (mEq/L)	3.96 (3.7–4.03)	3.84 (3.6–4.03)	N=24	4.10 (3.71‐4.3)	N=18	0.080
Chloride (mEq/L)	118.3 (117.6–120.5)	118.1 (116.9–120.3)	N=24	118.4 (116.5–122.9)	N=18	0.857

Variables were tested with Kruskal Wallis Test and represented as the median (inter quartile range, IQR). The *post hoc* analysis was Dunn's test. Abbreviation: SNHR, serum NTBI‐to‐hematocrit ratio; BUN, Blood urea nitrogen; USG, urine specific gravity; UPC, urine protein‐to‐creatinine ratio a: IRIS stage I and stage II b: IRIS stage III and stage IV c: Achieved significance with control group. d: Achieved significance with early stage group.

**Figure:**
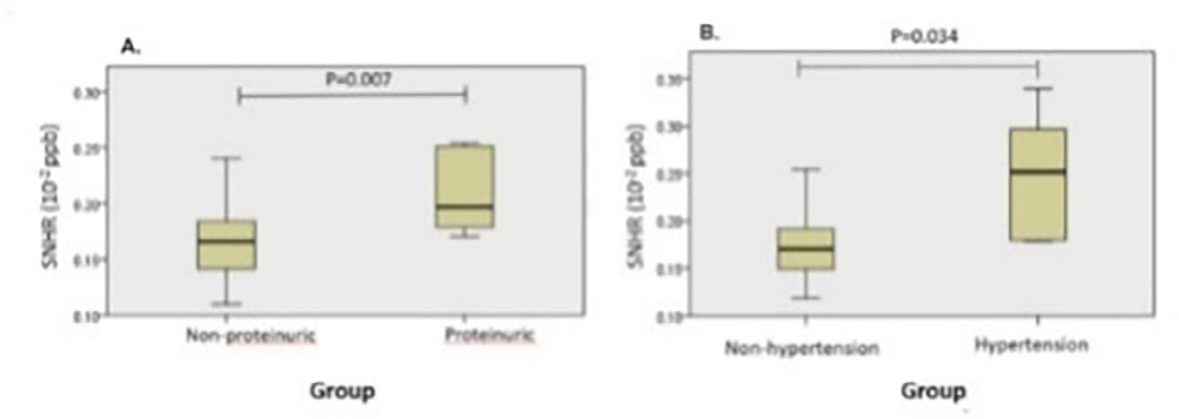
**Figure 1.** SNHR in cats with proteinuria and hypertension compared to those without in CKD group. A: SNHR in proteinuric CKD cats (0.20 [0.18–0.25] 10–2 ppb) and non‐proteinuric CKD cats (0.17 [0.14–0.19] 10–2 ppb). SNHR significantly increased in proteinuric cats compared to non‐proteinuric cats (P=0.007). B: SNHR in CKD cats with hypertension (0.25 [0.18–0.3] 10–2 ppb) and CKD cats with normal blood pressure (0.17 [0.15–0.19] 10–2 ppb). SNHR significantly increased in cats with hypertension (P=0.034).

## 194 Abstract NU19

### 194.1 Diltiazem Infusion Alterations on Glomerular Filtration Rate, Electrolyte Excretion, and Urine Output in Healthy Dogs

#### 194.1.1 **Megan Kelley** ^1^; William Whitehouse^2^, DVM, DACVIM (SAIM); Justin Thomason^3^, DVM, DACVIM (SAIM & Cardiology); Angela Thompson‐Butler^4^; Matthew Tanner^5^, DVM

##### 194.1.1.1 ^1^Veterinary Health Center, Kansas State University; ^2^Assistant Professor, College of Veterinary Medicine, Kansas State University; ^3^Clinical Associate Professor, College of Veterinary Medicine, Kansas State University; ^4^College of Veterinary Medicine, Kansas State University; ^5^Clinical Assistant Professor, College of Veterinary Medicine, Kansas State University

**Background:** Canine acute kidney injury (AKI) has a high mortality rate. Diltiazem may improve renal function but has not been adequately studied in dogs.

**Hypothesis/Objectives:** To determine if a diltiazem constant rate infusion (CRI) improves renal function through changes in GFR, fractional excretion of sodium (FE_Na_), and UOP in healthy dogs.

**Animals:** Ten healthy, client‐owned dogs.

**Methods:** A prospective, randomized, crossover study. Dogs were randomized to receive intravenous diltiazem (loading dose of 240 mg/kg followed by a CRI of 6 mg/kg/min for 300 minutes) or the same volume of 5% dextrose in water (D5W). The opposite treatment was given after a 7 day washout period.

**Results:** GFR did not significantly increase from baseline with diltiazem (pre‐diltiazem median = 2.371 mL/min/kg, range = 1.605–4.359; post‐diltiazem median = 2.305 mL/min/kg, range = 1.629–4.387; P=0.846), and there was no statistical difference in GFR post‐diltiazem or post‐D5W (median = 2.389 mL/min/kg, range = 1.600–3.557; P=0.695). FE_Na_ did not significantly increase from baseline with diltiazem (pre‐diltiazem median = 0.1%, range = 0–0.5; post‐diltiazem median = 0.1%, range = 0–0.9; P=0.813), and there was no statistical difference in FE_Na_ post‐diltiazem or post‐D5W (median = 0.17%, range = 0.1–0.4; P=0.258). There was a trend towards an increase in UOP with diltiazem, but it was not statistically significant (P=0.065).

**Conclusion:** Diltiazem does not appear to improve markers of renal function in healthy dogs. Further studies are needed in dogs with AKI.

## 195 Abstract NU20

### 195.1 Urinary Angiotensin‐Converting Enzyme 2 Concentration and Activity in Cats with Naturally Occurring Chronic Kidney Disease

#### 195.1.1 **Tzu‐Chien Kuo** ^1^; Ya‐jane Lee^2^, PhD

##### 195.1.1.1 ^1^Institute of Veterinary Clinical Science, School of Veterinary Medicine, College of Bio‐Resources and Agriculture, National Taiwan University; ^2^Associate Professor, Institute of Veterinary Clinical Science, School of Veterinary Medicine, College of Bio‐Resources and Agriculture, National Taiwan University, Taipei, Taiwan

**Background:** Urinary angiotensin‐converting enzyme 2 (UACE2) can shed from the injured renal tubules but whether UACE2 activity would be increased or decreased in CKD was inconclusive in people and laboratory animal study. However, ACE2 in cat's urine was never investigated.

**Hypothesis/Objectives:** We hypothesized that UACE2 and UACE2 activity would be significantly different between the healthy and the CKD cats.

**Animals:** Seventy‐five cats included 24 healthy cats and 51 CKD cats. Cats were enrolled and grouped according to the International Renal Interest Society (IRIS) staging system.

**Methods:** Retrospective case‐control study. Using ELISA and fluorometric assay kits to measure UACE2 and UACE2 activity. UACE2 activity‐to‐UACE2 ratio was calculated as the urinary ACE2 functional unit.

**Results:** The UACE2‐to‐creatinine ratio of CKD cats (median [interquartile range]: 3.88 [2.13–5.10]×10–6) was significantly higher than that of healthy cats (0.89 [0.61–1.08]×10–6, P<0.001), but CKD cats had the significantly lower UACE2 activity‐to‐UACE2 ratio (0.38 [0.23–0.80] pmol/min/ng) than healthy cats (2.93 [1.21–4.74] pmol/min/ng, P<0.001). UACE2‐to‐creatinine ratio significantly positively correlated with age, plasma creatinine, BUN, phosphate, urine protein‐to‐creatinine ratio, and negatively with hematocrit and urine specific gravity (USG) (all P<0.005). After using stepwise multiple regression analysis, BUN (P=0.008) and USG (P=0.040) were the independent factors.

**Conclusion and Clinical Importance:** Urinary ACE2‐to‐creatinine ratio was significantly associated with decreased renal function in CKD cats but shed ACE2 in CKD cats was less functional than that in healthy cats.

**Table:** Table 1. The differences in various variables between the control and CKD groups. Variables were tested with Kruskal Wallis Test (*post hoc* with Dunn test) and represented as the median (inter quartile range, IQR)

Variable	Control group (n=24)		CKD group (n=51)				P*
			**Early stage** ^**a**^ **(n=26)**		**Late stage** ^**b**^ **(n=25)**		
	**Median (IQR)**	n	**Median (IQR)**	n	**Median (IQR)**	n	
Age (year)	6.5 (2.5–8.0)	24	8.0 (5.0–12.5)	25	13.0 (7.5–15.0)^c,d^	25	<0.001
HCT (%)	41.2 (35.8–45.5)	23	40.3 (38.2–44.8)	25	37.0 (31.6–39.0) d	25	0.025

Abbreviation: AG ratio, albumin‐to‐globulin ratio; BUN, Blood urea nitrogen; HCT, hematocrit; SAP, systolic arterial pressure; UACE2, urinary angiotensin‐converting enzyme 2; UPC, urine protein‐to‐creatinine ratio. a: IRIS stage 1 and stage 2 b: IRIS stage 3 and stage 4 c: Achieved significance with control group. d: Achieved significance with early stage group. *P value<0.05 as significant

**Table:** Table 2. Simple and stepwise multiple linear regression analysis for UACE2‐to‐creatinine ratio as a dependent variable

Variables	Simple regression analysis			Stepwise regression analysis		
	Adjustment β	t	P*	Adjustment β	t	P*
Age (year)	0.445	4.221	<0.001			
UPC	0.607	6.118	<0.001			
SAP (mmHg)	‐0.056	‐0.324	0.748			
HCT (%)	‐0.479	‐4.624	<0.001			
Creatinine (mg/dl)	0.677	7.859	<0.001			
BUN (mg/dl)	0.718	8.804	<0.001	0.477	2.929	0.008
Phosphate (mg/dl)	0.447	3.357	0.002			
Urine specific gravity	‐0.645	‐7.219	<0.001	‐0.354	‐2.176	0.040

Abbreviation: BUN, blood urea nitrogen; HCT, hematocrit; SAP, systolic arterial pressure; UACE2, urinary angiotensin‐converting enzyme 2; UPC, urine protein‐to‐creatinine ratio. *P<0.05 as significant

## 196 Abstract NU21

### 196.1 Pilot Field Study of Hypoxia‐Inducible Factor Prolyl Hydroxylase Inhibitor in Chronic Kidney Disease‐Associated Anemic Cats

#### 196.1.1 **Catherine E. Langston** ^1^; Samuel Charles^2^; Ricarda Huesken^2^; Dorothee Stanneck^2^; Terry Settje^3^; Chantal Lainesse^4^

##### 196.1.1.1 ^1^The Ohio State University; ^2^Elanco Animal Health; ^3^Olathe, Kansas; ^4^Integral Consulting Strategies, Inc.

**Background:** Safety and erythropoietic effects of molidustat sodium, a novel hypoxia‐inducible factor prolyl hydroxylase inhibitor (HIF‐PHI), were previously demonstrated in healthy cats.

**Objective:** To evaluate in‐use safety and erythropoietic response of anemic cats with chronic kidney disease (CKD) to daily oral administrations of molidustat.

**Animals:** Twenty‐one client‐owned CKD cats (4–17 years old; both sexes) with non‐regenerative anemia.

**Methods:** This multicenter pilot field study was randomized, blinded, and placebo controlled. Cats were treated once daily for 28 days with oral suspensions of either control product (CP; n=6) or 5 mg/kg of molidustat (n=15). Hematocrit (HCT) was evaluated at weekly intervals. Individual cat treatment success was defined as a ≥4‐percentage point increase in HCT compared to baseline.

**Results:** Control group mean HCT remained low throughout the study (20.1–23.4%). Mean HCT of molidustat‐treated group increased weekly and a statistically significant increase compared to baseline (23.6%) was first observed on day 21 (27.3%, p=0.0005, 95%CI [1.69–5.67]). Compared to the CP group, mean HCT was significantly greater on day 21 (27.3% versus 20.1%; p=0.001, 95%CI [2.91–10.75]) approaching statistical significance on day 28 (27.8% versus 23.4%; p=0.061, 95%CI [‐0.23–9.88]). The number of individual treatment successes on day 28 was higher among molidustat‐treated cats (7/14) compared to CP‐treated cats (1/5). However, there was no significant difference between groups.

**Conclusions and Clinical Importance:** Daily oral molidustat administrations stimulated a clinically relevant erythropoietic response in anemic cats with CKD. This HIF‐PHI may offer a safe alternative for managing anemia in cats compared to current recombinant EPO treatment.

## 197 Abstract NU22

### 197.1 Apolipoprotein B100 Is a Potential Urine Biomarker for Membranoproliferative Glomerulonephritis in Dogs with Protein‐Losing Nephropathy

#### 197.1.1 **Crystal Ma** ^1^; Jessica Hokamp^2^, DVM, PhD, DACVP (Clinical Pathology); Rachel Cianciolo^3^, DVM, PhD, DACVP (Anatomic Pathology)

##### 197.1.1.1 ^1^College of Veterinary Medicine, The Ohio State University; ^2^Assistant Professor, Veterinary Biosciences, College of Veterinary Medicine, The Ohio State University; ^3^Zoetis

**Background:** Membranoproliferative glomerulonephritis (MPGN) causes glomerular damage in dogs. Renal biopsy is required to definitively diagnose MPGN and mixed MPGN (both have subendothelial immune deposits) and guide immunosuppressive therapy; minimally invasive and economical methods of MPGN diagnosis are needed. Liquid chromatography‐tandem mass spectrometry (LC‐MS/MS) found that urine Apolipoprotein B100 (ApoB100) is a candidate urine biomarker of canine MPGN.

**Objectives:** Optimize an antibody for Western blot detection of canine urine ApoB100 and confirm LC‐MS/MS data. Explore urine ApoB100 as a biomarker of canine MPGN.

**Methods:** Retrospective, case‐control study. Human ApoB100 antibody was optimized for Western blot detection of canine urine ApoB100. A 550 kDa band, compatible with ApoB100, was confirmed in urine samples in which ApoB100 had previously been detected by LC‐MS/MS. Urine supernatant from 86 dogs with MPGN/mixed MPGN (n=40) and non‐MPGN (n=46) (membranous glomerulonephritis (n=12), amyloidosis (n=15), focal segmental glomerulosclerosis (n=12), mesangioproliferative glomerulonephritis (n=6), and podocytopathy (n=1)), were probed with Human ApoB100 antibody. Results were designated positive for ApoB100 if a band was present at 550 kDa and negative if the band was absent. Chi‐squared analysis was used to assess for a significant difference in ApoB100 positivity between MPGN and non‐MPGN urine samples.

**Results:** Urine samples were positive for ApoB100 in a significantly greater proportion of dogs with MPGN (68%, n=27) than in dogs with non‐MPGN glomerular diseases (26%, n=12) (p<0.05).

**Conclusions:** Human ApoB100 antibody cross reacts with canine urine ApoB100. Western blot results confirm LC‐MS/MS data and suggests urine ApoB100 is a biomarker of canine MPGN.

## 198 Abstract NU23

### 198.1 Electrophoretic Urine Protein‐Banding Patterns as a Diagnostic Biomarker for Canine Membranoproliferative Glomerulonephritis

#### 198.1.1 **Madison R. McKay** ^1^; Nahvid Etedali^2^, DVM, DACVIM; Rachel Cianciolo^3^, VMD, PhD, DACVP (Anatomic); Jessica Hokamp^4^, DVM, PhD, DACVP (Clinical Pathology)

##### 198.1.1.1 ^1^The Animal Medical Center; ^2^Senior Veterinarian, Internal Medicine, The Animal Medical Center; ^3^Associate Professor, Veterinary Biosciences Department, The Ohio State University; ^4^Assistant Professor, Department of Veterinary Biosciences, The Ohio State University

**Background:** Electrophoretic urine protein banding patterns correlate with glomerular damage severity; their ability to differentiate glomerular disease forms is unknown. Anecdotally, the relative surface area of electrophoretic urine proteins bands >200 kDa (rSA200) appears predictive of canine membranoproliferative glomerulonephritis (MPGN), a form immune complex mediated glomerulonephritis (ICGN), and might improve detection of ICGN and MPGN compared to urine protein:creatinine (UPC).

**Objective:** Assess rSA200 and UPC of common glomerular disease subtypes. Determine significant differences in rSA200 and UPC between ICGN and non‐IGCN dogs and between MPGN and non‐MPGN dogs.

**Animals:** Urine supernatant from 114 dogs submitted to the International Veterinary Renal Pathology Service concurrently with renal biopsy.

**Methods:** Retrospective study. rSA200, determined from review of electrophoretic urine protein banding patterns, and UPC, reported at time of sample submission, were compared across glomerular disease subtypes (amyloid, focal segmental glomerulosclerosis, mesangioproliferative glomerulonephritis, membranous glomerulonephritis (MGN)/mixed MGN, and MPGN/mixed MPGN) and according to generalized categories: all ICGN vs. non‐ICGN dogs and MPGN vs. non‐MPGN dogs. Wilcoxon rank sum analysis was used to determine significant differences in rSA200 and UPC between ICGN vs. non‐ICGN dogs and between MPGN vs. non‐MPGN dogs.

**Results:** Table 1 summarizes rSA200 and UPC data. rSA200 is significantly greater (p<0.01) in MPGN than non‐MPGN dogs; rSA200 is not significantly different between ICGN vs. non‐ICGN dogs. UPC is not significantly different between MPGN vs. non‐MPGN dogs nor between ICGN vs. non‐ICGN dogs.

**Table:** Table 1

	Amyloid	FSGS	Mesangioproliferative GN	MGN/Mixed MGN	MPGN/Mixed MPGN	ICGN	Non‐ICGN	MPGN	Non‐MPGN
N	13	29	11	15	30	56	42	30	68
rSA200, mean (grayscale units)	0.32	0.17	0.10	0.11	1.04	0.61	0.21	1.04	0.17
rSA200, mean (range) (grayscale units)	0.02 0–3.15)	0 (0–2.37)	0 (0–0.71)	0 (0–1.37)	0.28 (0–7.75)	0.02 (0–7.75)	0 (0–3.15)	0.28 (0–7.75)	0 (0–3.15)
UPC, mean	16.2	7.7	6.3	6.6	9.3	8.0	10.6	9.3	9.1
UPC, median (range)	10.4 (3.1–41.1)	7.1 (1–24)	4.8 (3.5–15.5)	6.6 (0.3–14.9)	8.3 (2–19.8)	6.8 (0.3–19.8)	8.1 (1–41.4)	8.3 (2–19.8)	6.8 (0.3–41.4)

**Conclusion:** rSA200 is a potential biomarker of canine MPGN. UPC does not distinguish ICGN from non‐ICGN.

## 199 SMALL ANIMAL INTERNAL MEDICINE – OTHER

## 200 Abstract OT01

### 200.1 Using End‐of‐Life Survey to Investigate the Relationship between Quality‐of‐Life, Manner and Location of Death

#### 200.1.1 **Kellyn McNulty** ^1^; Kate Creevy^2^, DVM, MS, DACVIM (SAIM); Annette Fitzpatrick^3^, PhD; Audrey Ruple^4^, DVM, MS, PhD, DACVPM, MRCVS

##### 200.1.1.1 ^1^College of Veterinary Medicine and Biomedical Sciences, Texas A&M University; ^2^Professor, Small Animal Clinical Sciences, College of Veterinary Medicine and Biomedical Sciences, Texas A&M University; ^3^Research Professor, Family Medicine, Epidemiology and Global Health, University of Washington; ^4^Associate Professor, Population Health Sciences, Virginia‐Maryland College of Veterinary Medicine

**Background:** The Dog Aging Project (DAP) is a longitudinal study of aging in >30,000 American dogs. When a DAP dog dies, End‐of‐Life Survey (EOLS) is employed to understand factors contributing to the dog's death. Previous analysis of EOLS data showed 83% of deceased DAP dogs were euthanized, and 73% of respondents elected euthanasia due to their dog's pain and suffering or poor quality‐of‐life (QOL).

**Objectives:** To examine the relationship between a dog's manner of death (euthanasia vs unassisted), location of death, and QOL two weeks prior to death.

**Animals:** Deceased DAP dogs whose owners voluntarily completed EOLS (n=646).

**Methods:** Survey results were analyzed qualitatively.

**Results:** The owner‐reported perimortem QOL was variable, but there was an inverse relationship between QOL and age at death and between QOL and likelihood of euthanasia. Older dogs (>11 years) were more likely to have a poorer QOL. Owners reporting a QOL of “always bad days” were 1.5 times more likely to elect euthanasia compared to owners who reported a QOL of “always good days.” Most dogs (65.9%) died at a veterinary facility while 32.4% died in their owner's home; 96.5% and 58.4% of these dogs were euthanized, respectively.

**Conclusions and Clinical Importance:** Dogs with poorer perimortem QOL were more likely to be older and to be euthanized. Euthanasia accounted for the majority of deaths regardless of location. These valuable insights highlight the utility and necessity of EOLS in gathering this end‐of‐life information directly from dog owners.

## 201 Abstract OT02

### 201.1 Quality and Timing Identified as Most Common Diagnostic‐Related Incidents in Small Animal Veterinary Care

#### 201.1.1 **Lisen Schortz** ^1^; Liz Mossop^2^; Annika Bergström^3^; Catherine Oxtoby^4^

##### 201.1.1.1 ^1^Anicura, University of Lincoln; ^2^Professor, University of Lincoln; ^3^Swedish University of Agricultural Sciences; ^4^Veterinary Defence Society

**Background:** Veterinary care can be complicated, and like with any type of healthcare, there are risks of unintentional harm. Rising expectations from pet owners in combination with the humanization of pets may further increase complexity. Effective diagnostics are vital to identifying appropriate treatment procedures and evaluating therapies. There is a need to expand the understanding of diagnostic‐related incidents to identify risks, and thereby facilitate improvements. This study aimed to explore which were the most common types of diagnostic‐related incidents and their severity by analyzing incident reports.

**Methods:** Descriptive statistical analysis was utilized to characterize key features in incident reports relating to diagnosis recorded between April 2018 and December 2021. The reports were gathered through a company‐specific voluntary reporting system in 63 small animal practices in mainland Europe.

**Results:** A total of 322 incident reports were analyzed, of which 62% (n=201) for dogs, 36% (n=116) for cats, 1% (n=3) for rabbits and 1% (n=2) for birds and exotics. The majority, 59% (n=189) did not result in patient harm. Of the remaining incidents, 24% (n=77) resulted in temporary harm, 6% (n=20) in permanent harm and 11% (n=36) in patient death. Most commonly harm was caused when the diagnostics were incompletely or inadequately performed or because they were not performed when indicated.

**Conclusion:** These findings suggest what type of diagnostics‐related incidents impose the most risk and thereby may inform further exploration of where to focus improvement initiatives.

## 202 Abstract OT03

### 202.1 A Force‐Activated Separation Device Reduces the Rate of Intravenous Catheter Complications in Dogs

#### 202.1.1 Kristin M. Zersen; Sydney Simpson

##### 202.1.1.1 Veterinary Teaching Hospital, Colorado State University

**Background:** Intravenous catheter (IVC) complications may include extravasation, dislodgement, phlebitis, and occlusion. These complications may have serious consequences for the patient including infection, pain, venous depletion, and failure to deliver prescribed treatments.

**Objective:** To determine if the use of a force‐activated separation device (FASD) reduces the rate of IVC complications in hospitalized dogs.

**Animals:** 367 dogs hospitalized on IV fluids were included in this study.

**Methods:** A prospective, randomized clinical trial was performed. Hospitalized dogs receiving intravenous fluids were randomized to the FASD group (n=180) or to the control group (n=187). Dogs in the FASD group had the FASD attached to their IVC according to manufacturer instructions, and the date/time of each separation was documented. For dogs in both groups, all IVC complications were documented and each complication was classified as extravasation, phlebitis, dislodgement, occlusion, or line breakage.

**Results:** The IVC complication rate in the FASD group was 8.9% and the IVC complication rate in the control group was 24.5%. There was a significant decrease in the number of patients that suffered an IVC complication (p=0.004) and in the total number of IVC complications reported in the FASD group (p≤0.001).

**Conclusions/Clinical Importance:** The use of a FASD significantly reduces the rate of IVC complications in hospitalized dogs receiving intravenous fluids. Reducing IVC complications can reduce client costs, minimize pain for the dog, and ensure delivery of all prescribed treatments in a timely manner.

## 203 Abstract OT04

### 203.1 Evaluation of a Multiple‐Mini Interview Format for Small Animal Internal Medicine Residency Programs

#### 203.1.1 **Tracy Hill** ^1^; Jo Smith^2^

##### 203.1.1.1 ^1^University of Minnesota; ^2^Associate Professor, Department of Small Animal Medicine and Surgery, University of Georgia

**Background:** The multiple‐mini interview (MMI) format is utilized by some veterinary schools and multiple human medical training programs to evaluate candidates. The MMI is correlated with candidate success, is more objective, and is perceived as fairer compared to traditional interview formats.

**Hypothesis/Objectives:** To assess residency candidates’ opinions of an MMI format.

**Animals:** Residency candidates over a 3‐year period.

**Methods:** 4 MMI stations were developed based on AAVMC clinical competencies. Candidates were selected for interview based on their VIRMP application. Interviews were performed in person or virtually. Match rank of candidates was based solely on MMI score. Before match day, each interviewee was sent a Qualtrics survey to assess their impressions of the MMI interview experience.

**Results:** 63 interviewees completed the survey. Most (71%) interviewed via video chat or phone. The MMI was evaluated as fairer by 70% (95% CI, 57%, 81%). 51% (95% CI, 38%, 64%) of interviewees felt the MMI allowed them to demonstrate their strengths. Interviewees found the MMI more stressful (83%, 95% CI 71%, 91%). Overall, 63% (95% CI 50%, 75%) would rank this program more highly based on their experience with the process.

**Conclusions and Clinical Importance:** MMIs can feasibly be used for residency interviews in veterinary medicine. Because interviewees perceived the process as fairer and were more likely to rank the program more highly because of the program, residency training programs could consider replacement of a traditional interview with an MMI format. The correlation between MMI performance and resident candidate success needs to be evaluated in specialty veterinary medicine.

## 204 Abstract OT05

### 204.1 Prevalence of Owner‐Reported Medical Conditions in the Most Popular Breeds in the Dog Aging Project

#### 204.1.1 Kiersten K. Forsyth^1^; Kate Creevy^2^, DVM, MS, DACVIM (SAIM); Brianah McCoy^3^, BS; Sarah Schmid^4^, DVM, DACVIM (SAIM)

##### 204.1.1.1 ^1^College of Veterinary Medicine and Biomedical Sciences, Texas A&M University; ^2^Associate Professor, Small Animal Clinical Sciences, College of Veterinary Medicine and Biomedical Sciences, Texas A&M University; ^3^PhD Student, Snyder‐Mackler Lab, School of Life Sciences and Center for Evolution and Medicine, Arizona State University; ^4^Clinical Instructor, Small Animal Internal Medicine, School of Veterinary Medicine, University of Wisconsin‐Madison

**Background:** Data on the prevalence of various medical conditions across dog breeds in the United States is sparse.

**Hypothesis/Objectives:** To estimate the prevalence of medical conditions most common among dogs in the United States and determine if purebred dogs have an increased prevalence of medical conditions compared to mixed‐breed dogs.

**Animals:** 27,541 companion dogs living across the United States.

**Methods:** This cross‐sectional study evaluated owner‐reported survey data collected through the Dog Aging Project (DAP) Health and Life Experience Survey. The 10 most commonly reported medical conditions in each of the 25 most popular dog breeds enrolled in the DAP were determined. Prevalence estimates for these conditions were compared between mixed‐breed and purebred populations. Dogs with no owner‐reported medical conditions were identified.

**Results:** A total of 53 medical conditions made up the top 10 conditions for the 25 most popular breeds. The prevalence of dogs with no owner‐reported medical conditions was not significantly different (p=0.796) between purebred (22.26%) and mixed‐breed dogs (20.74%). The medical conditions reported most frequently across breeds were dental calculus (24/25), dog bites (23/25), extracted teeth (21/25), osteoarthritis (15/25), and Giardia (15/25).

**Conclusions and Clinical Importance:** The data suggest that purebred dogs do not show an increased prevalence of medical conditions compared to mixed‐breed dogs. However, individual breeds often show an increased prevalence for specific conditions. Dental disease is an important health concern commonly reported by owners. Dog bites are also common and occur in both small and large breed dogs.

## 205 Abstract OT06

### 205.1 Establishing a Frailty Phenotype for Aging Dogs

#### 205.1.1 **Katharine Russell**; Gilad Fefer, DVM; Alejandra Mondino Vero, DVM, MSc; Natasha Olby, Vet MB, PhD, MRCVS, DACVIM (Neurology)

##### 205.1.1.1 North Carolina State University

**Background:** Frailty is a well‐established clinical state in people that correlates with mortality and impacts treatment decisions; however, few measures of frailty have been validated in dogs.

**Objectives:** To develop a simple and reliable method of measuring frailty phenotype in older dogs.

**Animals:** Fifty‐one client‐owned dogs participating in a longitudinal study on aging.

**Methods:** Criteria for frailty in 5 domains (nutritional status, sarcopenia, mobility, activity, and exhaustion) were selected from questionnaire and physical examination data. Receiver operating characteristic curve analysis was used to define frailty cutoffs for each criterion. Cox proportional hazard analysis was performed to evaluate 6‐month survival.

**Results:** The following criteria were identified to define frailty in each domain: poor body condition or appetite; severe epaxial muscle atrophy; owner‐reported poor mobility; owner‐reported low activity; owner‐perceived exhaustion. Each domain was individually associated with 6‐month mortality (p<0.05), (sensitivity 36–88%; specificity 70–100%). Dogs were classified as overall frail (10/51 dogs) if they met the frailty criteria for two or more domains. Overall frailty was associated with 6‐month mortality (p=0.001), (hazard ratio, 8.01 [95% CI, 1.8–36.7]; sensitivity 78%; specificity 83%). Total frailty score (the sum of frail domains) was associated with mortality (p<0.001) (hazard ratio, 2.7 [95% CI, 1.8–4.09]).

**Conclusions:** This study defines a frailty phenotype which is simple to perform and positively correlates with 6‐month mortality, allowing it to act as an aid for clinical decision making. It is now being validated in a large prospective study.

## 206 Abstract OT07

### 206.1 Hepatic Dearterialization in Dogs and Cats with Massive or Diffuse Liver Tumor

#### 206.1.1 **Michelle Nguyen**; Chick Weisse, VMD, DACVS; Stacy Kaneko

##### 206.1.1.1 The Animal Medical Center

**Background:** Transarterial coil embolization of the hepatic artery may provide a palliative option for non‐resectable massive or diffuse liver tumors in dogs and cats.

**Hypothesis:** Hepatic dearterialization is a safe palliative management option in dogs/cats with liver tumors.

**Animals:** Four dogs and two cats with massive or diffuse liver masses evaluated at the Animal Medical Center (AMC).

**Methods:** A retrospective review of patients who underwent transarterial coil embolization of the hepatic artery from the origin of the gastroduodenal artery to the proximal hepatic artery. Medical records were reviewed for patient signalment, clinical signs, biochemical changes, cross sectional imaging, complications, and response to treatment. Bloodwork and tumor dimensions were recorded prior to, and approximately six weeks following hepatic dearterialization.

**Results:** All six patients survived to discharge and four (4/6) were discharged 24 hours after treatment. Two (2/6) patients experienced mild short‐term vomiting and anorexia, one of whom required repeat hospitalization. Elevated pre‐treatment hepatocellular enzymes exponentially increased 24 hours post‐operatively in all patients. Repeat bloodwork analysis ~6 weeks after treatment demonstrated improved ALT and AST in 4/5 patients evaluated and improved ALP in 3/5 patients when compared to pre‐treatment levels. Repeat imaging performed and post‐mortem results demonstrated tumor regression in 4/4 patients evaluated. Overall survival times ranged from 60–505 days (MST 197.4 days).

**Conclusion:** Hepatic dearterialization was performed successfully in all six patients with limited reported side effects. Tumor regression and improved hepatocellular enzymes were appreciated in most patients approximately 6 weeks after treatment.

## 207 Abstract OT08

### 207.1 Medical Errors: The Experiences, Attitudes, and Perspectives of Incoming and Outgoing Fourth‐Year Veterinary Students

#### 207.1.1 **Cordelia A. Alexander‐Leeder**; Sarah Guess, DVM, MS, DACVIM (SAIM); Denise Waiting, LVT; Elizabeth Davidow, DVM, DACVECC

##### 207.1.1.1 Washington State University

**Background:** Medical errors impact veterinary patient safety and students can help prevent errors. Medical error research is relevant to ACVIM and there are no other studies that address the role of veterinary students in patient safety.

**Objective:** To assess and compare the experiences and attitudes of incoming and outgoing fourth‐year veterinary students regarding medical errors, adverse events (AEs), near misses (NMs), and error disclosure.

**Sample:** Classes of 2021 and 2022 veterinary students (n=261).

**Methods:** An electronic survey was distributed to the Classes of 2021 (Class21) and 2022 (Class22) during April 2021. Response data were summarized and compared between class year groups with a Fisher's Exact Test and Mann‐Whitney U Test.

**Results:** Response rate was 26.8% (70/261). Most respondents (85.7%) reported being present during a medical error; and 60% reported causing a medical error. Class21 indicated lower agreement with documenting an error in the patient record and whether all errors should be disclosed. Class22 felt more distress surrounding potential errors and consequential career implications. Class21 agreed more that errors occur frequently in veterinary medicine and disagreed more that hospital staffing is adequate to ensure patient safety compared to Class22. Open responses recognized a need for communication training and identified that Class21 regarded errors actionably, whereas Class22 viewed errors more emotionally.

**Conclusions and Clinical Importance:** Most veterinary students will experience medical errors prior to graduation, and some appear to lack clarity around appropriate disclosure and documentation practices. Additional training on errors and disclosure may improve both patient safety and veterinary mental health.

## 208 Abstract OT09

### 208.1 Efficacy and Safety of Long‐Term Oral Imepitoin for Control of Canine Storm‐Associated Anxiety and Fear

#### 208.1.1 **Ana Clara Munoz** ^1^; Jennifer Maulini^1^; Jiashu Zhao^2^; Emily Griffith, PhD^3^; Margaret Gruen^4^, DVM, MVPH, PhD, DACVB

##### 208.1.1.1 ^1^College of Veterinary Medicine, North Carolina State University; ^2^Department of Statistics, North Carolina State University; ^3^Associate Research Professor, Department of Statistics, North Carolina State University; ^4^Assistant Professor of Behavioral Medicine, Department of Clinical Sciences, College of Veterinary Medicine, North Carolina State University

**Background:** Imepitoin is a low‐affinity partial agonist for benzodiazepine binding sites of gamma‐aminobutyric acid (GABAa) receptors with anxiolytic effects. It has been shown to reduce anxiety during noise‐related events in dogs when given at 30 mg/kg BID, although this dose was associated with ataxia and increased appetite.

**Objectives:** To assess safety and efficacy of imepitoin for storm anxiety starting at 10 mg/kg BID and titrating to effect up to 30 mg/kg during storm season.

**Animals:** Thirty‐three healthy client‐owned adult dogs diagnosed with storm anxiety participated and received medication.

**Methods:** This was a prospective, open‐label dose‐escalation study. Refer to Figure 1 for study design. Data were collected via owner survey (once weekly and per‐storm), and treatment periods were compared to baseline using matched pairs analysis.

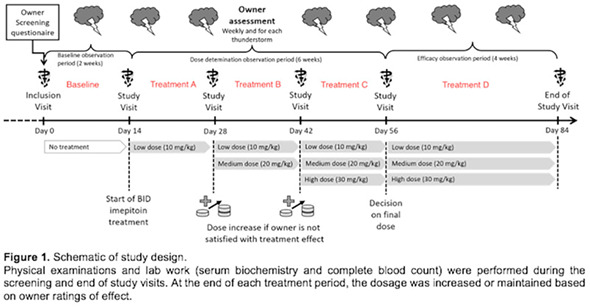

**Results:** Significant decreases in anxiety scores were seen in weekly surveys and decreases in anxiety scores were seen in storm logs (Table 1). Serious adverse events (AE) were not reported in any subject. Ataxia was the most commonly reported AE (14/33), followed by increased hunger (13/33). AEs occurred more frequently in the 20 mg/kg BID group than 10 mg/kg BID. No clinically significant changes were seen in lab work pre‐ and post‐study.

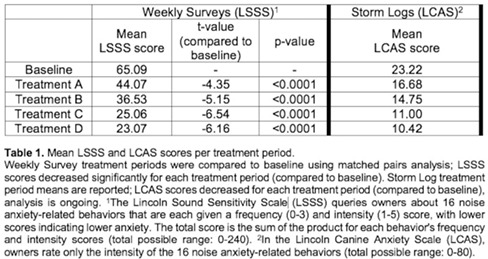

**Conclusions:** We recommend a starting imepitoin dosage of 10 mg/kg BID for treatment of fear and anxiety during storms in dogs; if adverse effects occur at 10–20mg/kg BID, with minimal decrease in anxiety, increasing the dosage is associated with more adverse effects than a further reduction in anxiety. Imepitoin at an individually titrated dose was effective for dogs in this study.

## 209 Abstract OT10

### 209.1 The PETSORT Statement: Reporting Guidelines for Randomized Controlled Trials Conducted in Dogs and Cats

#### 209.1.1 **Audrey Ruple** ^1^; Annette O'Connor^2^, BVSC, MVSC, DVSC, FANZCVSC; Jan Sargeant^3^, DVM, MSc, PhD, FCAHS; Laura Selmic^4^, BVetMed (Hons), MPH, DACVS‐SA, DECVS, ACVS Founding Fellow, Surgical Oncology, ACVS Founding Fellow, Maxillofacial and Oral Surgery

##### 209.1.1.1 ^1^Virginia Tech; ^2^Professor and Chairperson, Large Animal Clinical Sciences, Michigan State University; ^3^Professor, Population Medicine, University of Guelph; ^4^Associate Professor, Department of Veterinary Clinical Sciences, The Ohio State University

**Background:** Comprehensive reporting of randomized clinical trials (RCTs) is essential for the reader to evaluate the methodological rigor of the trial and interpret the generalizability. However, a recent study evaluating comprehensive reporting of published RCTs using dogs and cats found that there were significant deficiencies in reporting trial methods and other information crucial for interpretation of trial results. The Consolidated Standards of Reporting Trials (CONSORT) statement is used to improve reporting of published RCTs conducted in human populations.

**Objectives:** To create standardized guidelines based on the CONSORT statement to aid researchers in reporting RCTs conducted using dogs and cats.

**Animals:** None.

**Methods:** A consensus group of 52 experts in clinical trial design, biostatistics, epidemiology, and subject matter specialists worked together to modify the CONSORT (Consolidated Standards of Reporting Trials) statement to reflect the unique aspects of reporting RCTs conducted in small animals.

**Results:** The consensus group work resulted in the production of the PETSORT statement for reporting RCTs in dogs and cats and a 25‐item checklist that authors can use to ensure complete reporting.

**Conclusions and Clinical Importance:** The use of the PETSORT statement, which addresses issues unique to companion animal trials, when designing and publishing RCTs should improve the quality of study design and reporting for trials conducted in dogs and cats.

## 210 Abstract OT11

### 210.1 End of Life Survey—Free Text Analysis

#### 210.1.1 **Vanessa E. Wilkins** ^1^; Kelly McNulty^2^, DVM; Kate Creevy^3^, DVM, MS, DACVIM (SAIM); Annette Fitzpatrick^4^, PhD; Audrey Ruple^5^, DVM, MS, PhD, DACVPM, MRCVS

##### 210.1.1.1 ^1^College of Veterinary Medicine, Texas A&M University; ^2^Veterinary Resident, Small Animal Veterinary Teaching Hospital, College of Veterinary Medicine, Texas A&M University; ^3^Professor, Small Animal Clinical Sciences, College of Veterinary Medicine, Texas A&M University; ^4^Research Professor, Family Medicine, Epidemiology and Global Health, University of Washington; ^5^Associate Professor, Population Health Sciences, Virginia‐Maryland College of Veterinary Medicine

**Background:** End‐of‐Life Survey (EOLS) is a questionnaire created by the Dog Aging Project (DAP) and completed by owners with the purpose of collecting canine cause and manner of death and additional factors associated with death. Many EOLS respondents provided free‐text responses in addition to forced‐choice responses.

**Hypothesis/Objectives:** We hypothesized that by comparison to forced‐choice response, the free‐text responses:

- Would not provide additional medically relevant information about canine death
- Would provide additional information about owner decision‐making

**Animals:** Owners of deceased DAP dogs throughout the U.S. completed EOLS (n=646).

**Methods:** For nineteen (19) multiple‐choice questions, participants had the option to choose “other, please describe” and utilize a free‐text box. At the end of EOLS, participants were asked if there was any additional information they wished to share regarding the death of their dog, and they were provided a free‐text box to respond (“additional narrative information”).

**Results:** Analysis of both forms of free‐text responses within EOLS did not reveal any novel medically relevant information about canine death. In the additional narrative information, owners reported unique themes regarding thoughts, feelings, and perceptions of their experiences around the time of their dog's death.

**Conclusions and Clinical Importance:** EOLS survey design and available forced‐choice responses effectively captured the medically relevant information about canine death without the need for free‐text elaboration. In the additional narrative information, owners provided a novel source of data regarding their motivations and decision‐making processes around the time of their dog's death, which may better equip veterinary practitioners to support bereaved dog owners.

## 211 SMALL ANIMAL INTERNAL MEDICINE – PHARMACOLOGY

## 212 Abstract P01

### 212.1 The Effect of Feeding on the Pharmacokinetics of Telmisartan Oral Solution in Dogs

#### 212.1.1 **Allison G. Bechtel** ^1^; Zhong Li^2^, PhD; Jennifer Reinhart^3^, DVM, PhD, DACVIM (SAIM), DACVCP

##### 212.1.1.1 ^1^University of Illinois Urbana‐Champaign; ^2^Director, Metabolomics Lab, Roy J. Carver Biotechnology Center, University of Illinois Urbana‐Champaign; ^3^Assistant Professor, Veterinary Clinical Medicine, University of Illinois Urbana‐Champaign

**Background:** Telmisartan is an angiotensin receptor blocking drug that has great potential to improve the treatment of cardiovascular disease, renal disease, and hypertension in dogs. A telmisartan oral solution (TOS) is approved for use in cats, but the pharmacokinetics of this formulation have not been established in dogs. Previous studies have reported decreased bioavailability of telmisartan in cats and humans when administered with food, but whether the same is true for dogs is currently unknown.

**Hypothesis/Objectives:** To establish the pharmacokinetics of TOS and determine the effect of feeding on drug absorption in dogs.

**Methods:** Seven healthy dogs were included in this two‐way, balanced, randomized, cross‐over study. In two phases separated by a 7‐day washout period, dogs were administered 1 mg/kg of TOS orally with or without food and underwent serial measurement of plasma telmisartan concentrations over 24 hours.

**Results:** No significant differences were found between the fasted and fed states in maximum concentration (253.46±139.12 vs. 241.84±121.85 ng/mL), time to maximum concentration (1.32±0.66 vs. 1.46±1.18 h), area under the curve (1376.06±454.68 vs. 1239.05±369.13 ng/mL), or terminal half‐life (6.32±2.13 vs. 5.35±1.12 h).

**Conclusions and Clinical Importance:** These results indicate that TOS may be administered with or without food in dogs without a detrimental effect on bioavailability. However, additional pharmacokinetic and pharmacodynamic studies are warranted to confirm this recommendation and establish therapeutic targets to provide rational dosage recommendations for telmisartan in dogs.

## 213 Abstract P02

### 213.1 Prednisolone Prescribing Practices for Dogs in Australia

#### 213.1.1 **Bonnie L. Purcell** ^1^; Julien Dandrieux^2^, BSc, DrMedVet, PhD, DACVIM (SAIM); Anke Wiethoelter^3^

##### 213.1.1.1 ^1^University of Melbourne; ^2^Senior Lecturer and Head of Small Animal Medicine and Oncology Service, University of Melbourne; ^3^Senior Lecturer in Veterinary Epidemiology (One Health), University of Melbourne

**Background:** Prednisolone is a routinely prescribed medication for a variety of medical conditions. There is a lack of information regarding prescribing practices by veterinarians for dogs in Australia.

**Hypothesis/Objectives:** To describe the demographic of dogs receiving prednisolone in Australia. To describe the disease processes treated with prednisolone in this population. To describe the average dose in mg/kg and mg/m^2^ and frequency of doses used in the physiologic, anti‐inflammatory and immunosuppressive range. To report on the frequency of inappropriately high doses of prednisolone being used.

**Animals:** Two thousand Australian dogs prescribed prednisolone.

**Methods:** Retrospective medical record review using Australian Vet Compass records from July 2016 to July 2018. Search terms were used to identify dogs prescribed prednisolone containing products and a random sample of 2000 records were taken for review.

**Results:** Median dose prescribed was 0.83 mg/kg/day (20.66 mg/m^2^/day) and 58.4% of prescriptions were between 0.3 and 1.0 mg/kg/day. Prescriptions were predominantly (82.2%) for disease of the skin and ears. 152 dogs (7.6%) were prescribed immunosuppressive doses of prednisolone for conditions where an anti‐inflammatory dose would be recommended. Median age was 73 months, weight 17 kg and the most common breed category were terriers (24.5%). The most common breeds were Staffordshire bull terrier, Labrador retriever and Maltese terrier.

**Conclusions and Clinical Importance:** Prednisolone is primarily used as an anti‐inflammatory, particularly for skin and ear disease in this population of Australian dogs. Inappropriate use of immunosuppressive doses of prednisolone, for inflammatory conditions, occurs in a sizeable minority of prescriptions.

## 214 Abstract P03

### 214.1 Prevalence of Antibiotic Use for Cats and Dogs in U.S. Veterinary Teaching Hospitals, August 2020

#### 214.1.1 **Jennifer L. Granick** ^1^; Amanda Beaudoin^2^, DVM, PhD, DACVPM; Emma Bollig^3^, MPH

##### 214.1.1.1 ^1^College of Veterinary Medicine, University of Minnesota; ^2^Director of One Health Antibiotic Stewardship, Minnesota Department of Health; ^3^Program Manager, Epidemiologist for Antimicrobial Stewardship, Veterinary Clinical Sciences, College of Veterinary Medicine, University of Minnesota

**Background:** Point prevalence surveys of antibiotic use (AU) in small animal medicine can identify opportunities to improve antibiotic prescribing practices and track progress.

**Objectives:** To estimate prevalence of AU for dogs and cats treated at United States veterinary teaching hospitals and to describe the most common antibiotics prescribed, indications for use, and evidence of infections associated with each prescription.

**Animals:** Cross‐sectional survey of medical records from cats and dogs evaluated by a veterinarian on primary and urgent care, emergency and critical care, internal medicine, and surgery services on a single day at 14 veterinary teaching hospitals.

**Methods:** Medical record data, including signalment, clinical service, inpatient or outpatient status, clinical problems, diagnostic tests, and name, dose, route of antibiotics prescribed on the study date or the day prior, were entered into a secure online database.

**Results:** Overall AU prevalence was 36.5% (95% CI 33.3–39.6) with 57.7% (235/407) of hospitalized and 18.3% (87/476) of outpatient cats and dogs prescribed an antibiotic during the study. For both dogs and cats cefazolin, ampicillin‐sulbactam, amoxicillin‐clavulanic acid, and enrofloxacin were the most prescribed systemic antibiotics. Of the 365 prescriptions for dogs, surgical (91, 24.9%), respiratory (38, 10.4%), dermatologic (28, 7.7%), and urinary (23, 6.3%) were the most common indications. For cats, common indications included surgical (11/87, 12.6%), urinary (9/87, 10.3%), and hepatic (7/87, 8.0%).

**Conclusions and Clinical Importance:** Use of broad‐spectrum antibiotics is common in the teaching hospital setting. Further analysis of appropriateness will help determine where reductions in prescribing would be safe and effective.

## 215 Abstract P04

### 215.1 Evaluating the Effects of Telmisartan in Healthy Dogs as a Preclinical Model for Shar‐Pei Fever

#### 215.1.1 **Kara M. Maslyn** ^1^; Jennifer Hawley^1^; Michael Lappin^2^, DVM, PhD, DACVIM; Craig Webb^2^, PhD, DVM, DACVIM; Tracy Webb^2^, DVM, PhD

##### 215.1.1.1 ^1^Colorado State University; ^2^Clinical Sciences, Colorado State University

**Background:** Chinese Shar‐Pei dogs commonly develop Shar‐Pei fever, a pro‐inflammatory disease characterized by fever of unknown origin, renal amyloidosis, peritonitis, synovitis, and proteinuria. While traditional therapies help alleviate clinical signs, optimal therapies remain unknown. Telmisartan is an angiotensin II receptor blocker used primarily to help control hypertension and proteinuria in dogs, but other effects have been described.

**Hypothesis/Objectives:** The aim of this study was to evaluate the immunomodulatory properties and antioxidant effects of telmisartan in healthy dogs.

**Animals:** Eight healthy, mixed sex, purpose‐bred research beagles were used for this study.

**Methods:** Dogs were administered telmisartan 10 mg once daily for 28 days. A complete blood count (CBC), serum cytokine/chemokine panel, and the following antioxidant assays were performed six times over the course of the study: total antioxidant capacity (TAC), superoxide dismutase (SOD), catalase (CAT), glutathione reductase (GR), and glutathione peroxidase (GPX). Group mean values after starting telmisartan administration were compared to baseline values by ANOVA with significance defined as P<0.05.

**Results:** While significant changes in CBC or cytokines/chemokines were not detected, telmisartan significantly upregulated the activity of serum TAC (P<0.007), serum SOD (P<1.78E‐5), and plasma GPX (P<0.001) over the 28 day trial.

**Conclusions/Clinical Importance:** Telmisartan has antioxidant effects in healthy dogs, which suggests an additional indication to use this drug in the management of Shar‐Pei fever. A clinical trial designed to evaluate the therapeutic effects of telmisartan in dogs affected by Shar‐Pei fever is ongoing.

## 216 Abstract P05

### 216.1 Pharmacokinetics and Anti‐Nausea Effects of Intravenous Ondansetron in Hospitalized Dogs Exhibiting Clinical Signs of Nausea

#### 216.1.1 **Cindy Sotelo** ^1^; Kristin Zersen^2^, DVM, DACVECC; Daniel Gustafson^3^; Jessica Quimby^4^; Sarah Shropshire^3^, DVM, PhD, DACVIM

##### 216.1.1.1 ^1^Internal Medicine, Veterinary Teaching Hospital, Colorado State University; ^2^Clinical Sciences, Veterinary Teaching Hospital, Colorado State University; ^3^Veterinary Teaching Hospital, Colorado State University; ^4^Veterinary Teaching Hospital, The Ohio State University

**Background:** Ondansetron is a 5‐HT_3_ receptor antagonist used to treat nausea and vomiting in dogs.

**Objectives:** Evaluate the pharmacokinetics of intravenous (IV) ondansetron in a population of hospitalized dogs exhibiting clinical signs of nausea.

**Methods:** Twenty‐four dogs were randomly assigned to one of the following IV ondansetron protocols: 1 mg/kg q12h, 0.5 mg/kg q12h, 1 mg/kg q8h, 0.5 mg/kg q8h. Serum was collected at 0, 0.25, 0.5, 1, 2, 4, 8, 16, and 24 h after the first dose and nausea scores were recorded at multiple time points. Ondansetron and arginine vasopressin (AVP) concentrations were measured via HPLC and ELISA, respectively. Groups were compared with non‐parametric analyses as appropriate.

**Results:** In the 0.5 mg/kg group, mean C_max_=214 ng/ml, AUC_0‐8h_=463 ng/ml*h, and calculated half‐life was 1.9 h. In the 1 mg/kg group, mean C_max_=541 ng/ml, AUC_0‐8h_=1057 ng/ml*h and calculated half‐life was 1.6 h. Serum ondansetron levels were not significantly different between dogs that required rescue anti‐nausea medication (median 116.2; range 44.9–605.3) and dogs that did not require rescue therapy (118.8; 13.4–460.9). Nausea scores at 4 hours (2; 0–4) (P=0.002) and 8/12 hours (1; 0–6) (P<0.0001) were significantly decreased from baseline (3; 2–4). AVP levels were variable and did not correlate with nausea scores (r=0.1662; 95% CI ‐0.37–0.62).

**Conclusion and Clinical Relevance:** Ondansetron displayed linear pharmacokinetics. Nausea scores decreased regardless of dosage protocol and AVP was not a reliable biomarker of nausea in this group of dogs.

## 217 Abstract P06

### 217.1 Reversal of the Effects of a Medetomidine‐vatinoxan Combination Drug (Zenalpha) with Atipamezole

#### 217.1.1 Heta Turunen

##### 217.1.1.1 Vetcare Ltd

**Background:** The ability to reverse the effects of a novel canine sedative containing α_2_‐agonist medetomidine and α_2_‐antagonist vatinoxan (Zenalpha) with atipamezole increases the appeal for use in short‐lasting clinical examinations and procedures.

**Hypothesis/Objectives:** The objective of this study was to demonstrate that atipamezole can be used safely and effectively to reverse the effects of Zenalpha.

**Animals:** Eight healthy purpose‐bred, instrumented Beagle dogs.

**Methods:** This was a randomized, blinded, experimental cross‐over study. The dogs received IM label doses of medetomidine (1 mg/m^2^) and vatinoxan (20 mg/m^2^), followed by atipamezole (5 mg/m^2^) 30 min later. Control group did not receive atipamezole. Heart rate (HR), ECG and mean arterial pressure (MAP) were recorded continuously, and sedation was assessed at intervals, until 120 min. Differences between treatments and baseline were evaluated with a linear mixed model and Dunnett adjustment for multiple comparison (*p*<0.05).

**Results**

Recoveries were calm and smooth without relapses into sedation. HR and MAP increased and were significantly higher, and sedation scores decreased and were significantly lower from 10 minutes following atipamezole administration until the end of the observation period when compared to control group. HR, MAP and sedation scores returned to baseline values following atipamezole administration within 10, 30 and 40 min, respectively. Single 1–5 min lasting periods of mild, moderate or marked sinus tachycardia were observed in the ECG after atipamezole administration in 50% of the dogs probably due to physiological responses.

**Conclusions and Clinical Importance:** The effects of Zenalpha were successfully reversed by atipamezole without safety concerns.

## 218 Abstract P07

### 218.1 Clinical Audit of POM‐V/POM Prescriptions by Remote Consultation via a Veterinary Video Telemedicine Smartphone Application

#### 218.1.1 **Adele Williams** ^1^; Sam Davies^2^, Bsc; Tamsin Day^3^, BVetMed, MRCVS; Trevor Hardcastle^4^, BSc, MSc, PhD; Sheila Smith^5^, BVMS, MRCVS; Samantha Webster^6^, BVetMed, MRCVS

##### 218.1.1.1 ^1^Vet‐AI; ^2^Data Analyst, Tech, Vet‐AI; ^3^Veterinary Researcher, Telehealth Excellence, Vet‐AI; ^4^Chief Data Scientist, AI and Research, Vet‐AI; ^5^Veterinary Researcher, Telehealth Excellence, Vet‐AI; ^6^Clinical Exec, Clinical, Vet‐AI

**Objective:** To assess outcomes of a limited period (7 months) of remote video consultation with prescribing of POM/POM‐V medications by RCVS registered veterinary surgeons to UK clients via a veterinary telemedicine smartphone application.

**Background:** Objective evidence is needed to inform the veterinary profession on the impact that remote prescribing, without physical examination in person, has on animal health and welfare. During the COVID‐19 pandemic, the RCVS allowed remote prescribing temporarily.

**Methods:** Clinical records from all veterinary video consultations from 1st April thru 31st October 2020, were reviewed. Details were assessed pertaining to signalment, body condition/disease categories managed, referrals into practice, medication classes prescribed and outcomes following POM‐V/POM medications. Records of adverse events and antimicrobial prescribing were reviewed.

**Results:** The 16.6% (n=3,541/21,383) video consults had a POM‐V/POM prescribed; with a (mild) adverse event rate of 0.7% (n=30/4,282). Antibacterials were prescribed in 5.9% of all consultations (n=1,267/21,383), 99.3% (n=1258/1267) being first line. Follow up on prescribing was available in 67.9% (n=2,907/4,282) of cases. 89.4% (n=2598/2907) of all known treatment outcomes were complete/expected response to treatment. Dermatological disease was the most common body system disease category seen and prescribed for.

**Conclusion:** Low prescribing rates (including antibacterials) were recorded, treatments were efficacious and no harm was done by prescribing remotely via a veterinary video consult app.

**Application:** Veterinary surgeons and governing bodies are invited to use the information provided in this clinical audit to inform decisions on the suitability of remote consultations and prescribing in veterinary medicine.

**Figure:**
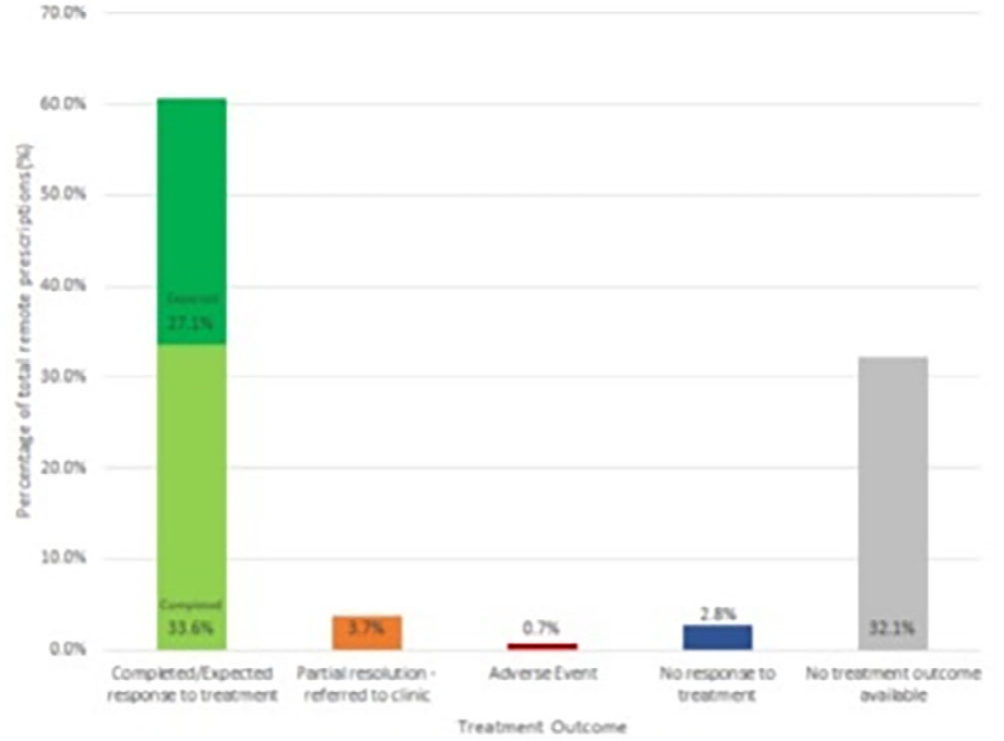
Image 1

**Figure:**
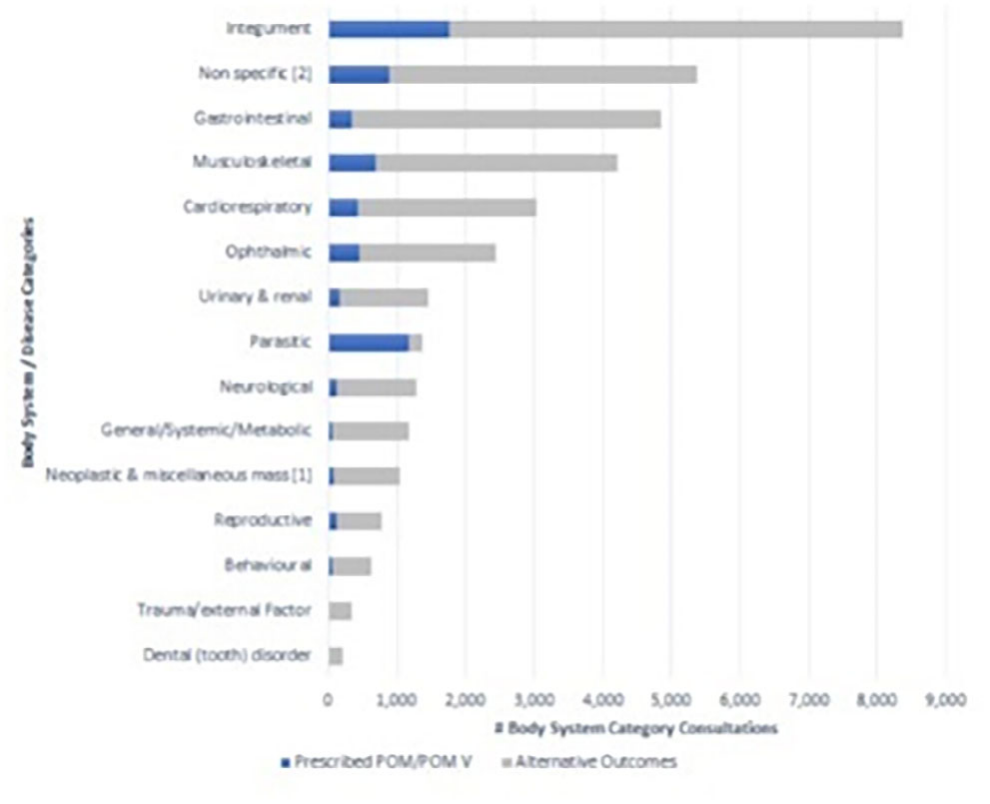
Image 2

## 219 Abstract P08

### 219.1 Plasma Pharmacokinetics of a Hypoxia‐Inducible Factor Prolyl Hydroxylase Inhibitor (Molidustat) in Healthy Cats

#### 219.1.1 Kristine Fraatz^1^; Ralph Krebber^2^

##### 219.1.1.1 ^1^Elanco Animal Health; ^2^Bayer AG

**Background:** Molidustat is under development for treatment of cats suffering from non‐regenerative anemia associated with chronic kidney disease. Molidustat is presented as sodium salt in a 2.5% (m/v) oily suspension used for once daily oral dosing of 5 mg molidustat sodium per kg.

**Objectives:** The pharmacokinetic (PK) profile of molidustat was investigated in a series of three GLP‐compliant PK studies.

**Animals:** Fifty‐two healthy laboratory adult cats.

**Methods:** Oral bioavailability was derived in 8 cats receiving a single dose of 5 mg/kg molidustat either orally or intravenously. Dose proportionality was investigated at 2.5, 5, and 10 mg/kg representing 0.5X, 1X and 2X the intended therapeutic dose in 8 cats each. Molidustat PK profile was also determined after repeated once daily dosing in 8 cats using 5 mg/kg for 6 days. Excretion pathways were explored after single oral and intravenous dosing of 5 mg/kg to 6 cats each. Bioanalytical analyses were performed using a validated HPLC‐MS/MS method.

**Results:** After a single oral dose, molidustat reached a maximum plasma concentration of 3.83 mg/L one hour after dosing. Oral bioavailability (F=82%) was high with an AUC_last_ of 12.71 mg*h/L. Plasma half‐life was 4.6 hours. Dose proportionality was evident. No accumulation was observed (Accumulation Index = 1.1). Molidustat underwent extensive hepatic metabolism, mainly N‐glucuronidation. Unchanged molidustat was excreted via feces (~40% of dose) with minor contributions from the renal route (<0.5%).

**Conclusions and Clinical Importance:** The derived PK profile of molidustat in cats supports the proposed once daily 5 mg/kg dosing regimen.

## 220 Abstract P09

### 220.1 Fluconazole Has Variable Oral Systemic Absorption in Dogs

#### 220.1.1 **Butch KuKanich**; Kate KuKanich, DVM, PhD, DACVIM (SAIM); Geraldine Magnin, PhD, DABT

##### 220.1.1.1 College of Veterinary Medicine, Kansas State University

**Background:** Fluconazole is commonly administered to dogs, but there are few published data on the fluconazole pharmacokinetics. A population pharmacokinetic study in dogs with systemic fungal disease demonstrated large unexplained variability in fluconazole drug exposure.

**Hypothesis/Objectives:** Our hypotheses were feeding would decrease fluconazole bioavailability and large variability across manufacturers would occur. The objective of this study was to assess the effects of feeding and manufacturer on the oral relative bioavailability of fluconazole in dogs.

**Animals:** The KSU IACUC approved the study. Six healthy purpose‐bred dogs weighing 9.5–13.7 kg were enrolled.

**Methods:** Fluconazole tablets (100 mg) from three manufacturers (Citron Pharma, Glenmark Pharmaceuticals, Harris Pharmaceutical) were administered separately in a randomized crossover block to 12‐hour fasted and fed dogs (i.e., six treatments per dog). The washout period was at least 10 days between treatments. Blood was collected and fluconazole plasma concentrations were determined with mass spectrometry. Pharmacokinetics were calculated with computer software.

**Results:** All dogs completed the study (dose range 9.5–13.7 mg/kg). The overall variability in dose normalized drug exposure (AUC/dose) was large, ranging from 1.9–2.9x within each treatment, while the overall variability across all treatments was even larger, 3.2‐fold. The mean fed relative oral bioavailability was lower (82–90%) compared to fasted for each formulation, and only a 1.3‐fold range of mean AUC/dose occurred across all six treatments.

**Conclusions and Clinical Importance:** These data suggest feeding and manufacturer were only minor contributors to the overall large variability in fluconazole drug exposure in dogs.

## 221 Abstract P10

### 221.1 Voluntary Acceptance of Compounding Flavors in Cats

#### 221.1.1 **Amy Nichelason** ^1^; Kelly Schultz^1^, DVM; Alyssa Bernard^1^, PharmD, CVT; Elizabeth Alvarez^2^, DVM, DABVP (Canine and Feline Practice); Juliet Caviness^1^, DVM

##### 221.1.1.1 ^1^School of Veterinary Medicine, University of Wisconsin; ^2^Clinical Assistant Professor, School of Veterinary Medicine, University of Wisconsin

**Background:** Providing oral medications to feline patients is often difficult. Many medications come as unpalatable liquids, tablets or capsules that can be problematic to administer. Because of this challenge, veterinarians often seek compounding flavors to enhance compliance. However, few published studies exist evaluating flavoring agents in cats.

**Hypothesis/Objectives:** The primary aim of this study is to determine which 10 commercially available compounding flavors cats accept. A secondary aim is to determine whether owner perception of acceptance is a validated metric based on residual sample weight.

**Animals:** 46 healthy employee‐owned cats between the ages of 1 and 12 years.

**Methods:** A randomized, blinded prospective controlled trial. Each cat was randomly offered 10 compounding flavors. Owners graded enjoyment on a 1–7 Likert‐type scale. Residual sample weights were calculated to determine amount ingested.

**Results:** Cats accepted oil‐based flavors significantly more than water‐based flavors (p<0.001) (95% CI [‐1.24, ‐0.65]). Chicken and fish were the most enjoyed oil flavors and liver was the least accepted oil flavor. Tuna was the most accepted water flavor. Vanilla butternut and marshmallow flavors were less accepted. Owner perception of acceptance was a valid metric for assessing flavor acceptance (p<0.001) (R=‐0.47).

**Conclusions and Clinical Importance:** This data suggests that oil‐based compounding flavors should be selected when available and sweet flavors should be avoided. This information can be used to select compounding flavors for feline patients to increase compliance. This study also found that owner perception can be used to assess flavor acceptance.

## 222 SMALL ANIMAL INTERNAL MEDICINE ‐ RESPIRATORY

## 223 Abstract R01

### 223.1 Infrared Thermography and 6MWT for Assessment of Thermoregulation in Dogs with Brachycephalic Obstructive Airway Syndrome

#### 223.1.1 **Jeremy Gallman** ^1^; Tekla Lee‐Fowler^2^; Stuart Clark‐Price^2^; Megan Grobman^2^, DVM, MS, DACVIM (SAIM), PhD

##### 223.1.1.1 ^1^College of Veterinary Medicine, Auburn University; ^2^Auburn University

**Background:** Brachycephalic obstructive airway syndrome (BOAS) is associated with significant morbidity and mortality. Routine clinical evaluation is insufficient to detect physiologic consequences of BOAS including exercise intolerance and impaired thermoregulation. Infrared thermography (IRT) has been used in dogs to assess thermoregulation. Combined use of a six‐minute walk test (6MWT) and IRT may aid clinical management by assessing the physiologic consequences of BOAS.

**Hypothesis/Objectives:** Compare 6MWT and IRT parameters between healthy mesocephalic (Meso) and brachycephalic (Brachy) dogs, and dogs with clinical BOAS (BOAS).

**Animals: 3**1 adult companion dogs: Meso (n=16), Brachy (n=9), and BOAS (n=6).

**Methods:** Prospective study. 6MWT parameters (normalized distance (ND), rectal temperature, pulse, respiratory rate, and SPO2) and IRT temperatures (T_mean_) at 3 regions of interest over the head/neck (rostral, dorsoventral, pharyngeal) were collected. Evaluation timepoints were pre‐6MWT, T_0_, T_5min_, and T_15min_. Comparisons were made by Mann‐Whitney Rank Sum Test, One‐way ANOVA on Ranks, and Spearman Rank Order correlation (*p*<0.05).

**Results:** No significant difference in ND or rectal temperatures were found between groups (*p*>0.05). No differences in pharyngeal IRT were found between groups at baseline (*p*>0.05). Significantly increased pharyngeal temperatures were detected at T_0_ for BOAS compared to Meso (*p*=0.031), and at T_5min_ for BOAS compared to Meso (*p*=0.01) and BOAS compared to Brachy groups (*p*=0.01). Pharyngeal IRT did not correlate with ND or rectal temperatures (*p*>0.05).

**Conclusions and Clinical Importance:** IRT may detect subclinical impaired thermoregulation, characterized by increases in pharyngeal T_mean_ in dogs with clinical BOAS, independent of rectal temperature or ND.

## 224 Abstract R02

### 224.1 Videofluoroscopic Swallow Study Diagnosis of Aerodigestive Disorders in Dogs

#### 224.1.1 **Jennifer Howard** ^1^; Carol Reinero^2^, DVM, PhD, DACVIM (SAIM); Megan Grobman^3^, DVM, MS, PhD, DACVIM (SAIM)

##### 224.1.1.1 ^1^University of Missouri; ^2^Professor, Small Animal Internal Medicine, University of Missouri; ^3^Assistant Professor, Small Animal Internal Medicine, Auburn University

**Background:** Aerodigestive diseases are hybrid disorders representing a pathologic link between the respiratory and alimentary tracts. Dogs presenting solely for respiratory clinical signs without dysphagia, vomiting or regurgitation do not receive diagnostics targeting identification of alimentary tract disease. Videofluoroscopic study (VFSS) can identify many abnormalities.

**Objectives/Hypothesis:** Comparing dogs with respiratory but not alimentary clinical signs to healthy controls, we hypothesized the former would have more VFSS abnormalities and a higher penetration‐aspiration score (PAS).

**Animals:** Client‐owned dogs with respiratory signs (n=40) and healthy controls (n=15).

**Methods:** Prospectively, all dogs had VFSS. Advanced respiratory diagnostics were performed in respiratory dogs. VFSS metrics (n=8) were subjectively assessed and a PAS score (scale of 1–7) assigned. Fishers Exact test compared differences between groups (presence or absence of VFSS abnormalities); PAS score was compared via Mann‐Whitney Rank Sum test (p<0.05 significant).

**Results:** Dogs with respiratory disease had significantly more abnormalities on VFSS compared to controls (31/40 vs 6/15 dogs; p=0.021). Penetration was noted in 7/40 and 2/15 affected and control dogs, respectively. Aspiration was noted only in dogs with respiratory disease (6/40). PAS score did not differ between groups (median (IQR) 1 (1–3) affected and 1 (1–2) control dogs, respectively; p=0.577).

**Conclusions:** Significantly more VFSS abnormalities were identified in affected (78%) versus control (40%) dogs underscoring the need to identify and address silent alimentary tract disease. The unexpected high prevalence of VFSS abnormalities in controls merits follow‐up to determine if these dogs developed respiratory disease.

## 225 Abstract R03

### 225.1 Development of a 3D‐Printed Canine Airway Model as a Simulator for Canine Bronchoscopy

#### 225.1.1 **Julien Dandrieux** ^1^; Dongjin Kang^2^; Stewart Ryan^1^

##### 225.1.1.1 ^1^Faculty of Veterinary and Agricultural Sciences, University of Melbourne; ^2^University of Melbourne

**Background:** Utilising live animals or cadavers for canine bronchoscopy training is problematic due to ethical and biohazard reasons, and risks to patients when novices conduct live procedures. Currently, there are no commercially available canine bronchoscopy simulators on the market. A canine bronchoscopy simulator would be valuable for teaching airway anatomy and endoscope handling skills prior to *in vivo* training for bronchoscopy.

**Hypothesis/Objectives:** This study aimed to create a canine airway model with excellent anatomical fidelity via 3D‐printing and to face and content validate the model as a canine bronchoscopy simulator.

**Animals:** A CT scan of a normal dog's thorax was used for modelling.

**Methods:** The canine airway model was created utilising 3D Slicer (Slicer Org.) and Meshmixer (AutoCad Inc.). Two models were created with proprietary build materials for two 3D printers: Accura ClearVue, ProJet 7000HD (3D Systems Inc.), and ABSm30 Fortus 900 (Stratasys Ltd.). The ABSm30 model was used for the validation study.

The validation study consisted of veterinarians (n=4) with bronchoscopy experience conducting a standard bronchoscopic examination and a 12‐question, 5‐point Likert scale survey.

**Results:** The participants gave high approval ratings for the model's teaching utility and preference for using the model to teach first‐year residents. A wide range of ratings were observed for questions regarding the build's rigidity.

**Conclusions and Clinical Importance:** The ABSm30 model has anatomical fidelity and could be utilised to teach veterinary novice students in bronchoscopy. Both models require further optimisations to improve endoscope access and textual realism of the model.

## 226 Abstract R04

### 226.1 Evaluation of Sobetirome for Pulmonary Fibrosis in West Highland White Terriers

#### 226.1.1 **Elizabeth Rozanski** ^1^; Luis dos Santos^2^; John Rush^3^; Lindsay Merkel^4^

##### 226.1.1.1 ^1^Tufts University; ^2^Cardiologist, Purdue; ^3^Professor, Tufts University; ^4^Professor, University of Minnesota

**Background:** Pulmonary fibrosis (PF) a fatal disease with no current therapy. In a mouse model, the thyroid mimetic, sobetirome, ameliorated histopathological evidence of fibrosis.

**Hypothesis/Objectives:** Sobetirome will improve exercise tolerance and decrease pulmonary hypertension in dogs with PF. Therapy with sobetirome will decrease cholesterol and T4 without other side effects.

**Animals:** Healthy beagles, West Highland White terriers (WHWT) with PF.

**Methods:** Safety study; Six beagles were administered Sobetirome over 2 weeks. Pilot clinical trial. WHWT were evaluated with non‐sedated CT, echocardiography for determination of tricuspid regurgitant velocity (TRV), 6 minute walk test (6MWT) and cholesterol/T4 measurement at baseline and after at least 6 months of treatment. Results were compared using a paired T‐test or Mann‐Whitney Rank‐Sum.

**Results:** All beagles completed the study without complications. Cholesterol and T4 decreased (P<0.001). In WHWT, 13 dogs were recruited, and 6 dogs completed the study. Of the 7 dogs that did not complete the study, three died of PF, two owners withdrew and two moved. 5/6 dogs had TRV >3.2 m/sec at enrollment, and this increased over the study. (p=0.01) Cholesterol and thyroid decreased insignificantly (p=0.17 and p=0.15). 6MTW was unchanged (p=0.71).

**Conclusions and Clinical Importance:** Sobetirome is well‐tolerated in dogs and decreases cholesterol and T4 in beagles. PF is a heterogenous disease with variable progression. There was no clear benefit to therapy in this small pilot study of dogs with PF. Clinical trials during COVID were challenging.

## 227 Abstract R05

### 227.1 Metabolomic Profiling of Bronchoalveolar Lavage Fluid in Pet Cats with Asthma and Non‐Asthmatic Respiratory Diseases

#### 227.1.1 **Aida I. Vientos‐Plotts** ^1^; Hans Rindt^2^, PhD; Aaron Ericsson^3^, DVM, PhD; Carol Reinero^4^, DVM, DACVIM (Small Animal Internal Medicine), PhD

##### 227.1.1.1 ^1^College of Veterinary Medicine, University of Missouri; ^2^Senior Research Associate, Veterinary Medicine and Surgery, College of Veterinary Medicine, University of Missouri; ^3^Assistant Professor, Veterinary Pathobiology, University of Missouri; ^4^Professor, Veterinary Medicine and Surgery, College of Veterinary Medicine, University of Missouri

**Background:** The lung microbiota, characterized in spontaneous and experimentally asthmatic cats, deviates from health. Taxonomic studies are limited by inability to provide functional information including metabolic profiles. Alterations in the lower airway microbiota affect the generation of local metabolites which may have implications for disease development or progression.

**Objectives/Hypothesis:** We hypothesize gas chromatography/mass spectrometry (GC‐MS) will allow characterization of bronchoalveolar lavage fluid (BALF) metabolites and show distinct differences between asthmatic and non‐asthmatic cats.

**Animals:** Client‐owned cats with asthma (n=8) and non‐asthmatic respiratory disease (n=5) undergoing BALF collection.

**Methods:** Banked BALF was processed using GC‐MS and analyzed with MetaboAnalyst. PERMANOVA was used to compare overall metabolite composition between groups and a volcano plot was used to identify significant differences in metabolites between groups (p<0.05 significant).

**Results:** The metabolome in BALF of asthmatic cats was significantly different compared to non‐asthmatic cats (p=0.0155; F=5.256). A volcano plot showed 13 metabolites had at least a 2‐fold difference and p<0.5 between asthmatic and non‐asthmatic cats. While most of the metabolites are unknown, threonic acid, ribose and arabinose were significantly higher in asthmatic cats.

**Conclusions:** Using GC‐MS, metabolites were detected in BALF, a dilute sample reflecting the respiratory environment. Specific metabolites including threonic acid, ribose and arabinose were significantly different between asthmatic and non‐asthmatic cats. Future studies should focus correlating the microbiota with the metabolome in asthmatic cats.

## 228 Abstract R06

### 228.1 Effectiveness of Pediatric Inhaler Chambers in Cats with Asthma

#### 228.1.1 **Mark Nagel** ^1^; Nathan Hoffman^2^, MSc; Shae Sartori^3^

##### 228.1.1.1 ^1^Trudell Medical International; ^2^Laboratory Technician, Aerosol Lab, Trudell Medical International; ^3^Sales and Marketing Manager, Animal Health, Trudell Medical International

**Background:** Pediatric chambers are sometimes used to deliver inhaled medications to animals, but aerosol delivery has never been validated against feline anatomy or respirology.

**Objectives:** To determine if chambers designed for human infants are effective at delivering medication to feline lower airways where needed.

**Animals:** 3D Cat Anatomical Respiratory Inhalation Needs Model (CARIN) based on feline MRI/CT scans.

**Methods:** Delivery of fluticasone propionate (Flovent‐HFA 110‐mcg) from two common pediatric chambers (AeroChamber,* BabyHaler) with different masks (conical/round), size (small/large), and antistatic properties were compared against the AeroKat* using CARIN coupled to a breathing simulator. Performance was assessed by the amount of drug delivered to the modelled lower airways measured via HPLC of particle mass deposited on a filter at the distal end of CARIN. The amount of drug retained in the chamber and mask leakage (percent drop in continuous flow rate (10‐l pm) from the chamber's inhaler port to CARIN's outlet‐mask placement optimized to minimize leakage) were measured to validate performance differences.

**Results:** AeroKat delivered 38–193x more to the lower airways than the human pediatric chambers (see Figure). Round masks leaked less, but BabyHaler's shallower design provided a worse muzzle seal. The AeroChamber leaked most but delivered more than the BabyHaler which retained the most drug in the chamber, possibly due to its larger volume relative to cats’ tidal flow and lack of antistatic properties.

**Figure:**
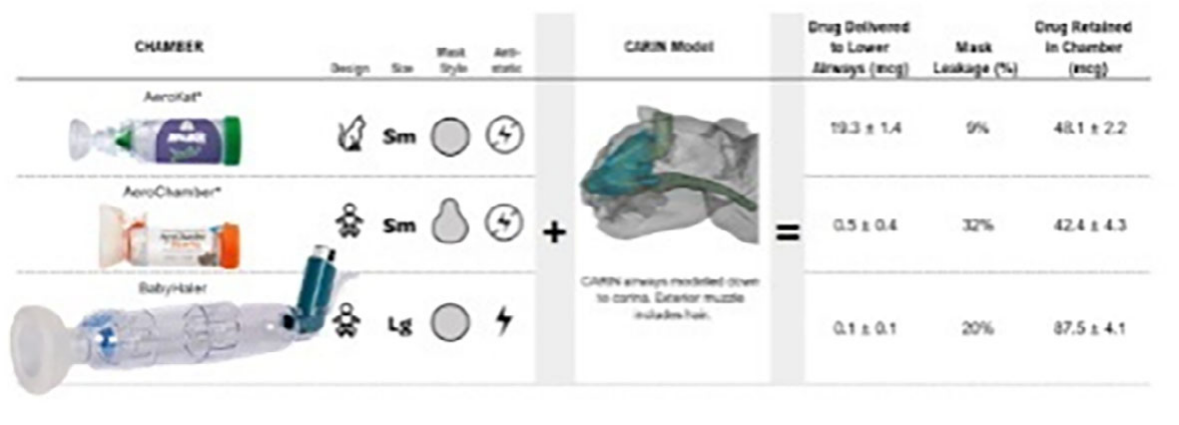
Figure 1

**Conclusions:** Chambers designed for infants/pediatrics disregard cat anatomy and airway physiology. For effective feline delivery, the custom designed cat chamber as tested would be required.

## 229 Abstract R07

### 229.1 Study of Nasal Microbiome in Dogs with Nasal Diseases and Healthy Dogs

#### 229.1.1 **Yuta Nakazawa** ^1^; Ryoko Kibe^2^; Takafumi Ohshima^3^; Naoko Yayoshi^4^; Yuji Hamamoto^4^; Yasuyuki Negishi^5^; Michio Fujita^3^; Aki Fujiwara‐Igarashi^3^

##### 229.1.1.1 ^1^Nippon Veterinary and Life Science University; ^2^Veterinary Microbiology, Nippon Veterinary and Life Science University; ^3^Veterinary Radiology, Nippon Veterinary and Life Science University; ^4^Veterinary Medical Teaching Hospital, Nippon Veterinary and Life Science University; ^5^Microbiology and Immunology, Nippon Medical School

**Background:** Bacterial communities are thought to affect the host's immune system and are associated with various autoimmune diseases in dogs, but there are only a few reports of their changes and roles in canine nasal diseases.

**Hypothesis/Objectives:** The purpose of this study was to evaluate and compare the nasal microbiome in dogs diagnosed with nasal diseases and healthy dogs.

**Animals:** Nine healthy dogs, five dogs diagnosed with nasal neoplasia, and six dogs with non‐infectious rhinitis.

**Methods:** Nasal swabs were collected using three methods: without anesthesia, under anesthesia, and posttreatment with nasal flashing using saline. Bacterial DNA was extracted and 16S rRNA gene sequencing was performed. The data was analyzed using Quantitative Insights Into Microbial Ecology 2.

**Results:** A total of 22 bacterial phyla were detected in the nasal cavity of the dogs. Among the collection methods, operational taxonomic units were significantly increased in posttreatment with nasal flashing compared to pretreatment (*p*=0.026). *Moraxella* spp. was the most common species in healthy dogs regardless of the collection methods, whereas it was decreased in nasal neoplasia and non‐infectious rhinitis. In nasal neoplasia, *Neisseriaceae* and *Pasteurellaceae* were present in an increased amount, and no significant difference was observed between them.

**Conclusion and Clinical Importance:** This study revealed differences in the tendency of the nasal microbiome among collection methods and between healthy dogs and dogs with nasal diseases. These differences may help elucidate the pathology of nasal diseases influenced by commensal microbiota, and thereby, lead to the use accurate antibiotics for their treatment.

## 230 EQUINE

## 231 Abstract E01

### 231.1 Investigating the Relationship Between Cardiac Function and Insulin Sensitivity in Horses

#### 231.1.1 **Natasha Williams** ^1^; Michael Davis^2^, DVM, PhD, DACVIM (LAIM), DACVSMR; Veronique Lacombe^2^, DVM, PhD, DACVIM (LAIM), DECEIM; Allison Campolo^3^, PhD; Cristobal Navas de Solis^3^, LV, MS, PhD, DACVIM (LAIM); Martin Furr^2^, DVM, PhD, DACVIM (LAIM), MA Ed

##### 231.1.1.1 ^1^Hagyard Equine Medical Institute; ^2^Oklahoma State University; ^3^Alcon Research LLC; ^4^University of Pennsylvania

**Background:** Metabolic syndrome in humans is commonly associated with cardiovascular dysfunction, including atrial fibrillation and left ventricular diastolic dysfunction. Although many differences exist between human and equine metabolic syndrome, both of these conditions share some degree of insulin resistance.

**Hypothesis/Objectives:** The aims of this study were to investigate the relationship between magnitude of insulin resistance and degree of myocardial dysfunction. We hypothesised that insulin sensitivity would not be correlated with changes in cardiac function, as measured with tissue Doppler imaging (TDI)‐ and 2‐dimensional speckle tracking (2DST)‐derived indices of systolic and diastolic function.

**Animals:** 7 research horses

**Methods:** Each horse underwent insulin‐modified frequently sampled intravenous glucose tolerance testing to determine insulin sensitivity (I_S_), and echocardiography including TDI and 2DST. Pearson and Spearman correlation analyses were used to determine associations between insulin sensitivity and echocardiographic measures of cardiac function.

**Results:** The study population included 5 mares and 2 geldings (age 17±4.2 years, weight 524±73 kg), with a mean I_S_ of 2.21±0.03 × 10^‐4^ L/min/mU. I_S_ was found to be significantly correlated with peak myocardial velocity during late diastole (r=0.89, *P*=0.0419), ratio between peak myocardial velocity in early and late diastole (r=−0.92, *P*=0.0263), isovolumetric relaxation time (r=−0.97, *P*=0.0072), and isovolumetric contraction time (ρ=−0.90, *P*=0.0374).

**Conclusions:** These data demonstrated that decreased insulin sensitivity is correlated with alterations in both systolic and diastolic function, as measured with tissue Doppler imaging (TDI). Due to the small sample size of this study, the relationship between insulin sensitivity and myocardial function requires further investigation.

## 232 Abstract E02

### 232.1 The Effect of Metformin on the Insulin Response to Oral Sugar in Insulin‐Dysregulated Horses

#### 232.1.1 **Sarah F. Colmer** ^1^; Amanda Adams^2^, PhD; Emma Adam^2^, PhD, DACVIM, DACVS; Rachel Miller^3^; Darko Stefanovski^4^; Jeaneen Kulp^5^; Andrew Van Eps^6^

##### 232.1.1.1 ^1^School of Veterinary Medicine, University of Pennsylvania; ^2^Associate Professor, Gluck Equine Research Center, Department of Veterinary Science, University of Kentucky; ^3^Department of Clinical Sciences, College of Veterinary Medicine, Lincoln Memorial University; ^4^Associate Professor of Biostatistics, New Bolton Center, Department of Clinical Sciences, School of Veterinary Medicine, University of Pennsylvania; ^5^Research Specialist, New Bolton Center, Department of Clinical Sciences, School of Veterinary Medicine, University of Pennsylvania; ^6^Associate Professor, Equine Musculoskeletal Research, New Bolton Center, Department of Clinical Sciences, School of Veterinary Medicine, University of Pennsylvania

**Background:** A single dose of metformin administered 1 h prior to oral glucose challenge was previously shown to reduce insulinemic responses in horses with experimentally‐induced insulin dysregulation (ID). This effect could be useful for controlling post‐prandial hyperinsulinemia in horses with naturally‐occurring ID.

**Hypothesis/Objectives:** The objective was to compare insulinemic and glycemic responses to oral sugar testing (OST) performed at different intervals after a single dose of metformin in horses with naturally‐occurring ID. We hypothesized that metformin would significantly decrease the insulinemic response to OST.

**Animals:** Eight university‐owned adult horses with naturally‐occurring ID

**Methods:** In a randomized crossover design, OST was performed 1 h, 2 h and 6 h following a single oral dose of metformin (30 mg/kg) or after placebo (60 ml water) in all horses with a 7‐day washout between. Plasma insulin, c‐peptide and glucose concentrations were measured at 0, 60 and 90 min after 0.45 ml/kg Karo Light corn syrup and the effect of treatment (and interval since dosing) examined using a mixed effects linear regression model.

**Results:** Metformin treatment had no significant effect on plasma glucose, insulin or c‐peptide concentrations at any time point compared to placebo (p>0.05). For OST 1 h post metformin, median (IQR) plasma insulin was 91.3 uIu/mL (62.4–114.9) at 60 min vs 76.2 uIu/mL (59.1–134.5) for placebo (p=0.8) and 62.7 uIu/mL (31.4–109.7) at 90 min vs 51.8 uIu/mL (29.2–126.3) for placebo (p=0.9).

**Conclusions and Clinical Importance:** The results do not support the use of targeted metformin treatment to reduce post‐prandial hyperinsulinemia in horses with naturally‐occurring ID.

## 233 Abstract E03

### 233.1 Evaluation of Commonly Used Field‐Testing Protocols to Diagnose Insulin Dysregulation and the Association with Laminitis

#### 233.1.1 **Brianna L. Clark** ^1^; Allison Stewart^2^, BVSc (Hons I), MS, DACVIM, DACVECC, PhD, MANZCVS; Kate Kemp^3^, BSc, BEquineSc (Hons); Nicholas Bamford^4^, BVSc (Hons), MANZCVS, DACVIM, PhD; Francois‐Rene Bertin^5^, DVM, MS, PhD, DACVIM (LAIM)

##### 233.1.1.1 ^1^The University of Queensland; ^2^Senior Lecturer, The University of Queensland; ^3^PhD student, The University of Queensland; ^4^Lecturer, The University of Melbourne; ^5^Associate Professor, The University of Queensland

**Background:** Insulin dysregulation (ID) consists of hyperinsulinemia (HI) with or without insulin resistance (IR). Various protocols have been proposed but not evaluated to determine laminitis risk.

**Hypothesis/Objectives:** Evaluate the oral sugar test (OST) and 2‐step insulin tolerance test to diagnose ID the association with laminitis.

**Animals:** One hundred and forty‐four ponies.

**Methods:** Insulin concentrations >20 or >50 μIU/mL at baseline or >45 or >65 μIU/mL at 60 or 90 minutes after oral administration of 0.45 mL/kg of corn syrup defined HI. Reduction of blood glucose concentration to <50% of baseline after intravenous administration of 0.1 IU/kg insulin was defined IR. Laminitis was defined as modified‐Obel score >1. Descriptive statistics and receiver operating characteristic curves were performed.

**Results:** Overall, 31% ponies had laminitis. Basal HI was present in 15% (20‐μIU/mL cut‐off) and 10% (50‐μIU/mL cut‐off) of ponies. On OST, HI was present in 42% and 45% (45‐μIU/mL cut‐off at 60 and 90 minutes respectively), and 35% and 38% (60‐μIU/mL cut‐off at 60 and 90 minutes respectively) of ponies. Fifty‐three percent were IR. All ponies with basal HI had post‐OST HI; however, 69% with post‐OST HI did not have basal HI. Of IR ponies, 77% did not have basal HI and 27% did not have post‐OST HI. A 45‐μIU/mL cut‐off at 60 minutes and IR had better sensitivities and specificities, with laminitis as the outcome.

**Conclusions and Clinical Importance:** With an incomplete overlap between HI and IR, performing both tests improves evaluation of ID and laminitis.

## 234 Abstract E04

### 234.1 Assessment of the Hypothalamic‐Pituitary‐Adrenocortical Axis Function Utilizing a Vasopressin Stimulation Test in Healthy Foals

#### 234.1.1 **Erin M. Elder** ^1^; Hannah Robertson^1^; David Wong^2^, DVM, DACVIM; Katheryn Johnson^3^, DVM; Meghan Marner^4^; Katarzyna Dembek^5^, DVM, DACVIM, PhD

##### 234.1.1.1 ^1^North Carolina State University; ^2^Department Chair, Veterinary Clinical Sciences, Iowa State University; ^3^Large Animal Internal Medicine Resident, Iowa State University; ^4^Iowa State University; ^5^Assistant Professor, Equine Internal Medicine, North Carolina State University

**Background:** Sepsis is a major cause of death in foals and associated with hypothalamic‐pituitary‐adrenal axis (HPAA) dysfunction. HPAA function can be evaluated by utilizing an arginine‐vasopressin (AVP) stimulation test.

**Hypotheses/Objectives:** Administration of AVP will stimulate a rise in systemic adrenocorticotropin‐releasing hormone (ACTH) and cortisol in healthy foals.

**Animals:** Eleven healthy foals

**Methods:** HPAA function was assessed in foals utilizing three doses of AVP (2.5, 5, 7.5 IU), administered between 24–48 h of age in this randomized cross‐over study. Cortisol, ACTH, and corticotropin‐releasing hormone (CRH) were measured at 0 (baseline), 15, 30, 60 and 90 minutes after AVP administration with immunoassays. A fold increase 15 and 30 minutes from baseline was calculated for cortisol and ACTH.

**Results:** All doses of AVP resulted in a significant increase of cortisol concentration over time, and a dose‐dependent increase of ACTH concentration over time. ACTH and cortisol increased 15 and 30 minutes, respectively after all three doses of AVP compared to baseline (P<0.01). A 50‐fold increase in cortisol was seen with 5 and 7.5, and a 20‐fold increase with 2.5 IU. A 10‐fold increase in ACTH was seen with 7.5 IU, 8‐fold increase with 5 IU, and 4‐fold increase with 2.5 IU (P<0.05). There was no effect of AVP on endogenous CRH.

**Conclusion and Clinical Importance:** Administration of AVP is safe and results in a significant rise in ACTH and cortisol in healthy foals. A stimulation test with AVP (5 IU) can be considered for HPAA assessment in septic foals.

## 235 Abstract E05

### 235.1 The Enteroinsular Axis in Hospitalized Foals with Gastrointestinal Disease

#### 235.1.1 **Hannah M. Kinsella** ^1^; Ahmed Kamr^2^, PhD; Laura Hostnik^3^, DVM, MS, DACVIM; Julia Horton^4^; Jamie Summers^4^; Stephen Reed^5^, DVM, DACVIM; Nathan Slovis^6^, DVM, DACVIM; Teresa Burns^7^, DVM, MS, PhD, DACVIM; Ramiro Toribio^8^, DVM, MS, PhD, DACVIM

##### 235.1.1.1 ^1^The Ohio State University; ^2^Faculty, Veterinary Medicine, University of Sadat City; ^3^Assistant Professor, Veterinary Clinical Sciences, College of Veterinary Medicine, The Ohio State University; ^4^College of Veterinary Medicine, The Ohio State University; ^5^Rood and Riddle Equine Hospital; ^6^Hagyard Equine Medical Institute; ^7^Associate Professor, Veterinary Clinical Sciences, College of Veterinary Medicine, The Ohio State University; ^8^Professor, Veterinary Clinical Sciences, College of Veterinary Medicine, The Ohio State University

**Background:** Critical illness in the foal causes dysregulation of various endocrine systems, including the enteroinsular axis (EIA) that includes insulin and incretins (glucose‐dependent insulinotropic polypeptide [GIP], glucagon‐like peptide‐1 [GLP‐1], and glucagon‐like peptide‐2 [GLP‐2]). Recently, alterations in incretin concentrations were observed in hospitalized foals.

**Hypothesis/Objectives:** Investigate dynamics of the EIA in critically ill foals with gastrointestinal (GI) disease.

**Animals:** Hospitalized foals (n=109) ≤72h of age were categorized based on admission data: GI disease (n=54), non‐GI disease (NON; n=26), and healthy (H; n=29). Foals with GI disease were further categorized by inflammatory (GI‐I; n=31) and non‐inflammatory (GI‐N; n=23) disease.

**Methods:** Blood samples were collected over 72 h to measure insulin, GIP, GLP‐1, and GLP‐2 concentrations using immunoassays.

**Results:** In all hospitalized foals (GI‐I, GI‐N, NON), GIP was lower at admission and 24 hours compared to H foals (P<0.05). GLP‐1 was increased at admission in GI‐I compared to H foals (P<0.05). GLP‐2 concentrations >13.1 ng/mL were associated with non‐survival in foals with GI disease (GI‐I, GI‐N; OR=13.5; 95% CI=1.4–124.7; P<0.05). Incretin and insulin concentrations were not different between GI‐I and GI‐N foals (P>0.05).

**Conclusions/Importance:** In hospitalized foals, GIP concentrations were lower at admission and 24 hours compared to healthy foals. Alterations in GLP‐1 and GLP‐2 may indicate that GI disturbances induce dysregulation of the EIA. However, differences in the EIA were not apparent between inflammatory and non‐inflammatory conditions.

## 236 Abstract E06

### 236.1 Attenuation of Post‐Prandial Hyperglycemia by 5’‐Adenosine Monophosphate‐Activated Protein Kinase Agonists in Experimentally‐induced Equine Insulin Dysregulation

#### 236.1.1 **Erin F. Pinnell** ^1^; Laura Hostnik^2^, DVM, MS, DACVIM; Mauria Watts^2^; Kathryn Timko^2^, DVM, MS; Allison Thriffiley^2^, DVM; Mercedes Stover^2^; Lauren Koenig^2^; Ramiro Toribio^2^, DVM, MS, PhD, DACVIM; Teresa Burns^2^, DVM, MS, PhD, DACVIM

##### 236.1.1.1 ^1^College of Veterinary Medicine, The Ohio State University; ^2^The Ohio State University

**Background:** 5’‐Adenosine monophosphate‐activated protein kinase (AMPK) agonists, such as metformin (MET) and aspirin (ASP), have been shown to improve experimentally‐induced insulin dysregulation (ID) when co‐administered. Resveratrol (RES) is an AMPK agonist and also inhibits mammalian target of rapamycin (mTOR) signaling, which is activated in laminitis, making this an attractive therapeutic target for equine metabolic syndrome‐associated laminitis (EMSAL).

**Hypothesis/Objectives:** The purpose of this study was to evaluate the effect of combination treatments with RES (10 mg/kg PO q 12 hr), MET (30 mg/kg PO q 12 hr), and ASP (20 mg/kg PO q 24 hr) on experimentally‐induced ID. We hypothesized that co‐administration of AMPK agonists would improve insulin and glucose dynamics in horses with experimentally‐induced ID.

**Methods:** ID was induced in 24 healthy adult light‐breed horses using dexamethasone (0.08 mg/kg PO q 24 hr). Horses were assigned to one of 5 treatment groups: RES, MET/ASP, RES/ASP, RES/MET/ASP, and control (CON). Frequently‐sampled insulin‐modified IV glucose tolerance tests (FSIGTT) and oral sugar tests (OST) were performed at baseline, one week after ID, and after ID plus one week of treatment.

**Results:** Insulin sensitivity (SI) and disposition index (DI) decreased, while basal insulin and glucose concentrations increased following experimentally‐induced ID (p<0.0001). Area under the glucose curve (AUC_g_) during OST decreased after combination treatment with RES/MET/ASP (p=0.0013).

**Conclusions and Clinical Importance:** Dexamethasone‐induced ID alters glucose and insulin dynamics in horses reflected in minimal model parameters. Additionally, treatment with a combination of RES/MET/ASP significantly attenuates post‐prandial hyperglycemia and should be evaluated as a novel treatment for equine ID.

## 237 Abstract E07

### 237.1 Culture‐Enriched 16S RNA Sequencing Profile of Cecal Contents of Horses With and Without Typhlocolitis

#### 237.1.1 **Luiza S. Zakia** ^1^; Diego Gomez^2^, MV, MSc, MVSc, PhD, DACVIM; Patrick Boerlin^3^, PhD; Michael Surette^4^, PhD; Luis Arroyo^5^, Lic. Med. Vet., DVSc, PhD, DACVIM‐LA

##### 237.1.1.1 ^1^Ontario Veterinary College, University of Guelph; ^2^Assistant Professor, Clinical Studies, Ontario Veterinary College, University of Guelph; ^3^Associate Professor, Pathobiology, Ontario Veterinary College, University of Guelph; ^4^Professor, Division of Gastroenterology, Department of Medicine, McMaster University; ^5^Associate Professor, Clinical Studies, Ontario Veterinary College, University of Guelph

**Background:** Homeostasis of the gut microbiota is critical to the health of the host. Next generation sequencing has demonstrated that richness and diversity of the gut microbiota is largely affected in horses.

**Hypothesis/Objectives:** To evaluate the bacterial composition of the cecum content of horses with and without typhlocolitis through direct and culture‐enriched 16S gene sequencing.

**Animals:** Six healthy horses and six horses with typhlocolitis.

**Methods:** Case‐control study. Cecal content was collected after euthanasia. An aliquot was used for direct 16S gene sequencing. Another was serially diluted with Brain Heart Infusion (BHI) and plated onto 5 different agar media. All culture medias, except for MacConkey, were incubated anaerobically. Bacterial colonies were harvested in bulk and used for DNA extraction, 16S PCR amplification, and sequenced using the Illumina MiSeq platform.

**Results:** Predominant phyla in healthy and diseased horses were Firmicutes, followed by Bacteroidetes in all cultured medias, except for the MacConkey, in which Proteobacteria was the dominant phylum (P<0.05). Greater bacterial richness was identified in sequenced cecal contents as compared to cultured plates (P<0.05). In direct 16S gene amplification, the alpha diversity indices were lower in diarrheic horses compared to healthy horses (P<0.05). A higher abundance of Fusobacteria was found in two of the six samples from diarrheic horses.

**Conclusions and Clinical Importance:** Culture‐enriched molecular profiling combined with 16S rRNA gene sequencing offer an alternative method for the study of the gut microbiota of horses. The role of fusobacteria in equine colitis deserves investigation.

## 238 Abstract E08

### 238.1 Effect of Holding Time and Sampling Protocol on VCM‐Vet Parameters Using Fresh Equine Blood

#### 238.1.1 **Sandra Diaz Yucupicio** ^1^; Scott Austin^2^, DVM, MS, DACVIM (LAIM); Rebecca Bishop^3^, DVM, MS; Meghan Fick^4^, DVM, MS, DACVECC; Anne Barger^5^, DVM, MS, DACVP; Bailey Stolsworth^6^, BS; Pamela Wilkins^7^, DVM, MS, PhD, DACVIM (LAIM), DACVECC

##### 238.1.1.1 ^1^College of Veterinary Medicine, University of Illinois; ^2^Associate Professor, Equine Internal Medicine, Veterinary Clinical Medicine, College of Veterinary Medicine, University of Illinois; ^3^Resident in Surgery, Veterinary Clinical Medicine, College of Veterinary Medicine, University of Illinois; ^4^Assistant Professor, Emergency and Critical Care, Veterinary Clinical Medicine, College of Veterinary Medicine, University of Illinois; ^5^Clinical Professor and Section Head of Clinical Pathology, Veterinary Clinical Medicine, College of Veterinary Medicine, University of Illinois; ^6^Veterinary Student, Veterinary Clinical Medicine, College of Veterinary Medicine, University of Illinois; ^7^Professor, Equine Internal Medicine, Veterinary Clinical Medicine, College of Veterinary Medicine, University of Illinois

**Background:** Viscoelastic testing of equine blood has become more common with great potential to improve case management. A point‐of‐care (POC) testing device, the VCM‐Vet, provides practical repeatable results in fresh equine blood.

**Hypothesis/Objectives:** Sample holding time and reuse will affect VCM‐Vet coagulation parameters when using fresh equine blood without anticoagulant.

**Animals:** Eight adult horses from University Research/Teaching herd.

**Methods:** Blood collected by direct jugular venipuncture (18‐ga needle, 3‐ml syringe) was held at 37°C for 2, 4, 6 or 8 minutes according to 1 of 2 protocols. Syringes were gently inverted 2x, a small amount of blood expressed, testing cartridges filled and placed within the VCM‐Vet device. Protocol 1: samples were processed from a single syringe. Protocol 2: 4 syringes were drawn through a single needle. VCM‐Vet measures assessed included Clot Time (CT), Clot Formation Time (CFT), Alpha Angle, Amplitude 10/20 min (A10/A20), Maximal Clot Firmness (MCF) and Lysis Index 30/45 min (LI30/LI45). Differences examined using R, ANOVA Type Statistic (ATS) with Bonferroni adjustment, P≤0.05.

**Results:** Protocol 1: CT decreased over time (P=0.0003), 2–6 min (P=0.023, 2–8 min (P<0.001). Protocol 2: P>0.05 for all parameters. Protocol 1 vs. Protocol 2: There was a significant effect of protocol but not time: CT (P=0.003), CFT (P=0.003), A10 (P<0.001), A20 (P<0.001), MCF (P<0.001).

**Conclusions and Clinical Importance:** Holding time and handling protocol impact VCM‐Vet testing result of fresh blood that is neither anticoagulated nor activated. Samples held unagitated for up to 8 min, while warm, are useful.

## 239 Abstract E09

### 239.1 Plasma Inflammatory Cytokine Profiles in Colitis Cases with and Without Equine Neorickettsiosis (Potomac Horse Fever)

#### 239.1.1 **Laurence Leduc** ^1^; Jeaneen Kulp^2^; Andrew Van Eps^3^, BVSc, DACVIM (LAIM), PhD

##### 239.1.1.1 New Bolton Center, University of Pennsylvania; Research Specialist, New Bolton Center; Associate Professor, New Bolton Center

**Background:** Laminitis appears to be a more common complication of Potomac Horse Fever (PHF) compared with colitis of other etiologies. Differences in the severity of the systemic inflammatory response may be responsible for this and warrant investigation.

**Hypothesis/Objectives:** The objective was to compare plasma inflammatory cytokine profiles between horses with PHF colitis (PHF[+]) and colitis due to other causes (PHF[‐]). We hypothesized that PHF[+] horses would have increased proinflammatory cytokine concentrations in plasma at hospital admission compared to PHF[‐].

**Animals:** 21 client‐owned mixed breed adult horses diagnosed with colitis

**Methods:** In a prospective case‐control study, plasma samples were collected from horses diagnosed with colitis at admission to hospital and analyzed using a multiplex ELISA for inflammatory cytokines including IL‐1β, IL‐6, IL‐8 and TNF‐α. Cytokine concentrations were compared between PHF[+] (n=6) and PHF[−] (n=15) cases using Mann‐Whitney tests.

**Results:** Laminitis was more frequent in PHF[+] (4/6) compared to PHF[−] (0/15) cases (p=0.003). Median (IQR) plasma cytokine concentrations were not different between PHF[+] and PHF[−] cases: IL‐1β (PHF[+] 37.4 [0–98] pg/ml vs PHF[−] 59.8 [0–142.8] pg/ml), p=0.6; IL‐6 (PHF[+] 22.8 [4–51.1] pg/ml vs. PHF[−] 19.5 [0–134] pg/ml), p=0.8; IL‐8 (PHF[+] 95.3 [60–138.3] pg/ml vs. PHF[−] 104 [69–173.5] pg/ml), p=0.8; TNF‐α (PHF[+] 17.8 [10.4–46.9] pg/ml vs. PHF[−] 18 [1–128] pg/ml), p>0.99.

**Conclusions and Clinical Importance:** The results do not indicate that more profound systemic inflammation was responsible for the higher incidence of laminitis in PHF[+] cases in this study.

## 240 Abstract E10

### 240.1 Renal Dysplasia in Horses: 25 Cases (1991–2021)

#### 240.1.1 **Camilo Jaramillo‐Morales** ^1^; K. Gary Magdesian^2^, DVM, DACVIM, DACVECC, DACVCP

##### 240.1.1.1 ^1^University of California, Davis; ^2^Associate Professor, Medicine and Epidemiology, University of California, Davis

**Background:** Renal dysplasia (RD) is a rare congenital disorder representing abnormal nephrogenesis. There are no large studies evaluating the clinical findings associated with RD in horses.

**Hypothesis/Objectives:** The objectives were to describe the clinical findings in horses with RD.

**Animals:** 25 horses diagnosed with RD on histopathology.

**Methods:** Retrospective study of horses with a diagnosis of RD that were presented to the VMTH between 1994 and 2021.

**Results:** 25 horses (15 males,10 females) were included. The most common breeds were Quarter Horse/Paint (n=9), Thoroughbred (n=4) and American Miniature (n=4). Twelve horses were neonates (1–8 days), seven were 3–24 months, and six were 5–24 years. Seven horses had neurological signs associated with dysnatremia. Creatinine concentration had a median of 3.0 (range, 0.9–15.1) and BUN 33 (10–364) mg/dL; sodium was 131.5 (91–152), potassium 4.5 (3.2–8.4) and chloride 88.5 (49–107) mmol/L. Eight horses had a normal creatinine. Five of six cases had ultrasonographic abnormalities. Treatment varied from fluid therapy and slow correction of sodium to hemodialysis and nephrectomy. The most common histopathological findings were primitive mesenchyme (n=8) and fetal glomeruli (n=6). The RD was diffuse in 17 horses and segmental in 8 horses and it was bilateral in 21 horses.

**Conclusions and Clinical Importance:** RD can be found in a variety of breeds and ages of horses and in both sexes. It can be unilateral or bilateral, and segmental or diffuse with or without changes in renal parameters, electrolytes, or ultrasound findings. RD can be an incidental pathologic finding in some horses.

## 241 Abstract E11

### 241.1 Effects of Phenylbutazone, Firocoxib, and Dipyrone on Furosemide‐Induced Diuresis in Horses

#### 241.1.1 **Julianne White** ^1^; Aimee Colbath^2^, VMD, PhD, DACVS‐LA; Harold Schott^3^, DVM, PhD, DACVIM (LAIM)

##### 241.1.1.1 ^1^College of Veterinary Medicine, Michigan State University; ^2^Assistant Professor of Large Animal Surgery and Emergency, Large Animal Clinical Sciences, College of Veterinary Medicine, Michigan State University; ^3^Professor of Large Animal Medicine, Large Animal Clinical Sciences, College of Veterinary Medicine, Michigan State University

**Background:** Administration of NSAIDs to horses to control inflammation via cyclooxygenase enzymes (COX) inhibition may have adverse renal effects. Both COX‐1 and COX‐2 regulate renal blood flow, glomerular filtration, and urinary concentrating ability. Treatment with phenylbutazone (a COX‐1 inhibitor) has been demonstrated to decrease the diuretic and natriuretic effects of furosemide by nearly 30%. The effect of COX‐2 specific inhibitors (firocoxib) and atypical NSAIDs (dipyrone) on the renal effects of furosemide in horses are unknown.

Hypothesis: Furosemide‐induced diuresis after pre‐treatment with firocoxib or dipyrone is diminished to a lesser extent than after treatment with phenylbutazone.

**Animals:** Eight healthy mares.

**Methods:** Each mare received four treatments in a replicated 4×4 Latin Square design: furosemide alone (F), furosemide and firocoxib (FF), furosemide and dipyrone (FD), and furosemide and phenylbutazone (FP). After 2 days of NSAID treatment at recommended dosages, ureteral catheters were placed for continual urine collection. After a 30‐minute baseline collection period, furosemide (1.0 mg/kg IV) was administered, and urine and blood samples were collected for 4 hours. Data were assessed by one‐way repeated measures ANOVA.

**Results:** 4‐hour urine volume was decreased (P<0.001) 20–25% after pretreatment with all NSAIDs, as compared to F, but there were no differences between FF, FD, or FP. Variability in individual responses to furosemide administration after pretreatment with different NSAIDs was observed.

**Conclusions and Clinical Importance:** Though COX‐2 selective NSAIDs have less adverse GI effects in horses, our data suggest there are minimal differences in effects on furosemide‐induced diuresis.

## 242 Abstract E12

### 242.1 Effect of Ambulation Following ^18^F‐fluorodeoxyglucose Injection on Standing Positron Emission Tomography of the Equine Digit

#### 242.1.1 **Andrea D. Oliver** ^1^; Kathryn Wulster^1^; Mathieu Spriet^2^; Sarah Ciamillo^3^; Mathew Ford^3^; Jeaneen Kulp^3^; Andrew van Eps^3^

##### 242.1.1.1 ^1^University of Pennsylvania; ^2^School of Veterinary Medicine, University of California, Davis; ^3^New Bolton Center, University of Pennsylvania

**Background:** Standing ^18^F‐fluorodeoxyglucose (FDG) positron emission tomography (PET) may be useful for diagnosis of laminitis. The effect of ambulatory activity (which is known to affect lamellar microvascular perfusion) following injection on FDG uptake in the lamellae needs to be established.

**Objectives:** Measure FDG uptake in the dorsal lamellae and associated tissues of healthy horses subjected to different ambulatory conditions between the time of FDG injection and subsequent PET image acquisition.

**Animals:** 8 healthy, university‐owned adult horses

**Methods:** In a randomized crossover design, horses were walked (AMB) or cross tied (NON‐AMB) immediately after injection with 3 MBq/kg FDG until scan acquisition (45 minutes later), with steps quantified using an accelerometer. Standardized uptake values (mean [SUVmean] and maximum [SUVmax]) for regions of interest (ROIs) in the mid‐dorsal lamellae and coronary band dermis were compared between AMB and NON‐AMB using Wilcoxon signed rank tests.

**Results:** Median (IQR) step count for AMB (564 [500–644]) was higher than NON‐AMB (63 [30–118]) p=0.02. The SUVmean in the mid‐dorsal lamellae was not different between AMB (1.9 [1.8–2.2]) and NON‐AMB (1.7 [1.3–1.9]) p=0.1. The SUVmax in the dorsal lamellae was not different between AMB (2.7 [2.2–3.0]) and NON‐AMB (2.5 [2.0–2.7]) p=0.5. Coronary band SUVmean for AMB (4 [3.6–4.2] was not different to NON‐AMB (3.8 [3.3–4.6]), p=0.5 and SUVmax AMB (6.8 [6.4–7.6]) was not different to NON‐AMB (6.5 [5.0–7.5]) p=0.1.

**Conclusions:** Mean lamellar FDG uptake was approximately half that of the coronary band and was not affected by ambulatory activity post‐injection in healthy horses.

## 243 Abstract E13

### 243.1 The Effect of Oral Trazodone on Ambulation and Recumbency in Horses

#### 243.1.1 **Kallie J. Hobbs** ^1^; Daniela Luethy^2^, DVM, DACVIM; Jennifer Davis^3^, DVM, PhD, DACVIM (LA), DACVCP; Martha Mallicote^4^, DVM, MBA, DACVIM; Catherine Futterman^5^, BS; Freya Cooper^5^, BS; Catherine Torcivia^6^, VMD; Andrew van Eps^7^, BVSc, PhD, MACVSc, DACVIM

##### 243.1.1.1 ^1^University of Florida; ^2^Clinical Assistant Professor, Large Animal Internal Medicine, University of Florida; ^3^Associate Professor of Clinical Pharmacology, VA‐MD College of Veterinary Medicine; ^4^Clinical Associate Professor, Large Animal Internal Medicine, College of Veterinary Medicine, University of Florida; ^5^Veterinary Student, College of Veterinary Medicine, University of Florida; ^6^Teaching and Research Associate, University of Pennsylvania; ^7^Associate Professor of Equine Musculoskeletal Research, University of Pennsylvania

**Background:** Trazodone may be a useful adjunct therapy in the initial management of acute laminitis cases if it minimizes ambulatory activity and/or encourages recumbency.

**Hypothesis/Objectives:** To evaluate the effects of oral trazodone on ambulatory activity and recumbency in healthy horses.

**Animals/Methods:** In a randomized cross‐over design, 8 healthy research horses housed in stalls received placebo (molasses water) or trazodone at 2 dose rates (2.5 mg/kg and 7.5 mg/kg) orally every 12 h for 48 h with a 14‐day washout between study periods. Forelimb step frequency was measured using a hoof mounted accelerometer and continuous video monitoring was used to detect recumbency. Groups were compared using Friedman's test with post hoc Dunn's test.

**Results:** Step frequency was significantly lower (p=0.008) with 7.5 mg/kg trazodone treatment (median 64 [IQR, 38–96] steps/h) compared to placebo (128 [78–173] steps/h), with a mean step reduction of 44±11%. There was no difference in step frequency with 2.5 mg/kg trazodone (110 [62–177] steps/h) compared to control (p>0.99) or 7.5 mg/kg trazodone (p=0.07). There was no difference in the number of recumbent episodes or total duration of recumbency between any of the treatments (p=0.92).

**Conclusions/Clinical Importance:** Although it did not affect recumbency, trazodone at 7.5 mg/kg q 12 h reduced step frequency by approximately 40%, an effect that may be clinically useful to minimize the mechanical strains associated with ambulation during acute laminitis.

## 244 Abstract E14

### 244.1 Multi‐Dose Misoprostol Pharmacokinetics and Its Effect on the Fecal Microbiome in Healthy, Adult Horses

#### 244.1.1 **Rachel L. Pfeifle** ^1^; Aaron Ericsson^2^, DVM, PhD; Annette McCoy^3^, DVM, MS, PhD, DACVS; Dawn Boothe^4^, DVM, MS, PhD, DACVIM, DACVCP; Anne Wooldridge^1^, DVM, MS, PhD, DACVIM; Erin Groover^1^, DVM, DACVIM; Tamara Sierra‐Rodriquez^1^, DVM, MS, DACVIM; Kara Lascola^1^, DVM, MS, DACVIM

##### 244.1.1.1 ^1^Department of Clinical Sciences, Auburn University; ^2^Department of Veterinary Pathobiology, University of Missouri; ^3^Department of Veterinary Clinical Medicine, University of Illinois; ^4^Department of Anatomy, Physiology, and Pharmacology, Auburn University

**Background:** Misoprostol is administered to treat gastric ulcers in horses and may possess anti‐inflammatory properties. Misoprostol's multi‐dose pharmacokinetics and effect on the fecal microbiome require investigation.

**Hypothesis/Objectives:** To compare the pharmacokinetics between repeated doses and to characterize changes in the fecal microbiome after oral and rectal multi‐dose misoprostol administration.

**Animals:** Six healthy university‐owned geldings.

**Methods:** In a randomized, cross‐over study, misoprostol (5 μg/kg) was administered orally or rectally every 8 hours for 10 doses, or not administered (control), with a 21‐day washout between treatments. Concentration‐versus‐time data for dose 1 and dose 10 were subject to non‐compartmental analysis. For microbiota analysis using 16sRNA amplicon sequencing, manure was collected at −7 days, immediately prior to dose 1, then 6 hours, 7 days, and 14 days after dose 10, with time‐matched points in controls.

**Results:** Repeated dosing related differences in pharmacokinetic parameters were not detected for either administration route. Area under the concentration‐versus‐time curve was greater (p<0.04) after oral versus rectal administration. Relative bioavailability of rectal administration was 4–86% that of oral administration. Microbial composition, richness, and β‐diversity differed among subjects (p<0.001 all). Richness was decreased 6 hours after dose 10 and at the control‐matched timepoint (p=0.0109) in all subjects. No other differences for timepoints, treatments, or their interactions were observed.

**Conclusions and Clinical Importance:** Differences in systemic exposure were associated with route of administration, but were not detected after repeated administration of misoprostol. Differences in microbiota parameters appeared related to inter‐individual variation rather than misoprostol administration.

## 245 Abstract E15

### 245.1 How Sedation and Recumbency Influence Distribution of Ventilation Measured by Electrical Impedance Tomography in Foals

#### 245.1.1 **Muriel Sacks** ^1^; Sharanne Raidal^2^, BVSc, MVSt, PhD, GradDipEd, FANZCVS; Giselle Hosgood^3^, BVSc (Hons), MS, PhD, FACVSc, DACVS (SAIM); Melanie Catanchin^4^, BA (Arch), BVetBiol, BVetSc (Hons 1), GradDipEd (Tertiary); Martina Mosing^1^, Dr. Magister, PD (Habil.), DECVAA, MANZCVS

##### 245.1.1.1 ^1^Murdoch University; ^2^Professor, Equine Medicine, Charles Sturt University Wagga Wagga; ^3^Professor, Small Animal Surgery, Murdoch University; ^4^Lecturer, Veterinary Anaesthesia, Charles Sturt University Wagga Wagga

**Background:** Interventions performed in the clinical management of foals include sedation and postural changes, such as lateral recumbency, which may affect ventilation.

**Objectives:** To assess the effects of sedation and changed body position on the distribution of ventilation by non‐invasive imaging using thoracic electrical impedance tomography (EIT).

**Animals:** Six healthy research Thoroughbred foals with a median age of 23 days (range 8 to 29 days).

**Methods:** In this prospective interventional pre‐ and post‐study EIT data was recorded in healthy foals using an electrode belt around the thorax. Impedance changes of ten individual breaths were averaged from standing foals pre‐sedation and ten minutes after diazepam sedation, and after ten minutes in lateral recumbency. Respiratory rate (RR), center of ventilation, dependent (DSS) and non‐dependent silent spaces were documented. Tidal volume was estimated from the total ventilation‐induced tidal impedance variation (TIV) in arbitrary units. Statistical analysis was performed using linear mixed effects model with significance determined at p<0.05.

**Results:** Sedation induced a significant reduction in RR (p=0.03) and increase in TIV (p=0.0002). Lateral recumbency induced a significant increase in DSS (p=0.0228) and shift of ventilation towards the non‐dependent lung (p=0.0001).

**Conclusion and Clinical Importance:** Diazepam sedation led to decreased respiratory rate, compensated by increased tidal volume in standing foals. Ten minutes of lateral recumbency caused decreased ventilation of the lower (dependent) lung, suggesting increased lung collapse. These findings are important in directing clinical management regarding sedation and positioning of foals during medical procedures.

## 246 Abstract E16

### 246.1 Clinical Findings and Outcome Predictors for Equine Multinodular Pulmonary Fibrosis: 46 Cases (2009–2019)

#### 246.1.1 **Amanda Craven** ^1^; Ignacio Corradini^2^, MV, MSc, ECEIM; Angelika Schoster^3^, Dr.med.vet, DVSc, DACVIM/ECEIM; Astrid Spierenburg^4^; Ilana Glasberg^5^; Rachel Liepman^6^, DVM, MS, DACVIM (LAIM); Daniela Luethy^7^, DVM, DACVIM (LAIM); Gillian Perkins^1^, DVM, DACVIM (LAIM); Pamela Wilkins^8^, DVM, MS, PhD, DACVIM (LAIM), DACVECC; David Wong^9^, DVM, MS, DACVIM (LAIM), DACVECC; Amy Todd‐Donato^1^, DVM; Tracy Stokol^1^, BVSc, PhD, DACVP; Joy Tomlinson^10^, DVM, DACVIM (LAIM)

##### 246.1.1.1 ^1^Department of Clinical Sciences, College of Veterinary Medicine, Cornell University, Ithaca, NY; ^2^Universidad Cardenal Herrera CEU; ^3^University of Zurich; ^4^Utrecht University; ^5^Comstock Equine Hospital; ^6^Chaparral Veterinary Medical Center; ^7^University of Florida; ^8^Department of Veterinary Clinical Medicine, College of Veterinary Medicine, University of Illinois; ^9^Veterinary Clinical Sciences, Iowa State University; ^10^Baker Institute for Animal Health, College of Veterinary Medicine, Cornell University

**Background:** Prognostic indicators for equine multinodular pulmonary fibrosis (EMPF), an interstitial fibrosing lung disease, are poorly described.

**Hypothesis/Objectives:** Describe clinical and diagnostic findings and determine outcome predictors in horses diagnosed with EMPF.

**Animals:** Forty‐six adult horses from academic and private referral institutions diagnosed with EMPF by the attending clinician. Cases were excluded if there was a diagnosis of primary bacterial or fungal pneumonia, or neoplasia.

**Methods:** Retrospective multicenter case series from 2009–2019. Medical records were reviewed. Convenience sample of radiograph and ultrasound imaging from 29 EMPF horses and bronchoalveolar lavage fluid (BALF) cytology from 6 EMPF and 13 asthma cases were independently reviewed, blinded to diagnosis and outcome. Associations between predictor variables and survival to discharge (short‐term) and to 3 months (long‐term) were assessed by predictor screening followed by Fisher's exact and Wilcoxon rank sum tests.

**Results:** Twenty‐seven horses (59%) and 11 (24%) survived to discharge and to 3 months, respectively. BALF macrophage atypia was seen in more EMPF than asthmatic horses (67% vs. 8%, p=0.017). Lower maximal temperature and band neutrophil counts, higher BALF lymphocyte:neutrophil ratios, and corticosteroid treatment were associated with short‐term survival. Lower respiratory rate, higher BALF lymphocyte:neutrophil ratios, and blood lymphocyte counts were associated with long‐term survival. Sonographic and radiographic findings were not associated with survival.

**Conclusions and clinical importance:** BALF macrophage atypia warrants additional testing for EMPF in horses with fever and weight loss. The prognosis for EMPF horses is poor and corticosteroid treatment does not improve long‐term survival.

**Table:** Table 1. Prognostic indicators for survival with EMPF. Predictor screening identified top predictors for short‐ and long‐term survival. Results confirmed by Fisher's exact test and Wilcoxon Rank Sums. Proportion or median (range) shown.

	Short‐term survival			Long‐term survival		
Predictor	Non‐survivors	Survivors	P‐value	Non‐survivors	Survivors	P‐value
Maximal temperature (°F)	103.3 (99.3–106.2)	102 (98.7–104.9)	0.022			
Band neutrophils (x10^9^ cells/L)	0 (0–1.3)	0 (0–0.3)	0.0037			
Steroid treatment	8/19, 42%	20/25, 80%	0.013			
BALF lymphocyte: neutrophil ratio	0.073 (0–0.47)	2.6 (0.14–38)	0.0011	0.47 (0–5.6)	4.72 (0.14–38)	0.013
Respiratory rate (brpm)				40 (12–64)	30 (16–50)	0.048
Blood lymphocyte count (x10^9^ cells/L)				0.9 (0.3–4.29)	1.25 (0.8–4.92)	0.02

## 247 Abstract E17

### 247.1 Developing a Tool to Detect and Track Laryngeal Dysfunction in Horses

#### 247.1.1 **Kile S. Townsend** ^1^; Ashley Kloepper^2^; Ali Hamad^3^; Filiz Bunyak^3^; Philip Johnson^4^, BVSc (Hons), MS, DACVIM, DECEIM; Teresa Lever^5^, PhD, CCC‐SLP

##### 247.1.1.1 ^1^University of Missouri; ^2^School of Medicine, University of Missouri; ^3^College of Electrical Engineering and Computer Science, University of Missouri; ^4^Professor of Veterinary Internal Medicine, Veterinary Medicine and Surgery, University of Missouri; ^5^Associate Professor, Otolaryngology‐Head and Neck Surgery, School of Medicine, University of Missouri

**Background:** Recurrent laryngeal neuropathy (RLN) is an idiopathic, progressive distal axonopathy that commonly leads to paresis/paralysis of the left side of the larynx in horses.

**Hypothesis/Objectives:** A prototype diagnostic tool was developed for objective evaluation of the **thoraco‐laryngeal adductor reflex** (TLAR). The objective was to establish normative TLAR parameters for healthy mature horses, paving the way for investigation of horses at high risk for RLN.

**Animals:** Twelve healthy adult horses (Quarter Horse and related breeds) were recruited from the university teaching herd.

**Materials and Methods:** TLAR responses were evoked via the ‘slap test’ during video‐recorded endoscopic examination of the larynx. Multiple TLARs per horse were quantified using our prototype ‘SlapTrack’ software that permits semi‐automated video tracking and objective quantification of bilateral laryngeal motion.

**Results:** The mean (standard error) TLAR duration was 213 (±5) ms in healthy adult horses, without left/right differences (p>0.05). Subcomponents of the TLAR had a stereotypical duration profile (p<0.05): glottic closure (4±1 ms) < laryngeal adduction (84±2 ms) < laryngeal abduction (126±5 ms). Glottic angle during the TLAR ranged from 41 (±4) to 14 (±2) degrees.

**Conclusions and Clinical Importance:** The TLAR can be objectively measured in horses and thus holds promise as a screening test and tool to monitor equine laryngeal dysfunction in conjunction with existing subjective grading scales. Ultimately, the goal is to develop an unbiased protocol to facilitate early detection, prediction, and long‐term monitoring of disease progression and treatment response in equine RLN.

## 248 Abstract E18

### 248.1 Seasonal Assessment of Serum 25‐Hydroxyvitamin‐D concentrations in Healthy and Asthmatic Horses

#### 248.1.1 Assaad Khoury^1^, DVM; **Daniela Bedenice** ^2^, DMV, DACVIM, DACVECC; Sarah Reed^3^, PhD; Lauren Bookbinder^4^, DVM, DACVIM; Melissa Larkin^5^; Victoria Trautwein^5^; Jillian Minuto^6^, DVM; Ana Pacheco^7^, DVM, DACVIM; Fiona Lehmann^8^, DVM; Melissa Mazan^9^, DVM, DACVIM

##### 248.1.1.1 ^1^Cumming School of Veterinary Medicine at Tufts University; ^2^Associate Professor, Clinical Sciences, Cumming School of Veterinary Medicine at Tufts University; ^3^Associate Professor, Department of Animal Science, University of Connecticut; ^4^Clinical Assistant Professor, Env & Pop Health‐Cummings‐Vet, Cummings School of Veterinary Medicine; ^5^Veterinary Student, Clinical Sciences, Cumming School of Veterinary Medicine at Tufts University; ^6^Resident, Clinical Sciences, Cumming School of Veterinary Medicine at Tufts University; ^7^Clinical Assistant Professor, Oregon State University; ^8^Intern, Loomis Basin Equine Medical Center; ^9^Professor, Clinical Sciences, Cumming School of Veterinary Medicine at Tufts University

**Background:** A small pilot study previously suggested that low serum 25‐hydroxyvitamin‐D concentrations may relate to equine asthma (EA) and airway neutrophilia. Similarly, human data proposes a strong association between vitamin‐D deficiency and bronchial asthma, a condition similar to EA.

**Hypothesis/Objectives:** The study goal was to evaluate the relationship between serum 25‐hydroxyvitamin‐D, diet, season, and the diagnosis of EA.

**Animals:** Ninety‐one adult horses from comparable stabling environments in New England.

**Methods:** Serum 25‐hydroxyvitamin‐D analysis, bronchoalveolar lavage fluid (BALF) cytology and non‐invasive pulmonary function testing (PFT: spirometry, forced oscillatory mechanics) were evaluated based on seasonal daylight duration, clinical exam, and horses’ management history. BALF slides were stained with modified Wright stain and Toluidine Blue. Groups and categories were compared using independent samples analyses according to data normality (SPSS‐26), with P<0.05 considered significant.

**Results:** A diagnosis of EA was established in 57/91 (62.6%) horses, as previously defined (2007‐ACVIM consensus), including 45/57 horses with airway neutrophilia >5% (median: 14.5%, inter‐quartile range, IQR‐34) and 31 with increased BALF mast cells >2% (median: 2.4%, IQR‐3.5). Asthmatic horses with ≥12 hours seasonal daylight exposure (intense‐light, IL season, n=34), showed significantly higher serum 25‐hydroxyvitamin‐D concentrations (23.4±8.5 mmol/L) than asthmatic horses (n=23) presented during the low‐light (LL) season (<12 hours daylight; 17.8±7.7 mmol/L). However, serum 25‐hydroxyvitamin‐D concentrations remained unrelated to BALF cytology, PFT or the diagnosis of EA.

**Conclusions and Clinical Importance:** This study does not support an association between serum 25‐hydroxyvitamin‐D concentrations and EA, when considering seasonal variation (IL versus LL) and horse management.

## 249 Abstract E19

### 249.1 Transthoracic Echocardiographic Parameters in Critically Ill Newborn Foals: Comparison with Healthy Foals

#### 249.1.1 **Francesca Freccero** ^1^; Giovanni Romito^2^, DVM, PhD, DECVIM‐CA (Cardiology); Carolina Beato^3^; Aliai Lanci^4^, DVM, PhD; Jole Mariella^2^, DVM, PhD; Nicola Ellero^5^, DVM; Mario Cipone^6^, DVM; Carolina Castagnetti^6^, DVM, PhD, DECAR

##### 249.1.1.1 ^1^University of Bologna; ^2^Assistant Professor, University of Bologna; ^3^Private Practitioner; ^4^Assistant Professor (Junior), University of Bologna; ^5^PhD Student, University of Bologna; ^6^Full Professor, University of Bologna

**Background:** No data is available on transthoracic echocardiography in critically ill newborn foals (CINF).

**Hypothesis/Objectives:** To describe transthoracic echocardiographic parameters in CINF, and compare them with parameters obtained in healthy foals.

**Animals:** Thirty‐three foals less than three days‐old prospectively enrolled during three foaling seasons: 10 CINF suffering of various neonatal diseases (i.e., sepsis, neonatal syndrome, prematurity) and 23 healthy foals born from mares hospitalized for attended parturition.

**Methods:** Prospective observational study. In all foals, a complete (M‐mode, two‐dimensional and Doppler) transthoracic echocardiography was obtained from a right parasternal and subcostal view without sedation. Conventional techniques and formulas were applied for the estimation of left ventricular (LV) volumes, fractional shortening, ejection fraction, stroke volume and cardiac output. CINF underwent echocardiography before receiving any treatment. Echocardiographic variables were compared between groups using a Mann‐Whitney U test. A p‐value <0.05 was considered statistically significant.

**Results:** In all foals, 40 echocardiographic variables were analyzed (13 M‐Mode, 21 two‐dimensional and 6 pulsed‐wave Doppler). Several statistically significant differences were found between groups: CINF had reduced LV diameters and volumes, left atrium and stroke volume as well as increased heart rate and LV wall thicknesses, fractional shortening and ejection fraction. Conversely, no relevant differences were found for cardiac output irrespective of the methods of measurement used (Tables 1–2).

**Table:** Table 1. Selected M‐Mode echocardiographic variables in healthy and critically ill newborn foals

Parameter	Healthy	CINF	p‐value
IVSd (cm)	1.5	1.7	0.0267
LVIDd (cm)	5.4	3.8	0.0022
LVFWd (cm)	0.9	1.0	0.0075
IVSs (cm)	2.2	2.5	0.0131
LVIDs (cm)	3.5	2.0	0.0006
LVFWs (cm)	1.5	2.0	0.0029
EDV (mL)	138.9	63.0	0.0022
ESV (mL)	50.8	13.1	0.0006
FS (%)	36.4	44.2	0.0015
EF (%)	66.0	76.0	0.0015
HR (bpm)	88	125	0.0065
SV (mL)	90.7	56.6	0.0087
CO (L/min)	8.1	6.9	0.1959

As all data were not normally distributed, they were presented as median values. In bold values with statistical differences. Bpm: beats per minute; CINF: critically ill newborn foals; CO: cardiac output; EDV: left ventricular end‐diastolic volume; EF: ejection fraction; ESV: left ventricular end‐systolic volume; FS: fractional shortening; HR: heart rate; IVSd: interventricular septum thickness at end‐diastole; IVSs: interventricular septum thickness at end‐systole; LVIDd: left ventricular internal dimension at end‐diastole; LVIDs: left ventricular internal dimension at end‐systole; LVPWd: left ventricular posterior wall thickness at end‐diastole; LVPWs: left ventricular posterior wall thickness at end‐systole; SV: stroke volume.

**Table:** Table 2. Selected two‐dimensional and pulsed‐wave Doppler echocardiographic variables in healthy and critically ill newborn foals

Parameter	Healthy	CINF	p‐value
Two‐dimensional			
LAD_La_ (cm)	5.7	5.0	0.0267
LA/Ao_Sa_	1.5	1.3	0.0073
EDV_S_ (mL)	145.7	77.4	0.0004
ESV_S_ (mL)	54.7	15.2	0.0005
SV_S_ (mL)	86.9	62.5	0.0011
CO_S_ (L/min)	8.0	7.9	0.8293
EDV_B_ (mL)	147.1	93.1	0.0005
ESV_B_ (mL)	47.3	9.1	0.0007
SV_B_ (mL)	109.9	84.0	0.0016
CO_B_ (L/min)	9.3	8.6	0.24
Pulsed‐wave Doppler			
SV_Ao_ (mL)	151.9	103.7	0.00817
CO_Ao_ (L/min)	14.4	10.0	0.4862

As all data were not normally distributed, they were presented as median values. In bold values with statistical differences. CINF: critically ill newborn foals; CO_Ao_, CO_B_ and CO_S_: cardiac output assessed by the aortic‐flow based method, bullet method and single plane Simpson's method of discs, respectively; EDV_B_ and EDV_S_: left ventricular end‐diastolic volume assessed by the bullet method and single plane Simpson's method of discs, respectively; ESV_B_ and ESV_S_: left ventricular end‐systolic volume assessed by the bullet method and single plane Simpson's method of discs, respectively; LA/Ao_Sa_: left atrial‐to‐aortic ratios obtained from a right parasternal short axis view; LAD_La_: maximal left atrial diameter obtained from a right parasternal long axis view; SV_Ao_, SV_B_ and SV_S_: stroke volume assessed by the aortic‐flow based method, bullet method and single plane Simpson's method of discs, respectively.

**Conclusions and Clinical Importance:** Echocardiographic signs consistent with volume depletion, pseudohypertrophy and sympathetic response aimed at maintaining a normal cardiac output occur in CINF. In CINF, transthoracic echocardiography may improve understanding of pathophysiology and guide treatment.

## 250 Abstract E20

### 250.1 Systematic Review and Meta‐Analysis of Risk Factors for Conversion and Recurrence of Atrial Fibrillation

#### 250.1.1 **Antoine Premont** ^1^; Louise Lafin^2^, DVM; Rebecca Lewis^3^, BSc (Hons), PhD, FHEA; Celia Marr^4^, BVMS, MVM, DEIM, PhD, DECEIM, FRCVS; Kamalan Jeevaratnam^5^, DVM, MRCVS, PhD

##### 250.1.1.1 ^1^University of Surrey; ^2^Equine Veterinary Clinic of Meheudin; ^3^Lecturer in Physiology, Pre‐clinical Sciences, University of Surrey; ^4^Veterinary Surgeon, Rossdales Equine Hospital; ^5^Head of Department, Pre‐clinical Sciences, University of Surrey

**Background:** Both electrical and pharmacological methods for atrial fibrillation (AF) treatment are widely used. Determination of reliable prognostic factors for success and recurrence is essential to improve the therapeutic decision process.

**Objective:** The objective was to evaluate the impact of risk factors on the cardioversion success and recurrence rates of all the current AF treatments.

**Method:** After a systematic search in three international databases, studies reporting treatment of spontaneous AF in at least two horses were included and critically evaluated. A qualitative and quantitative analysis were performed.

**Results:** Twenty‐three studies met the inclusion criteria for the analysis of the success rate and 47 for cardioversion success. The success rate estimation was 86.8% ([78.5;93.6]) for oral quinidine treatment and 96.8% ([89.1;100]) for electrical cardioversion. Horses successfully treated with quinidine were affected for a significantly shorter period than those that failed (mean difference 240, [62; 417], p‐value=0.008). Recurrence was also associated with a longer AF duration. A significant effect of cardiac disease on cardioversion success could not be demonstrated (p‐value=0.2), however, some structural and functional echocardiographic parameters were reported to influence recurrence risk (left atrial area and fractional area change). Electrical remodelling demonstrated by higher fibrillation rate was associated with both lower success and higher recurrence. No clear effect of height, weight, or age could be demonstrated.

**Conclusion:** Both methods for cardioversion are highly efficient but recurrence is frequent. Duration of arrhythmia, structural and electrical remodeling parameters are the most influential factors on AF conversion success and recurrence.

## 251 Abstract E21

### 251.1 Effect of a Single Intravenous Dose of Sirolimus on Insulin Dynamics in Healthy Adult Horses

#### 251.1.1 **Demia J. de Tonnerre** ^1^; Carlos Medina Torres^2^, DVM, MSc, DVSc, PhD, DACVIM, DECEIM, MANZCVS; Darko Stefanovki^3^, BS, MS, PhD; Mary Robinson^4^, VMD, PhD, DACVCP; Kate Kemp^5^, BSc, BEquineSc (Hons); François‐René Bertin^6^, DVM, MS, PhD, DACVIM (LAIM); Andrew van Eps^7^, BVSc, PhD, DACVIM (LAIM)

##### 251.1.1.1 ^1^The University of Queensland; ^2^Head of Internal Medicine, Pferdeklinik SaarLorLux GmbH; ^3^Associate Professor of Biostatistics, School of Veterinary Medicine, University of Pennsylvania; ^4^Assistant Professor of Veterinary Pharmacology, School of Veterinary Medicine, University of Pennsylvania; ^5^PhD Candidate, School of Veterinary Science, The University of Queensland; ^6^Associate Professor in Equine Internal Medicine, School of Veterinary Science, The University of Queensland; ^7^Associate Professor of Large Animal Musculoskeletal Research, School of Veterinary Medicine, University of Pennsylvania

**Background:** Sirolimus, a mechanistic target of rapamycin (mTOR) inhibitor, can suppress insulin production and has therapeutic potential for insulin dysregulated (ID) horses.

**Hypothesis/Objective:** Determine the pharmacokinetics of intravenous sirolimus in healthy adult horses and evaluate its effect on insulin dynamics.

**Animals:** 8 healthy adult Standardbred geldings.

**Methods:** A pharmacokinetic study was performed followed by a placebo‐controlled, randomized, crossover study. Serial blood sirolimus concentrations were measured by liquid chromatography/mass spectrometry for 72 h after an intravenous dose (0.06 mg/kg). PK indices were estimated by fitting a 2‐compartment model using non‐linear least squares regression. Next, an oral glucose test was conducted before and 4, 24, 72 and 144 h after administration of a single dose of sirolimus or placebo (propylene glycol). The effects of time, treatment and animal on blood glucose and insulin concentrations were analyzed using mixed‐effects linear regression.

**Results:** No adverse effects were observed. Median (range) maximum sirolimus concentration was 277.0 (247.5–316.06) ng/mL at 5 (5–10) minutes and half‐life was 3552 (3248–4767) minutes. Sirolimus had a significant effect on insulin concentration 24 h after dosing only: median (interquartile range) insulin concentration at 60 min (5.0 [3.7–7.0] μIU/mL) was 37% (−5–54%) less than placebo (8.7 [5.8–13.7] μIU/mL, p=0.03); and at 120 min (10.2 [8.4–12.2] μIU/mL) was 28% (−15–53%) less than placebo (14.9 [8.4–24.8] μIU/mL, p=0.02). There was no effect on blood glucose.

**Conclusion and Clinical Importance:** Sirolimus was well tolerated and decreased the insulinemic response to glucose challenge in healthy horses. Sirolimus warrants investigation for control of hyperinsulinemia in ID horses.

## 252 Abstract E22

### 252.1 Fluorescent Immunoassay‐Established Reference Intervals and Circannual Variation of Equine Plasma Adrenocorticotropic Hormone Concentrations in Qatar

#### 252.1.1 **Camilla A. Jamieson** ^1^; Daniela Amado^2^, DVM; Sarah Baillie^3^, BSc (Hons), REVN, DHE, CVN, DAVN; Marcello Conte^4^, DVM; Janinne Manuel^5^, CMLS; Mohammad Ali^6^, DVM, MSc, PhD; Ian Thompson^7^, PhD

##### 252.1.1.1 ^1^Hagyard Equine Medical Institute; ^2^Intern, Internal Medicine, EVMC; ^3^Head Medicine Nurse, Internal Medicine, EVMC; ^4^Senior Veterinarian, Al Shaqab Equine Education; ^5^Lab Technician, Diagnostic Laboratory, EVMC; ^6^Diagnostic Laboratory Manager, Diagnostic Laboratory, EVMC; ^7^Bioinformatics Specialist, Qatar Biomedical Research Institute, Hamad Bin Khalifa University

**Background:** Measuring endogenous plasma adrenocorticotropic hormone (ACTH) is the most common diagnostic test for equine pituitary pars intermedia dysfunction. Accurate diagnosis depends on understanding normal values and ACTH secretion patterns.

Published reference intervals for ACTH are based on costly chemiluminescent assays. An affordable fluorescent immunoassay has recently been adapted for the equine sector. Studies have shown acceptable intra‐and‐inter‐run variation with poor value agreement between analysis methodology.

Additionally, circannual variation with a physiological increase in plasma ACTH as the daily photoperiod shortens has been demonstrated in both the northern and southern hemispheres. This has not been evaluated in the Gulf region, where the circannual photoperiod variability is attenuated compared to other locations

**Objectives:** To establish fluorescent‐immunoassay ACTH reference ranges and evaluate circannual variability

**Animals:** 60 healthy adult riding‐school horses, sampled once; 15 additional, sampled monthly for twelve months

**Methods:** Potassium‐EDTA anticoagulated plasma was analyzed via AIA‐360 analyzer, and reference intervals were established by determining normality, then the 2.5^th^–97.5^th^ percentile of the dataset. Seasonal variation was assessed with Benjamini‐Hochberg corrected pairwise t‐tests, to compare the means of monthly datasets.

**Results:** Reference interval established: **10.83–32.66 pg/ml**.

All monthly means and standard deviations lay within the established reference intervals; however, significant differences between individual monthly means were identified.

**Conclusions:** This is the first reported reference interval for AIA‐360 analyzer‐generated plasma ACTH in horses. The diminished circannual photoperiod variability in the Gulf appears to attenuate the seasonal variability of ACTH secretion, eliminating the need for seasonally adjusted reference intervals.

## 253 Abstract E23

### 253.1 Equine Hyperinsulinemia Causes Tissue‐Specific Alterations of Cytokines and Acute Phase Proteins in a NFκB‐Independent Manner

#### 253.1.1 **Weerasekara M.N.K. Jayathilake** ^1^; Risco Carlos^2^; Martin Furr^3^; Veronique Lacombe^4^; Melody de Laat^5^

##### 253.1.1.1 ^1^Oklahoma State University; ^2^Dean, Veterinary Medicine, Oklahoma State University; ^3^Head of the Department, Veterinary Physiology, Oklahoma State University; ^4^Professor, Veterinary Physiology, Oklahoma State University; ^5^Queensland University of Technology

Equine metabolic syndrome (EMS) causes hyperinsulinemia leading to debilitating sequela including laminitis.

Despite the increased clinical recognition, the pathophysiology underlying EMS and laminitis is not elucidated. Whether EMS horses develop cardiovascular complication is also not well established. Therefore, we hypothesized that hyperinsulinemia induces an increased expression of inflammatory proteins in a tissue‐specific manner.

Cardiac and skeletal muscle and lamellae biopsies were previously collected from horses following a 48‐hour prolonged euglycemic‐hyperinsulinemic clamp, and from electrolyte‐treated control horses.

Protein expression was quantified via western blotting. All hyperinsulinemic horses developed laminitis despite being previously healthy. Protein expression of HSP90, alpha 2 macroglobulin (A2M), fibrinogen isoforms was upregulated in lamellae of hyperinsulinemic horses, as well as inflammatory cytokines including interleukin‐1, interleukin 6 (IL‐6) and tumor necrosis factor. In contrast, protein expression of inflammatory cytokines and acute phase proteins was unchanged in cardiac and skeletal muscle muscles of hyperinsulinemic horses compared to their control counterparts. Total, phospho‐NFκB protein expression and the ratio phospho/total NFκB were not changed in the lamellae, heart, or skeletal muscles of hyperinsulinemic horses compared to controls.

In conclusion, upregulation of inflammatory cytokines and acute phase proteins in lamellae during equine hyperinsulinemia‐induced laminitis, which occur in a NF‐κB independent manner, may reveal novel biomarkers and potential therapeutic targets for EMS. Further, the lack of increase of inflammatory proteins and cytokines in the heart following hyperinsulinemia may underscore potential cardioprotective mechanisms in this species.

## 254 FOOD ANIMAL INTERNAL MEDICINE

## 255 Abstract F01

### 255.1 Role of Nasal BRSV‐IgG1 Titers on Clinical Protection of Calves Against Experimental Challenge with BRSV

#### 255.1.1 **David A. Martinez** ^1^; Manuel Chamorro^2^, DVM, MS, PhD, DACVIM (LAIM); Amelia Woolums^3^, DVM, MS, PhD, DACVIM (LAIM), DACVM; Thomas Passler^4^, DVM, PHD, DACVIM (LAIM); Gage Raithel^5^, BS; Scott Silvis^6^, BS, MS; Merrilee Thoresen^7^, PhD; Paul Walz^8^, DVM, MS, PhD, DACVIM (LAIM)

##### 255.1.1.1 ^1^College of Veterinary Medicine, Auburn University; ^2^Associate Professor, Clinical Sciences, College of Veterinary Medicine, Auburn University; ^3^Professor, Pathobiology and Population Medicine, College of Veterinary Medicine, Mississippi State University; ^4^Professor, Clinical Sciences, College of Veterinary Medicine, Auburn University; ^5^Technician, Pathobiology, College of Veterinary Medicine, Auburn University; ^6^Lead Technician, Pathobiology, College of Veterinary Medicine, Auburn University; ^7^Laboratory Manager, Pathobiology and Population Medicine, College of Veterinary Medicine, Mississippi State University; ^8^Professor and Head, Pathobiology, College of Veterinary Medicine, Auburn University

**Background:** The role of nasal BRSV IgG1 titers transferred from colostrum on clinical protection against BRSV infection in calves is unknown.

**Hypothesis/Objectives:** To determine if greater titers of nasal BRSV IgG1 transferred from colostrum protected calves against BRSV challenge.

**Animals:** Forty, 3‐month‐old beef steers.

**Methods:** Randomized, controlled, clinical trial. Forty calves were assigned to 1 of 2 treatment groups. Group Vacc (n=20) nursed colostrum from cows vaccinated with 2 doses of an inactivated‐BRSV vaccine before calving. Group NoVacc (n=20) nursed colostrum from unvaccinated cows. At 3 months of age, calves were challenged with BRSV by nebulization. Following challenge, respiratory signs were scored. Nasal secretion and serum samples were collected before and after challenge for nasal IgG1 and neutralizing antibody testing. Nasal secretion samples were collected after challenge for identification of BRSV by RT‐PCR.

**Results:** Following challenge, mild respiratory scores were recorded in both groups (P>0.05). The proportion of calves with fever was greater in NoVacc calves. Nasal BRSV IgG1 titers were greater in Vacc calves at 48 hours of life (P<0.05); however, decayed to undetectable levels in both groups before challenge (P>0.05). Serum BRSV antibodies were greater in Vacc calves at 48 hours and on challenge day. A greater proportion of NoVacc calves tested positive by BRSV RT‐PCR after challenge (P<0.05).

**Conclusions and Clinical Importance:** Nasal BRSV IgG‐1 from colostrum does not play a role in clinical protection of 3‐month‐old calves experimentally challenged with BRSV.

## 256 Abstract F02

### 256.1 Safety and Immunogenicity of Two Bovine Coronavirus Vaccines in Goats

#### 256.1.1 **Meera Heller** ^1^; Maggie Buktenica^1^; Fauna Smith^2^, DVM, DACVIM; Rosie Busch^3^, DVM; Maia Laabs^1^; Joan Rowe^4^, DVM, MPVM, PhD

##### 256.1.1.1 ^1^University of California, Davis; ^2^Center for Comparative Medicine, University of California, Davis; ^3^Assistant Professor, Cooperative Extension, Population Health and Reproduction, University of California, Davis; ^4^Professor Emeritus, Population Health and Reproduction, University of California, Davis

**Background:** A novel coronavirus, closely related to bovine/bovine‐like coronaviruses, was identified in 2017, associated with an outbreak of gastrointestinal disease in goats. A bovine coronavirus ELISA adapted to detect anti‐bovine coronavirus antibodies in goats has shown coronavirus is an important pathogen in California goat populations.

**Objectives:** To determine the safety and immunogenicity of bovine coronavirus vaccines, a modified live intranasal (MLV) and an intramuscular killed (KV) in goats.

**Animals:** Twenty 18‐month‐old seronegative does randomized into two groups: MLV and KV, 7 vaccinates and 3 placebo controls per group.

**Methods:** Vaccines were administered per manufacturer's instructions for cattle; a single intranasal dose for the MLV and a 2‐dose series for the KV. Serum samples, nasal and rectal swabs, injection site monitoring and physical exams were done regularly. PCR for coronavirus was done on swabs. ELISA was performed on serum, results analyzed using a 2‐way ANOVA.

**Results:** Coronavirus PCR: One positive nasal swab 2 days post vaccination in an MLV vaccinate, all other swabs were PCR negative. ELISA: All KV vaccinates seroconverted, none of the MLV or control animals did. Titers in KV vaccinates were significantly higher than controls at booster (p=0.029), increased following booster, with peak titers occurring 2 weeks post‐booster (p<0.0001). No reactions were seen post MLV or first dose KV dose; second KV dose resulted in local reactions in 5/7 goats.

**Conclusions and Clinical Importance:** While seroconversion occurred with the KV, clinical efficacy of both vaccines remains unknown; however, both seem to be safe to use in goats.

## 257 Abstract F03

### 257.1 Blood Cultures and Outcomes in Sick Neonatal Beef Calves

#### 257.1.1 Luis A. Rivero^1^; Pamela Adkins^2^, DVM, PhD, DACVIM

##### 257.1.1.1 ^1^University of Missouri; ^2^Assistant Professor, University of Missouri

**Background:** Bacteremia leading to sepsis is a life‐threatening condition in neonatal beef calves.

**Hypothesis:** Blood culture‐positive calves are less likely to survive to discharge compared to blood culture‐negative calves.

**Animals:** Beef calves presented for illness were eligible for enrollment. Inclusion criteria included being a beef calf that was less than 21 days old, an identifiable focal site of infection, and client consent.

**Methods:** Three aseptic venous blood samples were collected sequentially from 3 different sites from each enrolled calf. Samples were aerobically and anaerobically cultured. Bacteria were identified and susceptibility profiles were performed. A calf was defined as bacteremic if 2/3 cultures were positive with the same bacterial species. Follow‐up survival data was collected through client communications at 2‐weeks and 3‐months post discharge.

**Results:** To date, 19 calves have been enrolled. Median age was 8 days (range 1–21), including 7 male and 12 female calves. Breeds included 10 Angus, 4 Mixed, 3 Gelbvieh, 1 Charolais, and 1 Brangus. Eighteen calves had 1 focal site of infection and 1 calf had two. Focal sites of infection included gastrointestinal (13/20), joint (2/20), umbilicus (2/20), ocular (2/20) and respiratory (1/20). Blood cultures were positive in 4 (21%) calves. *Escherichia coli* was identified in 3 calves and *Bacteroides uniformis* in 1. None of the bacteremic calves survived to discharge while 10/15 (67%) non‐bacteremic calves survived until 3 months after discharge.

**Clinical Importance:** Bacteremia is associated with non‐survival and utilizing the three‐culture technique led to a decreased frequency of bacteremia than previously reported.

## 258 Abstract F04

### 258.1 Pathogen‐Specific Intramammary Infection Prevalence, Persistence, and Somatic Cell Count Association in Lactating Jersey Cows

#### 258.1.1 **Samantha Haw** ^1^; Pamela Adkins^2^, DVM, PhD, DACVIM; Veronique Gosselin^3^, DVM, MSc, PhD, DACVIM; John Middleton^4^, DVM, PhD, DACVIM

##### 258.1.1.1 ^1^University of Missouri; ^2^Assistant Professor, Food Animal Medicine and Surgery, University of Missouri; ^3^Assistant Professor, Clinic for Ruminants, University of Bern; ^4^Professor, Food Animal Medicine and Surgery, University of Missouri

**Background:** There is limited data available regarding pathogen‐specific intramammary infection (IMI) in Jersey cows.

**Objectives:** The objectives were to characterize the prevalence and persistence of bacterial IMI in lactating Jersey cattle, evaluate pathogen associations with somatic cell count (SCC), and determine if first sampling SCC could be predictive of IMI persistence.

**Animals:** Lactating cows (n=753) were enrolled from four Jersey dairies in the lower Midwest USA.

**Methods:** This observational longitudinal study was conducted using aseptically collected mammary quarter foremilk samples, collected once monthly for three consecutive months. Pathogens were identified using aerobic milk culture and matrix‐assisted laser desorption/ionization‐time‐of‐flight mass spectrometry. A commercial laboratory measured SCC using flow cytometry. Results were defined as IMI (genus, species or unidentified pathogen), mixed infection, contaminated, and persistent or non‐persistent infection.

**Results:** *Staphylococcus chromogenes* and *Staphylococcus simulans* were the most commonly isolated pathogens among the 7,370 quarter level samples. Some pathogen IMIs were associated with SCC and days in milk (P≤0.01). *Staphylococcus chromogenes*, *S. simulans*, *S. aureus,* and *Streptococcus uberis* were the most common species that caused persistent IMI. Mixed logistic regression followed by pairwise comparison showed no detectable differences in odds of persistence. First sampling SCC was not predictive of an IMI persisting beyond first sampling.

**Conclusions and Clinical Importance:** Non‐*aureus* staphylococci were the most prevalent organisms identified. Pathogen type and initial SCC were not predictive of IMI persistence. Evaluating associations among commonly identified pathogens and SCC helped define how prevalent pathogens may affect milk quality in Jersey cows.

## 259 Abstract F05

### 259.1 Left Ventricular Systolic Function in Neonatal Calves with Diarrhea

#### 259.1.1 **Osman Safa Terzi** ^1^; Yasin Şenel^2^; Erdal Kara^2^; Hasan Albasan^3^

##### 259.1.1.1 ^1^Ankara University; ^2^Kirikkale Uni.; ^3^Pet Depot Veterinary Hospital

**Background:** Cardiac muscle damages in newborn calves with diarrhea due to hyperkalemic changes has been reported by many researchers.

**Objectives:** The aim of this study was to investigate whether the systolic function of the left ventricle was affected in neonatal calves with diarrhea.

**Animals:** Study materials were consisted of 27 Holstein calves; 12 (8‐male and 4‐female) were healthy (mean age±std; 18±10.85 days) and 15 (9‐male and 6‐female) were with diarrhea (mean age±std; 12.60±3.73 days). All calves survived.

**Methods:** Radial strain (Rst), circumferential strain (Cst) and M mode echocardiography data at the short axis papillary muscle level were recorded for offline analysis. Speckle tracking echocardiography data were obtained from the Mid‐anteroseptal (MAS), Mid‐anterior (MA), Mid‐anterolateral (MAL), Mid‐inferolateral (MIL), Mid‐inferior (MI), and Mid‐inferoseptal (MIS) regions at the short axis papillary muscle level.

**Results:** Independent t‐test was applied. When values of ESV (p=0.3080), EDV (p=0.1789), SV (0.5024) and EF (p=0.0833) from M mode data was compared, there was no statistically significant difference. Moreover, when values of MAS (Rst p=0.0700, Cst p=0.0251), MA (Rst p=0.0470, Cst p=0.0342), MAL (Rst p=0.0990, Cst p=0.0546), MIL (Rst p=0.0998, Cst p=0.2546), MI (Rst p=0.0145, Cst p=0.0161) and MIS (Rst p=0.5997, Cst p=0.0246) from the radial and circumferential stress data of the regions compared, there was no statistically significant difference.

**Conclusions and Clinical Importance:** Results of present study indicated that neonatal diarrhea did not have any effects on the contractile functions of the left ventricle in calves.

## 260 Abstract F06

### 260.1 The Association Between Fecal Microbiota, Endoparasitism and Age of Adult Alpacas

#### 260.1.1 **Daniela Bedenice** ^1^; Jessica Resnick^2^, MS; Lauren Bookbinder^3^, DVM, DACVIM; Victoria Trautwein^4^; Hannah Creasey^5^; Giovanni Widmer^6^, PhD

##### 260.1.1.1 ^1^Cumming School of Veterinary Medicine at Tufts University; ^2^Infectious Disease & Global Health, Cumming School of Veterinary Medicine at Tufts University; ^3^Clinical Assistant Professor, Env & Pop Health‐Cummings‐Vet, Cummings School of Veterinary Medicine; ^4^Veterinary Student, Cumming School of Veterinary Medicine at Tufts University; ^5^Senior Research Technician, Infectious Disease & Global Health, Cumming School of Veterinary Medicine at Tufts University; ^6^Professor, Infectious Disease & Global Health, Cummings School of Veterinary Medicine

**Background:** Endoparasitism causes substantial morbidity and mortality in alpacas, with growing emergence of anthelmintic resistance.

**Hypothesis/Objectives:** The study purpose was to evaluate the effect of nematode infection and host phenotype on the intestinal microbiota of adult alpacas.

**Animals:** 102 healthy adult (2.1–11.2 years), client‐owned alpacas at a single farm, managed under identical conditions.

**Methods:** Fecal samples were collected per‐rectum from alpacas at 3 separate timepoints (pre‐ and post‐treatment with 8.8 mg/kg oral levamisole, and 4.6 months later), and analyzed using 16S amplicon sequencing. Serial clinical exams and fecal egg counts (FEC) were compared using related‐samples analyses (SPSS‐26).

**Results:** The microbiota in different alpacas was relatively similar. Alpha (Shannon)‐diversity was significantly correlated between timepoints, where animals harboring a more diverse microbiota maintained these throughout the study period. Pairwise β‐diversity between samples was low, ranging from 0.16–0.21 UniFrac distance units, and was significantly correlated between collections, indicating microbiota stability over time. *Bacteroidales* was the most abundant order at all timepoints. The intensity of HOT‐complex nematode infection was only a significant predictor of microbiota composition in samples collected 14 days after levamisole treatment. Among the four clinical predictors tested (age, weight, HOT FEC, persistent HOT‐shedder status), age explained the largest proportion (but <10%) of fecal microbiota variation.

**Conclusions and Clinical Importance:** The fecal microbiota of identically managed, healthy alpacas was characterized by a high level of temporal stability, as both α and β‐diversity tended to persist across sampling timepoints. Alpaca age was the only consistently significant predictor of fecal microbiota taxonomic composition.

## 261 Abstract F07

### 261.1 Minimum Colostral Immunoglobulin G Concentration Required for Pooling to Achieve Adequate Immunity in Dairy Calves

#### 261.1.1 **Ailbhe King** ^1^; Hilari French^1^; Katherine Bandlow^2^; Felix Toka^1^; Munashe Chigerwe^2^

##### 261.1.1.1 ^1^Ross University School of Veterinary Medicine; ^2^UC Davis

**Background:** Despite known colostrum feeding recommendations, 10–21% of calves suffer from failure of transfer of passive immunity in the US. Pooling colostrum occurs commonly on dairies but practice standards for colostrum quality when pooling are poorly defined.

**Hypotheses:** Predicted mean pool Immunoglobulin G (IgG) will equal the measured pool IgG

9 Minimum required IgG for pooling is 62–65 g IgG

**Animals:** 202 Jersey cows.

**Methods:** Prospective cohort study with 27 colostrum pools composed of 4–10 cows per pool. Parity, total number of cows contributing to a pool, individual cow colostral volume, and total pool volume were recorded. IgG concentrations in pre and post‐heat treatment individual and pooled colostrum was measured by radial immunodiffusion. Variables predicting adequate colostral pool IgG (200 g) were determined by mixed model regression analysis.

**Results:** Post‐heat treatment pooled colostral IgG concentrations were not different than the predicted IgG from individual cow colostral IgG concentrations (P=0.465). The minimum pool colostral IgG required to ensure adequate transfer assuming 4L of colostrum fed was 68–70 g/L. Number of cows contributing to the pool (P<0.0001*) and total pool volume (P<0.0011) were significant predictors of pool IgG. Colostrum pools contributed by ≤7 cows or pool volumes of ≤40 L were associated with adequate colostral IgG.

**Conclusions and Clinical Importance:** A minimum pool IgG of 68–70 g/L is required to achieve adequate immunity in dairy calves. To ensure sufficient colostral IgG concentrations, pools from ≤7 cows or pool volumes of ≤40L are recommended.

## 262 Abstract F08

### 262.1 Effect of Tulathromycin Metaphylaxis on *Mannheimia haemolytica* Isolation and Health Outcomes in Stocker Heifers

#### 262.1.1 **William B. Crosby** ^1^; Alexandra Pittman^2^; Brandi Karisch^3^, PhD; John Loy^4^, DVM, PhD, ACVM; William Epperson^5^, DVM, DACVPM (Epidemiology); Sarah Capik^6^, DVM, PhD; Paul Morley^7^, DVM, PhD, DACVIM (LAIM); Jonathan Frye^8^, PhD; Charlene Jackson^9^, PhD; Amelia Woolums^10^, DVM, PhD, DACVIM (LAIM)

##### 262.1.1.1 ^1^College of Veterinary Medicine, Mississippi State University; ^2^Lecturer, Department of Animal and Dairy Science, Mississippi State University; ^3^Associate Professor (Research/Extension), Department of Animal and Dairy Science, Mississippi State University; ^4^Associate Professor, Nebraska Veterinary Diagnostic Laboratory, University of Nebraska, Lincoln; ^5^Professor and Department Head, Pathobiology and Population Medicine, Mississippi State University; ^6^Assistant Professor, Department of Veterinary Pathobiology, Texas A&M University and AgriLife Research; ^7^Professor and Director of Research, VERO Center, Texas A&M University; ^8^Research Microbiologist, Bacterial Epidemiology and Antimicrobial Resistance Research, USDA‐ARS; ^9^Research Microbiologist, Bacterial Epidemiology and Antimicrobial Resistance Research, USDA‐ARS; ^10^Professor, Pathobiology and Population Medicine, Mississippi State University

**Background:** Bovine respiratory disease (BRD) is commonly controlled by metaphylaxis, but increasing prevalence of antimicrobial resistant (AMR) *Mannheimia haemolytica* (MH) may decrease efficacy.

**Hypothesis/Objectives:** Determine the effect of macrolide metaphylaxis on 1) morbidity and mortality in stocker cattle over a 10‐week period and 2) nasopharyngeal isolation of MH, and MH genotypes.

**Animals:** Commercial beef cross heifers (n=335, 232±17.8 kg) purchased from regional auction markets for 4 trials from October 2019 to October 2021 (3 Fall and 1 Spring).

**Methods:** Cattle were randomized to receive tulathromycin at 2.5 mg/kg SC (META, n=168) or not (NO META, n=167). Nasopharyngeal swabs (NPS) were obtained at arrival and 3 and 10 weeks later for culture, identification, and MH genotyping. Groups were separated with no contact; any calves requiring additional antimicrobial treatment were moved into separate pastures. After the 3‐week sampling, calves were commingled.

**Results:** Over all trials, total and BRD morbidity was significantly lower in META animals than NO META (Table 1, Chi‐square, P=0.002); however, difference in BRD morbidity was due only to the Spring and Fall 2021 trials (Chi‐square, P=0.002 and P=0.037, respectively). There was no difference in mortality, Week 3 and Week 10 MH isolation rate, or the genotypes isolated between treatment groups (Tables 1 and 2).

**Table:** Table 1

Trial	Animals	Arrival	Mh Culture	Arrival Weight—SD (kg)	Animals Treated—Total (%)	Animals Treated—BRD (%)	Mortality—Overall (%)
Fall 2019	84	15.5%	229.5±18.3	28.6	22.6	0.0	0.0
Meta	42	26.2%	227.7±18.6	28.6	21.4	0.0	0.0
No meta	42	4.8%	231.4±18.0	28.6	23.8	0.0	0.0
Fall 2020	84	20.2%	229.1±16.3	26.2	17.9	4.8	4.8
Meta	42	19.0%	228.6±15.9	21.4	16.7	4.8	4.8
No meta	42	21.4%	230.0±17.0	31.0	19.0	4.8	4.8
Spring 2021	83	8.4%	231.4±18.9	27.7*	25.0*	15.7	12.0
Meta	42	4.8%	231.4±18.5	11.9*	9.5*	9.5	4.8
No meta	41	11.9%	231.4±19.5	43.9*	41.5*	22.0	19.5
Fall 2021	84	42.9%	239.1±16.1	23.8	22.6*	2.3	2.3
Meta	42	45.2%	237.3±15.9	14.3	11.9*	0	0
No meta	42	40.5%	240.9±16.4	33.3	33.3*	4.8	4.8
All trials	335	21.8%	232.3±17.8	26.6*	22.1*	6.0	5.1
Meta	168	23.8%	231.4±17.5	19.0*	14.9*	3.6	2.4
No meta	167	19.8%	233.2±18.1	34.1*	29.3*	8.4	7.8

Table 1. Arrival weights and morbidity and mortality data. (*) indicates statistical difference (P<0.05, Chi‐square test).

**Table:** Table 2

Trial	Arrival Mh culture	3‐week Mh Culture	10‐week Mh Culture
Fall 2019	15.5%	39.0%	4.8%
Meta	26.2%	48.8%	4.8%
No meta	4.8%	29.3%	4.8%
Fall 2020	20.2%	55.6%	9.5%
Meta	19.0%	58.5%	14.3%
No meta	21.4%	52.5%	4.8%
Spring 2021	8.4%	28.9%	20.0%
Meta	4.8%	37.5%	23.7%
No meta	11.9%	19.4%	15.6%
Fall 2021	42.9%	48.2%	12.3%
Meta	45.2%	42.9%	14.3%
No meta	40.5%	53.7%	10.2%
All trials	21.8%	43.2%	11.3%
Meta	23.8%	46.9%	14.0%
No meta	19.8%	39.2%	8.4%

*M. haemolytica* isolation rate.

**Conclusions:** BRD morbidity was reduced in calves receiving metaphylaxis, but the effect varied across trials. MH isolation was not decreased in META cattle, suggesting MH persisted despite metaphylaxis. Further investigation of the impact of AMR on MH in cattle receiving metaphylaxis is warranted.

## 263 Abstract F09

### 263.1 Fecal Microbiota of Diarrheic Calves and Its Association with Acid‐Base Disorders

#### 263.1.1 **Diego E. Gomez** ^1^; Lynna Li^1^; Hanne Goetz^2^; Jennifer MacNicol^3^; Lisa Gamsjaeger^4^; David Renaud^5^

##### 263.1.1.1 ^1^Department of Clinical Studies, Ontario Veterinary College, University of Guelph; ^2^Department of Population Medicine, Ontario Veterinary College, University of Guelph; ^3^Department of Animal Biosciences, Ontario Agricultural College, University of Guelph; ^4^Bovine Medicine, Vetsuisse Faculty, University of Zurich; ^5^Department of Population Medicine, Ontario Veterinary College, University of Guelph

**Background:** During diarrhea, an alteration of the bacterial communities of the gut occurs as does the acid‐base balance of calves.

**Objective:** To compare the fecal microbiota of healthy and diarrheic calves and investigate its association with acid‐base imbalances.

**Animals:** The calves used in this study were sourced from local dairy farms, auctions, and a drover. Fecal samples from twenty healthy dairy calves with normal fecal scores and 31 diarrheic calves with loose or watery fecal consistency were collected.

**Materials and Methods:** The fecal microbiota for this case‐control study was assessed by sequencing the 16S ribosomal RNA gene amplicons. Blood gas analysis was performed using an i‐Stat analyzer.

**Results:** The fecal community membership and structure were significantly different between healthy and diarrheic calves (P<0.001). In healthy calves, Lachnospiraceae*,* Ruminococcaceae, *Butyricicoccus*, *Phocaeicola*, *Bacteroides*, *Prevotella* and *Faecalibacterium*, were enriched, while *Escherichia/Shigella*, *Enterococcus*, *Gallibacterium, Streptococcus*, *Lactobacillus* and *Ligilactobacillus* were enriched in diarrheic calves. In diarrheic calves, an enrichment in lactate‐producing bacteria (*Lactobacillus*, *Streptococcus*, *Ligilactobacillus*, *Olsenella* and *Veillonella*) was identified. Diarrheic calves had a lower blood pH and HCO_3_^‐^ concentration and a higher AG than healthy calves (P<0.05 for all comparisons).

**Conclusions:** A shift from obligated to facultative anaerobes and expansion of lactate‐producing bacteria was identified in diarrheic calves and this shift was related to acidemia and an increased AG. This suggests an important role of the gut microbiota in the development of AG acidosis in diarrheic calves.

## 264 Abstract F10

### 264.1 Bovine Myeloid Antimicrobial Peptide‐28 (BMAP‐28) mRNA Expression by Bovine Cells and Effects on *Mannheimia haemolytica*

#### 264.1.1 **Santiago Cornejo Tonnelier** ^1^; Cassandra Barber^2^; Merrilee Thoresen^2^, PhD; Daryll Vannover^3,4^; Hannah Peck^3,4^; Philip Santangelo^3,4^; Amelia Woolums^2^, DVM, MVSc, PhD, DACVIM, DACVM

##### 264.1.1.1 ^1^Mississippi State University; ^2^Pathobiology and Population Medicine, Mississippi State University; ^3^Biomedical Engineering, Georgia Tech; ^4^Emory University

**Background:** *Mannheimia haemolytica* (MH) is the principal bacterial pathogen associated with bovine respiratory disease (BRD) in cattle. Existing antimicrobials do not consistently prevent BRD due to this pathogen; bovine antimicrobial peptides (AMP) have immune‐stimulating and antimicrobial effects that could improve BRD control. Messenger RNA (mRNA) treatment could be used to induce AMP expression in cattle, but efficacy must first be confirmed *in vitro*.

**Hypothesis/Objectives:** We hypothesized that bovine cells can express synthetic mRNA coding for the AMP BMAP‐28 and that synthetic BMAP‐28 can inhibit the growth of MH.

**Methods:** Madin‐Darby bovine kidney cells were cultured and transfected with mRNA coding for BMAP‐28 linked to the reporter nanoluciferase. After 4, 12, 24, and 72 hrs, relative light units (RLU) and protein concentration were measured. Results were expressed as RLU/μg of protein.

MH at 500 cfu/ml was incubated with synthetic BMAP‐28 at 10 or 100 μg/ml for 0, 12, or 24 hrs and quantitative culture was performed.

**Results:** Bovine kidney cells expressed mRNA coding for BMAP‐28 with peak expression occurring at 24 hrs in cell lysates and supernatants. Synthetic BMAP‐28 at 10 μg/ml inhibited MH growth at 12 and 24 hrs post‐treatment.

**Conclusions and Clinical Importance:** Treatment of bovine cells with synthetic mRNA induces BMAP‐28 expression *in vitro*. MH growth can be inhibited by BMAP‐28. These results provide support for further research to test the mRNA‐expressed product against BRD pathogens *in vitro* and *in vivo*. mRNA treatment to induce AMP expression could lead to new BRD control strategies.

## 265 Abstract F11

### 265.1 Associations Between Measured Climate Parameters, Barn Characteristics, and Health Indicators in Swiss Veal Calf Herds

#### 265.1.1 **Mireille Meylan**; Christoph Weber, Dr. med. vet.; Petra Bucher‐Schnyder, Dr. med. vet.; Lutz Schönecker, Dr. med. vet., DACVM; Dimitri Stucki, Dr. Sc. Nat., PhD

##### 265.1.1.1 University of Bern, Switzerland

**Background:** Easy‐to‐use devices to measure climate parameters are available commercially, but their use in veal calf barns has not been evaluated.

**Hypothesis/Objectives:** Comparison of climate parameters measured with different methods in different locations in calf barns, evaluation of associations between barn characteristics and measured climate parameters, and evaluation of associations of measured climate parameters and barn characteristics with indicators of calf health (antimicrobial use, AMU, mortality, and daily weight gain, DWG).

**Animals:** 4014 veal calves in 43 veal calf operations.

**Methods:** Prospective cohort study; each farm was visited 6 times over one year; temperature, humidity, ammonia and carbon dioxide concentrations were measured each time in 5 locations. Temperature and humidity were also measured continuously over 72 hours in winter and summer. Barn characteristics (e.g., ventilation, outdoor pen, barn size) were recorded. Correlations among measured climate parameters, associations between barn characteristics and measured climate parameters, as well as whether AMU, mortality and DWG can be predicted based on barn characteristics and measured climate parameters were explored statistically.

**Results:** Relevant correlations were observed neither among measured climate parameters and locations nor between measured climate parameters and barn characteristics. Only farm characteristics (e.g., group size, ventilation or air volume per calf) were associated with calf health indicators.

**Conclusions and Clinical Importance:** Measured barn climate parameters were not associated with health indicators, and thus appear inadequate to predict calf health in veal fattening operations. These (easily) measured parameters are difficult to interpret and should be considered critically.

## 266 Abstract F12

### 266.1 Detection Times of Florfenicol/Florfenicol Amine in Lactating Meat and Dairy Goats, and Milk‐Fed Kids

#### 266.1.1 **Jennifer L. Davis** ^1^; Sierra Guynn^1^; Kevin Pelzer^1^; Emily Richards^2^; Melissa Mercer^2^; Scott Wetzlich^2^; Maaike Clapham^2^; Lisa Tell^2^

##### 266.1.1.1 ^1^VA‐MD College of Veterinary Medicine; ^2^UC Davis School of Veterinary Medicine

**Background:** Florfenicol is used extralabel in goats and data is needed to prevent illegal drug residues.

**Objectives:** To determine detection times of florfenicol and florfenicol amine in meat and dairy goat plasma and milk, and evaluate exposure in plasma and tissues in kids fed milk from treated does.

**Methods:** Florfenicol (40 mg/kg SC) was administered twice, q 96 h, to healthy, adult, lactating meat (n=8, volume: 4.1–7.6 mL, site: cervical) and dairy (n=5, volume 10.4–15.1 mL, site: axilla, split) goats. Plasma and milk samples were collected. Kids were allowed to nurse (meat breeds) or were bottle fed (dairy breeds) milk from treated does and plasma and tissue samples collected. Drug concentrations were determined by LC‐MS/MS (Quantification limit: 4 μg/mL plasma, 8 μg/mL milk, 0.1 ppm tissue).

**Results:** Florfenicol was quantified longer in dairy vs. meat goats in plasma (720 h vs. 432 h) and milk (624 h vs. 432 h). Meat goat kids allowed to nurse had higher plasma florfenicol concentrations at 24 h after the second dose than bottle‐fed dairy goat kids (18.7 μg/mL vs. 8.01 μg/mL), but drug was detected longer in dairy goat kids (7 d vs. 11 d). Maximum concentrations (≤670 ppb; liver) of florfenicol/florfenicol amine in kid tissues were below tolerance for cattle (3700 ppb).

**Conclusions and Clinical Importance:** Florfenicol is quantifiable in milk for up to 26 d in goats administered 40 mg/kg SC twice. Differences between meat and dairy goats may relate to volume and site of injection. Detection differed in kids exposed through milk; overall drug exposure and tissue concentrations were low.

## 267 Abstract F13

### 267.1 The Pharmacokinetics and Clinical Efficacy of Levamisole in Adult Alpacas after Oral Administration

#### 267.1.1 **Kylie McLaughlin** ^1^; Daniela Bedenice^2^, Dr. med. vet, DACVIM, DACVECC; Nicholas Pena^3^; Isabella Ceresia^4^; Mark Bohlke^5^; Lauren Holley^6^, DVM; Lauren Bookbinder^7^, DVM, DACVIM; Chloe Deveney^8^; Iman Zaghloul^9^, PhD; Michelle Ceresia^10^, PharmD, FACVP

##### 267.1.1.1 ^1^Massachusetts College of Pharmacy and Health Sciences; ^2^Associate Professor, Clinical Sciences, Cumming School of Veterinary Medicine at Tufts University; ^3^Student, North Salem High School; ^4^Student, University of Massachusetts Amherst; ^5^Massachusetts College of Pharmacy and Health Sciences; ^6^Resident, Clinical Sciences, Cumming School of Veterinary Medicine at Tufts University; ^7^Clinical Assistant Professor, Environmental & Population Health, Cummings School of Veterinary Medicine; ^8^Student, School of Arts, Sciences, and Engineering, College of Liberal Arts, Tufts University; ^9^Professor of Pharmaceutics, Massachusetts College of Pharmacy and Health Sciences; ^10^Professor, Pharmacy Practice, Massachusetts College of Pharmacy and Health Sciences

**Background:** Targeted anthelmintic use is indicated to counteract progressive emergence of anthelmintic‐resistant nematodes. The pharmacokinetics and pharmacodynamics of levamisole in alpacas currently remain unknown.

**Hypothesis/Objectives:** The study purpose was to characterize the disposition of orally administered levamisole‐HCL (8.8 mg/kg) and to evaluate the fecal egg count reduction (FECR, efficacy) after drug administration in adult alpacas of a single herd.

**Animals:** 8 clinically‐healthy adult Huacaya alpacas for pharmacokinetic assessment, and 44 alpacas with known HOT‐complex (*Haemonchus*/*Ostertagia*/*Trichostrongylus*) infection, evaluating FECR.

**Methods:** Heparinized plasma samples were obtained from 8 alpacas (2.2–15.1 years) at designated time‐points following oral Levamisole‐HCL (Prohibit Drench Powder^â^) administration. Samples were frozen at ‐80°C until assayed by liquid chromatography tandem mass‐spectrometry; and analyzed using WinNonlin‐6.4^â^. Additionally, clinical and FEC data were compared using repeated measures analyses, before and 2 weeks after deworming of HOT‐complex positive alpacas at the same dose.

**Results:** The pharmacokinetic parameters were calculated using non‐compartmental analysis. The mean maximum concentration, time to maximum concentration, area under the curve and mean residence time were 1.35±0.45 mg/L (C_max_), 0.26±0.13 hours (T_max_), 4.21±0.998 mg*h/L (AUC0‐¥) and 6.78±0.98 hours (MRT), respectively. The median oocyst count of 44 HOT‐complex affected alpacas was 300 opg (range: 150–4200), with a >99% FECR in 35/44 (80%) alpacas, 2 weeks after deworming. Five alpacas showed a 40–50% FECR, and 4 animals exhibited no HOT‐complex change. Neither hematocrit nor total solids changed related to treatment.

**Conclusions and Clinical Importance:** The AUC normalized for dose for alpacas was higher than reported in goats and lower than in sheep.

## 268 CARDIOLOGY

## 269 Abstract C25

### 269.1 Effect of Audible Static on Blood Pressure Measurements by Doppler Ultrasonic Sphygmomanometry in Cats

#### 269.1.1 **Sayaka Uematsu** ^1^; Jessica Quimby^2^, DVM, PhD, DACVIM (SAIM); Stacie Summers^3^, DVM, DACVIM (SAIM), PhD

##### 269.1.1.1 ^1^Oregon State University; ^2^Associate Professor, Department of Veterinary Clinical Sciences, Ohio State University; ^3^Assistant Professor, Carlson College of Veterinary Medicine, Oregon State University

**Background:** It is not known whether audible static from the Doppler ultrasonic flow detector device affects blood pressure readings in cats.

**Objective:** To determine whether the use of headphones by the veterinary professional during Doppler ultrasonic sphygmomanometry alters the average blood pressure measurement in conscious cats.

**Animals:** Twenty‐seven client‐owned cats (>1 year)

**Methods:** Randomized, crossover study design. Healthy cats (>1 year) were enrolled. Blood pressure measurements using the right forelimb were obtained twice 14 days apart with Doppler ultrasonic sphygmomanometry with or without the use of headphones by a veterinarian. A fear, anxiety and stress (FAS) score (0 = relaxed; 1 = mild signs; 2–3 = moderate signs; 4 = severe signs) was recorded. The first blood pressure measurement was discarded and the average measurement was calculated using the remaining 5 measurements. A repeated measures two‐way ANOVA was used to compare the effect of headphones on blood pressure measurements. Spearman correlation was used to determine association between blood pressure measurement and FAS score.

**Results:** Seventeen adult cats and 10 mature and senior cats with a median age of 4 years (range, 1–14 years) participated. The mean±SD blood pressure measurement taken with headphones (133±19 mm Hg) was lower compared to the measurement taken without headphones (143±18 mm Hg; mean difference, 10±SE 4 mm Hg; P=0.01). No association between the average blood pressure measurement and FAS score was found.

**Conclusions:** The use of headphones during Doppler ultrasonic sphygmomanometry reduces situational hypertension in conscious cats.

## 270 Abstract C26

### 270.1 Mitral Regurgitation Severity Index Predicts Outcome in Diverse Populations of Canine Myxomatous Mitral Valve Disease

#### 270.1.1 **Michelle M. Vereb** ^1^; Clarke Atkins^2^, DVM, DACVIM (SAIM and Cardiology); Darcy Adin^3^, DVM, DACVIM (Cardiology); Thomas Blondel^4^; Melissa Coffman^5^, DVM; Seunggon Lee^6^, MS, PhD, DAiCVIM (Asian College of Veterinary Medicine, Cardiology); Emilie Guillot^4^, DVM; Jessica Ward^1^, DVM, DACVIM (Cardiology)

##### 270.1.1.1 ^1^Iowa State University; ^2^North Carolina State University; ^3^University of Florida; ^4^Ceva Santé Animale; ^5^Ceva Animal Health; ^6^Seoul Animal Heart Hospital

**Background:** Outcome is variable in dogs with preclinical myxomatous mitral valve disease (MMVD). Mitral regurgitation severity index (MRSI) combines 3 variables (heart rate [HR], age, and left atrium to aorta ratio [LA:Ao]) into a single index that appears to have prognostic utility.

**Hypothesis/Objectives:** MRSI will predict time to first‐onset congestive heart failure (CHF) and all‐cause mortality in dogs with MMVD stage B2 and will have superior prognostic utility compared to its constituent variables alone.

**Animals:** 235 client‐owned dogs with MMVD Stage B2 from 3 diverse populations.

**Methods:** Retrospective study pooling data from 3 study populations. MRSI was calculated as: ([HR]/120 × LA:Ao × [age in years]/10 × 100). Cox proportional hazard modeling and time‐dependent receiver‐operator characteristic curves quantified prognostic performance of MRSI, HR, LA:Ao, and age. The maximally selected rank statistics determined the optimal MRSI cutoff.

**Results:** MRSI >156 was predictive of time to CHF (median 407 days for MRSI >156 vs. 1404 days for MRSI ≤156; AUC 0.68; hazard ratio 3.05 [95% CI 1.9–4.9]; p<0.001). MRSI >173 was predictive of all‐cause mortality (median survival 868 days for MRSI >173 vs. 1843 days for MRSI ≤171; AUC 0.63; hazard ratio 3.98 [95% CI 2.3–6.9]; p<0.001). MRSI showed superior predictive value compared to HR, LA:Ao, or age.

**Conclusions and Clinical Importance:** MRSI was predictive of outcome and outperformed each index component alone. MRSI shows potential as a clinically useful prognostic tool in MMVD stage B2.

## 271 Abstract C27

### 271.1 Serial Evaluation of Left Ventricular Systolic Function in Dogs Undergoing Chemotherapy with Doxorubicin

#### 271.1.1 **Marlos Gonçalves Sousa** ^1^; Marcela Wolf^1^; Stephany Lucina^1^; Vinícius Silva^1^; Matheus Silveira^1^; Victoria Silva^1^; Claudia Custódio^2^

##### 271.1.1.1 ^1^Federal University of Paraná; ^2^Clinivet Veterinary Hospital

**Background:** Cardiotoxicity leading to left ventricular dysfunction is a possible complication in patients undergoing chemotherapy. Advanced echocardiography techniques, such as the global longitudinal strain (GLS) and tissue motion annular displacement (TMAD), allow the early detection of myocardial dysfunction due to cardiotoxicity in relation to conventional examination in human beings with cancer.

**Objectives:** To assess whether TMAD, GLS and the global circumferential strain (GCS) are able to detect early cardiotoxicity in dogs.

**Animals:** 12 dogs with two different types of cancer (N=6 multicentric lymphoma; N=6 breast adenocarcinoma) undergoing chemotherapy with doxorubicin.

**Methods:** 12 dogs undergoing chemotherapy with doxorubicin combined with other chemotherapy agents underwent conventional echocardiography and Speckle tracking analysis before chemotherapy (day 0), 7 (7d), 60 (60d), 120 (120d) and 180 (180d) days after the first administration of doxorubicin.

**Results:** There was no difference in volume variables by Simpson's method (P>0.05), as well as fractional shortening (P=0.59), GLS (P=0.28) and GCS (0.183) at different evaluation times. However, a reduction was observed in the lateral MAPSE index (P=0.0233), and in the global TMAD% (P=0.046) when compared to the pre‐chemotherapy evaluation with 180d (figure 1), with a mean of 2.06 cm/m^2^ (1.17‐3.19) and 1.64 cm/m^2^ (0.83‐2.77) and median of 12.07% (±2.34) and 14.52% (±2.72) (table 1), respectively.

**Conclusion:** A reduction in longitudinal systolic function can be detected by MAPSE index and TMAD in dogs receiving a considered safe dose of doxorubicin.

**Table:** Table 1. Parameters representing the group, cumulative dose of doxorubicin and the main echocardiographic variables compared over time.

	Pre (day 0)	7 d	21 d	60 d	120 d	180 d	P
(N)	12	12	12	12	12	12	
M/F	5/7	5/7	5/7	5/7	5/7	5/7	
Cumulative dose of doxorubicin (mg/kg)	0	1 (0.88–1)	1 (0.88–2)	2 (1.74–3)	4 (2.6–5)	6 (3.45–6)	
E_mitral_ (cm/s)	72.95 (49.0–123.0)	70.20 (51.60–115.0)	71.45 (56.50–122.0)	76.45 (53.40–111.0)	72.95 (51.40–105.0)	69.65 (43.90–139.0)	0.6281
A_mitral_ (cm/s)	87.20 (70.10–103)	87.05 (53.80–113.0)	81.05 (53.80–114.0)	84.15 (47.60–95.50	78.70 (61.30–108.0)	75.20 (49.60–173.0)	0.6219
E:A	0.84 (0.58–1.28)	0.90 (0.57–1.18)	0.95 (0.64–1.50)	0.94 (0.57–1.80)	0.95 (0.60–1.30)	0.92 (0.50–1.70)	0.772
IVRT (ms)	63.33 (±9.92)	65.42 (±13.71)	62.25 (±7.68)	66.33 (±9.71)	65.0 (±14.23)	69.83 (±10.33)	0.3426
LA/Ao	1.15 ab (1.08–1.40)	1.14a (1.0–1.38)	1.29b (1.06–1.56)	1.30b (1.10–1.53)	1.23ab (1.0–1.67)	1.27ab (1.0–1.67)	**0.0086**
LVIDD_n_	1.45 (1.21–1.75)	1.47 (1.29–1.71)	1.40 (0.45–1.78)	1.49 (1.30–1.70)	1.41 (1.33–1.91)	1.45 (1.24–1.73)	0.8652
LVIDS_n_	0.82 (0.59–1.07)	0.82 (0.61–0.96)	0.77 (0.71–1.30)	0.78 (0.70–0.98)	0.81 (0.60–1.15)	0.85 (0.56–0.96)	0.7526
FS (%)	41.89 (±6.65)	42.51 (±6.67)	43.76 (±6.29)	42.41 (±7.36)	42.24 (±6.60)	40.08 (±7.26)	0.5960
EF (AP4 Simpson's method) (%)	75.45 (62.20–80.50)	72.55 (61.0–81.50)	71.70 (59.10–75.80)	73.0 (68.30–80.80)	72.65 (59.20–77.30)	70.35 (60.50–79.0)	0.0970
EF (AP2 Simpson's method) (%)	74.76 (±7.12)	75.87 (±6.42)	72.28 (±6.51)	73.79 (±6.33)	73.56 (±6.41)	69.89 (±3.99)	0.0609
EF (Simpson's Biplane) (%)	74.24 (±5.05)	73.75 (±6.41)	71.66 (±5.67)	74.15 (±3.26)	72.62 (±4.92	70.31 (±4.29)	0.1440
EDVdi AP4 (ml/m^2^)	38.34 (18.98–80.21)	36.49 (20.65–72.07)	37.18 (23.19–63.20)	38.50 (17.70–57.85)	37.90 (18.34–84.61)	37.85 (22.80–64.91)	0.4159
ESVdi AP4 (ml/m^2^)	8.75 (5.94–24.20)	9.90 (5.04–22.97)	11.63 (5.61–22.80)	9.71 (4.14–18.37)	11.16 (4.16–20.49)	11.63 (5.84–15.22)	0.2837
S'septal (cm/s)	10.97 (±2.54)	10.33 (±1.74)	10.16 (±1.46)	10.60 (±2.88)	9.60 (±2.07)	10.33 (±2.10)	0.4119
E’ septal (cm/s)	7.41 (4.93–15.30)	6.49 (5.07–8.59)	7.34 (4.54–11.80)	7.61 (4.87–9.63)	6.62 (5.17–9.55)	6.66 (4.77–9.33)	0.3696
A'septal (cm/s)	10.09 (±1.85)	9.11 (±1.98)	9.29 (±1.93)	9.60 (±2.14)	8.94 (±2.54)	9.32 (±2.66)	0.4179
S’ lateral (cm/s)	11.40 (8.41–20.10)	11.05 (7.96–14.30)	11.05 (6.45–13.70)	10.80 (6.29–17.40)	10.30 (7.76–18.10)	9.36 (6.96–13.80)	0.2693
E’ lateral (cm/s)	8.45 (5.57–15.60)	7.80 (4.77–12.60)	7.30 (4.62–11.60)	7.60 (5.10–12.20)	7.47 (5.57–14.30)	6.79 (4.89–11.30)	0.1519
A’ lateral (cm/s)	11.0 (8.43–15.80)	9.77 (6.27–21.0)	8.82 (6.67–14.0)	9.78 (6.77–13.90)	9.66 (7.16–15.10)	10.40 (6.68–12.40)	0.1673
MAPSEi lateral (cm/m^2^)	2.06^a^ (1.17–3.19)	1.93^a^ (1.0–3.33)	1.82^ab^ (0.70–2.88)	1.83^ab^ (0.93–3.38)	1.66^ab^ (0.88–3.20)	1.64^b^ (0.83–2.77)b	0.0041
MAPSEi septal (cm/m^2^)	2.18^a^ (0.97–3.24)	1.97^ab^ (0.76–2.81)	1.81^b^ (0.85–2.73)	1.87^ab^ (0.71–3.79)	1.84^ab^ (0.89–2.62)	1.73^ab^ (0.75–2.68)	0.0174
Strain AP4 (%)	22.80 (±4.46)	21.91 (±4.55)	22.10 (±4.05)	20.64 (±4.28)	21.58 (±4.33)	20.32 (±4.85)	0.0945
Strain AP3 (%)	18.69 (±3.10)	19.85 (±6.10)	19.13 (±4.21)	17.83 (±3.19)	19.24 (±3.65)	18.38 (±4.25)	0.4309
Strain AP2 (%)	22.43 (±4.32)	22.39 (±6.43)	21.36 (±4.43)	22.96 (±5.42)	22.62 (±4.42)	20.27 (±4.68)	0.3066
GLS (%)	21.30 (±3.24)	21.38 (±5.40)	20.86 (±3.95)	20.48 (±3.82)	21.14 (±3.87)	19.65 (±4.29)	0.2889
GCS (%)	18.62 (±4.60)	18.16 (±4.03)	19.20 (±3.41)	18.04 (±3.54)	18.92 (±1.48)	19.11 (±3.14)	0.183
Global TMAD(%)	14.52 (±2.72)^a^	13.16 (±2.15)^ab^	13.48 (±2.35)^ab^	13.37 (±2.23)^ab^	13.18 (±2.23)^ab^	12.07 (±2.34)^b^	0.0465

(N): number of animals in each group AP4: apical 4‐chamber; AP2: apical two‐chamber; EF: ejection fraction; E_mitral_: early diastolic mitral inflow velocity; A_mitral_: late diastolic mitral inflow velocity; E’: ‘peak velocity of early diastolic mitral annular motion as determined by pulsed wave Doppler; A’: peak velocity of diastolic mitral annular motion as determined by pulsed wave Doppler; FS: fractional shortening; GCS: global circumferential strain; GLS: global longitudinal strain IVRT: isovolumic relaxation time; LA/Ao: left atrium‐to‐aorta ratio; LVIDd_n_: normalized left ventricular internal dimension at end‐diastole; LVID_sn_: normalized left ventricular internal dimension at end‐systole; MAPSEi: mitral annular plane systolic excursion indexed to the body surface area; S’: peak velocity of systolic mitral annular motion as determined by pulsed wave Doppler; TMAD: tissue motion annular displacement. Data with normal distribution were expressed by the mean and standard deviation and data with abnormal distribution were expressed by the median and interquartile range. Values with different superscripted letters indicate statistically significant differences between groups and equal letters represents equality between groups.

**Figure:**
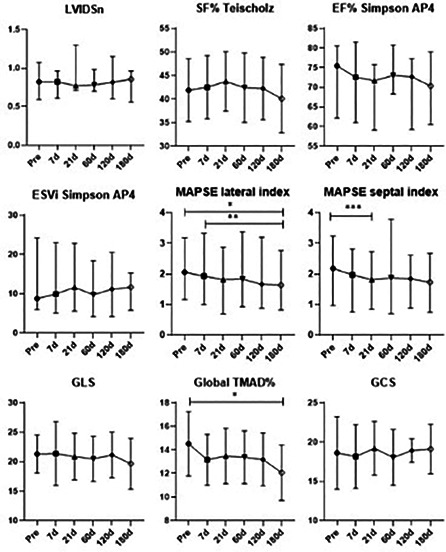
**Figure 1** Graphs representing the main echocardiographic variables of systolic function compared over time. The differences between the groups are represented by the bars, in which (*) represents the difference between the pre‐chemotherapy and 180‐day assessment, (**) between the 7‐day and 180‐day assessment, and (***) between the pre‐ and 21‐day assessment.

## 272 Abstract C28

### 272.1 Procedural Surgical Protocol for Canine Mitral Valve Repair

#### 272.1.1 **Takuma Aoki** ^1^; Takashi Miyamoto^2^; Naoyuki Fukamachi^3^; Yao Jingya^1^; Seiya Niimi^1^; Yoshito Wakao^1^

##### 272.1.1.1 ^1^Azabu University, Doctor of Veterinary Science Department; ^2^Kodama Kyodo Hospital; ^3^Gunma Chidren's Medical Center

**Background:** Lack of objective and reproducible surgical techniques for mitral regurgitation (MR) in dogs.

**Hypothesis/Objectives:** To develop a procedural protocol for mitral valve repair.

**Animals:** Six consecutive client‐owned dogs with MR underwent surgery at the Azabu University Veterinary Teaching Hospital in 2021.

**Methods:** Surgical procedure protocol included the Modified Loop technique and De Vega annuloplasty (MOLD). In MOLD, an artificial loop comprising 80% of the length of the second order chordae tendineae is attached to the tip of the mitral valve leaflets. A purse string suture was used for the mitral annulus, so that the anterior leaflet could act as a monocusp.

**Results:** The stages that followed the guidelines were stages B2 and C in three dogs each. Breeds included mongrel, Spitz, Pomeranian, Cavalier King Charles Spaniel, and Chihuahua, with an average age and weight of 11.4±2.3 years and 5.49±2.98 kg, respectively. The aortic cross‐clamp, pumping, and operative times were 64.0±7.5, 168.5±39.1, and 321.0±53.1 minutes, respectively. After MOLD, left atrial‐to‐aortic ratios significantly decreased from 2.20±0.18 to 1.26±0.22 (p<0.01); left ventricular internal diameter end diastole normalized for body weight significantly decreased from 2.03±0.26 to 1.48±0.20 (p<0.05). Both of the above postoperative echocardiographic indices were within normal limits. In all cases, clinical signs significantly improved or disappeared.

**Conclusions and Clinical Importance:** MOLD fixed the heart defect and clinical signs in dogs with MR significantly improved.

## 273 Abstract C29

### 273.1 Retrospective Evaluation of Sacubitril/Valsartan (Entresto) in 20 Dogs with Advanced Congestive Heart Failure

#### 273.1.1 **Charlotte R. Donnan**; Jonathan Lichtenberger, DVM, MSc., DACVIM (Cardiology)

##### 273.1.1.1 Toronto Animal Health Partners

**Background:** Sacubitril/valsartan (Entresto) is a novel drug combining an angiotensin receptor blocker and neprilysin inhibitor that has been demonstrated to provide survival benefits in humans with congestive heart failure (CHF).

**Hypothesis/Objectives:** To describe the use of Entresto in dogs with naturally‐occurring CHF that have failed standard therapy.

**Animals:** Twenty dogs from a single hospital population were included—17 with myxomatous mitral valve disease and three with dilated cardiomyopathy.

**Methods:** Electronic medical records of dogs receiving Entresto were reviewed retrospectively.

**Results:** Entresto was initiated at a median dose of 12.6 mg/kg (range 5.9–23.1 mg/kg) PO q 12 h. The median time in CHF prior to Entresto was 6.9 months (range 2.3–26.9 months). Dose adjustments occurred in six dogs: increased in five (max dose 29.4 mg/kg q 12 h) and discontinued following mitral valve repair in one. No dogs required dose reduction or discontinuation due to side effects. Creatinine increased mildly but significantly following initiation of Entresto (p<0.001, median difference of 26 μmol/L). There was no significant change in BUN or electrolyte concentrations after initiating Entresto.

The median duration of Entresto administration was 6.8 months (0.6–31.1 months). Eight dogs are still alive at the time of writing. Nine died or were euthanized due to cardiac disease, two were euthanized due to azotemia, and one died of a non‐cardiac cause.

**Conclusions and Clinical Importance:** Entresto appears to be well tolerated in the majority of dogs and may be clinically beneficial. Prospective studies are needed to determine its efficacy compared to conventional angiotensin‐converting enzyme inhibitors.

## 274 Abstract C30

### 274.1 Case Series of Six Dogs with Primary Tricuspid and Right Ventricular Outflow Tract Neoplasms

#### 274.1.1 **Laura Letwin** ^1^; Sara Degl'Innocenti^2^; Antonia Morey Matamalas^2^, BVM, MRCVS, AFHEA; Hayley McDonald^1^, BVSc(Distinction), DECVIM (Cardiology), PhD

##### 274.1.1.1 ^1^Dick White Referrals; ^2^School of Veterinary Medicine and Science, The University of Nottingham

**Background:** Primary tricuspid and right ventricular outflow tract neoplasms have been associated with poor outcomes in canine patients. However, clinical signs, histological features and outcome of right‐sided cardiac masses are limited to single case reports.

**Objectives:** To describe the historical, physical examination, histological features and echocardiographic findings in a series of dogs with tricuspid or right ventricular outflow tract masses.

**Animals:** Six dogs with primary right‐sided intracavitary cardiac masses.

**Methods:** A retrospective case series. Medical records of dogs with relevant cardiac masses were reviewed to collect data including clinical history, echocardiographic findings, treatment and outcomes. Data from post‐mortem examinations was also available and reviewed for two dogs.

**Results:** Echocardiography identified dogs with a tricuspid mass (n=5) and a right ventricular outflow tract mass (n=1). Common presenting clinical signs were increased respiratory rate and effort, abdominal distension, exercise intolerance and excitement‐related syncopal episodes. Physical exam findings included: heart murmur (n=5) and evidence of congestive heart failure (n=5). Three dogs were alive at the end of the study (5–11 months post diagnosis) while three dogs were euthanized within three months of diagnosis. Post‐mortem examination available for two dogs with tricuspid masses, revealed an extra skeletal osteosarcoma and soft tissue sarcoma.

**Conclusions and Clinical Importance:** Primary intracardiac masses are rarely reported in dogs and can have unusual histogenesis (osteosarcoma). Congestive heart failure can be a common clinical manifestation in these patients with a poor long‐term outcome, whereas other cases have been associated with reasonable survival time post‐diagnosis.

## 275 Abstract C31

### 275.1 Retrospective Evaluation of the Effect of Sotalol on Survival in Dogs with Severe Subaortic Stenosis

#### 275.1.1 **Annie Showers** ^1^; Sonja Tjostheim^2^, DVM, DACVIM (Cardiology); Caitlin Obernberger^3^, CVT; Melissa Shear^4^, DVM

##### 275.1.1.1 ^1^School of Veterinary Medicine, University of Wisconsin; ^2^Clinical Assistant Professor, School of Veterinary Medicine, University of Wisconsin; ^3^Veterinary Student, School of Veterinary Medicine, University of Wisconsin; ^4^Veterinarian, VCA Veterinary Emergency Service and Veterinary Specialty Center

**Background:** Dogs with severe subaortic stenosis (SAS) are at risk of dying suddenly from fatal arrhythmias. Survival is not improved when treated with pure beta‐adrenergic receptor (β) blockers; however, the effect of other antiarrhythmic drugs on survival is unknown. Sotalol is both a β‐blocker and a class III antiarrhythmic drug, two anti‐arrhythmic mechanisms that might be well suited for dogs with severe SAS.

**Hypothesis/Objectives:** To evaluate the effect of sotalol on survival time in dogs diagnosed with severe SAS.

**Animals:** Forty‐three owned dogs diagnosed with severe SAS (pressure gradient ≥80 mm Hg) between 2003 and 2021.

**Methods:** Retrospective cohort study. The primary outcome was to compare survival in dogs with severe SAS that were treated with either sotalol or atenolol. The secondary outcome was to evaluate the effect of pressure gradient, age, breed, and aortic regurgitation on survival.

**Results:** No statistical difference was identified in survival time between dogs treated with sotalol (n=14) and those treated with atenolol (n=29) when evaluating all‐cause mortality (p=0.17, Figure 1) or cardiac‐related mortality (p=0.16). Of the dogs that died suddenly, survival time was significantly shorter in dogs treated with sotalol compared to those treated with atenolol (p=0.05, Figure 2). Univariable analysis showed that pressure gradient and age at diagnosis had a significant effect on survival.

**Figure:**
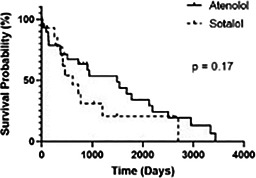
**Figure 1** Kaplan‐Meier survival curve of all‐cause mortality in dogs with severe subaortic stenosis treated with atenolol (n=29) or sotalol (n= 4). Tick marks represent censored data for animals still alive at the study end or lost to follow‐up. There is no significant difference in survival between groups (p=0.17).

**Figure:**
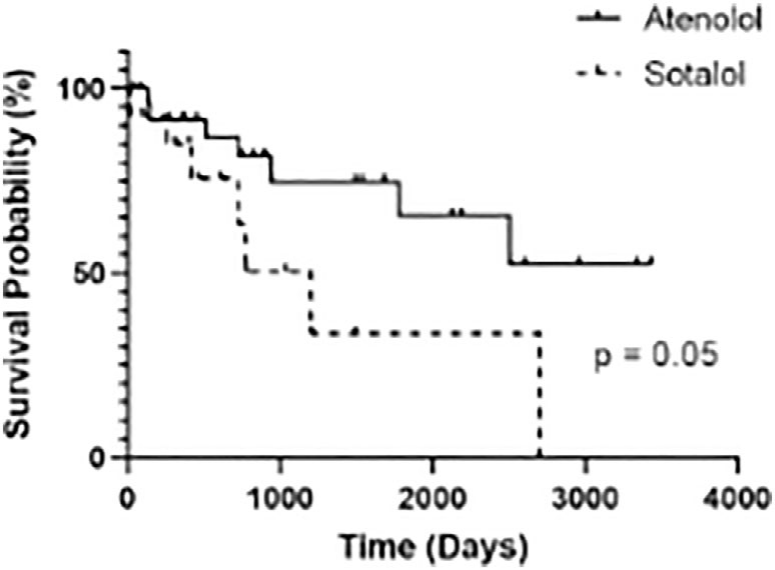
**Figure 2** Kaplan‐Meier survival curve of dogs with severe subaortic stenosis that died suddenly during treatment with atenolol (n=29) or sotalol (n=14). Survival time was significantly shorter in dogs treated with sotalol compared to dogs treated with atenolol (p=0.05).

**Conclusion:** Sotalol did not have a significant effect on survival overall but may increase the risk of sudden death in dogs with severe SAS.

## 276 Abstract C32

### 276.1 Effect of Autologous Blood Collection for Mitral Valve Repair on Hemodynamics and Cardiac Function

#### 276.1.1 **Arane Takahashi** ^1^; Masami Uechi^2^, DVM, PhD, DAiCVIM

##### 276.1.1.1 ^1^JASMINE Animal Referral Hospital/Cardiovascular Medical Center Cardiology and Anesthesiology Unit; ^2^JASMINE Animal Referral Hospital

**Background:** Mitral regurgitation (MR) in dogs is treated with surgery for mitral valve repair (MVR), which often requires blood transfusion. Autologous blood collection (ABC) is optimal to supply the limited blood resource but there are no reports on the safety of ABC during MVR in dogs.

**Objective:** To evaluate cardiac function following ABC in dogs undergoing MVR.

**Animals:** Dogs with MR undergoing MVR during 2017–2018 were included. ABC was performed if cardiomegaly was present and the mean blood pressure was maintained above 40 mm Hg during the anesthesia.

**Methods:** Approximately 10% of the circulating blood volume (CBV) was taken from the jugular vein under anesthesia. Cardiac function values were compared before and after ABC.

**Results:** One dog died due to postoperative hemorrhage. A significant reduction was observed in E wave, mitral annular area, left ventricular volume, stroke volume (SV), cardiac output (CO), and mitral regurgitation color flow area (MRCFA). Blood pressure (BP) and ejection fraction did not change.

**Discussion:** No apparent side effects were observed following ABC. E wave reduction with no BP changes indicated a decrease in left atrial preload. Reduction of the valvular area and MRCFA suggested improvement of mitral coaptation and MR. The decreases in CO and SV were explained by a reduction of CBV due to ABC.

**Conclusion and Clinical Importance:** Intraoperative ABC could be safely achieved in dogs with MR for approximately 10% of the CBV. Moreover, it reduced the left atrial preload. Further research on hemodynamic changes of CO is required.

## 277 Abstract C33

### 277.1 Tissue Renin‐Angiotensin System Enzyme Activity in Canine Post‐Mortem Myocardial and Kidney Samples

#### 277.1.1 **Emma C. Weitzhandler** ^1^; Marisa Ames^2^, DVM, DACVIM (Cardiology); Oliver Domenig^3^, PhD

##### 277.1.1.1 ^1^School of Veterinary Medicine, University of California, Davis; ^2^Associate Professor, Medicine and Epidemiology, University of California, Davis; ^3^Head of Laboratory, Attoquant Diagnostics GmbH

**Background:** Methods to quantify the activity of the tissue renin‐angiotensin system (RAS) are needed.

**Hypothesis/Objectives:** RAS enzyme activity is quantifiable in post‐mortem tissue.

**Animals:** Five dogs that died or were euthanized for various reasons unrelated to this study.

**Methods:** Paired myocardial and kidney samples were taken at necropsy and seven hours later. Tissues were homogenized and incubated with angiotensin I (AngI) under four conditions: no additive, chymostatin (chymase‐inhibitor), chymostatin and lisinopril (angiotensin‐converting enzyme [ACE]‐inhibitor), or LBQ‐657 (neutral endopeptidase [NEP]‐inhibitor) or incubated with angiotensin II (AngII) under three conditions: no additive, ZPP (prolyl‐carboxypeptidase [PCP]‐inhibitor), or ZPP and MLN‐4760 (ACE2‐inhibitor). The production of AngII or angiotensin 1‐7 (Ang1‐7) was measured via liquid chromatography‐tandem mass spectrometry, which quantifies activities of AngII formers ACE and chymase and Ang1‐7 formers ACE2, PCP, and NEP.

**Results:** Production rates of AngII and Ang1‐7 are shown in Table 1. Initial samples were collected 5 to 28 hours post‐mortem. Angiotensin converting enzyme was responsible for most AngII formation in myocardial and kidney samples. Both PCP and ACE2 contributed to Ang1‐7 formation in the myocardial samples, whereas ACE2 played a larger role in Ang1‐7 formation in kidney samples. Neprilysin did not play a major role in Ang1‐7 formation in either tissue. There was ‐32 to +40% change in the ratio of Ang1‐7 to AngII formation between baseline and paired myocardial samples (Figure 1).

**Table:** Table 1. Median (min‐max) concentrations of angiotensin peptides produced from homogenized tissues incubated with and without various enzyme inhibitors.

	AngII formation (pg/μg protein)/h Median (min‐max)			Ang1‐7 formation (pg/μg protein)/h Median (min‐max)			Ang1‐7 formation (pg/μg protein)/h Median (min‐max)	
	Control	Chymostatin	Chymostatin and lisinopril	Control	ZPP	ZPP and MLN‐4760	Control	LBQ‐657
LVPW	24.5 (14.8–46.9)	24.7 (13.8–43.5)	all below LLOQ	613 (277–935)	178 (140–340)	all below LLOQ	2004 (1831–2334)	1824 (1760–2238)
IVS	28 (17.9–54.9)	22.5 (15.1–54.0)	all below LLOQ	647 (283–919)	155 (118–355)	all below LLOQ	2232 (1746–2793)	2005 (1640–2733)
Kidney	557.2 (114.7–1485.4)	553.9 (110.5–1418.3)	all below LLOQ	24297 (14573–29700)	21960 (12213–26524)	889.5 (508–1372)	3862 (3221–4132)	1964.5 (1810–2641)

Ang1‐7, Angiotensin 1‐7; AngII, Angiotensin II; IVS, interventricular septum; LLOQ, lower limit of quantitation (<5 [pg/μg protein]/h); LVPW, left ventricular posterior wall.

**Figure:**
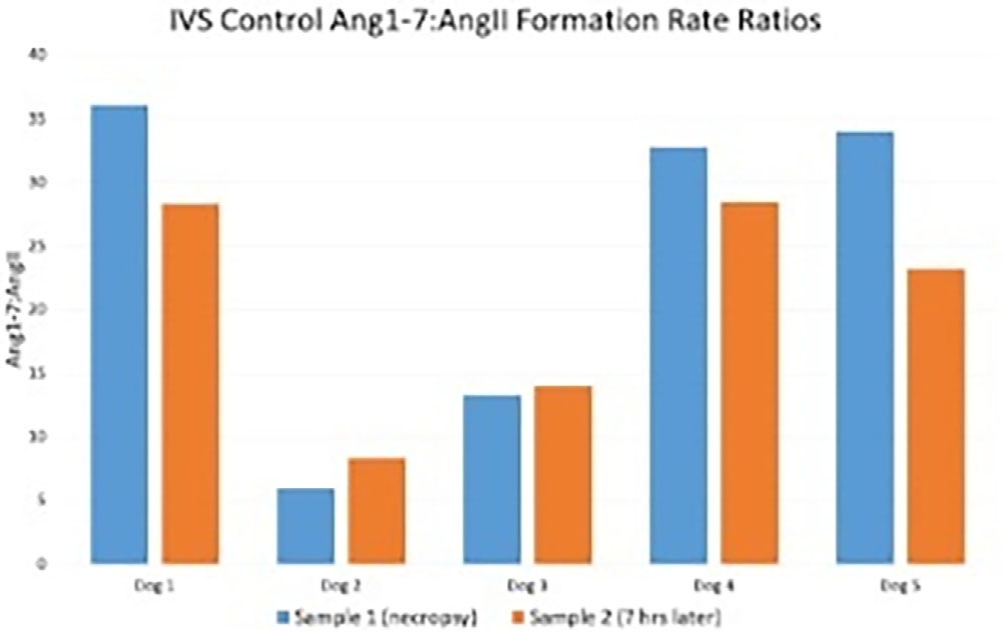
**Figure 1** Ratio of tissue production of angiotensin 1‐7 (Ang1‐7) to angiotensin II (AngII) in the interventricular septum (IVS) in five dogs.

**Conclusions and Clinical Importance:** Renin‐angiotensin system enzymes remain active for hours post‐mortem. The myocardial Ang1‐7:AngII formation rate was relatively stable over time.

## 278 Abstract C34

### 278.1 Cardiovascular Abnormalities in Dogs with Acute Pancreatitis

#### 278.1.1 **Harry Cridge** ^1^; Daniel Langlois^2^, DVM, DACVIM (SAIM); Robert Sanders^3^, DVM, MS, DACVIM (Cardiology)

##### 278.1.1.1 ^1^Michigan State University; ^2^Assistant Professor, College of Veterinary Medicine, Michigan State University; ^3^Associate Professor, College of Veterinary Medicine, Michigan State University

**Background:** Cardiovascular complications of acute pancreatitis (AP) are well documented in humans; however, limited information is published about dogs.

**Hypothesis/Objectives:** To evaluate the incidence of cardiac abnormalities in dogs with AP, and to determine whether they correlate with AP severity.

**Animals:** 13 dogs with AP.

**Methods:** Prospective observational study. Diagnosis of AP was based on history, elevated pancreatic lipase concentration, and ≥2 ultrasonographic features of AP. Dogs had an echocardiogram performed and a Holter monitor placed. Cardiac troponin I (hs‐cTnI) and N‐terminal pro‐B‐type natriuretic peptide (NT‐proBNP) concentrations were measured. Disease severity was determined by the canine acute pancreatitis severity (CAPS) score. Biomarker concentrations were compared between dogs with and without a CAPS score ≥11 using Mann‐Whitney U tests. Spearman's rank correlation coefficients (Rs) were used to investigate associations between CAPS scores and arrhythmias.

**Results:** 91.7% and 58.3% of dogs with AP had elevated hs‐cTnI (mean: 0.582±0.824, RI: ≤0.06 ng/ml) and NT‐proBNP concentrations (mean: 1027±641, RI: 0–900 pmol/L), respectively. Cardiac troponin I (*P*=0.76) and NT‐proBNP concentration (*P*=0.41) were not associated with CAPS score. Mean supraventricular and ventricular burdens were low at 0.79±1.11% and 0.495±1.26%, respectively. Supraventricular (Rs=0.167, *P*=0.58) and ventricular burdens (Rs=‐0.271, *P*=0.37) were not associated with CAPS scores. Echocardiographic abnormalities were uncommon.

**Conclusions and Importance:** Myocardial injury is common in dogs with AP, but clinically relevant disease appears to be uncommon. Cardiac biomarkers must be interpreted with caution in dogs with AP.

## 279 Abstract C35

### 279.1 Chronic Myxomatous Valve Disease Prevalence and Pathologic Features—Canine Mitral, Tricuspid, Aortic, and Pulmonic Valves

#### 279.1.1 **Hunter L. Enderle** ^1^; Taryn Donovan^2^, DVM, DACVP; Philip Fox^3^, DVM, DACVIM/DECVIM‐Cardiology, DACVECC

##### 279.1.1.1 ^1^Schwartzman Animal Medical Center; ^2^Pathologist, Pathology, Schwartzman Animal Medical Center; ^3^Cardiologist, Cardiology, Schwartzman Animal Medical Center

**Background:** Chronic myxomatous valve disease (CMVD) is the leading cause of canine cardiac morbidity. Yet, contemporary information detailing related valve pathology is scarce.

**Hypothesis/Objectives:** Identify CMVD prevalence, gross pathologic features, severity across age, breed, and valves.

**Animals:** 67 randomly selected hearts archived between 2000–2021.

**Methods:** Mitral (MV), tricuspid (TV), aortic (Ao) and pulmonic (PV) valves were prospectively examined visually and with stereomicroscopy when required. We developed and applied a modified Pomerance‐Whitney severity grading scheme. Based upon gross valve morphology: Pattern‐I, small, discrete, non‐coalescing, myxomatous opacities within proximal leaflet cusps; Pattern‐II, larger, nodular, valvular densities with some coalescing; thickened leaflet edges; Pattern‐III, widely coalescing densities creating diffuse valve thickening and severe deformation (for MV‐occasional, proximal chordae tendineae thickening/rupture).

**Results:** CMVD prevalence: MV‐98.5%, TV‐85.1%; Ao‐29.9%; PV‐6%. CMVD was identified in all small‐to‐giant breeds in this study (median age‐9 years, range‐0.5–17 years; median weight‐12.5 kg, range‐2.2–82.2 kg; 64% males). Lesion severity was mild (pattern‐I)‐ 8/67 (11.9%; 1–10 years; median‐7.5 years; MV only in 3, MV+TV in 5); moderate (pattern‐II)‐ 16/67 (23.9%; 0.5–12 years; median‐8.5 years; MV only‐4/16; MV+TV‐12/16); severe (pattern‐III)‐ 39/67 (58.2%; 0.6–14 years; median‐7.5 years; MV+TV 39/39 with MV >TV in 15, TV >MV in 3, MV=TV in 21). CMVD was mild‐to‐moderate in Ao (29.9%; pattern‐I, 12/67; pattern‐II, 8/67), and rare in pulmonic valves (6%; pattern‐1, 4/67).

**Conclusions and Clinical Importance:** CMVD occurred in all breeds across all ages studied. TV involvement was higher than previously reported.

## 280 Abstract C36

### 280.1 International Evaluation of Clinical Characteristics and Outcomes in 137 Dogs with Reverse Patent Ductus Arteriosus

#### 280.1.1 **Logan L. Funk** ^1^; Stacey Leach^2^, DACVIM (Cardiology); Loren Schultz^3^, DVM

##### 280.1.1.1 ^1^University of Missouri; ^2^Associate Teaching Professor, Cardiology, University of Missouri; ^3^Associate Teaching Professor, Public Health, University of Missouri

**Background:** Left‐to‐right shunting patent ductus arteriosus (PDA) consistently ranks among the most common congenital cardiac anomalies in dogs. Conversely, right‐to‐left shunting PDA (rPDA), a recognized cause of cyanotic heart disease (CHD), is apparently rare. To date, there is no large‐scale data regarding rPDA.

**Hypothesis/Objectives:** The objective of this multi‐center, retrospective study was to describe the signalment, clinical signs, diagnostic findings, and outcomes of dogs with rPDA.

**Animals:** One hundred and thirty‐eight client‐owned dogs with rPDA.

**Methods:** Medical records from 20 institutions world‐wide were retrospectively reviewed. Dogs were included if they were diagnosed with rPDA by a cardiologist. Dogs with other forms of CHD were excluded. Signalment, clinical findings, treatments, incidence of right‐sided congestive heart failure (RCHF), and outcomes were recorded.

**Results:** Most dogs were female (63.8%), and the median (range) age at diagnosis was 29 (1–142) months. Clinical signs were reported in 92% of dogs and included hindlimb weakness (41%), differential cyanosis (35%), and syncope (28%). The mean (±SD) maximum packed cell volume was 67 (±12)%, and RCHF was diagnosed in 17% of dogs. Therapy included phosphodiesterase‐5 inhibitors (68%), phlebotomy (41%), and hydroxyurea (15%). Dogs that received phosphodiesterase‐5 inhibitors lived longer (p=0.015) than those that did not, with median (range) lifespans of 90.2(1.6–181.4) months versus 28.8 (3.2–173.6) months, respectively.

**Conclusions and Clinical Importance:** Most dogs exhibited clinical signs at a young age, while a minority developed RCHF. Phosphodiesterase‐5 inhibitors were the most common therapy and yielded significant prolongation of median survival time.

**Survival analysis**

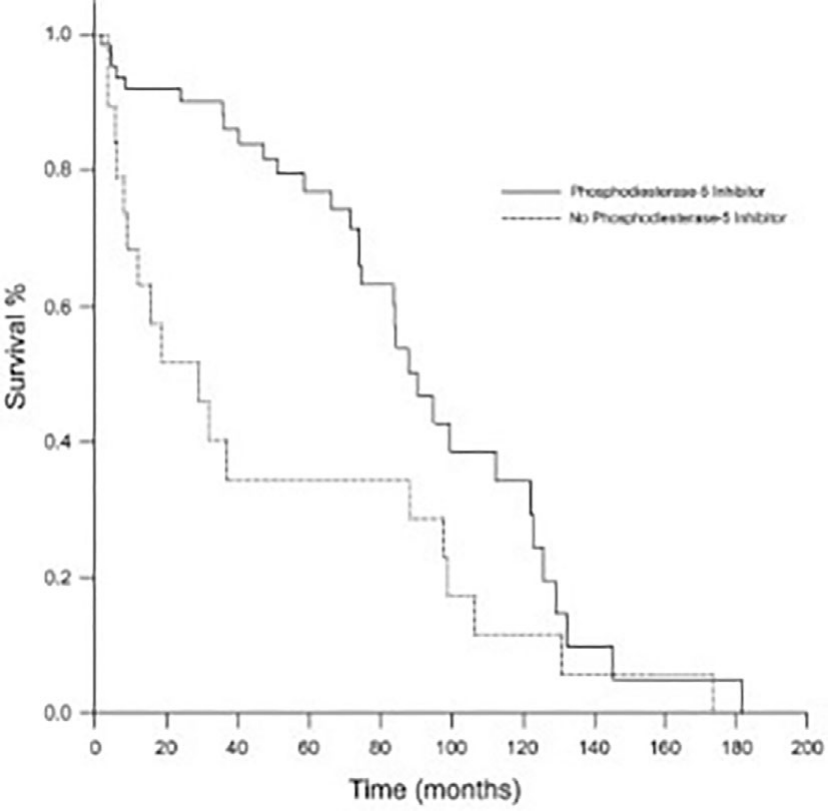

## 281 Abstract C37

### 281.1 Multivariate Statistical Analysis in the Screening of Subclinical Hypertrophic Cardiomyopathy Phenotype in Domestic Cats

#### 281.1.1 **Fabio N. Gava** ^1^; Ana Paula Maingue^2^; Mariana Podleskis^2^; Patrick Luz^2^; Roberto Ampuero^3^; Patricia Pereira^2^; Lucas Gomes^2^

##### 281.1.1.1 ^1^Department of Veterinary Clinics, Londrina State University; ^2^Londrina State University; ^3^Jaboticabal, São Paulo

**Background:** Hypertrophic cardiomyopathy (HCM) is the most frequent heart disease in cats and screening studies all over the world are important, as well as the echocardiographic methodology used.

**Hypothesis/Objectives:** Echocardiography was conducted in domestic cats in the south of Brazil and multivariate statistical analysis was performed to illustrate mathematically if measurements of interventricular septum (IVS) and left ventricle free wall obtained by B‐mode are better than M‐mode for the identification of the HCM phenotype.

**Animals:** One hundred asymptomatic cats were recruited and 92 allowed echocardiography.

**Methods:** Echocardiographic measurements were submitted to multivariate statistical analysis; blood pressure measurement and serum total T4 concentration data were submitted to univariate statistical analysis.

**Results:** A frequency of 30.44% of the HCM phenotype was observed. The discriminant analysis showed that diastolic IVS and free wall through B‐mode evaluation had a 78,57% hit while M‐mode had only 17,85% hit for the HCM phenotype identification. The cluster analysis showed larger linkage distance between normal and HCM cats using B‐mode measurements. The correspondence analysis showed higher frequency of normal diastolic function and fused E and A waves in normal cats and higher frequency of diastolic dysfunction in HCM cats, but did not reach statistical importance to differentiate them. Hypertension and hyperthyroidism were not cause of HCM phenotype in this study.

**Conclusions and Clinical Importance:** The frequency of HCM phenotype found was high and the multivariate statistical analysis showed mathematically that B‐mode measurements are better to identify the HCM phenotype in domestic cats.

## 282 Abstract C38

### 282.1 Hypertensive Cardiomyopathy in Dogs: Echocardiography and Sex Differences

#### 282.1.1 **Fabio N. Gava** ^1^; Geovanna Benedito^2^; Carolina Milhorine^2^; Karina Marques da Costa Flaiban2

##### 282.1.1.1 ^1^Department of Veterinary Clinics, Londrina State University; ^2^Londrina State University

**Background:** Chronic kidney disease (CKD) is the main cause of systemic arterial hypertension (SAH) in dogs and can lead to hypertensive cardiomyopathy (HC). Sex differences in the HC are still unclear in dogs.

**Hypotheses/Objectives:** To describe the echocardiographic changes in SAH secondary to CKD and sex differences.

**Animals:** Forty‐one dogs, from clinical care, constituted the following groups: CG (control, n=22); GHAS (hypertensive, n=19) and the subgroups: GC‐M (control, males, n=10); GHAS‐M (hypertensive, males n=9); GC‐F (control, females, n=12); GHAS‐F (hypertensive, females, n=10); GC‐FC (control, spayed females, n=6); GC‐FNC (control, non‐spayed females, n=6); GHAS‐FC (hypertensive, spayed females, n=5); GHAS‐FNC (hypertensive, non‐spayed females, n=5).

**Methods:** Measurement of systolic arterial pressure (SAP), laboratory tests and echocardiography were performed on the same day. The anatomical measurements were normalized by weight.

**Results:** Hypertensive dogs presented higher diastolic values (cm) of interventricular septum (IVSd) and left ventricular free wall (LVFWd) than healthy dogs (IVSd: GC: 0.52±0.01; GHAS: 0.63±0.03; P=0.0023) (LVFWd: GC: 0.48±0.01; GHAS: 0.60±0.03; P<0.0001). Hypertensive male dogs presented higher IVSd and LVFWd values than females (IVSd: GHAS‐M: 0.70±0.04; GHAS‐F: 0.57±0.04; P<0.05) (LVFWd: GHAS‐M: 0.68±0.03; GHAS‐F: 0.54±0.03; P<0.01). There were no differences between control and hypertensive females, neither between spayed and non‐spayed hypertensive females. There was a positive correlation between SAP and LVFWd.

**Conclusions:** Hypertensive cardiomyopathy was observed in males but not in females; castration does not interfere in the cardioprotection observed in females; there is a correlation between SAP and cardiac remodeling in dogs.

## 283 Abstract C39

### 283.1 Mitral Annular Plane Systolic Excursion in Dogs with Systemic Arterial Hypertension

#### 283.1.1 **Fabio N. Gava** ^1^; Geovanna Benedito^2^

##### 283.1.1.1 ^1^Department of Veterinary Clinics, Londrina State University; ^2^Londrina State University

**Background:** Chronic kidney disease (CKD) is commonly associated with systemic arterial hypertension (SAH) and can lead to concentric ventricular hypertrophy. It is known that longitudinal fibers are more susceptible to fibrosis compared to radial fibers, demonstrating the importance of longitudinal systolic function evaluation in hypertensive dogs.

**Hypothesis/Objectives:** To evaluate the mitral annulus systolic excursion (MAPSE) in dogs with SAH secondary to CKD.

**Animals:** Thirty‐seven dogs, allocated in two groups: GC: control (healthy, n=20); GSAH: hypertensive dogs secondary to CKD (n=17).

**Methods:** Echocardiography and systolic blood pressure (SBP, Doppler method) were performed on the same day. The MAPSE was obtained from the left parasternal window, in the apical four‐chamber view, positioning the M‐mode cursor on the septal mitral annulus. Measurements were performed in triplicate and the values were normalized by body weight (mm/kg). Patients with other diseases or B2, C and D stages of mitral valve disease were excluded.

**Results:** SBP values (mm Hg) were higher in patients with SAH (GC: 136.0±3.0; GSAH: 205.3±6.7; P<0.0001). Hypertensive patients had MAPSE values significantly decreased (GC: 1.10±0.14; GSAH: 0.70±0.08; P=0.04) and there was a significant correlation between the SBP values and MAPSE (P=0.008, r=‐0.42).

**Conclusions and Clinical Importance:** Dogs with SAH secondary to CKD have reduction in MAPSE, which worsens with the increase of systolic blood pressure. This echocardiographic measurement may be used in the investigation of hypertensive cardiomyopathy in dogs.

## 284 Abstract C40

### 284.1 QT Interval, Electrolyte and Acid‐Base Evaluation in Dogs with Chronic Kidney Disease

#### 284.1.1 **Fabio N. Gava** ^1^; Geovanna Benedito^2^; Gabriela Arias^2^; Carolina Milhorine^2^; Priscila Valente Pereira^2^; Karina Marques da Costa Flaiban^2^

##### 284.1.1.1 ^1^Department of Veterinary Clinics, Londrina State University; ^2^Londrina State University

**Background:** Chronic kidney disease (CKD) leads to electrolyte and acid‐base disturbances, which can cause electrocardiographic changes, especially related to ventricular repolarization.

**Hypothesis/Objectives:** To characterize electrocardiographic and blood gas analysis in dogs with CKD stages 3 and 4.

**Animals:** Twenty‐eight dogs, allocated into three groups: GC: Control (healthy, n=13); G‐CKD3: CKD stage 3 (n=5) and G‐CKD4: CKD stage 4 (n=10).

**Methods:** Venous blood gas analysis and electrocardiography were performed at the same time.

**Results:** QT interval (ms) was prolonged in CKD stage 4 (GC: 189.7±3.1; G‐CKD3: 206.8±5.3; G‐CKD4: 241.7±8.3; P<0.001), with no difference in QRS complex duration. There was a decrease in pH between GC vs. G‐CKD3 and GC vs. G‐CKD4 (GC: 7.379±0.01; G‐CKD3: 7.301±0.03; G‐CKD4: 7.283±0.02; P<0.01); excess bases (BE, mmol/L) (GC: ‐2.82±0.75; G‐CKD3: ‐8.88±3.37; G‐CKD4: ‐11.45±1.51) and bicarbonate (HCO_3_, mmol/L) (GC: 21.69±0.45; G‐CKD3: 17.08±1.97; G‐CKD4: 15.62±1.09; P<0.001). The Strong Ion Difference (SID) had no changes (GC: 39.51±0.51; G‐CKD3: 37.65±3.75; G‐CKD4: 44.80±6.70). Anion gap (mEq/L) was increased in G‐CKD4 (GC: 16.52±1.15; G‐CKD3: 20.64±4.45; G‐CKD4: 25.28±3.57; P<0.05). No differences for sodium and chloride, but for potassium and ionized calcium between GC vs. G‐CKD4, however, within normal values for dogs. Phosphate was increased in G‐CKD3 and G‐CKD4, with difference between GC vs. G‐CKD4. Moderate negative correlation was observed between QT interval and pH, BE and HCO_3_.

**Conclusions and Clinical Importance:** Metabolic acidosis in dogs with CKD is multifactorial and responsible for QT interval prolongation, mainly in stage 4.

## 285 Abstract C41

### 285.1 Validation of a Point‐of‐Care Quantitative Assay for Feline NT‐proBNP

#### 285.1.1 **Emily Javery** ^1^; Ryan Fries^2^, DVM, DACVIM (Cardiology); Lindsey Humphries^3^, DVM, MS; Saki Kadotani^4^, DVM, DACVIM (Cardiology); Leah Kruckman^3^, DVM; Sumana Prabhakar^3^, DVM; Michael Rosser^4^, DVM, MS, DACVP (Clinical Pathology)

##### 285.1.1.1 ^1^University of Illinois at Urbana‐Champaign; ^2^Assistant Professor, Veterinary Clinical Medicine, University of Illinois at Urbana‐Champaign; ^3^Cardiology Resident, Veterinary Clinical Medicine, University of Illinois at Urbana‐Champaign; ^4^Clinical Assistant Professor, Veterinary Clinical Medicine, University of Illinois at Urbana‐Champaign

**Background:** Point‐of‐care testing (POC) is widely utilized for rapid results for many different analytes. A new feline‐specific N‐terminal pro‐Brain natriuretic peptide (NT‐proBNP) quantitative assay (Vcheck V200, Bionote Inc.) is currently available but has not undergone independent validation.

**Hypothesis/Objectives:** To validate the Vcheck POC quantitative assay for feline cardiac NT‐proBNP.

**Animals:** Serum samples from 62 cats.

**Methods:** Validation was performed in accordance with the American Society for Veterinary Clinical Pathology guidelines. Precision was determined for low (50–100 pmol/L), mid (101–300 pmol/L), and high (>301 pmol/L) pools within‐day (short‐term, 15 repetitions) and within‐week (long‐term, 5 repetitions each day). Linearity was used to assess accuracy. Bias was determined from paired serum samples (n=49) submitted to IDEXX Laboratories.

**Results:** The within‐day median values for NT‐proBNP were low=86.75 pmol/L, mid=102 pmol/L and high=392.4 pmol/L and the within‐week median values were low=88.4 pmol/L, mid=200.3 pmol/L and high=361 pmol/L. The within‐day coefficients of variability (CV) were low=12.59%, mid=10.37%, and high=8.69%. The within‐week CV were low=9.99%, mid=14.92%, and high=6.89%. Accuracy of the assay determined by linearity was 107%. Paired samples between IDEXX and Vcheck assays demonstrated a bias of 2.2% and an r^2^=0.95 with no statistical difference between either assay (P=0.375).

**Conclusions and Clinical Importance:** The Vcheck V200 has acceptable precision, accuracy, and bias and is as a viable POC quantitative assay for feline NT‐proBNP.

## 286 Abstract C42

### 286.1 Cartilage Intermediate Layer Protein 1 as a Novel Biomarker for Canine Myxomatous Mitral Valve Degeneration

#### 286.1.1 **Hyeon‐Jin Kim** ^1^; Ha‐Jung Kim^2^, DVM, PhD; Soomin Kim^1^; Ji‐Hye Lee^1^; Yoonji Kim^1^; Ji‐Hee Kim^1^; Jihyun Kim^1^; Yeji Kim^1^; Yunji Song^1^

##### 286.1.1.1 ^1^Chonnam National University; ^2^Professor, Veterinary Medicine, Chonnam National University

**Abstract:** Cartilage intermediate layer protein 1 reflects cardiac remodeling in canine myxomatous mitral valve degeneration

**Background:** Serum cartilage intermediate layer protein 1 (CILP1) level reflects severity of cardiac remodeling in canine myxomatous mitral valve degeneration (MMVD).

**Hypothesis/Objectives:** The aim of this study was to determine whether CILP1 could be used as a biomarker of canine MMVD.

**Animals:** Twenty‐seven client‐owned dogs diagnosed with MMVD were enrolled as the patient group. Four beagles and four client‐owned dogs for more than seven years were enrolled as healthy controls.

**Methods:** Serum CILP1 level and cardiac remodeling values were measured for the whole cohort. Serum CILP1 level was compared with conventional cardiac biomarkers and clinical values of MMVD. Data analysis was performed employing the Kruskal‐Wallis test, Dunn's multiple comparison test, Spearman's correlation analysis, and receiver operating characteristic curve. Statistical significance was set at P<0.05.

**Results:** Serum CILP1 level of the patient group was higher than that in the healthy control group (P=0.0283, 95% confidence level: ‐0.01653 to 1.112). Simple linear regression of cardiac remodeling values and CILP1 demonstrated a correlation. The whole cohort was classified according to the serum CILP1 cut‐off value. Left ventricular end diastolic diameter normalized against body weight showed the most significant P value (P=0.0005, 95% confidence level: 1.815 to 5.751).

**Conclusions and Clinical Importance:** Serum CILP1 level reflects cardiac remodeling in canine MMVD patients. CILP1 can be a potential biomarker of canine MMVD. This is the first study about CILP1 in veterinary medicine.

**Image 1**

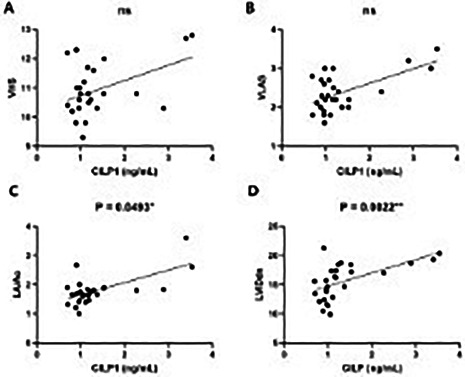

**Image 2**

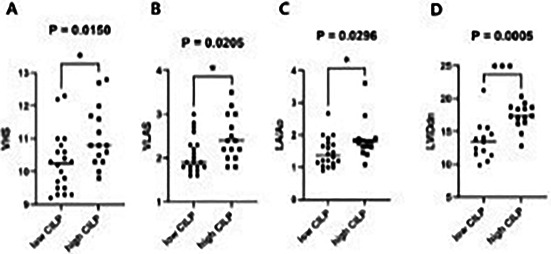

## 287 Abstract C43

### 287.1 Intrarenal Venous Flow Analysis by Ultrasound in Dogs with Heart Disease

#### 287.1.1 **Tomoya Morita**; Hiroki Terukina; Masahiro Yamasaki

##### 287.1.1.1 Iwate University

**Background:** In humans, cardiorenal syndrome is important in the pathophysiology of heart failure. Renal congestion due to decreased right ventricular (RV) function and increased right atrial (RA) pressure is the main factor underlying cardiorenal syndrome. Intrarenal venous flow (IRVF) analysis is used to evaluate renal congestion in human patients with heart failure. Venous impedance index (VII) reflecting venous compliance is elevated, and discontinuous IRVF pattern is strongly associated with worsening prognosis in heart failure patients. However, there is no study that analyzed IRVF in dogs. This study investigated the factors affecting IRVF and the characteristics of dogs with discontinuous IRVF pattern.

**Methods:** Seventy‐one dogs without heart disease and forty‐six dogs with heart disease (33 myxomatous mitral valve disease; 12 pulmonary hypertension; 1 filariasis) were prospectively enrolled. IRVF analysis was performed in the right lateral decubitus position. Sample volume was set at interlobar veins. VII was calculated as follows: (maximum velocity − minimum velocity)/maximum velocity. Doppler waveforms of IRVF were divided into continuous and discontinuous patterns. Discontinuous pattern was defined as a pattern in which the minimum velocity was zero.

**Results:** All dogs without heart disease were subdivided into continuous pattern. Dogs with heart disease were subdivided continuous (n=38, 82.6%) and discontinuous (n=8, 17.4%) pattern. The discontinuous pattern group had a higher tricuspid regurgitation velocity, larger RV and RA size, and an impaired RV function. RA area (r=0.83) and RV fractional area change (r= −0.61) correlated with VII.

**Conclusions:** IRVF was affected by RV function and RA pressure.

## 288 Abstract C44

### 288.1 High Prevalence and Clinical Features of Mitral Regurgitation in Young Chihuahuas Without Heart Murmurs

#### 288.1.1 **Seiya Niimi** ^1^; Haruna Kobayashi^2^; Yukika Take^1^; Shiori Ikoma^1^; Saki Namikawa^1^; Yoko Fujii^1^

##### 288.1.1.1 ^1^Azabu University; ^2^Primo Animal Hospital

**Background:** Chihuahuas are predisposed to myxomatous mitral valve disease (MMVD); however, the clinical features associated with the disease onset have not been investigated.

**Objectives:** This study was conducted to identify the prevalence and characteristic features of mitral regurgitation in young Chihuahuas in the absence of heart murmur.

**Animals:** We included thirty Chihuahuas privately owned by breeders. Chihuahuas were apparently healthy, <7 years old, and with no heart murmur.

**Methods:** Echocardiography was performed to evaluate mitral valve thickening, prolapse, and the regurgitation area in addition to routine measurements (e.g., M‐mode parameters, left atrial to aortic diameter ratio). The timing of mitral regurgitation onset was also evaluated.

**Results:** Regurgitation, thickening, and prolapse of the mitral valve were observed in 26/30 (86.7%), 17/30 (56.7%), and 14/30 (46.7%) dogs, respectively. The prevalence of these findings increased with age. All dogs aged over four years exhibited mitral valve regurgitation, thickening, and prolapse. The timing of mitral regurgitation onset in younger Chihuahuas without the pathological changes associated with mitral valves (thickening or prolapse) was late systole (after the second half of systole). Routine measurements in all dogs were within reference ranges.

**Conclusions and Clinical Importance:** Young Chihuahuas without heart murmur had a high prevalence of mitral regurgitation. Interestingly, the late onset of mitral regurgitation without pathological changes of the valve was a unique feature in this breed. This may be related to the development of MMVD in this breed; therefore, this hypothesis requires further investigation.

## 289 Abstract C45

### 289.1 Plasma Serotonin, Endothelin, and VEGF‐D: A Differential Marker of Pre and Postcapillary Pulmonary Hypertension

#### 289.1.1 **Dmitrii Oleynikov;** Belij Klyk

289.1.1.1

**Abstract:** Pulmonary hypertension (PH) in dogs is a syndrome that could be primary or secondary. Due to the inability of routine right heart catheterization, there is a lack of differential criteria between PH forms. Thus, circulating markers could be useful to find out the mechanism of pulmonary hypertension.

Following all previous data in studies, we supposed that circulating serotonin, endothelin‐1, and VEGF‐D would show a predominance of affected part of pulmonary circulation.

We studied 59 small‐breed dogs. Groups were formed: healthy dogs (HD, n=8); dogs with MMVD and postcapillary PH (PostPH, n=23); dogs with MMVD and precapillary PH (PrePH, n=28). Animals were diagnosed by standard algorithms. Blood samples were collected at the moment of presentation and frozen in ‐80°C fridge. For biochemistry analysis. We used species‐specific ELISA kits, provided by Cloud‐Clone Corp. (USA).

PostPH dogs had a higher VEGF‐D concentration in comparison to control and PrePH (р<0.001, for both). Endothelin‐1 was higher in PrePH in comparison to PostPH and controls (р<0.001, for both). Serotonin concertation was higher in PrePH than in the control (р<0.033) and postcapillary PH group (р<0.006). ROC‐analysis showed that plasma concentrations of Endothelin‐1 (0.99) and VEGF‐D (0.92) had a high differentiation value for PH forms.

**Conclusion:** This study showed a correlation between circulating biomarkers (serotonin, endothelin‐1, and VEGF‐D). We found a connection between endothelin‐1 and right‐sided heart failure and VEGF‐D and left heart failure in the PH context.

**Table:** Table 1. Biomarkers concentrations in PH groups

Indices	Control (N=8)	Precapillary PH (N=28)	Postcapillary PH (N=23)	Differences between Control and PrePH	Differences between Control and PostPH	Differences between PrePH and PostPH
VEGF D (pg/ml)	33.1 (29.7–36.9)	36.0 (26.1–59.8)	81.2 (73.3–96.2)	>0.05	<0.001	<0.001
Endothelin‐1 (pg/ml)	17.8 (15.0–19.2)	36.6 (33.1–39.0)	20.6 (17.2–23.1)	<0.001	>0.05	<0.001
Serotonin (ng/ml)	26.1 (21.0–30.7)	30.2 (26.1–67.9)	26.6 (22.4‐30.5)	0.033	>0.05	0.006

## 290 Abstract C46

### 290.1 Prognostic Value of Left Atrial Stiffness Estimated Using Echocardiography in Canine Myxomatous Mitral Valve Disease

#### 290.1.1 **Tatsuyuki Osuga**; Kyoko Kuroda; Tomoya Morita; Kensuke Nakamura; Noboru Sasaki; Mitsuyoshi Takiguchi

##### 290.1.1.1 Hokkaido University

**Background:** Left atrial (LA) function, consisting of reservoir, conduit, and booster pump functions, plays a pivotal role in modulating left ventricular filling. There are conflicting reports about the prognostic usefulness of echocardiographic evaluation of LA function in dogs with myxomatous mitral valve disease (MMVD). Left atrial stiffness (LASt) is an important determinant of LA reservoir function. In humans, LASt estimated using echocardiography is a more useful prognostic indicator than LA reservoir function assessed in terms of LA reservoir strain (εS).

**Hypothesis:** Increased LASt estimated by echocardiography is associated with shortened survival in dogs with MMVD.

**Animals:** Seventy‐two dogs with MMVD.

**Methods:** Retrospective cohort study. Dogs underwent echocardiographic examinations at enrollment. Peak velocities of transmitral early diastolic flow (E) and early diastolic myocardial velocity (E') were determined. εS was determined by using two‐dimensional speckle tracking echocardiography of LA. LASt was estimated by the formula: (E/E')/ εS.

**Results:** Dogs with increased LASt (LASt >0.56; median survival time, 484 days; 95% confidence interval, 283 days–indeterminable) had a shorter (P<0.001) survival time than those without increased LASt (LASt ≤0.56; median survival time, >1112 days; 95% confidence interval, indeterminable). Multivariate Cox proportional hazard analysis demonstrated that the left atrial to aortic ratio and LASt were independent predictors of cardiac‐related death among heart rate, conventional echocardiographic indices, εS, and LASt.

**Conclusions and Clinical Importance:** Increased LASt estimated using echocardiography is associated with reduced survival times of dogs with MMVD. LASt estimated by echocardiography might be a more useful prognostic indicator than εS.

## 291 Abstract C48

### 291.1 Clinical Outcome of Idiopathic Juvenile Ventricular Arrhythmias in 25 Dogs

#### 291.1.1 **Anna A. Reuter** ^1^; Teresa DeFrancesco^2^, DVM, DACVIM (Cardiology), DACVECC; Kathryn Meurs^3^, DVM, PhD, DACVIM (Cardiology)

##### 291.1.1.1 ^1^Purdue University Veterinary Teaching Hospital; ^2^Professor, Cardiology, College of Veterinary Medicine, North Carolina State University; ^3^Professor, Cardiology, Associate Dean for Research and Graduate Studies, College of Veterinary Medicine, North Carolina State University

**Background:** Juvenile ventricular arrhythmias (JVA) have been characterized in a number of canine breeds with limited long‐term data.

**Objectives:** Describe the clinical outcome of JVA in a variety of dog breeds.

**Animals:** Twenty‐five dogs with a diagnosis of JVA at a university hospital.

**Methods:** A retrospective case series of dogs under two years of age with ventricular ectopy from 2003 until 2020. Cases were included in the absence of structural heart disease, systemic illness, and with normal troponin (if performed). Holter monitor data was evaluated for ventricular beat number and complexity at the time of diagnosis and over time. Long term follow‐up was achieved through client and primary veterinarian contact.

**Results:** Of the initial 154 dogs identified, 25 were included in the study. Breeds included German shepherd (8), boxer (4), Great Dane (3), mixed breed (3), and one each of the following: Anatolian shepherd, French bulldog, golden retriever, Great Pyrenees, Labrador retriever, Shiloh shepherd, and Siberian husky. The average age at diagnosis was 7.9 months (range, 2–22 months). The overall median survival was 10.96 years (range, 1.75–15.66 years). There was an average reduction in the number of ventricular beats by 86.7% per year (p value = 0.0257) based on Holter data.

**Conclusion:** Idiopathic JVA had a favorable long term prognosis with reduced ectopy over time in this case series. JVA remains a diagnosis of exclusion but can be considered in a broader range of dog breeds than previously described.

**Overall survival**

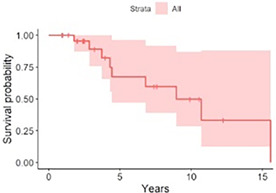

## 292 Abstract C49

### 292.1 Echocardiographic Comparison of Right Ventricular Function Between Dogs with Pre‐ and Post‐capillary Pulmonary Hypertension

#### 292.1.1 **Ryohei Suzuki** ^1^; Yunosuke Yuchi^2^, DVM; Takahiro Teshima^3^, DVM, PhD; Hirotaka Matsumoto^3^, DVM, PhD; Hidekazu Koyama^4^, DVM, PhD

##### 292.1.1.1 ^1^Nippon Veterinary and Life Science University; ^2^Graduate student, Veterinary Medicine, Nippon Veterinary and Life Science University; ^3^Associate Professor, Veterinary Medicine, Nippon Veterinary and Life Science University; ^4^Professor, Veterinary Medicine, Nippon Veterinary and Life Science University

**Background:** Pulmonary hypertension (PH) is classified into two subtypes according to the cause of PH: pre‐capillary and post‐capillary PH. However, few studies have compared the right ventricular (RV) function between the pre‐ and post‐capillary PH.

**Hypothesis/Objectives:** To compare the RV morphology and function in dogs with PH due to various causes.

**Animals:** Twenty‐five dogs with pre‐capillary PH and thirty‐five dogs with post‐capillary PH.

**Methods:** Prospective cross‐sectional study. Post‐capillary PH were classified by pulmonary vascular resistance estimated by echocardiography (PVRecho): isolated post‐capillary PH (Ipc‐PH) defined as PVRecho <0.60, combined post‐ and pre‐capillary PH (Cpc‐PH) defined as PVRecho ≥0.60. All dogs underwent echocardiography including two‐dimensional speckle‐tracking echocardiography.

**Results:** Eleven dogs with pre‐capillary PH (44%), one of twenty‐one dogs with Ipc‐PH (5%), and nine of fourteen dogs with Cpc‐PH (64%) showed right heart failure. The RV internal dimension and area were significantly higher, and RV fractional area change and RV strain were lower in dogs with Cpc‐PH and pre‐capillary PH than Ipc‐PH (all *P*<0.05). No significant differences in RV morphology and function were found between Cpc‐PH and pre‐capillary PH.

**Conclusions and Clinical Importance:** Pre‐capillary PH showed more deteriorated RV function than Ipc‐PH. Our results suggest that pre‐capillary PH might be more serious than post‐capillary PH, possibly reflecting the higher RV afterload and lack of ventricular interdependence associated with left heart disease. Additionally, in dogs with post‐capillary PH, progression to Cpc‐PH might worsen RV morphology and function as the same extent as pre‐capillary PH.

## 293 Abstract C50

### 293.1 Up‐regulation of STATs and IRF‐1 in Canines with Heart Murmur and Associated Valvular Cardiovascular Disorder

#### 293.1.1 **Selena K. Tavener**; Rachel Rusk, MS; Becky Stone, DVM; Kiran Panickar, PhD

##### 293.1.1.1 Science & Technology Ctr, Hill's PNC

**Background:** Myxomatous mitral valve disease (MMVD) is a commonly diagnosed canine heart disease that affects all breeds. Immune activation of transcription factors including STAT and IRF are key mediators of inflammation and have been implicated in cardiovascular disorders.

**Hypothesis:** Increased expression of pro‐inflammatory signaling pathways contributes to cardiovascular disorders in canines.

**Animals:** Canines housed in Hill's animal colony and diagnosed with heart murmur (n=6; 4 dogs‐grade 5/6 and 2 dogs‐grade 4/6; 9.3–15.11 yr) by a veterinarian and controls (n=6; 9.4–15.10 yr) with no history of cardiovascular problems.

**Methods:** Total circulating RNA was used to assess gene expression using NanoString nCounter® platform and analyzed using the nSolver software.

**Results:** When comparing dogs diagnosed with heart murmur to controls, there was a significant up‐regulation of the transcription factors STAT‐1 and IRF‐1, as well as IL‐35 and TGFBR1 (p<0.05). There was also an increase in STAT‐2, CCR1, IL‐4, TGFB1, and TNFSF10 (all ns). Significant down‐regulation was reported in CD19, CD21 and BCL10 (p<0.05).

**Conclusions and Clinical Importance:** STAT‐1 and IRF‐1 induce interferon‐stimulated genes and subsequent inflammation. CD19, a receptor on B‐cells, forms a complex with CD21, a receptor for the cleavage products for complement C3, plays an important role in the amplification of B‐cell responses. While a decrease in CD19 and CD21 in our study indicates a possible disruption in the homeostasis of B‐cell activation, its role in MMVD is unclear. Our results may serve as important targets for reducing inflammation‐associated signaling pathways in canines with cardiovascular valve disorders.

## 294 Abstract C51

### 294.1 Serum Carnitine Profile of Cats with Hypertrophic Cardiomyopathy

#### 294.1.1 **Mutsuki Umezawa**; Shun Sunaga; Seiya Niimi, DVM; Takuma Aoki, DVM, PhD; Shinpei Kawarai; Yoko Fujii, DVM, PhD, DACVIM (Cardiology)

##### 294.1.1.1 Azabu University

**Background:** The carnitine profile has been recognized as an indicator of myocardial fatty acid metabolism that is modified in various cardiac diseases.

**Objectives:** This study assessed the carnitine profile of cats diagnosed with various stages of hypertrophic cardiomyopathy (HCM) and compared it with that of healthy cats.

**Animals:** Cats with HCM and with stored frozen samples were retrospectively recruited from the medical facility of the Azabu University veterinary teaching hospital. The cats were classified into stages (Stage B1, B2, and C/D) in accordance with the guideline for feline HCM.

**Methods:** Total and free carnitine levels were measured by the enzymatic cycling method and the acylcarnitine/free carnitine ratio was calculated. These carnitine profiles were compared among HCM and healthy cats (control group).

**Results:** Fifty‐seven HCM cats and 72 serum samples were included in one group (B1;41, B2;13, C/D;18) and 13 experimental cats were included in the control group. The acylcarnitine/free carnitine ratio was significantly low in the B1, B2, and C/D group as compared to that in the control group. Significant differences were not observed in total carnitine, free carnitine, and acylcarnitine concentrations between these groups.

**Conclusions and Clinical Importance:** A modified carnitine profile was observed even in the early stage (B1) of HCM. This change could be primary anomaly, not to be due to hemodynamic compromise, and may reflect the myocardial metabolic impairment in HCM.

## 295 Abstract C52

### 295.1 Taurine Concentrations in Cavalier King Charles Spaniels: Reference Intervals, Diet, and Mitral Valve Disease Effects

#### 295.1.1 **Sonya R. Wesselowski**; Amanda Blake; Sonya Gordon; Jan Suchodolski; Joerg Steiner

##### 295.1.1.1 Texas A&M University

**Background:** Reference intervals for whole blood (WB) and plasma taurine may be breed‐specific. Platelets are rich in taurine and Cavalier King Charles Spaniels (CKCS) are frequently affected by thrombocytopenia and macrothrombocytosis. Additionally, increased plasma taurine concentrations have been reported in some dogs with myxomatous mitral valve disease (MMVD) while decreased concentrations have been reported in some dogs eating diets that do not meet World Small Animal Veterinary Association (WSAVA) guidelines.

**Hypothesis/Objectives:** Determine breed‐specific reference intervals for WB and plasma taurine concentrations in CKCS and determine whether taurine concentrations differ across MMVD stages or between CKCS eating diets that meet WSAVA guidelines versus other diets.

**Animals:** Two hundred CKCS.

**Methods:** Asymptomatic CKCS were prospectively recruited. Diet and supplement history was collected. Dogs were staged by echocardiography using MMVD consensus guidelines. Taurine concentrations were measured from deproteinized lithium heparin blood and plasma samples.

**Results:** There were 12 Stage A (6%), 150 Stage B (75%), and 38 Stage B2 (19%) CKCS. Seventy‐eight dogs (39%) ate diets meeting WSAVA guidelines and 116 (58%) did not. Taurine concentrations in plasma (P=0.444) and WB (P=0.073) were not significantly different across MMVD stages or between CKCS eating diets meeting WSAVA guidelines versus other diets (P=0.345 and P=0.527, respectively). Reference intervals for WB taurine (152–373 μM) and plasma taurine (51–217 μM) concentrations in CKCS were generated.

**Conclusions and Clinical Importance:** In CKCS, taurine concentrations do not differ significantly based on MMVD stage or diet.

## 296 Abstract C53

### 296.1 Peripheral Edema in Dogs: Clinical Characteristics and Etiologies

#### 296.1.1 **Bradley D. Whelchel**; Jessica Ward, DVM, DACVIM (Cardiology)

##### 296.1.1.1 Iowa State University

**Background:** Peripheral edema occurs secondary to numerous pathophysiologic processes; however, no previous reports have described clinical characteristics of peripheral edema across multiple etiologies.

**Hypothesis/Objectives:** To determine the prevalence of different etiologies of peripheral edema in dogs and describe characteristics that vary between etiologies.

**Animals:** 527 dogs diagnosed with peripheral edema at two tertiary referral hospitals.

**Methods:** A retrospective medical record review extracted clinical features including edema distribution and concurrent cavitary effusion, as well as clinicopathologic results. Final diagnosis and etiology of edema were determined based on evaluation of the complete medical record. Variables were compared amongst groups using t‐tests and Fisher's Exact tests with Bonferroni correction.

**Results:** The most common etiologies of peripheral edema were vasculitis (197 dogs, 37%), lymphatic or venous obstruction (LVO; 113 dogs, 21%), and hypoalbuminemia (94 dogs, 18%). Right‐sided congestive heart failure (R‐CHF) was uncommon (25 dogs, 5%). Edema was localized in 377 (72%) dogs and generalized in 142 (27%) dogs, and hypoalbuminemia was more likely to cause generalized edema compared to LVO or vasculitis (p<0.0001). Concurrent abdominal effusion (155 dogs, 29%) was more common than pleural (77 dogs, 15%) or pericardial (12 dogs, 2%) effusion. Abdominal and pleural effusion occurred more commonly in dogs with hypoalbuminemia or R‐CHF compared to LVO or vasculitis (p<0.0001). Pericardial effusion was more common in R‐CHF than hypoalbuminemia, LVO, or vasculitis (p<0.0001).

**Conclusions and Clinical Importance:** Distribution of edema and concurrent cavitary effusions can help predict the underlying etiology of peripheral edema in dogs.

## 297 Abstract C54

### 297.1 Effect of Beraprost on Hemodynamics and Cardiac Function in Dogs with Pulmonary Hypertension

#### 297.1.1 **Yunosuke Yuchi** ^1^; Ryohei Suzuki^2^, DVM, PhD; Takahiro Teshima^3^, DVM, PhD; Hirotaka Matsumoto^3^, DVM, PhD; Hidekazu Koyama^4^, DVM, PhD

##### 297.1.1.1 ^1^Nippon Veterinary and Life Science University; ^2^Assistant Professor, Veterinary Medicine, Nippon Veterinary and Life Science University; ^3^Associate Professor, Veterinary Medicine, Nippon Veterinary and Life Science University; ^4^Professor, Veterinary Medicine, Nippon Veterinary and Life Science University

**Background:** Pulmonary hypertension (PH) is a life‐threatening disease, characterized by increased pulmonary artery pressure and/or pulmonary vascular resistance (PVR). In humans, beraprost has been used for the treatment of PH, but the clinical efficacy of beraprost has not been reported in dogs with PH.

**Hypothesis/Objectives:** To evaluate the clinical effect of beraprost in dogs with PH.

**Animals:** Eleven dogs with PH (five dogs with pre‐capillary PH and six dogs with post‐capillary PH).

**Methods:** Prospective cohort study. All dogs underwent echocardiography and non‐invasive blood pressure measurements before and after beraprost administration. Continuous beraprost administration was performed twice daily at target dose of 15 μg/kg. Systemic vascular resistance (SVR) was calculated using systemic arterial pressure and left ventricular (LV) stroke volume, and PVR was calculated using tricuspid regurgitation pressure gradient and right ventricular (RV) stroke volume.

**Results:** Five dogs with right heart failure received beraprost and sildenafil concomitantly. With the beraprost administration (range: 15.2–22.0 μg/kg), SVR and PVR were significantly decreased, and LV and RV stroke volume were increased (all *P*<0.05). Additionally, beraprost increased LV ejection fraction, and LV and RV myocardial strain (all *P*<0.05). In dogs with post‐capillary PH, the beraprost administration caused no significant worsening in left atrial pressure indicators. No side effects of beraprost were observed in all dogs.

**Conclusions and Clinical Importance:** Beraprost improved pulmonary and systemic circulation through pulmonary and systemic vasodilating effects, suggesting that beraprost might be an additional treatment option in dogs with PH.

## 298 Abstract C56

### 298.1 Echocardiographic Assessment of Cardiac Function in Dogs with Pulmonary Hypertension Secondary to Respiratory Disease

#### 298.1.1 **Yunosuke Yuchi** ^1^; Ryohei Suzuki^2^, DVM, PhD; Takahiro Teshima^3^, DVM, PhD; Hirotaka Matsumoto^3^, DVM, PhD; Hidekazu Koyama^4^, DVM, PhD

##### 298.1.1.1 ^1^Nippon Veterinary and Life Science University; ^2^Assistant Professor, Veterinary Medicine, Nippon Veterinary and Life Science University; ^3^Associate Professor, Veterinary Medicine, Nippon Veterinary and Life Science University; ^4^Professor, Veterinary Medicine, Nippon Veterinary and Life Science University

**Background:** Pulmonary hypertension secondary to respiratory disease (RD‐PH) is caused by pulmonary vascular remodeling and hypoxia. Severe RD‐PH would induce fatal clinical signs of right‐sided heart failure (RHF) including syncope through right ventricular (RV) afterload and circulation insufficiency. However, no study has evaluated detailed cardiac function in dogs with RD‐PH.

**Hypothesis/Objectives:** To evaluate the cardiac function in dogs with RD‐PH.

**Animals:** Seventeen dogs with RD‐PH and fifteen healthy dogs.

**Methods:** Prospective cross‐sectional study. Dogs with RD‐PH were classified according to the PH probability (low/intermediate/high), respiratory disease (obstructive/restrictive), and the presence of RHF. All dogs underwent echocardiography including two‐dimensional speckle‐tracking echocardiography.

**Results:** Eight dogs with RD‐PH showed RHF (41%). Pulmonary artery to aortic diameter ratio (PA/Ao) was significantly higher in RD‐PH dogs without RHF than that in healthy dogs (P<0.01). Higher RV area, and lower left ventricular (LV) internal dimension, stroke volume, and LV and RV myocardial strain were observed in RD‐PH dogs with RHF (all P<0.05). Dogs with restrictive disease showed higher pulmonary vascular resistance estimated by echocardiography and lower RV myocardial strain than dogs with obstructive disease (all P<0.05).

**Conclusions and Clinical Importance:** Results of PA/Ao suggest that pulmonary arterial enlargement might be useful for the early detection of RD‐PH. In addition to RV, LV assessment might also be important in dogs with RD‐PH to estimate the progression to RHF. Restrictive respiratory disease might cause increased pulmonary vascular resistance and RV dysfunction more severely than obstructive disease.

## 299 Abstract C57

### 299.1 Transesophageal Echocardiography‐Related Complications During Mitral Valve Repair in Dogs

#### 299.1.1 **Kentaro Kurogochi**; Yasuyuki Nii; Tomoya Suzuki; Arane Takahashi; Masami Uechi

##### 299.1.1.1 JASMINE Veterinary Cardiovascular Medical Center

**Background:** Adverse events in transesophageal echocardiography (TEE) have been reported in human medicine. However, there are only a few such reports in veterinary medicine.

**Hypothesis/Objectives:** We aimed to determine the incidence and types of complications following TEE manipulation during mitral valve repair in dogs.

**Animals:** Fifty‐two client‐owned dogs (40 dogs: <5 kg, 12 dogs: >5 kg) underwent TEE to support mitral valve repair.

**Methods:** A small probe (9T; GE healthcare, Chicago) was used in dogs weighing <5 kg, and a large probe (6VT‐D) was used in dogs weighing >5 kg. The changes in the blood pressure and heart rate before and after TEE insertion were recorded. Narrowband imaging (NBI) endoscopy was performed twice, once after the induction of anesthesia and once before extubation, to evaluate the occurrence of new esophageal lesions. The type of injury was classified as follows: complex lesions (intramural hematoma, mucosal laceration), minor lesions (petechiae, ecchymosis), and minute lesions (only visible in NBI).

**Results:** The incidence of new lesions was not different between small and large probes (28% vs. 50%; *p*=0.17), and the most common injury type was minute lesions (major: 0%, minor: 18%, minute: 82%). Systolic blood pressure and heart rate were significantly decreased after TEE insertion (95 to 92 mm Hg; *p*<0.01, 127 to 120 bpm; *p*<0.01, respectively).

**Conclusions and Clinical Importance:** Esophageal mucosal injury was minimized after TEE. Since TEE might affect blood pressure and heart rate, hemodynamic changes should be monitored during this procedure.

## 300 NEUROLOGY

## 301 Abstract N24

### 301.1 Functional Connectivity Magnetic Resonance Imaging in Dogs Anesthetized with Dexmedetomidine and Propofol

#### 301.1.1 **Karen R. Munana** ^1^; SungHo Lee^2^, DVM, PhD; Erin Keenihan^1^, BVSc, DACVR; Gianluca Bini^3^, DMV, MRCVS, DACVAA; Jordan Schachar^1^, DVM

##### 301.1.1.1 ^1^College of Veterinary Medicine, North Carolina State University; ^2^University of North Carolina at Chapel Hill School of Medicine; ^3^College of Veterinary Medicine, The Ohio State University

**Background:** Functional connectivity magnetic resonance imaging (fc‐MRI) is a noninvasive technique to evaluate neuronal activity that measures blood oxygen level dependent (BOLD) signals. It involves the detection of spontaneous low frequency fluctuations in the brain's blood flow at rest, that are analyzed to identify anatomically distinct but functionally connected brain structures, or intrinsic brain networks, such as the default mode network (DMN).

**Hypothesis/Objectives:** This pilot study aimed to determine optimal intravenous anesthetic and imaging parameters for fc‐MRI in dogs.

**Animals:** Six healthy adult dogs.

**Methods:** Dogs were anesthetized with dexmedetomidine and propofol constant rate infusions (CRIs). Using a 3T scanner and 15 channel knee coil, BOLD‐Echo Planar Imaging (EPI) was performed to acquire fc‐MRI data while varying the dexmedetomidine dose and imaging parameters of repetition time (TR), echo time (TE), and voxel size. Temporal signal‐to‐noise ratio was calculated to determine optimal acquisition parameters. Data driven dictionary learning was used to distinguish intrinsic brain networks.

**Results:** Optimal acquisition parameters for BOLD‐EPI were: TR=1500 ms, TE=27 ms, pixel bandwidth=1775 Hz, slice thickness=3 mm, slice distance=3.75 mm, matrix size=6×64×26, and field of view=19.2×19.2×9.75 cm^3^, using a dexmedetomidine CRI of 2 μg/kg/hr. The DMN, visual and auditory networks, and cerebellum were identified with reasonable reliability (>0.5) and reproducibility (>0.7) based on intra‐class correlation and dual regression analyses, respectively.

**Conclusions and Clinical Importance:** fc‐MRI data obtained from dogs anesthetized with dexmedetomidine and propofol allows evaluation of intrinsic brain networks. This technique is a promising, noninvasive tool to investigate canine brain disease.

## 302 Abstract N26

### 302.1 Retrospective Review of Nerve Root Signature Associated with Cervical Intervertebral Disc Disease in Dogs

#### 302.1.1 **Jordan Schachar**; Alan Bocage, DVM; Peter Early, DVM, DACVIM (Neurology); Chris Mariani, DVM, PhD, DACVIM (Neurology); Karen Munana, DVM, MS, DACVIM (Neurology); Nathan Nelson, DVM, MS, DACVR; Natasha Olby, Vet MB, PhD, DACVIM (Neurology), MRCVS; James Robertson, MS

##### 302.1.1.1 College of Veterinary Medicine, North Carolina State University

**Background:** Intervertebral disc disease (IVDD) is the most common spinal cord disease in dogs. Little information is available regarding the unique clinical presentation of nerve root signature (NRS) due to IVDD.

**Hypothesis/Objective:** To detail the clinical and magnetic resonance imaging (MRI) findings in dogs displaying NRS associated with cervical IVDD.

**Animals:** 47 client‐owned dogs presenting with thoracic limb lameness not attributed to an orthopedic cause and MRI confirmed IVDD.

**Methods:** Medical records from 2010 to 2020 were retrospectively reviewed. Imaging studies were evaluated to characterize location and severity of neural tissue compression.

**Results:** The majority (77%) of dogs were >7 years old. The dachshund (n=10) was the most common breed, and there was a relatively even distribution of small (<10 kg) (55%) and large breed (45%) dogs. Disc material was significantly more likely to be lateralized (p=0.0005) and involve C5‐T2 discs (p=0.0009), however 42% cases involved C2‐5 discs. The most commonly affected site was C6‐7 (p=0.001), which was significantly more prevalent compared to historical canine IVDD populations. Severe nerve root compression was not found in all dogs, with some displaying absent compression entirely.

**Conclusion/Clinical Importance:** This study confirmed that NRS is most commonly associated with lateralized IVDD affecting the cervical intumescence, however, it can be seen with disease anywhere along the cervical spine. The high prevalence of C6‐7 intervertebral disc involvement suggests there may be unique anatomic factors that contribute to development of NRS at this site. Older dogs may be more likely to develop NRS.

## 303 Abstract N27

### 303.1 Spinal Cord and Dural Sac Termination and Morphometry in Different Dog Breeds

#### 303.1.1 **Amanda J. Valentino**; Courtney Sparks, PhD; Natasha Olby, Vet MB, PhD, MRCVS, DACVIM (Neurology)

##### 303.1.1.1 North Carolina State University

**Background:** Termination of the spinal cord is developmentally complex and prone to congenital malformations. Despite breed differences in vertebral morphology, there is little information available on this region in different dog breeds.

**Hypothesis/Objectives:** Describe the location of spinal cord and dural sac termination and filum terminale internum length (FTIL) across dog breeds and identify influencing factors.

**Animals:** 120 dogs aged >1 year with normal lumbar magnetic resonance imaging (MRI) studies.

**Methods:** Blinded retrospective study. Vertebral location of spinal cord and dural sac termination were recorded from sagittal T2‐weighted and HASTE lumbar MRIs, FTIL was measured on T2 images. Breed, weight, sex, and craniofacial classification were recorded. Vertebral location of terminal structures and FTIL were compared with weight, sex, and craniofacial classification using multivariate logistic models.

**Results:** There were 42 breeds, with 32 brachycephalic, 79 mesaticephalic, and 9 dolichocephalic dogs; 59 female, 61 male and median weight: 23.3 kg (4.1 to 62 kg). Sex and craniofacial classification were not related to vertebral level of spinal cord and dural sac termination or FTIL while weight was (P=0.0009, 0.0037, and <0.0001 respectively). Boston terriers, Corgis, and CKCS were outliers compared with similar weight dog breeds, with more cranial spinal cord termination in Boston terriers, and more caudal in Corgis and CKCS.

**Conclusions and Clinical Significance:** The location of spinal cord terminal structures is affected by weight, but not sex or cranial morphology. It does appear to differ in breeds known to have congenital skeletal malformations. These deserve further evaluation in larger cohorts.

## 304 SMALL ANIMAL INTERNAL MEDICINE ‐ GASTROENTEROLOGY

## 305 Abstract GI24

### 305.1 Feasibility Study Using Undercarboxylated Osteocalcin as a Surrogate for Serum Vitamin‐K in Chronic Enteropathy Dogs

#### 305.1.1 **Jillian M. Smith** ^1^; Elizabeth Lennon^2^, DVM, PhD, DACVIM

##### 305.1.1.1 ^1^University of Tennessee; ^2^Pamela Cole Assistant Professor of Internal Medicine, University of Pennsylvania

**Background:** Vitamin K deficiency has been described in chronic enteropathy, though evidence is sparse. Undercarboxylated osteocalcin (%ucOC) is the gold standard to assess vitamin K status in humans, but assays have not been validated in dogs.

**Hypothesis/Objectives:** To determine if commercially available enzyme immunoassays (EIAs) for undercarboxylated and carboxylated osteocalcin could be validated for use in dogs.

**Animals:** Previously banked canine serum was used.

**Methods:** EIAs for human undercarboxylated (Glu‐) and carboxylated (Gla‐) osteocalcin were performed according to the manufacturer's instructions. Undercarboxylated recombinant canine osteocalcin (rcOC) was used to spike samples. Assay performance was determined by measuring percent recovery, linearity, and dilutional parallelism.

**Results:** Gla‐OC and Glu‐OC had a recovery of 73.3±0.9% and 77.1±10.7%, respectively, with acceptable linearity and dilutional parallelism. Intra‐and inter‐assay % coefficient of variation was 1.7±0.3% and 4.4–13.6%, respectively, for Gla‐OC and 3.5±2.8% and 0.15±0.9% for Glu‐OC. rcOC demonstrated excellent accuracy and dilutional parallelism for Gla‐OC but could not be determined for Glu‐OC due to concentrations near the low end of the detectable range.

**Conclusions and Clinical Importance:** Validation of the Glu‐ and Gla‐ assays shows that these EIAs could potentially be used in dogs, but further study is needed to correlate to vitamin K status.

## 306 Abstract GI25

### 306.1 Comparison of Metronidazole versus a Synbiotic for Treating Dogs with Acute Diarrhea

#### 306.1.1 **Helene Stübing** ^1^; Andrea Reisinger^2^; Melanie Werner^3^; Stefan Unterer^3^; Jan Suchodolsky^4^; Jörg Steiner^4^; Jonathan Lidbury^4^; Katrin Hartmann^2^; Kathrin Busch^2^

##### 306.1.1.1 ^1^Clinic of Small Animal Medicine, LMU Munich, Munich, Germany; ^2^Clinic of Small Animal Medicine, Centre for Clinical Veterinary Medicine, LMU Munich, Munich, Germany; ^3^Clinic for Small Animal Internal Medicine, Vetsuisse Faculty, University of Zurich, Zurich, Switzerland; ^4^Gastrointestinal Laboratory, Department of Small Animal Clinical Sciences, Texas A&M University, College Station, TX, USA

**Background:** Although acute diarrhea (AD) in dogs is typically self‐limiting, metronidazole or synbiotic agents are frequently used to treat this condition.

**Objective:** To assess the effect of metronidazole compared to a synbiotic containing *Enterococcus faecium* on clinical improvement and fecal microbiota.

**Animals:** Nineteen dogs with AD of <5 days duration.

**Methods:** Prospective, randomized, double‐blinded clinical trial. Dogs were assigned to a metronidazole (METg; n=12; 10–20 mg/kg PO q12h for 7 days) or synbiotic group (SYNg; n=7; *E. faecium* DSM 10663 NCIMB 10415/4b1707; 10^8^ CFU/kg PO q12h for 7 days). Fecal consistency and defecation frequency were recorded daily for eleven days. The fecal microbiota was analyzed using the dysbiosis index (DI) on day 0, 6 and 30. Data were analyzed using mixed models with repeated measures.

**Results:** No significant difference was observed in fecal consistency (*p=0.25*) or defecation frequency (*p=0.91*) between METg and SYNg at any time point. There was a significant difference in the fecal DI on day 6 (METg median: 7.3; min–max: 0.9 to 8.9; SYNg median: ‐4.8; min–max: ‐5.9 to ‐1.0; *p<0.0001*) and day 30 (METg median: ‐2.0; min–max: ‐5.8 to 6.7; SYNg median: ‐4.4; min–max: ‐5.8 to ‐2.5; *p<0.0001*) between treatment groups.

**Conclusion and Clinical Importance:** Our study could not show any difference in the improvement of fecal consistency or defecation frequency between dogs with AD treated with metronidazole or a synbiotic. However, metronidazole leads to fecal dysbiosis, which persists for up to three weeks in some dogs.

## 307 Abstract GI26

### 307.1 Fecal Bile Acids Profiles in Cats with Chronic Enteropathy

#### 307.1.1 **Chi‐Hsuan Sung** ^1^; Sina Marsilio^2^; Betty Chow^3^; Kailee Zornow^4^; Jennifer Slovak^4^; Rachel Pilla^1^; Jonathan Lidbury^1^; Joerg Steiner^1^; Steve Hill^5^; Jan Suchodolski^1^

##### 307.1.1.1 ^1^Texas A&M University; ^2^Veterinary Medicine and Epidemiology, School of Veterinary Medicine, University of California‐Davis, Davis, CA, USA; ^3^VCA Animal Specialty and Emergency Center; ^4^Animal Medical Center; ^5^Flagstaff Veterinary Internal Medicine Consulting

**Background:** Dysmetabolism of bile acids (BAs) has been linked to chronic gastrointestinal diseases in humans and dogs.

**Objectives:** To assess fecal concentrations of unconjugated BAs and their correlation with the abundance of *Clostridium hiranonis* in cats with chronic enteropathy (CE).

**Animals:** 45 healthy cats, 22 cats with inflammatory bowel disease (IBD), and 34 cats with intestinal small cell lymphoma (SCL).

**Methods:** Fecal concentrations of unconjugated primary (i.e., cholic and chenodeoxycholic acids) and secondary BAs (i.e., lithocholic, deoxycholic, and ursodeoxycholic acids) were measured by gas chromatography‐mass spectrometry. Fecal *C. hiranonis* abundance was determined by quantitative PCR. Concentrations of BAs were compared between groups. The correlation between the *C. hiranonis* abundance and BAs concentrations was evaluated by Spearman's correlation test. Statistical significance was set at P<0.05.

**Results:** Total fecal BA concentrations were higher (*P*<0.0004; Mann‐Whitney test) in cats with CE (IBD and SCL; median [range]: 6,254 [125–17,620] ng/mg) than in healthy control cats (4,316 [1,150–12,910]). Fourteen percent (8/56) of cats with CE had total fecal primary BA concentration above the upper limit of the reference interval. The composition of primary and secondary BAs was abnormal in 23% (13/56) of cats with CE. The abundance of *C. hiranonis* and percentage of primary BAs were negatively correlated (r_s_ = ‐0.67; 95% CI: [‐0.78, ‐0.56]; *P*<0.0001). No significant differences were found between cats with IBD and cats with SCL.

**Conclusions and Clinical Importance:** A subset of cats with CE had evidence of BA dysmetabolism.

## 308 Abstract GI27

### 308.1 Prevalence of *Clostridioides difficile* in Canine Feces and Its Association with Dysbiosis

#### 308.1.1 **Melanie Werner** ^1^; Patricia Ishii^2^; Rachel Pilla^2^; Jonathan Lidbury^2^; Joerg Steiner^2^; Kathrin Busch^3^; Stefan Unterer^4^; Jan Suchodolski^2^

##### 308.1.1.1 ^1^Clinic for Small Animal Internal Medicine, Vetsuisse Faculty, Zurich, Switzerland; ^2^Gastrointestinal Laboratory, Department of Small Animal Clinical Sciences, Texas A&M University; ^3^Clinic of Small Animal Medicine, Ludwig‐Maximilians‐Universitaet; ^4^Clinic for Small Animal Internal Medicine, Vetsuisse Faculty

**Background:** Although *Clostridioides difficile* (CD) can be detected in dog feces, its clinical significance is controversial. In humans, CD is associated with microbial bile‐acid‐dysmetabolism.

**Hypothesis/Objectives:** To correlate the prevalence of CD, intestinal dysbiosis, and the abundance of the bile‐acid‐converting bacterium *Clostridium hiranonis*.

**Animals:** 358 canine fecal samples submitted for routine diagnostics and clinical research purposes. Thirty‐five samples were from dogs with chronic enteropathy where information on treatment response was available.

**Methods:** Retrospective cohort study. Fecal samples were analyzed by qPCR for CD and the dysbiosis index (DI), including *C. hiranonis*.

**Results:** 130 of 358 samples (36%) were positive for CD. Samples positive for CD had a significantly higher DI (median [range]: 4.4 [‐3.5 to 8.8]) compared to negative samples (‐1.0 [‐7.2 to 8.9]; p<0.0001). Only 15/130 samples (12%) that were positive for CD had a normal DI (<0). Dogs positive for CD had significantly lower abundances of *C. hiranonis* (median [range]: 0.1 [0.1–7.5]) compared to those that were negative (6.2 [0.1–7.5]; p<0.0001). Only 17/130 (13%) samples positive for CD had a normal abundance of *C. hiranonis*. Six of the 35 dogs (17%) with chronic enteropathy tested positive for CD (4/6 food‐responsive, 1/6 steroid‐responsive, 1/6 antibiotic‐responsive).

**Conclusions and Clinical Importance:** An association between the presence of CD and dysbiosis and decreased abundance of *C. hiranonis* was observed. Four dogs with CE that tested positive for CD responded well to dietary modification without concurrent antimicrobial treatment.

## 309 Abstract GI28

### 309.1 A Randomized Non‐Controlled Open‐Label Trial in Cats Comparing Cyclosporine and Prednisolone for Treating Chronic Pancreatitis

#### 309.1.1 **Yu‐An Wu** ^1^; Jonathan Lidbury^1^; M. Katherine Tolbert^1^; Samiran Sinha^2^; Jan Suchodolski^1^; Jörg Steiner^1^

##### 309.1.1.1 ^1^Gastrointestinal Laboratory, Texas A&M University; ^2^Department of Statistics, Texas A&M University

**Background:** Cyclosporine or prednisolone has been recommended for treating chronic pancreatitis (CP) in cats in addition to symptomatic treatment. However, no prospective studies have compared the efficacy of these two medications.

**Hypothesis:** Cyclosporine is more effective than prednisolone in reducing serum feline pancreatic lipase immunoreactivity (fPLI).

**Animals:** Thirty‐one client‐owned cats with at least a two‐week history of clinical signs suggestive of CP and a recent serum fPLI concentration >10 μg/L and a pre‐treatment baseline concentration >5.3 μg/L.

**Methods:** Randomized non‐controlled open‐label clinical trial. Cats were randomized to receive either cyclosporine (5 mg/kg orally once daily for 21 days) or prednisolone (2 mg/kg orally twice daily for the first 5 days then tapered to 1 mg/kg orally twice daily for next 16 days). Serum fPLI concentration was measured at baseline and on days 10 and 21 of the assigned treatment.

**Results:** Baseline serum fPLI concentrations (median, range) were comparable between the cyclosporine (19.9, 5.5–206.4 μg/L) and prednisolone groups (20.3, 6.7–158.0 μg/L; P=0.85). Endpoint serum fPLI concentrations were not significantly different between the cyclosporine (9.2, 3–172 μg/L) and prednisolone groups (28.3, 2.6–156 μg/L; P=0.14) (Figure 1). Absolute and percent changes of endpoint serum fPLI from baseline and the proportion of cats with at least 50% improvement of baseline serum fPLI were not different between the two groups (P=0.06, 0.07, and 0.11, respectively).

**Figure:**
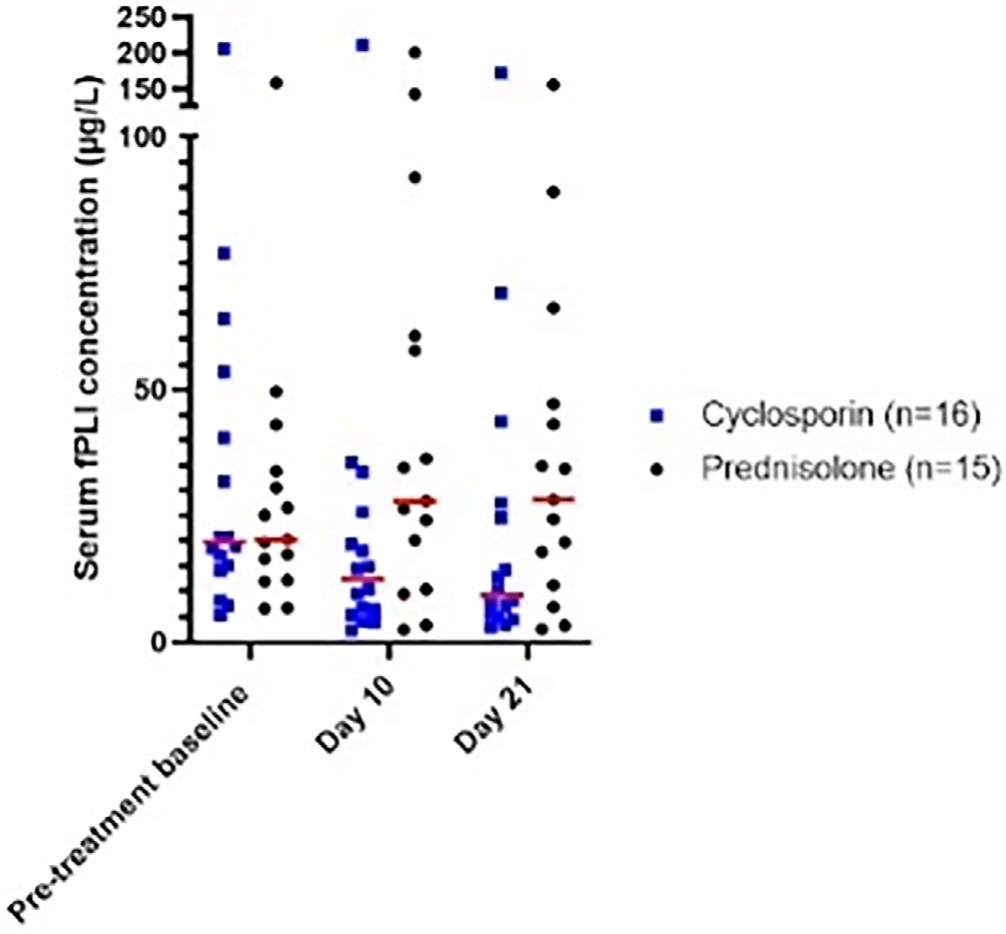
**Figure 1** Serum fPLI concentrations of each cat receiving cyclosporine or prednisolone over time. Red bars denote the median of the serum fPLI concentrations. fPLI: feline pancreatic lipase immunoreactivity.

**Conclusions and Clinical Importance:** The efficacy was not statistically different between cyclosporine and prednisolone.

## 310 Abstract GI29

### 310.1 Histopathologic Examination of Serial Pancreatic Sections from Shelter Cats

#### 310.1.1 **Yu‐An Wu** ^1^; Shelley Newman^2^; Jörg Steiner^1^

##### 310.1.1.1 ^1^Gastrointestinal Laboratory, Texas A&M University; ^2^Newman Specialty VetPath

**Background:** It has previously been suggested that mild lymphocytic infiltrates are normal in the feline pancreas.

**Objectives:** To describe the prevalence and characteristics of exocrine pancreatic histopathological lesions in cats euthanized for population control.

**Animals:** Forty‐eight shelter cats euthanized for population control.

**Methods:** Pancreata were collected within 1.5 hours of death, sectioned every 1 cm, and H&E‐stained slides were prepared for grading by a board‐certified veterinary pathologist using a previously published scoring system. Blood was collected and submitted for measuring serum feline pancreatic lipase immunoreactivity (fPLI). The mean cumulative score of lymphocytic inflammation (MCS‐LI) for each cat was calculated as the sum of the lymphocytic inflammation score of each section divided by number of sections. Correlation between MCS‐LI and fPLI was evaluated.

**Results:** Only 1 (2%) cat showed lesions consistent with mild acute pancreatitis, but 15 (31%) showed lesions consistent with mild chronic pancreatitis in at least one section. Among the 15 cats with lesions suggestive of chronic pancreatitis, 13 (87%) showed lymphocytic/mononuclear inflammation, 8 (53%) fibrosis, and 2 (13%) cystic degeneration. Most cats with lesions had few sections affected (Figure 1). Serum fPLI was not significantly correlated with MCS‐LI (rs=0.16, P=0.29), but the cat with most of its sections affected (11/12 sections) had the highest fPLI concentration (Figure 2).

**Figure:**
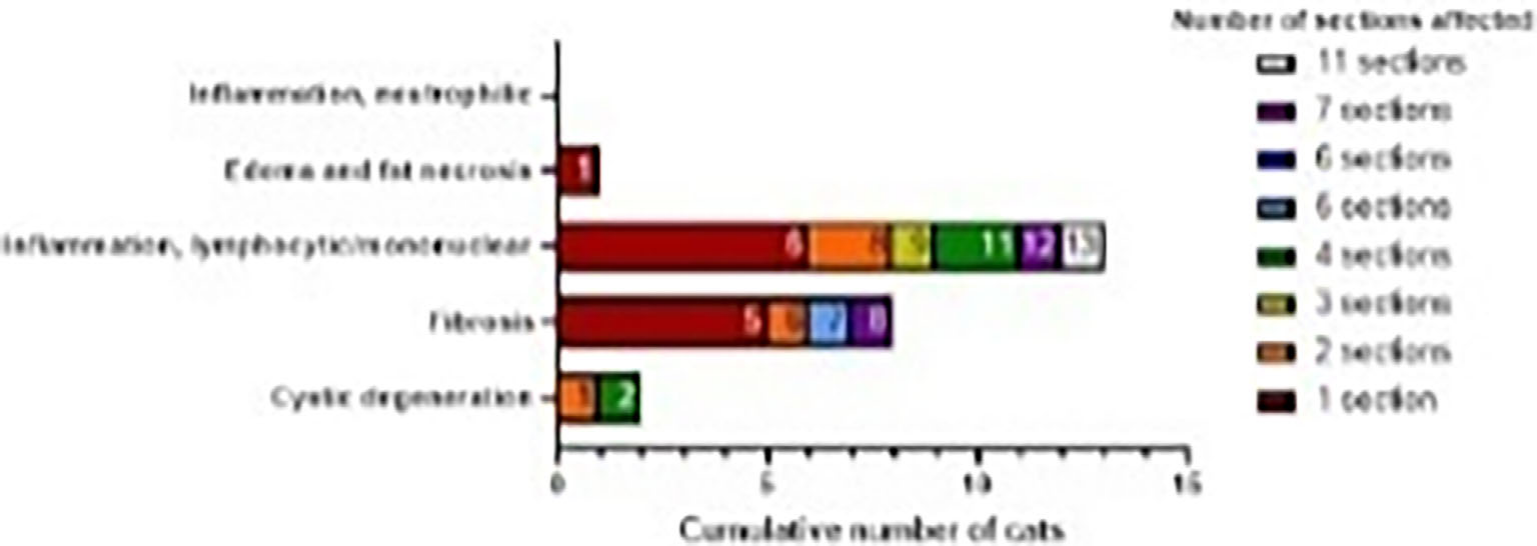
**Figure 1** Number of cats by lesion presented in at least one section. Color denotes the number of sections affected.

**Figure:**
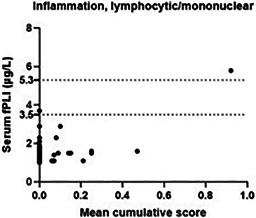
**Figure 2** Relationship between serum fPLI and mean cumulative score of lymphocytic inflammation for each cat. Dotted lines denote the lower and upper limit of the grey zone of fPLI. fPLI: feline pancreatic lipase immunoreactivity.

**Conclusions and Clinical Importance:** While mild lymphocytic inflammation was frequently identified, it was also highly localized. The clinical significance of these lesions remains to be determined.

## 311 Abstract GI30

### 311.1 Characterizing the Serum Proteome of Cats with Chronic Enteropathy

#### 311.1.1 **Jane Yu** ^1^; Lara Boland^2^, BVSc (Hons I), FANZCVS (Feline Medicine), DECVIM (CA); Melissa Catt^3^, BVSc, MANZCVS (Medicine of Cats); Leah Puk^3^; Nadia Wong^4^, BVSc, MANZCVS (Small Animal Surgery); Peter Bennett^5^, BVSc, FANZCVS (Canine Medicine), DACVIM (Oncology, Small Animal Medicine); Valerie Wasinger^6^, PhD; Craig Ruaux^7^, BVSc (Hons), PhD, MACVSc, DACVIM (SAIM)

##### 311.1.1.1 ^1^Sydney School of Veterinary Science, The University of Sydney; ^2^Specialist in Feline Medicine and Small Animal Medicine, Senior Lecturer, UVTHS, Sydney School of Veterinary Science; ^3^Paddington Cat Hospital; BVSc (Hons) MANZCVS (Medicine of Cats); ^4^McIvor Road Veterinary Centre; ^5^Specialist in Veterinary Oncology and Internal Medicine, Associate Professor, UVTHS, Sydney School of Veterinary Science; ^6^Senior Research Scientist, Senior Lecturer, Bioanalytical Mass Spectrometry Facility, UNSW; ^7^Hospital Director of UVTHS, Specialist in Small Animal Medicine, Associate Professor, UVTHS, Sydney School of Veterinary Science

**Background:** Serum protein biomarkers are used as novel diagnostic and monitoring tools for people with chronic enteropathies but have not been explored in cats.

**Hypothesis/Objectives:** The aim of this study was to characterize serum proteome in cats to identify markers differentiating healthy cats from cats with chronic enteropathies including idiopathic inflammatory bowel disease (IBD) and low‐grade alimentary lymphoma (LGAL).

**Animals:** Ten cats with IBD, 2 with LGAL, and 19 healthy control cats were recruited.

**Methods:** A prospective, multicenter study was performed. Cats presenting with vomiting, diarrhoea, inappetence and/or weight loss of at least 4 weeks were recruited. Cats with extra‐gastrointestinal diseases were excluded and only cats with histologically confirmed gastrointestinal inflammation or LGAL were included. Healthy cats were recruited. Serum samples were analysed using mass spectrometry based proteomic techniques.

**Results:** Twenty‐seven proteins were significantly (p<0.02) differentially expressed among IBD cats and controls with ≥5‐fold change in abundance. Thrombospondin‐1 (TSP1) showed a significant difference with >60‐fold increase in abundance (p<0.01) in cats with IBD (median 4.5, interquartile range 10) compared to controls (median 0, interquartile range 0) using the Mann‐Whitney U test. There was also a tendency towards reduced TSP1 in the two cats with LGAL compared to IBD cats.

**Conclusion and Clinical Importance:** The presence of gastrointestinal inflammatory disease is associated with increased abundance of several proteins, including TSP1, in the serum proteome of cats. Further investigation of TSP1 as a diagnostic and prognostic marker for feline chronic enteropathies is warranted.

**Table:** 

Identified proteins	Fisher's exact test (p<0.02)	Fold change
Coagulation factor V	<0.00010	102.60
Thrombospondin‐1	<0.00010	61.75
Heparin cofactor 2	0.0002	39.90
Apolipoprotein E	<0.00010	38.95
GDH/6PGL endoplasmic bifunctional protein	0.0003	36.10
Antithrombin‐III	<0.00010	33.25
Phosphatidylcholine‐sterol acyltransferase	0.0009	32.30
Carboxypeptidase N catalytic chain	<0.00010	31.35
Plastin‐2	0.0034	28.50
Apolipoprotein C‐III	<0.00010	28.50
Inter‐alpha‐trypsin inhibitor heavy chain H3	<0.00010	27.55
Complement factor I	0.0053	26.60
Profilin‐1	0.0003	22.80
Apolipoprotein B‐100	0.013	22.80
Coagulation factor IX	0.0003	20.90
Tropomyosin alpha‐4 chain	0.0054	10.77
Insulin‐like growth factor‐binding protein complex acid labile subunit	0.014	9.03
Apolipoprotein C‐I	<0.00010	8.21
Transthyretin	0.0095	7.92
Apolipoprotein E	0.0086	7.13
Hemoglobin subunit beta	0.0063	6.49
Serum paraoxonase/arylesterase 1	0.013	6.27
Complement	0.0055	5.95
Fibronectin (Fragment)	<0.00010	5.32
Actin, cytoplasmic 1	0.0012	5.27
Hemopexin	0.0150	5.13
Beta‐enolase	0.0003	‐5.79

Serum proteins (n=27) showing significantly differing abundances (fold change ≥5 (p<0.02) between cats with idiopathic IBD and healthy controls.

**Image 1**

**Figure:**
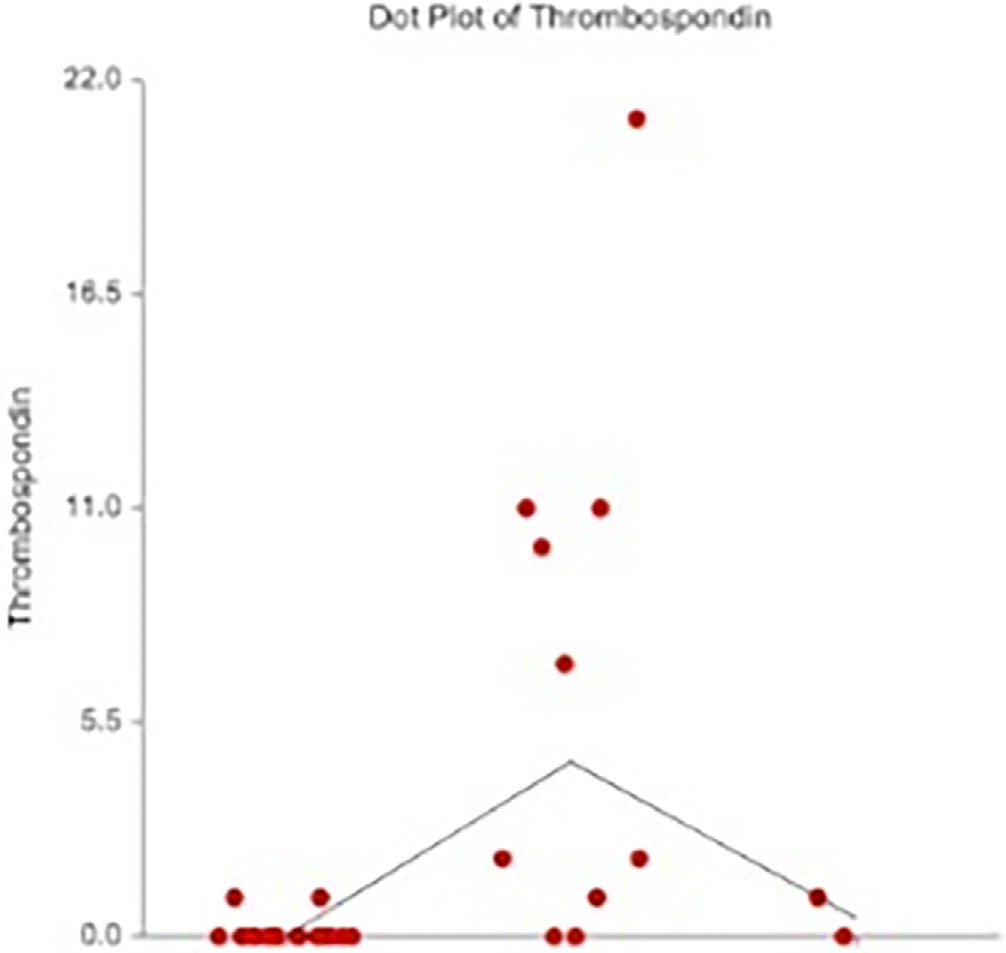
A dot plot showing relative abundance, based on spectral count, of thrombospondin‐1 in healthy controls, cats with idiopathic IBD and cats with LGAL.

## 312 Abstract GI31

### 312.1 Microbiome Responses to Fecal Microbiota Transplantation in Cats

#### 312.1.1 Dawn D. Kingsbury^1^; Connie Rojas^2^, PhD; Zhandra Entrolezo^3^, BS; Jessica Jarett^3^, PhD; Guillaume Jospin^3^, MS; Alex Martin^3^, BA; Jonathan Eisen^4^, PhD; **Holly Ganz** ^3^, PhD

##### 312.1.1.1 ^1^AnimalBiome; ^2^Postdoc, Department of Evolution and Ecology, Genome Center, University of California, Davis; ^3^AnimalBiome; ^4^Professor, Department of Evolution and Ecology, Genome Center, University of California, Davis

**Background:** Delineation of fecal microbiota transplantation (FMT)'s improvement in feline chronic enteropathy (CE) with concordant changes in the fecal microbiome is imperative.

**Objective:** To evaluate FMT's ability to improve feline CE signs, examining corresponding microbiome shifts.

**Animals:** FMT capsules contained 8 healthy pet cats’ lyophilized feces. Sixty‐eight CE cats were recruited online. Data from 82 cats with no signs or disease diagnoses comprised a healthy reference.

**Methods:** FMT treatment course consisted of one capsule given orally twice daily for 25 days.

CE fecal samples were collected before and 2 weeks post‐FMT. Owners reported on health and response to treatment. Bacterial 16S rRNA amplicons from fecal samples were sequenced and analyzed.

**Results:** Seventy‐eight percent (53/68) of cats reported improvement in signs (responders), and 22% (15/68) exhibited no change or a worsening of clinical signs (Non‐responders). Changes in relative abundances in pre and post‐FMT samples indicate Responders exhibited increased *Peptococcus* (P=0.001), decreased *Prevotella* 9 (P=0.03), and decreased *Sutterella* (P=0.03) compared to Non‐responders.

*Escherichia* abundances increased significantly post‐treatment (P=0.039) in Non‐responders, remaining unchanged in Responders. Responders’ microbiomes grew more similar to healthy cat microbiomes post‐FMT; Non‐responders grew less similar to the healthy reference (P=0.0486). FMT recipients with a prior IBD diagnosis, and those reported to have chronic diarrhea exhibited increases in similarity to the healthy reference set.

**Conclusions and Clinical Importance:** Signs improved after FMT. Responders shifted positively towards healthy reference set. *Escherichia* is an underappreciated CE response indicator to FMT.

**Figure:**
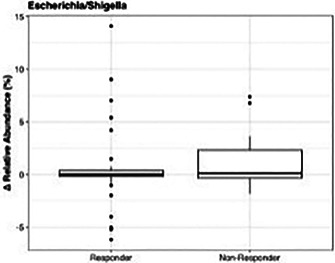
**Figure 1** Escherichia abundances increase in Non‐Responder animals coterminously with FMT treatment. The change in relative abundance (postFMT–preFMT) of the genera *Escherichia* was plotted for cats that reported improvement in their clinical signs (Responders), and those that exhibited no change or a worsening of their clinical signs (Non‐responders). The two groups differed in their relative abundances of *Escherichia* (Linear Mixed Model LRT χ2=4.23, p=0.039). The model specified Δ *Escherichia* abundances as the dependent variable, response to treatment, initial symptoms, IBD status, and dietary category as independent variables, and two random effects (age in years and breed).

**Figure:**
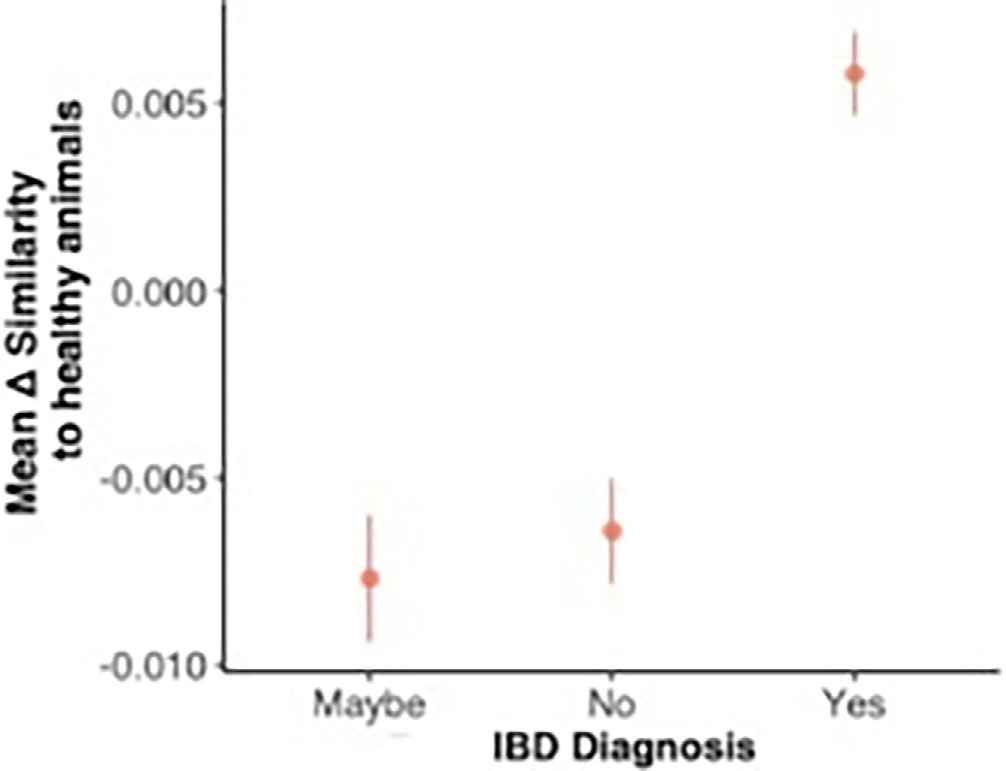
**Figure 2** The gut microbiomes of cats with a prior IBD diagnosis grow similar to those of healthy animals after FMT treatment. We computed the similarity between the gut microbiomes of recipient animals to the healthy reference set using Weighted Unifrac distances (1 minus Weighted Unifrac dissimilarity scores). The average similarity scores for the three IBD categories were plotted. The groups vary in their similarity scores (Linear Mixed Model LRT χ2=80.24, p<0.001). The model specified similarity scores as the dependent variable, and response to treatment, initial symptoms, and IBD diagnosis as independent variables.

## 313 Abstract GI32

### 313.1 Food‐Responsive Enteropathy Occurrence in Dogs with Chronic Enteropathy in São Paulo‐Brazil

#### 313.1.1 **Ana Rita C. Pereira** ^1^; Ricardo Duarte^2^; Patricia Eri Ishii^3^; Fabio Teixeira^4^

##### 313.1.1.1 ^1^School of Veterinary Medicine and Animal Science, University of São Paulo, Brazil; ^2^GastroVet/AllCare Vet/Centro Universitário das Faculdades Metropolitanas Unidas (FMU); ^3^Gastrointestinal Laboratory, Department of Small Animal Clinical Sciences, Texas A&M University, TAMU, College Station, TX, USA; ^4^Anclivepa's Veterinary College

**Background:** Food‐responsive enteropathy (FRE) is a form of chronic enteropathy (CE) in which patients respond to a dietary trial. There is no data regarding the occurrence of FRE in dogs with CE in Brazil.

**Objectives:** To assess the occurrence of FRE among dogs with CE and describe the frequency of all gastrointestinal disorders in a gastroenterology service in São Paulo, Brazil.

**Animals:** Retrospective study of 137 dogs presented at a gastroenterology service in São Paulo, Brazil.

**Methods:** Retrospective analysis of medical records of dogs treated in a gastroenterology service in two hospitals in São Paulo–Brazil, during the year of 2021. Dogs presented with vomiting, diarrhea, or both for over 3 weeks were included in the study after the exclusion of parasites, infections, and hypoadrenocorticism. Dogs were diagnosed with FRE when clinical signs remission occurred after a dietary trial with a hydrolyzed hypoallergenic commercial diet up to 45 days.

**Results:** 152 dogs visited the gastroenterology service during the year of 2021. Fifteen dogs were excluded for no follow‐up evaluation. Among 137 dogs with gastrointestinal disorders, there were 71 dogs (51.8%) diagnosed with CE, 26 with gallbladder disorders (19%), 12 (8.8%) with chronic hepatitis, 9 (6.6%) with protein losing enteropathy, and 19 (13.9%) with other diagnosis. From all CE cases, 44 (62.0%) were FRE.

**Conclusions:** FRE was the most frequent gastrointestinal disorder [32.1% (44/137)] among dogs with CE. CE was the main cause of clinical appointments in a gastroenterology service in Brazil.

## 314 Abstract GI33

### 314.1 Retrospective Evaluation of the Risk of Gastrointestinal Bleeding in Dogs Receiving Ophthalmic Non‐steroidal Anti‐inflammatory Drugs

#### 314.1.1 **Laura R. Van Vertloo** ^1^; Rachel Allbaugh^2^, DVM, MS, DACVO; Hannah Terhaar^3^, DVM; Austin Viall^4^, DVM, MS, DACVP

##### 314.1.1.1 ^1^Iowa State University; ^2^Associate Professor, Veterinary Clinical Sciences, Iowa State University; ^3^Ophthalmology Resident, Colorado State University; ^4^Associate Professor, Veterinary Pathology, Iowa State University

**Background:** Topical ophthalmic NSAIDs are routinely administered to dogs with risk factors for gastrointestinal (GI) bleeding, including concurrent systemic administration of glucocorticoids or NSAIDs, despite the known systemic absorption of some ophthalmic drugs. Whether ophthalmic NSAID administration increases risk of GI bleeding is unknown.

**Hypothesis/Objectives:** To determine if ophthalmic NSAID administration is associated with increased risk of GI bleeding in dogs.

**Animals:** 204 dogs that received ophthalmic NSAIDs with or without concurrent systemic prednisone or NSAID, 152 dogs that received oral prednisone alone, and 136 dogs that received oral carprofen alone from a university hospital population.

**Methods:** Retrospective study. Medical records of dogs receiving ophthalmic NSAIDs (ketorolac, diclofenac, or flurbiprofen) were reviewed for signalment, GI bleed, type of ophthalmic NSAID, concurrent medications, and comorbidities. Comparisons of GI bleed incidence were made between: 1) dogs on each ophthalmic NSAID; 2) dogs receiving ophthalmic NSAIDs alone and dogs receiving combined ophthalmic NSAIDs and oral prednisone or carprofen; 3) dogs receiving combined ophthalmic NSAIDs and oral prednisone or carprofen and dogs receiving oral prednisone or carprofen alone.

**Results:** 18/204 (0.9%) dogs receiving ophthalmic NSAIDs developed a GI bleed. There was no significant association between type of ophthalmic NSAID received or co‐administration of systemic prednisone or carprofen and GI bleeding. Co‐administration of ophthalmic NSAID and systemic prednisone or carprofen did not increase the risk of GI bleeding when compared to systemic administration alone.

**Conclusions and Clinical Importance:** In this study administration of ophthalmic NSAIDs did not increase the risk of GI bleeding in dogs.

## 315 Abstract GI34

### 315.1 Oxidative Stress in Dogs with Acute Pancreatitis

#### 315.1.1 **Harry Cridge** ^1^; Nicole Tusa^2^, DVM; Nyssa Levy^3^, MS, DVM, DACVECC; Daniel Langlois^3^, DVM, DACVIM (SAIM); Angel Abuelo^3^, DVM, PhD, DAVBP, DECBHM

##### 315.1.1.1 ^1^Michigan State University; ^2^Resident, College of Veterinary Medicine, Michigan State University; ^3^Assistant Professor, College of Veterinary Medicine, Michigan State University

**Background:** Oxidative stress is considered a pathomechanism of acute pancreatitis (AP), but no studies have characterized oxidant status in dogs with naturally occurring AP.

**Hypothesis/Objectives:** To evaluate measures of oxidant status in dogs with AP and to determine if these measures correlate with AP severity.

**Animals:** 15 dogs with AP and 9 control dogs.

**Methods:** Prospective observational study. Diagnosis of AP was based on history, elevated pancreatic lipase concentration, and ≥2 ultrasonographic findings consistent with AP. Plasma reactive metabolite (RM) concentrations and antioxidant potential (AOP) were measured in dogs with AP and healthy controls. Mann‐Whitney U tests were used to compare concentrations of RM, AOP, and the RM to AOP ratio (RM:AOP) between study populations. Spearman's rank correlation coefficients (Rs) were used to investigate potential associations between oxidant status and disease severity, as measured by the canine acute pancreatitis severity (CAPS) score.

**Results:** RM concentrations (median 65 RFU/μL, range 20–331 RFU/μL) and RM:AOP (7, 4–109) were higher in AP dogs than control dogs (RM: 25 RFU/μL, 16–41 RFU/μL) (RM:AOP: 4, 2–7) (P<0.001 for both comparisons). AOP concentrations were not different between populations (P=0.59). Neither RM (R_s_=0.23, P=0.41) nor RM:AOP (R_s_=0.002, P=0.99) were correlated with disease severity. No dogs died.

**Conclusions and Clinical Importance:** Dogs with AP had altered oxidant status, but these alterations did not correlate with a disease severity index. Larger studies are needed to determine if oxidant status is associated with mortality.

## 316 SMALL ANIMAL INTERNAL MEDICINE ‐ NEPHROLOGY/UROLOGY

## 317 Abstract NU24

### 317.1 Benign Feline Ureteral Obstruction: Outcome of Cats Undergoing Medical Management

#### 317.1.1 **Isabelle Merindol** ^1^; Catherine Vachon^2^, DVM, DES, DACVIM (SAIM); Marilyn Dunn^3^, DVM, MVSc, DACVIM (SAIM)

##### 317.1.1.1 ^1^Centre Hospitalier Universitaire Vétérinaire (St‐Hyacinthe) Faculté de Médecine Vétérinaire, Université de Montréal; ^2^CHUV, Faculty of Veterinary Medicine, University of Montreal, St Hyacinthe, Quebec; ^3^Professor and Director of Interventional Medicine, CHUV, Faculty of Veterinary Medicine, University of Montreal

**Background:** Scarce information is available regarding outcome of medical management (MM) of feline benign ureteral obstruction (FUO).

**Hypothesis/Objectives:** Describe clinical characteristics and report outcome of MM of FUO.

**Animals:** Seventy‐two client‐owned cats with 103 obstructed kidneys.

**Methods:** Medical records of cats diagnosed with FUO between 2010 and 2021 that received >72 hours of MM were retrospectively reviewed. Clinical data, treatment, and outcome were recorded. Outcome was classified as success, partial success, and failure based on urinary tract ultrasound.

**Results:** Seventy‐two cats with 103 obstructed kidneys were enrolled. Cause of obstruction was: ureterolith(s) in 73% (75/103), stricture in 14% (14/103), and pyonephrosis in 14% (14/103) of kidneys. Median creatinine at presentation was 355 umol/L (range: 115–1885). Median creatinine following MM was 235 umol/L (range: 104–1326). Outcome following MM was considered a success in 30% (31/103), partial success in 13% (13/103), and failure in 57% (59/103) of kidneys. Success was reported in 23% (17/75) of kidneys with ureterolith(s), 50% (7/14) with pyonephrosis, 50% (7/14) with strictures. Median time for a successful outcome was 19 days (range: 3–115). Mean ureterolith length was 1.96 (1–3.3), 3.41 (1.9–5.1), and 3.33 mm (1–7.7) in patients considered a success, partial success, and failure, respectively. Mean survival time (MST) was 509 days (4–1801) for deceased patients. At time of writing, 20 cats were alive with MST of 919 days (120–3459).

**Conclusions and Clinical Importance:** Our study suggests a higher success rate for MM of FUO than previously reported.

## 318 Abstract NU26

### 318.1 Urinary Clusterin and Cystatin B Concentrations in Dogs with Kidney Disease

#### 318.1.1 **Carolina Rivera** ^1^; Nick Jeffery^2^, BVSc, PhD, MSc; Rachel Cianciolo^3^; Jessica Hokamp^4^, DVM, PhD, DACVP; Sarah Peterson^5^, MD, PhD; Murthy Yerramilli^5^, PhD; Mary Nabity^6^, DVM, PhD, DACVP

##### 318.1.1.1 ^1^College of Veterinary Medicine and Biomedical Sciences, Texas A&M; ^2^Professor, Small Animal Clinical Sciences, College of Veterinary Medicine and Biomedical Sciences, Texas A&M; ^3^Assistant Professor, Veterinary Medical Center, The Ohio State University; ^4^Assistant Professor, Veterinary Medical Center, The Ohio State University; ^5^IDEXX Laboratories, Inc.; ^6^Associate Professor, Veterinary Pathobiology, College of Veterinary Medicine, Texas A&M University

**Background:** Urinary clusterin (uClusterin) and cystatin B (uCysB) were recently developed for detection of active renal injury in dogs.

**Objectives:** Determine the concentration of these biomarkers in different forms of biopsy‐confirmed renal disease and evaluate their association with conventional biomarkers and disease progression.

**Animals:** Urine from client‐owned dogs (n=200) with kidney disease submitted to the International Veterinary Renal Pathology Service between 2008 and 2018.

**Methods:** Retrospective. uClusterin and uCysB were measured using an ELISA. Median and interquartile range were determined for diagnostic categories containing >10 cases. Correlation of biomarkers with serum creatinine, symmetric dimethylarginine (SDMA), and urine protein‐creatinine ratio (UPC) was performed using StataBE 17. Kruskal‐Wallis nonparametric ANOVA was used to test for significant differences among disease categories and IRIS stages.

**Results:** Using a cutoff of 350 ng/mL for uClusterin and 50 ng/mL for uCysB, biomarkers were increased in 85% and 70% of samples, respectively, and median concentration was highest in the membranoproliferative glomerulonephritis (MPGN) and mixed MPGN categories (Table 1). Biomarkers were weakly positively correlated with creatinine, SDMA, and UPC (r=0.200–0.398). Increasing concentrations of uClusterin and uCysB were associated with International Renal Interest Society (IRIS) stage (p˂0.05; Table 2). Normalization of biomarkers with urine creatinine did not significantly alter correlations with traditional biomarkers or association with IRIS stages.

**Table:** Table 1. Urinary clusterin and cystatin B in dogs with kidney disease. Data are presented as median and interquartile range (Q1–Q3).

	n	uClusterin (ng/mL)	uCysB (ng/mL)
Focal segmental glomerular sclerosis	37	2966* (1007–6341)	113* (54–197)
Membranoproliferative glomerulonephritis (MPGN)	21	8627* (3990–12,500)	280* (97–375)
Mixed MPGN	12	11,878* (4809–12,500)	204* (91–357)
Membranous GN (MGN)	19	4101* (1853–7682)	145* (58–269)
Mesangioproliferative GN	15	2904* (1760–5455)	121* (74–314)
Maldevelopment	20	528 (109–2351)	50 (18–129)
Amyloidosis	15	1073* (594–7430)	115* (45–261)
Acute tubular necrosis	11	3160* (441–8097)	40 (15–255)
GBM‐opathy**	11	3523* (1229–7800)	106 (34–160)
Other***	39	1950 (208–5252)	80 (19–239)

* Significant difference (p˂0.05) between each disease category as compared to biomarker concentrations in the maldevelopment category.

** Disorder of the glomerular basement membrane

*** Disease categories with less than <10 cases include: mixed MGN, podocytopathy, sclerosing immune complex mediated GN, thrombotic microangiopathy, interstitial nephritis, nephrosclerosis, glomeruli lipid emboli, obstructive nephropathy, arterionephrosclerosis, non‐amyloidotic fibrillary glomerulopathy, large cell lymphoma, and non‐characterized/diagnosed cases.

**Table:** Table 2. Urinary clusterin, cystatin B and SDMA in dogs with varying IRIS stages. Data presented as medians.

IRIS Stage	n	uCysB (ng/mL)	uClusterin (ng/mL)	SDMA (ug/dL)
1	96	86	2338	15
2	34	118	2243	25
3	47	144*	4453*	34

* Significant difference (p˂0.05) between IRIS stage 1 compared to Stages 3 and 4.

**Conclusions and Clinical Importance:** uClusterin and uCysB might identify dogs with active renal injury that could lead to kidney disease progression.

## 319 Abstract NU27

### 319.1 Urinary Cystatin B Differentiates Progressive versus Stable Stage I Chronic Kidney Disease in Dogs

#### 319.1.1 **Gilad Segev** ^1^; Shelly Vaden^2^, DVM, PhD, DACVIM (SAIM); Sheri Ross^3^, DVM, PhD, DACVIM (SAIM); Cedric Dufayet^3^, DVM; Leah Cohn^4^, DVM, PhD, DACVIM (SAIM); Giosi Farace^5^; Donald Szlosek^5^, MPH; Zenhwa Ouyang^5^, VMD, PhD, MS, MSE; Sarah Peterson^5^, MD, PhD; Melissa Beall^5^, DVM, PhD; Murthy Yerramilli^5^, PhD; David Polzin^6^, DVM, PhD, DACVIM (SAIM); Larry Cowgill^3^, DVM, PhD, DACVIM (SAIM)

##### 319.1.1.1 ^1^Koret School of Veterinary Medicine, Hebrew University, Jerusalem, Israel; ^2^North Carolina State University; ^3^Veterinary Medical Center, University of California, San Diego; ^4^University of Missouri; ^5^IDEXX Laboratories, Inc.; ^6^University of Minnesota

**Background:** Early identification of dogs with progressive versus stable chronic kidney disease (CKD) might afford opportunity for interventions that would slow progression. However, currently there is no surrogate biomarker to reliably predict CKD progression.

**Hypothesis/Objectives:** Urinary cystatin B (uCysB), a novel kidney injury biomarker, has potential to predict progression of early CKD.

**Animals:** Twenty dogs from four university centers with IRIS Stage I CKD (Stage I), with IDEXX SDMA® up to 17 μg/dL and no systemic comorbidities, and 107 clinically healthy staff‐owned dogs (control) from a fifth university center.

**Methods:** A multicenter prospective longitudinal study was conducted between 2016 and 2021 to assess the pattern of expression of uCysB in Stage I CKD and control dogs. Dogs were followed to a maximum of 3 years. Stage I was subgrouped as stable or progressive CKD by 1/IDEXX SDMA® slope, calculated from a minimum of three monthly timepoints during the initial 120‐day period. Dogs with slope above or below ‐0.0007 week *dL/μg were classified as stable or progressive, respectively, as we have shown previously. Mixed effects modeling was used to assess the association between uCysB and progression rate.

**Results:** Estimated uCysB was 16 ng/mL for control, 23 ng/mL for stable CKD, and 204 ng/mL for progressive CKD predicting active ongoing injury (Figure 1, p<0.05, dashed lines represent 95% confidence intervals).

**Figure:**
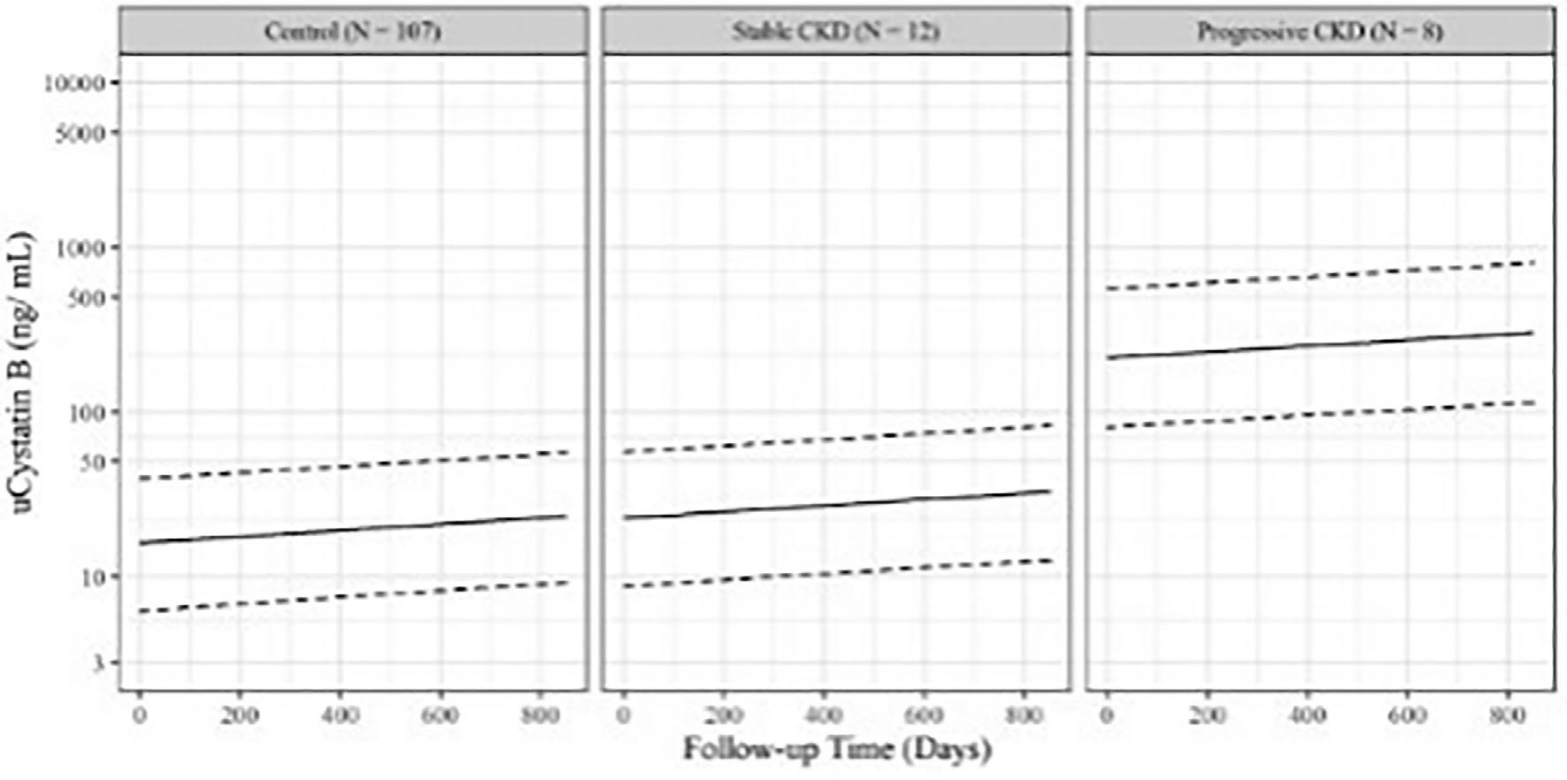
Figure 1

**Conclusions and Clinical Importance:** uCysB differentiated stable versus progressive Stage I CKD. Early discrimination of dogs with progressive CKD creates an opportunity for interventions to arrest progression and improve patient management.

## 320 Abstract NU28

### 320.1 Mineralocorticoid Receptor Expression and Activation in Feline Chronic Kidney Disease and Associations with Disease Progression

#### 320.1.1 **Sarah E. Spencer** ^1^; Jonathan Elliott^2^; Caroline Wheeler‐Jones^3^

##### 320.1.1.1 ^1^Royal Veterinary College; ^2^Professor, Comparative Biological Sciences, Royal Veterinary College; ^3^Professor, Comparative Biological Sciences, Royal Veterinary College

**Background:** Chronic kidney disease (CKD) frequently causes death in ageing cats. Mineralocorticoid receptor (MR) activation contributes to hypoxia‐induced kidney injury in humans and laboratory animals.

**Objectives:** To quantify kidney MR and serum glucocorticoid‐regulated kinase‐1 (SGK‐1) mRNA expression and explore associations with hypoxia‐induced profibrotic genes, clinicopathological and histopathological characteristics in feline CKD.

**Animals:** Fifty‐one client‐owned cats: 14 geriatric (≥9 years) non‐azotemic (Controls), 37 with IRIS CKD stage 2–4 (CKD). Disease progression analysis in 31 cats (14 progressive [CKD‐P], 17 non‐progressive [CKD‐NP]).

**Methods:** Cross‐sectional and longitudinal study. Kidney tissue obtained post‐mortem underwent histopathological scoring and reverse transcription‐quantitative PCR to measure MR, SGK‐1, transforming growth factor‐β1 (TGF‐β1), hypoxia‐inducible factor‐1α (HIF‐1α) and collagen‐1α1 (COL1A1) transcripts. Relative mRNA levels were compared between groups (Control vs. CKD; Control vs. CKD‐P vs. CKD‐NP). Associations between transcript levels and histopathological and clinicopathological characteristics were explored.

**Results:** Relative SGK‐1 expression was lower in CKD cats versus controls and in CKD‐P versus controls (Table 1). MR and SGK‐1 expression (Table 2) were positively correlated with each other and hypoxia‐related profibrotic genes. SGK‐1 expression negatively correlated with serum creatinine concentration and interstitial fibrosis score. MR expression negatively correlated with serum magnesium concentration. Interstitial inflammation score positively correlated with TGF‐β1 (r=0.349, P=0.025) and COL1A1 expression (r=0.3297, P=0.022), and glomerular score with TGF‐β1 (r=0.340, P=0.030), HIF‐1α (r=0.317, P=0.028) and COL1A1 (r=0.339, P=0.018) expression.

**Table:** Table 1. Differences in relative renal SGK‐1 expression between disease groups

**Cross‐sectional analysis**	**Controls (n=14)** **[median, range]**	**CKD (n=37)** **[median, range]**		**P value**
	1.075(0.155‐3.80)	0.570(0.153‐5.21)		**0.015**
Longitudinal analysis	Controls (n=14) [median, range]	CKD‐P (n=14) [median, range]	CKD‐NP (n=17) [median, range]	P value
	1.070^a^ (0.155–3.80)	0.436^a^ (0.153–3.65)	0.651 (0.162–5.21)	^**a**^ **0.043**

**Table:** Table 2. Correlations between relative renal MR, SGK‐1 and other gene expression and variables in 51 geriatric cats

Genes and variables of interest	MR expression		SGK‐1 expression	
	Spearman's correlation	P value	Spearman's correlation	P value
SGK‐1 expression	0.616	<0.001	‐	‐
TGF‐β1 expression	0.431	0.004	0.389	0.009
HIF‐1α expression	0.433	0.002	0.504	<0.001
COL1A1 expression	0.433	0.002	0.171	0.231
Serum creatinine	‐0.150	0.298	‐0.290	0.040
Serum magnesium	‐0.599	0.009	‐0.261	0.296
Interstitial fibrosis score	‐0.242	0.109	‐0.320	0.030

**Conclusions and Clinical Importance:** SGK‐1 may play a protective role in feline CKD. MR expression is associated with hypoxic profibrotic pathways and serum magnesium concentration in feline CKD.

## 321 Abstract NU29

### 321.1 Clinical Effects of Asymptomatic *E. coli* Administration Compared to Oral Antimicrobials for Canine Recurrent UTI

#### 321.1.1 **Jodi L. Westropp** ^1^; Gilad Segev^2^, DVM, ECVIM‐CA; Jonathan Dear^1^, DVM, MS, DACVIM; Jane Sykes^3^, BVSc (Hons), PhD, DACVIM; Jully Pires^1^; David Klumpp^4^, PhD; Anthony Schaeffer^5^, MD

##### 321.1.1.1 ^1^School of Veterinary Medicine, University of California‐Davis, Davis, CA, USA; ^2^Professor, Koret School of Veterinary Medicine; ^3^Professor, School of Veterinary Medicine, University of California‐Davis, Davis, CA, USA; ^4^Professor, Feinberg School of Medicine, Northwestern University; ^5^Feinberg School of Medicine, Northwestern University

**Background:** Asymptomatic *E. coli* 212 (ASB 212) has shown promise in dogs with recurrent UTI (rUTI)

**Hypothesis:** Administration of ASB 212 is noninferior to antimicrobial therapy for clinical rUTI.

**Animals:** Client‐owned dogs with rUTI.

**Methods:** Multicenter randomized noninferiority trial. Dogs were randomized to Group 1 (amoxicillin‐clavulanic acid; 13.75–19 mg/kg PO q12h for 7d) or Group 2 (intravesical administration of ASB 212; repeated if necessary). Owners completed a questionnaire regarding their dogs’ signs; data were analyzed using the Farrington‐Manning test.

**Results:** Nine dogs were enrolled in Group 1 and 11 in Group 2. The median (range) clinical scores at day 0, 7 and 14 for Group 1 was 8 (3–17), 3 (0–6) and 2 (1–7), respectively. Group 2 scores were 8 (3–14), 4 (1–15) and 3 (1–15), respectively. After day 7, two Group 2 dogs were failures due to early withdrawal; 1 was lost to follow up. Five (55%) Group 1 dogs and 8 (72%) Group 2 dogs had >65% reduction in their clinical score on day 7 compared to baseline. For day 7, Group 2 was not inferior to Group 1 according to the selected margin of noninferiority (20%; P=0.04). Five of 9 dogs in Group 2 had *E. coli* cultured on day 7; bacterial strain typing is pending. No major adverse events were reported.

**Conclusion and Clinical Importance:** Clinical outcomes in some dogs treated with intravesical ASB 212 were positive. The biotherapeutic was easy to administer.

## 322 Abstract NU30

### 322.1 Untargeted Metabolomic Profiling of Serum from Cats with Chronic Kidney Disease

#### 322.1.1 **Stacie Summers** ^1^; Jessica Quimby^2^, DVM, PhD, DACVIM (SAIM); Jenessa Winston^2^, DVM, PhD, DACVIM (SAIM)

##### 322.1.1.1 ^1^Oregon State University; ^2^The Ohio State University

**Background:** Evaluation of the metabolome could discover novel pathophysiologic pathways and identify potential biomarkers of disease. To date, characterization of the serum metabolome of client‐owned cats with chronic kidney disease (CKD) has not been reported.

**Objective:** To investigate differences in serum metabolome among client‐owned healthy adult and mature/senior cats, and cats with CKD.

**Animals:** Eight healthy adult cats (<8 years), 17 healthy mature/senior cats (≥8 years), and 30 cats with CKD (International Renal Interest Society stage 1 [3 cats], stage 2 [14 cats], stage 3 [9 cats], and stage 4 [4 cats] CKD).

**Methods:** Cross‐sectional prospective study. Serum metabolome was analyzed with an untargeted metabolomics approach using ultrahigh performance liquid chromatography‐tandem mass spectrometry. Stage 1 and 2 cats and stage 3 and 4 cats were grouped for statistical analysis. Significant difference defined as P<0.05.

**Results:** Eight hundred and thirty metabolites were identified. Principle component analysis plots of the data showed clear separation between stage 1/2 CKD cats, stage 3/4 CKD cats and healthy cats, with overlap between healthy adult and senior cats. Sixty‐three metabolites significantly differed between healthy adult and senior cats and 410 metabolites were significantly different between healthy cats and CKD cats. Significant alterations in metabolites associated with amino acid, nucleotide, vitamin, and carbohydrate metabolism were found in the CKD cats compared to healthy cats.

**Conclusions and Clinical Importance:** The serum metabolome varies among healthy adult cats, mature/senior cats, and CKD cats. In healthy cats, age appears to have minimal impact on the metabolic profile.

## 323 Abstract NU31

### 323.1 Reduced Antioxidant System and Increased Inflammation in Canines with Chronic Kidney Disease

#### 323.1.1 **Selena K. Tavener** ^1^; Dennis Jewell^2^, PhD; Kiran Panickar^1^, PhD

##### 323.1.1.1 ^1^Science and Technology Ctr, Hill's PNC; ^2^Kansas State University

**Background:** Inflammation and oxidative stress (OS) contribute to the progression of CKD. Increased levels of reactive oxygen species (ROS) and reactive nitrogen species (RNS), together with reduced anti‐oxidative systems, are associated with impaired renal function.

**Hypothesis:** Antioxidant enzymes that protect against renal cellular damage are diminished in canines with CKD.

**Animals:** Canines housed in Hill's animal colony and diagnosed with naturally occurring CKD showed higher circulating BUN and creatinine (n=7; 7.6–15.11 yr). Controls had no known renal problems (n=13; 7.3–16.4 yr).

**Methods:** RT^2^ Profiler^™^ PCR Array Dog Oxidative Stress panel was used to assess 84 OS and inflammation genes in blood. Data were analyzed using the ΔΔCt method normalized to RPLP1.

**Results:** When comparing dogs with CKD to controls, there was a significant up‐regulation in CCL5 (p<0.05), as well as increases in eosinophil peroxidase, and DHCR24 (both ns). Down‐regulation was observed in EPX orthologous genes myeloperoxidase and lactoperoxidase (both ns). There was also a decrease in glutathione peroxidase 5, glutathione peroxidase 6, glutathione peroxidase 7, glutathione peroxidase 8, superoxide dismutase 2, superoxide dismutase 3, NAD(P)H dehydrogenase [quinone] 1, and apolipoprotein (all ns). In response to OS, a possible compensatory increase in antioxidant enzymes including PRDX2 and PRDX4 was observed in CKD (p<0.05 and ns, respectively).

**Conclusions and Clinical Importance:** Our results indicate a decline in several important antioxidant enzymes in CKD dogs. Gene expression provides mechanistic insights for reducing OS and inflammation‐associated signaling and may serve as important targets for nutritional interventions.

## 324 Abstract NU32

### 324.1 Evaluation of Novel Renal Injury Markers in Dogs with Ehrlichiosis

#### 324.1.1 **André N. Vieira Le Sueur** ^1^; Adriana Lopes de Souza^2^, MV; Regina Takahira^2^; Alessandra Melchert^2^, MV, MSc, PhD; Michael Coyne^3^, DVM, PhD; Rachel Murphy^3^; Donald Szlosek^3^; Sarah Peterson^3^, MD, PhD; Antônio Paes^2^, MV, MSc, PhD; Priscylla Chalfun Guimarães‐Okamoto^2^, MV, MSc, PhD

##### 324.1.1.1 ^1^Translational Veterinary Medicine, KURI Institute; ^2^School of Veterinary Medicine and Animal Science, São Paulo State University, UNESP; ^3^IDEXX Laboratories Inc.

**Background:** Canine Monocytic Ehrlichiosis (CME) has been observed to affect the kidneys. Currently, the recognition of renal injury is through the nonspecific biomarker serum creatinine (sCr). Novel markers of renal injury such as urinary Clusterin (uClust) and urinary Cystatin B (uCysB) may increase our understanding of the relationship between Ehrlichiosis and renal cellular injury.

**Hypothesis/Objectives:** To evaluate novel renal injury biomarkers in dogs with CME.

**Animals:** Twenty healthy dogs were enrolled in the control group (CG), and 16 dogs naturally infected with Ehrlichia canis were included in the Ehrlichia Group (EG).

**Methods:** All dogs were followed for 45 days. EG dogs were treated with doxycycline twice daily for the first 30 days. Urine and serum were collected at: 0, 0.5, 1, 15, 30, and 45 days after start of treatment. Urine concentrations of uClust and uCysB were determined using a research ELISA. A linear mixed model was used to estimate population mean of renal injury markers with patient as the random effect, and day and treatment as fixed effects.

**Results:** EG was observed to have higher uClust values compared to CG (estimated population mean EG: 213 ng/dL vs. CG: 84 ng/dL, P<0.001). EG was observed to have higher uCysB values compared to CG (estimated population mean EG: 248 ng/dL vs. CG: 38 ng/dL, P<0.001).

**Conclusion and Clinical Importance:** Increases in uCysB and uClust suggest the presence of renal injury and a possible mechanism for the observed predisposition to chronic kidney disease in dogs with Ehrlichiosis.

## 325 Abstract NU33

### 325.1 C‐Reactive Protein and Canine Ehrlichiosis: A New Clinical Perspective

#### 325.1.1 **André N. Vieira Le Sueur** ^1^; Maria Reis Castiglioni^2^, MV, MSc, PhD; Maria Santos Breda^2^, MV, MSc; Alessandra Melchert^2^, MV, MSc, PhD; Fabiana de Souza^2^, MV, MSc, PhD; Michael Coyne^3^, DVM, PhD; Rachel Murphy^3^; Donald Szlosek^3^; Antonio Paes^2^, MV, MSc, PhD; Priscylla Chalfun Guimarães‐Okamoto^2^, MV, MSc, PhD

##### 325.1.1.1 ^1^Translational Veterinary Medicine, KURI Institute; ^2^School of Veterinary Medicine and Animal Science, São Paulo State University, UNESP; ^3^IDEXX Laboratories Inc.

**Background:** C‐reactive protein (CRP) is commonly utilized in dogs and humans as an inflammatory response biomarker. Canine monocytic ehrlichiosis (EMC) is a clinically important, global vector‐borne disease caused by Ehrlichia canis.

**Hypothesis/Objectives:** To evaluate the performance of CRP during the diagnosis, treatment, and monitoring of dogs naturally infected with E. canis.

**Animals:** Nineteen healthy dogs were enrolled in the control group (CG), and 16 dogs naturally infected with E. canis were included in the Ehrlichia Group (EG).

**Methods:** All dogs were followed for 45 days. EG dogs were treated with doxycycline twice daily for the first 30 days. Hematology, biochemistry profile, and CRP concentrations were determined on days 0, 0.5, 1, 15, 30 and 45. A linear mixed model was used to estimate population mean of each parameter with patient as the random effect, and day and treatment as fixed effect.

**Results:** In the EG, CRP concentrations were observed to have a negative log‐linear relationship with treatment time (P<0.001). Additionally, serum creatinine demonstrated a negative log‐linear relationship with CRP (P<0.002). The EG was observed to have higher CRP concentrations and higher CRP‐sCr relationship compared to the CG (P<0.001, P<0.006; respectively). Furthermore, globulin concentrations and UPC were observed to have a positive log‐linear relationship with CRP (P<0.008, P<0.001; respectively).

**Conclusions and Clinical Importance:** CRP concentrations decreased significantly with treatment. CRP is a useful biomarker in monitoring the response to treatment of dogs naturally infected by E. canis.

## 326 Abstract NU34

### 326.1 Effect of Tamsulosin on Urethral Tone in Healthy Male Cats

#### 326.1.1 **Zhe (Alice) Wang**; Amanda Diaze; Rachael Isdale; Patricia Franco; Kristin Kofron; Michael Lappin, DVM, DACVIM (SAIM), PhD

##### 326.1.1.1 Colorado State University

**Background:** Alpha‐adrenergic antagonists are commonly used to prevent recurrent urethral obstruction in cats with mixed reports of efficacy. No data on tamsulosin use in cats is available.

**Objectives:** To determine whether tamsulosin can effectively decrease urethral tone without causing significant hypotension in male cats.

**Animals:** 5 young healthy adult male cats from a research colony.

**Methods:** Prospective pilot study. Cats were administered tamsulosin 0.1 mg per cat PO SID for 10 days. UPP and blood pressure were performed on baseline, day 4 and day 10 of treatment. Maximum urethral pressure (MUP) for the prostatic and penile urethra, functional urethral length (FPL), functional area (FA) and systolic blood pressures were recorded.

**Results:** Significant changes in blood pressures on day 4 (116.2±8.0 vs; P=0.43) and on day 10 (108±19.0, P=0.97) compared to day 0 (104.7±24) were not detected. The 5 Fr urinary catheters could only be passed in 2 cats on days 4 and 10. On Day 4, 52.6% and 64.3% decreases in the pre‐prostatic and prostatic FA were noted in the 2 cats. On day 10, a 21.5% decrease and 27.0% increase in this FA were noted. Increases in prostatic MUP (43.0%, 53.8%) were noted in the two cats on day 10.

**Conclusion and Clinical Importance:** Tamsulosin can potentially decrease urethral tone without causing significant hypotension but repeated catheterization could override the effect. Further studies with a smaller catheter and additional cats are ongoing.

## 327 Abstract NU35

### 327.1 Serum Concentrations of Leptin and Adiponectin in Dogs with Chronic Kidney Disease

#### 327.1.1 **Taesik Yun** ^1^; Dongjoon Choi^2^, DVM, MS; Dohee Lee^2^, DVM, MS; Yoonhoi Koo^2^, DVM, MS; Yeon Chae^2^, DVM, MS; Mhan‐Pyo Yang^2^, DVM, MS, PhD; Byeong‐Teck Kang^2^, DVM, MS, PhD; Hakhyun Kim^2^, DVM, MS, PhD

##### 327.1.1.1 ^1^College of Veterinary Medicine, Chungbuk National University; ^2^Laboratory of Veterinary Internal Medicine, College of Veterinary Medicine, Chungbuk National University

**Background:** An imbalance in adipokines is associated with the progression of chronic kidney disease (CKD) in humans. However, alterations in adipokines in dogs with CKD remain unclear.

**Objectives:** This study aimed to examine whether adipokine concentrations differed between healthy dogs and dogs with CKD and to determine the correlation between circulating adipokine concentrations and CKD severity in dogs.

**Animals:** Twenty dogs with CKD and 10 healthy dogs.

**Methods:** In this cross‐sectional study, serum concentrations of leptin, adiponectin, interleukin (IL)‐6, IL‐10, IL‐18, and tumor necrosis factor (TNF)‐α were measured in healthy dogs and dogs with CKD, which were classified according to the International Renal Interest Society (IRIS) guidelines.

**Results:** Serum leptin concentrations were positively correlated with systolic arterial blood pressure (*P*=0.03, r^2^=0.17), creatinine concentrations (*P*=0.03, r^2^=0.15), and symmetric dimethylarginine (SDMA) concentrations (*P*<0.001, r^2^=0.53). Median (range) serum adiponectin concentrations were significantly different between proteinuric dogs (13.95 [6.4–22.1] ng/mL) and borderline or non‐proteinuric dogs (20.25 [14.9–45.8] ng/mL; *P*=0.01). Serum IL‐6 (43.27 [24.30–537.30] vs. 25.63 [6.83–61.03] pg/mL; *P*=0.02), IL‐18 (25.98 [11.52–280.55] vs. 10.77 [3.53–38.45] pg/mL; *P*=0.01), and TNF‐α concentrations (11.44 [8.54–38.45] vs. 6.105 [3.97–30.68] pg/mL; *P*=0.02) were significantly higher in proteinuric dogs than in borderline or non‐proteinuric dogs.

**Conclusions and Clinical Importance:** Circulating leptin and adiponectin concentrations might be associated with CKD severity and proteinuria severity in dogs with CKD, respectively.

## 328 EQUINE

## 329 Abstract E24

### 329.1 Effect of Phenylbutazone Administration on Insulin and Glucose Dynamics in Horses

#### 329.1.1 **Kate L. Kemp** ^1^; François‐René Bertin^2^, DVM, MS, PhD, DACVIM (LAIM)

##### 329.1.1.1 ^1^The University of Queensland; ^2^Associate Professor, School of Veterinary Science, The University of Queensland

**Background:** Phenylbutazone is the most prescribed compound for the management of hyperinsulinemia‐associated laminitis (HAL); however, it has been shown in people that, non‐steroidal anti‐inflammatory drugs (NSAIDs) increased insulin secretion suggesting that phenylbutazone administration could result in hyperinsulinemia and exacerbate HAL.

**Hypothesis/Objectives:** Phenylbutazone administration would exacerbate insulin dysregulation (ID) in horses.

**Animals:** Nineteen University‐owned horses (12 mares and 7 geldings) including 10 diagnosed with ID and 9 controls.

**Methods:** A randomized crossover design was used with horses receiving intravenous phenylbutazone (4.4 mg/kg SID) or placebo. After 9 days of treatment, horses underwent an oral glucose test (OGT). Following a 10‐day washout period, horses received the alternative treatment for 9 days, and another OGT was performed. Insulin was measured on the IMMULITE 1000 and glucose by handheld glucometer. Changes in insulin and glucose concentrations were analysed with a linear mixed effect model, with P<0.05 considered significant.

**Results:** There were significant effects of phenylbutazone, ID status and phenylbutazone x ID status on glucose concentrations (P=0.007, 0.0006 and 0.04, respectively) with significantly lower blood glucose concentrations in ID horses receiving phenylbutazone (P<0.0001). Despite a significant effect of ID status on insulin concentration, no effect of phenylbutazone was detected (P=0.0001 and 0.3, respectively).

**Conclusions and Clinical Importance:** Phenylbutazone decreases blood glucose concentration in ID horses; however, this effect would not be mediated by an increased insulin secretion suggesting more complex insulin and glucose dynamics in ID horses receiving NSAIDs. This effect could have diagnostic and therapeutic implications.

## 330 Abstract E25

### 330.1 Prospective Assessment of Clinical Signs and ACTH Concentrations in Horses Transitioning to PPID

#### 330.1.1 **Naomi C. Kirkwood** ^1^; Kristopher Hughes^1^, BVSc (Hons1), FANZCVS (Equine Medicine), DECEIM, AssocACVIM; Allison Stewart^2^, BVSc (Hons 1), MS, DACVIM, DACVECC

##### 330.1.1.1 ^1^Charles Sturt University; ^2^Equine Medicine Specialist, University of Queensland

**Background:** Poor recognition of subtle clinical signs and equivocal ACTH concentrations makes early diagnosis of PPID in horses difficult. Sensitivity is improved by measurement of thyrotropin releasing hormone (TRH)‐stimulated ACTH concentrations compared to basal ACTH concentrations. Progressive clinical signs and corresponding basal and TRH‐stimulated ACTH concentrations in horses transitioning to PPID over time have not been documented.

**Hypothesis/Objectives:** To describe clinical signs and ACTH concentrations associated with transition from subclinical to clinical PPID.

**Animals:** Seven university‐owned horses of various breeds, >12 years of age with ACTH concentrations equivocal for PPID (based on locally derived, seasonally‐adjusted diagnostic‐cut off values (DCOV)) were selected. No horse had been administered pergolide mesylate (Prascend) in the previous 12 months.

**Methods:** A prospective cohort study with measurement of basal and TRH‐stimulated ACTH concentrations and recording of clinical signs from October 2017 to November 2021.

**Results:** In two horses, severe hypertrichosis developed, and basal ACTH concentrations increased over the study period. Both had 1/11 basal ACTH concentrations below the DCOV in 2018 and subsequently all basal ACTH concentrations were increased. One horse was treated with pergolide which normalised basal ACTH concentrations. Five horses with intermittent, mild hypertrichosis had TRH‐stimulated ACTH concentrations greater than the DCOV, particularly in late summer/early fall and only occasional increased basal ACTH concentrations.

**Conclusions and Clinical Importance:** Horses with early PPID can have equivocal test results. Post‐TRH ACTH concentrations in late summer/early fall identified most transitional cases.

## 331 Abstract E26

### 331.1 Seasonal Effects of Plasma ACTH in Horses and Donkeys Residing Near the Equator

#### 331.1.1 **Erik W. Peterson** ^1^; Cynthia Xue^2^, DVM; Rachel Lemcke^3^, BS, MS; Jamie Pribyl^4^; Steven Grubbs^5^, DVM, PhD, DACVIM

##### 331.1.1.1 ^1^Ross University School of Veterinary Medicine; ^2^Assistant Professor, Equine Internal Medicine, Ross University School of Veterinary Medicine; ^3^Owner, Amwell Data Services; ^4^Boehringer‐Ingelheim; ^5^Professional Services Veterinarian, Boehringer‐Ingelheim

**Background:** Pars intermedia activity in horses follows a robust seasonal rhythm but investigation of seasonal plasma ACTH biorhythm near the equator is limited. Additionally, little information about the effect of photoperiod and season on plasma ACTH exists in donkeys.

**Objective:** To evaluate non‐fall and fall basal ACTH concentrations in horses and donkeys residing near the equator.

**Animals:** Fifteen healthy university and client‐owned horses (Group 1) and thirty healthy university‐owned donkeys (Group 2) located at 17.3578°N/62.7830°W. All animals were three years or older. There were no restrictions on breed or sex.

**Methods:** Blood was collected in EDTA tubes from all animals (Groups 1 and 2) for measurement of basal ACTH concentrations within three days during the non‐fall (March 2021) and fall (September 2021) seasons. Plasma was separated immediately from centrifuged samples and stored at ‐80°C prior to submission to the Animal Health Diagnostic Center (Cornell University) for analysis.

**Results:** Non‐fall basal ACTH levels averaged 20.29±7.73 pg/mL and 26.76±16.02 pg/mL for horses and donkeys, respectively. Fall ACTH levels rose to 94.31±94.15 pg/mL and 85.47±36.82 pg/mL for horses and donkeys, respectively, demonstrating a statistically significant autumnal seasonal increase averaging 74.02±6.68 pg/mL and 58.71±42.60 pg/mL (P<0.0001), respectively.

**Conclusions:** Horses and donkeys near the equator demonstrate autumnal increases of basal ACTH concentrations, corroborating with similar studies performed at higher latitudes. Increased ACTH in equids at latitudes approaching 0° during late summer or autumn should be interpreted with caution in the absence of seasonally and geographically derived species‐specific reference ranges.

**Seasonal effects of resting ACTH in horses residing near the Equator**

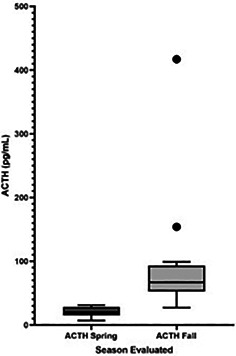

**Seasonal effects of resting ACTH in donkey residing near the Equator**

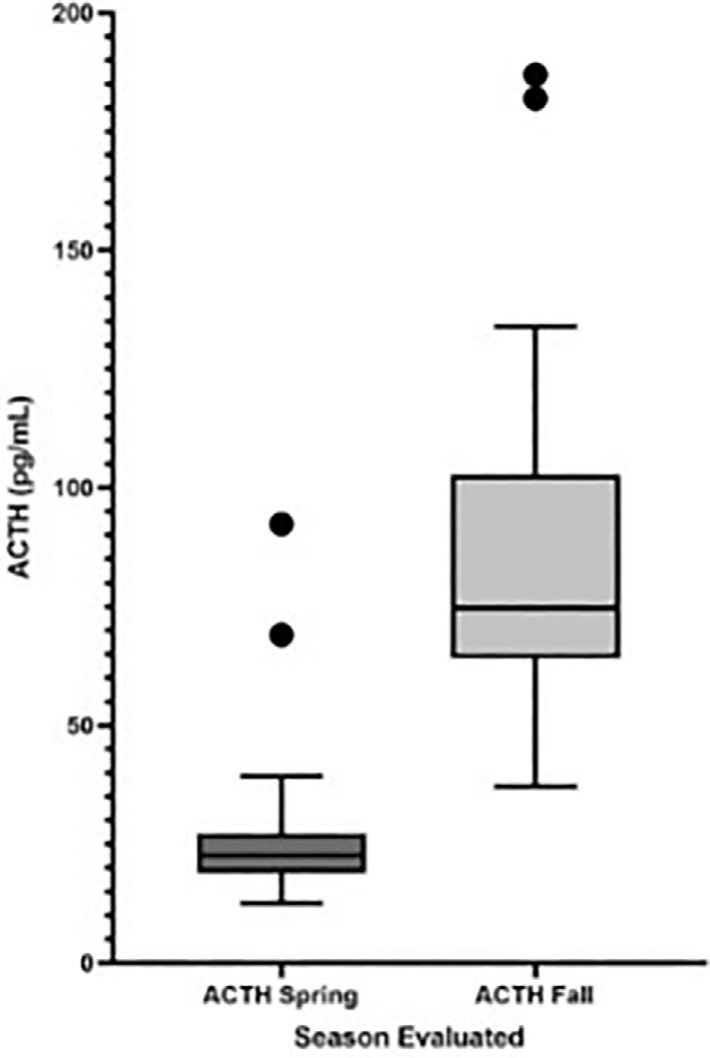

## 332 Abstract E27

### 332.1 Effects of Latitude, Age and Season on Equine Adrenocorticotropic Hormone Concentrations in the United States

#### 332.1.1 **Toby L. Pinn‐Woodcock** ^1^; Sebastian Gonzalo Llanos Soto^2^; Renata Ivanek^2^; Erin Goodrich^2^; Elisha Frye^1^; Joseph Guiness^2^; Amy Wells^1^; Stephen Lamb^1^; Ned Place^1^

##### 332.1.1.1 ^1^Animal Health Diagnostic Center, Cornell University; ^2^Cornell University

**Background:** The influence of latitude on seasonal variation in equine adrenocorticotropic hormone (ACTH) concentration is not well described in the United States, thereby confounding diagnostic interpretation in horses with pituitary pars intermedia dysfunction.

**Objective:** The goal of this study was to determine how seasonal variation in plasma ACTH concentrations of healthy horses is affected by latitude in the United States.

**Animals:** Healthy research and privately‐owned horses from 9 locations were classified into three cohorts by latitude zone: northern (n=36), central (n=85), and southern (n=33).

**Methods:** Cohort study in which plasma ACTH was measured once to twice monthly by chemiluminescence immunoassay. Horses with multiple outlier ACTH concentrations were identified through the robust regression and outlier removal method. ACTH upper reference limits were calculated following Clinical Laboratory and Standards Institute guidelines. Linear mixed effect regression was used to identify factors associated with ACTH concentrations.

**Results:** The ACTH upper reference limits were higher during July–November as compared to December–June in all latitude zones (p<0.0001), but no significant differences were detected between latitude zones. Horses >15 years exhibited higher ACTH upper reference limits during the late summer/fall compared to younger horses (p<0.0001).

**Conclusions and Clinical Importance:** These results indicate that plasma ACTH concentrations are elevated in healthy horses during late summer/fall throughout the latitudinal range of the contiguous U.S. Age is likely to be an important factor when interpreting ACTH results during the months of seasonal elevation. Study limitations included small sample size.

## 333 Abstract E28

### 333.1 Comparison of a Newly Developed Glycemic Pellets Challenge with the Oral Sugar Test in Horses

#### 333.1.1 **Kristen Thane** ^1^; Johanna Sonntag^2^; Dania Reiche^2^; Nicholas Frank^1^, DVM, PhD, DACVIM (LAIM)

##### 333.1.1.1 ^1^Tufts Cummings School of Veterinary Medicine; ^2^Boehringer Ingelheim Vetmedica GmbH

**Background:** Testing for insulin dysregulation (ID) in horses is commonly performed to guide management and therapeutic strategies.

**Objective:** To evaluate a newly‐developed glycemic pellet challenge (GPC) and compare results to those from the oral sugar test (OST).

**Animals:** Twenty‐four adult horses with unknown insulin status.

**Methods:** Subjects underwent GPC (0.5 g glycemic carbohydrates/kg bodyweight) and OST (0.15 mL corn syrup/kg bodyweight) 7 days apart. Feed was withheld prior to testing and blood samples were collected at T0, T60, T120, and T180 for the GPC and at T0, T60, and T90 for the OST. Blood glucose (BG) was measured with a point‐of‐care glucometer. Insulin was measured by radioimmunoassay. Comparisons were made using nonparametric tests and linear regression. Bland‐Altman plots demonstrated bias. Significance was set at P=0.05.

**Results:** Eighteen horses consumed >85% of the GPC pellets offered and had acceptable OST results. Maximum glucose and insulin concentrations were significantly higher (P=0.02 and P=0.007, respectively) for the GPC when compared with the OST. Time to maximum insulin concentration (Tmax[ins]) varied within and between tests and was not correlated with the time horses took to consume pellets (P=0.28).

**Conclusions:** BG and insulin concentrations increase during the GPC and reach higher concentrations than observed with the OST. Both tests detect differences in BG and insulin responses among horses, and the GPC may be used to diagnose ID in horses once diagnostic thresholds are established. Tmax[ins] varied for the GPC and OST, highlighting the importance of collecting multiple blood samples to capture diagnostic changes in insulin concentration.

## 334 Abstract E29

### 334.1 Effect of AMPK Agonists on Hepatic Lipid Content in Horses with Experimentally‐Induced Insulin Dysregulation

#### 334.1.1 **Kathryn Timko**; Laura Hostnik; Mauria Watts; Chiaming Chen; Adam Bercz; Ramiro Toribio; James Belknap; Teresa Burns

##### 334.1.1.1 The Ohio State University

**Background:** Insulin dysregulation (ID), a hallmark of equine metabolic syndrome (EMS), is associated with dyslipidemias (hypertriglyceridemia, hyperleptinemia). Similarly, metabolic syndrome in humans contributes to the development of non‐alcoholic fatty liver disease (NAFLD), increasing the risk of cirrhosis or neoplasia. 5’‐Adenosine‐monophosphate‐activated protein kinase (AMPK) agonists reduce liver lipid content in animal models.

**Hypothesis/Objectives:** Evaluate the hepatic lipid content in response to AMPK agonists (metformin [MET], aspirin [ASP], and combination [MET/ASP]) in horses with experimentally‐induced ID.

**Animals:** Fourteen adult, light‐breed research colony horses.

**Methods:** Randomized, prospective clinical trial. Dexamethasone was administered (0.08 mg/kg PO SID) for 7 days to induce ID, then MET (30 mg/kg PO q 12 h, n=7) or ASP (20 mg/kg PO q 24 h, n=7) were administered with dexamethasone for 7 days. Seven horses then received MET/ASP with dexamethasone for 7 additional days. Liver biopsies were obtained at four timepoints: baseline, ID, ID + monotherapy (ASP or MET), and ID + combination therapy (ASP/MET) and stained with hematoxylin and eosin. The samples were evaluated by light microscopy and assigned a steatosis score based on percentage of vacuolar change: 0; <5%, 1; 6–33%, 2; 34–66%, 3; >67%.

**Results:** Steatosis scores were increased compared to baseline at the ID and ID + monotherapy timepoint (P<0.01). The steatosis score at ID + combination therapy timepoint was no longer significantly increased compared to baseline (P=0.3), suggesting a reduction in hepatic lipid content.

**Conclusions:** Combination therapy with metformin and aspirin may be useful for treating dyslipidemias in equids with metabolic syndrome.

## 335 Abstract E30

### 335.1 Effect of High‐Carbohydrate Feeding and Corticosteroid Administration on Lipid Content of Equine Liver

#### 335.1.1 **Kathryn Timko**; Laura Hostnik; Mauria Watts; Chiaming Chen; Adam Bercz; Ramiro Toribio; James Belknap; Teresa Burns

##### 335.1.1.1 The Ohio State University

**Background:** Nonalcoholic fatty liver disease (NAFLD), a sequela of human metabolic syndrome, is associated with excess lipid accumulation in the liver. Insulin dysregulation and dyslipidemias are also a hallmark of equine metabolic syndrome and may contribute to similar pathologies.

**Hypothesis/Objectives:** Evaluate the effect of a high non‐structural carbohydrate (NSC) diet and corticosteroid administration on liver lipid content in equids.

**Animals:** 20 mixed‐breed healthy ponies and 14 light‐breed healthy horses from research herds.

**Methods:** Randomized clinical trial. Mixed‐breed ponies were allocated into four groups based on body condition score (BCS) (Lean; [BCS]<4, obese; BCS>7) and diet (Control; NSC ~6%, High NSC; NSC ~42%): Lean Control (n=5), Lean High NSC (n=6), Obese Control (n=4), Obese High NSC (n=5) and fed their respective diet for 7d. Liver samples were collected after the feeding protocol and stained with hematoxylin and eosin (H&E). Liver biopsies were obtained from 14 light‐breed horses before and after dexamethasone administration (0.08 mg/kg PO q 24 h) for 7d and stained with H&E. Liver samples were evaluated by light microscopy using a NAFLD scoring system adapted from humans: Steatosis (0–3), microvesicular steatosis (0–1), ballooning injury (0–2), inflammation (0–3), steatosis location (0–3).

**Results:** The Lean and Obese High NSC diet ponies had higher NAFLD scores compared to control fed obese ponies (P=0.01, 0.04, respectively). The NAFLD scores following dexamethasone treatment were increased compared to baseline (P<0.001).

**Conclusions:** A high NSC diet and dexamethasone administration can induce histopathologic changes consistent with NAFLD in equids within 7 days.

## 336 Abstract E31

### 336.1 Effects of Dopamine Suppression on Postprandial Glucose and Insulin Responses to Glucose Feeding in Horses

#### 336.1.1 **Nicolas C. Galinelli** ^1^; Nicholas Bamford^1^; Madison Erdody^1^; Patricia Harris^2^; Martin Sillence^3^; Simon Bailey^1^

##### 336.1.1.1 ^1^Melbourne Veterinary School, The University of Melbourne; ^2^Equine Studies Group, Waltham Petcare Science Institute; ^3^School of Biology and Environmental Science, Queensland University of Technology

**Background:** Hyperinsulinemia is a key risk factor for laminitis in horses. Central and peripheral dopamine concentrations are known to affect insulin regulation in other species, but their role in horses is unclear.

**Objectives:** To determine whether a short‐term reduction in dopamine affects postprandial glucose and insulin responses to a meal containing glucose.

**Animals:** Six adult Standardbred horses.

**Methods:** To suppress dopamine production, horses were treated with alpha‐methyl‐para tyrosine (AMPT), a reversible inhibitor of tyrosine hydroxylase, in a randomized cross‐over design (AMPT 40 mg/kg orally, or placebo). A chaff‐based meal containing glucose powder (1 g/kg BW) was offered, and 14 blood samples were collected over 6 hours. Blood glucose, plasma insulin and prolactin (an indicator of central dopamine production) were measured. Peak responses and area under the curve (AUC) for glucose and insulin were compared using Wilcoxon matched‐paired rank tests.

**Results:** Prolactin concentrations increased following AMPT administration, consistent with decreased dopamine production. The glucose response to feeding was not different when horses were treated with AMPT (p>0.9). However, peak insulin concentration and AUC were both higher following AMPT treatment (both p=0.03).

**Conclusions:** In horses, physiological concentrations of dopamine attenuate the postprandial insulin response to a meal containing glucose. Further studies on dopamine action may help to elucidate the pathophysiology of insulin dysregulation and any link with pituitary pars intermedia dysfunction in horses.

## 337 Abstract E32

### 337.1 The Effect of Trailering on Thyrotropin Releasing Hormone Stimulation of Adrenocorticotropic Hormone Concentration in Horses

#### 337.1.1 Steve Grubbs^1^; **John Haffner** ^2^, DVM; Rhonda Hoffman^3^, MS, PhD

##### 337.1.1.1 ^1^Boehringer Ingelheim; ^2^Associate Professor, Equine Science, Middle Tennessee State University; ^3^Director, Equine Science, Middle Tennessee State University

**Background:** Stress from trailering horses for PPID testing can affect basal ACTH concentrations. The effect of trailering on the TRH stimulation test (T10ACTH) is unknown.

**Objective:** Does trailer stress affect T10ACTH concentration following TRH administration?

**Animals:** Ten adult PPID negative horses were randomized using a 5x5 Latin Square design to avoid effects unrelated to trailering stress.

**Methods:** Randomized prospective study. Two groups of 5 horses hauled 40 minutes every 4 weeks using a 5x5 Latin Square design, rotating through 5 trailer positions and TRH‐stimulation at 0, 15, 30, 60, and 120‐min post‐trailering (PT). Each horse was unloaded at respective post‐trailering times, blood was collected before (T0ACTH) and after (T10ACTH) TRH administration. Blood samples were refrigerated, centrifuged, and plasma frozen (‐80°C) until analysis. A mixed model with repeated measures compared ACTH pre‐ and post‐trailering and after TRH stimulation, using horse as subject and time as repeated effect.

**Results:** Basal ACTH was significantly (p=0.002) elevated (PT0min) compared to pre‐ACTH. Basal ACTH concentrations appeared falsely PPID positive in four horses at PT0min, three horses at PT15min, and three horses at PT30min. Post‐trailering, T10ACTH was not significantly different from pre‐T10ACTH at PT0min or PT15min after unloading whereas PT30min, PT60min and PT120min T10ACTH were significantly (p=0.006) lower. One horse appeared falsely PPID positive at PT0min following TRH administration.

**Conclusions and Clinical Importance:** A 40‐minute trailer ride resulted in multiple false positive results (basal ACTH) for PT30‐min. The T10ACTH does not appear to be affected by trailer stress compared to basal ACTH.

## 338 Abstract E33

### 338.1 A Combined Procedure to Identify Pituitary Pars Intermedia Dysfunction and Insulin Dysregulation in Horses

#### 338.1.1 Steve Grubbs^1^; **John Haffner** ^2^, DVM; Rhonda Hoffman^3^, MS, PhD

##### 338.1.1.1 ^1^Boehringer Ingelheim; ^2^Associate Professor, Equine Science, Middle Tennessee State University; ^3^Director, Equine Science, Middle Tennessee State University

**Background:** Combining thyrotropin releasing hormone (TRH) test and insulin tolerance test (ITT) to diagnose PPID/ID in horses was previously reported using T30ACTH determining PPID status. In North America, T10ACTH is exclusively utilized following TRH administration. Does 10‐minute blood collection for T10ACTH affect the T30 blood glucose concentration when combining the ITT and TRH stimulation test?

**Objective:** This study evaluated combined ITT/TRH stimulation test using T10ACTH instead of T30ACTH.

**Animals:** Ten adult research horses, 4 normal horses, 5 horses with PPID only, 1 horse with PPID/ID.

**Methods:** Randomized prospective study. Horses underwent insulin tolerance test alone, TRH stimulation test alone, then a combined TRH/ITT (TRH/insulin in same syringe) using T10ACTH determining PPID status. Data were tested for normality using Shapiro‐Wilk statistic, then analyzed using a mixed model with repeated measures using horse as subject and treatment as repeated effect. Statistical significance was p≤0.05.

**Results:** In PPID positive horses, TRH/insulin stimulation T10ACTH concentrations were significantly higher (p=0.015) compared to T10ACTH concentrations to TRH only. The T10ACTH concentrations were not different in PPID negative horses stimulated with TRH or combined TRH/insulin. The T30 blood glucose concentrations were not different in PPID‐negative horses stimulated with insulin only or TRH/insulin. In PPID positive horses, T30 glucose concentrations were lower (p=0.05) in insulin only, compared to TRH/insulin.

**Conclusions and Clinical Importance:** The combined procedure did not misclassify PPID or ID status in horses. The combined TRH/ITT test (using T10ACTH) appears to be a valid procedure.

## 339 Abstract E34

### 339.1 Dynamic Insulin Responses to High Carbohydrate Diet Acclimation in Normal and Insulin Dysregulated Horses

#### 339.1.1 **Hailey Maresca‐Fichter** ^1^; Stephanie Valberg^1^, DVM, PhD, DACVIM, ACVSMR; Callum Donnelly^2^, BVBiol/BVSc (Hons 1), DACT, DACVIM (LA); Carrie Finno^2^; Jane Manfredi^1^, DVM, MS, PhD, DACVS‐LA, DACVSMR (Equine)

##### 339.1.1.1 ^1^Michigan State University; ^2^UC Davis

**Background:** Current recommendations for insulin dysregulated (ID) horses are to avoid high starch diets; however, previous work has shown improvements in oral sugar test (OST) insulin responses in normal horses acclimated to sweet feed diets. It is unknown if acclimation to a high carbohydrate diet will affect the results of an OST or a combined glucose‐insulin test (CGIT) in previously diagnosed ID horses.

**Hypothesis:** Acclimation to a high carbohydrate diet will result in improved insulin sensitivity and less marked postprandial hyperinsulinemia.

**Animals:** 17 adult Quarter Horse mares that were determined to be ID (n=9) or normal (n=8) by prior OSTs.

**Methods:** Horses were fed a hay and sweet feed diet (8 g/kg BW of a 25% non‐structural carbohydrate concentrate) for four weeks. A CGIT (150 mg/kg glucose IV, then immediately 0.1 IU/kg insulin IV) followed by a high dose (0.45 ml/kg BW Karo Light Syrup) OST four days later were performed to evaluate glucose and insulin dynamics. Data was analyzed with a McNemar's mid‐p test (significant at P<0.05).

**Results:** There was no correlation between prior OST results and tissue level insulin resistance (11/17 positive) or postprandial hyperinsulinemia (4/17 positive) after the acclimation diet (p=0.73). Six/nine ID horses were OST negative (less postprandial hyperinsulinemia) after acclimation (p=0.07).

**Conclusions and Clinical Importance:** Carbohydrate acclimation does not appear to improve tissue level insulin sensitivity. More work evaluating grain type, duration fed, and OST insulin responses in ID horses should be performed.

## 340 Abstract E35

### 340.1 Myenteric Ganglionitis Underlying Intestinal Motor Dysfunction in 3 Horses Diagnosed with Equine Herpesvirus 1 Post‐mortem

#### 340.1.1 **Catarina G. Barros** ^1^; Nathan Slovis^2^, DVM, DACVIM, CHT; Lutz Goehring^3^; Alan Loynachan^3^; Kathy MacGillivray^2^, DVM, DACVIM; Rana Bozorgmanesh^2^, BSc (Hons), BVetMed (Hons), DACVIM, MRCVS; Udeni Balasuriya^4^; Mariano Carossino^4^

##### 340.1.1.1 ^1^Hagyard Equine Medical Institute; ^2^Internal Medicine, Hagyard Equine Medical Institute; ^3^Gluck Equine Research Center, Veterinary Science, University of Kentucky; ^4^Pathobiological Sciences, School of Veterinary Medicine, Louisiana State University

**Background:** Myenteric ganglionitis (MG) represents an infiltrative neuropathy of the enteric nervous system leading to impaired gastrointestinal motility, frequently associated with long‐term poor prognosis in horses. Equine herpesvirus 1 (EHV‐1) has been detected in the enteric nervous system following cell‐associated viremia and was suggested as a potential underlying cause of MG. In other species, herpesvirus myenteric plexus infection triggers inflammatory cell recruitment with secondary neurodegeneration and development of peristalsis abnormalities.

**Objective:** To illustrate the clinical course of three cases treated for recurrent colic, diagnosed with MG and EHV‐1 infection on post‐mortem exam.

**Case Descriptions and Outcomes:** Three thoroughbred geldings, aged between 4 and 6 years old, were treated for recurrent colic. Clinical signs and disease progression varied despite medical therapy. Two horses were euthanized following acute intestinal rupture. One horse underwent exploratory laparotomy, and after no significant abnormalities were found and continuous clinical deterioration post‐surgery, the horse was humanely destroyed. Post‐mortem examination revealed multifocal MG of diverse intestinal segments in all cases, characterized by a lymphocytic inflammatory infiltrate and myenteric plexus neuronal degeneration. Colon samples, from all cases, were RT‐PCR positive for EHV‐1 wild‐type (non‐neuropathogenic). EHV‐1 immunohistochemistry results were equivocal in all three cases.

**Clinical Importance:** To our knowledge, this is the first case series reporting the concomitant diagnosis of MG and EHV‐1 infection in horses. Further studies are required to investigate underlying pathophysiological mechanisms that relate the occurrence of myenteric plexus inflammation/neurodegeneration and equine herpesvirus infection.

## 341 Abstract E36

### 341.1 Age and Breed Are Associated with the Requirement for Surgical Intervention for Left Dorsal Displacements

#### 341.1.1 **Georgia Dollemore** ^1^; Kate McGovern^2^, BVetMed CertEM (Int. Med.), MS, DACVIM, DECEIM, MRCVS; Rose Tallon^3^, BA, VetMB, MVetMed, DACVIM, MRCVS

##### 341.1.1.1 ^1^Donnington Grove Veterinary Group; ^2^Clinical Director, Equine Internal Medicine, Donnington Grove Veterinary Group; ^3^Equine Associate, Equine Internal Medicine, Donnington Grove Veterinary Group

**Background:** Left dorsal displacements (LDDs) are a common cause of colic seen in referral hospitals. Warmbloods (WBs) are overrepresented. The proportion of horses of each breed requiring surgical or medical treatment is currently unknown.

**Hypothesis/Objectives:** To determine if the requirement for surgical intervention for LDDs is breed specific, and to assess breed differences in pre‐operative parameters. The hypothesis was that young Thoroughbreds (TBs) would be over‐represented in the surgical group.

**Animals:** Client‐owned horses admitted to a UK referral hospital between 2007–2021, with a suspected or confirmed LDD.

**Methods:** A retrospective cross‐sectional study; data was collected from hospital records. T‐tests and Chi‐squared were used for statistical analysis. Horses were included if LDD was highly suspicious on transrectal examination, ultrasonography or confirmed surgically.

**Results:** 161 horses were included; 43 underwent surgery. Of the horses undergoing medical treatment 59 were TBs and 59 were non‐TBs, including 16 WB (27.1%). Of the surgical cases 69.8% were TBs and 30.2% were non‐TBs (p=0.026), (5 WBs). In the TB group, younger age was significantly associated with the need for surgical intervention (p=0.0005). Of the horses undergoing surgery, admission or pre‐operative heart rate, white blood cell concentration, PCV and TP as well as blood and peritoneal lactate were not significantly associated with breed.

**Conclusions and Clinical Importance:** Younger Thoroughbreds were more likely to require surgical correction of LDD when compared to other breeds including WBs. This information is useful for discussions of treatment plans with clients.

## 342 Abstract E37

### 342.1 Fecal Microbiota of Diarrheic Horses and Its Association with Laminitis

#### 342.1.1 **Cosette Ayoub** ^1^; Luis Arroyo^2^, Lic. Med. Vet., DVSc, PhD, DACVIM; Jennifer MacNicol^3^, BSc.H, MSc; David Renaud^4^, DVM, PhD; Scott Weese^5^, DVM, DVSc Guelph, DACVIM; Diego Gomez^6^, DVM, MSc, MVSc, PhD, DACVIM

##### 342.1.1.1 ^1^Ontario Veterinary College; ^2^Associate Professor, Clinical Studies, Ontario Veterinary College; ^3^PhD Candidate, University of Guelph; ^4^Assistant Professor, Population Medicine, Ontario Veterinary College; ^5^Professor, Pathobiology, Ontario Veterinary College; ^6^Assistant Professor, Clinical Studies, Ontario Veterinary College

**Background:** The changes in fecal microbiota associated with dysbiosis during acute equine diarrhea and their link with the development of laminitis are largely unknown.

**Hypothesis/Objectives:** To describe the fecal microbiota of horses with acute diarrhea and to investigate the bacterial communities of diarrheic horses with and without laminitis.

**Animals:** Diarrheic horses (n=55) admitted to a teaching hospital and healthy horses (n=36), both teaching horses and client owned, were sampled. Horses with diarrhea were further divided into those with (n=15) and without (n=39) laminitis.

**Methods:** Unmatched case‐control study. The V4 region of the 16S ribosomal RNA gene was amplified and sequenced. Alpha‐ and beta‐diversity indices were calculated and compared between groups. LefSe analysis was used to determine enriched taxa in each group.

**Results:** The community membership and structure were significantly different between healthy and diarrheic horses (P<0.001) but no differences were detected between laminitic and non‐laminitic horses (P>0.05). The LefSe analysis showed that facultative anaerobes (Enterobacteriaceae, *Lactobacillus*, *Streptococcus* and *Enterococcus*) were enriched in diarrheic horses while healthy horses had an enrichment of obligate anaerobes (Ruminococcaceae and Lachnospiraceae). Diarrheic horses that developed laminitis had an enrichment of *Streptococcus*, *Lactobacillus*, and Enterobacteriaceae.

**Conclusions and Clinical Importance:** The association between diarrhea and an enrichment of facultative anaerobes suggests a potentially important microbial shift that occurs during gastrointestinal inflammation. Changes in fecal microbiota of horses that developed laminitis were similar to those reported in horses with oligofructose‐ and starch‐induced laminitis.

## 343 Abstract E38

### 343.1 Right Dorsal Colitis in Horses: A Retrospective Study of 35 Cases

#### 343.1.1 **Jordan A. Flood** ^1^; Jennifer Bauquier^2^; David Byrne^3^, MVB, MVetClinStud, MVetIntMed, MANZCVS, FHEA; Gustavo Ferlini Agne^4^, MV, MS, DACVIM‐Large Animal; Carlos Medina‐Torres^1^; Allison Stewart^1^, BVSc (Hons I), MS, DACVIM (LAIM), DACVECC, MANZCVS, PhD; Olivia Sullivan^2^; Jessica Wise^5^, BVetBio/BVSc, MANZCVS, DVStud, DECEIM; Kelly Wood^1^, DVM MANZCVS

##### 343.1.1.1 ^1^University of Queensland; ^2^University of Melbourne; ^3^Murdoch University; ^4^University of Adelaide; ^5^Charles Sturt University

**Background:** Right dorsal colitis (RDC) is a non‐steroidal anti‐inflammatory drug (NSAID)‐induced, protein losing enteropathy in horses associated with high mortality. Factors associated with survival have not been reported.

**Objectives:** To describe signalment, NSAID usage, clinical presentations, clinical pathology, ultrasonographic findings, treatments, outcomes, and factors associated with survival in horses diagnosed with RDC.

**Animals:** 35 horses from seven equine referral hospitals definitively or presumptively diagnosed by large animal internal medicine specialists with RDC.

**Methods:** Retrospective case series. Clinical records of RDC cases were submitted by internists. Descriptive data analysis was performed for categorical and continuous variables. Univariate binominal logistic regression was used to assess factors associated with survival. Statistical significance was set at p<0.05.

**Results:** An overdose of NSAIDs occurred in 84% (21/25) cases. Common clinical presentations included diarrhoea (69%; 22/32), colic (61%; 20/33) and tachycardia (53%, 17/32). Common clinicopathological findings included hypoalbuminemia (83.4%; 26/31), hypocalcaemia (79.3%, 23/29) and hyperlactatemia (77.8%, 14/18). The right dorsal colon wall appeared subjectively thickened in 77.4% (24/31) cases using ultrasonography. Mortality rate was 42.9% (15/35). Odds of survival significantly decreased with increasing heart rate (odds 0.84, 95% CI=0.74–0.96, p=0.01), packed cell volume (odds 0.92, 95% CI=0.84–1.00, p=0.04) and abnormal appearance of mucous membranes (odds 0.03, 95% CI=0.003–0.37, p=0.005) on presentation.

**Conclusion:** NSAIDs should be administered within recommended dosage guidelines. Horses receiving NSAIDs should have frequent monitoring of serum/plasma albumin concentration.

## 344 Abstract E39

### 344.1 Ultrasonographic Assessment of Small Intestinal Motility Following Hyoscine Butylbromide Administration in Horses

#### 344.1.1 **Simon Libak Haugaard** ^1^; Kate McGovern^2^, BVetMed, CertEM(Int.Med), MSc, DACVIM, DECEIM, MRCVS; Rose Tallon^3^, MA, VetMB, MVetMed, DACVIM (LAIM), MRCVS; Rachel Gough^4^, MA (Oxon), MA (Cantab), VetMB, MRCVS, CertAVP; Natalia Watrobska^5^, MRCVS

##### 344.1.1.1 ^1^Donnington Grove Veterinary Group; ^2^Hospital Director, Donnington Grove Veterinary Group; ^3^Internal Medicine Clinician, Donnington Grove Veterinary Group; ^4^ECEIM Resident, Donnington Grove Veterinary Group; ^5^Donnington Grove Equine Vets

**Background:** Hyoscine butylbromide (HB) reduces intestinal motility. Horses commonly receive HB prior to hospital admission for colic; this could alter the appearance of the small intestine (SI) on ultrasound scan.

**Hypothesis/Objectives:** We hypothesized that HB administration would lead to reduced SI motility on ultrasound scan as well as increased heart rate (HR).

**Animals:** Client‐owned horses were included with owner's consent. Horses were hospitalised for medical colic and had normal baseline abdominal ultrasound examination.

**Methods:** Ultrasonography was performed in three locations (right inguinal, left inguinal and hepatoduodenal window) before and 1, 5, 15, 30, 45, 60, 90 and 120 minutes after intravenous injection of 0.3 mg/kg HB. Video clips of 30 seconds duration were recorded. Three blinded reviewers assessed SI motility using a subjective grading scale: 1=normal motility, 2=reduced motility but loops contract fully down, 3=reduced motility with incomplete contraction, 4=no motility. Data were analysed with Friedman's test and repeated measures one‐way ANOVA.

**Results:** Six horses were included. Hyoscine butylbromide did not lead to significant differences in SI‐motility grade in any location (P=0.60 left inguinal, P=0.16 right inguinal, P=0.09 duodenum). Some interindividual and interobserver variability was observed, but no horse developed dilated turgid SI‐loops. Heart rate was consistently increased for 45 minutes following HB administration (P<0.01).

**Conclusions and Clinical Importance:** The appearance of dilated turgid SI‐loops common with strangulating intestinal lesions did not develop following HB. Hyoscine butylbromide administered shortly before abdominal ultrasound examination would not be expected to affect clinical decision making.

## 345 Abstract E40

### 345.1 Effect of Post‐Operative Reflux on Survival of Horses with Large Colon Volvulus

#### 345.1.1 **Natalia Watrobska** ^1^; Kate McGovern^2^, DACVIM; Rachel Gough^3^, MA, VetMB, MRCVS, CertAVP; Simon Haugaard^4^, MRCVS

##### 345.1.1.1 ^1^Donnington Grove Veterinary Group; ^2^Clinical Director, Equine Internal Medicine, Donnington Grove Veterinary Group; ^3^Equine Internal Medicine, Donnington Grove Veterinary Group; ^4^Donnington Grove Equine Vets

**Background:** Post‐operative reflux (POR) is rare following a large colon volvulus (LCV), but does occur despite the absence of small intestinal lesions. The prevalence, risk factors and effects on survival of POR after LCV are currently unknown.

**Hypothesis/Objectives:** To determine the prevalence of POR in LCVs and its effect on survival. A further objective was to assess factors which may predict POR. The hypothesis was that horses which produce POR following LCV surgery have a poorer prognosis for survival compared to those that do not.

**Animals:** Client‐owned horses which underwent colic surgery at a single UK referral hospital between 2008–2021, where LCV was the primary finding. Horses with concurrent lesions, and those that did not survive past anaesthetic recovery, were excluded from analysis.

**Methods:** A retrospective cross‐sectional study was performed. Clinical data were retrieved from hospital records. Statistical analyses included chi‐squared and t‐tests. POR was defined as ≥5 litres of gastric reflux produced on at least one occasion.

**Results:** 126 horses were included, 21 of which had POR (16.7%). Overall survival to hospital discharge was 84.9%, 94.3% in the non‐POR and 38.1% in the POR group (P<0.00001). Pre‐operative heart rate, white blood cell count, TP, blood and peritoneal lactate were not associated with POR. Pre‐operative PCV was associated with POR (P=0.028).

**Conclusions and Clinical Importance:** POR in LCVs is a negative prognostic indicator for survival. High PCV pre‐surgery suggests an increased likelihood of POR. Other predictors of POR could not be determined from this study.

## 346 Abstract E41

### 346.1 Post‐mortem Prevalence of Gastric Ulceration in Diarrheic Horses

#### 346.1.1 **Cosette Ayoub** ^1^; Luis Arroyo^2^, Lic. Med. Vet., DVSc, PhD, DACVIM; Mary Brown^3^, HBSc; Rosalie Fortin‐Trahan^4^, DVM, MSc; Diego Gomez^5^, MV. MSc. MVSc. PhD. DACVIM

##### 346.1.1.1 ^1^Ontario Veterinary College; ^2^Associate Professor, Clinical Studies, Ontario Veterinary College; ^3^Research Assistant, Clinical Studies, University of Guelph; ^4^Large Animal Internal Medicine Resident, Clinical Studies, Ontario Veterinary College; ^5^Assistant Professor, Clinical Studies, Ontario Veterinary College

**Background:** Proton pump inhibitors are commonly prescribed to horses with acute diarrhea for the treatment and prevention of equine gastric ulcer syndrome (EGUS). However, the prevalence of EGUS in acute colitis is unknown and evidence supporting acid suppressive treatment is lacking.

**Hypothesis/Objectives:** To investigate the post‐mortem prevalence of EGUS in horses with acute diarrhea.

**Animals:** 224 horses, >1 year‐old, submitted for post‐mortem examination at a university pathology department.

**Methods:** Retrospective study. Review of post‐mortem records of horses that succumbed to acute diarrhea between 2007 and 2020. The following information was recorded: age, breed, sex, diagnosis, and the presence of gastric ulceration.

**Results:** A total of 224 horses (mares=90; males=126 and unknown=8) with an average age of 9±6 years. Most common breeds were Thoroughbreds (50/224), Standardbreds (47/224), Warmbloods (29/224), and Quarter Horses (27/224). Only 19% (42/224) of horses with acute diarrhea had evidence of EGUS with 57% (24/42) of the horses having lesions in the squamous region of the stomach, 33% (14/42) in the glandular, and 5% (2/42) in both regions. In 5% (2/42) of the cases the ulceration location was not reported. Standardbreds and Thoroughbreds represented 50% (21/42) of the horses with EGUS.

**Conclusions and Clinical Importance:** The post‐mortem prevalence of gastric ulceration of diarrheic horses was similar to post‐mortem prevalence reported for the general population. The low prevalence of EGUS in horses that succumbed to acute diarrhea questions the use of proton pump inhibitors in diarrheic horses without confirmed diagnosis.

## 347 Abstract E42

### 347.1 Bacterial Viability in Different Preparation Protocols of Fecal Microbiota Transplantation Solution

#### 347.1.1 **Marcio Costa** ^1^; Rebecca Di Pietro^1^; Julia Arantes^2^; Mélanie Ratté^1^; Mathilde Leclère^1^; Luis Arroyo^3^

##### 347.1.1.1 ^1^University of Montreal; ^2^Universidade de São Paulo (USP); ^3^University of Guelph

**Background:** Fecal microbiota transplantation (FMT) is a therapeutic option for gastrointestinal diseases in horses. Several preparation and conservation protocols to improve bacterial survival have been studied in other species.

**Objectives:** To evaluate a cryoprotectant and the impact of oxygen exposure on bacterial viability of FMT solutions using horse feces.

**Animals:** Ten healthy horses.

**Methods:** Fresh feces were collected in Ziploc bags and transferred into an anaerobic chamber within 10 minutes. Feces from each horse were homogenized, and divided in eight aliquots: four to be analyzed within an hour (fresh) and four to be frozen at ‐20°C for 90 days. 1.8 grams of feces were diluted in 7.2 mL of a cryoprotectant containing nutrients and antioxidants or in 7.2 mL of 10% glycerol. The solution was vortexed for 30 seconds and filtered. Half of the aliquots were processed inside the anaerobic chamber, the other half at room air. Bacterial viability was assessed using flow cytometry. A mixed linear model and the Friedman and Wilcoxon tests were used depending on data distribution.

**Results:** There was no difference of viability between fresh solutions (p=0.16). Viability significantly decreased after one freeze‐thaw cycle (51±27% before, 27±8% after; p<0.001). Glycerol was superior to the cryoprotectant after freezing (32±8% glycerol, 24±8% cryoprotectant; p<0.001). Oxygen exposure did not affect viability (p=0.13).

**Conclusions and Clinical Importance:** Fresh FMT solutions may be better for treatment of horses, but if freezing cannot be avoided, glycerol should be used for dilution of feces.

## 348 Abstract E43

### 348.1 Bacterial Translocation in Horses with Colic Addressed by DNA Sequencing

#### 348.1.1 **Marcio Costa** ^1^; Julia Arantes^2^; Marília Ferreira^2^; Pedro Henrique Brito^2^; Caio Bustamante^3^; Carlos Augusto Valadão^3^; Renata Dória^2^

##### 348.1.1.1 ^1^University of Montreal; ^2^Universidade de São Paulo; ^3^Universidade Estadual de São Paulo

**Background:** Bacterial translocation is the movement of bacteria across the intestinal wall. Colic and intestinal lesions are predisposing factors, but culture‐based techniques are limited in bacterial detection.

**Objectives:** To use next‐generation sequencing to detect bacterial DNA in the peritoneal fluid of horses with colic.

**Animals:** Twenty adult horses requiring laparotomy for large intestinal lesion (colic group) and 4 healthy horses (controls) were enrolled.

**Methods:** Peritoneal fluid collection was performed under general anesthesia, immediately after midline incision for the colic group, and in standing position for controls. Negative controls were collected to exclude environmental contamination. The V4 region of the 16S rRNA gene was sequenced with an Illumina MiSeq. The analysis of molecular variance was used to compare bacterial communities. Samples were also submitted for standard culture.

**Results:** Bacterial DNA was found in all samples**,** while standard culturing method detected bacteria in only one sample**.** Bacterial composition in peritoneal fluid from the colic group was significantly different from controls (p=0.001) and 80% of horses with colic presented similar bacterial composition represented mainly by *Gemella*, *Actinobacillus* and *Bacterioides* spp. All other samples had similar composition to negative controls, indicating absence of bacteria. There was no difference in the composition of samples considering time of evolution, type of lesion (displacement, luminal obstruction, or torsion) or survival.

**Conclusions and Clinical Importance:** Next‐generation sequencing is more sensitive than culturing to detect bacteria in the peritoneal fluid of horses with colic. Molecular methods might be used to target common taxa present during translocation.

## 349 Abstract E44

### 349.1 The Fecal Bacterial Microbiota in Healthy and Sick Neonatal Foals

#### 349.1.1 **Katarzyna Dembek** ^1^; David Wong^2^, DVM, MS, DACVIM, DACVECC; Diego Gomez^3^, DVM, PhD, DACVIM

##### 349.1.1.1 ^1^North Carolina State University; ^2^Department Chair, Iowa State University; ^2^Assistant Professor, University of Guelph

**Background:** The fecal bacterial microbiota of normal foals and foals with enterocolitis has been characterized using next‐generation sequencing technology; however, there are no reports investigating the gut microbiota in foals hospitalized for other perinatal diseases.

**Hypothesis/Objective:** To describe and compare the fecal bacterial microbiota in healthy and sick foals using next‐generation sequencing techniques.

**Animals:** 22 healthy, 18 sick (hospitalized) foals <7 days old.

**Methods:** Case‐control study. Fecal samples were collected from healthy and sick foals on admission. Following DNA extraction, the V4 region of the 16S rRNA gene was amplified using a PCR assay, and the final product was sequenced with an Illumina MiSeq. The relative abundance of predominant taxa, alpha, and beta diversity indices were compared between groups. The LEfSe analysis was used to identify differentially abundant taxa in both groups.

**Results:** Diversity, richness, and evenness were significantly lower in healthy foals than sick foals (p<0.05). LEfSe analysis identified unclassified genera of the family *Lachnospiraceae* to be enriched in healthy foals and the genera *Akkermansia*, *Lactobacillus, Phascolarctobacterium*, *Atopostipes*, and unclassified genera of the phyla Verrucomicrobiota and *Bacteroidetes* to be enriched in sick foals. Differences in bacterial membership (Jaccard index) and structure (Yue & Clayton index) of the fecal microbiota of healthy and sick foal were not detected (AMOVA, P=0.06 and 0.33, respectively).

**Conclusions and Clinical Importance:** Equine neonatal disease is associated with significant alteration to the fecal bacterial microbiota. Different from adult horses, a greater diversity was associated with disease in neonatal foals.

## 350 Abstract E45

### 350.1 Confirmed or Suspected Alloimmune Thrombocytopenia in Mule Foals

#### 350.1.1 **Emily H. Berryhill** ^1^; K. Gary Magdesian^2^, DVM, DACVIM (LAIM), DACVECC, DACVCP

##### 350.1.1.1 ^1^School of Veterinary Medicine, University of California, Davis; ^2^Professor, Medicine and Epidemiology, School of Veterinary Medicine, University of California, Davis

**Background:** Alloimmune thrombocytopenia is a risk for neonatal mule foals as evidenced by experimental induction. There are little data on the prevalence and clinical findings of naturally occurring alloimmune thrombocytopenia in mule foals.

**Hypothesis/Objectives:** To monitor platelet counts in mule foals over time and document development of thrombocytopenia, flow cytometry results, clinical signs and treatment. The hypothesis was that alloimmune thrombocytopenia is common among mule foals, but can be subclinical.

**Animals:** Eighteen neonatal mule foals.

**Methods:** Prospective longitudinal study. Signalment, immunoglobulin source and plasma concentration, clinical signs of and treatment for thrombocytopenia, comorbidities, and serial platelet counts were recorded. Thrombocytopenia was defined as platelet counts <100,000/μL. Results are expressed as median (range).

**Results:** Twelve of 18 foals (67%) developed thrombocytopenia, with lowest platelet counts of 11,000 (4,000–50,000)/μL on day 1–5 of life. Six of 12 thrombocytopenic foals (50%) developed clinical signs consistent with thrombocytopenia, and 3/6 (50%) received specific treatment. Eight of 11 tested thrombocytopenic foals were positive for platelet bound antibodies on flow cytometry. Clinically affected foals had lower platelet counts than those without clinical signs (9,900 μL vs 78,000/μL, *P*=0.01). Platelet counts were documented to return to reference values by 12 (7–50) days. No foals were euthanized or died due to thrombocytopenia.

**Conclusions and Clinical Importance:** Based on this study, approximately 67% of mule foals develop thrombocytopenia; however, up to 50% remain subclinical, even with severe thrombocytopenia. Affected foals have a good prognosis and platelet counts eventually normalized.

## 351 Abstract E46

### 351.1 Optimization of a mRNA Vaccine Candidate to Immunize Foals Against *Rhodococcus equi*

#### 351.1.1 **Rebecca M. Legere** ^1^; Jeannine Ott^2^; Jocelyne Bray^3^; Cameron Martin^4^; Cristina Poveda^5^; Michael Criscitiello^6^; Luc Berghman^7^; Angela Bordin^3^; Jeroen Pollet^5^; Noah Cohen^3^

##### 351.1.1.1 ^1^Texas A&M University; ^2^Department of Veterinary Pathobiology, College of Veterinary Medicine & Biomedical Sciences; ^3^Department of Large Animal Clinical Sciences, College of Veterinary Medicine & Biomedical Sciences; ^4^Department of Poultry Science, College of Agriculture & Life Sciences, Texas A&M University; ^5^Department of Pediatrics, National School of Tropical Medicine, Baylor College of Medicine; ^6^Department of Veterinary Pathobiology, College of Veterinary Medicine & Biomedical Sciences; ^7^Department of Poultry Science, College of Agriculture & Life Sciences, Texas A&M University

**Background:** A vaccine against *Rhodococcus equi* (*R. equi*) is lacking because foals become infected after birth, when maternal antibodies inhibit responses to traditional vaccines. Vaccination with messenger RNA (mRNA) was recently demonstrated to circumvent maternal antibody inhibition and protect neonatal mice against influenza. We aim to develop a mRNA vaccine for foals targeting the virulence‐associated protein A (VapA) of *R. equi*.

**Hypothesis/Objectives:** Expression and secretion of VapA by equine cells will vary significantly among 4 mRNA vaccine constructs encoding: 1) native VapA, including its transmembrane domain (TM); 2) a codon‐optimized version of construct 1; 3) VapA without the TM domain and with an equine leader peptide; and, 4) a codon‐optimized version of construct 3.

**Animals:** Equine bronchial fibroblasts (EBFBs) and equine bronchial epithelial cells (EBECs) cultured from healthy adult horses.

**Methods:** EBFBs and EBECs were transfected with each of the 4 mRNA constructs formulated in lipid nanoparticles and expression of VapA was evaluated by western immunoblot of cell lysates and supernatants using anti‐VapA monoclonal antibody.

**Results:** Constructs lacking the TM domain (3 and 4) were expressed by EBFBs and EBECs. Construct 3 was more highly expressed than construct 4 in cells and supernatants. ELISA results for VapA expression in supernatants are pending.

**Conclusions and Clinical Importance:** Removing the TM domain improved secretion whereas codon optimization did not improve *in vitro* expression. Based on these results, we plan to evaluate the immunogenicity of construct 3 formulated in lipid nanoparticles by either nebulization or intramuscular injection to neonatal foals.

## 352 Abstract E48

### 352.1 Comparison of Microbroth Dilution and Disk Diffusion Methods for Antimicrobial Sensitivity of Equine Bacterial Isolates

#### 352.1.1 **Surita du Preez** ^1^; Sharanne Raidal^1^, BVSc, MVSt, PhD, GradDipEd, FANZCVS; Darren Trott^3^, BSc (Hon), BVMS (Hon), PhD

##### 352.1.1.1 ^1^The University of Adelaide; ^2^Professor, School of Animal and Veterinary Sciences, Charles Sturt University; ^3^Professor, Department of Pathobiology, Infectious Disease & Public Health, School of Animal and Veterinary Sciences, The University of Adelaide

**Background:** Broth dilution techniques, where bacterial isolates are grown in liquid growth media with incremental concentrations of antimicrobials, allow precise determination of the concentration of each antimicrobial that will inhibit bacterial growth *in vitro* (MIC), and hence are better suited to pharmacokinetic/pharmacodynamic approaches to treatment.

**Hypothesis/Objectives:** Determination of MIC using microbroth techniques for equine clinical isolates, and comparison with conventional disk‐diffusion (ADDT) sensitivity testing results.

**Methods:** Antimicrobial sensitivity of bacteria isolated from equine clinical submissions to veterinary diagnostic laboratories at Charles Sturt University and the University of Adelaide were determined using ADDT and Sensititre® microbroth dilution to determine MIC. Resistance profiles were generated for each antimicrobial agent across the range of concentrations tested, using MIC distribution frequency tables, and results were compared between techniques.

**Results:** Sensititre® MIC results were available for 229 isolates tested against twenty commonly used antimicrobials. Multiple drug resistance was observed in 79.5% of isolates (95% CI 73.8–84.2%) compared to 51.7% of isolates based on ADDT, but extreme drug resistance was less commonly recognised. Agreement between MIC and ADDT results was observed on 1505 of 1932 (77.9%) occasions, and was better for gram‐negative isolates (82.9%) than for gram‐positive isolates (74.4%, P<0.001). Conventional results over‐estimated sensitivity for aminoglycosides, but underestimated susceptibility to other antimicrobials tested.

**Conclusions and Clinical Importance:** The current study demonstrated the value of MIC determination of antimicrobial susceptibility for equine patients. High error rates considerably restrict the use of ADDT for determination of antimicrobial sensitivity testing.

## 353 Abstract E49

### 353.1 Impact of the Fecal Microbiome on Subclinical *Salmonella* Shedding in Horses

#### 353.1.1 Emily C. Herring^1^, DVM; Tara Gaire^2^; Jessica Brown^3^; Erin Groover^3^, DVM, DACVIM (LA); Noelle Noyes^2^, DVM, PhD; **Brandy Burgess** ^4^, DVM, PhD, DACVIM (LA), DACVPM

##### 353.1.1.1 ^1^University of Georgia; ^2^Veterinary Population Medicine, University of Minnesota; ^3^Auburn University; ^4^Population Health, University of Georgia

**Background:** Subclinical shedding of *Salmonella* in horses is more common than clinical disease and more likely to go undetected, posing a persistent biosecurity risk to equine facilities. Known risk factors for *Salmonella* positivity suggest that disruptions of the gastrointestinal microflora play a key role in initiating fecal *Salmonella* shedding; elucidating these microbial drivers is critical to developing targeted interventions to prevent transmission of *Salmonella* from subclinical horses.

**Hypothesis/Objectives:** This study aimed to characterize the fecal taxonomic profile of horses with subclinical salmonellosis and correlate changes in *Salmonella* shedding status over time with shifts in the fecal microbiome.

**Animals:** Six adult horses from a resident herd at a veterinary teaching hospital with fecal culture‐confirmed subclinical salmonellosis were included.

**Methods:** Samples from a prospective longitudinal study were selected for retrospective analysis. For each horse, serial *Salmonella* fecal cultures were performed weekly for 8 weeks. DNA was isolated from banked fecal samples (n=48) and subjected to PCR amplification and 16S rRNA amplicon sequencing.

**Results:** Genus‐level alpha diversity did not differ significantly over time or by *Salmonella* shedding status (linear mixed model ANOVA; *P*=0.38, *P*=0.14). Horses that resumed shedding *Salmonella* after ≥4 consecutive negative cultures (considered truly *Salmonella*‐negative) had a simultaneous decrease in the relative abundance of Proteobacteria.

**Conclusions and Clinical Importance:** While microbial diversity of the fecal microbiome does not appear to drive fecal *Salmonella* shedding among subclinical horses, it is possible that a decrease in Proteobacteria creates an ecological niche that allows *Salmonella* to proliferate.

## 354 Abstract E50

### 354.1 Use of CRISPR‐SeroSeq to Detect Multiple *Salmonella* Serotypes in Equine Fecal Samples

#### 354.1.1 Emily C. Herring^1^, DVM; Nikki Shariat^2^, PhD; **Brandy Burgess** ^**2**^, DVM, PhD, DACVIM (LA), DACVPM

##### 354.1.1.1 ^2^University of Georgia; ^2^Population Health, University of Georgia

**Background:** By convention, *Salmonella* shedding status among horses is characterized using a single isolate per sample. However, in a recent longitudinal study, we found that 18% of horses shed multiple serotypes and that the majority of these were subclinically affected, suggesting a healthy gastrointestinal microbiome may restrict proliferation of a single serotype. CRISPR‐SeroSeq is a novel sequencing technology that can identify multiple serotypes in a single sample.

**Hypothesis/Objectives:** We hypothesized that horses with subclinical salmonellosis are more likely to shed more than one serotype compared to horses with clinical salmonellosis.

**Animals:** Thirty‐five *Salmonella*‐positive horses (12 clinical, 23 subclinical) identified through a network of veterinary hospitals in the U.S.

**Methods:** DNA was isolated from frozen *Salmonella‐*positive tetrathionate‐enriched fecal cultures (n=35) derived from a prospective longitudinal study. *Salmonella* CRISPR sequences were PCR‐amplified, barcoded for indexing, and pooled libraries were sequenced to determine serovar diversity.

**Results:** Among 35 horses included in this study, multiple serotypes were detected in enriched cultures from 7 horses (20%). Serotypes most commonly found in combination with others included Oranienburg, Javiana, Newport, and Typhimurium. CRISPR sequences were successfully amplified in 69% of samples (24/35); PCR cycles ranged from 25–28 indicating a low level of *Salmonella* shedding among these horses.

**Conclusions and Clinical Importance:** Horses, particularly those with subclinical infections, can shed multiple *Salmonella* serotypes. CRISPR‐SeroSeq may provide a more sensitive method to identify serotypes shed at low levels within a fecal sample and an opportunity to determine the clinical significance of multi‐serotype infections in horses.

## 355 Abstract E51

### 355.1 MALDI‐TOF Mass Spectrometry for Improved Identification of Equine Bacterial Isolates

#### 355.1.1 **Sharanne L. Raidal** ^1^; Surita du Preez^2^, BVSc, MANZCVS, DECEIM, DVStud; Darren Trott^3^, BSc (Hons), BVMS (Hons), PhD

##### 355.1.1.1 ^1^School of Agricultural, Environmental and Veterinary Sciences, Charles Sturt University; ^2^Senior Lecturer in Equine Medicine, Equine Health and Performance Centre, The University of Adelaide; ^3^Professor of Veterinary Microbiology, Australian Centre for Antimicrobial Resistance Ecology, The University of Adelaide

**Background:** Matrix‐assisted laser desorption ionization time‐of‐flight mass spectrometry (MALDI‐TOF‐MS) is widely used in medical diagnostic settings for rapid (within minutes) identification of bacteria from single colonies or a limited number of clinical specimens (positive blood cultures and urine). There is little information on the use of MALDI‐TOF‐MS for identification of equine bacterial isolates.

**Hypothesis/Objectives:** MALDI‐TOF‐MS would rapidly and accurately identify equine clinical isolates.

**Methods:** Bacteria isolated from clinical submissions to veterinary diagnostic laboratories at Charles Sturt University and the University of Adelaide were cultured and identified routinely on primary submission, and were stored frozen on beads at ‐80°C for subsequent MALDI‐TOF‐MS identification. Agreement between methods was determined at family, genus and species level, and discrepancies were reviewed based on likely effect on clinical outcome.

**Results:** MALDI‐TOF‐MS identification was successful to species level for 309 of 327 (94.5%) isolates (15 could not be identified and 3 were identified only to genus). Phenotypic characterisation extended to species level for only 262 of 337 isolates (77.7%). Agreement was evident between techniques at family level for 91.3% (285/312), at genus level for 87.8% (274/312) and at species level for 77.2% (241/312) of isolates, and was high for bacteria of biosecurity consequence (e.g., *Salmonella* spp., 25/25, 100%; and *Streptococcus equi* ss *equi*, 9/10, 90%).

**Conclusions and Clinical Importance:** MALDI‐TOF‐MS demonstrated improved accuracy for bacterial identification of clinical isolates, performing slightly better for Gram‐negative isolates than Gram‐positive isolates. Cost and time savings afforded by MALDI‐TOF‐MS are substantial.

## 356 Abstract E52

### 356.1 SARS‐CoV‐2 Pseudovirus Infects Equine Bronchial Epithelial Cells *In Vitro*

#### 356.1.1 **Rebecca M. Legere** ^1^; Angelica Allegro^2^; Yvonne Affram^3^; Kelsey Wells^3^; Robert Burghardt^4^; Gus Wright^5^; Jeroen Pollet^6^; Angela Bordin^2^; Paul de Figueiredo^3^; Julian Leibowitz^3^; Noah Cohen^2^

##### 356.1.1.1 ^1^Texas A&M University; ^2^Department of Large Animal Clinical Sciences, College of Veterinary Medicine & Biomedical Sciences, Texas A&M University; ^3^Department of Microbial Pathogenesis and Immunology, College of Medicine, Texas A&M University; ^4^Department of Veterinary Integrative Biosciences, College of Veterinary Medicine & Biomedical Sciences, Texas A&M University; ^5^Department of Veterinary Pathobiology, College of Veterinary Medicine & Biomedical Sciences, Texas A&M University; ^6^Department of Pediatrics, National School of Tropical Medicine, Baylor College of Medicine

**Background:** Severe acute respiratory syndrome coronavirus 2 (SARS‐CoV‐2), the causal agent of COVID‐19, can infect animals by binding to the angiotensin‐converting enzyme 2 (ACE2). Equine infection appears likely because of high homology between human and equine ACE2 and evidence of infection of cell lines transfected to express equine ACE2.

**Hypothesis/Objectives:** Equine bronchial epithelial cells (EBECs) can be infected with a SARS‐CoV‐2 pseudovirus.

**Animals:** Primary EBEC cultures were established from healthy adult horses, and commercially‐sourced human bronchial epithelial cells (HBECs) were used as a positive control.

**Methods:** ACE2 expression by EBECs was demonstrated using immunofluorescence and western immunoblot. EBECs were transduced with a lentivirus pseudotyped with the SARS‐CoV‐2 spike protein that binds to ACE2 and expresses the enhanced green fluorescent protein (eGFP) as a reporter. Cells were co‐cultivated with the pseudovirus at a multiplicity of infection of 1 for 6 hours, washed, and maintained in media for 96 hours. After 96 hours, eGFP expression in EBECs was assessed by fluorescence microscopy of cell cultures and quantitative PCR.

**Results:** Fluorescence microscopy and quantitative PCR results demonstrated pseudovirus infection of EBECs, albeit at lower efficiency than HBECs.

**Conclusions and Clinical Importance:** Equine respiratory tract cells were susceptible to infection with a SARS‐CoV‐2 pseudovirus. Lower replication efficiency in EBECs suggests that horses are unlikely to be an important zoonotic host of SARS‐CoV‐2, but viral mutations could render some strains more infective to horses. Serological and virological monitoring of horses in contact with persons shedding SARS‐CoV‐2 is warranted.

## 357 Abstract E53

### 357.1 Effect of a Supplement Containing Cannabidiol (CBD) on Sedation and Ataxia Scores and Health Parameters

#### 357.1.1 Frank Andrews^1^, DVM, MS, DACVIM (LAIM); Michael St. Blanc^2^, DVM; **Anna Chapman** ^3^, DVM, DACVIM (LAIM); Michael Keowen^4^, BS; Frank Garza^4^, BS, MS; Lydia Gray, DVM

##### 357.1.1.1 ^1^Director Equine Health Studies Program (EHSP), Veterinary Clinical Sciences, Louisiana State University; ^2^Surgery Resident, Equine Health Studies Program, Veterinary Clinical Sciences, Louisiana State University; ^3^Associate Professor, Equine Health Studies Program, Veterinary Clinical Sciences, Louisiana State University; ^4^Research Associate, Equine Health Studies Program, Veterinary Clinical Sciences, Louisiana State University; ^5^Veterinarian, SmartPak, Inc

Supplements containing cannabidiol (CBD) have recently been introduced into the equine market, but few research studies on their safety have been published. The purpose of this study was to determine if a supplement containing CBD would lead to sedation and/or ataxia or changes in other health parameters after being fed for 56 days. Twenty clinically normal horses were housed in stalls for 56 days of the study. On day −1, prior to treatment, horses underwent physical examinations, blood work (CBC and biochemistry panel) and were scored for sedation and ataxia. Horses were then randomly divided into treatment (CBD, 150 mg in supplement pellets top‐dressed on a grain ration once daily [n=10]) and control (CTR; [n=10]) groups. Horses were observed daily and scored weekly for sedation and ataxia, 2 hours after feeding, by 2 masked observers using validated scoring systems. A CBC and biochemical panel were performed on days 28 and 56, 2 hours after administration. The supplement was readily consumed, and no adverse effects were observed. Sedation and ataxia scores were near zero in most horses during the weekly exams. There were no treatment effects on blood values, including anemia and blood proteins, liver enzymes, kidney values, or electrolytes and calcium. Body weight significantly increased in all of the horses by day 56 of the study, but a significant treatment by day effect was not observed. The supplement containing CBD (150 mg) did not cause sedation, ataxia or negative effects on health or blood parameters during 56 days of treatment.

## 358 Abstract E54

### 358.1 Computed Tomographic Myelography in Horses with Cervical Vertebral Compressive Myelopathy

#### 358.1.1 **Sonia Gonzalez‐Medina** ^1^; Tawfik Aboellail^2^, DVM, DACVP, PhD; Myra Barrett^3^, DVM, DACVR, MS, CSU; Brad Nelson^4^, DVM, PhD, DACVS; Yvette Nout‐Lomas^5^, DVM, MS, DACVIM‐LA, DACVECC, PhD

##### 358.1.1.1 ^1^Colorado State University; ^2^Associate Professor, Veterinary Pathology, Colorado State University; ^3^Associate Professor, Veterinary Diagnostic Imaging, Colorado State University; ^4^Equine Surgery, Colorado State University; ^5^Associate Professor, Equine Medicine, Colorado State University

**Background:** Radiographic myelography underestimates the presence of CVCM, mainly due to lack of three‐dimensional assessment of the spinal cord, which is possible using CT myelography.

**Hypothesis/Objectives:** Establish morphometric measures of cervical spine structures in sound and CVCM horses. We hypothesize that focal cervical vertebral canal and spinal cord dimensions are smaller in horses with CVCM and that CT myelography detects fewer and sometimes other compressed sites.

**Animals:** Five neurologically sound and 9 CVCM horses were used. Hospital population. IACUC approval through protocol 16‐6674AA.

**Methods:** Case‐control study from hospital‐based population. All horses underwent a neurologic examination, radiographic and CT myelography, and post‐mortem evaluation. Myelographic dorsal column and dural and spinal cord height and areas were compared between sound and CVCM horses, and between compressed and non‐compressed sites.

**Results:** There were no sites of spinal cord compression identified via CT myelography that had not been detected by radiographic myelography. Spinal cord and dural areas were consistently smaller in CVCM horses, but these were statistically significant only for C5–C6 (p=0.02) and C6–C7 (p=0.04). The ratio of full myelographic area at the intervertebral space to vertebral body height of either cranial or caudal body were significantly smaller in the CVCM group (p<0.001).

**Conclusions:** CT‐myelography was not superior to radiographic myelography for diagnosing CVCM in this population. CVCM horses had smaller spinal cords than control horses. Using a ratio of full myelographic area compared to vertebral body height is useful for diagnosing spinal cord compression.

## 359 Abstract E55

### 359.1 Echocardiographic Assessment of Fluid‐Responsiveness in Critically Ill Newborn Foals: A Pilot Study

#### 359.1.1 **Francesca Freccero** ^1^; Giovanni Romito^2^, DVM, PhD, DECVIM‐CA (Cardiology); Carolina Beato^3^, DVM; Jole Mariella^4^, DVM, PhD; Aliai Lanci^5^, DVM, PhD; Nicola Ellero^6^, DVM; Mario Cipone^7^, DVM; Carolina Castagnetti^7^, DVM, PhD, DECAR

##### 359.1.1.1 ^1^University of Bologna; ^2^Assistant Professor, University of Bologna; ^3^Private Practitioner; ^4^Associate Professor, University of Bologna; ^5^Assistant Professor (Junior), University of Bologna; ^6^PhD Student, University of Bologna; ^7^Full Professor, University of Bologna

**Background:** Intravenous fluid requirements are difficult to estimate accurately on a clinical basis in critically ill newborn foals (CINF). Transthoracic echocardiography may help monitoring fluid responsiveness in these patients.

**Hypothesis/Objectives:** To study echocardiographic changes before and after a replacement fluid therapy in CINF.

**Animals:** Prospective observational study. Eight CINF less than three days old suffering of various neonatal diseases (sepsis, neonatal syndrome, prematurity) were enrolled.

**Methods:** On admission, unsedated CINF underwent a complete (M‐mode, two‐dimensional and Doppler) transthoracic echocardiography obtained from a right parasternal and subcostal views before any treatment. Conventional techniques/formulas were applied for the estimation of left ventricular volumes, fractional shortening, ejection fraction, stroke volume and cardiac output. An identical echocardiographic assessment was repeated at the end of a fluid challenge (Ringer's lactate, 10–20 ml/kg boluses). Comparison of echocardiographic parameters obtained before and after fluid therapy was made using a Wilcoxon signed rank test. A p‐value<0.05 was considered statistically significant.

**Results:** In all foals, initial echocardiographic variables overall indicated volume depletion. After fluid therapy (1–4 boluses), a statistically significant increase of left atrial size, left ventricular volumes, stroke volume and cardiac output was documented (Table 1). Remaining echocardiographic changes did not change significantly.

**Table:** Table 1. Significant echocardiographic variables obtained in critically ill newborn foals before (T0) and after (T1) a fluid challenge therapy (one to four 10–20 ml/kg boluses of Lactated Ringer's solution)

Parameter	T0	T1	p‐value
LAD_La_ (cm)	4.8	5.7	0.0078
EDV_B_ (mL)	93.1	100.4	0.0313
SV_Ao_ (mL)	89.3	160.7	0.0313
CO_Ao_ (L/min)	9.7	15.2	0.0313
CO_M_ (L/min)	5.9	8.6	0.0234

As all data were not normally distributed, they were presented as median values.

CO_Ao_ and CO_M_: cardiac output assessed by the aortic‐flow based method and M‐Mode echocardiography, respectively; EDV_B_: left ventricular end‐diastolic volume assessed by the bullet method; LAD_La_: maximal left atrial diameter obtained from a right parasternal long axis view; SV_Ao_: stroke volume assessed by the aortic‐flow based method.

**Conclusions and Clinical Importance:** In CINF, many echocardiographic variables change after fluid bolus therapy, overall indicating a correction of volume depletion and an improvement of stroke volume and cardiac output. Integrating transthoracic echocardiography in the pre‐ and post‐fluid therapy assessment of CINF may allow a better evaluation of fluid responsiveness and optimal titration.

## 360 Abstract E56

### 360.1 Pain Scoring Systems for Predicting Clinical Outcomes in Hospitalized Equine Ophthalmology Patients

#### 360.1.1 **Dayna R. Jodzio**; Sally DeNotta, DVM, PhD, DACVIM (LAIM); Caryn Plummer, DVM, DACVO; Chris Sanchez, DVM, PhD, DACVIM (LAIM)

##### 360.1.1.1 College of Veterinary Medicine, University of Florida

**Background:** Pain recognition in hospitalized horses is difficult. Although pain scoring has recently gained recognition for a variety of equine conditions, the utility of pain scoring in equine ophthalmology patients is poorly described.

**Hypothesis/Objectives:** To evaluate the Horse Grimace Scale (HGS) and Behavior Pain Scores (BPS) in hospitalized equine ophthalmology patients. We hypothesized that temporal trends in HGS and BPS would predict clinical outcome.

**Animals:** Privately‐owned horses hospitalized for ocular disease between September 2018 and September 2020.

**Methods:** Prospective observational study. Horses were assigned HGS and BPS scores daily throughout hospitalization. Outcomes were defined as surgery (e.g., keratectomy, corneal graft, etc.), enucleation, or discharge with medical management only. Temporal trends in HGS and BPS were assessed using linear regression. Correlations between slope, intercept, and outcome were determined by Kruskal‐Wallis test.

**Results:** Of the 46 horses that met the criteria for inclusion in the analysis, 9 (20%) had surgery, 8 (17%) underwent enucleation, and 29 (63%) were discharged following medical management. BPS scores at admission were greater in horses that were managed medically than those that underwent enucleation (P=0.012). Horses requiring enucleation displayed a greater rise in HGS (P=0.018) and BPS (P=0.012) during hospitalization than horses that were discharged, and a greater rise in BPS (P=0.039) than horses requiring surgery.

**Conclusions and Clinical Importance:** Pain scoring may represent a useful tool for predicting clinical outcomes in hospitalized equine ophthalmology patients.

## 361 Abstract E57

### 361.1 Effects of Equine Platelet Lysate on *Ex Vivo* Vasculogenesis

#### 361.1.1 **Mariano Mora‐Pereira** ^1^; Lindsey Boone^2^, DVM, PhD, DACVS‐LA; Maria Naskou^3^, DVM, PhD, DACVP; Anne Wooldridge^4^, DVM, MS, PhD, DACVIM (LAIM)

##### 361.1.1.1 ^1^Auburn University; ^2^Associate Professor Equine Surgery and Sports Medicine, Clinical Sciences, Auburn University; ^3^Assistant Professor of Clinical Pathology, Clinical Sciences, Auburn University; ^4^Professor, Clinical Sciences, Auburn University

**Background:** Regenerative vasculogenesis is a promising treatment for ischemic conditions in horses; however, preservation of endothelial cell phenotype and function in culture is challenging.

**Objectives:** Determine the effect of different concentrations of equine platelet lysate (ePL) on vasculogenesis. Equine‐specific growth factors in ePL will enhance vasculogenesis in an *ex vivo* arterial ring model.

**Animals:** Six horses for ePL harvesting. Facial arteries were dissected from 5 horses following euthanasia.

**Methods:** Arterial rings were embedded in Matrigel® and randomly assigned to different growth medias: 10xePL, 5xePL, 2xePL, HS (horse serum), PPP (platelet‐poor plasma) or EBM (endothelial basal media). Photomicrographs from days 0–3 were analyzed for quantification of branches, density and vascular network area (VNA). Supernatant vascular endothelial growth factor (VEGF) concentration was determined by ELISA on day 3. Means were compared among groups at each timepoint by repeated measures ANOVA. Growth rate and VEGF effect were determined by linear regression. P<0.05 was significant.

**Results:** Treatment and time had a significant effect on VNA (p=0.0238), and area growth rate differed between groups (p=0.0234). Rings in the 10xePL and 5xePL groups trended toward higher branch number and density; however, differences were not significant. For all groups, there was a significant increase in all parameters over time (p<0.0001). On day 3, VEGF concentration was significantly different between groups (p=0.0351) and had a positive effect on VNA (p=0.0063).

**Conclusions and Clinical Importance:** Equine‐specific growth factors in ePL have a positive effect on vascular growth. This could be an alternative source of growth factors, improving progenitor cell culture.

## 362 Abstract E58

### 362.1 Transdermal EMLA (Lidocaine/Prilocaine) Cream for Intravenous Catheterisation in Horses

#### 362.1.1 **Bianca O. Amiet**; Wendy Goodwin, BVSc, PhD, FANZCVS; Jo Rainger, BVSc, PhD, FANZCVS; Allison Stewart, BVSc, MS, DACVIM‐LAIM, DACVECC; Solomon Woldeyohannes, BSc, MPH, PhD; Steven Zedler, VMD, DACVS

##### 362.1.1.1 University of Queensland

**Background:** Eutectic mixture of local anaesthetics (EMLA) is a lidocaine/prilocaine cream that has been used in the management of pain associated with intravenous (IV) catheterisation in humans and animals. EMLA cream has been demonstrated to be as effective as lidocaine in reducing superficial pain perception in horses.

**Hypothesis/Objectives:** It was hypothesised that there would be no difference in aversive behavioural reactions in response to IV catheterisation following either treatment. Additionally, it was hypothesised that application of EMLA cream would result in reduced aversive behavioural reactions compared to lidocaine infiltration.

**Animals:** Twenty‐six horses from the University of Queensland School of Veterinary Science Research Herd were enrolled in the study.

**Methods:** Jugular sites of 26 horses were randomly assigned to be treated with 1 g/cm^2^ of 5% EMLA cream for 60 minutes prior to IV catheter placement. The contralateral side was treated with 30 mg (1.5 mL) of subcutaneous lidocaine. A simple descriptive scale was used to score behavioural reactions between 0–3 by blinded observers.

**Results:** Mean behavioural reaction scores to treatment with EMLA cream and lidocaine were 0.3 (±0.3) and 0.6 (±0.5), respectively. Mean behavioural reaction scores to catheterisation following treatment with EMLA cream and lidocaine were 0.8 (±0.5) and 0.4 (±0.5), respectively.

**Conclusions and Clinical Importance:** Lidocaine is commonly used to desensitise the jugular catheterisation site in horses, however, may cause transient pain on injection. EMLA cream represents a less invasive alternative and may offer clinically relevant desensitisation of the skin.

## 363 Abstract E59

### 363.1 Pharmacokinetics of Single‐Dose Administration of 20 and 40 mg/kg of Acetaminophen in Neonatal Foals

#### 363.1.1 **Jenifer R. Gold** ^1^; Tamara Grubb^2^, DVM, PhD, DACVAA; Lais Malavasi^3^, DVM, PhD; Michael Court^4^, BVSc, PhD, DACVAA; Nicolas Villarino^5^, Vet Med, DVSc, PhD, DACVP

##### 363.1.1.1 ^1^Washington State University; ^2^Adjunct Faculty, Veterinary Clinical Sciences, Washington State University; ^3^Assistant Professor, Veterinary Clinical Sciences, Washington State University; ^4^Professor, Veterinary Clinical Sciences, Washington State University; ^5^Associate Professor, Veterinary Clinical Sciences, Washington State University

**Introduction:** Pain control in equine neonates is typically controlled with the use of nonsteroidal anti‐inflammatory drugs (NSAIDs). Adverse effects such as renal papillary necrosis and gastrointestinal ulceration can occur with these drugs; therefore, alternatives for neonatal foals would be ideal. Acetaminophen is commonly used in human medicine. It does not appear to cause adverse effects in adult horses and older foals, but the pharmacokinetics and if acetaminophen is safe or efficacious in neonatal foals is unknown.

**Objective:** The objective was to determine the pharmacokinetics of acetaminophen following oral administration of a single dose of 20 mg/kg and 40 mg/kg to neonatal foals.

**Animals:** Eight clinically healthy 6–8‐day old foals.

**Methods:** In a randomized clinical study foals received a single oral dose of acetaminophen either 20 or 40 mg/kg. Complete blood count and biochemistry profiles were performed before and seven days after each dose administration. Blood samples were collected one time before and then 9 times after acetaminophen administration for 48 hours to quantify plasma acetaminophen concentrations. Plasma pharmacokinetic parameters were estimated using compartmental analysis.

**Results:** Peak plasma concentrations occurred at 1.3 hours and 1.5 hours for 20 and 40 mg/kg. The maximum plasma concentration was 12 ug/ml and 14 ug/ml for 20 and 40 mg/kg respectively. The median AUC0‐∞ ranged from 45.1 to 100 and 77 to 216 h*ug/mL for the 20 and 40 mg/kg dose respectively. All bloodwork remained within normal limits.

**Conclusion:** Further studies are needed to establish a safe/effective dose for treating foals of different ages.

**Table:** Table 1. Plasma pharmacokinetic parameters (median [range]) derived from a 2‐compartmental model with first‐order absorption for acetaminophen in foals after a single oral administration at 20 and 40 mg/Kg of body weight.

PK parameter	Units	20 mg/Kg	40 mg/Kg
K01	1/h	1.4 (0.4–2.3)	2.2 (0.7–4.3)
K01_HL	h	1.0 (0.3–1.6)	0.4 (0.2–0.8)
K10	1/h	0.2 (0.1–0.3)	0.2 (0.1–0.3)
K10_HL	h	6.7 (2.1–14)	4.6 (3.3–6.7)
K12	1/h	n/a	0.1 (0.0–0.2)
K21	1/h	n/a	1 (0.0–0.3)
Tmax	h	1.3 (0.5–2.3)	1.5 (0.7–1.9)
Cmax	ug/mL	12 (7.5–18)	14 (12–18)
AUC	h*ug/mL	74 (45.1–100)	130 (77–216)

AUC0‐∞= area under the plasma concentration‐time curve from 0 hours to infinity after dosing; Cmax = maximum concentration; Tmax = time to maximum concentration, K01 = absorption rate constant; K01_HL = half‐life of absorption; K10 = terminal rate constant; K10_HL = half‐life of terminal portion of the curve after oral administration. K12 and K21 are the distribution and redistribution rate constants. N/a = not applicable. PK parameters for 2 horses in the 20 mg/Kg dose were calculated using noncompartmental analysis because no pharmacokinetic model fit their acetaminophen disposition.

## 364 Abstract E60

### 364.1 Detection of Oxycodone Metabolites in Plasma After Oral Administration to Horses

#### 364.1.1 **Joanne E. Haughan** ^1^; Jaclyn Missanelli^2^, BS; Mary Robinson^2,3^, PhD, VMD, DACVCP; Youwen You^2^, PhD

##### 364.1.1.1 ^1^Equine Pharmacology Lab, Penn Vet, New Bolton Center; ^2^PA Equine Toxicology & Research Laboratory; ^3^Director, Clinical Studies, PennVet Equine Pharmacology Laboratory

**Background:** Oxycodone, a μ‐agonist commonly prescribed to alleviate pain in humans, has been detected in plasma and urine samples collected for equine post‐race drug testing. Previous work has shown that oxycodone is rapidly absorbed and eliminated in horses resulting in short plasma detection times.

**Hypothesis/Objectives:** We hypothesized that metabolites of oxycodone would achieve higher concentrations for longer periods in equine plasma, allowing for longer detection of oxycodone administration.

**Animals:** 12 healthy horses (6 Thoroughbreds, 6 Standardbreds, aged 8.6±2.7 years) from the institutional research herd were used.

**Methods:** Oxycodone (5 mg) was administered orally to each horse and plasma samples were collected for up to 96 hours. Oxycodone, oxymorphone and noroxycodone plasma concentrations were measured using validated LC‐MS/MS methodology with a lower limit of quantification (LLOQ) of 5 pg/mL, 25 pg/mL and 25 pg/mL, respectively. The following parameters were compared: Cmax (maximum plasma concentration); Tmax (time at Cmax); Clast (last concentration >LLOQ) and Tlast (Time at Clast).

**Results:** Cmax, and Tlast were higher for oxymorphone and lower for noroxycodone when compared to oxycodone (Table 1). Oxymorphone plasma concentrations remained above LLOQ longer than oxycodone or noroxycodone (Figure 1). Median Tlast for oxymorphone (Median: 20 h range: 16–24 h) was much higher than oxycodone (Median: 8 h; range 6–16 h). In contrast, noroxycodone Tlast was much lower (Median: 4 h; range 0.75–6 h).

Figure 1. Mean plasma concentration over time of oxycodone, oxymorphone and noroxycodone after administration of 5 mg oxycodone orally to 12 horses

**Figure:**
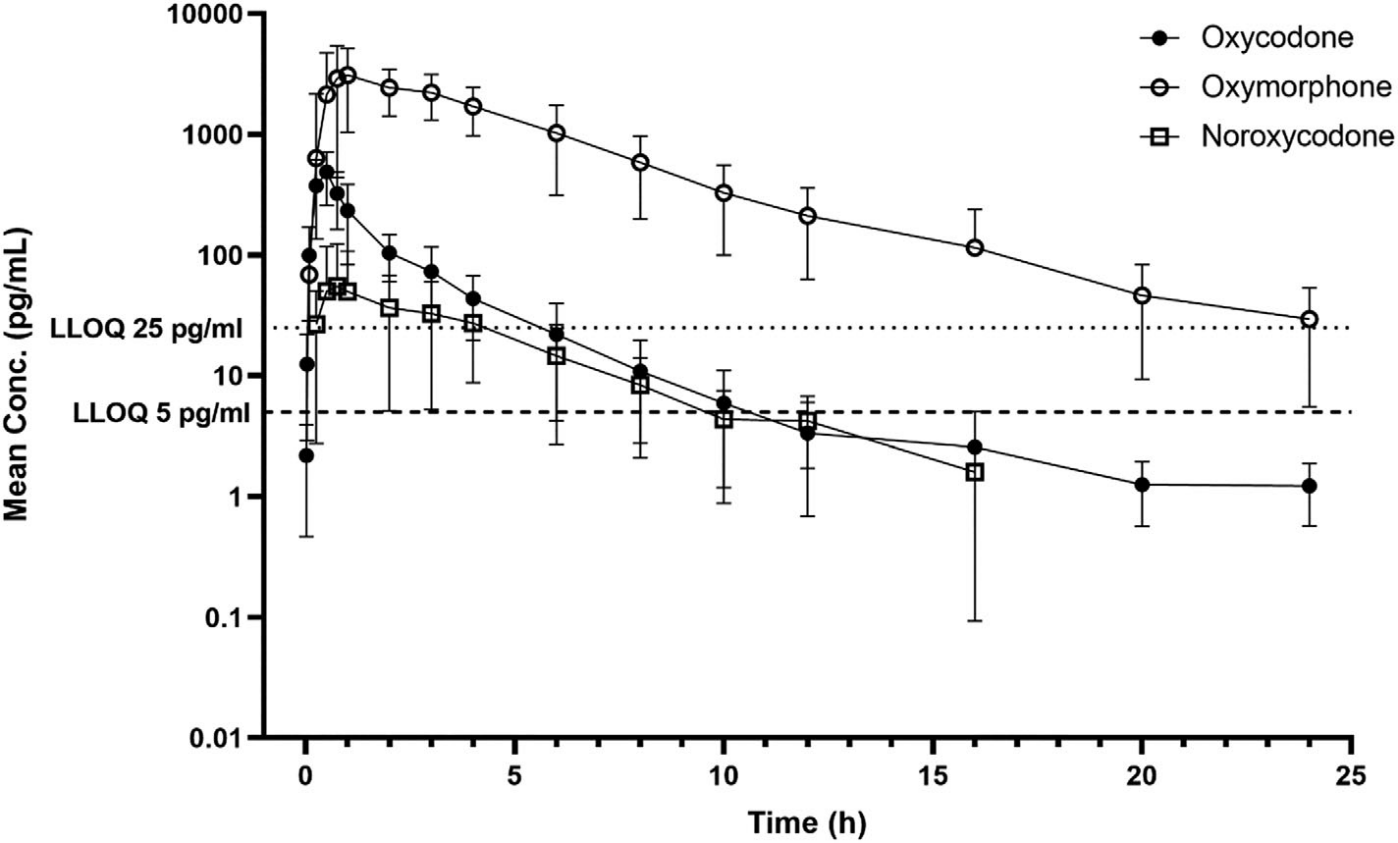
LLOQ is 5 pg/ml for oxycodone (dashed line) and 25 pg/ml for oxymorphone and noroxycodone (dotted line). Error bars show standard deviation from the mean at each time point.

**Table:** Table 1. Plasma concentration over time parameters of oxycodone, oxymorphone and noroxycodone after administration of 5 mg oxycodone orally to 12 horses

Parameter	**Oxycodone** **(LLOQ 5 pg/ml)**	**Oxymorphone** **(LLOQ 25 pg/ml)**	**Noroxycodone** **(LLOQ 25 pg/ml)**
**Cmax (pg/ml)**	525.5 (191–836)	3,635 (1,600–9,150)	52.5 (0–250)
**Tmax (h)**	0.5 (0.25–0.75)	1 (0.5–3)	0.75 (0–4)
**Clast (pg/ml)**	7.96 (5.31–11.9)	37.35 (28.8–75.2)	27.9 (25.2–37.9)
**Tlast (h)**	8 (6–16)	20 (16–24)	4 (0.75–6)

Median values are given with range in parentheses.

Cmax: maximum plasma concentration (pg/ml); Tmax: time at Cmax (h); Clast: last concentration >LLOQ; Tlast: Time at Clast (h).

**Conclusion:** This is the first study to evaluate oxycodone metabolites in horses. Identification of oxymorphone in equine plasma will enable regulatory bodies to detect oral oxycodone administration to horses for a longer time period.

## 365 Abstract E61

### 365.1 Short‐Term Clinical Outcome and Racing Performance of Thoroughbreds Treated with Enrofloxacin as Juveniles: 146 Cases

#### 365.1.1 **Natasha Williams** ^1^; Nimet Browne^1^, DVM, MPH, DACVIM (LAIM); Jared Taylor^2^, DVM, MPH, PhD, DACVIM (LAIM), DACVPM; Nathan Slovis^1^, DVM, DACVIM (LAIM)

##### 365.1.1.1 ^1^Hagyard Equine Medical Institute; ^2^Oklahoma State University

**Background:** Despite its extended spectrum of activity and widespread use in adult horses, adverse effects of enrofloxacin, including chondrotoxicity, have precluded its use in juveniles.

**Objectives:** To evaluate the short‐term outcome of juvenile and neonatal Thoroughbreds treated with enrofloxacin (5 mg/kg IV), and evaluate the racing performance of survivors.

**Animals:** 146 juvenile Thoroughbreds

**Methods:** Retrospective case series. Cases were divided into 3 age groups for the purposes of analysis: 12–24 months (group 1); 6–12 months (group 2); and 0–6 months (group 3). Racing records for surviving animals and maternal siblings were examined.

**Results:** Median treatment duration was 5 days (range 1–63). 17/146 (11.6%) cases were ascribed one or more complications attributed to treatment, including synovitis (n=8), tendon laxity (10), lameness (3), and anaemia (2), with complications occurring on median day 4 of treatment (range 2–5).107/146 (73%) cases survived to discharge. Survivors did not differ from siblings with regard to percentage of starters for all groups. Number of wins was significantly lower for foals from group 3 compared to their siblings; however, no significant difference was observed between cases and siblings in groups 1 and 2. Earnings per start were significantly lower compared to siblings for foals in groups 2 and 3.

**Conclusions:** Surviving juveniles receiving enrofloxacin were as likely to start races as their untreated siblings, but age <6 months at treatment resulted in fewer starts and lower earnings. These outcomes are similar to that reported for NICU survivors.

## 366 Abstract E62

### 366.1 Electrical Impedance Tomography Can Determine Airflow in the Respiratory System of Healthy Adult Horses

#### 366.1.1 **David Byrne** ^1^; Giselle Hosgood^2^, BVSc (Hons), MS, PhD, FACVSc, DACVS; Ben Keeshan^3^, PhD; Andy Adler^4^, PhD; Martina Mosing^5^, Dr. Med. Vet., PhD, DECVAA

##### 366.1.1.1 ^1^Murdoch University; ^2^Professor of Small Animal Surgery, Murdoch University; ^3^Postdoctoral Researcher, Systems and Computer Engineering, Carleton University; ^4^Professor, P.Eng, Systems and Computer Engineering, Carleton University; ^5^Senior Lecturer in Veterinary Anaesthesia, Murdoch University

**Background:** Electrical impedance tomography (EIT) is a non‐invasive method of evaluating lung function.

**Objective:** To compare respiratory flow variables calculated from thoracic EIT measurements with corresponding spirometry variables.

**Animals:** Ten healthy Thoroughbred and Standardbred research horses.

**Methods:** The horses were sedated and instrumented with spirometry and a single‐plane EIT electrode belt around the thorax. Horses were exposed to sequentially increasing volumes of dead space between 1000 and 8500 ml, in 5 to 7 steps, to induce carbon dioxide rebreathing, until clinical hyperpnoea or a tidal volume of 150% baseline was reached. A two‐minute stabilisation period followed by two minutes of data collection occurred at each timepoint. Breathing pattern based on the total impedance curve, inspiratory and expiratory times, peak inspiratory and expiratory flow, and nadir expiratory flow were evaluated with EIT and spirometry. Bland‐Altman analysis was used to evaluate the agreement. Results are given as the mean bias expressed as a proportion [95% confidence interval] of the ratio of EIT to spirometry.

**Results:** Sedated horses intermittently exhibited a breathing pattern with incomplete expiration in between breaths (**crown‐like** breaths). There was good agreement between EIT‐derived and spirometry‐derived inspiratory (‐0.0615 [‐0.2535–0.1799]) and expiratory (0.0467 [‐0.0895–0.2033]) times and peak inspiratory (‐0.1529 [‐0.456–0.3191]) and expiratory (‐0.0961 [‐0.3193–0.2003]) flows. Agreement for nadir flow estimates was insufficient (‐0.2237 [‐0.8716–3.6921]).

**Conclusions and Clinical Importance:** Electrical impedance tomography can quantify airflow variables over increasing tidal volumes, when compared with spirometry in standing sedated horses.

## 367 Abstract E64

### 367.1 An Updated Description of Bacterial Pneumonia in Adult Horses and Factors Associated with Non‐Survival

#### 367.1.1 **Kimberly Hallowell** ^1^; Katarzyna Dembek^2^, DVM, PhD, DACVIM (LAIM); Kate Hepworth‐Warren^3^, DVM, DACVIM (LAIM)

##### 367.1.1.1 ^1^North Carolina State University; ^2^Assistant Professor of Equine Internal Medicine, Department of Clinical Sciences, North Carolina State University; ^3^Assistant Clinical Professor, Department of Clinical Sciences, North Carolina State University

**Background:** Available studies on equine pneumonia describing common bacteria, antimicrobial susceptibility, and factors associated with survival are outdated or focus on specific horse or bacterial populations.

**Hypothesis/Objectives:** To describe the clinical presentation and bacterial isolates of horses with bacterial pneumonia and identify factors associated with non‐survival.

**Animals:** 113 horses, >2 years old, with bacterial pneumonia.

**Methods:** Retrospective case series. Data regarding history, physical examination, clinicopathologic features, bacterial culture and sensitivity, treatment, and outcome were collected.

**Results:** *Streptococcus equi* subspecies *zooepidemicus* was the most commonly isolated bacteria (50%), followed by *Klebsiella* spp. (19.7%), other *Streptococcus* species (17.7%), *Escherichia coli* (16.6%), and *Bacillus* spp. (14.5%). *Streptococcus equi* ssp. *zooepidemicus* isolates had good susceptibility (95.1–100%) to all antibiotics tested aside from trimethoprim sulfa (66.7%). *Escherichia coli* and *Klebsiella* isolates showed reduced susceptibility to gentamicin (70.6%, 66.7%), enrofloxacin (87.5%, 50%), and sulfa drugs (47.1%, 55.6%). Survival to discharge was 71.1%. Tachycardia (OR 23.12, CI 95% 2.96–587.73) and elevated creatinine (OR 13.30, CI 95% 2.22–213.03) increased risk of non‐survival. Increasing albumin (OR 0.04, CI 95% 0.00–0.30) and lymphocyte count (OR 0.20, CI 95% 0.04–0.63) were protective against non‐survival.

**Conclusions and Clinical Importance:** Although bacterial isolates were similar to historical studies, rates of antibiotic susceptibility for *Escherichia coli* and *Klebsiella* spp. were lower than previously reported. Changing antimicrobial susceptibility patterns may influence empiric treatment decisions. The increased risk of non‐survival associated with tachycardia and azotemia is consistent with previous studies in horses and can be utilized in initial case assessment.

## 368 Abstract E65

### 368.1 Utility of Serum Amyloid A in Monitoring Response to Antimicrobial Therapy in Equine Pneumonia

#### 368.1.1 **Kate L. Hepworth‐Warren** ^1^; Krista Estell^2^, DVM, DACVIM (LAIM); Bobby Cowles^3^, DVM, MS, MBA; Deborah Amodie^4^; Mark Crisman^5^, DVM, MS, DACVIM (LAIM)

##### 368.1.1.1 ^1^College of Veterinary Medicine, North Carolina State University; ^2^Clinical Assistant Professor, Virginia Tech's Marion duPont Scott Equine Medical Center; ^3^Managing Veterinarian, Equine Technical Services, Zoetis; ^4^Associate Director, Outcomes Research, Zoetis; ^5^Senior Veterinarian, Equine Technical Services, Zoetis

**Background:** Serum amyloid A (SAA) is a major acute‐phase protein in horses but has not previously been monitored over the course of bacterial pneumonia and may provide a useful tool for assessing response to antimicrobial therapy.

**Objectives:** To monitor SAA in response to antimicrobial therapy and identify associations between SAA, WBC count and fibrinogen in bacterial pneumonia of adult horses.

**Animals:** 18 adult horses with bacterial pneumonia.

**Methods:** Prospective clinical study. Horses hospitalized with bacterial pneumonia were enrolled and SAA and vital parameters were assessed daily. SAA was measured utilizing a handheld meter. CBC and fibrinogen were assessed on Days 0, 1, and 2, then every 3 days until discharge. Antimicrobial therapy, results of transtracheal wash cultures, and ultrasonographic findings were also recorded. Data was assessed with a 2‐sided t‐test. Least square (LS) means that were not normally distributed were log‐transformed and significance was set at p≤0.05.

**Results/Findings:** Mean SAA on Day 0 was 1126 μg/mL (range 0–2929 μg/mL). Horses were hospitalized for a mean of 9.3 days (range 4–17 days). SAA was significantly higher on Day 1 (p=0.036), Day 2 (p=0.0016), and Day 3 (p=0.0114) when compared to Day 6. SAA peaked at Day 2 (mean 1059 μg/mL) and decreased until discharge. Fibrinogen and WBC did not change significantly from Day 1 to 6.

**Conclusions and Clinical Importance:** SAA changed significantly over the course of treatment as compared to WBC and fibrinogen and appeared to correlate with clinical improvement of pneumonia.

## 369 Abstract E67

### 369.1 Comparative Efficacy and Adverse Effects of Salbutamol and N‐Butylscopolammonium Bromide in Horses with Severe Asthma

#### 369.1.1 **Berta Mozo Vives** ^1^; Jean‐Pierre Lavoie^2^, DVM, DACVIM; Sophie Mainguy‐Seers^2^, DVM, DACVIM

##### 369.1.1.1 ^1^Veterinary University of Montreal; ^2^Department of Clinical Sciences, Veterinary University of Montreal

**Background:** Salbutamol and N‐butylscopolammonium bromide (NBB) are bronchodilators commonly used to relieve bronchoconstriction in horses with severe asthma (SEA). As these horses might be more susceptible to the cardiovascular effects of NBB because of right ventricular dysfunction during exacerbation, determining the comparative efficacy of both drugs is warranted.

**Objective:** To compare the bronchodilation potency and duration of salbutamol and NBB in SEA.

**Animals:** Six horses in exacerbation of SEA from a research herd were studied.

**Methods:** The effects of inhaled salbutamol (1000 mg) and NBB (150 mg, IV) were assessed in a randomized blinded cross‐over experiment with a 3‐day washout period. Intestinal borborygmi, heart rate, and lung function (resistance and reactance measured using oscillometry) were assessed before and 5, 10, 15, 30, 60, 90, 120 and 180 minutes after administration and analyzed with a mixed effect analysis and Dunnett's multiple comparison tests.

**Results:** Both treatments resulted in a significant improvement in lung function. Pulmonary resistance and reactance returned to baseline values within 30 minutes after NBB administration, while salbutamol improved reactance until 180 minutes (mean improvement of 0.045 Kpa/L/s, 95% CI=0.004–0.086; p=0.023 of the reactance at 5 Hz). The heart rate increased, and gut sounds decreased within 30 minutes of NBB administration only.

**Conclusions and Clinical Importance:** Both drugs have a similar bronchodilator effect in SEA, but with a longer duration for salbutamol. Digestive and cardiovascular effects were noted only with NBB, suggesting the preferential use of salbutamol to relieve bronchoconstriction in horses with asthma.

